# *LAMA4*-Regulating miR-4274 and Its Host Gene *SORCS2* Play a Role in *IGFBP6*-Dependent Effects on Phenotype of Basal-Like Breast Cancer

**DOI:** 10.3389/fmolb.2019.00122

**Published:** 2019-11-08

**Authors:** Maxim Shkurnikov, Sergey Nikulin, Stepan Nersisyan, Andrey Poloznikov, Shan Zaidi, Ancha Baranova, Udo Schumacher, Daniel Wicklein, Alexander Tonevitsky

**Affiliations:** ^1^National Medical Research Radiological Center, Ministry of Health of the Russian Federation, Obninks, Russia; ^2^Far Eastern Federal University, Vladivostok, Russia; ^3^Faculty of Mechanics and Mathematics, Lomonosov Moscow State University, Moscow, Russia; ^4^School of Systems Biology, George Mason University, Fairfax, VA, United States; ^5^Research Center of Medical Genetics, Moscow, Russia; ^6^Institute of Anatomy and Experimental Morphology, University Cancer Center, University Medical-Center Hamburg-Eppendorf, Hamburg, Germany; ^7^Faculty of Biology and Biotechnologies, Higher School of Economics, Moscow, Russia; ^8^Shemyakin-Ovchinnikov Institute of Bioorganic Chemistry RAS, Moscow, Russia; ^9^Art Photonics GmbH, Berlin, Germany

**Keywords:** miRNA, TCGA, breast cancer, intragenic miRNA, laminins

## Abstract

Specificity of RNAi to selected target is challenged by off-target effects, both canonical and non-canonical. Notably, more than half of all human microRNAs are co-expressed with hosting them proteincoding genes. Here we dissect regulatory subnetwork centered on *IGFBP6* gene, which is associated with low proliferative state and high migratory activity of basal-like breast cancer. We inhibited expression of *IGFBP6* gene in a model cell line for basal-like breast carcinoma MDA-MB-231, then traced secondary and tertiary effects of this knockdown to *LAMA4*, a laminin encoding gene that contributes to the phenotype of triple-negative breast cancer. *LAMA4*-regulating miRNA miR-4274 and its host gene *SORCS2* were highlighted as intermediate regulators of the expression levels of *LAMA4*, which correlated in a basal-like breast carcinoma sample subset of TCGA to the levels of *SORCS2* negatively. Overall, our study points that the secondary and tertiary layers of regulatory interactions are certainly underappreciated. As these types of molecular event may significantly contribute to the formation of the cell phenotypes after RNA interference based knockdowns, further studies of multilayered molecular networks affected by RNAi are warranted.

## 1. Introduction

MicroRNAs constitute one of the largest families of non-coding RNAs and are known to regulate the proliferative and migratory activity of tumor cells (Makarova et al., 2015). According to the current estimates, microRNAs regulate expression of more than 60% of protein-coding genes (Makarova et al., 2016). An increase in levels of certain microRNA may lead to a decrease in mRNA levels of their target genes and *vice versa*. In turn, this may lead to significant alterations of cell phenotypes (Galatenko et al., 2018a).

RNA interference (RNAi) is a widely used tool for specific downregulation of expression of a certain target gene. Its central assumption, namely, its specificity to selected target, has been challenged by commonly detected off-target effects, driven both by “seed” sequence based annealing of shRNA to the genes encoding related proteins of same family (Jackson et al., 2006) or even to unrelated mRNA sequences, which may be targeted by either canonical or noncanonical interactions (Birmingham et al., 2006; Seok et al., 2018).

Notably, microRNA encoding genes may be arranged not only as independent transcription units but also within the introns and exons of both protein-coding and non-coding RNA genes (Makarova et al., 2016). More than half of all human microRNAs are co-expressed with the proteincoding genes and are located within their introns (Hinske et al., 2010, 2014). Previously, we have demonstrated the regulatory interactions between the protein-coding genes which host certain miRNAs and the protein-coding target genes of these miRNA (Galatenko et al., 2018a).

In this paper we concentrate on a dissection of a particular regulatory subnetwork centered on *IGFBP6* gene, which is associated with low proliferative state and high migratory activity of basal-like breast cancer (Poloznikov et al., 2019). Previous studies suggest that RNAi-guided downregulation of *IGFBP6* gene expression leads to an elevation in the expression of genes encoding miR-100 and miR-let-7a (Poloznikov et al., 2019). Surprisingly, known targets of the above-mentioned microRNAs include *IGF2, IGF1R*, and *INSR* genes, known as drivers of the process of metastasis (Nikulin et al., 2018). In other words, suppression of anti-migratory gene *IGFBP6* may take place on two different levels, direct and indirect, with the latter being realized through miRNA based suppression of *INSR/IGF1R* 1 axis of pro-metastatic signaling (Poloznikov et al., 2019).

To explore putative miRNA-based co-regulation followed by the suppression of the *IGFBP6* gene, we inhibited expression of this gene in MDA-MB-231 cells, a model cell line for basal-like breast carcinoma, by shRNA, then we have traced secondary and tertiary effects of this knockdown to *LAMA4*, a laminin encoding gene that contributes to the phenotype of triple-negative breast cancer.

## 2. Materials and Methods

### 2.1. Cell Culture

The MDA-MB-231 cells were cultivated in standard 25 cm^2^ plates (TPP, Switzerland) in DMEM medium containing 4.5 g/l glucose (Gibco, USA), 10% ETS (Gibco), 2 mM L-glutamine (PanEco, Russia), penicillin (100 units/ml) and streptomycin (100 μg/ml) (Gibco). The media was changed every 2–3 days.

### 2.2. RNAi

For RNAi-mediated knockdown of the *IGFBP6* gene, the MDA-MB-231 cells were transduced with lentiviral expression vector pLVX-shRNA1 (Clontech Laboratories, USA) carrying the following shRNA-encoding sequence: 5′-gatccGCCCAATTGTGACCATCGATCGATTCAAGAGATCGATCGTCACAATGGGGCTTTTTTTACGCGTg-3′). The control cell line was transduced with the same vector encoding shRNA targeting firefly (*Photinus pyralis*) luciferase gene. The production of lentiviral particles, transduction, and selection procedures were performed according to the protocol described earlier (Knyazev et al., 2015). Phenotypic characteristics of resultant MDA-MB-231 cell line with stable knockdown of *IGFBP6* were described in our earlier work (Nikulin et al., 2018). The levels of endogenous expression of *IGFBP6* in this stable cell line are at 22% of that in luciferase expression control.

### 2.3. RNA Extraction and Analysis of Expression

Before RNA isolation, cells were suspended in 2.5 ml of culture media, plated onto 6-wells plates (2 × 10^5^ cells/ml) and incubated for 48 h at 37° C in 5% CO_2_. Afterward, the cells were washed three times with cold FSB solution (PanEco) and lysed in Qiazol Reagent (Qiagen, Germany). RNA was isolated with miRNeasy Mini Kit (Qiagen) according to the manufacturer's protocol. The concentrations and the quality of the purified RNA were assessed using both Nanodrop ND-1000 spectrophotometer (Thermo Fisher Scientific, USA) and Agilent Bioanalyzer 2100 (Agilent Genomics, USA) (Fomicheva et al., 2017). A260/A280 ratios were in range of 1.92.2. Each RNA sample was isolated from independently grown flask, with three flasks per cell type undergoing mRNA extraction. RNA quality controls were performed as described in Krainova et al. (2013). The RNA integrity number (RIN) values were higher than 9.0.

Procedures for the preparation of total RNA samples for hybridization on Human Transcriptome Arrays 2.0 microarrays (Affymetrix, USA) were carried out according to the manufacture protocol as described in Khaustova et al. (2017) and Kudriaeva et al. (2017). cDNA synthesis was performed using 500 ng total RNA as the starting material. Hybridization of fragmented and labeled cDNA on microarrays, array washing, staining, and scanning were performed as described in Sakharov et al. (2012). Experiment has been repeated in three biological replicates. Scans of the microarrays were converted into CEL files using the scanner software, and were then processed in Transcriptome Analysis Console 4.0.1.36 according to the summation method Gene + Exon - SST-RMA (Analysis Version 2) and the ebayes statistics; respective *p*-values were calculated after Benjamin-Hochberg correction for multiple tests. The fold changes in gene expression were shown using a log2 scale.

GSEA analyses were performed the Pathway Studio environment (www.pathwaystudio.com, accessed in Sep 2019).

### 2.4. Bioinformatics Analysis

The bioinformatics data analysis was carried out in R statistical environment according to the following algorithm:

1. Genes, which have changed their expression 2-fold or larger (with FDR *p* ≤ 0.01) after *IGFBP6* knockdown were selected;
2. Among those genes, the genes encoding microRNAs were further determined (Hinske et al., 2014);
3. For each intragenic miRNA identified at Step 2, miRTarBase 7.0 (Chou et al., 2017) was utilized to compile the list of its target genes;
4. Target lists were analyzed to identify any target genes with expression changed of more than four-fold (at FDR *p* ≤ 0.01);
5. The directionality of the expression changes of each host gene to each target gene was explored in triple-negative breast cancer sample subset of TCGA database (*N* = 190), accessed on July 28th, 2019.

## 3. Results

Transcriptional knockdown of *IGFBP6* gene in MDA-MB-231 cells resulted in inhibition of cell migration with concomitant increase in the rate of proliferation (Nikulin et al., 2018). A comparison of *IGFBP6* knockdown and the control population of cells revealed a significant change in expression levels of 380 genes (FDR *p* ≤ 0.01, fold change ≥ 2). Among those, 183 genes were downregulated, while the expression of other 197 genes was increased. Enrichment analysis revealed that “Mesenchymal Cell Differentiation” as the most altered Molecular Function, along with a predominance of “Cell Adhesion” and “Cell Communication” centered gene lists among the most altered biological processes (Supplementary Table 1). When analyses of upregulated and downregulated genes were performed separately, cellular and molecular functions performed by upregulated components were minimally perturbed when compared to that of entire list of genes with altered expression (Supplementary Table 2). Surprisingly, Top cellular component perturbed by downregulation was “vesicle,” while the Top of the list of downregulated biological processes was dominated by various aspects of tissue morphogenesis (Supplementary Table 3).

Importantly, a total of 29 upregulated genes were co-located with various microRNAs (Table 1). These co-located intragenic miRNA loci encode a total of 35 pre-miRNAs and 41 different mature microRNAs.

**Table 1:** The list of 15 microRNA host genes with the most prominent expression changes observed in MDA-MB-231 cells after shRNA knockdown of *IGFBP6*.

**Gene**	**Expression levels in IGFBP6 KO cells, log2**	**Expression levels in control cells, log2**	**Fold change, log2**	***P*-value after multiple test correction**	**Pre-micro-RNA**
SORCS2	5.3 ± 0.18	9.6 ± 0.58	−4.32	2.03 × 10^−11^	hsa-mir-4798, hsa-mir-4274
SYTL3	9.4 ± 0.54	5.12 ± 0.06	4.25	5.02 × 10^−14^	hsa-mir-3918
F13A1	4.7 ± 0.26	8.16 ± 0.91	−3.45	4.18 × 10^−8^	hsa-mir-7853, hsa-mir-5683
DMD	8.2 ± 0.29	4.8 ± 0.15	3.36	2.78 × 10^−12^	hsa-mir-3915, hsa-mir-548f-5
CEMIP	4.8 ± 0.3	8.12 ± 0.68	−3.32	2.17 × 10^−7^	hsa-mir-549a
CACHD1	10.1 ± 0.3	6.9 ± 0.14	3.22	1.22 × 10^−13^	hsa-mir-4794
ARRB1	6.5 ± 0.2	9.74 ± 0.47	−3.20	2.45 × 10^−10^	hsa-mir-326
DOCK10	11.6 ± 0.14	8.54 ± 0.11	3.01	9.21 × 10^−10^	hsa-mir-4439
IRAK3	8.8 ± 0.34	6.01 ± 0.13	2.81	3.95 × 10^−12^	hsa-mir-6502
PDCD4	13.2 ± 0.07	10.41 ± 0.61	2.81	9.74 × 10^−8^	hsa-mir-4680
HLA-B	6.6 ± 0.51	9.38 ± 0.56	−2.80	5.72 × 10^−6^	hsa-mir-6891
ROBO1	10.1 ± 0.15	7.34 ± 0.15	2.76	1.96 × 10^−12^	hsa-mir-3923
CLU	5.8 ± 0.15	8.52 ± 0.33	−2.73	1.15 × 10^−9^	hsa-mir-6843
SCP2	9.3 ± 0.09	11.96 ± 0.37	−2.69	8.43 × 10^−12^	hsa-mir-1273f, hsa-mir-5095, hsa-mir-1273g
UGCG	10.1 ± 0.07	12.79 ± 0.17	−2.66	2.85 × 10^−13^	hsa-mir-4668

**Figure 1:**
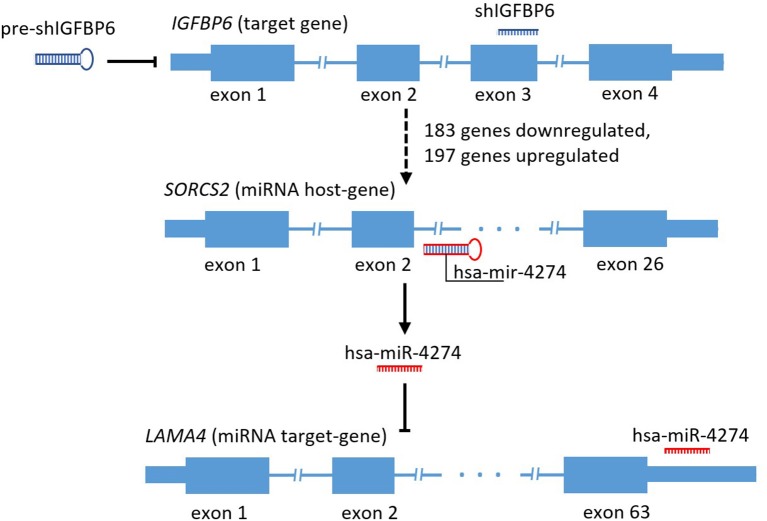
Scheme reflecting traced subset of the secondary and tertiary effects of *IGFBP6* knockdown in MDA-MB-231 cells.

Mining of miRTarBase 7.0 database (Chou et al., 2017) for possible interactions of these 41 microRNAs resulted in a total of 3,429 miRNA-target gene interactions cataloged. For each of these target genes, gene expression changes were assessed by applying the threshold of FDR *p* ≤ 0.01 and at least 4-fold change of both miRNA host and miRNA target genes. According to these criteria, there was only one host/target gene pair with significant decrease of miRNA host and concomitant increase of respective target gene expression, namely, *SORCS2* as a host (a decrease of 4.32-fold) and *LAMA4* as a target (an increase of 5.46 fold). The regulator connecting *LAMA4* and other target genes was hsa-miR-4274 encoded by the second intron of *SORCS2*. Figure 1 shows a scheme of *SORCS2* gene with miR-4274 location.

Taking into account that interactions between hsa-miR-4274 and *LAMA4* gene has been detected in course of PAR-CLIP (Whisnant et al., 2013) experiment only, secondary analysis of the host and target genes expression was performed in the basal-like breast cancer dataset of 190 samples retrieved from TCGA and annotated using the pam50 classifier (geneFu package).

**Figure 2:**
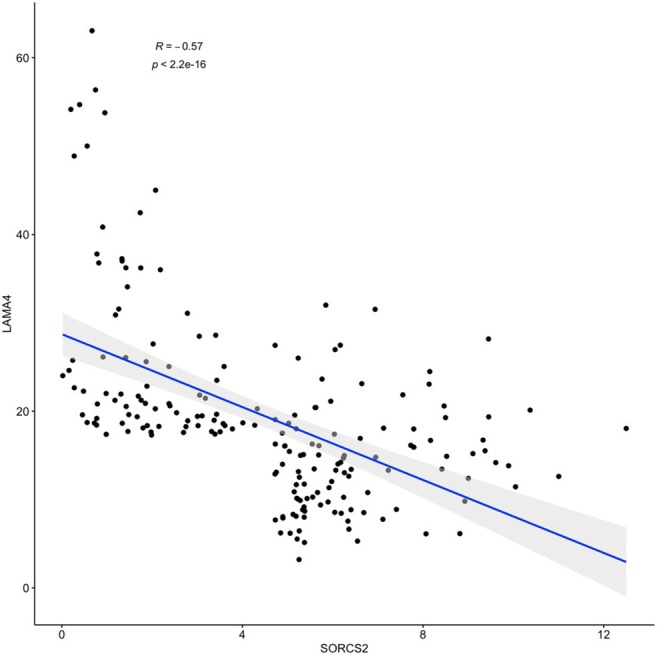
A negative correlation of observed expression levels of *SORCS2* and *LAMA4* genes in basal-like breast cancer samples; rpkm. Filtering: *SORCS2* and *LAMA4* at 90th percentile or higher.

The results of *SORCS2* and *LAMA4* genes co-expression analysis are shown in Figure 2. Notably, in basal-like breast carcinoma subdataset of TCGA, expression levels of these two genes negatively correlate with each other (Spearman's *R* = −0.57, *p* < 2.2 × 10^−16^).

## 4. Discussion

miRNAs are well-known modulators of gene expression, playing their roles both at the level of the transcripts and through epigenetic gene silencing. Although significant progress has been made in understanding functions of individual miRNA, mechanisms regulating their own transcription remain elusive. Speaking generally, intragenic miRNAs are expected to be co-expressed with their host genes, displaying either synergistic or antagonistic correlation patterns. Because of that, shRNA based suppression of the activity of individual gene, which hosts miRNA in its intron, is expected to change expression pattern for both the host gene and the miRNA it harbors.

*IGFBP6* gene, a well-known suppressor of IGF-II signaling, suppresses proliferation and stimulates apoptosis in a wide range of cells. On the other hand, in some tumor cells, including those of rhabdomyosarcoma and basal types of breast cell carcinoma (Poloznikov et al., 2019), it induces migration, likely by an IGF-independent mechanism. Respectively, depending on particular cell type, effects of *IGFBP6* may be classified as that of a tumor suppressor, or a facilitator of the metastatic spread. Thus, understanding the physiological role of *IGFBP6* protein may be context-dependent and requires thorough evaluation of the relative balance of its IGF-dependent and IGF-independent actions.

In a previous study, shRNA based suppression of *IGFBP6* in basal-like breast cancer cell line MDA-MB-231 led to a notable inhibition of cell migration along with an increase in its rate of proliferation (Poloznikov et al., 2019). GSEA analysis showed that knockdown of IGFBP6 profoundly changes expression programs related to cell adhesion and cell communication, while sparing machinery involved in propagation of the cell cycle. Among pathways most enriched by downregulated mRNAs were ones directly involved in tissue morphogenesis, and in the formation of vesicles, including exosomes. These effects were accompanied by the elevation in the expression of a metastasis-suppressing miR-100 and miR-let-7a (Poloznikov et al., 2019). These two observations may be tied together by the hypothesis of intracellular accumulation of miRNAs due to suppression of their transport into intercellular space and/or circulation.

Further dissection of the transcription program enacted in response to the treatment with *IGFBP6* shRNA revealed concerted changes in the levels of many transcripts lacking canonical complementarity to its intended target. Many previous works have previously acknowledged detrimental miRNA-like effects of shRNA (Anderson et al., 2008; Seok et al., 2018), including unwanted toxicities, and their clear potential for misleading an interpretation of resultant phenotypes. In this paper, we show that off-target effects of a transcriptional knockdown of *IGFBP6* are multilayered. After knockdown of *IGFBP6*, a total of 380 genes change their expression significantly, representing the first layer of the off-target complexity. As many of these genes (*N* = 29) hosted various intragenic pre-microRNA (Table 1), we attempted to uncover a second regulatory layer by embarking on a further analysis of the putative targets of these miRNA. Indeed, analysis of coordinated changes in expression of the host genes along with the targets for miRNAs embed in these genes revealed a number of candidate pairs. Expression levels of the strongest co-regulated gene pair, *SORCS2* as a host and *LAMA4* as a target, correlated negatively (*R* = −0.57, *p* < 2.2 × 10^−16^), when analyzed in a basal-like breast carcinoma sample subset of TCGA (*N* = 190) (Figure 2).

Interestingly, gene variant located in a vicinity of *SORCS2* (rs4234798) demonstrated genome-wide significant associations with circulating concentrations of *IGFBP-3* and *IGF-I* (Kaplan et al., 2011; Teumer et al., 2016), previously described as a prognostic marker in basal-like triple-negative breast cancer (Marzec et al., 2015; Julovi et al., 2018), and as a part of 19-gene expression signature which substantially outperforms Dukes' classification predicting the survival of patients with colorectal cancer (Aziz et al., 2016). Protein encoded by *SORCS2* gene coordinates intracellular transport of the glutamate and cysteine transporter *EAAT3* (Malik et al., 2019) in the neurons, where it provides a degree of protection from oxidative stress and epilepsy-induced cell death (Shkurnikov et al., 2013). Given that expression of *SORCS2* mRNA is highly restricted to the nervous system and the kidneys, it is tempting to speculate that the function of relatively high levels of SORCS2 expression observed in basal-like breast carcinomas should be attributed to intragenic miRNA hsa-miR-4274.

As many other miRNAs, miR-4274 is capable of binding to many different mRNAs. One of its confirmed targets, the *LAMA4* mRNA, encodes an α4-chain of laminins, the secreted heterotrimeric glycoproteins involved in the induction of the epithelial-mesenchymal transition. Elevation of *LAMA4/LAMA5* protein ratio correlates with an increase in the permeability of the basal membrane as well as the metastatic and invasive potential of colorectal cancer (Galatenko et al., 2018b). Elevated level of *LAMA4* expression as well as two other chains of laminin 411 is observed in metastatic lymph nodes of prostate cancer patients in comparison with metastasis-free lymph nodes (Shkurnikov et al., 2018). In breast carcinomas, an increase in *LAMA4* expression marks the transition from pre-malignant lesions to malignant carcinomas, while in well-developed tumors an overexpression of *LAMA4* mRNA is associated with shorter relapse-free survival (Ross et al., 2015). Specifically, in basal-like triple negative breast carcinoma cells, the levels of both *LAMA4* mRNA and laminin-α4 are elevated when compared to that in adjacent normal tissue samples. Moreover, knockdown of *LAMA4* inhibits proliferation, migration and invasion of these cancer cells *in vitro* (Maltseva and Rodin, 2018; Yang et al., 2018). Taken together, observations concerning *LAMA4*, its regulating miRNA miR-4274 and its host gene *SORCS2*, point at a substantial role played by secondary and tertiary effects produced by a transcriptional knockdown of *IGFBP6* in basal-like breast carcinoma cells.

Overall, our study shows that the secondary and tertiary layers of regulatory interactions are certainly underappreciated. This study is limited in its scope, as it studies results of *IGFBP6* knockdown in only one model cell line rather than in a panel of cells of various tissue origins. Our findings were validated only in a specific type of human tumor, basal-like triple negative breast carcinoma and, therefore, cannot be generalized to all cancers. Moreover, preliminary data point that negative correlation of expression levels for genes *SORCS2* and *LAMA4* is a phenomenon which is, indeed, specific to only this tumor type. While it is tempting to speculate that strict cell type specificity of observed phenomenon may inform future therapy for triple negative breast cancer, detailed studies of *SORC2/LAMA4* relationship are warranted. Similarly, further studies of multilayered molecular networks to be uncovered by deconvoluting of miRNA mediated correlations in gene expression patterns should be performed in order to improve our understanding of the formation of cell phenotypes after RNA interference based knockdowns.

## Data Availability Statement

Publicly available datasets were analyzed in this study. This data can be found here: https://www.cancer.gov/tcga.

## Author Contributions

MS, AP, AB, and AT contributed conception and design of the study. MS and SNe performed the statistical analysis. SZ performed enrichment analysis. MS wrote the first draft of the manuscript. SNi, AB, US, DW, and AT wrote sections of the manuscript. All authors contributed to manuscript revision, read and approved the submitted version.

### Conflict of Interest

AT was employed by the company art photonics GmbH. The remaining authors declare that the research was conducted in the absence of any commercial or financial relationships that could be construed as a potential conflict of interest. The handling editor declared a past co-authorship with several of the authors MS, AB, and AT.

## References

[B1] Anderson E. Birmingham A. Baskerville S. Reynolds A. Maksimova E. Leake D.. (2008). Experimental validation of the importance of seed complement frequency to sirna specificity. RNA 14, 853–861. 10.1261/rna.704708 18367722 PMC2327361

[B2] Aziz N. Mokhtar N. Harun R. Mollah M. Rose I. Sagap I.. (2016). A 19-gene expression signature as a predictor of survival in colorectal cancer. BMC Med. Genom. 9:58. 10.1186/s12920-016-0218-1 27609023 PMC5016995

[B3] Birmingham A. Anderson E. Reynolds A. Ilsley-Tyree D. Leake D. Fedorov Y. (2006). 3′ utr seed matches, but not overall identity, are associated with rnai off-targets. Nat. Methods 3, 199–204. 10.1038/nmeth854 16489337

[B4] Chou C.-H. Shrestha S. Yang C.-D. Chang N.-W. Lin Y.-L. Liao K.-W.. (2017). miRTarBase update 2018: a resource for experimentally validated microrna-target interactions. Nucl. Acids Res. 46, D296–D302. 10.1093/nar/gkx1067 29126174 PMC5753222

[B5] Fomicheva K. Osip'yants A. Knyazev E. Samatov T. Shkurnikov M. (2017). Detection of potential metastatic prostate cancer circulating biomarkers by comparison of mirna profiles in du145 cells and culture medium. Bull. Exp. Biol. Med. 162, 792–796. 10.1007/s10517-017-3715-0 28429232

[B6] Galatenko V. Galatenko A. Samatov T. Turchinovich A. Shkurnikov M. Makarova J.. (2018a). Comprehensive network of miRNA-induced intergenic interactions and a biological role of its core in cancer. Sci. Rep. 8:2418. 10.1038/s41598-018-20215-5 29402894 PMC5799291

[B7] Galatenko V. Maltseva D. Galatenko A. Rodin S. Tonevitsky A. (2018b). Cumulative prognostic power of laminin genes in colorectal cancer. BMC Med. Genom. 11(Suppl. 1):9. 10.1186/s12920-018-0332-3 29504916 PMC5836818

[B8] Hinske L. C. França G. S. Torres H. A. M. Ohara D. T. Lopes-Ramos C. M. Heyn J.. (2014). miRIAD—integrating microrna inter- and intragenic data. Database 2014:bau099. 10.1093/database/bau099 25288656 PMC4186326

[B9] Hinske L. Galante P. Kuo W. Ohno-Machado L. (2010). A potential role for intragenic mirnas on their hosts' interactome. BMC Genomics 11:533. 10.1186/1471-2164-11-533 20920310 PMC3091682

[B10] Jackson A. L. Burchard J. Schelter J. Chau B. N. Cleary M. Lim L.. (2006). Widespread siRNA off-target transcript silencing mediated by seed region sequence complementarity. RNA 12, 1179–1187. 10.1261/rna.25706 16682560 PMC1484447

[B11] Julovi S. Martin J. Baxter R. (2018). Nuclear insulin-like growth factor binding protein-3 as a biomarker in triple-negative breast cancer xenograft tumors: effect of targeted therapy and comparison with chemotherapy. Front. Endocrinol. 9:120. 10.3389/fendo.2018.00120 29623068 PMC5874320

[B12] Kaplan R. Petersen A.-K. Chen M.-H. Teumer A. Glazer N. Döring A.. (2011). A genome-wide association study identifies novel loci associated with circulating igf-i and igfbp-3. Hum. Mol. Genet. 20, 1241–1251. 10.1093/hmg/ddq560 21216879 PMC3043664

[B13] Khaustova N. A. Maltseva D. V. Oliveira-Ferrer L. Stürken C. Milde-Langosch K. Makarova J. A.. (2017). Selectin-independent adhesion during ovarian cancer metastasis. Biochimie 142, 197–206. 10.1016/j.biochi.2017.09.009 28919578

[B14] Knyazev E. Nyushko K. Alekseev B. Samatov T. Shkurnikov M. (2015). Suppression of itgb4 gene expression in pc-3 cells with short interfering rna induces changes in the expression of b-integrins associated with rgd-receptors. Bull. Exp. Biol. Med. 159, 541–545. 10.1007/s10517-015-3011-9 26395630

[B15] Krainova N. A. Khaustova N. A. Makeeva D. S. Fedotov N. N. Gudim E. A. Ryabenko E. A. (2013). Evaluation of potential reference genes for qRT-PCR data normalization in HeLa cells. Appl. Biochem. Microbiol. 49, 743–749. 10.1134/S0003683813090032

[B16] Kudriaeva A. Galatenko V. Maltseva D. Khaustova N. Kuzina E. Tonevitsky A.. (2017). The transcriptome of type I murine astrocytes under interferon-gamma exposure and remyelination stimulus. Molecules 22:808. 10.3390/molecules22050808 28505143 PMC6153759

[B17] Makarova J. Shkurnikov M. Turchinovich A. Tonevitsky A. Grigoriev A. (2015). Circulating micrornas. Biochemistry 80, 1117–1126. 10.1134/S0006297915090035 26555465

[B18] Makarova J. Shkurnikov M. Wicklein D. Lange T. Samatov T. Turchinovich A.. (2016). Intracellular and extracellular microrna: an update on localization and biological role. Prog. Histochem. Cytochem. 51, 33–49. 10.1016/j.proghi.2016.06.001 27396686

[B19] Malik A. Szydlowska K. Nizinska K. Asaro A. Vliet E. Popp O.. (2019). SorCS2 controls functional expression of amino acid transporter EAAT3 and protects neurons from oxidative stress and epilepsy-induced pathology. Cell Rep. 26, 2792–2804. 10.1016/j.celrep.2019.02.027 30840898 PMC6410498

[B20] Maltseva D. V. Rodin S. A. (2018). Laminins in metastatic cancer. Mol. Biol. 52, 411–434. 10.1134/S0026893318030093 29989574

[B21] Marzec K. Lin M. Martin J. Baxter R. (2015). Involvement of p53 in insulin-like growth factor binding protein-3 regulation in the breast cancer cell response to dna damage. Oncotarget 6, 26583–26598. 10.18632/oncotarget.5612 26378048 PMC4694938

[B22] Nikulin S. Raigorodskaya M. Poloznikov A. Zakharova G. Schumacher U. Wicklein D.. (2018). Role of IGFBP6 protein in the regulation of epithelial-mesenchymal transition genes. Bull. Exp. Biol. Med. 164, 650–654. 10.1007/s10517-018-4051-8 29577195

[B23] Poloznikov A. Nikulin S. Raigorodskaya M. Fomicheva K. Zakharova G. Makarova Y.. (2019). Changes in the metastatic properties of mda-mb-231 cells after igfbp6 gene knockdown is associated with increased expression of mirna genes controlling insr, igf1r, and ccnd1 genes. Bull. Exp. Biol. Med. 166, 641–645. 10.1007/s10517-019-04409-z 30903488

[B24] Ross J. Huh D. Noble L. Tavazoie S. (2015). Identification of molecular determinants of primary and metastatic tumour re-initiation in breast cancer. Nat. Cell Biol. 17, 651–664. 10.1038/ncb3148 25866923 PMC4609531

[B25] Sakharov D. A. Maltseva D. V. Riabenko E. A. Shkurnikov M. U. Northoff H. Tonevitsky A. G.. (2012). Passing the anaerobic threshold is associated with substantial changes in the gene expression profile in white blood cells. Eur. J. Appl. Physiol. 112, 963–972. 10.1007/s00421-011-2048-3 21717121

[B26] Seok H. Lee H. Jang E. Chi S. (2018). Evaluation and control of mirna-like off-target repression for RNA interference. Cell. Mol. Life Sci. 75, 797–814. 10.1007/s00018-017-2656-0 28905147 PMC11105550

[B27] Shkurnikov M. Nechaev I. Khaustova N. Krainova N. Savelov N. Grinevich V.. (2013). Expression profile of inflammatory breast cancer. Bull. Exp. Biol. Med. 155, 667–672. 10.1007/s10517-013-2221-2 24288735

[B28] Shkurnikov M. Y. Maltseva D. V. Knyazev E. N. Alekseev B. Y. (2018). Expression of stroma components in the lymph nodes affected by prostate cancer metastases. Mol. Biol. 52, 810–816. 10.1134/S0026893318050126 30363056

[B29] Teumer A. Qi Q. Nethander M. Aschard H. Bandinelli S. Beekman M.. (2016). Genomewide meta-analysis identifies loci associated with igf-i and igfbp-3 levels with impact on age-related traits. Aging Cell 15, 811–824. 10.1111/acel.12490 27329260 PMC5013013

[B30] Whisnant A. W. Bogerd H. P. Flores O. Ho P. Powers J. G. Sharova N.. (2013). In-depth analysis of the interaction of HIV-1 with cellular microRNA biogenesis and effector mechanisms. MBio 4:e000193. 10.1128/mBio.00193-13 23592263 PMC3634607

[B31] Yang Z.-X. Zhang B. Wei J. Jiang G.-Q. Wu Y.-L. Leng B.-J.. (2018). MIR-539 inhibits proliferation and migration of triple-negative breast cancer cells by down-regulating LAMA4 expression. Cancer Cell Int. 18:16. 10.1186/s12935-018-0512-4 29434522 PMC5791727

