# Supplementary material for: LAMA4-Regulating miR-4274 and Its Host Gene SORCS2 Play a Role in IGFBP6-Dependent Effects on Phenotype of Basal-Like Breast Cancer
Source: Front Mol Biosci. 2019 Nov 8;6:122. doi: 10.3389/fmolb.2019.00122 (PMC6857517; doi:10.3389/fmolb.2019.00122)
Supplement: Supplementary file 1 [file Table_1.DOCX]

**Supplementary Table 1.** Enrichment analysis of a set of genes differentially expresssed after a transcriptional knockdown of *IGFBP6* gene in MDA-MB-231 cells (N=380) (FDR p 0:01, fold change 2).

| **Ontology Component** | **Gene Overlap** | **Percent Overlap** | **P-value** | **Jaccard similarity** | **List of overlapping genes** | |  |
| --- | --- | --- | --- | --- | --- | --- | --- |
| **Enrichment for Biological Processes** | | | | | | |  |
| Biological Adhesion | 50 | 5 | 1.23E-16 | 0.038 | PCDH17, PCDH11X, SRPX, MYL9, GBP1, GBP2, PGM5, GBP3, PIK3CG, CD22, CD33, TENM3, ROBO1, ADGRE5, NRP2, CDH11, IL1B, INHBB, ITGB3, BAIAP2L1, STXBP6, CCL2, PCDHB6, LAMA1, FAT4, PTPRS, CASK, HES1, CDON, ADAMTS12, PCDH11Y, DSP, PCDHB2, PDLIM1, LAMA3, LAMA4, PLCB1, ADGRL3, MPZL3, PCDHB5, CNTN5, FLRT2, EFNB2, TGFB2, THBS1, PCDHB16, EPHA3, NUAK1, SLAMF7, NEO1 | |  |
| Regulation Of Signal Transduction | 103 | 2 | 7.81E-16 | 0.026 | ARHGDIB, PLK2, ARRB1, ATP2B4, PDE10A, BCL2A1, MIR181B1, ABCA1, TSPYL5, CNKSR2, CARD8, CLU, CLIC2, BMP4, BMP1, IL24, ESM1, IRAK3, DMD, ARL2BP, TRIM6, PIK3AP1, CSF1, PLCB1, CYP2J2, FGF13, PTPN22, TIAM2, LPAR1, EPHA7, EPHA3, LYPD6B, EYA4, F2RL1, F3, F2RL2, EFEMP1, FBP1, PDCD4, GRB14, GATA6, GBP1, PRICKLE1, GJA1, IGFBP4, IL1B, INHBB, TRIB2, ITGB3, FOXA1, HES1, CDON, MEF2C, MAP3K1, MMP3, NRK, KISS1, G0S2, LTBP1, NEO1, PDE3B, PIK3CG, PLAU, ROBO1, LMO3, BAIAP2L1, CCL2, PTPRS, RGS10, SOX4, SLC44A2, ZMIZ1, TCF4, PMEPA1, SNAI2, SAMSN1, VEGFA, NXN, HHIP, TGFA, TGFB2, THBS1, TGFBR3, TGM2, TIMP3, TLR3, SHISA2, SRPX, CXCR4, GPRC5A, GPR65, CDC42BPA, LGR5, SAMD5, TNFSF10, CRADD, ADAMTS12, GMFG, IL1RL1, NOG, AFAP1L2, OPTN, NUAK1 | |  |
| Cell Adhesion | 46 | 4 | 2.87E-14 | 0.035 | PCDH17, PCDH11X, SRPX, MYL9, PGM5, PIK3CG, CD22, CD33, TENM3, ROBO1, ADGRE5, NRP2, CDH11, IL1B, ITGB3, BAIAP2L1, STXBP6, CCL2, PCDHB6, LAMA1, FAT4, PTPRS, CASK, HES1, CDON, ADAMTS12, PCDH11Y, DSP, PCDHB2, PDLIM1, LAMA3, LAMA4, PLCB1, ADGRL3, MPZL3, PCDHB5, CNTN5, FLRT2, EFNB2, TGFB2, THBS1, PCDHB16, EPHA3, NUAK1, SLAMF7, NEO1 | |  |
| Regulation Of Cell Communication | 108 | 2 | 8.95E-14 | 0.024 | ARHGDIB, PLK2, ARRB1, ATP2B4, PDE10A, BCL2A1, MIR181B1, ABCA1, TSPYL5, CNKSR2, CARD8, CLU, CLIC2, BMP4, BMP1, IL24, ESM1, IRAK3, DMD, ARL2BP, TRIM6, PIK3AP1, CPT1A, CSF1, PLCB1, CYP2J2, FGF13, PTPN22, TIAM2, LPAR1, EPHA7, EPHA3, LYPD6B, EYA4, F2RL1, F3, F2RL2, EFEMP1, FBP1, PDCD4, PCDH17, GRB14, GATA6, GBP1, PRICKLE1, GJA1, IGFBP4, IL1B, INHBB, TRIB2, ITGB3, FOXA1, HES1, CDON, MEF2C, MAP3K1, MMP3, NRK, KISS1, G0S2, LTBP1, NEO1, PDE3B, PIK3CG, PLAU, ROBO1, MCTP2, LMO3, BAIAP2L1, SCN9A, CCL2, PTPRS, RGS10, SOX4, SLC44A2, ZMIZ1, TCF4, PMEPA1, SNAI2, SAMSN1, VEGFA, NXN, HHIP, TGFA, TGFB2, THBS1, TGFBR3, TGM2, TIMP3, TLR3, SHISA2, SRPX, CXCR4, GPRC5A, GPR65, CDC42BPA, LGR5, CASK, SAMD5, TNFSF10, CRADD, ADAMTS12, GMFG, IL1RL1, NOG, AFAP1L2, OPTN, NUAK1 | |  |
| Regulation Of Multicellular Organismal Process | 100 | 2 | 1.27E-13 | 0.025 | ARHGDIB, PLK2, ARRB1, ATP2B4, MIR181B1, ABCA1, MYL9, NAV3, ADGRE5, SLITRK1, FBXO32, CARD8, CHRM3, CLU, CLIC2, BMP4, BMP1, IRAK3, CCND2, DMD, DPYSL2, DSP, TRIM6, CSF1, PLCB1, ADGRL3, CYP2J2, FGF13, PTPN22, LPAR1, S1PR3, FLRT2, EFNB2, EPHA7, EPHA3, F2RL1, F3, EFEMP1, TNFRSF21, PDCD4, GRB14, HAS2, NCKAP1L, HLA-A, GATA6, GBP1, PRICKLE1, GJA1, IL1B, INHBB, TRIB2, ITGB3, FOXA1, HES1, CDON, MEF2C, NRK, KISS1, LAMA3, LAMA4, ASAP1, BCL11A, SERPINB2, NEO1, PDE3B, PIK3CG, PLAU, PLXNA2, PLXNA1, TENM3, ROBO1, SCN5A, LAMA1, PTPRS, ZMIZ1, HEG1, ZNF608, TBXAS1, TCF4, SDC2, SNAI2, VEGFA, WT1, HHIP, TGFB2, THBS1, TGFBR3, TLR3, HIST2H4B, CXCR4, NRP2, FAT4, CASK, SERPINB7, ADAMTS12, IL1RL1, NOG, LIPG, AFAP1L2, OPTN | |  |
| Regulation Of Signaling | 108 | 2 | 1.36E-13 | 0.024 | ARHGDIB, PLK2, ARRB1, ATP2B4, PDE10A, BCL2A1, MIR181B1, ABCA1, TSPYL5, CNKSR2, CARD8, CLU, CLIC2, BMP4, BMP1, IL24, ESM1, IRAK3, DMD, ARL2BP, TRIM6, PIK3AP1, CPT1A, CSF1, PLCB1, CYP2J2, FGF13, PTPN22, TIAM2, LPAR1, EPHA7, EPHA3, LYPD6B, EYA4, F2RL1, F3, F2RL2, EFEMP1, FBP1, PDCD4, PCDH17, GRB14, GATA6, GBP1, PRICKLE1, GJA1, IGFBP4, IL1B, INHBB, TRIB2, ITGB3, FOXA1, HES1, CDON, MEF2C, MAP3K1, MMP3, NRK, KISS1, G0S2, LTBP1, NEO1, PDE3B, PIK3CG, PLAU, ROBO1, MCTP2, LMO3, BAIAP2L1, SCN9A, CCL2, PTPRS, RGS10, SOX4, SLC44A2, ZMIZ1, TCF4, PMEPA1, SNAI2, SAMSN1, VEGFA, NXN, HHIP, TGFA, TGFB2, THBS1, TGFBR3, TGM2, TIMP3, TLR3, SHISA2, SRPX, CXCR4, GPRC5A, GPR65, CDC42BPA, LGR5, CASK, SAMD5, TNFSF10, CRADD, ADAMTS12, GMFG, IL1RL1, NOG, AFAP1L2, OPTN, NUAK1 | |  |
| Regulation Of Cell Migration | 46 | 4 | 3.43E-13 | 0.033 | ARHGDIB, PLK2, HAS2, NCKAP1L, MIR181B1, CXCR4, NAV3, PLAU, PLXNA2, PLXNA1, ROBO1, NRP2, IL1B, ITGB3, SCP2, CCL2, LAMA1, BMP4, IL24, DOCK10, MEF2C, CEMIP, MAP3K1, MMP3, MMP1, MTUS1, KISS1, NOG, LAMA3, LAMA4, CSF1, PLCB1, SNAI2, PARD6B, PTPN22, VEGFA, LPAR1, FLRT2, TGFB2, THBS1, TGFBR3, EPHA3, F2RL1, F3, NEO1, EFEMP1 | |  |
| Regulation Of Response To Stimulus | 120 | 2 | 3.57E-13 | 0.023 | ARHGDIB, PLK2, ARRB1, ATP2B4, PDE10A, BCL2A1, MIR181B1, ABCA1, TSPYL5, CD33, CNKSR2, FBXO32, CARD8, CLU, CLIC2, BMP4, BMP1, IL24, C4BPB, ESM1, IRAK3, DMD, ARL2BP, TRIM6, PIK3AP1, CSF1, PLCB1, CYP2J2, FGF13, PTPN22, TIAM2, LPAR1, EFNB2, EPHA7, EPHA3, LYPD6B, EYA4, F2RL1, F3, F2RL2, EFEMP1, FBP1, TNFRSF21, PDCD4, GRB14, NCKAP1L, HLA-A, HLA-B, GATA6, GBP1, PRICKLE1, GJA1, IGFBP4, IL1B, IL13RA2, INHBB, TRIB2, ITGB3, HLA-DRA, FOXA1, HES1, CDON, MEF2C, MAP3K1, MMP3, NRK, KISS1, G0S2, LTBP1, PLPP4, SERPINB2, NEO1, PDE3B, PIK3CG, PLAU, ROBO1, LMO3, BAIAP2L1, CCL2, HMSD, PTPRS, RGS10, SOX4, SLC44A2, ZMIZ1, MTUS1, TCF4, PMEPA1, SNAI2, SAMSN1, VEGFA, NXN, HHIP, TGFA, TGFB2, THBS1, TGFBR3, TGM2, TIMP3, SLAMF7, TLR3, SHISA2, SRPX, CXCR4, GPRC5A, GPR65, CDC42BPA, LGR5, CASK, SAMD5, TNFSF10, CRADD, ADAMTS12, GMFG, IL1RL1, NOG, AFAP1L2, OPTN, NUAK1, CLEC2B | |  |
| Regulation Of Localization | 88 | 2 | 1.88E-12 | 0.025 | ARHGDIB, PLK2, ARRB1, ATP2B4, MIR181B1, ABCA1, NAV3, CARD8, CLU, CLIC2, BMP4, IL24, C4BPB, SYTL3, DMD, ARL2BP, DPYSL2, DSP, TRIM6, CPT1A, CSF1, PLCB1, CYP2J2, PTPN22, LPAR1, FLRT2, STOM, EPHA3, F2RL1, F3, EFEMP1, TNFRSF21, PCDH17, HAS2, NCKAP1L, GBP1, GJA1, IL1B, IL13RA2, INHBB, ITGB3, STXBP6, HES1, MEF2C, MAP3K1, MMP3, MMP1, KCNQ3, KISS1, LAMA3, LAMA4, LCP1, KCNIP3, CLIC6, NEO1, PKIA, PDE3B, PIK3CG, PLAU, PLCB4, PLXNA2, PLTP, PLXNA1, ROBO1, MCTP2, SCN5A, SCP2, SCN9A, CCL2, LAMA1, RAB27B, DOCK10, SOX4, CEMIP, MTUS1, SNAI2, VEGFA, TGFB2, THBS1, TGFBR3, CXCR4, NRP2, CLIC3, CASK, IL1RL1, NOG, LIPG, PARD6B | |  |
| Regulation Of Cell Motility | 46 | 4 | 3.39E-12 | 0.032 | ARHGDIB, PLK2, HAS2, NCKAP1L, MIR181B1, CXCR4, NAV3, PLAU, PLXNA2, PLXNA1, ROBO1, NRP2, IL1B, ITGB3, SCP2, CCL2, LAMA1, BMP4, IL24, DOCK10, MEF2C, CEMIP, MAP3K1, MMP3, MMP1, MTUS1, KISS1, NOG, LAMA3, LAMA4, CSF1, PLCB1, SNAI2, PARD6B, PTPN22, VEGFA, LPAR1, FLRT2, TGFB2, THBS1, TGFBR3, EPHA3, F2RL1, F3, NEO1, EFEMP1 | |  |
| Regulation Of Cell Adhesion | 38 | 4 | 3.62E-12 | 0.034 | TNFRSF21, ARHGDIB, HAS2, NCKAP1L, HLA-A, PDE3B, GBP1, PIK3CG, PLAU, PLXNA2, PLXNA1, TENM3, IL1B, ITGB3, CCL2, LAMA1, BMP4, FOXA1, CASK, HES1, ZMIZ1, CYTIP, DMD, ZNF608, LAMA3, LAMA4, CSF1, SNAI2, PTPN22, VEGFA, EFNB2, TGFB2, THBS1, TGM2, EPHA7, EPHA3, NUAK1, ABI3BP | |  |
| Regulation Of Cellular Component Movement | 48 | 3 | 3.98E-12 | 0.031 | ARHGDIB, PLK2, HAS2, NCKAP1L, MIR181B1, CXCR4, NAV3, PLAU, PLXNA2, PLXNA1, ROBO1, NRP2, IL1B, ITGB3, SCN5A, SCP2, CCL2, LAMA1, BMP4, IL24, DOCK10, MEF2C, CEMIP, MAP3K1, MMP3, MMP1, MTUS1, DSP, KISS1, NOG, LAMA3, LAMA4, CSF1, PLCB1, SNAI2, PARD6B, PTPN22, VEGFA, LPAR1, FLRT2, TGFB2, THBS1, TGFBR3, EPHA3, F2RL1, F3, NEO1, EFEMP1 | |  |
| Negative Regulation Of Multicellular Organismal Process | 55 | 3 | 5.36E-12 | 0.029 | ARHGDIB, PLK2, ARRB1, ATP2B4, PDE3B, PIK3CG, NAV3, PLAU, ROBO1, FBXO32, CARD8, BMP4, PTPRS, IRAK3, ZNF608, TCF4, SNAI2, FGF13, PTPN22, VEGFA, WT1, HHIP, LPAR1, EFNB2, TGFB2, THBS1, TGFBR3, EPHA7, EPHA3, TLR3, F2RL1, EFEMP1, TNFRSF21, PDCD4, GRB14, HIST2H4B, NCKAP1L, HLA-A, GATA6, GBP1, PRICKLE1, GJA1, IL1B, INHBB, TRIB2, FOXA1, HES1, ADAMTS12, MEF2C, IL1RL1, NOG, ASAP1, BCL11A, SERPINB2, NEO1 | |  |
| Regulation Of Intracellular Signal Transduction | 67 | 3 | 1.07E-11 | 0.027 | ARHGDIB, PLK2, ARRB1, ATP2B4, PDE10A, BCL2A1, MIR181B1, PDE3B, ABCA1, TSPYL5, PIK3CG, ROBO1, CARD8, LMO3, CLU, CLIC2, CCL2, BMP4, IL24, IRAK3, SLC44A2, DMD, ARL2BP, TRIM6, PIK3AP1, CSF1, PLCB1, SNAI2, PTPN22, TIAM2, VEGFA, LPAR1, TGFA, TGFB2, THBS1, TGFBR3, TGM2, TIMP3, EPHA7, EPHA3, TLR3, F2RL1, F3, F2RL2, FBP1, PDCD4, GBP1, GJA1, CXCR4, IGFBP4, IL1B, INHBB, TRIB2, ITGB3, GPR65, CDC42BPA, SAMD5, HES1, TNFSF10, CDON, MAP3K1, MMP3, NRK, IL1RL1, KISS1, OPTN, NUAK1 | |  |
| Regulation Of Locomotion | 47 | 3 | 1.16E-11 | 0.030 | ARHGDIB, PLK2, HAS2, NCKAP1L, MIR181B1, CXCR4, NAV3, PLAU, PLXNA2, PLXNA1, ROBO1, NRP2, IL1B, ITGB3, SCP2, CCL2, LAMA1, BMP4, IL24, DOCK10, MEF2C, CEMIP, MAP3K1, MMP3, MMP1, MTUS1, KISS1, NOG, LAMA3, LAMA4, CSF1, PLCB1, SNAI2, PARD6B, PTPN22, VEGFA, LPAR1, FLRT2, EFNB2, TGFB2, THBS1, TGFBR3, EPHA3, F2RL1, F3, NEO1, EFEMP1 | |  |
| Wound Healing | 17 | 11 | 1.82E-11 | 0.033 | MAP3K1, MMP3, DSP, SDC2, NOG, PLAU, LTBP1, OPTN, IL1B, VEGFA, ITGB3, SERPINB2, BMP4, IL24, TGFA, TGFB2, F13A1 | |  |
| Regulation Of Protein Phosphorylation | 57 | 3 | 1.87E-11 | 0.028 | ARRB1, PKIA, ATP2B4, MIR181B1, PIK3CG, ROBO1, LMO3, CLU, CCL2, BMP4, IL24, IRAK3, CCND2, CEMIP, DMD, ARL2BP, TRIM6, PIK3AP1, CSF1, PLCB1, PMEPA1, FGF13, PTPN22, SAMSN1, VEGFA, LPAR1, TGFA, TGFB2, THBS1, TGFBR3, TIMP3, EPHA7, EPHA3, TLR3, F2RL1, ACSL1, PDCD4, NCKAP1L, GBP1, CXCR4, IGFBP4, IL1B, INHBB, TRIB2, ITGB3, GPRC5A, SAMD5, HES1, CDON, GMFG, MAP3K1, NRK, KISS1, NOG, RAD50, AFAP1L2, CTDSPL | |  |
| Negative Regulation Of Response To Stimulus | 60 | 3 | 4.68E-11 | 0.027 | PLK2, ARRB1, ATP2B4, PDE10A, BCL2A1, PDE3B, PLAU, ROBO1, CARD8, LMO3, CLU, CLIC2, CCL2, BMP4, PTPRS, C4BPB, IRAK3, RGS10, DMD, PIK3AP1, PMEPA1, SNAI2, CYP2J2, PTPN22, SAMSN1, VEGFA, NXN, HHIP, LPAR1, TGFB2, THBS1, TGFBR3, TIMP3, EPHA7, EPHA3, EYA4, TLR3, F2RL1, FBP1, PDCD4, GRB14, SHISA2, HLA-A, GBP1, PRICKLE1, GJA1, IGFBP4, IL1B, IL13RA2, GPRC5A, CASK, TNFSF10, ADAMTS12, MMP3, IL1RL1, NOG, LTBP1, OPTN, SERPINB2, NEO1 | |  |
| Regulation Of Protein Modification Process | 65 | 3 | 5.82E-11 | 0.026 | ARRB1, PKIA, ATP2B4, ARRDC4, MIR181B1, TSPYL5, PIK3CG, ROBO1, LMO3, CLU, CCL2, BMP4, IL24, IRAK3, CCND2, SOX4, CEMIP, DMD, ARL2BP, TRIM6, PIK3AP1, CSF1, PLCB1, PMEPA1, SNAI2, FGF13, PTPN22, SAMSN1, VEGFA, NXN, LPAR1, TGFA, TGFB2, THBS1, TGFBR3, TIMP3, EPHA7, EPHA3, TLR3, F2RL1, ACSL1, PDCD4, NCKAP1L, GBP1, PRICKLE1, CXCR4, IGFBP4, IL1B, INHBB, TRIB2, ITGB3, GPRC5A, SAMD5, HES1, SH3RF2, CDON, GMFG, MAP3K1, NRK, KISS1, NOG, RAD50, AFAP1L2, CTDSPL, NUAK1 | |  |
| Positive Regulation Of Signal Transduction | 59 | 3 | 6.63E-11 | 0.027 | PLK2, ARRB1, MIR181B1, TSPYL5, PIK3CG, ROBO1, LMO3, CLU, CCL2, BMP4, IL24, ESM1, SOX4, SLC44A2, ZMIZ1, ARL2BP, TRIM6, PIK3AP1, CSF1, PLCB1, PTPN22, VEGFA, LPAR1, TGFA, TGFB2, THBS1, TGFBR3, TGM2, TIMP3, EPHA7, EPHA3, TLR3, F2RL1, F3, F2RL2, PDCD4, GRB14, SRPX, GATA6, GJA1, CXCR4, IGFBP4, IL1B, INHBB, ITGB3, GPR65, FOXA1, LGR5, SAMD5, HES1, TNFSF10, CRADD, CDON, MAP3K1, NRK, KISS1, G0S2, AFAP1L2, NEO1 | |  |
| Cell Surface Receptor Signaling Pathway | 76 | 2 | 8.37E-11 | 0.025 | BCL2A1, ABCA1, ADGRF1, PLXNA2, PLXNA1, CD22, ROBO1, ADGRE5, CCL2, LAMA1, BMP4, IL24, IRAK3, SOX4, SDC2, CSF1, PLCB1, ADGRL3, SNAI2, PTPN22, VEGFA, NXN, HHIP, S1PR3, FLRT2, EFNB2, TGFA, TGFB2, TGFBR3, EPHA7, EPHA3, TLR3, F3, F13A1, ACSL1, EFEMP1, TNFRSF21, PDCD4, GRB14, NCKAP1L, HLA-A, HLA-B, GBP1, PORCN, GBP2, PRICKLE1, CXCR4, NRP2, IL1B, IL13RA2, INHBB, ITGB3, CPEB4, CD82, SORBS2, HLA-DRA, FAT4, FOXA1, HES1, TNFSF10, CRADD, CDON, MEF2C, MAP3K1, MMP3, MMP1, IL1RL1, NOG, LCP1, G0S2, LTBP1, PLPP4, TSPAN2, SHC3, SERPINB2, FAM83B | |  |
| Regulation Of Phosphorylation | 58 | 3 | 8.38E-11 | 0.025 | | ARRB1, PKIA, ATP2B4, MIR181B1, PIK3CG, ROBO1, LMO3, CLU, CCL2, BMP4, IL24, IRAK3, CCND2, CEMIP, DMD, ARL2BP, TRIM6, PIK3AP1, CSF1, PLCB1, PMEPA1, FGF13, PTPN22, SAMSN1, VEGFA, LPAR1, TGFA, TGFB2, THBS1, TGFBR3, TIMP3, EPHA7, EPHA3, TLR3, F2RL1, ACSL1, FBP1, PDCD4, NCKAP1L, GBP1, CXCR4, IGFBP4, IL1B, INHBB, TRIB2, ITGB3, GPRC5A, SAMD5, HES1, CDON, GMFG, MAP3K1, NRK, KISS1, NOG, RAD50, AFAP1L2, CTDSPL |  |
| Positive Regulation Of Multicellular Organismal Process | 63 | 3 | 9.78E-11 | 0.027 | | PLK2, MIR181B1, PLAU, PLXNA2, PLXNA1, TENM3, ROBO1, ADGRE5, SLITRK1, CARD8, CHRM3, CLU, SCN5A, BMP4, BMP1, IRAK3, ZMIZ1, DMD, HEG1, TBXAS1, TCF4, TRIM6, CSF1, PLCB1, ADGRL3, SNAI2, CYP2J2, PTPN22, VEGFA, WT1, LPAR1, FLRT2, EFNB2, TGFB2, THBS1, TGFBR3, EPHA3, TLR3, F2RL1, F3, HAS2, NCKAP1L, HLA-A, GATA6, GJA1, CXCR4, NRP2, IL1B, INHBB, ITGB3, FOXA1, CASK, HES1, SERPINB7, CDON, MEF2C, IL1RL1, KISS1, NOG, LIPG, BCL11A, AFAP1L2, OPTN |  |
| Regulation Of Phosphate Metabolic Process | 62 | 3 | 1.44E-10 | 0.026 | | ARRB1, PKIA, ATP2B4, MIR181B1, PIK3CG, ROBO1, LMO3, CLU, CCL2, BMP4, IL24, IRAK3, CCND2, CEMIP, DMD, ARL2BP, TRIM6, PIK3AP1, CSF1, PLCB1, PMEPA1, FGF13, PTPN22, SAMSN1, VEGFA, LPAR1, TGFA, TGFB2, THBS1, TGFBR3, TIMP3, EPHA7, EPHA3, TLR3, F2RL1, ACSL1, FBP1, PDCD4, PCDH11X, NCKAP1L, GBP1, CXCR4, IGFBP4, IL1B, INHBB, TRIB2, ITGB3, GPRC5A, SAMD5, HES1, SH3RF2, CDON, GMFG, MEF2C, MAP3K1, NRK, KISS1, NOG, RAD50, AFAP1L2, CTDSPL, NUAK1 |  |
| Regulation Of Phosphorus Metabolic Process | 62 | 3 | 1.72E-10 | 0.026 | | ARRB1, PKIA, ATP2B4, MIR181B1, PIK3CG, ROBO1, LMO3, CLU, CCL2, BMP4, IL24, IRAK3, CCND2, CEMIP, DMD, ARL2BP, TRIM6, PIK3AP1, CSF1, PLCB1, PMEPA1, FGF13, PTPN22, SAMSN1, VEGFA, LPAR1, TGFA, TGFB2, THBS1, TGFBR3, TIMP3, EPHA7, EPHA3, TLR3, F2RL1, ACSL1, FBP1, PDCD4, PCDH11X, NCKAP1L, GBP1, CXCR4, IGFBP4, IL1B, INHBB, TRIB2, ITGB3, GPRC5A, SAMD5, HES1, SH3RF2, CDON, GMFG, MEF2C, MAP3K1, NRK, KISS1, NOG, RAD50, AFAP1L2, CTDSPL, NUAK1 |  |
| Anatomical Structure Development | 107 | 2 | 1.75E-10 | 0.026 | | ARHGDIB, ATP2B4, BCL2A1, AK4, NAV3, CDH11, SLITRK1, CHRM3, CLU, BMP4, BMP1, CCND2, TDRD7, DMD, DPYSL2, DSP, CSF1, PLCB1, ADGRL3, FGF13, FLG, LPAR1, FLRT2, EFNB2, EPHA7, EPHA3, EYA4, F2RL1, EFEMP1, HAS2, NCKAP1L, HLA-A, GATA6, PRICKLE1, GJA1, IGFBP4, INHBB, ITGB3, HMGCS1, FOXA1, SHISA3, HES1, CDON, MEF2C, MAP3K1, SAMD9L, KCNQ3, SNX10, KISS1, LAMA3, LAMA4, LCP1, LTBP1, BCL11A, SHC3, ZNF438, NEO1, PDE3B, PLAU, PLXNA2, PLXNA1, TENM3, ROBO1, MCTP2, SCN5A, PCDHB6, LAMA1, PTPRS, SOX4, ZMIZ1, HEG1, TCF4, PCDHB2, SDC2, PRDM8, SNAI2, TUFT1, UGCG, VEGFA, WT1, NXN, TGFA, TGFB2, THBS1, TGFBR3, TIMP3, TLL1, TLR3, SLC7A5, ILDR2, SHISA2, TNS3, EVI5, CXCR4, NRP2, GPR65, FAT4, LGR5, CASK, CLMP, TNFSF10, CRISPLD2, NOG, TSPAN2, RAD50, OPTN, RAPGEF5 |  |
| Negative Regulation Of Cell Communication | 52 | 3 | 3.14E-10 | 0.022 | | PLK2, ARRB1, ATP2B4, PDE10A, BCL2A1, PDE3B, ROBO1, CARD8, LMO3, CLU, CLIC2, BMP4, PTPRS, IRAK3, RGS10, DMD, PIK3AP1, PMEPA1, SNAI2, CYP2J2, PTPN22, VEGFA, NXN, HHIP, LPAR1, TGFB2, THBS1, TGFBR3, TIMP3, EPHA7, EPHA3, EYA4, TLR3, F2RL1, FBP1, PDCD4, PCDH17, GRB14, SHISA2, GBP1, PRICKLE1, IGFBP4, IL1B, INHBB, GPRC5A, TNFSF10, ADAMTS12, MMP3, IL1RL1, NOG, LTBP1, OPTN |  |
| Negative Regulation Of Signaling | 52 | 3 | 3.33E-10 | 0.028 | | PLK2, ARRB1, ATP2B4, PDE10A, BCL2A1, PDE3B, ROBO1, CARD8, LMO3, CLU, CLIC2, BMP4, PTPRS, IRAK3, RGS10, DMD, PIK3AP1, PMEPA1, SNAI2, CYP2J2, PTPN22, VEGFA, NXN, HHIP, LPAR1, TGFB2, THBS1, TGFBR3, TIMP3, EPHA7, EPHA3, EYA4, TLR3, F2RL1, FBP1, PDCD4, PCDH17, GRB14, SHISA2, GBP1, PRICKLE1, IGFBP4, IL1B, INHBB, GPRC5A, TNFSF10, ADAMTS12, MMP3, IL1RL1, NOG, LTBP1, OPTN |  |
| Negative Regulation Of Signal Transduction | 49 | 3 | 3.64E-10 | 0.028 | | ARRB1, ATP2B4, PDE10A, BCL2A1, PDE3B, ROBO1, CARD8, LMO3, CLU, CLIC2, BMP4, PTPRS, IRAK3, RGS10, DMD, PIK3AP1, PMEPA1, SNAI2, CYP2J2, PTPN22, VEGFA, NXN, HHIP, LPAR1, TGFB2, THBS1, TGFBR3, TIMP3, EPHA7, EPHA3, EYA4, TLR3, F2RL1, FBP1, PDCD4, GRB14, SHISA2, GBP1, PRICKLE1, IGFBP4, IL1B, GPRC5A, TNFSF10, ADAMTS12, MMP3, IL1RL1, NOG, LTBP1, OPTN |  |
| Regulation Of Multicellular Organismal Development | 70 | 2 | 3.81E-10 | 0.028 | | ARHGDIB, PLK2, MIR181B1, PDE3B, MYL9, PLAU, PLXNA2, PLXNA1, TENM3, ROBO1, ADGRE5, SLITRK1, LAMA1, BMP4, BMP1, PTPRS, IRAK3, CCND2, ZMIZ1, DMD, DPYSL2, ZNF608, TCF4, SDC2, CSF1, PLCB1, ADGRL3, SNAI2, FGF13, VEGFA, WT1, HHIP, LPAR1, S1PR3, FLRT2, EFNB2, TGFB2, THBS1, TGFBR3, EPHA7, EPHA3, TLR3, F3, EFEMP1, TNFRSF21, PDCD4, HIST2H4B, NCKAP1L, HLA-A, GATA6, PRICKLE1, GJA1, CXCR4, IL1B, ITGB3, FAT4, FOXA1, CASK, HES1, SERPINB7, CDON, ADAMTS12, MEF2C, NRK, NOG, LAMA3, LAMA4, ASAP1, BCL11A, NEO1 |  |
| Tube Morphogenesis | 22 | 6 | 6.96E-10 | 0.025 | | ZMIZ1, MEF2C, HEG1, GATA6, NOG, GJA1, CSF1, CXCR4, CTSD, VEGFA, WT1, HHIP, LAMA1, FAT4, BMP4, EFNB2, FOXA1, TGFB2, THBS1, TGFBR3, EPHA7, HES1 |  |
| Positive Regulation Of Response To Stimulus | 75 | 2 | 1.10E-09 | 0.032 | | PLK2, ARRB1, BCL2A1, MIR181B1, TSPYL5, CLU, BMP4, IL24, C4BPB, ESM1, IRAK3, ARL2BP, TRIM6, PIK3AP1, CSF1, PLCB1, PTPN22, LPAR1, EPHA7, EPHA3, EYA4, F2RL1, F3, F2RL2, TNFRSF21, PDCD4, GRB14, NCKAP1L, HLA-A, HLA-B, GATA6, GJA1, IGFBP4, IL1B, INHBB, ITGB3, HLA-DRA, FOXA1, HES1, CDON, MEF2C, MAP3K1, MMP3, NRK, KISS1, G0S2, PLPP4, NEO1, PIK3CG, ROBO1, LMO3, CCL2, HMSD, SOX4, SLC44A2, ZMIZ1, SNAI2, VEGFA, TGFA, TGFB2, THBS1, TGFBR3, TGM2, TIMP3, TLR3, SRPX, CXCR4, GPR65, LGR5, SAMD5, TNFSF10, CRADD, IL1RL1, AFAP1L2, OPTN |  |
| Developmental Process | 133 | 2 | 1.22E-09 | 0.024 | | ARHGDIB, ATP2B4, BCL2A1, AK4, NAV3, CDH11, SLITRK1, CHRM3, CLU, BMP4, BMP1, DMKN, CCND2, CYP24A1, DMD, DPYSL2, DSP, CPT1A, CSF1, CTSD, FGF13, FLG, LPAR1, S1PR3, EFNB2, EPHA7, EPHA3, EYA4, F2RL1, F3, EFEMP1, HAS2, NCKAP1L, HLA-A, GATA6, PRICKLE1, GJA1, IGFBP4, IL1B, ZDHHC15, INHBB, ITGB3, HMGCS1, FOXA1, SHISA3, HES1, MEF2C, MAP3K1, NRK, SAMD9L, KCNQ3, KISS1, LAMA3, LAMA4, LCP1, LTBP1, ZNF438, NEO1, PDE3B, PIK3CG, PLAU, PLXNA2, PLXNA1, ROBO1, RRBP1, SCN5A, SCP2, CCL2, LAMA1, PTPRS, SOX4, TCF4, SDC2, SNAI2, TUFT1, UGCG, VEGFA, WT1, TGFA, TGFB2, THBS1, TGFBR3, TGM2, TIMP3, TLL1, TLR3, SLC7A5, ILDR2, SHISA2, EVI5, CXCR4, NRP2, SORBS2, GPR65, LGR5, CASK, TNFSF10, NOG, TSPAN2, RAD50, OPTN, MBNL2, RAPGEF5, ESM1, TDRD7, PLCB1, ADGRL3, PTPN22, FLRT2, PDCD4, CDON, SNX10, BCL11A, SHC3, KRTAP4-8, RBM47, TENM3, MCTP2, PCDHB6, DOCK10, ZMIZ1, HEG1, PCDHB2, PRDM8, NXN, HHIP, TNS3, POF1B, FAT4, CLMP, CRISPLD1, CRISPLD2, PARD6B |  |
| Positive Regulation Of Molecular Function | 59 | 2 | 1.38E-09 | 0.020 | | ARHGDIB, PLK2, ARRB1, ATP2B4, ARRDC4, PIK3CG, ADGRF1, ROBO1, CARD8, CLU, CLIC2, CCL2, BMP4, IL24, DOCK10, IRAK3, RGS10, CCND2, CEMIP, DMD, TRIM6, CSF1, PLCB1, TBC1D30, FGF13, TIAM2, VEGFA, LPAR1, TGFA, TGFB2, THBS1, TGFBR3, EPHA7, EPHA3, TLR3, F2RL1, F3, ACSL1, SERINC2, NCKAP1L, EVI5, CXCR4, IL1B, TRIB2, ITGB3, TOR1AIP2, GPR65, FOXA1, SAMD5, HES1, TNFSF10, CRADD, GMFG, MEF2C, MAP3K1, NRK, ASAP1, RAD50, AFAP1L2 |  |
| Regulation Of Cellular Protein Metabolic Process | 79 | 2 | 1.54E-09 | 0.026 | | PLK2, ARRB1, ATP2B4, ARRDC4, BCL2A1, MIR181B1, TSPYL5, CARD8, CLU, BMP4, IL24, C4BPB, IRAK3, CCND2, DMD, ARL2BP, TRIM6, PIK3AP1, CSF1, PLCB1, CST4, FGF13, PTPN22, LPAR1, EPHA7, EPHA3, F2RL1, F3, ACSL1, PDCD4, NCKAP1L, GBP1, PRICKLE1, IGFBP4, IL1B, INHBB, TRIB2, ITGB3, HES1, SH3RF2, CDON, MAP3K1, NRK, KISS1, SERPINB2, PKIA, PIK3CG, ROBO1, LMO3, CCL2, HMSD, SOX4, CEMIP, PMEPA1, SNAI2, SAMSN1, VEGFA, WT1, NXN, TGFA, TGFB2, THBS1, TGFBR3, TIMP3, TLR3, CXCR4, CPEB4, GPRC5A, CST7, SAMD5, SERPINB7, TNFSF10, CRADD, GMFG, NOG, RAD50, AFAP1L2, CTDSPL, NUAK1 |  |
| Regulation Of Cell Differentiation | 63 | 2 | 1.70E-09 | 0.023 | | PLK2, MIR181B1, ABCA1, MYL9, PLXNA2, PLXNA1, TENM3, ROBO1, SLITRK1, LMO3, CLU, BMP4, PTPRS, CCND2, ZMIZ1, DMD, DPYSL2, ZNF608, TCF4, SDC2, TRIM6, CSF1, PLCB1, SNAI2, FGF13, VEGFA, LPAR1, S1PR3, FLRT2, EFNB2, TGFB2, THBS1, TGFBR3, EPHA7, EPHA3, TLR3, EFEMP1, TNFRSF21, PDCD4, GRB14, HAS2, HIST2H4B, NCKAP1L, HLA-A, GATA6, GBP1, PRICKLE1, GJA1, CXCR4, IL1B, TRIB2, ITGB3, FAT4, FOXA1, CASK, HES1, CDON, ADAMTS12, MEF2C, NOG, ASAP1, BCL11A, NEO1 |  |
| Response To Wounding | 22 | 6 | 2.00E-09 | 0.025 | | MAP3K1, MMP3, DSP, SDC2, NOG, LCP1, PLAU, LTBP1, OPTN, IL1B, INHBB, VEGFA, ITGB3, CLU, SERPINB2, BMP4, IL24, TGFA, TGFB2, F3, F2RL2, F13A1 |  |
| Positive Regulation Of Protein Modification Process | 47 | 3 | 2.55E-09 | 0.031 | | ARRB1, ATP2B4, ARRDC4, MIR181B1, TSPYL5, PIK3CG, ROBO1, CLU, CCL2, BMP4, IL24, CCND2, SOX4, CEMIP, ARL2BP, TRIM6, CSF1, PLCB1, SNAI2, FGF13, PTPN22, VEGFA, LPAR1, TGFA, TGFB2, THBS1, EPHA7, EPHA3, TLR3, F2RL1, ACSL1, NCKAP1L, PRICKLE1, CXCR4, IGFBP4, IL1B, INHBB, TRIB2, ITGB3, SAMD5, HES1, CDON, MAP3K1, NRK, KISS1, RAD50, AFAP1L2 |  |
| Regulation Of Mapk Cascade | 36 | 3 | 2.72E-09 | 0.027 | | PDCD4, ARRB1, MIR181B1, GBP1, CXCR4, PIK3CG, ROBO1, IGFBP4, IL1B, INHBB, LMO3, TRIB2, ITGB3, CCL2, BMP4, SAMD5, IRAK3, CDON, MAP3K1, DMD, NRK, KISS1, PIK3AP1, PLCB1, PTPN22, VEGFA, LPAR1, TGFA, TGFB2, THBS1, TGFBR3, TIMP3, EPHA7, EPHA3, TLR3, F2RL1 |  |
| Positive Regulation Of Cell Death | 35 | 4 | 2.84E-09 | 0.029 | | PDCD4, ARRB1, SRPX, GATA6, ROBO1, EFHC1, IL1B, FBXO32, CARD8, INHBB, CLU, SCP2, CCL2, BMP4, IL24, FOXA1, TNFSF10, CRADD, SOX4, MEF2C, MAP3K1, MMP3, G0S2, OPTN, TIAM2, WT1, LPAR1, EFNB2, TGFB2, THBS1, TGM2, TIMP3, EPHA7, TLR3, F3 |  |
| Regulation Of Developmental Process | 80 | 2 | 3.22E-09 | 0.029 | | ARHGDIB, PLK2, MIR181B1, ABCA1, MYL9, ADGRE5, SLITRK1, CLU, BMP4, BMP1, IRAK3, CCND2, DMD, DPYSL2, TRIM6, CSF1, PLCB1, ADGRL3, FGF13, LPAR1, S1PR3, FLRT2, EFNB2, EPHA7, EPHA3, F3, EFEMP1, TNFRSF21, PDCD4, GRB14, HAS2, NCKAP1L, HLA-A, GATA6, GBP1, PRICKLE1, GJA1, IL1B, TRIB2, ITGB3, FOXA1, HES1, CDON, MEF2C, NRK, LAMA3, LAMA4, ASAP1, BCL11A, NEO1, PDE3B, PLAU, PLXNA2, PLXNA1, TENM3, ROBO1, LMO3, CCL2, LAMA1, PTPRS, ZMIZ1, ZNF608, TCF4, SDC2, SNAI2, VEGFA, WT1, HHIP, TGFB2, THBS1, TGFBR3, TLR3, HIST2H4B, CXCR4, FAT4, CASK, SERPINB7, ADAMTS12, NOG, NUAK1 |  |
| Tissue Morphogenesis | 25 | 5 | 3.57E-09 | 0.023 | | MEF2C, MAP3K1, HEG1, DSP, NOG, GJA1, CSF1, CXCR4, SNAI2, CTSD, PLXNA1, NRP2, VEGFA, WT1, HHIP, LAMA1, FAT4, BMP4, EFNB2, FOXA1, TGFB2, TGFBR3, TGM2, EPHA7, HES1 |  |
| Positive Regulation Of Apoptotic Process | 33 | 4 | 3.81E-09 | 0.031 | | PDCD4, ARRB1, SRPX, GATA6, ROBO1, EFHC1, IL1B, FBXO32, CARD8, INHBB, CLU, SCP2, CCL2, BMP4, IL24, FOXA1, TNFSF10, CRADD, SOX4, MEF2C, MAP3K1, G0S2, OPTN, TIAM2, WT1, LPAR1, TGFB2, THBS1, TGM2, TIMP3, EPHA7, TLR3, F3 |  |
| Anatomical Structure Morphogenesis | 54 | 3 | 4.11E-09 | 0.029 | | PLXNA1, TENM3, ROBO1, CDH11, SLITRK1, CLU, SCN5A, CCL2, LAMA1, BMP4, DOCK10, SOX4, TDRD7, ZMIZ1, HEG1, DSP, SDC2, CSF1, PRDM8, SNAI2, CTSD, TUFT1, VEGFA, WT1, HHIP, S1PR3, FLRT2, EFNB2, TGFB2, THBS1, TGFBR3, TGM2, EPHA7, EYA4, EFEMP1, HAS2, NCKAP1L, GATA6, GJA1, CXCR4, POF1B, NRP2, ITGB3, FAT4, FOXA1, HES1, CDON, MEF2C, MAP3K1, CRISPLD1, CRISPLD2, NRK, NOG, PARD6B |  |
| Positive Regulation Of Catalytic Activity | 50 | 3 | 4.40E-09 | 0.026 | | ARHGDIB, ARRB1, ATP2B4, ARRDC4, PIK3CG, ROBO1, CARD8, CLU, CCL2, BMP4, IL24, DOCK10, RGS10, CCND2, CEMIP, CSF1, PLCB1, TBC1D30, FGF13, TIAM2, VEGFA, LPAR1, TGFA, TGFB2, THBS1, EPHA7, EPHA3, TLR3, F2RL1, F3, ACSL1, SERINC2, NCKAP1L, EVI5, CXCR4, IL1B, TRIB2, ITGB3, TOR1AIP2, GPR65, SAMD5, TNFSF10, CRADD, GMFG, MEF2C, MAP3K1, NRK, ASAP1, RAD50, AFAP1L2 |  |
| Positive Regulation Of Programmed Cell Death | 33 | 4 | 4.73E-09 | 0.026 | | PDCD4, ARRB1, SRPX, GATA6, ROBO1, EFHC1, IL1B, FBXO32, CARD8, INHBB, CLU, SCP2, CCL2, BMP4, IL24, FOXA1, TNFSF10, CRADD, SOX4, MEF2C, MAP3K1, G0S2, OPTN, TIAM2, WT1, LPAR1, TGFB2, THBS1, TGM2, TIMP3, EPHA7, TLR3, F3 |  |
| Regulation Of Protein Metabolic Process | 82 | 2 | 5.10E-09 | 0.029 | | PLK2, ARRB1, ATP2B4, ARRDC4, BCL2A1, MIR181B1, TSPYL5, CARD8, CLU, BMP4, IL24, C4BPB, IRAK3, CCND2, DMD, ARL2BP, TRIM6, PIK3AP1, CSF1, PLCB1, CST4, FGF13, PTPN22, LPAR1, EPHA7, EPHA3, F2RL1, F3, ACSL1, PDCD4, NCKAP1L, GBP1, PRICKLE1, GJA1, IGFBP4, IL1B, INHBB, TRIB2, ITGB3, HES1, SH3RF2, CDON, MAP3K1, NRK, SAMD9L, KISS1, SERPINB2, PKIA, PIK3CG, ROBO1, LMO3, CCL2, HMSD, SOX4, CEMIP, PMEPA1, SNAI2, SAMSN1, VEGFA, WT1, NXN, TGFA, TGFB2, THBS1, TGFBR3, TIMP3, TLR3, CXCR4, CPEB4, GPRC5A, CST7, SAMD5, SERPINB7, TNFSF10, CRADD, GMFG, NOG, LIPG, RAD50, AFAP1L2, CTDSPL, NUAK1 |  |
| Positive Regulation Of Protein Phosphorylation | 41 | 3 | 6.44E-09 | 0.022 | | ARRB1, ATP2B4, NCKAP1L, MIR181B1, CXCR4, PIK3CG, ROBO1, IGFBP4, IL1B, INHBB, ITGB3, CLU, CCL2, BMP4, IL24, SAMD5, HES1, CCND2, CDON, CEMIP, MAP3K1, ARL2BP, NRK, TRIM6, KISS1, CSF1, PLCB1, FGF13, RAD50, AFAP1L2, PTPN22, VEGFA, LPAR1, TGFA, TGFB2, THBS1, EPHA7, EPHA3, TLR3, F2RL1, ACSL1 |  |
| Positive Regulation Of Transferase Activity | 31 | 4 | 6.97E-09 | 0.027 | | ARRB1, ATP2B4, ARRDC4, NCKAP1L, CXCR4, PIK3CG, ROBO1, IL1B, TRIB2, ITGB3, CLU, BMP4, SAMD5, CCND2, CEMIP, MAP3K1, NRK, CSF1, FGF13, RAD50, AFAP1L2, VEGFA, LPAR1, TGFA, TGFB2, THBS1, EPHA7, EPHA3, TLR3, ACSL1, SERINC2 |  |
| Negative Regulation Of Developmental Process | 43 | 3 | 1.14E-08 | 0.029 | | ARHGDIB, PLK2, PDE3B, ABCA1, ROBO1, BMP4, PTPRS, ZNF608, TCF4, TRIM6, SNAI2, FGF13, VEGFA, WT1, HHIP, LPAR1, S1PR3, EFNB2, TGFB2, THBS1, TGFBR3, EPHA7, EPHA3, TLR3, EFEMP1, TNFRSF21, PDCD4, GRB14, HIST2H4B, HLA-A, GBP1, PRICKLE1, GJA1, IL1B, TRIB2, ITGB3, FOXA1, HES1, ADAMTS12, NOG, ASAP1, BCL11A, NEO1 |  |
| Positive Regulation Of Intracellular Signal Transduction | 42 | 3 | 1.16E-08 | 0.027 | | PDCD4, PLK2, ARRB1, MIR181B1, TSPYL5, GJA1, CXCR4, PIK3CG, ROBO1, IGFBP4, IL1B, ITGB3, CLU, CCL2, GPR65, BMP4, IL24, SAMD5, HES1, TNFSF10, CDON, SLC44A2, MAP3K1, ARL2BP, NRK, KISS1, PIK3AP1, CSF1, PLCB1, PTPN22, VEGFA, LPAR1, TGFA, TGFB2, THBS1, TGM2, EPHA7, EPHA3, TLR3, F2RL1, F3, F2RL2 |  |
| Regulation Of Cell Development | 40 | 3 | 1.46E-08 | 0.027 | | TNFRSF21, GRB14, PLK2, HAS2, HLA-A, GBP1, CXCR4, PLXNA2, PLXNA1, TENM3, ROBO1, SLITRK1, IL1B, ITGB3, BMP4, FOXA1, PTPRS, CASK, HES1, CCND2, CDON, MEF2C, DMD, DPYSL2, TCF4, SDC2, NOG, CSF1, ASAP1, FGF13, BCL11A, VEGFA, LPAR1, S1PR3, FLRT2, EFNB2, EPHA7, EPHA3, NEO1, EFEMP1 |  |
| Positive Regulation Of Cell Communication | 60 | 2 | 1.58E-08 | 0.027 | | PLK2, ARRB1, MIR181B1, TSPYL5, PIK3CG, ROBO1, LMO3, CLU, CCL2, BMP4, IL24, ESM1, SOX4, SLC44A2, ZMIZ1, ARL2BP, TRIM6, PIK3AP1, CSF1, PLCB1, PTPN22, VEGFA, LPAR1, TGFA, TGFB2, THBS1, TGFBR3, TGM2, TIMP3, EPHA7, EPHA3, TLR3, F2RL1, F3, F2RL2, PDCD4, GRB14, SRPX, GATA6, GJA1, CXCR4, IGFBP4, IL1B, INHBB, ITGB3, GPR65, FOXA1, LGR5, CASK, SAMD5, HES1, TNFSF10, CRADD, CDON, MAP3K1, NRK, KISS1, G0S2, AFAP1L2, NEO1 |  |
| Negative Regulation Of Cellular Process | 118 | 2 | 1.64E-08 | 0.024 | | ARHGDIB, PLK2, ARRB1, ATP2B4, PDE10A, BCL2A1, MIR181B1, ABCA1, NAV3, CD33, CARD8, CLU, CLIC2, BMP4, IL24, C4BPB, IRAK3, CCND2, DMD, TRIM6, PIK3AP1, PLCB1, CST4, TBC1D30, CYP2J2, FGF13, PTPN22, LPAR1, S1PR3, EFNB2, EPHA7, EPHA3, EYA4, FAM172BP, F2RL1, EFEMP1, FBP1, TNFRSF21, PDCD4, PCDH17, GRB14, PCDH11X, NCKAP1L, HLA-A, GATA6, GBP1, PRICKLE1, GJA1, IGFBP4, IL1B, IL13RA2, INHBB, TRIB2, ITGB3, STXBP6, FOXA1, HES1, SH3RF2, MEF2C, MAP3K1, MMP3, NRK, KISS1, KCNIP3, LTBP1, ASAP1, BCL11A, SERPINB2, ZNF438, NEO1, PKIA, PDE3B, PIK3CG, PLXNA2, PLXNA1, ROBO1, LMO3, SCP2, CCL2, HMSD, PTPRS, RGS10, SOX4, SSX1, ZNF608, TCF4, PMEPA1, PRDM8, SNAI2, SAMSN1, VEGFA, WT1, NXN, HHIP, TGFA, TGFB2, THBS1, TGFBR3, TIMP3, TLR3, SHISA2, HIST2H4B, SRPX, CPEB4, GPRC5A, CST7, CASK, SERPINB7, TNFSF10, CRADD, ADAMTS12, GMFG, PTGES, IL1RL1, NOG, RAD50, OPTN, CTDSPL |  |
| Positive Regulation Of Signaling | 60 | 2 | 1.66E-08 | 0.020 | | PLK2, ARRB1, MIR181B1, TSPYL5, PIK3CG, ROBO1, LMO3, CLU, CCL2, BMP4, IL24, ESM1, SOX4, SLC44A2, ZMIZ1, ARL2BP, TRIM6, PIK3AP1, CSF1, PLCB1, PTPN22, VEGFA, LPAR1, TGFA, TGFB2, THBS1, TGFBR3, TGM2, TIMP3, EPHA7, EPHA3, TLR3, F2RL1, F3, F2RL2, PDCD4, GRB14, SRPX, GATA6, GJA1, CXCR4, IGFBP4, IL1B, INHBB, ITGB3, GPR65, FOXA1, LGR5, CASK, SAMD5, HES1, TNFSF10, CRADD, CDON, MAP3K1, NRK, KISS1, G0S2, AFAP1L2, NEO1 |  |
| Positive Regulation Of Phosphorylation | 41 | 3 | 1.84E-08 | 0.024 | | ARRB1, ATP2B4, NCKAP1L, MIR181B1, CXCR4, PIK3CG, ROBO1, IGFBP4, IL1B, INHBB, ITGB3, CLU, CCL2, BMP4, IL24, SAMD5, HES1, CCND2, CDON, CEMIP, MAP3K1, ARL2BP, NRK, TRIM6, KISS1, CSF1, PLCB1, FGF13, RAD50, AFAP1L2, PTPN22, VEGFA, LPAR1, TGFA, TGFB2, THBS1, EPHA7, EPHA3, TLR3, F2RL1, ACSL1 |  |
| Regulation Of Catalytic Activity | 68 | 2 | 2.58E-08 | 0.026 | | ARHGDIB, ARRB1, PKIA, ATP2B4, ARRDC4, BCL2A1, PIK3CG, PLXNA2, PLXNA1, ROBO1, CARD8, CLU, CCL2, HMSD, BMP4, IL24, DOCK10, IRAK3, RGS10, CCND2, CEMIP, ARL2BP, CSF1, PLCB1, TBC1D30, FGF13, PTPN22, TIAM2, VEGFA, LPAR1, TGFA, TGFB2, THBS1, TIMP3, EPHA7, EPHA3, TLR3, F2RL1, F3, ACSL1, SERINC2, PDCD4, PCDH11X, NCKAP1L, EVI5, CXCR4, IL1B, TRIB2, ITGB3, GPRC5A, TOR1AIP2, GPR65, CST7, LGR5, SAMD5, SH3RF2, SERPINB7, TNFSF10, CRADD, GMFG, MEF2C, MAP3K1, NRK, ASAP1, RAD50, AFAP1L2, SERPINB2, NUAK1 |  |
| Blood Vessel Morphogenesis | 12 | 10 | 3.28E-08 | 0.023 | | ZMIZ1, HEG1, NOG, GJA1, VEGFA, LAMA1, BMP4, EFNB2, TGFB2, THBS1, TGFBR3, HES1 |  |
| Regulation Of Transport | 59 | 2 | 4.09E-08 | 0.026 | | ARRB1, PKIA, ATP2B4, PDE3B, ABCA1, PIK3CG, PLCB4, PLTP, MCTP2, CARD8, CLU, CLIC2, SCN5A, SCP2, SCN9A, CCL2, BMP4, C4BPB, RAB27B, SYTL3, SOX4, CEMIP, DMD, ARL2BP, DPYSL2, TRIM6, CPT1A, PLCB1, CYP2J2, PTPN22, VEGFA, TGFB2, THBS1, STOM, EPHA3, F2RL1, TNFRSF21, PCDH17, NCKAP1L, GBP1, GJA1, CXCR4, IL1B, IL13RA2, INHBB, CLIC3, ITGB3, STXBP6, CASK, HES1, MEF2C, KCNQ3, IL1RL1, KISS1, LCP1, KCNIP3, LIPG, CLIC6, NEO1 |  |
| Negative Regulation Of Cell Differentiation | 34 | 3 | 4.24E-08 | 0.024 | | PDCD4, GRB14, PLK2, HIST2H4B, HLA-A, ABCA1, GBP1, PRICKLE1, IL1B, TRIB2, ITGB3, BMP4, FOXA1, PTPRS, HES1, ADAMTS12, ZNF608, TRIM6, NOG, SNAI2, ASAP1, FGF13, BCL11A, VEGFA, LPAR1, S1PR3, EFNB2, TGFB2, TGFBR3, EPHA7, EPHA3, TLR3, NEO1, EFEMP1 |  |
| Positive Regulation Of Kinase Activity | 28 | 4 | 4.38E-08 | 0.027 | | ARRB1, ATP2B4, NCKAP1L, CXCR4, PIK3CG, ROBO1, IL1B, ITGB3, CLU, BMP4, SAMD5, CCND2, CEMIP, MAP3K1, NRK, CSF1, FGF13, RAD50, AFAP1L2, VEGFA, LPAR1, TGFA, TGFB2, THBS1, EPHA7, EPHA3, TLR3, ACSL1 |  |
| Signal Transduction | 135 | 1 | 4.56E-08 | 0.028 | | ARHGDIB, ARRB1, BCL2A1, ABCA1, CD22, CD33, ADGRE5, CHRM3, CLU, CLIC2, BMP4, CYP24A1, DMD, DPYSL2, OR2M3, PIK3AP1, OR5P2, CSF1, FGF13, LPAR1, S1PR3, EFNB2, EPHA7, EPHA3, F2RL1, F3, F2RL2, F13A1, ACSL1, EFEMP1, GPR1, GRB14, NCKAP1L, HLA-A, HLA-B, GBP1, GBP2, PRICKLE1, GJA1, GNGT2, IGFBP4, IL1B, IL13RA2, INHBB, ITGB3, CD82, HLA-DRA, FOXA1, HES1, MEF2C, MAP3K1, MMP3, MMP1, NRK, KISS1, LCP1, LTBP1, PLPP4, SERPINB2, OR5B21, NEO1, FAM83B, PDE3B, PIK3CG, PLAU, ADGRF1, PLCB4, PLXNA2, PLXNA1, ROBO1, RRBP1, CCL2, LAMA1, RAB27B, RGS10, SOX4, SDC2, SNAI2, TUFT1, VEGFA, TGFA, TGFB2, TGFBR3, TGM2, TLR3, CXCR4, NRP2, CLIC3, GPRC5A, SORBS2, GPR65, CDC42BPA, LGR5, TNFSF10, CRADD, PTGES, FCGR2C, IL1RL1, NOG, TSPAN2, OPTN, RAPGEF5, NUAK1, PLK2, PDE10A, CNKSR2, IL24, IRAK3, ARL2BP, PLCB1, ADGRL3, PTPN22, PITPNC1, TIAM2, FLRT2, TNFRSF21, PDCD4, OR2H1, CDON, KCNIP3, G0S2, SHC3, TENM3, MCTP2, DOCK10, HRASLS, SORCS2, PMEPA1, NXN, HHIP, GPR135, TNS3, PORCN, CPEB4, FAT4 |  |
| Regulation Of Molecular Function | 85 | 2 | 4.62E-08 | 0.019 | | ARHGDIB, PLK2, ARRB1, ATP2B4, ARRDC4, BCL2A1, CARD8, CLU, CLIC2, BMP4, BMP1, IL24, IRAK3, CCND2, DMD, ARL2BP, TRIM6, CSF1, PLCB1, TBC1D30, FGF13, PTPN22, TIAM2, LPAR1, STOM, EPHA7, EPHA3, LYPD6B, F2RL1, F3, ACSL1, EFEMP1, PDCD4, PCDH11X, NCKAP1L, GJA1, IL1B, INHBB, TRIB2, ITGB3, TOR1AIP2, FOXA1, HES1, SH3RF2, CDON, MEF2C, MAP3K1, NRK, ASAP1, SERPINB2, PKIA, PIK3CG, PLAU, ADGRF1, PLXNA2, PLXNA1, ROBO1, CCL2, HMSD, DOCK10, RGS10, CEMIP, VEGFA, TGFA, TGFB2, THBS1, TGFBR3, TIMP3, TLR3, SERINC2, EVI5, CXCR4, GPRC5A, GPR65, CST7, LGR5, SAMD5, SERPINB7, TNFSF10, CRADD, GMFG, NOG, RAD50, AFAP1L2, NUAK1 |  |
| Positive Regulation Of Phosphorus Metabolic Process | 42 | 3 | 4.80E-08 | 0.021 | | ARRB1, ATP2B4, MIR181B1, PIK3CG, ROBO1, CLU, CCL2, BMP4, IL24, CCND2, CEMIP, ARL2BP, TRIM6, CSF1, PLCB1, FGF13, PTPN22, VEGFA, LPAR1, TGFA, TGFB2, THBS1, EPHA7, EPHA3, TLR3, F2RL1, ACSL1, NCKAP1L, CXCR4, IGFBP4, IL1B, INHBB, ITGB3, SAMD5, HES1, CDON, MEF2C, MAP3K1, NRK, KISS1, RAD50, AFAP1L2 |  |
| Positive Regulation Of Phosphate Metabolic Process | 42 | 3 | 5.46E-08 | 0.026 | | ARRB1, ATP2B4, MIR181B1, PIK3CG, ROBO1, CLU, CCL2, BMP4, IL24, CCND2, CEMIP, ARL2BP, TRIM6, CSF1, PLCB1, FGF13, PTPN22, VEGFA, LPAR1, TGFA, TGFB2, THBS1, EPHA7, EPHA3, TLR3, F2RL1, ACSL1, NCKAP1L, CXCR4, IGFBP4, IL1B, INHBB, ITGB3, SAMD5, HES1, CDON, MEF2C, MAP3K1, NRK, KISS1, RAD50, AFAP1L2 |  |
| Regulation Of Cellular Component Organization | 71 | 2 | 5.46E-08 | 0.026 | | ARHGDIB, PLK2, ARRB1, ABCA1, NAV3, ADGRE5, SLITRK1, CLU, BMP4, C4BPB, ESM1, IRAK3, CCND2, DMD, DPYSL2, PLCB1, ADGRL3, TBC1D30, FGF13, LPAR1, FLRT2, EFNB2, EPHA7, EPHA3, F2RL1, EFEMP1, FBP1, HAS2, NCKAP1L, HLA-A, GBP1, GJA1, IGFBP4, IL1B, ITGB3, STXBP6, HES1, MEF2C, MAP3K1, MMP3, MMP1, LCP1, ASAP1, BCL11A, NEO1, PLXNA2, PLXNA1, TENM3, ROBO1, BAIAP2L1, CCL2, PTPRS, HRASLS, SDC2, PMEPA1, PRDM9, SNAI2, VEGFA, WT1, TGFA, TGFB2, THBS1, EVI5, CXCR4, GPR65, COLGALT1, CASK, TNFSF10, GMFG, RAD50, OPTN |  |
| Regulation Of Anatomical Structure Morphogenesis | 42 | 3 | 5.76E-08 | 0.022 | | PLK2, MIR181B1, PDE3B, PLAU, PLXNA2, PLXNA1, ROBO1, SLITRK1, CCL2, BMP4, PTPRS, DPYSL2, TCF4, SDC2, CSF1, SNAI2, FGF13, VEGFA, WT1, HHIP, LPAR1, EFNB2, TGFB2, THBS1, TGFBR3, EPHA7, TLR3, F3, HAS2, GATA6, GBP1, PRICKLE1, GJA1, CXCR4, IL1B, ITGB3, CASK, HES1, ADAMTS12, MEF2C, NOG, BCL11A |  |
| Positive Regulation Of Cellular Protein Metabolic Process | 52 | 2 | 6.07E-08 | 0.026 | | PLK2, ARRB1, ATP2B4, ARRDC4, MIR181B1, TSPYL5, PIK3CG, ROBO1, CARD8, CLU, CCL2, BMP4, IL24, CCND2, SOX4, CEMIP, ARL2BP, TRIM6, CSF1, PLCB1, SNAI2, FGF13, PTPN22, VEGFA, LPAR1, TGFA, TGFB2, THBS1, EPHA7, EPHA3, TLR3, F2RL1, F3, ACSL1, NCKAP1L, PRICKLE1, CXCR4, IGFBP4, IL1B, INHBB, TRIB2, ITGB3, SAMD5, HES1, TNFSF10, CRADD, CDON, MAP3K1, NRK, KISS1, RAD50, AFAP1L2 |  |
| Positive Regulation Of Protein Metabolic Process | 54 | 2 | 6.44E-08 | 0.024 | | PLK2, ARRB1, ATP2B4, ARRDC4, MIR181B1, TSPYL5, PIK3CG, ROBO1, CARD8, CLU, CCL2, BMP4, IL24, C4BPB, CCND2, SOX4, CEMIP, ARL2BP, TRIM6, CSF1, PLCB1, SNAI2, FGF13, PTPN22, VEGFA, LPAR1, TGFA, TGFB2, THBS1, EPHA7, EPHA3, TLR3, F2RL1, F3, ACSL1, NCKAP1L, PRICKLE1, GJA1, CXCR4, IGFBP4, IL1B, INHBB, TRIB2, ITGB3, SAMD5, HES1, TNFSF10, CRADD, CDON, MAP3K1, NRK, KISS1, RAD50, AFAP1L2 |  |
| Regulation Of Kinase Activity | 35 | 3 | 6.65E-08 | 0.024 | | PDCD4, ARRB1, PKIA, ATP2B4, NCKAP1L, CXCR4, PIK3CG, ROBO1, IL1B, TRIB2, ITGB3, GPRC5A, CLU, BMP4, SAMD5, IRAK3, CCND2, GMFG, CEMIP, MAP3K1, NRK, CSF1, FGF13, RAD50, AFAP1L2, PTPN22, VEGFA, LPAR1, TGFA, TGFB2, THBS1, EPHA7, EPHA3, TLR3, ACSL1 |  |
| Regulation Of Cellular Response To Growth Factor Stimulus | 19 | 5 | 7.50E-08 | 0.027 | | ADAMTS12, DMD, SHISA2, TCF4, GATA6, NOG, PMEPA1, LTBP1, ROBO1, IL1B, VEGFA, ITGB3, HHIP, BMP4, THBS1, TGFBR3, CASK, HES1, NEO1 |  |
| Regulation Of Neurogenesis | 35 | 3 | 7.53E-08 | 0.028 | | TNFRSF21, PLK2, HLA-A, CXCR4, PLXNA2, PLXNA1, TENM3, ROBO1, SLITRK1, IL1B, BMP4, FOXA1, PTPRS, CASK, HES1, CCND2, CDON, MEF2C, DMD, DPYSL2, TCF4, SDC2, NOG, CSF1, ASAP1, FGF13, BCL11A, VEGFA, LPAR1, FLRT2, EFNB2, EPHA7, EPHA3, NEO1, EFEMP1 |  |
| Regulation Of Nervous System Development | 37 | 3 | 8.90E-08 | 0.026 | | TNFRSF21, PLK2, HLA-A, CXCR4, PLXNA2, PLXNA1, TENM3, ROBO1, ADGRE5, SLITRK1, IL1B, BMP4, FOXA1, PTPRS, CASK, HES1, CCND2, CDON, MEF2C, DMD, DPYSL2, TCF4, SDC2, NOG, CSF1, ADGRL3, ASAP1, FGF13, BCL11A, VEGFA, LPAR1, FLRT2, EFNB2, EPHA7, EPHA3, NEO1, EFEMP1 |  |
| Positive Regulation Of Biological Process | 138 | 1 | 9.32E-08 | 0.026 | | ARRB1, ATP2B4, ARRDC4, BCL2A1, AADAC, ABCA1, TSPYL5, NAV3, EFHC1, ADGRE5, SLITRK1, FBXO32, CHRM3, CLU, CLIC2, BMP4, BMP1, C4BPB, CCND2, DMD, DPYSL2, TRIM6, PIK3AP1, CPT1A, CSF1, CYP2J2, FGF13, LPAR1, S1PR3, EFNB2, STOM, EPHA7, EPHA3, EYA4, F2RL1, F3, F2RL2, ACSL1, EFEMP1, GRB14, HAS2, NCKAP1L, HLA-A, HLA-B, GATA6, PRICKLE1, GJA1, IGFBP4, IL1B, IL13RA2, INHBB, ITGB3, HLA-DRA, FOXA1, HES1, MEF2C, MAP3K1, MMP3, MMP1, NRK, KISS1, LCP1, LTBP1, PLPP4, ZNF438, NEO1, PIK3CG, PLAU, ADGRF1, PLXNA2, PLTP, PLXNA1, ROBO1, SCN5A, SCP2, CCL2, HMSD, RAB27B, SOX4, TBXAS1, TCF4, SNAI2, VEGFA, WT1, TGFA, TGFB2, THBS1, TGFBR3, TGM2, TIMP3, TLR3, SRPX, CXCR4, NRP2, GPR65, LGR5, CASK, SAMD5, SERPINB7, TNFSF10, CRADD, PDLIM1, IL1RL1, NOG, LIPG, RAD50, OPTN, PLK2, MIR181B1, CARD8, IL24, ESM1, IRAK3, ARL2BP, PLCB1, ADGRL3, PTPN22, TIAM2, FLRT2, ABI3BP, TNFRSF21, PDCD4, TRIB2, CDON, G0S2, ASAP1, BCL11A, TENM3, LMO3, BAIAP2L1, SLC44A2, ZMIZ1, CEMIP, HEG1, PRDM9, TNS3, COLGALT1, AFAP1L2 |  |
| Regulation Of Neuron Differentiation | 31 | 3 | 9.68E-08 | 0.018 | | PLK2, HLA-A, CXCR4, PLXNA2, PLXNA1, TENM3, ROBO1, SLITRK1, IL1B, BMP4, FOXA1, PTPRS, CASK, HES1, CCND2, CDON, MEF2C, DMD, DPYSL2, TCF4, SDC2, ASAP1, FGF13, BCL11A, VEGFA, LPAR1, EFNB2, EPHA7, EPHA3, NEO1, EFEMP1 |  |
| Locomotion | 41 | 3 | 1.01E-07 | 0.027 | | ATP2B4, PIK3CG, PLAU, PLXNA2, PLTP, ROBO1, EFHC1, CCL2, BMP4, SDC2, ADGRL3, SNAI2, FGF13, VEGFA, WT1, LPAR1, FLRT2, EFNB2, TGFB2, THBS1, TGFBR3, EPHA7, EPHA3, F2RL1, SLC7A5, GRB14, NCKAP1L, TNS3, GJA1, CXCR4, NRP2, IL1B, ITGB3, CDC42BPA, HES1, ADAMTS12, MEF2C, MAFIP, MMP1, LCP1, NEO1 |  |
| Regulation Of Transferase Activity | 37 | 3 | 1.22E-07 | 0.025 | | PDCD4, ARRB1, PKIA, ATP2B4, ARRDC4, NCKAP1L, CXCR4, PIK3CG, ROBO1, IL1B, TRIB2, ITGB3, GPRC5A, CLU, BMP4, SAMD5, IRAK3, CCND2, GMFG, CEMIP, MAP3K1, NRK, CSF1, FGF13, RAD50, AFAP1L2, PTPN22, VEGFA, LPAR1, TGFA, TGFB2, THBS1, EPHA7, EPHA3, TLR3, ACSL1, SERINC2 |  |
| Regulation Of Epithelial Cell Proliferation | 22 | 4 | 1.25E-07 | 0.026 | | MEF2C, HAS2, NOG, GJA1, PLAU, SNAI2, ROBO1, NRP2, VEGFA, ITGB3, SCN5A, CCL2, BMP4, EFNB2, TGFA, TGFB2, THBS1, TGFBR3, CASK, HES1, F3, CCND2 |  |
| Negative Regulation Of Intracellular Signal Transduction | 26 | 4 | 1.38E-07 | 0.028 | | PDCD4, ARRB1, ATP2B4, PDE10A, BCL2A1, PDE3B, GBP1, IL1B, CARD8, LMO3, CLU, CLIC2, BMP4, IRAK3, DMD, MMP3, IL1RL1, SNAI2, OPTN, PTPN22, LPAR1, TGFB2, THBS1, TIMP3, F2RL1, FBP1 |  |
| Regulation Of Cytokine Production | 30 | 3 | 1.56E-07 | 0.027 | | TNFRSF21, PDCD4, ARRB1, NCKAP1L, HLA-A, GATA6, GBP1, NAV3, IL1B, CARD8, INHBB, TRIB2, CLU, PTPRS, SERPINB7, IRAK3, HEG1, TRIM6, IL1RL1, PLCB1, SNAI2, CYP2J2, AFAP1L2, OPTN, PTPN22, S1PR3, TGFB2, THBS1, TLR3, F2RL1 |  |
| Regulation Of Protein Kinase Activity | 32 | 3 | 1.57E-07 | 0.027 | | PDCD4, ARRB1, PKIA, ATP2B4, NCKAP1L, CXCR4, PIK3CG, ROBO1, IL1B, TRIB2, ITGB3, GPRC5A, CLU, BMP4, SAMD5, IRAK3, CCND2, GMFG, CEMIP, MAP3K1, NRK, CSF1, FGF13, AFAP1L2, PTPN22, VEGFA, LPAR1, TGFA, TGFB2, THBS1, TLR3, ACSL1 |  |
| Regulation Of Neuron Projection Development | 26 | 4 | 1.79E-07 | 0.026 | | PLK2, HLA-A, CXCR4, PLXNA2, PLXNA1, TENM3, ROBO1, SLITRK1, BMP4, PTPRS, CASK, HES1, MEF2C, DMD, DPYSL2, SDC2, ASAP1, FGF13, BCL11A, VEGFA, LPAR1, EFNB2, EPHA7, EPHA3, NEO1, EFEMP1 |  |
| Positive Regulation Of Protein Kinase Activity | 25 | 4 | 2.00E-07 | 0.027 | | ARRB1, ATP2B4, NCKAP1L, CXCR4, PIK3CG, ROBO1, IL1B, ITGB3, CLU, BMP4, SAMD5, CCND2, CEMIP, MAP3K1, NRK, CSF1, FGF13, AFAP1L2, VEGFA, LPAR1, TGFA, TGFB2, THBS1, TLR3, ACSL1 |  |
| Cellular Response To Organic Substance | 57 | 2 | 2.17E-07 | 0.027 | | ATP2B4, PDE3B, ABCA1, PIK3CG, PLAU, CD68, FBXO32, CLU, SCP2, CCL2, BMP4, IL24, CCND2, SOX4, TRIM6, CPT1A, PLCB1, SNAI2, PTPN22, VEGFA, WT1, LPAR1, THBS1, TIMP3, EPHA3, TLR3, FBP1, TNFRSF21, PDCD4, GRB14, HAS2, HLA-A, GATA6, GBP1, GBP2, GBP3, LAPTM5, GJA1, CXCR4, NRP2, IL1B, INHBB, ITGB3, CPEB4, HMGCS1, HES1, ADAMTS12, MEF2C, MMP3, MMP1, IL1RL1, SNX10, NOG, LTBP1, BCL11A, OPTN, GDAP1 |  |
| Response To Nitrogen Compound | 42 | 3 | 2.19E-07 | 0.023 | | ATP2B4, PDE3B, ABCA1, PIK3CG, PLAU, SCP2, BMP4, IRAK3, RGS10, CCND2, DPYSL2, SDC2, PLCB1, ADGRL3, PTPN22, VEGFA, WT1, TGFB2, THBS1, TGFBR3, TIMP3, TLR3, F3, FBP1, GRB14, GATA6, GJA1, CXCR4, IL1B, INHBB, ITGB3, CPEB4, HMGCS1, HES1, TNFSF10, MEF2C, MMP3, MMP1, KCNQ3, IL1RL1, BCL11A, OPTN |  |
| Response To Fluid Shear Stress | 8 | 15 | 2.30E-07 | 0.025 | | MEF2C, MMP1, HAS2, ABCA1, TGFB2, GJA1, PLAU, F3 |  |
| Morphogenesis Of An Epithelium | 19 | 5 | 2.83E-07 | 0.020 | | MEF2C, MAP3K1, NOG, GJA1, CSF1, CXCR4, CTSD, PLXNA1, VEGFA, WT1, HHIP, LAMA1, FAT4, BMP4, EFNB2, FOXA1, TGM2, EPHA7, HES1 |  |
| Cell Motility | 38 | 3 | 3.14E-07 | 0.027 | | SLC7A5, GRB14, ATP2B4, NCKAP1L, TNS3, GJA1, CXCR4, PIK3CG, PLAU, PLXNA2, PLTP, ROBO1, EFHC1, NRP2, IL1B, ITGB3, CCL2, CDC42BPA, HES1, ADAMTS12, MEF2C, MAFIP, MMP1, SDC2, LCP1, ADGRL3, SNAI2, FGF13, VEGFA, WT1, LPAR1, EFNB2, TGFB2, THBS1, TGFBR3, EPHA3, F2RL1, NEO1 |  |
| Positive Regulation Of Mapk Cascade | 26 | 4 | 3.34E-07 | 0.025 | | ARRB1, MIR181B1, CXCR4, PIK3CG, ROBO1, IGFBP4, IL1B, ITGB3, CCL2, BMP4, SAMD5, CDON, MAP3K1, NRK, KISS1, PLCB1, PTPN22, VEGFA, LPAR1, TGFA, TGFB2, THBS1, EPHA7, EPHA3, TLR3, F2RL1 |  |
| Cell-Cell Adhesion | 23 | 4 | 3.77E-07 | 0.026 | | PCDH17, PCDH11X, MYL9, PIK3CG, TENM3, ROBO1, CDH11, IL1B, ITGB3, BAIAP2L1, STXBP6, PCDHB6, FAT4, CDON, PCDH11Y, DSP, PCDHB2, PDLIM1, LAMA3, ADGRL3, PCDHB5, TGFB2, PCDHB16 |  |
| Negative Regulation Of Cytokine Production | 17 | 5 | 3.85E-07 | 0.027 | | TNFRSF21, PDCD4, ARRB1, NCKAP1L, IL1RL1, GATA6, GBP1, NAV3, PTPN22, CARD8, INHBB, TRIB2, PTPRS, TGFB2, THBS1, F2RL1, IRAK3 |  |
| Positive Regulation Of Cell Proliferation | 38 | 3 | 4.63E-07 | 0.026 | | ARRB1, HAS2, NCKAP1L, HLA-A, MIR181B1, TNS3, GATA6, TSPYL5, PLAU, NRP2, IL1B, ITGB3, CLU, SCN5A, BMP4, IL24, ESM1, HES1, SERPINB7, CCND2, SOX4, CDON, ZMIZ1, MEF2C, NOG, CSF1, OPTN, PTPN22, VEGFA, S1PR3, EFNB2, TGFA, TGFB2, THBS1, TGFBR3, TGM2, F3, EFEMP1 |  |
| Regulation Of Response To External Stimulus | 33 | 3 | 5.44E-07 | 0.025 | | PDCD4, NCKAP1L, GJA1, CXCR4, PIK3CG, PLAU, ROBO1, IL1B, CARD8, CLU, CCL2, PTPRS, C4BPB, CASK, ADAMTS12, MTUS1, TRIM6, IL1RL1, PIK3AP1, CSF1, SNAI2, OPTN, PTPN22, VEGFA, SERPINB2, LPAR1, EFNB2, THBS1, TGM2, TLR3, F2RL1, F3, NEO1 |  |
| Regulation Of Cell Death | 55 | 2 | 5.45E-07 | 0.025 | | PLK2, ARRB1, BCL2A1, PIK3CG, ROBO1, EFHC1, FBXO32, CARD8, CLU, SCP2, CCL2, BMP4, IL24, IRAK3, CCND2, SOX4, SNAI2, TIAM2, VEGFA, WT1, HHIP, LPAR1, EFNB2, TGFA, TGFB2, THBS1, TGFBR3, TGM2, TIMP3, EPHA7, EPHA3, EYA4, TLR3, F3, PDCD4, SRPX, NCKAP1L, GATA6, CXCR4, IL1B, INHBB, ITGB3, CPEB4, FOXA1, TNFSF10, CRADD, MEF2C, MAP3K1, MMP3, NOG, KCNIP3, G0S2, OPTN, SERPINB2, NEO1 |  |
| Regulation Of Cell Proliferation | 55 | 2 | 5.47E-07 | 0.023 | | ARRB1, MIR181B1, TSPYL5, PLAU, CD33, ROBO1, CLU, SCN5A, SCP2, CCL2, BMP4, IL24, ESM1, CCND2, SOX4, ZMIZ1, CSF1, SNAI2, PTPN22, VEGFA, WT1, S1PR3, EFNB2, TGFA, TGFB2, THBS1, TGFBR3, TGM2, F3, EFEMP1, TNFRSF21, PDCD4, HAS2, SRPX, NCKAP1L, HLA-A, TNS3, GATA6, GJA1, NRP2, IL1B, ITGB3, LGR5, CASK, HES1, SERPINB7, CDON, PTGES, MEF2C, NRK, IL1RL1, KISS1, NOG, OPTN, NUAK1 |  |
| Positive Regulation Of Cellular Component Organization | 42 | 2 | 5.47E-07 | 0.023 | | ARRB1, NAV3, PLXNA2, PLXNA1, TENM3, ROBO1, ADGRE5, SLITRK1, BAIAP2L1, CLU, CCL2, BMP4, DMD, PLCB1, ADGRL3, PRDM9, SNAI2, VEGFA, LPAR1, FLRT2, TGFA, TGFB2, EPHA3, F2RL1, EFEMP1, HAS2, NCKAP1L, CXCR4, IL1B, ITGB3, GPR65, COLGALT1, CASK, TNFSF10, MAP3K1, MMP3, MMP1, LCP1, ASAP1, BCL11A, RAD50, OPTN |  |
| Regulation Of Plasma Membrane Bounded Cell Projection Organization | 30 | 3 | 5.81E-07 | 0.024 | | PLK2, HLA-A, EVI5, CXCR4, NAV3, PLXNA2, PLXNA1, TENM3, ROBO1, SLITRK1, BMP4, PTPRS, CASK, HES1, MEF2C, DMD, DPYSL2, SDC2, TBC1D30, ASAP1, FGF13, BCL11A, VEGFA, LPAR1, EFNB2, EPHA7, EPHA3, F2RL1, NEO1, EFEMP1 |  |
| Negative Regulation Of Protein Metabolic Process | 41 | 2 | 6.18E-07 | 0.025 | | ARRB1, PKIA, ATP2B4, BCL2A1, MIR181B1, CARD8, LMO3, CLU, HMSD, BMP4, IL24, C4BPB, IRAK3, SOX4, DMD, PMEPA1, CST4, PTPN22, SAMSN1, VEGFA, WT1, NXN, THBS1, TIMP3, F2RL1, PDCD4, NCKAP1L, GBP1, IL1B, INHBB, TRIB2, ITGB3, CPEB4, GPRC5A, CST7, SH3RF2, SERPINB7, GMFG, NOG, CTDSPL, SERPINB2 |  |
| Extracellular Structure Organization | 20 | 4 | 6.33E-07 | 0.024 | | CRISPLD2, MMP3, MMP1, HAS2, ABCA1, LAMA3, LAMA4, LCP1, PLTP, LIPG, MPZL3, ITGB3, WT1, LAMA1, FLRT2, BMP1, TGFB2, THBS1, TLL1, ABI3BP |  |
| System Development | 35 | 3 | 6.33E-07 | 0.026 | | SLC7A5, CXCR4, NAV3, PLXNA2, ROBO1, IGFBP4, NRP2, CDH11, SLITRK1, CHRM3, PCDHB6, BMP4, BMP1, HES1, SOX4, MEF2C, HEG1, DPYSL2, TCF4, PCDHB2, SDC2, NOG, PRDM8, FGF13, SHC3, VEGFA, WT1, NXN, RAPGEF5, EFNB2, TGFB2, TIMP3, EPHA7, TLL1, NEO1 |  |
| Regulation Of Cell Projection Organization | 30 | 3 | 7.17E-07 | 0.025 | | PLK2, HLA-A, EVI5, CXCR4, NAV3, PLXNA2, PLXNA1, TENM3, ROBO1, SLITRK1, BMP4, PTPRS, CASK, HES1, MEF2C, DMD, DPYSL2, SDC2, TBC1D30, ASAP1, FGF13, BCL11A, VEGFA, LPAR1, EFNB2, EPHA7, EPHA3, F2RL1, NEO1, EFEMP1 |  |
| Response To Organic Cyclic Compound | 40 | 2 | 7.89E-07 | 0.025 | | ATP2B4, ABCA1, PIK3CG, PLAU, FBXO32, SCN5A, SCP2, CCL2, BMP4, IRAK3, CCND2, CYP24A1, DPYSL2, SDC2, CPT1A, PLCB1, ADGRL3, CYP2J2, VEGFA, WT1, TGFB2, THBS1, TIMP3, TLR3, F3, ACSL1, GATA6, GJA1, CXCR4, IL1B, INHBB, HMGCS1, FOXA1, HES1, PTGES, MEF2C, MMP3, MMP1, LTBP1, GDAP1 |  |
| Negative Regulation Of Neuron Projection Development | 13 | 6 | 1.11E-06 | 0.024 | | PLK2, HLA-A, ASAP1, FGF13, BCL11A, LPAR1, EFNB2, PTPRS, EPHA7, EPHA3, HES1, NEO1, EFEMP1 |  |
| Regulation Of Programmed Cell Death | 51 | 2 | 1.14E-06 | 0.024 | | PLK2, ARRB1, BCL2A1, PIK3CG, ROBO1, EFHC1, FBXO32, CARD8, CLU, SCP2, CCL2, BMP4, IL24, IRAK3, CCND2, SOX4, SNAI2, TIAM2, VEGFA, WT1, HHIP, LPAR1, TGFA, TGFB2, THBS1, TGFBR3, TGM2, TIMP3, EPHA7, EPHA3, EYA4, TLR3, F3, PDCD4, SRPX, NCKAP1L, GATA6, CXCR4, IL1B, INHBB, CPEB4, FOXA1, TNFSF10, CRADD, MEF2C, MAP3K1, NOG, KCNIP3, G0S2, OPTN, SERPINB2 |  |
| Cell Migration | 34 | 3 | 1.27E-06 | 0.022 | | SLC7A5, GRB14, NCKAP1L, TNS3, GJA1, CXCR4, PIK3CG, PLAU, PLXNA2, ROBO1, EFHC1, NRP2, IL1B, ITGB3, CCL2, CDC42BPA, HES1, ADAMTS12, MEF2C, MMP1, SDC2, LCP1, ADGRL3, SNAI2, FGF13, VEGFA, LPAR1, EFNB2, TGFB2, THBS1, TGFBR3, EPHA3, F2RL1, NEO1 |  |
| Outflow Tract Morphogenesis | 8 | 12 | 1.29E-06 | 0.024 | | MEF2C, VEGFA, BMP4, TGFB2, THBS1, TGFBR3, NOG, HES1 |  |
| Positive Regulation Of Developmental Process | 47 | 2 | 1.51E-06 | 0.019 | | PLK2, MIR181B1, PLXNA2, PLXNA1, TENM3, ROBO1, ADGRE5, SLITRK1, LMO3, CLU, BMP4, BMP1, IRAK3, ZMIZ1, DMD, TCF4, CSF1, PLCB1, ADGRL3, SNAI2, VEGFA, WT1, LPAR1, FLRT2, EFNB2, TGFB2, THBS1, TGFBR3, EPHA3, TLR3, F3, HAS2, NCKAP1L, HLA-A, GATA6, GJA1, CXCR4, IL1B, ITGB3, FOXA1, CASK, HES1, SERPINB7, CDON, MEF2C, NOG, BCL11A |  |
| Response To Oxygen-Containing Compound | 55 | 2 | 1.52E-06 | 0.023 | | ATP2B4, PDE3B, ABCA1, PIK3CG, PLAU, FBXO32, SCP2, CCL2, BMP4, IL24, IRAK3, CCND2, SOX4, CYP24A1, DPYSL2, TBXAS1, TRIM6, CPT1A, PLCB1, ADGRL3, PTPN22, VEGFA, WT1, LPAR1, TGFB2, THBS1, TGFBR3, TIMP3, EPHA3, F3, ACSL1, FBP1, PDCD4, GRB14, ILDR2, GATA6, GBP2, GJA1, IL1B, INHBB, ITGB3, CPEB4, HMGCS1, FOXA1, HES1, TNFSF10, PTGES, MEF2C, MMP3, MMP1, IL1RL1, LTBP1, BCL11A, OPTN, GDAP1 |  |
| Negative Regulation Of Cell Projection Organization | 14 | 6 | 1.77E-06 | 0.022 | | PLK2, HLA-A, TBC1D30, ASAP1, FGF13, BCL11A, LPAR1, EFNB2, PTPRS, EPHA7, EPHA3, HES1, NEO1, EFEMP1 |  |
| Regulation Of Glomerulus Development | 5 | 29 | 1.91E-06 | 0.024 | | ITGB3, WT1, BMP4, NOG, SERPINB7 |  |
| Response To Organonitrogen Compound | 38 | 2 | 1.92E-06 | 0.013 | | ATP2B4, PDE3B, ABCA1, PIK3CG, PLAU, SCP2, IRAK3, RGS10, CCND2, DPYSL2, SDC2, PLCB1, ADGRL3, PTPN22, VEGFA, WT1, TGFB2, TGFBR3, TIMP3, F3, FBP1, GRB14, GATA6, GJA1, CXCR4, IL1B, INHBB, ITGB3, CPEB4, HMGCS1, HES1, TNFSF10, MEF2C, MMP3, MMP1, IL1RL1, BCL11A, OPTN |  |
| Negative Regulation Of Cell Development | 19 | 4 | 2.01E-06 | 0.024 | | GRB14, PLK2, HLA-A, GBP1, NOG, ASAP1, FGF13, BCL11A, IL1B, LPAR1, S1PR3, BMP4, EFNB2, PTPRS, EPHA7, EPHA3, HES1, NEO1, EFEMP1 |  |
| Negative Regulation Of Cartilage Development | 6 | 20 | 2.01E-06 | 0.025 | | ADAMTS12, BMP4, TGFB2, NOG, SNAI2, EFEMP1 |  |
| Cardiac Septum Morphogenesis | 9 | 10 | 2.05E-06 | 0.015 | | SOX4, GATA6, NOG, ROBO1, NRP2, BMP4, TGFB2, TGFBR3, HES1 |  |
| Regulation Of Apoptotic Process | 50 | 2 | 2.12E-06 | 0.020 | | PLK2, ARRB1, BCL2A1, PIK3CG, ROBO1, EFHC1, FBXO32, CARD8, CLU, SCP2, CCL2, BMP4, IL24, IRAK3, CCND2, SOX4, SNAI2, TIAM2, VEGFA, WT1, HHIP, LPAR1, TGFA, TGFB2, THBS1, TGFBR3, TGM2, TIMP3, EPHA7, EPHA3, EYA4, TLR3, F3, PDCD4, SRPX, NCKAP1L, GATA6, IL1B, INHBB, CPEB4, FOXA1, TNFSF10, CRADD, MEF2C, MAP3K1, NOG, KCNIP3, G0S2, OPTN, SERPINB2 |  |
| Regulation Of Cell Adhesion Mediated By Integrin | 7 | 14 | 2.13E-06 | 0.022 | | ITGB3, NCKAP1L, PDE3B, TGFB2, PIK3CG, PLAU, SNAI2 |  |
| Response To Gonadotropin | 8 | 11 | 2.30E-06 | 0.017 | | RAD50, INHBB, WT1, SCP2, HMGCS1, GATA6, TGFBR3, EPHA3 |  |
| Movement Of Cell Or Subcellular Component | 45 | 2 | 2.43E-06 | 0.019 | | ATP2B4, PIK3CG, PLAU, PLXNA2, PLTP, PLXNA1, ROBO1, EFHC1, CCL2, DMD, DPYSL2, SDC2, ADGRL3, SNAI2, FGF13, VEGFA, WT1, LPAR1, FLRT2, EFNB2, TGFB2, THBS1, TGFBR3, EPHA7, EPHA3, F2RL1, SLC7A5, GRB14, NCKAP1L, TNS3, GJA1, CXCR4, NRP2, IL1B, ITGB3, CDC42BPA, HES1, ADAMTS12, MEF2C, MAFIP, MMP1, NOG, LCP1, SHC3, NEO1 |  |
| Negative Regulation Of Cellular Protein Metabolic Process | 38 | 2 | 2.73E-06 | 0.023 | | ARRB1, PKIA, ATP2B4, BCL2A1, MIR181B1, CARD8, LMO3, HMSD, BMP4, IL24, C4BPB, IRAK3, SOX4, DMD, PMEPA1, CST4, PTPN22, SAMSN1, VEGFA, WT1, NXN, THBS1, TIMP3, F2RL1, PDCD4, NCKAP1L, GBP1, IL1B, TRIB2, CPEB4, GPRC5A, CST7, SH3RF2, SERPINB7, GMFG, NOG, CTDSPL, SERPINB2 |  |
| Cellular Response To Oxygen-Containing Compound | 39 | 2 | 2.93E-06 | 0.023 | | ATP2B4, PDE3B, ABCA1, PIK3CG, PLAU, FBXO32, SCP2, CCL2, BMP4, IL24, CCND2, SOX4, CPT1A, PLCB1, PTPN22, VEGFA, WT1, LPAR1, THBS1, EPHA3, F3, FBP1, PDCD4, GRB14, GBP2, IL1B, INHBB, ITGB3, CPEB4, HMGCS1, HES1, MEF2C, MMP3, MMP1, IL1RL1, LTBP1, BCL11A, OPTN, GDAP1 |  |
| Response To Endogenous Stimulus | 44 | 2 | 3.14E-06 | 0.023 | | ATP2B4, PDE3B, ABCA1, PIK3CG, PLAU, FBXO32, SCP2, CCL2, BMP4, CCND2, PLCB1, SNAI2, PTPN22, VEGFA, WT1, TGFB2, THBS1, TGFBR3, TIMP3, EPHA3, F3, FBP1, GRB14, HAS2, GATA6, GJA1, IL1B, INHBB, ITGB3, CPEB4, HMGCS1, FOXA1, HES1, TNFSF10, ADAMTS12, MEF2C, MMP3, MMP1, IL1RL1, NOG, LTBP1, BCL11A, RAD50, OPTN |  |
| Positive Regulation Of Small Gtpase Mediated Signal Transduction | 8 | 11 | 3.27E-06 | 0.022 | | CDON, ROBO1, ARRB1, LPAR1, GPR65, CSF1, F2RL1, F2RL2 |  |
| Blood Vessel Development | 12 | 6 | 3.40E-06 | 0.019 | | MEF2C, LAMA4, PRICKLE1, CXCR4, LTBP1, ROBO1, VEGFA, WT1, BMP4, TGFB2, TGFBR3, HES1 |  |
| Regulation Of Chemotaxis | 14 | 5 | 3.61E-06 | 0.023 | | MTUS1, NCKAP1L, CSF1, CXCR4, SNAI2, ROBO1, IL1B, VEGFA, CCL2, LPAR1, EFNB2, THBS1, F2RL1, F3 |  |
| Cardiac Epithelial To Mesenchymal Transition | 6 | 18 | 3.65E-06 | 0.024 | | PDCD4, HAS2, BMP4, TGFB2, TGFBR3, SNAI2 |  |
| Positive Regulation Of Rho Protein Signal Transduction | 6 | 18 | 3.71E-06 | 0.015 | | ROBO1, ARRB1, LPAR1, GPR65, F2RL1, F2RL2 |  |
| Cellular Response To Chemical Stimulus | 60 | 2 | 3.71E-06 | 0.015 | | ATP2B4, PDE3B, ABCA1, PIK3CG, PLAU, CD68, FBXO32, CLU, SCN5A, SCP2, CCL2, BMP4, IL24, CCND2, SOX4, TRIM6, CPT1A, PLCB1, SNAI2, PTPN22, VEGFA, WT1, LPAR1, THBS1, TIMP3, EPHA3, TLR3, F3, FBP1, TNFRSF21, PDCD4, GRB14, HAS2, HLA-A, GATA6, GBP1, GBP2, GBP3, LAPTM5, GJA1, CXCR4, NRP2, IL1B, INHBB, ITGB3, CPEB4, HMGCS1, HES1, ADAMTS12, MEF2C, MMP3, MMP1, KCNQ3, IL1RL1, SNX10, NOG, LTBP1, BCL11A, OPTN, GDAP1 |  |
| Response To Organic Substance | 75 | 2 | 3.71E-06 | 0.021 | | ATP2B4, ABCA1, CD68, FBXO32, CLU, BMP4, IL24, IRAK3, CCND2, CYP24A1, DPYSL2, TRIM6, CPT1A, PLCB1, ADGRL3, CYP2J2, PTPN22, LPAR1, EPHA3, F3, ACSL1, FBP1, TNFRSF21, PDCD4, GRB14, HAS2, HLA-A, GATA6, GBP1, GBP2, GBP3, GJA1, IL1B, INHBB, ITGB3, HMGCS1, FOXA1, HES1, MEF2C, MMP3, MMP1, SNX10, LTBP1, BCL11A, GDAP1, PDE3B, PIK3CG, PLAU, SCN5A, SCP2, CCL2, RGS10, SOX4, TBXAS1, SDC2, SNAI2, VEGFA, WT1, TGFB2, THBS1, TGFBR3, TIMP3, TLR3, ILDR2, LAPTM5, CXCR4, NRP2, CPEB4, TNFSF10, ADAMTS12, PTGES, IL1RL1, NOG, RAD50, OPTN |  |
| Response To Chemical | 85 | 2 | 4.26E-06 | 0.020 | | ARRB1, ATP2B4, ABCA1, AK4, CD68, FBXO32, PTGR1, CLU, BMP4, IL24, IRAK3, CCND2, CYP24A1, DPYSL2, TRIM6, CPT1A, PLCB1, ADGRL3, CYP2J2, FGF13, PTPN22, LPAR1, EPHA3, F3, ACSL1, FBP1, TNFRSF21, PDCD4, GRB14, HAS2, NCKAP1L, HLA-A, GATA6, GBP1, GBP2, GBP3, GJA1, IL1B, INHBB, ITGB3, HMGCS1, FOXA1, HES1, MEF2C, MMP3, MMP1, KCNQ3, SNX10, LTBP1, BCL11A, GDAP1, PON2, PDE3B, PIK3CG, PLAU, SCN5A, SCP2, SCN9A, CCL2, RGS10, SOX4, TBXAS1, SDC2, SNAI2, VEGFA, WT1, TGFA, TGFB2, THBS1, TGFBR3, TIMP3, TLR3, ILDR2, LAPTM5, CXCR4, NRP2, CPEB4, TNFSF10, ADAMTS12, PTGES, IL1RL1, NOG, LIPG, RAD50, OPTN |  |
| Nervous System Development | 22 | 3 | 4.77E-06 | 0.019 | | SLC7A5, CXCR4, NAV3, ROBO1, NRP2, SLITRK1, CHRM3, PCDHB6, HES1, MEF2C, DPYSL2, TCF4, PCDHB2, SDC2, NOG, PRDM8, FGF13, VEGFA, RAPGEF5, EFNB2, EPHA7, NEO1 |  |
| Mesenchymal Cell Differentiation | 9 | 9 | 5.18E-06 | 0.024 | | PDCD4, MEF2C, HAS2, NOG, GJA1, SNAI2, BMP4, TGFB2, TGFBR3 |  |
| Pharyngeal Arch Artery Morphogenesis | 4 | 40 | 5.23E-06 | 0.020 | | BMP4, TGFB2, NOG, HES1 |  |
| Tissue Development | 28 | 3 | 5.45E-06 | 0.011 | | GATA6, GJA1, PLAU, PLXNA2, NRP2, LAMA1, BMP4, FOXA1, HES1, MEF2C, DMD, HEG1, DSP, KCNQ3, SNX10, KISS1, NOG, LAMA3, CSF1, SNAI2, TUFT1, UGCG, VEGFA, WT1, TGFB2, THBS1, TIMP3, EPHA3 |  |
| Cellular Response To Endogenous Stimulus | 33 | 3 | 5.54E-06 | 0.024 | | GRB14, ATP2B4, HAS2, PDE3B, GATA6, GJA1, PIK3CG, PLAU, FBXO32, INHBB, ITGB3, CPEB4, CCL2, BMP4, HMGCS1, HES1, CCND2, ADAMTS12, MEF2C, MMP3, MMP1, IL1RL1, NOG, PLCB1, SNAI2, LTBP1, BCL11A, OPTN, PTPN22, VEGFA, WT1, EPHA3, FBP1 |  |
| Negative Regulation Of Immune System Process | 21 | 3 | 5.54E-06 | 0.023 | | TNFRSF21, HIST2H4B, HLA-A, GBP1, IL13RA2, CCL2, BMP4, PTPRS, C4BPB, HES1, IRAK3, ZNF608, IL1RL1, PIK3AP1, PLCB1, PTPN22, SAMSN1, TGFB2, THBS1, TLR3, F2RL1 |  |
| Animal Organ Morphogenesis | 24 | 3 | 5.99E-06 | 0.024 | | HAS2, GATA6, CXCR4, TENM3, NRP2, SCN5A, CCL2, FAT4, BMP4, FOXA1, SOX4, ZMIZ1, MEF2C, NOG, CSF1, SNAI2, TUFT1, VEGFA, HHIP, FLRT2, EFNB2, TGFB2, TGFBR3, EFEMP1 |  |
| Extracellular Matrix Organization | 17 | 4 | 6.12E-06 | 0.024 | | CRISPLD2, MMP3, MMP1, HAS2, LAMA3, LAMA4, LCP1, MPZL3, ITGB3, WT1, LAMA1, FLRT2, BMP1, TGFB2, THBS1, TLL1, ABI3BP |  |
| Cellular Response To Interleukin-1 | 10 | 7 | 6.57E-06 | 0.024 | | ADAMTS12, MMP3, MMP1, HAS2, GBP1, GBP2, GBP3, INHBB, CCL2, HES1 |  |
| Positive Regulation Of Nervous System Development | 24 | 3 | 7.24E-06 | 0.021 | | CXCR4, PLXNA2, PLXNA1, TENM3, ROBO1, ADGRE5, SLITRK1, IL1B, BMP4, FOXA1, CASK, HES1, CDON, MEF2C, DMD, TCF4, NOG, CSF1, ADGRL3, BCL11A, VEGFA, LPAR1, FLRT2, EPHA3 |  |
| Regulation Of Ion Transport | 27 | 3 | 7.30E-06 | 0.024 | | ATP2B4, GJA1, CXCR4, PIK3CG, PLCB4, IL1B, CLIC3, ITGB3, CLIC2, SCN5A, SCN9A, CCL2, BMP4, CASK, HES1, MEF2C, CEMIP, DMD, DPYSL2, KCNQ3, PLCB1, KCNIP3, PTPN22, CLIC6, TGFB2, THBS1, STOM |  |
| Response To Mechanical Stimulus | 16 | 4 | 7.85E-06 | 0.024 | | MAP3K1, DMD, MMP3, MMP1, GJA1, CXCR4, LTBP1, IL1B, IL13RA2, ITGB3, BMP4, THBS1, TIMP3, TLR3, F3, CRADD |  |
| Ascending Aorta Morphogenesis | 3 | 75 | 8.37E-06 | 0.023 | | SOX4, TGFB2, HES1 |  |
| Biological Regulation | 246 | 1 | 8.52E-06 | 0.008 | | ARHGDIB, ARRB1, ATP2B4, BCL2A1, AADAC, ABCA1, CD22, CD33, ADGRE5, CHRM3, CLU, CLIC2, BMP4, BMP1, C4BPB, CCND2, CYP24A1, DMD, DPYSL2, DSP, CPT1A, CSF1, CST4, CYP2J2, FGF13, FLG, LPAR1, S1PR3, EFNB2, STOM, EPHA7, EPHA3, EYA4, F2RL1, F3, F2RL2, F13A1, ACSL1, EFEMP1, FBP1, GPR1, GRB14, HAS2, NCKAP1L, HLA-A, HLA-B, GATA6, GBP1, GBP2, GJA1, GNGT2, IGFBP4, IL1B, IL13RA2, INHBB, ITGB3, CD82, HLA-DRA, HMGCS1, FOXA1, HES1, MEF2C, MEIS3P1, MAP3K1, MMP3, MMP1, KCNQ3, KISS1, LAMA3, LAMA4, LCP1, LTBP1, SERPINB2, NEO1, PKIA, PDE3B, PIK3CG, PLAU, PLCB4, PLXNA2, PLTP, PLXNA1, ROBO1, RRBP1, SCN5A, SCP2, SCN9A, CCL2, PTPRS, RAB27B, RGS10, SOX4, SSX1, TBXAS1, TCF4, SDC2, SLC22A3, SNAI2, TUFT1, VEGFA, WT1, TGFA, TGFB2, THBS1, TGFBR3, TGM2, TIMP3, TLR3, HIST2H4B, SRPX, ZNF43, EVI5, CXCR4, NRP2, CLIC3, GPRC5A, SORBS2, GPR65, CDC42BPA, CST7, LGR5, CASK, SERPINB7, TNFSF10, CRADD, GMFG, PTGES, CYTIP, FCGR2C, PDLIM1, IL1RL1, NOG, LIPG, TSPAN2, RAD50, OPTN, MBNL2, CTDSPL, RAPGEF5, NUAK1, CLEC2B, PLK2, PDE10A, MYL9, CNKSR2, CARD8, IL24, ESM1, IRAK3, SP140, TDRD7, ARL2BP, PLCB1, ADGRL3, TBC1D30, PTPN22, PITPNC1, TIAM2, FLRT2, ABI3BP, TNFRSF21, PDCD4, PCDH17, PCDH11X, OR2H1, TRIB2, STXBP6, CDON, SNX10, KCNIP3, G0S2, ASAP1, BCL11A, SHC3, CLIC6, TENM3, MCTP2, LMO3, BAIAP2L1, ACOXL, DOCK10, HRASLS, SLC44A2, ZMIZ1, ATP8B2, CEMIP, HEG1, MTUS1, ZNF608, SORCS2, PMEPA1, PRDM9, PRDM8, SAMSN1, NXN, HHIP, SLAMF7, GPR135, TNS3, PORCN, CDK15, CPEB4, FAT4, COLGALT1, ADAMTS12, PARD6B, AFAP1L2, ANKRD30A, ARRDC4, TSPYL5, NAV3, EFHC1, SLITRK1, FBXO32, SYTL3, OR2M3, TRIM6, PIK3AP1, OR5P2, FOXR2, LYPD6B, FAM172BP, PRICKLE1, TOR1AIP2, SH3RF2, NRK, SAMD9L, PLPP4, OR5B21, ZNF438, FAM83B, HECTD4, ADGRF1, SSX8, LAMA1, HMSD, ZNF391, SERINC2, ILDR2, SHISA2, SAMD5, ZNF716, MIR181B1, UCA1, MIR604, MAFIP |  |
| Cytokine-Mediated Signaling Pathway | 24 | 3 | 8.74E-06 | 0.015 | | TNFRSF21, PDCD4, HLA-A, HLA-B, GBP1, GBP2, CXCR4, IL1B, IL13RA2, CCL2, HLA-DRA, IL24, IRAK3, MMP3, MMP1, IL1RL1, CSF1, LCP1, PLCB1, VEGFA, SERPINB2, F3, F13A1, ACSL1 |  |
| Epithelial To Mesenchymal Transition | 8 | 10 | 9.12E-06 | 0.024 | | PDCD4, HAS2, BMP4, TGFB2, TGFBR3, NOG, GJA1, SNAI2 |  |
| Negative Regulation Of Phosphate Metabolic Process | 24 | 3 | 9.32E-06 | 0.018 | | PDCD4, PCDH11X, ARRB1, PKIA, NCKAP1L, GBP1, IL1B, LMO3, TRIB2, GPRC5A, BMP4, SH3RF2, IRAK3, GMFG, DMD, NOG, PMEPA1, PTPN22, SAMSN1, CTDSPL, TGFB2, TIMP3, F2RL1, FBP1 |  |
| Negative Regulation Of Phosphorus Metabolic Process | 24 | 3 | 9.34E-06 | 0.024 | | PDCD4, PCDH11X, ARRB1, PKIA, NCKAP1L, GBP1, IL1B, LMO3, TRIB2, GPRC5A, BMP4, SH3RF2, IRAK3, GMFG, DMD, NOG, PMEPA1, PTPN22, SAMSN1, CTDSPL, TGFB2, TIMP3, F2RL1, FBP1 |  |
| Regulation Of Protein Serine/Threonine Kinase Activity | 22 | 3 | 9.57E-06 | 0.024 | | PDCD4, ARRB1, PKIA, ATP2B4, CXCR4, PIK3CG, ROBO1, IL1B, TRIB2, BMP4, SAMD5, IRAK3, CCND2, CEMIP, MAP3K1, NRK, PTPN22, VEGFA, LPAR1, TGFA, THBS1, ACSL1 |  |
| Morphogenesis Of A Branching Structure | 12 | 6 | 9.94E-06 | 0.024 | | CSF1, CXCR4, CTSD, VEGFA, WT1, HHIP, LAMA1, FAT4, BMP4, FOXA1, TGM2, EPHA7 |  |
| Neuron Projection Guidance | 14 | 5 | 1.03E-05 | 0.022 | | DPYSL2, NOG, CXCR4, PLXNA1, ROBO1, NRP2, SHC3, VEGFA, FLRT2, EFNB2, TGFB2, EPHA7, EPHA3, NEO1 |  |
| Negative Regulation Of Nervous System Development | 17 | 4 | 1.03E-05 | 0.023 | | TNFRSF21, PLK2, HLA-A, NOG, ASAP1, FGF13, BCL11A, IL1B, LPAR1, BMP4, EFNB2, PTPRS, EPHA7, EPHA3, HES1, NEO1, EFEMP1 |  |
| Regulation Of I-Kappab Kinase/Nf-Kappab Signaling | 13 | 5 | 1.04E-05 | 0.023 | | SLC44A2, PLK2, IL1RL1, PIK3AP1, GJA1, OPTN, IL1B, CARD8, LPAR1, TGM2, TLR3, F2RL1, TNFSF10 |  |
| Cell Differentiation | 61 | 2 | 1.04E-05 | 0.022 | | BCL2A1, RBM47, TENM3, ROBO1, RRBP1, LAMA1, BMP4, BMP1, DMKN, DOCK10, SOX4, CYP24A1, TDRD7, ZMIZ1, HEG1, DPYSL2, DSP, TCF4, SDC2, CPT1A, CSF1, PLCB1, PRDM8, SNAI2, FLG, PTPN22, VEGFA, WT1, NXN, LPAR1, EFNB2, TGFB2, TGFBR3, EPHA7, EPHA3, TLL1, EYA4, F2RL1, PDCD4, SLC7A5, ILDR2, HAS2, GATA6, GJA1, CXCR4, NRP2, INHBB, ITGB3, FAT4, FOXA1, LGR5, HES1, CDON, MEF2C, SAMD9L, SNX10, NOG, LAMA3, LAMA4, TSPAN2, BCL11A |  |
| Regulation Of Cellular Process | 223 | 1 | 1.07E-05 | 0.020 | | ARHGDIB, ARRB1, ATP2B4, BCL2A1, AADAC, ABCA1, CD22, CD33, ADGRE5, CHRM3, CLU, CLIC2, BMP4, BMP1, C4BPB, CCND2, CYP24A1, DMD, DPYSL2, DSP, CPT1A, CSF1, CST4, CYP2J2, FGF13, LPAR1, S1PR3, EFNB2, EPHA7, EPHA3, EYA4, F2RL1, F3, F2RL2, F13A1, ACSL1, EFEMP1, FBP1, GPR1, GRB14, HAS2, NCKAP1L, HLA-A, HLA-B, GATA6, GBP1, GBP2, GJA1, GNGT2, IGFBP4, IL1B, IL13RA2, INHBB, ITGB3, CD82, HLA-DRA, FOXA1, HES1, MEF2C, MEIS3P1, MAP3K1, MMP3, MMP1, KISS1, LAMA3, LAMA4, LCP1, LTBP1, SERPINB2, NEO1, PKIA, PDE3B, PIK3CG, PLAU, PLCB4, PLXNA2, PLXNA1, ROBO1, RRBP1, SCN5A, SCP2, SCN9A, CCL2, PTPRS, RAB27B, RGS10, SOX4, SSX1, TCF4, SDC2, SNAI2, TUFT1, VEGFA, WT1, TGFA, TGFB2, THBS1, TGFBR3, TGM2, TIMP3, TLR3, HIST2H4B, SRPX, ZNF43, EVI5, CXCR4, NRP2, CLIC3, GPRC5A, SORBS2, GPR65, CDC42BPA, CST7, LGR5, CASK, SERPINB7, TNFSF10, CRADD, GMFG, PTGES, FCGR2C, PDLIM1, IL1RL1, NOG, LIPG, TSPAN2, RAD50, OPTN, MBNL2, CTDSPL, RAPGEF5, NUAK1, PLK2, PDE10A, MYL9, CNKSR2, CARD8, IL24, ESM1, IRAK3, SP140, ARL2BP, PLCB1, ADGRL3, TBC1D30, PTPN22, PITPNC1, TIAM2, FLRT2, TNFRSF21, PDCD4, PCDH17, PCDH11X, OR2H1, TRIB2, STXBP6, CDON, KCNIP3, G0S2, ASAP1, BCL11A, SHC3, TENM3, MCTP2, LMO3, BAIAP2L1, DOCK10, HRASLS, SLC44A2, ZMIZ1, CEMIP, MTUS1, ZNF608, SORCS2, PMEPA1, PRDM9, PRDM8, SAMSN1, NXN, HHIP, SLAMF7, GPR135, TNS3, PORCN, CDK15, CPEB4, FAT4, COLGALT1, ADAMTS12, PARD6B, AFAP1L2, ANKRD30A, ARRDC4, TSPYL5, NAV3, EFHC1, SLITRK1, FBXO32, SYTL3, OR2M3, TRIM6, PIK3AP1, OR5P2, FOXR2, LYPD6B, FAM172BP, PRICKLE1, SH3RF2, NRK, PLPP4, OR5B21, ZNF438, FAM83B, ADGRF1, SSX8, LAMA1, HMSD, ZNF391, SHISA2, SAMD5, ZNF716, MIR181B1, MAFIP |  |
| Regulation Of Biological Process | 237 | 1 | 1.18E-05 | 0.015 | | ARHGDIB, ARRB1, ATP2B4, BCL2A1, AADAC, ABCA1, CD22, CD33, ADGRE5, CHRM3, CLU, CLIC2, BMP4, BMP1, C4BPB, CCND2, CYP24A1, DMD, DPYSL2, DSP, CPT1A, CSF1, CST4, CYP2J2, FGF13, LPAR1, S1PR3, EFNB2, STOM, EPHA7, EPHA3, EYA4, F2RL1, F3, F2RL2, F13A1, ACSL1, EFEMP1, FBP1, GPR1, GRB14, HAS2, NCKAP1L, HLA-A, HLA-B, GATA6, GBP1, GBP2, GJA1, GNGT2, IGFBP4, IL1B, IL13RA2, INHBB, ITGB3, CD82, HLA-DRA, HMGCS1, FOXA1, HES1, MEF2C, MEIS3P1, MAP3K1, MMP3, MMP1, KCNQ3, KISS1, LAMA3, LAMA4, LCP1, LTBP1, SERPINB2, NEO1, PKIA, PDE3B, PIK3CG, PLAU, PLCB4, PLXNA2, PLTP, PLXNA1, ROBO1, RRBP1, SCN5A, SCP2, SCN9A, CCL2, PTPRS, RAB27B, RGS10, SOX4, SSX1, TBXAS1, TCF4, SDC2, SNAI2, TUFT1, VEGFA, WT1, TGFA, TGFB2, THBS1, TGFBR3, TGM2, TIMP3, TLR3, HIST2H4B, SRPX, ZNF43, EVI5, CXCR4, NRP2, CLIC3, GPRC5A, SORBS2, GPR65, CDC42BPA, CST7, LGR5, CASK, SERPINB7, TNFSF10, CRADD, GMFG, PTGES, CYTIP, FCGR2C, PDLIM1, IL1RL1, NOG, LIPG, TSPAN2, RAD50, OPTN, MBNL2, CTDSPL, RAPGEF5, NUAK1, CLEC2B, PLK2, PDE10A, MYL9, CNKSR2, CARD8, IL24, ESM1, IRAK3, SP140, TDRD7, ARL2BP, PLCB1, ADGRL3, TBC1D30, PTPN22, PITPNC1, TIAM2, FLRT2, ABI3BP, TNFRSF21, PDCD4, PCDH17, PCDH11X, OR2H1, TRIB2, STXBP6, CDON, KCNIP3, G0S2, ASAP1, BCL11A, SHC3, CLIC6, TENM3, MCTP2, LMO3, BAIAP2L1, DOCK10, HRASLS, SLC44A2, ZMIZ1, CEMIP, HEG1, MTUS1, ZNF608, SORCS2, PMEPA1, PRDM9, PRDM8, SAMSN1, NXN, HHIP, SLAMF7, GPR135, TNS3, PORCN, CDK15, CPEB4, FAT4, COLGALT1, ADAMTS12, PARD6B, AFAP1L2, ANKRD30A, ARRDC4, TSPYL5, NAV3, EFHC1, SLITRK1, FBXO32, SYTL3, OR2M3, TRIM6, PIK3AP1, OR5P2, FOXR2, LYPD6B, FAM172BP, PRICKLE1, SH3RF2, NRK, SAMD9L, PLPP4, OR5B21, ZNF438, FAM83B, ADGRF1, SSX8, LAMA1, HMSD, ZNF391, SHISA2, SAMD5, ZNF716, MIR181B1, UCA1, MIR604, MAFIP |  |
| Regulation Of Glomerular Mesangial Cell Proliferation | 4 | 33 | 1.21E-05 | 0.015 | | ITGB3, WT1, BMP4, SERPINB7 |  |
| Regulation Of Stress-Activated Mapk Cascade | 14 | 5 | 1.26E-05 | 0.011 | | PDCD4, MAP3K1, NRK, MIR181B1, KISS1, PLCB1, PTPN22, IL1B, VEGFA, TGFB2, TGFBR3, SAMD5, TLR3, F2RL1 |  |
| Regulation Of Stress-Activated Protein Kinase Signaling Cascade | 14 | 5 | 1.27E-05 | 0.023 | | PDCD4, MAP3K1, NRK, MIR181B1, KISS1, PLCB1, PTPN22, IL1B, VEGFA, TGFB2, TGFBR3, SAMD5, TLR3, F2RL1 |  |
| Response To Interleukin-1 | 11 | 6 | 1.33E-05 | 0.023 | | ADAMTS12, MMP3, MMP1, HAS2, GBP1, GBP2, GBP3, INHBB, CCL2, HES1, IRAK3 |  |
| Positive Regulation Of Cellular Process | 118 | 1 | 1.35E-05 | 0.021 | | ARRB1, ATP2B4, ARRDC4, AADAC, TSPYL5, NAV3, EFHC1, ADGRE5, SLITRK1, FBXO32, CLU, CLIC2, BMP4, CCND2, DMD, DPYSL2, TRIM6, PIK3AP1, CPT1A, CSF1, CYP2J2, FGF13, LPAR1, S1PR3, EFNB2, EPHA7, EPHA3, EYA4, F2RL1, F3, F2RL2, ACSL1, EFEMP1, GRB14, HAS2, NCKAP1L, HLA-A, GATA6, PRICKLE1, GJA1, IGFBP4, IL1B, INHBB, ITGB3, FOXA1, HES1, MEF2C, MAP3K1, MMP3, MMP1, NRK, KISS1, LCP1, ZNF438, NEO1, PIK3CG, PLAU, PLXNA2, PLXNA1, ROBO1, SCN5A, SCP2, CCL2, RAB27B, SOX4, TCF4, SNAI2, VEGFA, WT1, TGFA, TGFB2, THBS1, TGFBR3, TGM2, TIMP3, TLR3, SRPX, CXCR4, NRP2, GPR65, LGR5, CASK, SAMD5, SERPINB7, TNFSF10, CRADD, PDLIM1, IL1RL1, NOG, RAD50, OPTN, PLK2, MIR181B1, CARD8, IL24, ESM1, ARL2BP, PLCB1, ADGRL3, PTPN22, TIAM2, FLRT2, PDCD4, TRIB2, CDON, G0S2, ASAP1, BCL11A, TENM3, LMO3, BAIAP2L1, SLC44A2, ZMIZ1, CEMIP, PRDM9, TNS3, COLGALT1, AFAP1L2 |  |
| Positive Regulation Of Cell Migration | 22 | 3 | 1.37E-05 | 0.018 | | PLK2, HAS2, NCKAP1L, MIR181B1, CXCR4, PLAU, NRP2, IL1B, ITGB3, CCL2, BMP4, CEMIP, MMP3, MMP1, CSF1, SNAI2, VEGFA, LPAR1, TGFB2, THBS1, F2RL1, F3 |  |
| Negative Regulation Of Protein Modification Process | 24 | 3 | 1.43E-05 | 0.023 | | PDCD4, ARRB1, PKIA, ATP2B4, NCKAP1L, GBP1, IL1B, LMO3, TRIB2, GPRC5A, BMP4, SH3RF2, IRAK3, SOX4, GMFG, DMD, NOG, PMEPA1, PTPN22, SAMSN1, CTDSPL, NXN, TIMP3, F2RL1 |  |
| Positive Regulation Of Ras Protein Signal Transduction | 7 | 11 | 1.44E-05 | 0.023 | | ROBO1, ARRB1, LPAR1, GPR65, CSF1, F2RL1, F2RL2 |  |
| Negative Regulation Of Neurogenesis | 16 | 4 | 1.70E-05 | 0.017 | | PLK2, HLA-A, NOG, ASAP1, FGF13, BCL11A, IL1B, LPAR1, BMP4, EFNB2, PTPRS, EPHA7, EPHA3, HES1, NEO1, EFEMP1 |  |
| Negative Regulation Of Collateral Sprouting | 4 | 30 | 1.72E-05 | 0.023 | | FGF13, BCL11A, PTPRS, EPHA7 |  |
| Epithelial Tube Branching Involved In Lung Morphogenesis | 5 | 19 | 1.80E-05 | 0.011 | | HHIP, LAMA1, BMP4, FOXA1, CTSD |  |
| Positive Regulation Of Protein Serine/Threonine Kinase Activity | 17 | 4 | 1.85E-05 | 0.013 | | ARRB1, CEMIP, MAP3K1, ATP2B4, NRK, CXCR4, PIK3CG, ROBO1, IL1B, VEGFA, LPAR1, BMP4, TGFA, THBS1, SAMD5, CCND2, ACSL1 |  |
| Regulation Of Kidney Development | 7 | 10 | 1.88E-05 | 0.023 | | VEGFA, ITGB3, WT1, FAT4, BMP4, NOG, SERPINB7 |  |
| Negative Regulation Of Neuron Differentiation | 14 | 5 | 1.88E-05 | 0.017 | | PLK2, HLA-A, ASAP1, FGF13, BCL11A, IL1B, LPAR1, EFNB2, PTPRS, EPHA7, EPHA3, HES1, NEO1, EFEMP1 |  |
| Enzyme Linked Receptor Protein Signaling Pathway | 25 | 3 | 1.91E-05 | 0.022 | | PDCD4, GRB14, NCKAP1L, NRP2, INHBB, ITGB3, FAT4, BMP4, MAP3K1, SDC2, NOG, CSF1, PLCB1, LTBP1, SHC3, VEGFA, FLRT2, EFNB2, TGFA, TGFB2, TGFBR3, EPHA7, EPHA3, EFEMP1, FAM83B |  |
| Homophilic Cell Adhesion Via Plasma Membrane Adhesion Molecules | 11 | 6 | 1.96E-05 | 0.023 | | PCDH17, PCDH11X, PCDH11Y, PCDHB2, TENM3, ROBO1, CDH11, PCDHB5, PCDHB6, FAT4, PCDHB16 |  |
| Regulation Of Map Kinase Activity | 17 | 4 | 1.98E-05 | 0.021 | | PDCD4, ARRB1, MAP3K1, NRK, CXCR4, PIK3CG, ROBO1, PTPN22, IL1B, VEGFA, TRIB2, LPAR1, BMP4, TGFA, THBS1, SAMD5, IRAK3 |  |
| Negative Regulation Of Biological Process | 122 | 1 | 2.01E-05 | 0.023 | | ARHGDIB, ARRB1, ATP2B4, BCL2A1, ABCA1, NAV3, CD33, FBXO32, CLU, CLIC2, BMP4, C4BPB, CCND2, DMD, TRIM6, PIK3AP1, CST4, CYP2J2, FGF13, LPAR1, S1PR3, EFNB2, EPHA7, EPHA3, EYA4, FAM172BP, F2RL1, EFEMP1, FBP1, GRB14, NCKAP1L, HLA-A, GATA6, GBP1, PRICKLE1, GJA1, IGFBP4, IL1B, IL13RA2, INHBB, ITGB3, FOXA1, HES1, SH3RF2, MEF2C, MAP3K1, MMP3, NRK, KISS1, LTBP1, SERPINB2, ZNF438, NEO1, PKIA, PDE3B, PIK3CG, PLAU, PLCB4, PLXNA2, PLXNA1, ROBO1, SCP2, CCL2, HMSD, PTPRS, RGS10, SOX4, SSX1, TCF4, SNAI2, VEGFA, WT1, TGFA, TGFB2, THBS1, TGFBR3, TIMP3, TLR3, SHISA2, HIST2H4B, SRPX, GPRC5A, CST7, CASK, SERPINB7, TNFSF10, CRADD, GMFG, PTGES, IL1RL1, NOG, RAD50, OPTN, CTDSPL, PLK2, PDE10A, MIR181B1, CARD8, IL24, IRAK3, PLCB1, TBC1D30, PTPN22, TNFRSF21, PDCD4, PCDH17, PCDH11X, TRIB2, STXBP6, MIR604, KCNIP3, ASAP1, BCL11A, LMO3, ZNF608, PMEPA1, PRDM8, SAMSN1, NXN, HHIP, CPEB4, ADAMTS12 |  |
| Coronary Vasculature Development | 6 | 13 | 2.01E-05 | 0.017 | | ROBO1, WT1, BMP4, TGFBR3, PRICKLE1, LTBP1 |  |
| Anatomical Structure Formation Involved In Morphogenesis | 27 | 3 | 2.10E-05 | 0.015 | | PDE3B, GATA6, PRICKLE1, PIK3CG, PLAU, PLXNA2, ROBO1, NRP2, ITGB3, CCL2, BMP4, FOXA1, ESM1, HES1, SOX4, CDON, MEF2C, NOG, VEGFA, WT1, EFNB2, TGFA, TGFB2, THBS1, TGFBR3, TGM2, NEO1 |  |
| Negative Regulation Of Protein Phosphorylation | 19 | 3 | 2.18E-05 | 0.023 | | PDCD4, ARRB1, PKIA, GBP1, IL1B, LMO3, TRIB2, GPRC5A, BMP4, IRAK3, GMFG, DMD, NOG, PMEPA1, PTPN22, SAMSN1, CTDSPL, TIMP3, F2RL1 |  |
| Regulation Of Vasculature Development | 19 | 3 | 2.50E-05 | 0.023 | | PDCD4, PLK2, MIR181B1, PDE3B, GATA6, CXCR4, IL1B, ITGB3, BMP4, SERPINB7, TCF4, VEGFA, WT1, HHIP, EFNB2, TGFB2, THBS1, TLR3, F3 |  |
| Positive Regulation Of Cell Differentiation | 34 | 2 | 2.50E-05 | 0.023 | | HAS2, NCKAP1L, MIR181B1, GATA6, GJA1, CXCR4, PLXNA2, PLXNA1, TENM3, ROBO1, SLITRK1, IL1B, LMO3, ITGB3, CLU, BMP4, FOXA1, CASK, HES1, CDON, ZMIZ1, MEF2C, DMD, TCF4, NOG, CSF1, PLCB1, SNAI2, BCL11A, VEGFA, LPAR1, EFNB2, TGFB2, EPHA3 |  |
| Regulation Of Metal Ion Transport | 19 | 3 | 2.61E-05 | 0.022 | | ATP2B4, GJA1, CXCR4, PIK3CG, PLCB4, ITGB3, CLIC2, SCN5A, CCL2, BMP4, CASK, HES1, CEMIP, DMD, PLCB1, KCNIP3, PTPN22, TGFB2, STOM |  |
| Negative Regulation Of Phosphorylation | 20 | 3 | 2.71E-05 | 0.023 | | PDCD4, ARRB1, PKIA, GBP1, IL1B, LMO3, TRIB2, GPRC5A, BMP4, IRAK3, GMFG, DMD, NOG, PMEPA1, PTPN22, SAMSN1, CTDSPL, TIMP3, F2RL1, FBP1 |  |
| Positive Regulation Of Cell Motility | 22 | 3 | 2.78E-05 | 0.023 | | PLK2, HAS2, NCKAP1L, MIR181B1, CXCR4, PLAU, NRP2, IL1B, ITGB3, CCL2, BMP4, CEMIP, MMP3, MMP1, CSF1, SNAI2, VEGFA, LPAR1, TGFB2, THBS1, F2RL1, F3 |  |
| Phosphate-Containing Compound Metabolic Process | 53 | 2 | 2.78E-05 | 0.023 | | LPCAT2, PLK2, PDE10A, PDE3B, PIK3CG, AK4, PLAU, PLCB4, ENTPD3, SCP2, CCL2, LAMA1, BMP4, IL24, PTPRS, CPPED1, IRAK3, SLC44A2, PIK3AP1, PLCB1, FGF13, PTPN22, EFNB2, TGFB2, TGFBR3, EPHA7, EPHA3, EYA4, TLR3, ACSL1, SERINC2, EFEMP1, FBP1, GATA6, CDK15, ELOVL7, IL1B, TRIB2, CDC42BPA, HMGCS1, CASK, HDDC2, GMFG, MEF2C, MAP3K1, NRK, LIPG, PLPP4, RAD50, SHC3, CTDSPL, SLC37A2, NUAK1 |  |
| Regulation Of Ion Transmembrane Transport | 20 | 3 | 3.05E-05 | 0.020 | | ATP2B4, GJA1, PIK3CG, CLIC3, ITGB3, CLIC2, SCN5A, SCN9A, BMP4, MEF2C, CEMIP, DMD, KCNQ3, PLCB1, KCNIP3, PTPN22, CLIC6, TGFB2, THBS1, STOM |  |
| Positive Regulation Of Kidney Development | 6 | 12 | 3.08E-05 | 0.023 | | VEGFA, ITGB3, WT1, BMP4, NOG, SERPINB7 |  |
| Ventricular Septum Morphogenesis | 6 | 12 | 3.09E-05 | 0.015 | | SOX4, ROBO1, TGFB2, TGFBR3, NOG, HES1 |  |
| Trabecula Morphogenesis | 5 | 17 | 3.09E-05 | 0.015 | | VEGFA, HEG1, TGFB2, TGFBR3, NOG |  |
| Morphogenesis Of A Branching Epithelium | 11 | 5 | 3.24E-05 | 0.013 | | CSF1, CXCR4, CTSD, VEGFA, WT1, HHIP, LAMA1, FAT4, BMP4, FOXA1, TGM2 |  |
| Response To Lipid | 37 | 2 | 3.30E-05 | 0.020 | | ABCA1, PLAU, FBXO32, SCP2, CCL2, BMP4, IL24, IRAK3, CCND2, CYP24A1, TBXAS1, TRIM6, CPT1A, PLCB1, PTPN22, VEGFA, LPAR1, TGFB2, THBS1, TGFBR3, EPHA3, F3, ACSL1, PDCD4, GBP2, GJA1, IL1B, INHBB, HMGCS1, FOXA1, HES1, PTGES, MEF2C, MMP3, MMP1, LTBP1, GDAP1 |  |
| Regulation Of Cellular Component Biogenesis | 31 | 2 | 3.36E-05 | 0.021 | | PLK2, HAS2, NCKAP1L, ABCA1, EVI5, GJA1, NAV3, ADGRE5, SLITRK1, BAIAP2L1, CLU, STXBP6, GPR65, GMFG, MEF2C, MAP3K1, MMP3, MMP1, LCP1, PMEPA1, ADGRL3, SNAI2, TBC1D30, ASAP1, VEGFA, LPAR1, FLRT2, THBS1, EPHA7, EPHA3, F2RL1 |  |
| Cell Communication | 31 | 2 | 3.47E-05 | 0.022 | | PCDH17, PCDH11X, GJA1, CD33, ADGRE5, IL1B, INHBB, CHRM3, CPEB4, SCN5A, CCL2, PCDHB6, BMP4, FOXA1, HES1, TNFSF10, PCDH11Y, MMP3, MMP1, PCDHB2, KCNQ3, LTBP1, FGF13, BCL11A, SHC3, PCDHB5, OPTN, GDAP1, EFNB2, TGFB2, PCDHB16 |  |
| Positive Regulation Of Cell Development | 23 | 3 | 3.59E-05 | 0.022 | | HAS2, CXCR4, PLXNA2, PLXNA1, TENM3, ROBO1, SLITRK1, IL1B, ITGB3, BMP4, FOXA1, CASK, HES1, CDON, MEF2C, DMD, TCF4, NOG, CSF1, BCL11A, VEGFA, LPAR1, EPHA3 |  |
| Regulation Of Secretion By Cell | 27 | 3 | 3.93E-05 | 0.022 | | TNFRSF21, ARRB1, PDE3B, GBP1, GJA1, MCTP2, IL1B, IL13RA2, CARD8, INHBB, STXBP6, CASK, RAB27B, SYTL3, SOX4, MEF2C, ARL2BP, DPYSL2, TRIM6, IL1RL1, KISS1, CPT1A, CYP2J2, PTPN22, TGFB2, F2RL1, NEO1 |  |
| Cell-Cell Adhesion Via Plasma-Membrane Adhesion Molecules | 13 | 4 | 3.94E-05 | 0.022 | | PCDH17, PCDH11X, PCDH11Y, PCDHB2, ADGRL3, TENM3, ROBO1, CDH11, PCDHB5, PCDHB6, FAT4, TGFB2, PCDHB16 |  |
| Regulation Of Epithelial To Mesenchymal Transition | 8 | 8 | 3.94E-05 | 0.021 | | NOG, IL1B, VEGFA, BMP4, FOXA1, TGFB2, TGFBR3, EPHA3 |  |
| Positive Regulation Of Stress-Activated Protein Kinase Signaling Cascade | 11 | 5 | 4.14E-05 | 0.018 | | MAP3K1, NRK, MIR181B1, KISS1, PLCB1, IL1B, VEGFA, TGFB2, SAMD5, TLR3, F2RL1 |  |
| Negative Regulation Of Cellular Metabolic Process | 66 | 2 | 4.21E-05 | 0.020 | | ARRB1, ATP2B4, BCL2A1, MIR181B1, CARD8, CLU, BMP4, IL24, C4BPB, IRAK3, DMD, TRIM6, PLCB1, CST4, PTPN22, FAM172BP, F2RL1, FBP1, PDCD4, PCDH11X, NCKAP1L, GATA6, GBP1, PRICKLE1, GJA1, IL1B, INHBB, TRIB2, ITGB3, FOXA1, HES1, SH3RF2, MEF2C, MMP3, KCNIP3, BCL11A, SERPINB2, ZNF438, PKIA, PIK3CG, LMO3, HMSD, SOX4, SSX1, ZNF608, TCF4, PMEPA1, PRDM8, SNAI2, SAMSN1, VEGFA, WT1, NXN, TGFB2, THBS1, TIMP3, HIST2H4B, CPEB4, GPRC5A, CST7, SERPINB7, GMFG, NOG, RAD50, OPTN, CTDSPL |  |
| Phosphorus Metabolic Process | 53 | 2 | 4.21E-05 | 0.019 | | LPCAT2, PLK2, PDE10A, PDE3B, PIK3CG, AK4, PLAU, PLCB4, ENTPD3, SCP2, CCL2, LAMA1, BMP4, IL24, PTPRS, CPPED1, IRAK3, SLC44A2, PIK3AP1, PLCB1, FGF13, PTPN22, EFNB2, TGFB2, TGFBR3, EPHA7, EPHA3, EYA4, TLR3, ACSL1, SERINC2, EFEMP1, FBP1, GATA6, CDK15, ELOVL7, IL1B, TRIB2, CDC42BPA, HMGCS1, CASK, HDDC2, GMFG, MEF2C, MAP3K1, NRK, LIPG, PLPP4, RAD50, SHC3, CTDSPL, SLC37A2, NUAK1 |  |
| Positive Regulation Of Neurogenesis | 21 | 3 | 4.26E-05 | 0.020 | | CXCR4, PLXNA2, PLXNA1, TENM3, ROBO1, SLITRK1, IL1B, BMP4, FOXA1, CASK, HES1, CDON, MEF2C, DMD, TCF4, NOG, CSF1, BCL11A, VEGFA, LPAR1, EPHA3 |  |
| Positive Regulation Of Cellular Component Movement | 22 | 3 | 4.30E-05 | 0.022 | | PLK2, HAS2, NCKAP1L, MIR181B1, CXCR4, PLAU, NRP2, IL1B, ITGB3, CCL2, BMP4, CEMIP, MMP3, MMP1, CSF1, SNAI2, VEGFA, LPAR1, TGFB2, THBS1, F2RL1, F3 |  |
| Regulation Of Cell Proliferation Involved In Kidney Development | 4 | 25 | 4.44E-05 | 0.022 | | ITGB3, WT1, BMP4, SERPINB7 |  |
| Homeostatic Process | 44 | 2 | 4.44E-05 | 0.011 | | HECTD4, ATP2B4, BCL2A1, PDE3B, ABCA1, PIK3CG, PLAU, EFHC1, SLITRK1, SCN5A, CCL2, BMP4, ACOXL, DOCK10, SOX4, CEMIP, DMD, TBXAS1, CSF1, CST4, FLG, VEGFA, NXN, LPAR1, S1PR3, TGM2, EPHA3, F2RL1, F2RL2, ILDR2, HIST2H4B, NCKAP1L, GJA1, CXCR4, IL1B, ITGB3, GPR65, FOXA1, MEF2C, SNX10, KISS1, LIPG, RAD50, NEO1 |  |
| Response To Tumor Necrosis Factor | 12 | 5 | 4.46E-05 | 0.021 | | TNFRSF21, ADAMTS12, MMP3, MMP1, HAS2, GBP1, GBP2, GBP3, OPTN, CCL2, THBS1, HES1 |  |
| Outflow Tract Septum Morphogenesis | 5 | 15 | 4.58E-05 | 0.021 | | ROBO1, NRP2, BMP4, TGFB2, GATA6 |  |
| Branching Morphogenesis Of An Epithelial Tube | 10 | 6 | 5.33E-05 | 0.013 | | CSF1, CXCR4, CTSD, VEGFA, WT1, HHIP, LAMA1, FAT4, BMP4, FOXA1 |  |
| Response To Vitamin D | 6 | 11 | 5.51E-05 | 0.019 | | CYP24A1, IL1B, MMP1, GDAP1, TGFB2, LTBP1 |  |
| Secretion | 32 | 2 | 5.54E-05 | 0.015 | | ILDR2, NCKAP1L, ABCA1, GJA1, PIK3CG, PLAU, CD33, CD68, ADGRE5, CHRM3, ITGB3, CLU, STXBP6, CCL2, CPPED1, CASK, RAB27B, SYTL3, SLC44A2, GMFG, CRISPLD2, DSP, SNX10, CTSD, OPTN, VEGFA, TGFB2, THBS1, TIMP3, STOM, F2RL1, F13A1 |  |
| Cellular Response To Tumor Necrosis Factor | 11 | 5 | 5.56E-05 | 0.021 | | TNFRSF21, ADAMTS12, MMP1, HAS2, GBP1, GBP2, GBP3, OPTN, CCL2, THBS1, HES1 |  |
| Cellular Response To Cholesterol | 4 | 23 | 5.57E-05 | 0.020 | | INHBB, SCP2, ABCA1, HMGCS1 |  |
| Response To Acid Chemical | 22 | 3 | 5.74E-05 | 0.011 | | ATP2B4, ABCA1, GJA1, IL1B, CPEB4, BMP4, HES1, PTGES, MEF2C, MMP3, TBXAS1, CPT1A, PLCB1, BCL11A, OPTN, VEGFA, GDAP1, TGFB2, TGFBR3, TIMP3, EPHA3, ACSL1 |  |
| Secretion By Cell | 29 | 2 | 5.81E-05 | 0.022 | | ILDR2, NCKAP1L, ABCA1, PIK3CG, PLAU, CD33, CD68, ADGRE5, ITGB3, CLU, STXBP6, CCL2, CPPED1, CASK, RAB27B, SYTL3, SLC44A2, GMFG, CRISPLD2, DSP, CTSD, OPTN, VEGFA, TGFB2, THBS1, TIMP3, STOM, F2RL1, F13A1 |  |
| Positive Regulation Of Cytosolic Calcium Ion Concentration Involved In Phospholipase C-Activating G-Protein Coupled Signaling Pathway | 6 | 11 | 6.17E-05 | 0.021 | | LPAR1, GPR65, KISS1, TGM2, F2RL1, F2RL2 |  |
| Positive Regulation Of Locomotion | 22 | 3 | 6.18E-05 | 0.015 | | PLK2, HAS2, NCKAP1L, MIR181B1, CXCR4, PLAU, NRP2, IL1B, ITGB3, CCL2, BMP4, CEMIP, MMP3, MMP1, CSF1, SNAI2, VEGFA, LPAR1, TGFB2, THBS1, F2RL1, F3 |  |
| Negative Regulation Of Epithelial Cell Proliferation | 11 | 5 | 6.34E-05 | 0.022 | | MEF2C, GJA1, SNAI2, ROBO1, CCL2, BMP4, EFNB2, TGFB2, THBS1, TGFBR3, CASK |  |
| Response To Drug | 34 | 2 | 6.38E-05 | 0.020 | | ARRB1, ABCA1, AK4, FBXO32, PTGR1, BMP4, RGS10, CCND2, DPYSL2, TBXAS1, SDC2, CPT1A, ADGRL3, PTPN22, VEGFA, TGFA, TGFB2, THBS1, TIMP3, TLR3, F3, ACSL1, FBP1, NCKAP1L, GATA6, CXCR4, IL1B, ITGB3, HMGCS1, MEF2C, MMP3, MMP1, BCL11A, OPTN |  |
| Activation Of Protein Kinase Activity | 15 | 4 | 6.62E-05 | 0.021 | | ARRB1, MAP3K1, NRK, NCKAP1L, CXCR4, FGF13, IL1B, VEGFA, ITGB3, LPAR1, BMP4, TGFA, TGFB2, THBS1, TLR3 |  |
| Cellular Response To Growth Factor Stimulus | 16 | 4 | 6.76E-05 | 0.021 | | ADAMTS12, MEF2C, HAS2, GATA6, NOG, PLAU, SNAI2, LTBP1, OPTN, VEGFA, ITGB3, CLU, CCL2, BMP4, THBS1, HES1 |  |
| Exocytosis | 24 | 3 | 6.79E-05 | 0.021 | | NCKAP1L, PIK3CG, PLAU, CD33, CD68, ADGRE5, ITGB3, CLU, STXBP6, CCL2, CPPED1, RAB27B, SYTL3, SLC44A2, GMFG, CRISPLD2, DSP, CTSD, VEGFA, TGFB2, THBS1, TIMP3, STOM, F13A1 |  |
| Positive Regulation Of Cell Adhesion | 18 | 3 | 6.88E-05 | 0.022 | | HAS2, NCKAP1L, HLA-A, IL1B, ITGB3, CCL2, FOXA1, HES1, ZMIZ1, DMD, CSF1, PTPN22, VEGFA, EFNB2, TGFB2, THBS1, TGM2, ABI3BP |  |
| Regulation Of Cardioblast Differentiation | 3 | 42 | 6.97E-05 | 0.022 | | TGFB2, GATA6, PRICKLE1 |  |
| Negative Regulation Of Cell Proliferation | 27 | 2 | 7.24E-05 | 0.008 | | TNFRSF21, PDCD4, SRPX, GJA1, CD33, ROBO1, IL1B, SCP2, CCL2, BMP4, IL24, CASK, HES1, SOX4, PTGES, MEF2C, NRK, IL1RL1, KISS1, NOG, SNAI2, OPTN, WT1, EFNB2, TGFB2, THBS1, TGFBR3 |  |
| Chronic Inflammatory Response | 4 | 22 | 7.28E-05 | 0.021 | | PTGES, IL1B, THBS1, GJA1 |  |
| Regulation Of Response To Stress | 41 | 2 | 7.31E-05 | 0.011 | | PLK2, ATP2B4, MIR181B1, PIK3CG, PLAU, CLU, PTPRS, C4BPB, IRAK3, TRIM6, PIK3AP1, PLCB1, SNAI2, PTPN22, VEGFA, TGFB2, THBS1, TGFBR3, TGM2, EYA4, TLR3, F2RL1, F3, PDCD4, HLA-A, GJA1, CXCR4, IL1B, CASK, SAMD5, ADAMTS12, MEF2C, MAP3K1, MMP3, NRK, IL1RL1, KISS1, OPTN, SERPINB2, NUAK1, NEO1 |  |
| Regulation Of Hydrolase Activity | 38 | 2 | 7.52E-05 | 0.020 | | ARHGDIB, ARRB1, BCL2A1, PLXNA2, PLXNA1, ROBO1, CARD8, CCL2, HMSD, IL24, DOCK10, RGS10, PLCB1, TBC1D30, TIAM2, VEGFA, LPAR1, TGFB2, THBS1, TIMP3, EPHA7, EPHA3, F2RL1, F3, PCDH11X, NCKAP1L, EVI5, TOR1AIP2, GPR65, CST7, SH3RF2, SERPINB7, TNFSF10, CRADD, MEF2C, ASAP1, SERPINB2, NUAK1 |  |
| Regulation Of Developmental Growth | 17 | 3 | 7.67E-05 | 0.021 | | MEF2C, DPYSL2, NRK, GATA6, NOG, GJA1, CSF1, PLCB1, CXCR4, FGF13, BCL11A, VEGFA, WT1, BMP4, PTPRS, TGFBR3, EPHA7 |  |
| Synapse Assembly | 7 | 8 | 7.80E-05 | 0.021 | | PCDHB5, SLITRK1, PCDHB6, PCDHB2, PCDHB16, ADGRL3, ADGRF1 |  |
| Positive Regulation Of Response To External Stimulus | 15 | 4 | 8.07E-05 | 0.016 | | PDCD4, NCKAP1L, IL1RL1, CSF1, PIK3CG, SNAI2, OPTN, IL1B, VEGFA, LPAR1, THBS1, TGM2, TLR3, F2RL1, F3 |  |
| Negative Regulation Of Epithelial To Mesenchymal Transition | 5 | 14 | 8.15E-05 | 0.021 | | VEGFA, FOXA1, TGFB2, TGFBR3, NOG |  |
| Regulation Of Ras Protein Signal Transduction | 12 | 4 | 8.32E-05 | 0.013 | | ARHGDIB, ARRB1, ABCA1, CSF1, ROBO1, TIAM2, LPAR1, GPR65, TGFB2, F2RL1, F2RL2, FBP1 |  |
| Calcium Ion Homeostasis | 19 | 3 | 8.45E-05 | 0.020 | | ATP2B4, GJA1, CXCR4, PIK3CG, EFHC1, IL1B, ITGB3, SCN5A, CCL2, GPR65, BMP4, CEMIP, SNX10, KISS1, LPAR1, S1PR3, TGM2, F2RL1, F2RL2 |  |
| Regulation Of Peptide Secretion | 20 | 3 | 8.69E-05 | 0.021 | | TNFRSF21, ARRB1, PDE3B, GBP1, GJA1, IL1B, CARD8, INHBB, CASK, SOX4, ARL2BP, TRIM6, IL1RL1, KISS1, CPT1A, CYP2J2, PTPN22, TGFB2, F2RL1, NEO1 |  |
| Macrophage Differentiation | 4 | 21 | 8.81E-05 | 0.021 | | VEGFA, BMP4, CSF1, PLCB1 |  |
| Response To Vitamin | 10 | 5 | 9.17E-05 | 0.011 | | CYP24A1, MEF2C, MMP1, LTBP1, IL1B, VEGFA, GDAP1, HMGCS1, TGFB2, TIMP3 |  |
| Regulation Of Protein Secretion | 19 | 3 | 9.52E-05 | 0.019 | | TNFRSF21, ARRB1, PDE3B, GBP1, GJA1, IL1B, CARD8, INHBB, CASK, SOX4, ARL2BP, TRIM6, IL1RL1, CPT1A, CYP2J2, PTPN22, TGFB2, F2RL1, NEO1 |  |
| Regulation Of Cytokine Production Involved In Immune Response | 7 | 8 | 9.80E-05 | 0.021 | | IL1B, HLA-A, TRIM6, TGFB2, TLR3, F2RL1, IRAK3 |  |
| Regulated Exocytosis | 22 | 3 | 1.02E-04 | 0.016 | | NCKAP1L, PIK3CG, PLAU, CD33, CD68, ADGRE5, ITGB3, CLU, CPPED1, RAB27B, SYTL3, SLC44A2, GMFG, CRISPLD2, DSP, CTSD, VEGFA, TGFB2, THBS1, TIMP3, STOM, F13A1 |  |
| Response To Nutrient | 15 | 4 | 1.06E-04 | 0.021 | | CYP24A1, MEF2C, MMP1, ABCA1, LIPG, LTBP1, BCL11A, OPTN, IL1B, VEGFA, GDAP1, HMGCS1, TGFB2, TIMP3, ACSL1 |  |
| Regulation Of Cell Morphogenesis Involved In Differentiation | 15 | 4 | 1.07E-04 | 0.021 | | DPYSL2, HAS2, SDC2, GBP1, PLXNA2, PLXNA1, ROBO1, FGF13, BCL11A, SLITRK1, VEGFA, ITGB3, PTPRS, CASK, EPHA7 |  |
| Hyaluronan Biosynthetic Process | 3 | 37 | 1.07E-04 | 0.021 | | IL1B, CEMIP, HAS2 |  |
| Regulation Of Cell-Cell Adhesion | 17 | 3 | 1.15E-04 | 0.008 | | TNFRSF21, ZMIZ1, HAS2, ZNF608, NCKAP1L, HLA-A, SNAI2, TENM3, PTPN22, IL1B, VEGFA, CCL2, BMP4, EFNB2, FOXA1, EPHA7, HES1 |  |
| Positive Regulation Of Bmp Signaling Pathway | 5 | 13 | 1.23E-04 | 0.021 | | BMP4, GATA6, TGFBR3, HES1, NEO1 |  |
| Regulation Of Rho Protein Signal Transduction | 9 | 6 | 1.25E-04 | 0.013 | | ARHGDIB, ARRB1, ABCA1, ROBO1, TIAM2, LPAR1, GPR65, F2RL1, F2RL2 |  |
| Negative Regulation Of Mapk Cascade | 11 | 5 | 1.25E-04 | 0.018 | | PDCD4, ARRB1, DMD, GBP1, PTPN22, IL1B, LMO3, BMP4, TIMP3, F2RL1, IRAK3 |  |
| Negative Regulation Of Nitrogen Compound Metabolic Process | 61 | 2 | 1.27E-04 | 0.019 | | ARRB1, ATP2B4, BCL2A1, MIR181B1, CARD8, CLU, BMP4, IL24, C4BPB, IRAK3, DMD, TRIM6, PLCB1, CST4, PTPN22, FAM172BP, F2RL1, FBP1, PDCD4, NCKAP1L, GATA6, GBP1, PRICKLE1, GJA1, IL1B, INHBB, TRIB2, ITGB3, FOXA1, HES1, SH3RF2, MEF2C, KCNIP3, BCL11A, SERPINB2, ZNF438, PKIA, LMO3, HMSD, SOX4, SSX1, ZNF608, TCF4, PMEPA1, PRDM8, SNAI2, SAMSN1, VEGFA, WT1, NXN, THBS1, TIMP3, HIST2H4B, CPEB4, GPRC5A, CST7, SERPINB7, GMFG, NOG, RAD50, CTDSPL |  |
| Regulation Of Leukocyte Migration | 11 | 5 | 1.28E-04 | 0.019 | | MTUS1, NCKAP1L, CSF1, PLCB1, PTPN22, IL1B, VEGFA, ITGB3, CCL2, THBS1, F2RL1 |  |
| Positive Regulation Of Transmembrane Receptor Protein Serine/Threonine Kinase Signaling Pathway | 8 | 6 | 1.32E-04 | 0.019 | | GATA6, INHBB, BMP4, TGFB2, THBS1, TGFBR3, HES1, NEO1 |  |
| Response To Hormone | 30 | 2 | 1.37E-04 | 0.017 | | GRB14, PDE3B, GATA6, GJA1, IL1B, FBXO32, INHBB, ITGB3, SCP2, BMP4, HMGCS1, FOXA1, HES1, CCND2, TNFSF10, MEF2C, MMP3, MMP1, PLCB1, LTBP1, RAD50, VEGFA, WT1, TGFB2, THBS1, TGFBR3, TIMP3, EPHA3, F3, FBP1 |  |
| Cellular Response To Mechanical Stimulus | 9 | 6 | 1.38E-04 | 0.021 | | MAP3K1, GJA1, LTBP1, IL1B, IL13RA2, ITGB3, BMP4, TLR3, CRADD |  |
| Adhesion Of Symbiont To Host | 4 | 19 | 1.39E-04 | 0.018 | | INHBB, GBP1, GBP2, GBP3 |  |
| Response To Abiotic Stimulus | 38 | 2 | 1.39E-04 | 0.010 | | ARRB1, ATP2B4, TSPYL5, PLAU, PLCB4, FBXO32, CLU, BMP4, CCND2, SOX4, DMD, SDC2, SNAI2, VEGFA, LPAR1, TGFB2, THBS1, TGFBR3, TIMP3, TLR3, F3, GATA6, GJA1, CXCR4, GNGT2, IL1B, IL13RA2, ITGB3, CPEB4, GPR65, HMGCS1, CRADD, MAP3K1, MMP3, MMP1, PDLIM1, NOG, LTBP1 |  |
| Regulation Of Smooth Muscle Cell Migration | 8 | 6 | 1.42E-04 | 0.020 | | MEF2C, MMP1, HAS2, PLAU, PLXNA1, ITGB3, LPAR1, F3 |  |
| Cell-Cell Junction Organization | 9 | 6 | 1.45E-04 | 0.017 | | HEG1, DSP, GJA1, SNAI2, POF1B, PARD6B, CDH11, TGFB2, F2RL1 |  |
| Cardiac Septum Development | 6 | 9 | 1.46E-04 | 0.018 | | ROBO1, HEG1, BMP4, HES1, CXCR4, LTBP1 |  |
| Regulation Of Transmembrane Transport | 21 | 3 | 1.50E-04 | 0.014 | | ATP2B4, GJA1, PIK3CG, IL1B, CLIC3, ITGB3, CLIC2, SCN5A, SCN9A, BMP4, MEF2C, CEMIP, DMD, KCNQ3, PLCB1, KCNIP3, PTPN22, CLIC6, TGFB2, THBS1, STOM |  |
| Negative Chemotaxis | 5 | 12 | 1.59E-04 | 0.021 | | ROBO1, NRP2, ITGB3, FLRT2, EPHA7 |  |
| Regulation Of Small Gtpase Mediated Signal Transduction | 14 | 4 | 1.60E-04 | 0.013 | | CDON, ARHGDIB, ARRB1, ABCA1, CSF1, ROBO1, TIAM2, LPAR1, GPR65, CDC42BPA, TGFB2, F2RL1, F2RL2, FBP1 |  |
| Chemotaxis | 15 | 3 | 1.64E-04 | 0.020 | | NCKAP1L, CXCR4, PIK3CG, PLAU, ROBO1, NRP2, IL1B, VEGFA, ITGB3, CCL2, LPAR1, BMP4, FLRT2, TGFB2, EPHA7 |  |
| Cell Activation | 30 | 2 | 1.65E-04 | 0.020 | | ARRB1, NCKAP1L, BCL2A1, CXCR4, PIK3CG, PLAU, CD33, CD68, ADGRE5, ITGB3, CLU, CPPED1, DOCK10, SOX4, SLC44A2, GMFG, MEF2C, CRISPLD2, DSP, TCF4, CSF1, LCP1, CTSD, BCL11A, PTPN22, STOM, SLAMF7, TLR3, F2RL1, F2RL2 |  |
| Cellular Response To Hormone Stimulus | 18 | 3 | 1.66E-04 | 0.020 | | GRB14, PDE3B, GATA6, GJA1, FBXO32, INHBB, ITGB3, BMP4, HMGCS1, CCND2, MEF2C, MMP1, PLCB1, LTBP1, VEGFA, WT1, EPHA3, FBP1 |  |
| Negative Regulation Of Cardiac Epithelial To Mesenchymal Transition | 2 | 100 | 1.66E-04 | 0.021 | | TGFB2, NOG |  |
| Negative Regulation Of Epithelial To Mesenchymal Transition Involved In Endocardial Cushion Formation | 2 | 100 | 1.67E-04 | 0.005 | | TGFB2, NOG |  |
| Ventricular Compact Myocardium Morphogenesis | 3 | 33 | 1.67E-04 | 0.005 | | DSP, TGFBR3, NOG |  |
| Positive Regulation Of Map Kinase Activity | 13 | 4 | 1.70E-04 | 0.008 | | ARRB1, MAP3K1, NRK, CXCR4, PIK3CG, ROBO1, IL1B, VEGFA, LPAR1, BMP4, TGFA, THBS1, SAMD5 |  |
| Regulation Of Peptide Transport | 25 | 2 | 1.71E-04 | 0.020 | | TNFRSF21, ARRB1, PKIA, PDE3B, GBP1, GJA1, IL1B, CARD8, INHBB, BMP4, CASK, SOX4, CEMIP, ARL2BP, TRIM6, IL1RL1, KISS1, CPT1A, LCP1, CYP2J2, PTPN22, TGFB2, STOM, F2RL1, NEO1 |  |
| Signaling | 24 | 2 | 1.71E-04 | 0.021 | | PCDH17, PCDH11X, GJA1, CD33, ADGRE5, IL1B, CHRM3, SCN5A, CCL2, PCDHB6, BMP4, FOXA1, HES1, TNFSF10, PCDH11Y, DSP, PCDHB2, KCNQ3, FGF13, SHC3, PCDHB5, EFNB2, TGFB2, PCDHB16 |  |
| Taxis | 15 | 3 | 1.72E-04 | 0.021 | | NCKAP1L, CXCR4, PIK3CG, PLAU, ROBO1, NRP2, IL1B, VEGFA, ITGB3, CCL2, LPAR1, BMP4, FLRT2, TGFB2, EPHA7 |  |
| Positive Regulation Of Chemotaxis | 9 | 5 | 1.75E-04 | 0.020 | | NCKAP1L, CSF1, SNAI2, IL1B, VEGFA, LPAR1, THBS1, F2RL1, F3 |  |
| Ventricular Septum Development | 5 | 12 | 1.78E-04 | 0.018 | | ROBO1, HEG1, HES1, CXCR4, LTBP1 |  |
| Positive Regulation Of Vasculature Development | 12 | 4 | 1.80E-04 | 0.012 | | PLK2, MIR181B1, GATA6, CXCR4, IL1B, VEGFA, ITGB3, EFNB2, THBS1, TLR3, F3, SERPINB7 |  |
| Negative Regulation Of Cell Death | 33 | 2 | 1.84E-04 | 0.019 | | PLK2, ARRB1, BCL2A1, PIK3CG, CLU, CCL2, BMP4, IL24, CCND2, SOX4, SNAI2, VEGFA, WT1, HHIP, TGFA, TGFB2, THBS1, TGFBR3, EPHA7, EPHA3, EYA4, PDCD4, NCKAP1L, GATA6, IL1B, ITGB3, CPEB4, TNFSF10, MEF2C, NOG, OPTN, SERPINB2, NEO1 |  |
| Regulation Of Erk1 And Erk2 Cascade | 15 | 3 | 1.84E-04 | 0.020 | | ARRB1, DMD, GBP1, PTPN22, IL1B, VEGFA, LMO3, ITGB3, CCL2, BMP4, TGFBR3, TIMP3, EPHA7, EPHA3, F2RL1 |  |
| Osteoblast Differentiation | 8 | 6 | 1.85E-04 | 0.020 | | CYP24A1, MEF2C, NOG, GJA1, SNAI2, RRBP1, BMP4, TGFBR3 |  |
| Artery Morphogenesis | 6 | 9 | 1.94E-04 | 0.017 | | ZMIZ1, VEGFA, BMP4, TGFB2, NOG, HES1 |  |
| Positive Regulation Of Extrinsic Apoptotic Signaling Pathway | 6 | 9 | 1.95E-04 | 0.014 | | SRPX, TGFB2, THBS1, TIMP3, G0S2, TNFSF10 |  |
| Regulation Of Protein Transport | 24 | 2 | 1.95E-04 | 0.014 | | TNFRSF21, ARRB1, PKIA, PDE3B, GBP1, GJA1, IL1B, CARD8, INHBB, BMP4, CASK, SOX4, CEMIP, ARL2BP, TRIM6, IL1RL1, CPT1A, LCP1, CYP2J2, PTPN22, TGFB2, STOM, F2RL1, NEO1 |  |
| Negative Regulation Of Chondrocyte Differentiation | 4 | 17 | 1.96E-04 | 0.021 | | ADAMTS12, BMP4, SNAI2, EFEMP1 |  |
| Cellular Response To Sterol | 4 | 17 | 2.01E-04 | 0.010 | | INHBB, SCP2, ABCA1, HMGCS1 |  |
| Cellular Developmental Process | 73 | 1 | 2.01E-04 | 0.010 | | BCL2A1, CDH11, SLITRK1, CLU, BMP4, BMP1, DMKN, CYP24A1, TDRD7, DMD, DPYSL2, DSP, CPT1A, CSF1, PLCB1, FLG, PTPN22, LPAR1, EFNB2, EPHA7, EPHA3, EYA4, F2RL1, PDCD4, HAS2, NCKAP1L, GATA6, GJA1, IL1B, INHBB, ITGB3, FOXA1, HES1, CDON, MEF2C, MAP3K1, NRK, SAMD9L, KCNQ3, SNX10, LAMA3, LAMA4, BCL11A, RBM47, TENM3, ROBO1, RRBP1, LAMA1, PTPRS, DOCK10, SOX4, ZMIZ1, HEG1, TCF4, SDC2, PRDM8, SNAI2, VEGFA, WT1, NXN, TGFB2, TGFBR3, TLL1, SLC7A5, ILDR2, CXCR4, POF1B, NRP2, FAT4, LGR5, NOG, TSPAN2, PARD6B |  |
| Negative Regulation Of Toll-Like Receptor Signaling Pathway | 5 | 11 | 2.01E-04 | 0.018 | | PTPRS, PIK3AP1, TLR3, F2RL1, IRAK3 |  |
| Positive Regulation Of Neuron Differentiation | 17 | 3 | 2.02E-04 | 0.012 | | CXCR4, PLXNA2, PLXNA1, TENM3, ROBO1, SLITRK1, BMP4, FOXA1, CASK, CDON, MEF2C, DMD, TCF4, BCL11A, VEGFA, LPAR1, EPHA3 |  |
| Regulation Of Morphogenesis Of A Branching Structure | 6 | 9 | 2.12E-04 | 0.020 | | BCL11A, IL1B, VEGFA, BMP4, NOG, SNAI2 |  |
| Positive Regulation Of Cytosolic Calcium Ion Concentration | 14 | 3 | 2.12E-04 | 0.014 | | CEMIP, ATP2B4, KISS1, GJA1, CXCR4, PIK3CG, IL1B, LPAR1, S1PR3, GPR65, BMP4, TGM2, F2RL1, F2RL2 |  |
| Regulation Of Production Of Molecular Mediator Of Immune Response | 9 | 5 | 2.14E-04 | 0.020 | | HLA-A, TRIM6, PTPN22, IL1B, IL13RA2, TGFB2, TLR3, F2RL1, IRAK3 |  |
| Regulation Of Cell-Matrix Adhesion | 8 | 6 | 2.16E-04 | 0.018 | | DMD, CSF1, PLAU, VEGFA, ITGB3, THBS1, CASK, EPHA3 |  |
| Platelet Degranulation | 8 | 6 | 2.17E-04 | 0.017 | | VEGFA, ITGB3, CLU, TGFB2, THBS1, RAB27B, TIMP3, F13A1 |  |
| Regulation Of Secretion | 27 | 2 | 2.17E-04 | 0.017 | | TNFRSF21, ARRB1, PDE3B, GBP1, GJA1, MCTP2, IL1B, IL13RA2, CARD8, INHBB, STXBP6, CASK, RAB27B, SYTL3, SOX4, MEF2C, ARL2BP, DPYSL2, TRIM6, IL1RL1, KISS1, CPT1A, CYP2J2, PTPN22, TGFB2, F2RL1, NEO1 |  |
| Negative Regulation Of Cellular Component Organization | 24 | 2 | 2.17E-04 | 0.020 | | PLK2, HLA-A, GBP1, NAV3, CLU, BMP4, PTPRS, HES1, IRAK3, GMFG, MAP3K1, PMEPA1, TBC1D30, ASAP1, FGF13, BCL11A, RAD50, LPAR1, EFNB2, THBS1, EPHA7, EPHA3, NEO1, EFEMP1 |  |
| Regulation Of Cell-Substrate Adhesion | 11 | 4 | 2.18E-04 | 0.021 | | DMD, HAS2, GBP1, CSF1, PLAU, VEGFA, ITGB3, THBS1, CASK, EPHA3, ABI3BP |  |
| Cell Junction Organization | 11 | 4 | 2.19E-04 | 0.019 | | HEG1, DSP, LAMA3, GJA1, SNAI2, POF1B, PARD6B, CDH11, ITGB3, TGFB2, F2RL1 |  |
| Transmembrane Receptor Protein Tyrosine Kinase Signaling Pathway | 18 | 3 | 2.19E-04 | 0.019 | | GRB14, NCKAP1L, NRP2, ITGB3, FAT4, SDC2, NOG, CSF1, PLCB1, SHC3, VEGFA, FLRT2, EFNB2, TGFA, EPHA7, EPHA3, EFEMP1, FAM83B |  |
| Cellular Response To Interferon-Beta | 5 | 11 | 2.20E-04 | 0.020 | | TRIM6, GBP1, GBP2, GBP3, TLR3 |  |
| Multicellular Organism Development | 38 | 2 | 2.27E-04 | 0.012 | | ARHGDIB, PLXNA1, ROBO1, CDH11, MCTP2, BMP4, BMP1, TDRD7, ZMIZ1, HEG1, DPYSL2, TCF4, SNAI2, FLG, VEGFA, NXN, FLRT2, EFNB2, TGFB2, TGFBR3, EPHA7, EPHA3, TLL1, EYA4, SLC7A5, SHISA2, GATA6, EVI5, GJA1, NRP2, INHBB, GPR65, FOXA1, SHISA3, HES1, MEF2C, NOG, BCL11A |  |
| Tube Development | 12 | 4 | 2.32E-04 | 0.020 | | GATA6, NOG, GJA1, PLXNA2, INHBB, ITGB3, WT1, FAT4, BMP4, TGFB2, HES1, CLMP |  |
| Negative Regulation Of Protein Secretion | 9 | 5 | 2.34E-04 | 0.019 | | TNFRSF21, PDE3B, GBP1, PTPN22, IL1B, CARD8, INHBB, F2RL1, NEO1 |  |
| Positive Regulation Of Sterol Transport | 4 | 16 | 2.38E-04 | 0.017 | | SCP2, ABCA1, PLTP, LIPG |  |
| Positive Regulation Of Cholesterol Transport | 4 | 16 | 2.39E-04 | 0.010 | | SCP2, ABCA1, PLTP, LIPG |  |
| Membranous Septum Morphogenesis | 3 | 30 | 2.39E-04 | 0.010 | | BMP4, TGFB2, NOG |  |
| Regulation Of Transforming Growth Factor Beta1 Production | 3 | 30 | 2.41E-04 | 0.008 | | GATA6, THBS1, SERPINB7 |  |
| Positive Regulation Of Cd8-Positive, Alpha-Beta T Cell Activation | 3 | 30 | 2.41E-04 | 0.008 | | PTPN22, NCKAP1L, HLA-A |  |
| Regulation Of Leukocyte Chemotaxis | 8 | 6 | 2.41E-04 | 0.008 | | MTUS1, NCKAP1L, CSF1, IL1B, VEGFA, CCL2, THBS1, F2RL1 |  |
| Divalent Inorganic Cation Homeostasis | 19 | 3 | 2.42E-04 | 0.017 | | ATP2B4, GJA1, CXCR4, PIK3CG, EFHC1, IL1B, ITGB3, SCN5A, CCL2, GPR65, BMP4, CEMIP, SNX10, KISS1, LPAR1, S1PR3, TGM2, F2RL1, F2RL2 |  |
| Regulation Of Heart Morphogenesis | 5 | 11 | 2.53E-04 | 0.020 | | ROBO1, BMP4, TGFB2, NOG, HES1 |  |
| Cellular Response To Nitrogen Compound | 22 | 2 | 2.53E-04 | 0.012 | | GRB14, ATP2B4, PDE3B, PIK3CG, PLAU, INHBB, ITGB3, CPEB4, CCND2, MEF2C, MMP3, MMP1, KCNQ3, IL1RL1, PLCB1, BCL11A, OPTN, PTPN22, WT1, THBS1, TLR3, FBP1 |  |
| Response To Growth Factor | 16 | 3 | 2.62E-04 | 0.020 | | ADAMTS12, MEF2C, HAS2, GATA6, NOG, PLAU, SNAI2, LTBP1, OPTN, VEGFA, ITGB3, CLU, CCL2, BMP4, THBS1, HES1 |  |
| Positive Regulation Of Transport | 31 | 2 | 2.70E-04 | 0.020 | | ARRB1, NCKAP1L, ABCA1, GJA1, CXCR4, PLTP, IL1B, CARD8, INHBB, CLU, SCN5A, SCP2, CCL2, BMP4, CASK, RAB27B, SOX4, CEMIP, DMD, DPYSL2, TRIM6, IL1RL1, KISS1, PLCB1, LIPG, CYP2J2, PTPN22, VEGFA, TGFB2, STOM, F2RL1 |  |
| Negative Regulation Of Apoptotic Process | 30 | 2 | 2.74E-04 | 0.020 | | PDCD4, PLK2, ARRB1, NCKAP1L, BCL2A1, GATA6, PIK3CG, IL1B, CPEB4, CLU, CCL2, BMP4, IL24, CCND2, TNFSF10, MEF2C, NOG, SNAI2, OPTN, VEGFA, WT1, SERPINB2, HHIP, TGFA, TGFB2, THBS1, TGFBR3, EPHA7, EPHA3, EYA4 |  |
| Negative Regulation Of Camp-Mediated Signaling | 4 | 15 | 2.75E-04 | 0.020 | | ATP2B4, PDE10A, LPAR1, PDE3B |  |
| Heart Trabecula Morphogenesis | 4 | 15 | 2.81E-04 | 0.010 | | HEG1, TGFB2, TGFBR3, NOG |  |
| Angiogenesis | 13 | 4 | 2.81E-04 | 0.010 | | PDE3B, PIK3CG, PLAU, NRP2, VEGFA, ITGB3, CCL2, BMP4, EFNB2, TGFA, TGFB2, THBS1, ESM1 |  |
| Regulation Of Response To Wounding | 10 | 5 | 2.82E-04 | 0.019 | | GJA1, CXCR4, PLAU, SERPINB2, PTPRS, THBS1, CASK, F2RL1, F3, NEO1 |  |
| Cell-Cell Signaling | 22 | 2 | 2.84E-04 | 0.018 | | PCDH17, PCDH11X, GJA1, CD33, ADGRE5, IL1B, CHRM3, CCL2, PCDHB6, BMP4, FOXA1, HES1, TNFSF10, PCDH11Y, PCDHB2, KCNQ3, FGF13, SHC3, PCDHB5, EFNB2, TGFB2, PCDHB16 |  |
| Cellular Response To External Stimulus | 16 | 3 | 2.87E-04 | 0.020 | | MAP3K1, MMP3, MMP1, GJA1, LTBP1, BCL11A, OPTN, IL1B, IL13RA2, INHBB, GDAP1, ITGB3, CPEB4, BMP4, TLR3, CRADD |  |
| Glial Cell Development | 6 | 8 | 2.91E-04 | 0.020 | | SOX4, TSPAN2, DMD, LPAR1, KCNQ3, PRDM8 |  |
| Heart Valve Morphogenesis | 5 | 10 | 2.94E-04 | 0.014 | | SOX4, ROBO1, MEF2C, BMP4, TGFB2 |  |
| Positive Regulation Of Vascular Endothelial Growth Factor Receptor Signaling Pathway | 4 | 15 | 3.13E-04 | 0.012 | | IL1B, VEGFA, ITGB3, BMP4 |  |
| Positive Regulation Of Toll-Like Receptor Signaling Pathway | 4 | 15 | 3.29E-04 | 0.010 | | PTPN22, PIK3AP1, TLR3, F2RL1 |  |
| Positive Regulation Of Mesonephros Development | 4 | 15 | 3.29E-04 | 0.010 | | VEGFA, WT1, BMP4, NOG |  |
| Regulation Of Interleukin-8 Production | 6 | 8 | 3.29E-04 | 0.010 | | AFAP1L2, PTPN22, IL1B, ARRB1, TLR3, F2RL1 |  |
| Regulation Of Calcium Ion Transport | 13 | 3 | 3.42E-04 | 0.014 | | CEMIP, DMD, GJA1, CXCR4, PIK3CG, PTPN22, ITGB3, CLIC2, CCL2, BMP4, TGFB2, CASK, HES1 |  |
| Negative Regulation Of Programmed Cell Death | 30 | 2 | 3.48E-04 | 0.019 | | PDCD4, PLK2, ARRB1, NCKAP1L, BCL2A1, GATA6, PIK3CG, IL1B, CPEB4, CLU, CCL2, BMP4, IL24, CCND2, TNFSF10, MEF2C, NOG, SNAI2, OPTN, VEGFA, WT1, SERPINB2, HHIP, TGFA, TGFB2, THBS1, TGFBR3, EPHA7, EPHA3, EYA4 |  |
| Positive Regulation Of Animal Organ Morphogenesis | 7 | 6 | 3.61E-04 | 0.020 | | NOG, CSF1, ROBO1, VEGFA, WT1, BMP4, TGFB2 |  |
| Heart Morphogenesis | 6 | 8 | 3.69E-04 | 0.015 | | ZMIZ1, VEGFA, FAT4, FLRT2, TGFB2, TGFBR3 |  |
| Negative Regulation Of Peptide Secretion | 9 | 5 | 3.69E-04 | 0.014 | | TNFRSF21, PDE3B, GBP1, PTPN22, IL1B, CARD8, INHBB, F2RL1, NEO1 |  |
| Negative Regulation Of Cell Adhesion | 13 | 3 | 3.73E-04 | 0.017 | | TNFRSF21, ARHGDIB, ZNF608, PDE3B, GBP1, SNAI2, PLXNA2, PLXNA1, PTPN22, VEGFA, BMP4, THBS1, CASK |  |
| Positive Regulation Of Endothelial Cell Apoptotic Process | 4 | 14 | 3.79E-04 | 0.019 | | PDCD4, CCL2, BMP4, THBS1 |  |
| Regulation Of Collateral Sprouting | 4 | 14 | 3.83E-04 | 0.010 | | FGF13, BCL11A, PTPRS, EPHA7 |  |
| Regulation Of Toll-Like Receptor Signaling Pathway | 6 | 8 | 3.83E-04 | 0.010 | | PTPN22, PTPRS, PIK3AP1, TLR3, F2RL1, IRAK3 |  |
| Intracellular Signal Transduction | 41 | 2 | 3.97E-04 | 0.014 | | ARHGDIB, PLK2, BCL2A1, ABCA1, PIK3CG, ADGRF1, PLCB4, MCTP2, CNKSR2, CLU, CLIC2, CCL2, RAB27B, DOCK10, IRAK3, SOX4, HRASLS, DMD, PLCB1, FGF13, TUFT1, TIAM2, TGFBR3, TLR3, GRB14, TNS3, CXCR4, IGFBP4, IL1B, GPR65, FAT4, CDC42BPA, LGR5, CRADD, MEF2C, MAP3K1, NRK, LCP1, SHC3, RAPGEF5, NUAK1 |  |
| Regulation Of Primary Metabolic Process | 123 | 1 | 4.12E-04 | 0.019 | | ARRB1, ANKRD30A, ATP2B4, ARRDC4, BCL2A1, AADAC, ABCA1, TSPYL5, CLU, BMP4, C4BPB, CCND2, DMD, TRIM6, PIK3AP1, CPT1A, CSF1, CST4, FGF13, FOXR2, LPAR1, EPHA7, EPHA3, EYA4, FAM172BP, F2RL1, F3, ACSL1, EFEMP1, FBP1, HAS2, NCKAP1L, GATA6, GBP1, PRICKLE1, GJA1, IGFBP4, IL1B, INHBB, ITGB3, HMGCS1, FOXA1, HES1, SH3RF2, MEF2C, MEIS3P1, MAP3K1, NRK, SAMD9L, KISS1, SERPINB2, ZNF438, NEO1, PKIA, PDE3B, PIK3CG, ADGRF1, SSX8, ROBO1, SCP2, CCL2, HMSD, SOX4, SSX1, TCF4, SNAI2, VEGFA, WT1, TGFA, TGFB2, THBS1, TGFBR3, TIMP3, TLR3, ZNF391, HIST2H4B, ZNF43, CXCR4, GPRC5A, CST7, CASK, SAMD5, SERPINB7, TNFSF10, CRADD, GMFG, PDLIM1, NOG, LIPG, RAD50, MBNL2, CTDSPL, NUAK1, PLK2, ZNF716, MIR181B1, CARD8, IL24, IRAK3, SP140, ARL2BP, PLCB1, PTPN22, TIAM2, TNFRSF21, PDCD4, TRIB2, CDON, MAFIP, KCNIP3, G0S2, BCL11A, LMO3, ZMIZ1, CEMIP, ZNF608, PMEPA1, PRDM9, PRDM8, SAMSN1, NXN, CPEB4, AFAP1L2 |  |
| Organophosphate Ester Transport | 7 | 6 | 4.14E-04 | 0.016 | | ATP8B2, ABCA1, GJA1, PLTP, PITPNC1, SCP2, SLC37A2 |  |
| Regulation Of Establishment Of Protein Localization | 24 | 2 | 4.15E-04 | 0.015 | | TNFRSF21, ARRB1, PKIA, PDE3B, GBP1, GJA1, IL1B, CARD8, INHBB, BMP4, CASK, SOX4, CEMIP, ARL2BP, TRIM6, IL1RL1, CPT1A, LCP1, CYP2J2, PTPN22, TGFB2, STOM, F2RL1, NEO1 |  |
| Chemical Homeostasis | 32 | 2 | 4.17E-04 | 0.020 | | HECTD4, ATP2B4, PDE3B, ABCA1, PIK3CG, PLAU, EFHC1, SCN5A, CCL2, BMP4, ACOXL, SOX4, CEMIP, TBXAS1, FLG, VEGFA, LPAR1, S1PR3, TGM2, F2RL1, F2RL2, GJA1, CXCR4, IL1B, ITGB3, GPR65, FOXA1, MEF2C, SNX10, KISS1, LIPG, NEO1 |  |
| Positive Regulation Of Smooth Muscle Cell Migration | 6 | 7 | 4.17E-04 | 0.020 | | MMP1, HAS2, ITGB3, LPAR1, PLAU, F3 |  |
| Positive Regulation Of Glomerulus Development | 3 | 25 | 4.27E-04 | 0.014 | | ITGB3, NOG, SERPINB7 |  |
| Cellular Response To Fluid Shear Stress | 4 | 14 | 4.34E-04 | 0.008 | | MEF2C, MMP1, HAS2, PLAU |  |
| Animal Organ Development | 44 | 2 | 4.42E-04 | 0.010 | | AK4, ROBO1, BMP4, BMP1, PTPRS, CCND2, SOX4, DMD, HEG1, DPYSL2, DSP, CSF1, PLCB1, ADGRL3, VEGFA, WT1, LPAR1, TGFB2, THBS1, TGFBR3, EPHA7, TLR3, EFEMP1, ILDR2, HAS2, GATA6, GJA1, CXCR4, NRP2, INHBB, FAT4, HMGCS1, FOXA1, HES1, TNFSF10, MEF2C, MAP3K1, CRISPLD2, SAMD9L, KCNQ3, NOG, LCP1, TSPAN2, RAD50 |  |
| Negative Regulation Of Secretion By Cell | 11 | 4 | 4.45E-04 | 0.019 | | TNFRSF21, PDE3B, GBP1, PTPN22, IL1B, IL13RA2, CARD8, INHBB, STXBP6, F2RL1, NEO1 |  |
| Cellular Response To Lipid | 22 | 2 | 4.45E-04 | 0.018 | | PDCD4, ABCA1, GBP2, PLAU, IL1B, FBXO32, INHBB, SCP2, CCL2, BMP4, IL24, HMGCS1, HES1, MEF2C, MMP1, CPT1A, PLCB1, LTBP1, VEGFA, GDAP1, LPAR1, EPHA3 |  |
| Regulation Of Animal Organ Morphogenesis | 11 | 4 | 4.58E-04 | 0.020 | | NOG, PRICKLE1, CSF1, PLAU, SNAI2, ROBO1, VEGFA, WT1, BMP4, TGFB2, HES1 |  |
| Positive Regulation Of Cardiocyte Differentiation | 5 | 10 | 4.61E-04 | 0.018 | | MEF2C, BMP4, EFNB2, TGFB2, GATA6 |  |
| Response To Cytokine | 26 | 2 | 4.63E-04 | 0.012 | | TNFRSF21, HAS2, HLA-A, GBP1, GBP2, GBP3, LAPTM5, CXCR4, NRP2, INHBB, CCL2, IL24, HES1, IRAK3, ADAMTS12, PTGES, MMP3, MMP1, TRIM6, SNX10, OPTN, TGFB2, THBS1, TIMP3, EPHA3, TLR3 |  |
| Cellular Calcium Ion Homeostasis | 17 | 3 | 4.66E-04 | 0.020 | | ATP2B4, GJA1, CXCR4, PIK3CG, EFHC1, IL1B, ITGB3, CCL2, GPR65, BMP4, CEMIP, KISS1, LPAR1, S1PR3, TGM2, F2RL1, F2RL2 |  |
| Cell Death | 27 | 2 | 4.84E-04 | 0.020 | | TNFRSF21, PDCD4, ARRB1, BCL2A1, GJA1, CXCR4, PIK3CG, PLAU, IL1B, CARD8, GPR65, BMP4, IL24, IRAK3, TNFSF10, CRADD, MEF2C, DSP, KCNIP3, G0S2, FGF13, OPTN, FLG, WT1, TGFB2, EPHA7, TLR3 |  |
| Regulation Of Cellular Response To Stress | 22 | 2 | 4.84E-04 | 0.020 | | PDCD4, PLK2, MIR181B1, IL1B, CLU, PTPRS, SAMD5, MAP3K1, MMP3, NRK, KISS1, PLCB1, SNAI2, PTPN22, VEGFA, TGFB2, TGFBR3, NUAK1, EYA4, TLR3, F2RL1, NEO1 |  |
| Epicardial Cell To Mesenchymal Cell Transition | 2 | 66 | 4.91E-04 | 0.020 | | TGFBR3, GJA1 |  |
| Positive Regulation Of Epithelial Cell Migration | 9 | 5 | 4.97E-04 | 0.005 | | PLK2, HAS2, SNAI2, NRP2, VEGFA, ITGB3, BMP4, TGFB2, THBS1 |  |
| Regulation Of Mesonephros Development | 4 | 13 | 5.01E-04 | 0.017 | | VEGFA, WT1, BMP4, NOG |  |
| Blood Vessel Remodeling | 5 | 9 | 5.07E-04 | 0.010 | | MEF2C, VEGFA, TGFB2, TGFBR3, TGM2 |  |
| Positive Regulation Of Striated Muscle Tissue Development | 7 | 6 | 5.08E-04 | 0.012 | | CDON, MEF2C, GATA6, GJA1, BMP4, EFNB2, TGFBR3 |  |
| Positive Regulation Of Muscle Organ Development | 7 | 6 | 5.21E-04 | 0.015 | | CDON, MEF2C, GATA6, GJA1, BMP4, EFNB2, TGFBR3 |  |
| Regulation Of Jnk Cascade | 10 | 4 | 5.21E-04 | 0.015 | | PDCD4, MAP3K1, NRK, PLCB1, PTPN22, IL1B, TGFBR3, SAMD5, TLR3, F2RL1 |  |
| Immune System Process | 59 | 1 | 5.23E-04 | 0.018 | | BCL2A1, CD33, CD68, ADGRE5, CLU, IFI44L, C4BPB, IRAK3, DSP, PIK3AP1, CSF1, CTSD, PTPN22, EFNB2, STOM, F2RL1, TNFRSF21, GRB14, NCKAP1L, HLA-A, HLA-B, GBP1, GBP2, GBP3, IL1B, IL13RA2, ITGB3, HLA-DRA, MEF2C, MAP3K1, MMP1, LCP1, PLPP4, BCL11A, PIK3CG, PLAU, CCL2, HMSD, CPPED1, DOCK10, SOX4, SLC44A2, SDC2, VEGFA, TGFB2, THBS1, TGFBR3, SLAMF7, TLR3, SLC7A5, CXCR4, GPR65, CST7, TNFSF10, GMFG, CRISPLD2, FCGR2C, IL1RL1, OPTN |  |
| Positive Regulation Of Metabolic Process | 75 | 1 | 5.29E-04 | 0.018 | | PLK2, ARRB1, ATP2B4, ARRDC4, MIR181B1, AADAC, TSPYL5, CARD8, CLU, BMP4, IL24, C4BPB, CCND2, ARL2BP, TRIM6, CPT1A, CSF1, PLCB1, FGF13, PTPN22, LPAR1, EPHA7, EPHA3, EYA4, F2RL1, F3, ACSL1, HAS2, NCKAP1L, GATA6, PRICKLE1, GJA1, IGFBP4, IL1B, INHBB, TRIB2, ITGB3, FOXA1, HES1, CDON, MEF2C, MAP3K1, NRK, KISS1, LTBP1, BCL11A, ZNF438, PIK3CG, PLAU, ROBO1, SCP2, CCL2, SOX4, ZMIZ1, CEMIP, TCF4, PRDM9, SNAI2, VEGFA, WT1, TGFA, TGFB2, THBS1, TLR3, CXCR4, CASK, SAMD5, SERPINB7, TNFSF10, CRADD, PDLIM1, NOG, RAD50, AFAP1L2, OPTN |  |
| Leukocyte Activation | 26 | 2 | 5.37E-04 | 0.017 | | NCKAP1L, BCL2A1, CXCR4, PIK3CG, PLAU, CD33, CD68, ADGRE5, CLU, CPPED1, DOCK10, SOX4, SLC44A2, GMFG, MEF2C, CRISPLD2, DSP, CSF1, LCP1, CTSD, BCL11A, PTPN22, STOM, SLAMF7, TLR3, F2RL1 |  |
| Positive Regulation Of Muscle Tissue Development | 7 | 6 | 5.50E-04 | 0.020 | | CDON, MEF2C, GATA6, GJA1, BMP4, EFNB2, TGFBR3 |  |
| Calcium-Dependent Cell-Cell Adhesion Via Plasma Membrane Cell Adhesion Molecules | 5 | 9 | 5.51E-04 | 0.015 | | CDH11, PCDHB5, PCDHB6, PCDHB2, PCDHB16 |  |
| Semaphorin-Plexin Signaling Pathway Involved In Neuron Projection Guidance | 3 | 23 | 5.57E-04 | 0.012 | | NRP2, PLXNA2, PLXNA1 |  |
| Venous Blood Vessel Morphogenesis | 3 | 23 | 5.59E-04 | 0.008 | | VEGFA, HEG1, EFNB2 |  |
| Regulation Of Apoptotic Signaling Pathway | 16 | 3 | 5.59E-04 | 0.008 | | SRPX, BCL2A1, IL1B, INHBB, CLU, BMP4, TNFSF10, CRADD, NOG, G0S2, SNAI2, VEGFA, TGFB2, THBS1, TIMP3, EYA4 |  |
| Regulation Of Signaling Receptor Activity | 19 | 3 | 5.73E-04 | 0.019 | | PLAU, IL1B, INHBB, GPRC5A, CCL2, BMP4, BMP1, IL24, TNFSF10, GMFG, MEF2C, NOG, CSF1, FGF13, VEGFA, TGFA, TGFB2, LYPD6B, EFEMP1 |  |
| Regulation Of Cell Morphogenesis | 18 | 3 | 5.80E-04 | 0.020 | | HAS2, GBP1, CXCR4, PLXNA2, PLXNA1, ROBO1, SLITRK1, ITGB3, CCL2, PTPRS, CASK, DPYSL2, SDC2, FGF13, BCL11A, VEGFA, LPAR1, EPHA7 |  |
| Positive Regulation Of I-Kappab Kinase/Nf-Kappab Signaling | 9 | 5 | 5.88E-04 | 0.019 | | SLC44A2, PLK2, GJA1, IL1B, LPAR1, TGM2, TLR3, F2RL1, TNFSF10 |  |
| Phospholipase C Activity | 4 | 13 | 5.89E-04 | 0.017 | | CHRM3, PLCB1, PLCB4, F2RL2 |  |
| Regulation Of Cardiocyte Differentiation | 6 | 7 | 5.91E-04 | 0.010 | | MEF2C, BMP4, EFNB2, TGFB2, GATA6, PRICKLE1 |  |
| Regulation Of Endothelial Cell Differentiation | 5 | 9 | 6.04E-04 | 0.014 | | IL1B, VEGFA, MIR181B1, S1PR3, BMP4 |  |
| Positive Regulation Of Peptide Secretion | 13 | 3 | 6.08E-04 | 0.012 | | SOX4, ARRB1, TRIM6, IL1RL1, KISS1, GJA1, CYP2J2, PTPN22, IL1B, CARD8, TGFB2, CASK, F2RL1 |  |
| Regulation Of Cation Transmembrane Transport | 14 | 3 | 6.10E-04 | 0.019 | | MEF2C, CEMIP, DMD, ATP2B4, PLCB1, PIK3CG, KCNIP3, PTPN22, ITGB3, CLIC2, SCN5A, BMP4, TGFB2, STOM |  |
| Positive Regulation Of Cytokine Production | 16 | 3 | 6.13E-04 | 0.019 | | HLA-A, IL1B, CARD8, CLU, SERPINB7, HEG1, TRIM6, IL1RL1, PLCB1, CYP2J2, AFAP1L2, OPTN, PTPN22, THBS1, TLR3, F2RL1 |  |
| Phosphoric Ester Hydrolase Activity | 15 | 3 | 6.26E-04 | 0.019 | | PDE10A, PDE3B, PLCB1, PLCB4, PLPP4, ENTPD3, PTPN22, CTDSPL, CHRM3, PTPRS, CPPED1, EYA4, F2RL2, FBP1, FAM83B |  |
| Regulation Of Axonogenesis | 10 | 4 | 6.27E-04 | 0.019 | | DPYSL2, PLXNA2, PLXNA1, ROBO1, FGF13, BCL11A, SLITRK1, VEGFA, PTPRS, EPHA7 |  |
| Regulation Of Gliogenesis | 8 | 5 | 6.48E-04 | 0.017 | | TNFRSF21, NOG, CSF1, CXCR4, IL1B, BMP4, HES1, EFEMP1 |  |
| Response To Cholesterol | 4 | 12 | 6.50E-04 | 0.016 | | INHBB, SCP2, ABCA1, HMGCS1 |  |
| Aorta Development | 4 | 12 | 6.58E-04 | 0.010 | | ROBO1, PRICKLE1, CXCR4, LTBP1 |  |
| Cellular Response To Organic Cyclic Compound | 19 | 2 | 6.58E-04 | 0.010 | | ATP2B4, ABCA1, PIK3CG, PLAU, IL1B, FBXO32, INHBB, SCP2, CCL2, BMP4, HMGCS1, MEF2C, MMP1, PLCB1, LTBP1, VEGFA, GDAP1, WT1, TLR3 |  |
| Positive Regulation Of Hydrolase Activity | 23 | 2 | 6.62E-04 | 0.019 | | ARHGDIB, ARRB1, NCKAP1L, EVI5, ROBO1, CARD8, TOR1AIP2, CCL2, GPR65, IL24, DOCK10, RGS10, TNFSF10, CRADD, MEF2C, PLCB1, TBC1D30, ASAP1, TIAM2, VEGFA, LPAR1, F2RL1, F3 |  |
| Negative Regulation Of Transport | 19 | 2 | 6.79E-04 | 0.019 | | TNFRSF21, PKIA, PDE3B, GBP1, PLCB4, IL1B, IL13RA2, CARD8, INHBB, ITGB3, CLIC2, STXBP6, HES1, PTPN22, TGFB2, THBS1, EPHA3, F2RL1, NEO1 |  |
| Regulation Of Cysteine-Type Endopeptidase Activity Involved In Apoptotic Process | 11 | 4 | 6.87E-04 | 0.019 | | ARRB1, BCL2A1, ROBO1, CARD8, VEGFA, IL24, THBS1, EPHA7, F3, TNFSF10, CRADD |  |
| Regulation Of Nitric Oxide Mediated Signal Transduction | 3 | 21 | 7.00E-04 | 0.018 | | ATP2B4, VEGFA, THBS1 |  |
| Regulation Of Cytosolic Calcium Ion Concentration | 14 | 3 | 7.04E-04 | 0.008 | | CEMIP, ATP2B4, KISS1, GJA1, CXCR4, PIK3CG, IL1B, LPAR1, S1PR3, GPR65, BMP4, TGM2, F2RL1, F2RL2 |  |
| Ventricular Cardiac Muscle Tissue Morphogenesis | 5 | 9 | 7.11E-04 | 0.019 | | HEG1, DSP, TGFB2, TGFBR3, NOG |  |
| Regulation Of Stem Cell Differentiation | 6 | 7 | 7.22E-04 | 0.012 | | TRIM6, GATA6, PRICKLE1, BMP4, TGFB2, HES1 |  |
| Negative Regulation Of Protein Transport | 10 | 4 | 7.35E-04 | 0.014 | | TNFRSF21, PKIA, PDE3B, GBP1, PTPN22, IL1B, CARD8, INHBB, F2RL1, NEO1 |  |
| Cellular Response To Drug | 16 | 3 | 7.43E-04 | 0.017 | | CXCR4, IL1B, FBXO32, ITGB3, BMP4, MEF2C, MMP3, MMP1, BCL11A, OPTN, PTPN22, VEGFA, THBS1, TLR3, F3, FBP1 |  |
| Regulation Of Transmembrane Receptor Protein Serine/Threonine Kinase Signaling Pathway | 11 | 4 | 7.45E-04 | 0.019 | | GATA6, NOG, PMEPA1, LTBP1, INHBB, BMP4, TGFB2, THBS1, TGFBR3, HES1, NEO1 |  |
| Glycosaminoglycan Binding | 11 | 4 | 7.45E-04 | 0.018 | | CEMIP, CRISPLD2, MAMDC2, LIPG, NRP2, VEGFA, BMP4, PTPRS, THBS1, TGFBR3, ABI3BP |  |
| Neuron Migration | 8 | 5 | 7.54E-04 | 0.018 | | MEF2C, GJA1, CXCR4, ADGRL3, FGF13, NRP2, VEGFA, NEO1 |  |
| Positive Regulation Of Cell Projection Organization | 16 | 3 | 7.76E-04 | 0.016 | | CXCR4, NAV3, PLXNA2, PLXNA1, TENM3, ROBO1, SLITRK1, BMP4, CASK, DMD, BCL11A, VEGFA, LPAR1, EPHA3, F2RL1, EFEMP1 |  |
| Spinal Cord Development | 5 | 8 | 7.78E-04 | 0.019 | | SOX4, ROBO1, DPYSL2, PTPRS, NOG |  |
| Regulation Of Extrinsic Apoptotic Signaling Pathway In Absence Of Ligand | 5 | 8 | 7.84E-04 | 0.012 | | IL1B, SRPX, TGFB2, EYA4, SNAI2 |  |
| Positive Regulation Of Secretion By Cell | 16 | 3 | 7.84E-04 | 0.012 | | ARRB1, GJA1, IL1B, CARD8, INHBB, CASK, RAB27B, SOX4, DPYSL2, TRIM6, IL1RL1, KISS1, CYP2J2, PTPN22, TGFB2, F2RL1 |  |
| Mucopolysaccharide Metabolic Process | 7 | 6 | 7.94E-04 | 0.019 | | CEMIP, HAS2, MAMDC2, B4GALT6, IL1B, VEGFA, CHST15 |  |
| Regulation Of Reproductive Process | 9 | 4 | 7.98E-04 | 0.015 | | GRB14, ARHGDIB, NRK, PLCB1, PLAU, PRDM9, INHBB, WT1, BMP4 |  |
| Protein Phosphorylation | 26 | 2 | 8.35E-04 | 0.017 | | PLK2, PIK3CG, CDK15, IL1B, TRIB2, CCL2, LAMA1, BMP4, CDC42BPA, IL24, CASK, IRAK3, GMFG, MEF2C, MAP3K1, NRK, FGF13, SHC3, EFNB2, TGFB2, TGFBR3, EPHA7, EPHA3, NUAK1, TLR3, EFEMP1 |  |
| Regulation Of Transforming Growth Factor Beta Production | 4 | 12 | 8.39E-04 | 0.019 | | TGFB2, GATA6, THBS1, SERPINB7 |  |
| Cellular Response To Stimulus | 71 | 1 | 8.39E-04 | 0.010 | | PLK2, ATP2B4, BCL2A1, ABCA1, TSPYL5, CD68, FBXO32, CLU, BMP4, IL24, CCND2, TRIM6, CPT1A, PLCB1, PTPN22, LPAR1, EPHA3, EYA4, F3, FBP1, TNFRSF21, PDCD4, GRB14, HAS2, HLA-A, GATA6, GBP1, GBP2, GBP3, GJA1, IL1B, IL13RA2, INHBB, ITGB3, HMGCS1, HES1, MEF2C, MAP3K1, MMP3, MMP1, KCNQ3, SNX10, LTBP1, BCL11A, GDAP1, PDE3B, PIK3CG, PLAU, SCN5A, SCP2, CCL2, SOX4, SNAI2, VEGFA, WT1, THBS1, TIMP3, TLR3, HIST2H4B, SRPX, LAPTM5, CXCR4, NRP2, CPEB4, CRADD, ADAMTS12, IL1RL1, NOG, RAD50, OPTN, NUAK1 |  |
| Regulation Of Interleukin-6 Production | 8 | 5 | 8.43E-04 | 0.017 | | ARRB1, NCKAP1L, AFAP1L2, PTPN22, IL1B, TLR3, F2RL1, IRAK3 |  |
| Positive Regulation Of Leukocyte Migration | 8 | 5 | 8.46E-04 | 0.016 | | NCKAP1L, CSF1, IL1B, VEGFA, ITGB3, CCL2, THBS1, F2RL1 |  |
| Negative Regulation Of Establishment Of Protein Localization | 10 | 4 | 8.46E-04 | 0.016 | | TNFRSF21, PKIA, PDE3B, GBP1, PTPN22, IL1B, CARD8, INHBB, F2RL1, NEO1 |  |
| Response To Interferon-Beta | 5 | 8 | 8.51E-04 | 0.017 | | TRIM6, GBP1, GBP2, GBP3, TLR3 |  |
| Positive Regulation Of Protein Secretion | 12 | 3 | 8.51E-04 | 0.012 | | SOX4, ARRB1, TRIM6, IL1RL1, GJA1, CYP2J2, PTPN22, IL1B, CARD8, TGFB2, CASK, F2RL1 |  |
| Pulmonary Valve Morphogenesis | 3 | 20 | 8.67E-04 | 0.018 | | ROBO1, BMP4, TGFB2 |  |
| Endocardial Cushion Development | 3 | 20 | 8.72E-04 | 0.008 | | BMP4, THBS1, EPHA3 |  |
| Cardiac Right Ventricle Morphogenesis | 3 | 20 | 8.72E-04 | 0.008 | | SOX4, BMP4, TGFB2 |  |
| Negative Regulation Of Keratinocyte Proliferation | 3 | 20 | 8.72E-04 | 0.008 | | EFNB2, CASK, SNAI2 |  |
| Regulation Of Glial Cell Differentiation | 6 | 6 | 8.72E-04 | 0.008 | | TNFRSF21, NOG, CXCR4, IL1B, BMP4, HES1 |  |
| Cellular Response To Platelet-Derived Growth Factor Stimulus | 4 | 11 | 8.86E-04 | 0.013 | | HAS2, ITGB3, SNAI2, LTBP1 |  |
| Negative Regulation Of Glomerular Mesangial Cell Proliferation | 2 | 50 | 9.41E-04 | 0.010 | | WT1, BMP4 |  |
| Positive Regulation Of Glomerular Filtration | 2 | 50 | 9.86E-04 | 0.005 | | GJA1, F2RL1 |  |
| Regulation Of Hepatocyte Growth Factor Receptor Signaling Pathway | 2 | 50 | 9.86E-04 | 0.005 | | ADAMTS12, ESM1 |  |
| Negative Regulation Of Cgmp-Mediated Signaling | 2 | 50 | 9.86E-04 | 0.005 | | PDE10A, THBS1 |  |
| Negative Regulation Of Nitric Oxide Mediated Signal Transduction | 2 | 50 | 9.86E-04 | 0.005 | | ATP2B4, THBS1 |  |
| Negative Regulation Of Glomerulus Development | 2 | 50 | 9.86E-04 | 0.005 | | WT1, BMP4 |  |
| Positive Regulation Of Ovulation | 2 | 50 | 9.86E-04 | 0.005 | | INHBB, PLAU |  |
| Epithelial Cell Proliferation Involved In Renal Tubule Morphogenesis | 2 | 50 | 9.86E-04 | 0.005 | | MEF2C, LGR5 |  |
| Negative Regulation Of Branching Morphogenesis Of A Nerve | 2 | 50 | 9.86E-04 | 0.005 | | BCL11A, IL1B |  |
| Regulation Of Immune System Process | 42 | 2 | 9.86E-04 | 0.005 | | BCL2A1, MYL9, CD33, CLU, CCL2, HMSD, BMP4, PTPRS, C4BPB, IRAK3, ZMIZ1, MTUS1, ZNF608, TRIM6, PIK3AP1, CSF1, PLCB1, PTPN22, SAMSN1, VEGFA, EFNB2, TGFB2, THBS1, SLAMF7, TLR3, F2RL1, TNFRSF21, HIST2H4B, NCKAP1L, HLA-A, HLA-B, GBP1, IL1B, IL13RA2, ITGB3, HLA-DRA, HES1, MEF2C, MAP3K1, IL1RL1, PLPP4, CLEC2B |  |
| Regulation Of Lipid Catabolic Process | 5 | 8 | 9.94E-04 | 0.018 | | IL1B, AADAC, PDE3B, CPT1A, PIK3CG |  |
| Inflammatory Response | 17 | 3 | 9.96E-04 | 0.012 | | GJA1, CXCR4, PIK3CG, IGFBP4, ADGRE5, IL1B, SCN9A, CCL2, PTGES, CSF1, TSPAN2, AFAP1L2, S1PR3, THBS1, EPHA3, TLR3, F2RL1 |  |
| Myeloid Leukocyte Differentiation | 7 | 5 | 1.01E-03 | 0.019 | | MEF2C, SNX10, CSF1, PLCB1, VEGFA, BMP4, F2RL1 |  |
| Cellular Divalent Inorganic Cation Homeostasis | 17 | 3 | 1.02E-03 | 0.015 | | ATP2B4, GJA1, CXCR4, PIK3CG, EFHC1, IL1B, ITGB3, CCL2, GPR65, BMP4, CEMIP, KISS1, LPAR1, S1PR3, TGM2, F2RL1, F2RL2 |  |
| Positive Regulation Of Angiogenesis | 10 | 4 | 1.03E-03 | 0.019 | | PLK2, MIR181B1, GATA6, CXCR4, IL1B, VEGFA, ITGB3, THBS1, TLR3, F3 |  |
| Regulation Of Leukocyte Proliferation | 11 | 3 | 1.04E-03 | 0.017 | | TNFRSF21, MEF2C, NCKAP1L, HLA-A, MIR181B1, CSF1, PTPN22, IL1B, CCL2, BMP4, HES1 |  |
| Positive Regulation Of Cellular Metabolic Process | 69 | 1 | 1.04E-03 | 0.017 | | PLK2, ARRB1, ATP2B4, ARRDC4, MIR181B1, AADAC, TSPYL5, CARD8, CLU, BMP4, IL24, CCND2, ARL2BP, TRIM6, CPT1A, CSF1, PLCB1, FGF13, PTPN22, LPAR1, EPHA7, EPHA3, EYA4, F2RL1, F3, ACSL1, HAS2, NCKAP1L, GATA6, PRICKLE1, IGFBP4, IL1B, INHBB, TRIB2, ITGB3, FOXA1, HES1, CDON, MEF2C, MAP3K1, NRK, KISS1, ZNF438, PIK3CG, PLAU, ROBO1, CCL2, SOX4, ZMIZ1, CEMIP, TCF4, PRDM9, SNAI2, VEGFA, WT1, TGFA, TGFB2, THBS1, TLR3, CXCR4, CASK, SAMD5, TNFSF10, CRADD, PDLIM1, NOG, RAD50, AFAP1L2, OPTN |  |
| B Cell Homeostasis | 4 | 11 | 1.05E-03 | 0.017 | | MEF2C, NCKAP1L, BCL2A1, DOCK10 |  |
| Response To Platelet-Derived Growth Factor | 4 | 11 | 1.05E-03 | 0.010 | | HAS2, ITGB3, SNAI2, LTBP1 |  |
| Regulation Of Biological Quality | 79 | 1 | 1.05E-03 | 0.010 | | PLK2, ARRB1, ATP2B4, BCL2A1, MIR181B1, ABCA1, EFHC1, SLITRK1, CHRM3, CLU, CLIC2, BMP4, C4BPB, DMD, ARL2BP, DSP, TRIM6, CPT1A, CSF1, CST4, FGF13, FLG, LPAR1, S1PR3, EPHA3, F2RL1, F3, F2RL2, F13A1, HAS2, NCKAP1L, GATA6, GJA1, IL1B, INHBB, ITGB3, FOXA1, MEF2C, MAP3K1, KCNQ3, SNX10, KISS1, LTBP1, SERPINB2, NEO1, HECTD4, PDE3B, PIK3CG, PLAU, PLXNA2, PLXNA1, MCTP2, BAIAP2L1, SCN5A, SCP2, SCN9A, CCL2, ACOXL, DOCK10, SOX4, ATP8B2, CEMIP, HEG1, TBXAS1, SLC22A3, VEGFA, NXN, TGFB2, THBS1, TGFBR3, TGM2, ILDR2, HIST2H4B, CXCR4, GPR65, CASK, GMFG, LIPG, RAD50 |  |
| Cellular Response To Parathyroid Hormone Stimulus | 3 | 18 | 1.06E-03 | 0.017 | | MEF2C, GJA1, LTBP1 |  |
| Negative Regulation Of Dendritic Spine Development | 3 | 18 | 1.06E-03 | 0.008 | | PLK2, PTPRS, ASAP1 |  |
| Positive Regulation Of Nitrogen Compound Metabolic Process | 67 | 1 | 1.06E-03 | 0.008 | | PLK2, ARRB1, ATP2B4, ARRDC4, MIR181B1, TSPYL5, CARD8, CLU, BMP4, IL24, C4BPB, CCND2, ARL2BP, TRIM6, CSF1, PLCB1, FGF13, PTPN22, LPAR1, EPHA7, EPHA3, EYA4, F2RL1, F3, ACSL1, HAS2, NCKAP1L, GATA6, PRICKLE1, GJA1, IGFBP4, IL1B, INHBB, TRIB2, ITGB3, FOXA1, HES1, CDON, MEF2C, MAP3K1, NRK, KISS1, ZNF438, PIK3CG, ROBO1, CCL2, SOX4, ZMIZ1, CEMIP, TCF4, PRDM9, SNAI2, VEGFA, WT1, TGFA, TGFB2, THBS1, TLR3, CXCR4, CASK, SAMD5, TNFSF10, CRADD, PDLIM1, NOG, RAD50, AFAP1L2 |  |
| Regulation Of Body Fluid Levels | 16 | 3 | 1.06E-03 | 0.017 | | HAS2, GATA6, GJA1, PLAU, CHRM3, ITGB3, C4BPB, HEG1, FLG, VEGFA, SERPINB2, THBS1, F2RL1, F3, F2RL2, F13A1 |  |
| Positive Regulation Of Cell Morphogenesis Involved In Differentiation | 9 | 4 | 1.11E-03 | 0.019 | | HAS2, PLXNA2, PLXNA1, ROBO1, BCL11A, SLITRK1, VEGFA, ITGB3, CASK |  |
| Regulation Of Gtpase Activity | 16 | 3 | 1.12E-03 | 0.016 | | ARHGDIB, ARRB1, NCKAP1L, EVI5, PLXNA2, PLXNA1, CCL2, GPR65, DOCK10, RGS10, PLCB1, TBC1D30, ASAP1, TIAM2, EPHA3, F2RL1 |  |
| Response To External Stimulus | 50 | 2 | 1.15E-03 | 0.019 | | ARRB1, ABCA1, PLAU, PLCB4, BAIAP2L1, CLU, CCL2, BMP4, IFI44L, IL24, IRAK3, CYP24A1, DMD, TRIM6, CTSD, PTPN22, VEGFA, TGFB2, THBS1, TIMP3, EPHA3, TLR3, F2RL1, F3, ACSL1, PDCD4, HLA-A, GBP1, GBP2, GBP3, GJA1, CXCR4, GNGT2, IL1B, IL13RA2, INHBB, ITGB3, CPEB4, HMGCS1, CRADD, PTGES, MEF2C, MAP3K1, MMP3, MMP1, LIPG, LTBP1, BCL11A, OPTN, GDAP1 |  |
| Regulation Of Keratinocyte Proliferation | 4 | 11 | 1.16E-03 | 0.018 | | HAS2, EFNB2, CASK, SNAI2 |  |
| Cell Proliferation | 22 | 2 | 1.17E-03 | 0.010 | | EVI5, CXCR4, PIK3CG, IGFBP4, CHRM3, CLU, FAT4, BMP4, LGR5, SOX4, MEF2C, SAMD9L, CSF1, LIPG, FGF13, VEGFA, HHIP, TGFA, TGFB2, TGFBR3, F2RL1, FAM83B |  |
| Regulation Of Wound Healing | 8 | 4 | 1.22E-03 | 0.019 | | GJA1, CXCR4, PLAU, SERPINB2, THBS1, CASK, F2RL1, F3 |  |
| Negative Regulation Of Secretion | 11 | 3 | 1.23E-03 | 0.015 | | TNFRSF21, PDE3B, GBP1, PTPN22, IL1B, IL13RA2, CARD8, INHBB, STXBP6, F2RL1, NEO1 |  |
| Lipid Metabolic Process | 32 | 2 | 1.23E-03 | 0.017 | | PON2, LPCAT2, AADAC, ABCA1, PIK3CG, PLCB4, PLTP, PTGR1, CLU, SCP2, ACOXL, HRASLS, CYP24A1, SLC44A2, TBXAS1, SDC2, PIK3AP1, CPT1A, PLCB1, CYP2J2, UGCG, ACSL1, SERINC2, GALC, GATA6, ELOVL7, HMGCS1, AGMO, PTGES, B4GALT6, LIPG, PLPP4 |  |
| Epithelial Tube Morphogenesis | 7 | 5 | 1.24E-03 | 0.019 | | MEF2C, GJA1, CXCR4, BMP4, EFNB2, EPHA7, HES1 |  |
| Negative Regulation Of Extrinsic Apoptotic Signaling Pathway | 7 | 5 | 1.24E-03 | 0.014 | | SNAI2, IL1B, VEGFA, BMP4, THBS1, EYA4, TNFSF10 |  |
| Cell Migration Involved In Sprouting Angiogenesis | 3 | 17 | 1.24E-03 | 0.014 | | ROBO1, VEGFA, EFNB2 |  |
| Regulation Of Cd8-Positive, Alpha-Beta T Cell Activation | 3 | 17 | 1.28E-03 | 0.008 | | PTPN22, NCKAP1L, HLA-A |  |
| Response To Sterol | 4 | 10 | 1.28E-03 | 0.008 | | INHBB, SCP2, ABCA1, HMGCS1 |  |
| Negative Regulation Of Catalytic Activity | 25 | 2 | 1.30E-03 | 0.010 | | PDCD4, PCDH11X, ARRB1, PKIA, ATP2B4, NCKAP1L, BCL2A1, IL1B, CARD8, TRIB2, GPRC5A, HMSD, BMP4, IL24, CST7, SH3RF2, SERPINB7, IRAK3, GMFG, PTPN22, VEGFA, SERPINB2, TGFB2, THBS1, TIMP3 |  |
| Regulation Of Multi-Organism Process | 15 | 3 | 1.32E-03 | 0.019 | | GRB14, ARHGDIB, MAP3K1, TRIM6, PLCB1, CXCR4, PLAU, RAD50, OPTN, PTPN22, IL1B, CARD8, INHBB, STOM, F2RL1 |  |
| Regulation Of Dendrite Development | 9 | 4 | 1.33E-03 | 0.018 | | PLK2, MEF2C, SDC2, ASAP1, ROBO1, BCL11A, LPAR1, PTPRS, CASK |  |
| Positive Regulation Of Epithelial Cell Proliferation | 10 | 4 | 1.33E-03 | 0.016 | | HAS2, NOG, NRP2, VEGFA, ITGB3, SCN5A, BMP4, TGFA, F3, CCND2 |  |
| Regulation Of Growth | 23 | 2 | 1.33E-03 | 0.017 | | GATA6, TSPYL5, GJA1, CXCR4, IGFBP4, BMP4, PTPRS, ESM1, HRASLS, MEF2C, DPYSL2, NRK, NOG, CSF1, PLCB1, FGF13, BCL11A, VEGFA, WT1, TGFB2, TGFBR3, EPHA7, FBP1 |  |
| Extracellular Matrix Disassembly | 5 | 7 | 1.33E-03 | 0.019 | | MMP3, MMP1, BMP1, TLL1, LCP1 |  |
| Regulation Of Cardiac Muscle Tissue Development | 7 | 5 | 1.34E-03 | 0.012 | | MEF2C, GATA6, NOG, GJA1, BMP4, EFNB2, TGFBR3 |  |
| Multicellular Organismal Process | 99 | 1 | 1.36E-03 | 0.014 | | ARHGDIB, PLK2, ARRB1, ABCA1, MYL9, CDH11, SLITRK1, FBXO32, CHRM3, BMP4, BMP1, C4BPB, CCND2, TDRD7, DMD, DPYSL2, OR2M3, DSP, OR5P2, CSF1, PLCB1, CTSD, CYP2J2, FGF13, FLG, OR2M4, S1PR3, FLRT2, EFNB2, EPHA7, EPHA3, EYA4, F2RL1, F3, F2RL2, F13A1, EFEMP1, PCDH17, HAS2, HLA-A, OR2H1, GATA6, GJA1, IL1B, INHBB, ITGB3, HLA-DRA, FOXA1, SHISA3, HES1, CDON, MEF2C, MMP3, NRK, SNX10, KCNIP3, MPZL3, BCL11A, SHC3, CNTN5, KRTAP4-8, OR5B21, PIK3CG, PLAU, ADGRF1, PLXNA2, PLTP, PLXNA1, ROBO1, MCTP2, SCN5A, SCN9A, LAMA1, SOX4, ZMIZ1, CEMIP, HEG1, TBXAS1, TCF4, SNAI2, VEGFA, WT1, NXN, HHIP, TGFB2, TGFBR3, TGM2, TIMP3, TLL1, SLC7A5, SHISA2, EVI5, CXCR4, NRP2, GPR65, FAT4, LGR5, NOG, LIPG |  |
| Positive Regulation Of Defense Response | 15 | 3 | 1.36E-03 | 0.016 | | PDCD4, MEF2C, MAP3K1, TRIM6, IL1RL1, PIK3AP1, GJA1, PIK3CG, OPTN, PTPN22, IL1B, TGM2, TLR3, F2RL1, IRAK3 |  |
| Vasculogenesis | 6 | 6 | 1.39E-03 | 0.018 | | ZMIZ1, HEG1, HAS2, VEGFA, WT1, TGFBR3 |  |
| Muscle Tissue Morphogenesis | 6 | 6 | 1.41E-03 | 0.013 | | HEG1, DSP, NOG, BMP4, TGFB2, TGFBR3 |  |
| Regulation Of Cysteine-Type Endopeptidase Activity | 11 | 3 | 1.41E-03 | 0.013 | | ARRB1, BCL2A1, ROBO1, CARD8, VEGFA, IL24, THBS1, EPHA7, F3, TNFSF10, CRADD |  |
| Regulation Of Organelle Organization | 33 | 2 | 1.42E-03 | 0.017 | | ARHGDIB, PLK2, ARRB1, NAV3, BAIAP2L1, CLU, BMP4, CCND2, PLCB1, PRDM9, SNAI2, TBC1D30, FGF13, VEGFA, LPAR1, TGFA, TGFB2, EPHA3, F2RL1, NCKAP1L, EVI5, IL1B, ITGB3, STXBP6, GPR65, TNFSF10, GMFG, MEF2C, MAP3K1, LCP1, ASAP1, RAD50, OPTN |  |
| Regulation Of Endothelial Cell Proliferation | 9 | 4 | 1.42E-03 | 0.018 | | MEF2C, GJA1, NRP2, VEGFA, ITGB3, CCL2, BMP4, THBS1, F3 |  |
| Negative Regulation Of Dendrite Development | 4 | 10 | 1.42E-03 | 0.016 | | BCL11A, PLK2, PTPRS, ASAP1 |  |
| Pituitary Gland Development | 4 | 10 | 1.44E-03 | 0.010 | | INHBB, BMP4, NOG, HES1 |  |
| Cellular Response To Gonadotropin Stimulus | 4 | 10 | 1.44E-03 | 0.010 | | WT1, HMGCS1, GATA6, EPHA3 |  |
| Response To Lipopolysaccharide | 17 | 2 | 1.44E-03 | 0.010 | | PDCD4, ABCA1, GBP2, GJA1, PLAU, IL1B, CCL2, IL24, IRAK3, PTGES, MEF2C, MMP3, MMP1, TRIM6, PTPN22, EPHA3, F3 |  |
| Positive Regulation Of Neuron Projection Development | 13 | 3 | 1.45E-03 | 0.018 | | DMD, CXCR4, PLXNA2, PLXNA1, TENM3, ROBO1, BCL11A, SLITRK1, VEGFA, LPAR1, BMP4, CASK, EPHA3 |  |
| Regulation Of Dendritic Spine Development | 6 | 6 | 1.47E-03 | 0.018 | | PLK2, MEF2C, ASAP1, LPAR1, PTPRS, CASK |  |
| Reverse Cholesterol Transport | 3 | 16 | 1.49E-03 | 0.013 | | CLU, ABCA1, LIPG |  |
| Positive Regulation Of Macromolecule Metabolic Process | 69 | 1 | 1.52E-03 | 0.008 | | PLK2, ARRB1, ATP2B4, ARRDC4, MIR181B1, TSPYL5, CARD8, CLU, BMP4, IL24, C4BPB, CCND2, ARL2BP, TRIM6, CSF1, PLCB1, FGF13, PTPN22, LPAR1, EPHA7, EPHA3, EYA4, F2RL1, F3, ACSL1, HAS2, NCKAP1L, GATA6, PRICKLE1, GJA1, IGFBP4, IL1B, INHBB, TRIB2, ITGB3, FOXA1, HES1, CDON, MEF2C, MAP3K1, NRK, KISS1, BCL11A, ZNF438, PIK3CG, ROBO1, CCL2, SOX4, ZMIZ1, CEMIP, TCF4, PRDM9, SNAI2, VEGFA, WT1, TGFA, TGFB2, THBS1, TLR3, CXCR4, CASK, SAMD5, TNFSF10, CRADD, PDLIM1, NOG, RAD50, AFAP1L2, OPTN |  |
| Glycosaminoglycan Metabolic Process | 8 | 4 | 1.54E-03 | 0.017 | | CEMIP, HAS2, SDC2, MAMDC2, B4GALT6, IL1B, VEGFA, CHST15 |  |
| Positive Regulation Of Leukocyte Chemotaxis | 6 | 6 | 1.55E-03 | 0.015 | | NCKAP1L, CSF1, IL1B, VEGFA, THBS1, F2RL1 |  |
| Cerebral Cortex Development | 6 | 6 | 1.57E-03 | 0.013 | | CDON, BCL2A1, PLCB1, FAT4, PTPRS, CASK |  |
| Positive Regulation Of Organelle Organization | 19 | 2 | 1.57E-03 | 0.013 | | ARRB1, NCKAP1L, NAV3, IL1B, BAIAP2L1, GPR65, TNFSF10, MAP3K1, LCP1, PLCB1, PRDM9, SNAI2, ASAP1, RAD50, OPTN, VEGFA, LPAR1, TGFA, F2RL1 |  |
| Cardiac Chamber Morphogenesis | 4 | 10 | 1.57E-03 | 0.018 | | SOX4, HEG1, BMP4, TGFB2 |  |
| Hyaluronan Metabolic Process | 4 | 10 | 1.59E-03 | 0.010 | | IL1B, CEMIP, VEGFA, HAS2 |  |
| Negative Regulation Of Macrophage Cytokine Production | 2 | 40 | 1.59E-03 | 0.010 | | TGFB2, IRAK3 |  |
| Atrial Septum Primum Morphogenesis | 2 | 40 | 1.63E-03 | 0.005 | | SOX4, TGFB2 |  |
| Cell Adhesion Involved In Heart Morphogenesis | 2 | 40 | 1.63E-03 | 0.005 | | FLRT2, TGFB2 |  |
| Apolipoprotein A-I-Mediated Signaling Pathway | 2 | 40 | 1.63E-03 | 0.005 | | ITGB3, ABCA1 |  |
| Positive Regulation Of Cardioblast Differentiation | 2 | 40 | 1.63E-03 | 0.005 | | TGFB2, GATA6 |  |
| Regulation Of Ovulation | 2 | 40 | 1.63E-03 | 0.005 | | INHBB, PLAU |  |
| Positive Regulation Of Cd8-Positive, Alpha-Beta T Cell Proliferation | 2 | 40 | 1.63E-03 | 0.005 | | PTPN22, HLA-A |  |
| Regulation Of Pathway-Restricted Smad Protein Phosphorylation | 5 | 7 | 1.63E-03 | 0.005 | | INHBB, BMP4, TGFB2, NOG, PMEPA1 |  |
| Regulation Of Myoblast Differentiation | 5 | 7 | 1.65E-03 | 0.012 | | CDON, MEF2C, BMP4, PRICKLE1, PLCB1 |  |
| Negative Regulation Of Response To Wounding | 6 | 6 | 1.65E-03 | 0.012 | | GJA1, PLAU, SERPINB2, PTPRS, CASK, NEO1 |  |
| Cellular Response To Organonitrogen Compound | 19 | 2 | 1.66E-03 | 0.013 | | GRB14, ATP2B4, PDE3B, PIK3CG, PLAU, INHBB, ITGB3, CPEB4, CCND2, MEF2C, MMP3, MMP1, IL1RL1, PLCB1, BCL11A, OPTN, PTPN22, WT1, FBP1 |  |
| Response To Fatty Acid | 7 | 5 | 1.68E-03 | 0.018 | | TBXAS1, CPT1A, PLCB1, IL1B, TGFBR3, HES1, ACSL1 |  |
| Positive Regulation Of Endothelial Cell Migration | 7 | 5 | 1.69E-03 | 0.014 | | PLK2, SNAI2, NRP2, VEGFA, ITGB3, BMP4, THBS1 |  |
| Metal Ion Homeostasis | 20 | 2 | 1.69E-03 | 0.014 | | ATP2B4, GJA1, CXCR4, PIK3CG, EFHC1, IL1B, ITGB3, SCN5A, CCL2, GPR65, BMP4, CEMIP, SNX10, KISS1, LPAR1, S1PR3, TGM2, F2RL1, F2RL2, NEO1 |  |
| Regulation Of Peptidyl-Tyrosine Phosphorylation | 11 | 3 | 1.71E-03 | 0.018 | | ARL2BP, AFAP1L2, PTPN22, SAMSN1, VEGFA, ITGB3, GPRC5A, IL24, TGFA, EPHA7, HES1 |  |
| Cellular Protein Modification Process | 63 | 1 | 1.71E-03 | 0.017 | | PLK2, ARRB1, ARRDC4, ABCA1, MGAT4A, FBXO32, MARCH3, CHRM3, BMP4, IL24, PRSS23, IRAK3, DSP, TRIM6, CSF1, FGF13, FLG, PTPN22, EFNB2, EPHA7, EPHA3, EYA4, F13A1, EFEMP1, TTLL11, IGFBP4, IL1B, ZDHHC15, TRIB2, SH3RF2, MEF2C, MAP3K1, CPA4, NRK, LTBP1, BCL11A, SHC3, HECTD4, MAMDC2, PIK3CG, KLHL4, CCL2, LAMA1, PTPRS, CPPED1, MARCH4, SDC2, PRDM9, PRDM8, FKBP10, TGFB2, THBS1, TGFBR3, TGM2, TLR3, PORCN, CDK15, CDC42BPA, CASK, GMFG, B4GALT6, CTDSPL, NUAK1 |  |
| Protein Modification Process | 63 | 1 | 1.73E-03 | 0.017 | | PLK2, ARRB1, ARRDC4, ABCA1, MGAT4A, FBXO32, MARCH3, CHRM3, BMP4, IL24, PRSS23, IRAK3, DSP, TRIM6, CSF1, FGF13, FLG, PTPN22, EFNB2, EPHA7, EPHA3, EYA4, F13A1, EFEMP1, TTLL11, IGFBP4, IL1B, ZDHHC15, TRIB2, SH3RF2, MEF2C, MAP3K1, CPA4, NRK, LTBP1, BCL11A, SHC3, HECTD4, MAMDC2, PIK3CG, KLHL4, CCL2, LAMA1, PTPRS, CPPED1, MARCH4, SDC2, PRDM9, PRDM8, FKBP10, TGFB2, THBS1, TGFBR3, TGM2, TLR3, PORCN, CDK15, CDC42BPA, CASK, GMFG, B4GALT6, CTDSPL, NUAK1 |  |
| Regulation Of Sodium Ion Transmembrane Transport | 5 | 7 | 1.73E-03 | 0.017 | | DMD, ATP2B4, SCN5A, STOM, PLCB1 |  |
| Ion Homeostasis | 23 | 2 | 1.77E-03 | 0.012 | | ATP2B4, ABCA1, GJA1, CXCR4, PIK3CG, EFHC1, IL1B, ITGB3, SCN5A, CCL2, GPR65, BMP4, CEMIP, TBXAS1, SNX10, KISS1, LIPG, LPAR1, S1PR3, TGM2, F2RL1, F2RL2, NEO1 |  |
| Neural Retina Development | 3 | 15 | 1.77E-03 | 0.018 | | OPTN, ATP2B4, TGFB2 |  |
| G-Protein Coupled Acetylcholine Receptor Signaling Pathway | 3 | 15 | 1.79E-03 | 0.008 | | CHRM3, PLCB1, RGS10 |  |
| Regulation Of Apoptotic Process Involved In Development | 3 | 15 | 1.79E-03 | 0.008 | | WT1, TGFB2, TGFBR3 |  |
| Positive Regulation Of Cellular Component Biogenesis | 17 | 2 | 1.79E-03 | 0.008 | | NCKAP1L, NAV3, ADGRE5, SLITRK1, BAIAP2L1, CLU, GPR65, MAP3K1, MMP3, MMP1, LCP1, ADGRL3, ASAP1, VEGFA, LPAR1, FLRT2, F2RL1 |  |
| Positive Regulation Of Apoptotic Signaling Pathway | 9 | 4 | 1.90E-03 | 0.018 | | SRPX, G0S2, INHBB, CLU, TGFB2, THBS1, TIMP3, TNFSF10, CRADD |  |
| Positive Regulation Of Peptidyl-Lysine Acetylation | 4 | 9 | 1.91E-03 | 0.016 | | SOX4, IL1B, ARRB1, SNAI2 |  |
| Regulation Of Neuron Death | 13 | 3 | 1.91E-03 | 0.010 | | ARRB1, MEF2C, KCNIP3, OPTN, IL1B, VEGFA, CPEB4, CLU, CCL2, EFNB2, TGFB2, EPHA7, NEO1 |  |
| Cellular Response To Cytokine Stimulus | 21 | 2 | 1.93E-03 | 0.017 | | TNFRSF21, HAS2, HLA-A, GBP1, GBP2, GBP3, LAPTM5, CXCR4, NRP2, INHBB, CCL2, IL24, HES1, ADAMTS12, MMP3, MMP1, TRIM6, SNX10, OPTN, THBS1, TLR3 |  |
| Response To Alkaloid | 8 | 4 | 1.93E-03 | 0.018 | | MMP1, DPYSL2, SDC2, CXCR4, PLAU, ADGRL3, IL1B, HES1 |  |
| Regulation Of Synapse Assembly | 6 | 5 | 1.93E-03 | 0.015 | | MEF2C, ADGRL3, ADGRE5, SLITRK1, FLRT2, EPHA7 |  |
| Cell Development | 24 | 2 | 1.93E-03 | 0.013 | | NCKAP1L, GATA6, CXCR4, TENM3, ROBO1, INHBB, BMP4, PTPRS, HES1, SOX4, TDRD7, MEF2C, DMD, HEG1, KCNQ3, PRDM8, SNAI2, TSPAN2, VEGFA, WT1, LPAR1, TGFB2, TGFBR3, F2RL1 |  |
| Epithelium Development | 9 | 4 | 1.96E-03 | 0.018 | | HEG1, NOG, GJA1, SNAI2, PLXNA2, WT1, BMP4, FOXA1, HES1 |  |
| Cellular Component Morphogenesis | 17 | 2 | 1.98E-03 | 0.016 | | NCKAP1L, GJA1, POF1B, CDH11, SLITRK1, ITGB3, CLU, HES1, DOCK10, MEF2C, MAP3K1, HEG1, NRK, SDC2, PRDM8, PARD6B, TGFB2 |  |
| Glycosaminoglycan Biosynthetic Process | 6 | 5 | 2.01E-03 | 0.018 | | CEMIP, HAS2, SDC2, B4GALT6, IL1B, CHST15 |  |
| Cell Projection Morphogenesis | 11 | 3 | 2.03E-03 | 0.013 | | NRK, NCKAP1L, SDC2, GJA1, PRDM8, PARD6B, CDH11, SLITRK1, ITGB3, CLU, DOCK10 |  |
| Phosphorylation | 30 | 2 | 2.05E-03 | 0.017 | | PLK2, PIK3CG, AK4, PLAU, CCL2, LAMA1, BMP4, IL24, IRAK3, PIK3AP1, FGF13, EFNB2, TGFB2, TGFBR3, EPHA7, EPHA3, TLR3, EFEMP1, CDK15, IL1B, TRIB2, CDC42BPA, CASK, GMFG, MEF2C, MAP3K1, NRK, RAD50, SHC3, NUAK1 |  |
| Regulation Of Blood Circulation | 12 | 3 | 2.06E-03 | 0.018 | | DMD, ATP2B4, DSP, TBXAS1, GJA1, PIK3CG, CYP2J2, CHRM3, CLIC2, SCN5A, TGFB2, F2RL1 |  |
| Myeloid Leukocyte Activation | 17 | 2 | 2.07E-03 | 0.017 | | NCKAP1L, PIK3CG, PLAU, CD33, CD68, ADGRE5, CLU, CPPED1, SLC44A2, GMFG, CRISPLD2, DSP, CSF1, CTSD, STOM, TLR3, F2RL1 |  |
| Epithelial Cell Differentiation Involved In Kidney Development | 3 | 15 | 2.08E-03 | 0.018 | | MEF2C, WT1, BMP4 |  |
| Regulation Of Lipid Transport | 7 | 5 | 2.08E-03 | 0.008 | | ABCA1, PLTP, LIPG, IL1B, ITGB3, SCP2, THBS1 |  |
| Endothelial Cell Development | 4 | 9 | 2.09E-03 | 0.014 | | DMD, HEG1, PTPRS, F2RL1 |  |
| Regulation Of Extracellular Matrix Organization | 4 | 9 | 2.10E-03 | 0.010 | | HAS2, ITGB3, TGFB2, COLGALT1 |  |
| Regulation Of Angiogenesis | 14 | 3 | 2.10E-03 | 0.010 | | PLK2, TCF4, MIR181B1, PDE3B, GATA6, CXCR4, IL1B, VEGFA, ITGB3, HHIP, TGFB2, THBS1, TLR3, F3 |  |
| Response To Estradiol | 10 | 3 | 2.13E-03 | 0.018 | | MMP3, GJA1, IL1B, VEGFA, SCP2, BMP4, FOXA1, TGFB2, F3, CCND2 |  |
| Response To Molecule Of Bacterial Origin | 17 | 2 | 2.19E-03 | 0.016 | | PDCD4, ABCA1, GBP2, GJA1, PLAU, IL1B, CCL2, IL24, IRAK3, PTGES, MEF2C, MMP3, MMP1, TRIM6, PTPN22, EPHA3, F3 |  |
| Regulation Of Actin Filament-Based Process | 14 | 3 | 2.19E-03 | 0.018 | | GMFG, ARHGDIB, MEF2C, MAP3K1, DSP, NCKAP1L, ITGB3, BAIAP2L1, SCN5A, LPAR1, GPR65, TGFB2, EPHA3, F2RL1 |  |
| Regulation Of Leukocyte Cell-Cell Adhesion | 12 | 3 | 2.22E-03 | 0.018 | | TNFRSF21, ZMIZ1, HAS2, ZNF608, NCKAP1L, HLA-A, PTPN22, IL1B, CCL2, BMP4, EFNB2, HES1 |  |
| Positive Regulation Of Cell-Substrate Adhesion | 7 | 5 | 2.27E-03 | 0.017 | | DMD, HAS2, CSF1, VEGFA, ITGB3, THBS1, ABI3BP |  |
| Regulation Of Cardiac Muscle Cell Proliferation | 5 | 7 | 2.27E-03 | 0.014 | | MEF2C, GATA6, TGFBR3, NOG, GJA1 |  |
| Artery Development | 4 | 9 | 2.28E-03 | 0.012 | | ROBO1, PRICKLE1, CXCR4, LTBP1 |  |
| Face Morphogenesis | 4 | 9 | 2.29E-03 | 0.010 | | CRISPLD1, CRISPLD2, TGFB2, NOG |  |
| Response To Retinoic Acid | 8 | 4 | 2.29E-03 | 0.010 | | PTGES, MEF2C, ABCA1, GJA1, GDAP1, BMP4, TGFB2, EPHA3 |  |
| Positive Regulation Of Supramolecular Fiber Organization | 9 | 4 | 2.31E-03 | 0.015 | | MAP3K1, NCKAP1L, NAV3, BAIAP2L1, CLU, LPAR1, GPR65, COLGALT1, F2RL1 |  |
| G-Protein Coupled Receptor Signaling Pathway, Coupled To Cyclic Nucleotide Second Messenger | 10 | 3 | 2.31E-03 | 0.016 | | GPR1, ARRB1, ABCA1, ADGRF1, CHRM3, CCL2, LPAR1, S1PR3, GPR65, LGR5 |  |
| Regulation Of Bmp Signaling Pathway | 6 | 5 | 2.31E-03 | 0.016 | | GATA6, NOG, BMP4, TGFBR3, HES1, NEO1 |  |
| Extrinsic Apoptotic Signaling Pathway | 6 | 5 | 2.36E-03 | 0.013 | | BCL2A1, G0S2, IL1B, TGFB2, TLR3, CRADD |  |
| Cellular Lipid Metabolic Process | 26 | 2 | 2.36E-03 | 0.013 | | PON2, LPCAT2, GALC, GATA6, PIK3CG, ELOVL7, PTGR1, SCP2, ACOXL, HMGCS1, HRASLS, AGMO, SLC44A2, PTGES, TBXAS1, SDC2, PIK3AP1, CPT1A, PLCB1, B4GALT6, LIPG, PLPP4, CYP2J2, UGCG, ACSL1, SERINC2 |  |
| Regulation Of Insulin Secretion | 9 | 4 | 2.38E-03 | 0.018 | | SOX4, ARRB1, ARL2BP, PDE3B, CPT1A, GJA1, IL1B, INHBB, CASK |  |
| Negative Regulation Of Vasculature Development | 9 | 4 | 2.38E-03 | 0.016 | | PDCD4, PLK2, TCF4, PDE3B, WT1, HHIP, BMP4, TGFB2, THBS1 |  |
| Positive Regulation Of Leukocyte Proliferation | 8 | 4 | 2.38E-03 | 0.016 | | MEF2C, NCKAP1L, HLA-A, MIR181B1, CSF1, PTPN22, IL1B, HES1 |  |
| Positive Regulation Of Fibroblast Migration | 3 | 14 | 2.39E-03 | 0.015 | | MMP1, ITGB3, THBS1 |  |
| Cellular Response To Vitamin D | 3 | 14 | 2.41E-03 | 0.008 | | MMP1, GDAP1, LTBP1 |  |
| Positive Regulation Of Cell Adhesion Mediated By Integrin | 3 | 14 | 2.41E-03 | 0.008 | | ITGB3, NCKAP1L, TGFB2 |  |
| Fever Generation | 2 | 33 | 2.41E-03 | 0.008 | | IL1B, EPHA3 |  |
| Muscular Septum Morphogenesis | 2 | 33 | 2.42E-03 | 0.005 | | BMP4, TGFBR3 |  |
| Negative Regulation Of Cell Proliferation Involved In Kidney Development | 2 | 33 | 2.42E-03 | 0.005 | | WT1, BMP4 |  |
| Bmp Signaling Pathway Involved In Heart Development | 2 | 33 | 2.42E-03 | 0.005 | | BMP4, NOG |  |
| Positive Regulation Of Transforming Growth Factor Beta1 Production | 2 | 33 | 2.42E-03 | 0.005 | | THBS1, SERPINB7 |  |
| Interferon-Gamma-Mediated Signaling Pathway | 5 | 6 | 2.42E-03 | 0.005 | | HLA-A, HLA-B, HLA-DRA, GBP1, GBP2 |  |
| Positive Regulation Of Lipid Transport | 5 | 6 | 2.43E-03 | 0.012 | | IL1B, SCP2, ABCA1, PLTP, LIPG |  |
| Regulation Of Epithelial Cell Migration | 11 | 3 | 2.43E-03 | 0.012 | | PLK2, MEF2C, HAS2, SNAI2, NRP2, VEGFA, ITGB3, BMP4, TGFB2, THBS1, TGFBR3 |  |
| Negative Regulation Of Developmental Growth | 7 | 4 | 2.45E-03 | 0.017 | | NOG, GJA1, FGF13, BCL11A, BMP4, PTPRS, EPHA7 |  |
| Programmed Cell Death | 24 | 2 | 2.46E-03 | 0.014 | | TNFRSF21, PDCD4, ARRB1, BCL2A1, GJA1, CXCR4, PIK3CG, IL1B, CARD8, GPR65, BMP4, IL24, IRAK3, TNFSF10, CRADD, MEF2C, DSP, KCNIP3, G0S2, FGF13, FLG, WT1, EPHA7, TLR3 |  |
| Cellular Response To Bmp Stimulus | 4 | 9 | 2.48E-03 | 0.018 | | ADAMTS12, BMP4, GATA6, NOG |  |
| Response To Bmp | 4 | 9 | 2.49E-03 | 0.010 | | ADAMTS12, BMP4, GATA6, NOG |  |
| Muscle Adaptation | 4 | 9 | 2.49E-03 | 0.010 | | IL1B, MEF2C, FBXO32, GATA6 |  |
| Regulation Of Vascular Endothelial Growth Factor Receptor Signaling Pathway | 4 | 9 | 2.49E-03 | 0.010 | | IL1B, VEGFA, ITGB3, BMP4 |  |
| Regulation Of Fibroblast Growth Factor Receptor Signaling Pathway | 4 | 9 | 2.49E-03 | 0.010 | | SHISA2, HHIP, THBS1, NOG |  |
| Pancreas Development | 4 | 9 | 2.49E-03 | 0.010 | | ILDR2, TGFB2, GATA6, HES1 |  |
| Positive Regulation Of Epithelial Cell Apoptotic Process | 4 | 9 | 2.49E-03 | 0.010 | | PDCD4, CCL2, BMP4, THBS1 |  |
| Negative Regulation Of Cellular Response To Growth Factor Stimulus | 8 | 4 | 2.49E-03 | 0.010 | | ADAMTS12, SHISA2, NOG, PMEPA1, LTBP1, THBS1, TGFBR3, CASK |  |
| Phospholipid Transport | 5 | 6 | 2.56E-03 | 0.015 | | ATP8B2, PITPNC1, SCP2, ABCA1, PLTP |  |
| Peptide Cross-Linking | 6 | 5 | 2.58E-03 | 0.012 | | DSP, MAMDC2, FLG, THBS1, TGM2, F13A1 |  |
| Positive Regulation Of Axonogenesis | 6 | 5 | 2.59E-03 | 0.013 | | PLXNA2, PLXNA1, ROBO1, BCL11A, SLITRK1, VEGFA |  |
| Negative Regulation Of Cell Migration | 12 | 3 | 2.59E-03 | 0.013 | | ARHGDIB, MEF2C, KISS1, NOG, PLCB1, NAV3, ROBO1, SCP2, CCL2, IL24, THBS1, TGFBR3 |  |
| Epithelial Cell Differentiation | 14 | 3 | 2.61E-03 | 0.017 | | SOX4, TDRD7, MEF2C, DSP, GATA6, CPT1A, CXCR4, FLG, VEGFA, WT1, LAMA1, BMP4, FOXA1, HES1 |  |
| Regulation Of Peptide Hormone Secretion | 10 | 3 | 2.61E-03 | 0.017 | | SOX4, ARRB1, ARL2BP, PDE3B, KISS1, CPT1A, GJA1, IL1B, INHBB, CASK |  |
| Leukocyte Migration | 13 | 3 | 2.65E-03 | 0.016 | | SLC7A5, GRB14, MMP1, NCKAP1L, SDC2, CXCR4, PIK3CG, IL1B, VEGFA, ITGB3, CCL2, TGFB2, F2RL1 |  |
| Regulation Of Animal Organ Formation | 4 | 8 | 2.67E-03 | 0.017 | | ROBO1, WT1, BMP4, HES1 |  |
| Epithelial Cell Proliferation | 6 | 5 | 2.71E-03 | 0.010 | | MEF2C, IGFBP4, VEGFA, BMP4, TGFA, LGR5 |  |
| Mononuclear Cell Differentiation | 3 | 13 | 2.72E-03 | 0.013 | | MEF2C, VEGFA, BMP4 |  |
| Monocyte Differentiation | 3 | 13 | 2.76E-03 | 0.008 | | MEF2C, VEGFA, BMP4 |  |
| Response To Parathyroid Hormone | 3 | 13 | 2.76E-03 | 0.008 | | MEF2C, GJA1, LTBP1 |  |
| Aminoglycan Biosynthetic Process | 6 | 5 | 2.76E-03 | 0.008 | | CEMIP, HAS2, SDC2, B4GALT6, IL1B, CHST15 |  |
| Regulation Of Embryonic Development | 7 | 4 | 2.85E-03 | 0.013 | | NRK, LAMA3, LAMA4, PLCB1, LAMA1, BMP4, HES1 |  |
| Positive Regulation Of Cardiac Muscle Tissue Development | 5 | 6 | 2.88E-03 | 0.014 | | MEF2C, BMP4, EFNB2, GATA6, TGFBR3 |  |
| Response To Decreased Oxygen Levels | 14 | 3 | 2.90E-03 | 0.011 | | SOX4, MMP3, PDLIM1, SDC2, GATA6, NOG, CXCR4, PLAU, IL1B, VEGFA, CPEB4, TGFB2, THBS1, TGFBR3 |  |
| Regulation Of P38Mapk Cascade | 4 | 8 | 2.93E-03 | 0.017 | | PTPN22, IL1B, VEGFA, MIR181B1 |  |
| Regulation Of Oligodendrocyte Differentiation | 4 | 8 | 2.93E-03 | 0.010 | | TNFRSF21, BMP4, HES1, CXCR4 |  |
| Negative Regulation Of Apoptotic Signaling Pathway | 10 | 3 | 2.93E-03 | 0.010 | | BCL2A1, NOG, SNAI2, IL1B, VEGFA, CLU, BMP4, THBS1, EYA4, TNFSF10 |  |
| Granulocyte Chemotaxis | 6 | 5 | 2.95E-03 | 0.016 | | NCKAP1L, PIK3CG, IL1B, VEGFA, CCL2, TGFB2 |  |
| Aminoglycan Metabolic Process | 8 | 4 | 2.98E-03 | 0.013 | | CEMIP, HAS2, SDC2, MAMDC2, B4GALT6, IL1B, VEGFA, CHST15 |  |
| Positive Regulation Of Secretion | 16 | 2 | 3.02E-03 | 0.015 | | ARRB1, GJA1, IL1B, CARD8, INHBB, CASK, RAB27B, SOX4, DPYSL2, TRIM6, IL1RL1, KISS1, CYP2J2, PTPN22, TGFB2, F2RL1 |  |
| Inorganic Ion Homeostasis | 21 | 2 | 3.04E-03 | 0.018 | | ATP2B4, GJA1, CXCR4, PIK3CG, EFHC1, IL1B, ITGB3, SCN5A, CCL2, GPR65, BMP4, CEMIP, TBXAS1, SNX10, KISS1, LPAR1, S1PR3, TGM2, F2RL1, F2RL2, NEO1 |  |
| Plasma Membrane Organization | 5 | 6 | 3.06E-03 | 0.018 | | BAIAP2L1, CLU, FAT4, DMKN, CXCR4 |  |
| Regulation Of Cytokine Secretion | 9 | 3 | 3.08E-03 | 0.011 | | TNFRSF21, TRIM6, IL1RL1, GBP1, CYP2J2, PTPN22, IL1B, CARD8, F2RL1 |  |
| Regulation Of Heart Growth | 6 | 5 | 3.11E-03 | 0.015 | | MEF2C, GATA6, NOG, GJA1, WT1, TGFBR3 |  |
| Behavioral Response To Pain | 3 | 13 | 3.12E-03 | 0.013 | | SCN9A, THBS1, KCNIP3 |  |
| Response To Inactivity | 3 | 13 | 3.14E-03 | 0.008 | | FBXO32, MMP1, SCN5A |  |
| Positive Regulation Of Branching Involved In Ureteric Bud Morphogenesis | 3 | 13 | 3.14E-03 | 0.008 | | VEGFA, BMP4, NOG |  |
| Positive Regulation Of Cytokine Production Involved In Immune Response | 4 | 8 | 3.14E-03 | 0.008 | | IL1B, HLA-A, TRIM6, F2RL1 |  |
| Interleukin-12-Mediated Signaling Pathway | 4 | 8 | 3.17E-03 | 0.010 | | PDCD4, SERPINB2, LCP1, PLCB1 |  |
| Synapse Organization | 8 | 4 | 3.17E-03 | 0.010 | | PCDHB2, ADGRL3, ADGRF1, ROBO1, PCDHB5, SLITRK1, PCDHB6, PCDHB16 |  |
| Apoptotic Process | 21 | 2 | 3.23E-03 | 0.015 | | TNFRSF21, PDCD4, ARRB1, BCL2A1, GJA1, CXCR4, PIK3CG, IL1B, CARD8, GPR65, BMP4, IL24, IRAK3, TNFSF10, CRADD, MEF2C, KCNIP3, G0S2, FGF13, WT1, EPHA7 |  |
| Regulation Of System Process | 19 | 2 | 3.23E-03 | 0.018 | | TNFRSF21, ATP2B4, MYL9, GJA1, PIK3CG, IL1B, FBXO32, INHBB, CHRM3, CLIC2, SCN5A, MEF2C, DMD, DSP, TBXAS1, KISS1, CYP2J2, TGFB2, F2RL1 |  |
| Regulation Of Cellular Metabolic Process | 119 | 1 | 3.24E-03 | 0.018 | | ARRB1, ANKRD30A, ATP2B4, ARRDC4, BCL2A1, AADAC, TSPYL5, CLU, BMP4, C4BPB, CCND2, DMD, TRIM6, PIK3AP1, CPT1A, CSF1, CST4, FGF13, FOXR2, LPAR1, EPHA7, EPHA3, EYA4, FAM172BP, F2RL1, F3, ACSL1, EFEMP1, FBP1, HAS2, NCKAP1L, GATA6, GBP1, PRICKLE1, GJA1, IGFBP4, IL1B, INHBB, ITGB3, FOXA1, HES1, SH3RF2, MEF2C, MEIS3P1, MAP3K1, MMP3, NRK, KISS1, SERPINB2, ZNF438, NEO1, PKIA, PIK3CG, PLAU, ADGRF1, SSX8, ROBO1, CCL2, HMSD, SOX4, SSX1, TCF4, SNAI2, VEGFA, WT1, TGFA, TGFB2, THBS1, TGFBR3, TIMP3, TLR3, ZNF391, HIST2H4B, ZNF43, CXCR4, GPRC5A, CST7, CASK, SAMD5, SERPINB7, TNFSF10, CRADD, GMFG, PDLIM1, NOG, LIPG, RAD50, OPTN, MBNL2, CTDSPL, NUAK1, PLK2, ZNF716, MIR181B1, CARD8, IL24, IRAK3, SP140, ARL2BP, PLCB1, PTPN22, PDCD4, PCDH11X, TRIB2, CDON, MAFIP, KCNIP3, BCL11A, LMO3, ZMIZ1, CEMIP, ZNF608, PMEPA1, PRDM9, PRDM8, SAMSN1, NXN, CPEB4, AFAP1L2 |  |
| Tissue Remodeling | 6 | 5 | 3.25E-03 | 0.016 | | MEF2C, GJA1, VEGFA, TGFB2, TGFBR3, TGM2 |  |
| Regulation Of Neuron Apoptotic Process | 10 | 3 | 3.26E-03 | 0.013 | | ARRB1, MEF2C, KCNIP3, OPTN, IL1B, VEGFA, CPEB4, CCL2, TGFB2, EPHA7 |  |
| Comma-Shaped Body Morphogenesis | 2 | 28 | 3.27E-03 | 0.016 | | WT1, HES1 |  |
| Positive Regulation Of Glomerular Mesangial Cell Proliferation | 2 | 28 | 3.36E-03 | 0.005 | | ITGB3, SERPINB7 |  |
| Epithelial-Mesenchymal Cell Signaling | 2 | 28 | 3.36E-03 | 0.005 | | BMP4, FOXA1 |  |
| Corticospinal Tract Morphogenesis | 2 | 28 | 3.36E-03 | 0.005 | | CDH11, PRDM8 |  |
| Positive Regulation Of Behavioral Fear Response | 2 | 28 | 3.36E-03 | 0.005 | | MEF2C, GJA1 |  |
| Negative Regulation Of Peptidyl-Cysteine S-Nitrosylation | 2 | 28 | 3.36E-03 | 0.005 | | DMD, ATP2B4 |  |
| Positive Regulation Of Fear Response | 2 | 28 | 3.36E-03 | 0.005 | | MEF2C, GJA1 |  |
| Regulation Of Cd8-Positive, Alpha-Beta T Cell Proliferation | 2 | 28 | 3.36E-03 | 0.005 | | PTPN22, HLA-A |  |
| Retinal Blood Vessel Morphogenesis | 2 | 28 | 3.36E-03 | 0.005 | | VEGFA, LAMA1 |  |
| Mammary Duct Terminal End Bud Growth | 2 | 28 | 3.36E-03 | 0.005 | | ROBO1, CSF1 |  |
| Positive Regulation Of Muscle Cell Apoptotic Process | 4 | 8 | 3.36E-03 | 0.005 | | PDCD4, ARRB1, FBXO32, GATA6 |  |
| Negative Regulation Of Cytokine Secretion | 5 | 6 | 3.43E-03 | 0.010 | | TNFRSF21, PTPN22, CARD8, GBP1, F2RL1 |  |
| Cardiac Muscle Tissue Morphogenesis | 5 | 6 | 3.44E-03 | 0.011 | | HEG1, DSP, TGFB2, TGFBR3, NOG |  |
| Negative Regulation Of Response To External Stimulus | 12 | 3 | 3.44E-03 | 0.011 | | PDCD4, GJA1, PLAU, SNAI2, ROBO1, CARD8, SERPINB2, CCL2, PTPRS, THBS1, CASK, NEO1 |  |
| Eye Development | 7 | 4 | 3.47E-03 | 0.017 | | MAP3K1, INHBB, VEGFA, WT1, BMP4, TGFB2, EFEMP1 |  |
| Lung Development | 7 | 4 | 3.47E-03 | 0.014 | | CRISPLD2, HEG1, VEGFA, BMP4, FOXA1, TGFB2, HES1 |  |
| Positive Regulation Of Stem Cell Differentiation | 3 | 12 | 3.47E-03 | 0.014 | | BMP4, TGFB2, GATA6 |  |
| Animal Organ Formation | 3 | 12 | 3.56E-03 | 0.008 | | BMP4, GATA6, NOG |  |
| Cellular Response To Low-Density Lipoprotein Particle Stimulus | 3 | 12 | 3.56E-03 | 0.008 | | ABCA1, HMGCS1, F3 |  |
| Regulation Of Endopeptidase Activity | 15 | 2 | 3.56E-03 | 0.008 | | ARRB1, BCL2A1, ROBO1, CARD8, HMSD, IL24, SERPINB7, TNFSF10, CRADD, VEGFA, SERPINB2, THBS1, TIMP3, EPHA7, F3 |  |
| Regulation Of Epithelial Cell Differentiation | 8 | 4 | 3.60E-03 | 0.017 | | MIR181B1, IL1B, VEGFA, S1PR3, FAT4, BMP4, TGFB2, HES1 |  |
| Negative Regulation Of Interleukin-6 Production | 4 | 8 | 3.67E-03 | 0.015 | | PTPN22, ARRB1, NCKAP1L, IRAK3 |  |
| Leukocyte Differentiation | 12 | 3 | 3.70E-03 | 0.010 | | SOX4, MEF2C, BCL2A1, SNX10, CSF1, PLCB1, BCL11A, PTPN22, VEGFA, BMP4, F2RL1, DOCK10 |  |
| Negative Regulation Of Metabolic Process | 71 | 1 | 3.70E-03 | 0.017 | | ARRB1, ATP2B4, BCL2A1, MIR181B1, CARD8, CLU, BMP4, IL24, C4BPB, IRAK3, DMD, TRIM6, PLCB1, CST4, CYP2J2, PTPN22, FAM172BP, F2RL1, FBP1, PDCD4, PCDH11X, NCKAP1L, GATA6, GBP1, PRICKLE1, GJA1, IL1B, INHBB, TRIB2, ITGB3, FOXA1, HES1, SH3RF2, MIR604, MEF2C, MMP3, KCNIP3, BCL11A, SERPINB2, ZNF438, PKIA, PDE3B, PIK3CG, ROBO1, LMO3, HMSD, SOX4, SSX1, ZNF608, TCF4, PMEPA1, PRDM8, SNAI2, SAMSN1, VEGFA, WT1, NXN, TGFB2, THBS1, TGFBR3, TIMP3, HIST2H4B, CPEB4, GPRC5A, CST7, SERPINB7, GMFG, NOG, RAD50, OPTN, CTDSPL |  |
| Response To Hypoxia | 13 | 3 | 3.71E-03 | 0.016 | | SOX4, MMP3, PDLIM1, SDC2, GATA6, NOG, CXCR4, PLAU, IL1B, VEGFA, TGFB2, THBS1, TGFBR3 |  |
| Carbohydrate Derivative Metabolic Process | 24 | 2 | 3.77E-03 | 0.017 | | PDE10A, HAS2, PDE3B, MAMDC2, GALC, PORCN, AK4, MGAT4A, ELOVL7, IL1B, SCP2, HMGCS1, CASK, ADAMTS12, HDDC2, CEMIP, SDC2, B4GALT6, UGCG, VEGFA, SLC37A2, CHST15, ACSL1, FBP1 |  |
| Cell Morphogenesis | 10 | 3 | 3.80E-03 | 0.018 | | MEF2C, MAP3K1, HEG1, NCKAP1L, POF1B, CDH11, ITGB3, CLU, TGFB2, HES1 |  |
| Negative Regulation Of Cell Motility | 12 | 3 | 3.81E-03 | 0.016 | | ARHGDIB, MEF2C, KISS1, NOG, PLCB1, NAV3, ROBO1, SCP2, CCL2, IL24, THBS1, TGFBR3 |  |
| Positive Regulation Of Lipid Catabolic Process | 3 | 11 | 3.94E-03 | 0.016 | | IL1B, AADAC, CPT1A |  |
| Negative Regulation Of Stem Cell Differentiation | 3 | 11 | 4.00E-03 | 0.008 | | TRIM6, PRICKLE1, HES1 |  |
| Regulation Of Calcium Ion Transmembrane Transport | 8 | 4 | 4.00E-03 | 0.008 | | CEMIP, DMD, PIK3CG, PTPN22, ITGB3, CLIC2, BMP4, TGFB2 |  |
| Regulation Of Peptidase Activity | 16 | 2 | 4.03E-03 | 0.014 | | ARRB1, BCL2A1, ROBO1, CARD8, HMSD, IL24, CST7, SERPINB7, TNFSF10, CRADD, VEGFA, SERPINB2, THBS1, TIMP3, EPHA7, F3 |  |
| Positive Regulation Of Immune System Process | 30 | 2 | 4.04E-03 | 0.017 | | BCL2A1, CLU, CCL2, HMSD, C4BPB, IRAK3, ZMIZ1, TRIM6, PIK3AP1, CSF1, PTPN22, VEGFA, EFNB2, TGFB2, THBS1, TLR3, F2RL1, TNFRSF21, NCKAP1L, HLA-A, HLA-B, IL1B, IL13RA2, ITGB3, HLA-DRA, HES1, MEF2C, MAP3K1, IL1RL1, PLPP4 |  |
| Positive Regulation Of Cytokine Secretion | 7 | 4 | 4.10E-03 | 0.017 | | TRIM6, IL1RL1, CYP2J2, PTPN22, IL1B, CARD8, F2RL1 |  |
| Unsaturated Fatty Acid Metabolic Process | 6 | 5 | 4.16E-03 | 0.014 | | PTGES, TBXAS1, CYP2J2, PTGR1, SCP2, ACSL1 |  |
| Regulation Of Jun Kinase Activity | 6 | 5 | 4.23E-03 | 0.013 | | PDCD4, MAP3K1, NRK, PTPN22, IL1B, SAMD5 |  |
| Positive Regulation Of Protein Acetylation | 4 | 7 | 4.23E-03 | 0.013 | | SOX4, IL1B, ARRB1, SNAI2 |  |
| Positive Regulation Of Gtpase Activity | 13 | 3 | 4.27E-03 | 0.010 | | ARHGDIB, ARRB1, NCKAP1L, EVI5, PLCB1, TBC1D30, ASAP1, TIAM2, CCL2, GPR65, F2RL1, DOCK10, RGS10 |  |
| Positive Regulation Of Developmental Growth | 9 | 3 | 4.32E-03 | 0.017 | | MEF2C, GATA6, CSF1, PLCB1, CXCR4, BCL11A, VEGFA, WT1, TGFBR3 |  |
| Negative Regulation Of Cysteine-Type Endopeptidase Activity | 6 | 5 | 4.35E-03 | 0.015 | | ARRB1, BCL2A1, CARD8, VEGFA, IL24, THBS1 |  |
| Cell Part Morphogenesis | 11 | 3 | 4.41E-03 | 0.013 | | NRK, NCKAP1L, SDC2, GJA1, PRDM8, PARD6B, CDH11, SLITRK1, ITGB3, CLU, DOCK10 |  |
| Regulation Of Protein Localization | 26 | 2 | 4.42E-03 | 0.016 | | TNFRSF21, ARRB1, PKIA, PDE3B, GBP1, GJA1, IL1B, CARD8, INHBB, BMP4, CASK, SOX4, CEMIP, ARL2BP, TRIM6, IL1RL1, CPT1A, LCP1, CYP2J2, PTPN22, VEGFA, TGFB2, STOM, EPHA3, F2RL1, NEO1 |  |
| S-Shaped Body Morphogenesis | 2 | 25 | 4.43E-03 | 0.017 | | WT1, HES1 |  |
| Blood Vessel Endothelial Cell Proliferation Involved In Sprouting Angiogenesis | 2 | 25 | 4.44E-03 | 0.005 | | VEGFA, BMP4 |  |
| Positive Regulation Of Gonadotropin Secretion | 2 | 25 | 4.44E-03 | 0.005 | | INHBB, KISS1 |  |
| Regulation Of Transforming Growth Factor Beta2 Production | 2 | 25 | 4.44E-03 | 0.005 | | TGFB2, GATA6 |  |
| Positive Regulation Of Toll-Like Receptor 3 Signaling Pathway | 2 | 25 | 4.44E-03 | 0.005 | | PTPN22, F2RL1 |  |
| Regulation Of Epithelial To Mesenchymal Transition Involved In Endocardial Cushion Formation | 2 | 25 | 4.44E-03 | 0.005 | | TGFB2, NOG |  |
| Negative Regulation Of Apoptotic Process Involved In Development | 2 | 25 | 4.44E-03 | 0.005 | | WT1, TGFBR3 |  |
| Interleukin-1 Beta Secretion | 2 | 25 | 4.44E-03 | 0.005 | | ABCA1, F2RL1 |  |
| Regulation Of Branching Morphogenesis Of A Nerve | 2 | 25 | 4.44E-03 | 0.005 | | BCL11A, IL1B |  |
| Positive Regulation Of Cysteine-Type Endopeptidase Activity Involved In Apoptotic Process | 7 | 4 | 4.44E-03 | 0.005 | | ARRB1, ROBO1, CARD8, IL24, F3, TNFSF10, CRADD |  |
| Regulation Of Metanephros Development | 3 | 11 | 4.46E-03 | 0.014 | | WT1, FAT4, BMP4 |  |
| Regulation Of Branching Involved In Ureteric Bud Morphogenesis | 3 | 11 | 4.48E-03 | 0.008 | | VEGFA, BMP4, NOG |  |
| Positive Regulation Of Calcium Ion Import | 3 | 11 | 4.48E-03 | 0.008 | | CCL2, CASK, CXCR4 |  |
| Regulation Of Cellular Response To Vascular Endothelial Growth Factor Stimulus | 3 | 11 | 4.48E-03 | 0.008 | | ADAMTS12, ROBO1, TCF4 |  |
| Positive Regulation Of Protein Complex Assembly | 10 | 3 | 4.48E-03 | 0.008 | | MAP3K1, MMP3, MMP1, NCKAP1L, LCP1, NAV3, ASAP1, VEGFA, BAIAP2L1, CLU |  |
| Positive Regulation Of Erk1 And Erk2 Cascade | 10 | 3 | 4.53E-03 | 0.016 | | ARRB1, PTPN22, IL1B, VEGFA, ITGB3, CCL2, BMP4, EPHA7, EPHA3, F2RL1 |  |
| Plasma Lipoprotein Particle Organization | 4 | 7 | 4.53E-03 | 0.016 | | ABCA1, BMP1, PLTP, LIPG |  |
| Regulation Of Granulocyte Chemotaxis | 4 | 7 | 4.58E-03 | 0.010 | | IL1B, NCKAP1L, THBS1, CSF1 |  |
| Telencephalon Cell Migration | 4 | 7 | 4.58E-03 | 0.010 | | EFHC1, FGF13, NRP2, CXCR4 |  |
| Odontogenesis | 6 | 5 | 4.58E-03 | 0.010 | | GATA6, CSF1, TUFT1, SCN5A, BMP4, TGFB2 |  |
| Response To Bronchodilator | 5 | 5 | 4.59E-03 | 0.013 | | MMP3, DPYSL2, BMP4, THBS1, RGS10 |  |
| Leukocyte Proliferation | 6 | 5 | 4.73E-03 | 0.011 | | MEF2C, CSF1, CXCR4, PIK3CG, CLU, F2RL1 |  |
| Granulocyte Migration | 6 | 5 | 4.78E-03 | 0.013 | | NCKAP1L, PIK3CG, IL1B, VEGFA, CCL2, TGFB2 |  |
| Regulation Of Nik/Nf-Kappab Signaling | 6 | 5 | 4.78E-03 | 0.013 | | PDCD4, TRIM6, PTPN22, IL1B, SAMD5, TLR3 |  |
| Neuron Projection Morphogenesis | 10 | 3 | 4.78E-03 | 0.013 | | NRK, NCKAP1L, SDC2, GJA1, PRDM8, PARD6B, CDH11, SLITRK1, CLU, DOCK10 |  |
| Positive Regulation Of Interleukin-8 Production | 4 | 7 | 4.86E-03 | 0.015 | | AFAP1L2, IL1B, TLR3, F2RL1 |  |
| Regulation Of Chondrocyte Differentiation | 4 | 7 | 4.91E-03 | 0.010 | | ADAMTS12, BMP4, SNAI2, EFEMP1 |  |
| Positive Regulation Of Jnk Cascade | 7 | 4 | 4.91E-03 | 0.010 | | MAP3K1, NRK, PLCB1, IL1B, SAMD5, TLR3, F2RL1 |  |
| Tube Formation | 7 | 4 | 4.94E-03 | 0.014 | | SOX4, NOG, PRICKLE1, VEGFA, BMP4, TGFB2, TGM2 |  |
| Positive Regulation Of Reproductive Process | 5 | 5 | 4.94E-03 | 0.014 | | PLCB1, PLAU, PRDM9, INHBB, WT1 |  |
| Lung Morphogenesis | 3 | 11 | 4.97E-03 | 0.011 | | BMP4, FOXA1, NOG |  |
| Negative Regulation Of Lipid Catabolic Process | 3 | 11 | 5.00E-03 | 0.008 | | IL1B, PDE3B, PIK3CG |  |
| Positive Regulation Of P38Mapk Cascade | 3 | 11 | 5.00E-03 | 0.008 | | IL1B, VEGFA, MIR181B1 |  |
| Camera-Type Eye Morphogenesis | 3 | 11 | 5.00E-03 | 0.008 | | TENM3, VEGFA, BMP4 |  |
| Developmental Growth | 13 | 2 | 5.00E-03 | 0.008 | | ZMIZ1, HEG1, HLA-A, GJA1, CSF1, PLAU, PLXNA1, ROBO1, NRP2, SLITRK1, SORBS2, BMP4, TIMP3 |  |
| Organonitrogen Compound Metabolic Process | 103 | 1 | 5.03E-03 | 0.016 | | ARRB1, ARRDC4, ABCA1, AK4, FBXO32, MARCH3, CHRM3, CLU, BMP4, BMP1, C4BPB, DMD, DSP, TRIM6, CPT1A, CSF1, CTSD, FGF13, FLG, EFNB2, EPHA7, EPHA3, EYA4, F3, F13A1, ACSL1, EFEMP1, HAS2, GALC, GATA6, TTLL11, IGFBP4, IL1B, ZDHHC15, HMGCS1, SH3RF2, MEF2C, MAP3K1, MMP3, MMP1, NRK, LTBP1, SFTA3, SLC37A2, HECTD4, TMPRSS15, PDE3B, MAMDC2, PIK3CG, PLAU, RRBP1, SCP2, CCL2, LAMA1, PTPRS, SDC2, UGCG, VEGFA, TGFB2, THBS1, TGFBR3, TGM2, TLL1, TLR3, SERINC2, SLC7A5, HIST2H4B, CDC42BPA, CASK, GMFG, PTGES, B4GALT6, CTDSPL, NUAK1, PLK2, PDE10A, MGAT4A, IL24, PRSS23, IRAK3, PTPN22, TRIB2, HDDC2, CPA4, BCL11A, SHC3, CHST15, LPCAT2, TENM3, KLHL4, CPPED1, SLC44A2, CEMIP, MARCH4, PRDM9, PRDM8, FKBP10, PORCN, CDK15, ELOVL7, CPEB4, ADAMTS12, APOL6 |  |
| Macromolecule Modification | 64 | 1 | 5.05E-03 | 0.016 | | PLK2, ARRB1, ARRDC4, ABCA1, MGAT4A, FBXO32, MARCH3, CHRM3, BMP4, IL24, PRSS23, IRAK3, DSP, TRIM6, CSF1, FGF13, FLG, PTPN22, EFNB2, EPHA7, EPHA3, EYA4, F13A1, EFEMP1, TTLL11, IGFBP4, IL1B, ZDHHC15, TRIB2, SH3RF2, MEF2C, MAP3K1, CPA4, NRK, LTBP1, BCL11A, SHC3, HECTD4, RBM47, MAMDC2, PIK3CG, KLHL4, CCL2, LAMA1, PTPRS, CPPED1, MARCH4, SDC2, PRDM9, PRDM8, FKBP10, TGFB2, THBS1, TGFBR3, TGM2, TLR3, PORCN, CDK15, CDC42BPA, CASK, GMFG, B4GALT6, CTDSPL, NUAK1 |  |
| Response To Extracellular Stimulus | 19 | 2 | 5.09E-03 | 0.016 | | ABCA1, IL1B, INHBB, CPEB4, HMGCS1, CYP24A1, MEF2C, MMP3, MMP1, CTSD, LIPG, LTBP1, BCL11A, OPTN, VEGFA, GDAP1, TGFB2, TIMP3, ACSL1 |  |
| Response To Oxygen Levels | 15 | 2 | 5.12E-03 | 0.017 | | GATA6, CXCR4, PLAU, IL1B, CPEB4, SOX4, MMP3, PDLIM1, SDC2, NOG, VEGFA, LPAR1, TGFB2, THBS1, TGFBR3 |  |
| Regulation Of Lipid Metabolic Process | 13 | 2 | 5.16E-03 | 0.017 | | TNFRSF21, AADAC, PDE3B, ABCA1, CPT1A, PIK3CG, G0S2, SNAI2, IL1B, TIAM2, SCP2, HMGCS1, ACSL1 |  |
| Positive Regulation Of Gliogenesis | 5 | 5 | 5.22E-03 | 0.016 | | NOG, CSF1, CXCR4, IL1B, HES1 |  |
| Negative Regulation Of Erk1 And Erk2 Cascade | 5 | 5 | 5.22E-03 | 0.011 | | ARRB1, DMD, GBP1, LMO3, TIMP3 |  |
| Memory | 7 | 4 | 5.22E-03 | 0.011 | | PLK2, NOG, PLCB1, ADGRF1, FGF13, IL1B, CCND2 |  |
| Cation Homeostasis | 20 | 2 | 5.28E-03 | 0.013 | | ATP2B4, GJA1, CXCR4, PIK3CG, EFHC1, IL1B, ITGB3, SCN5A, CCL2, GPR65, BMP4, CEMIP, SNX10, KISS1, LPAR1, S1PR3, TGM2, F2RL1, F2RL2, NEO1 |  |
| Cellular Component Organization | 102 | 1 | 5.32E-03 | 0.017 | | ARRB1, ABCA1, TSPYL5, EFHC1, CDH11, SLITRK1, CLU, BMP1, DMKN, SYTL3, DMD, DPYSL2, DSP, TRIM6, CPT1A, CTSD, FGF13, LPAR1, STOM, EPHA3, EYA4, FAM172BP, F2RL1, FBP1, HAS2, NCKAP1L, HLA-A, GBP1, GJA1, TTLL11, ZDHHC15, ITGB3, TOR1AIP2, HLA-DRA, FOXA1, HES1, MEF2C, MAP3K1, MMP3, MMP1, NRK, SAMD9L, KISS1, LAMA3, LAMA4, LCP1, MPZL3, ADGRF1, PLTP, ROBO1, SCP2, CCL2, LAMA1, TCF4, SDC2, SNAI2, WT1, TGFA, TGFB2, THBS1, TGFBR3, TGM2, TLL1, HIST2H4B, SRPX, CXCR4, SORBS2, GPR65, CDC42BPA, GMFG, LIPG, TSPAN2, RAD50, OPTN, PLK2, ADGRL3, PCDHB5, FLRT2, ABI3BP, PCDH17, MAFIP, CPA4, SNX10, ASAP1, GDAP1, LPCAT2, BAIAP2L1, PCDHB6, DOCK10, HRASLS, ZMIZ1, ATP8B2, HEG1, PCDHB2, PRDM9, PRDM8, FKBP10, PCDHB16, POF1B, FAT4, CRISPLD2, PARD6B |  |
| Heart Development | 11 | 3 | 5.36E-03 | 0.016 | | SOX4, MEF2C, HEG1, GATA6, GJA1, ROBO1, RAD50, NRP2, WT1, BMP4, TGFB2 |  |
| Positive Regulation Of Neuron Death | 6 | 4 | 5.37E-03 | 0.016 | | OPTN, IL1B, CLU, EFNB2, TGFB2, EPHA7 |  |
| Regulation Of Nitrogen Compound Metabolic Process | 114 | 1 | 5.39E-03 | 0.012 | | ARRB1, ANKRD30A, ATP2B4, ARRDC4, BCL2A1, TSPYL5, CLU, BMP4, C4BPB, CCND2, DMD, TRIM6, PIK3AP1, CSF1, CST4, FGF13, FOXR2, LPAR1, EPHA7, EPHA3, EYA4, FAM172BP, F2RL1, F3, ACSL1, EFEMP1, FBP1, HAS2, NCKAP1L, GATA6, GBP1, PRICKLE1, GJA1, IGFBP4, IL1B, INHBB, ITGB3, FOXA1, HES1, SH3RF2, MEF2C, MEIS3P1, MAP3K1, NRK, SAMD9L, KISS1, SERPINB2, ZNF438, NEO1, PKIA, PIK3CG, ADGRF1, SSX8, ROBO1, CCL2, HMSD, SOX4, SSX1, TCF4, SNAI2, VEGFA, WT1, TGFA, TGFB2, THBS1, TGFBR3, TIMP3, TLR3, ZNF391, HIST2H4B, ZNF43, CXCR4, GPRC5A, CST7, CASK, SAMD5, SERPINB7, TNFSF10, CRADD, GMFG, PDLIM1, NOG, LIPG, RAD50, MBNL2, CTDSPL, NUAK1, PLK2, ZNF716, MIR181B1, CARD8, IL24, IRAK3, SP140, ARL2BP, PLCB1, PTPN22, PDCD4, TRIB2, CDON, MAFIP, KCNIP3, BCL11A, LMO3, ZMIZ1, CEMIP, ZNF608, PMEPA1, PRDM9, PRDM8, SAMSN1, NXN, CPEB4, AFAP1L2 |  |
| Male Gonad Development | 7 | 4 | 5.41E-03 | 0.015 | | GATA6, INHBB, WT1, HMGCS1, TGFB2, TLR3, TNFSF10 |  |
| Positive Regulation Of Protein Transport | 14 | 2 | 5.46E-03 | 0.013 | | ARRB1, GJA1, IL1B, CARD8, CASK, SOX4, CEMIP, TRIM6, IL1RL1, CYP2J2, PTPN22, TGFB2, STOM, F2RL1 |  |
| Regulation Of Cardiac Muscle Contraction | 5 | 5 | 5.46E-03 | 0.017 | | DMD, DSP, PIK3CG, CLIC2, SCN5A |  |
| Cellular Response To Nutrient | 5 | 5 | 5.49E-03 | 0.011 | | MMP1, LTBP1, BCL11A, OPTN, GDAP1 |  |
| Prostate Gland Epithelium Morphogenesis | 3 | 10 | 5.49E-03 | 0.011 | | BMP4, FOXA1, NOG |  |
| Response To Lipoprotein Particle | 3 | 10 | 5.54E-03 | 0.008 | | ABCA1, HMGCS1, F3 |  |
| Forebrain Cell Migration | 4 | 7 | 5.54E-03 | 0.008 | | EFHC1, FGF13, NRP2, CXCR4 |  |
| Regulation Of Calcium Ion Import | 4 | 7 | 5.60E-03 | 0.010 | | CCL2, CASK, HES1, CXCR4 |  |
| Regulation Of Proteolysis | 23 | 2 | 5.60E-03 | 0.010 | | PLK2, ARRB1, BCL2A1, PRICKLE1, ROBO1, IL1B, CARD8, TRIB2, CLU, HMSD, IL24, CST7, C4BPB, SERPINB7, TNFSF10, CRADD, CST4, VEGFA, SERPINB2, THBS1, TIMP3, EPHA7, F3 |  |
| Regulation Of Heart Contraction | 9 | 3 | 5.63E-03 | 0.017 | | DMD, ATP2B4, DSP, GJA1, PIK3CG, CYP2J2, CLIC2, SCN5A, TGFB2 |  |
| Antigen Processing And Presentation Of Exogenous Peptide Antigen Via Mhc Class I, Tap-Independent | 2 | 22 | 5.65E-03 | 0.015 | | HLA-A, HLA-B |  |
| Positive Regulation Of Cytokine Secretion Involved In Immune Response | 2 | 22 | 5.67E-03 | 0.005 | | TRIM6, F2RL1 |  |
| Heart Field Specification | 2 | 22 | 5.67E-03 | 0.005 | | MEF2C, BMP4 |  |
| Secondary Heart Field Specification | 2 | 22 | 5.67E-03 | 0.005 | | MEF2C, BMP4 |  |
| Regulation Of Ventricular Cardiac Muscle Cell Membrane Depolarization | 2 | 22 | 5.67E-03 | 0.005 | | SCN5A, GJA1 |  |
| Positive Regulation Of Cell Proliferation Involved In Kidney Development | 2 | 22 | 5.67E-03 | 0.005 | | ITGB3, SERPINB7 |  |
| Regulation Of Cardiac Epithelial To Mesenchymal Transition | 2 | 22 | 5.67E-03 | 0.005 | | TGFB2, NOG |  |
| Regulation Of Vitamin D Biosynthetic Process | 2 | 22 | 5.67E-03 | 0.005 | | IL1B, SNAI2 |  |
| Cellular Response To L-Glutamate | 2 | 22 | 5.67E-03 | 0.005 | | BCL11A, OPTN |  |
| Inositol Trisphosphate Metabolic Process | 2 | 22 | 5.67E-03 | 0.005 | | SCP2, PLCB1 |  |
| Regulation Of Myosin-Light-Chain-Phosphatase Activity | 2 | 22 | 5.67E-03 | 0.005 | | NCKAP1L, NUAK1 |  |
| Visceral Serous Pericardium Development | 2 | 22 | 5.67E-03 | 0.005 | | WT1, TGFBR3 |  |
| Cellular Chemical Homeostasis | 21 | 2 | 5.67E-03 | 0.005 | | ATP2B4, GJA1, CXCR4, PIK3CG, PLAU, EFHC1, IL1B, ITGB3, CCL2, GPR65, BMP4, SOX4, MEF2C, CEMIP, TBXAS1, KISS1, LPAR1, S1PR3, TGM2, F2RL1, F2RL2 |  |
| Cellular Metal Ion Homeostasis | 17 | 2 | 5.68E-03 | 0.017 | | ATP2B4, GJA1, CXCR4, PIK3CG, EFHC1, IL1B, ITGB3, CCL2, GPR65, BMP4, CEMIP, KISS1, LPAR1, S1PR3, TGM2, F2RL1, F2RL2 |  |
| Plasma Membrane Bounded Cell Projection Morphogenesis | 10 | 3 | 5.70E-03 | 0.017 | | NRK, NCKAP1L, SDC2, GJA1, PRDM8, PARD6B, CDH11, SLITRK1, CLU, DOCK10 |  |
| Blood Coagulation | 8 | 3 | 5.72E-03 | 0.015 | | GATA6, PLAU, ITGB3, C4BPB, F2RL1, F3, F2RL2, F13A1 |  |
| Cellular Component Organization Or Biogenesis | 103 | 1 | 5.74E-03 | 0.014 | | ARRB1, ARRDC4, ABCA1, TSPYL5, EFHC1, CDH11, SLITRK1, CLU, BMP1, DMKN, SYTL3, DMD, DPYSL2, DSP, TRIM6, CPT1A, CTSD, FGF13, LPAR1, STOM, EPHA3, EYA4, FAM172BP, F2RL1, FBP1, HAS2, NCKAP1L, HLA-A, GBP1, GJA1, TTLL11, ZDHHC15, ITGB3, TOR1AIP2, HLA-DRA, FOXA1, HES1, MEF2C, MAP3K1, MMP3, MMP1, NRK, SAMD9L, KISS1, LAMA3, LAMA4, LCP1, MPZL3, ADGRF1, PLTP, ROBO1, SCP2, CCL2, LAMA1, TCF4, SDC2, SNAI2, WT1, TGFA, TGFB2, THBS1, TGFBR3, TGM2, TLL1, HIST2H4B, SRPX, CXCR4, SORBS2, GPR65, CDC42BPA, GMFG, LIPG, TSPAN2, RAD50, OPTN, PLK2, ADGRL3, PCDHB5, FLRT2, ABI3BP, PCDH17, MAFIP, CPA4, SNX10, ASAP1, GDAP1, LPCAT2, BAIAP2L1, PCDHB6, DOCK10, HRASLS, ZMIZ1, ATP8B2, HEG1, PCDHB2, PRDM9, PRDM8, FKBP10, PCDHB16, POF1B, FAT4, CRISPLD2, PARD6B |  |
| Regulation Of Striated Muscle Tissue Development | 8 | 3 | 5.88E-03 | 0.015 | | CDON, MEF2C, GATA6, NOG, GJA1, BMP4, EFNB2, TGFBR3 |  |
| Response To Peptide | 15 | 2 | 5.90E-03 | 0.014 | | GRB14, PDE3B, GJA1, IL1B, INHBB, ITGB3, SCP2, CCND2, TNFSF10, MMP1, IL1RL1, PLCB1, PTPN22, TGFBR3, FBP1 |  |
| Protein-Lipid Complex Subunit Organization | 4 | 7 | 5.97E-03 | 0.017 | | ABCA1, BMP1, PLTP, LIPG |  |
| Cell Fate Determination | 4 | 7 | 5.97E-03 | 0.010 | | MEF2C, BMP4, GATA6, HES1 |  |
| Epidermal Growth Factor Receptor Signaling Pathway | 4 | 7 | 5.97E-03 | 0.010 | | SHC3, TGFA, EFEMP1, FAM83B |  |
| Positive Regulation Of Notch Signaling Pathway | 4 | 7 | 5.97E-03 | 0.010 | | ROBO1, ZMIZ1, TGFB2, HES1 |  |
| Regulation Of Collagen Biosynthetic Process | 4 | 7 | 5.97E-03 | 0.010 | | BMP4, SERPINB7, LTBP1, CYP2J2 |  |
| Negative Regulation Of Macromolecule Metabolic Process | 66 | 1 | 5.97E-03 | 0.010 | | ARRB1, ATP2B4, BCL2A1, MIR181B1, CARD8, CLU, BMP4, IL24, C4BPB, IRAK3, DMD, TRIM6, PLCB1, CST4, PTPN22, FAM172BP, F2RL1, FBP1, PDCD4, NCKAP1L, GATA6, GBP1, PRICKLE1, GJA1, IL1B, INHBB, TRIB2, ITGB3, FOXA1, HES1, SH3RF2, MIR604, MEF2C, KCNIP3, BCL11A, SERPINB2, ZNF438, PKIA, ROBO1, LMO3, HMSD, SOX4, SSX1, ZNF608, TCF4, PMEPA1, PRDM8, SNAI2, SAMSN1, VEGFA, WT1, NXN, TGFB2, THBS1, TGFBR3, TIMP3, HIST2H4B, CPEB4, GPRC5A, CST7, SERPINB7, GMFG, NOG, RAD50, OPTN, CTDSPL |  |
| Adenylate Cyclase-Modulating G-Protein Coupled Receptor Signaling Pathway | 8 | 3 | 6.02E-03 | 0.016 | | ARRB1, ABCA1, ADGRF1, CHRM3, LPAR1, S1PR3, GPR65, LGR5 |  |
| Positive Regulation Of Protein Oligomerization | 3 | 10 | 6.07E-03 | 0.014 | | MMP3, MMP1, CLU |  |
| Regulation Of Muscle System Process | 10 | 3 | 6.12E-03 | 0.008 | | MEF2C, DMD, ATP2B4, DSP, MYL9, PIK3CG, FBXO32, CHRM3, CLIC2, SCN5A |  |
| Regulation Of Actin Cytoskeleton Organization | 12 | 3 | 6.13E-03 | 0.015 | | GMFG, ARHGDIB, MEF2C, MAP3K1, NCKAP1L, ITGB3, BAIAP2L1, LPAR1, GPR65, TGFB2, EPHA3, F2RL1 |  |
| Growth | 13 | 2 | 6.13E-03 | 0.016 | | ZMIZ1, HEG1, HLA-A, GJA1, CSF1, PLAU, PLXNA1, ROBO1, NRP2, SLITRK1, SORBS2, BMP4, TIMP3 |  |
| Ossification | 7 | 4 | 6.15E-03 | 0.016 | | MEF2C, NOG, CSF1, CDH11, FAT4, BMP4, BMP1 |  |
| Long-Chain Fatty Acid Metabolic Process | 6 | 4 | 6.22E-03 | 0.013 | | PTGES, TBXAS1, CPT1A, CYP2J2, SCP2, ACSL1 |  |
| Positive Regulation Of Protein Ubiquitination | 6 | 4 | 6.28E-03 | 0.012 | | ARRB1, ARRDC4, TSPYL5, PRICKLE1, PTPN22, TRIB2 |  |
| Regulation Of Blood Coagulation | 5 | 5 | 6.28E-03 | 0.012 | | PLAU, SERPINB2, THBS1, F2RL1, F3 |  |
| Regulation Of Hemostasis | 5 | 5 | 6.32E-03 | 0.011 | | PLAU, SERPINB2, THBS1, F2RL1, F3 |  |
| Coagulation | 8 | 3 | 6.32E-03 | 0.011 | | GATA6, PLAU, ITGB3, C4BPB, F2RL1, F3, F2RL2, F13A1 |  |
| Regulation Of Muscle Tissue Development | 8 | 3 | 6.42E-03 | 0.014 | | CDON, MEF2C, GATA6, NOG, GJA1, BMP4, EFNB2, TGFBR3 |  |
| Cellular Protein Metabolic Process | 69 | 1 | 6.42E-03 | 0.014 | | PLK2, ARRB1, ARRDC4, ABCA1, MGAT4A, FBXO32, MARCH3, CHRM3, BMP4, IL24, PRSS23, IRAK3, DSP, TRIM6, CSF1, FGF13, FLG, PTPN22, EFNB2, EPHA7, EPHA3, EYA4, F13A1, EFEMP1, GATA6, TTLL11, IGFBP4, IL1B, ZDHHC15, TRIB2, SH3RF2, MEF2C, MAP3K1, MMP1, CPA4, NRK, LTBP1, BCL11A, SHC3, SFTA3, HECTD4, MAMDC2, PIK3CG, RRBP1, KLHL4, CCL2, LAMA1, PTPRS, CPPED1, MARCH4, SDC2, PRDM9, PRDM8, FKBP10, TGFB2, THBS1, TGFBR3, TGM2, TLR3, HIST2H4B, PORCN, CDK15, CPEB4, CDC42BPA, CASK, GMFG, B4GALT6, CTDSPL, NUAK1 |  |
| Positive Regulation Of Reactive Oxygen Species Metabolic Process | 6 | 4 | 6.50E-03 | 0.016 | | MIR181B1, PLAU, IL1B, CLU, THBS1, F2RL1 |  |
| Cellular Response To Interferon-Gamma | 6 | 4 | 6.52E-03 | 0.012 | | HLA-A, GBP1, GBP2, GBP3, CCL2, TLR3 |  |
| Hemostasis | 8 | 3 | 6.52E-03 | 0.012 | | GATA6, PLAU, ITGB3, C4BPB, F2RL1, F3, F2RL2, F13A1 |  |
| Signal Transduction Involved In Cellular Response To Ammonium Ion | 3 | 10 | 6.60E-03 | 0.014 | | CHRM3, PLCB1, RGS10 |  |
| Regulation Of Endothelial Cell Chemotaxis | 3 | 10 | 6.74E-03 | 0.008 | | VEGFA, THBS1, SNAI2 |  |
| Acetylcholine Receptor Signaling Pathway | 3 | 10 | 6.74E-03 | 0.008 | | CHRM3, PLCB1, RGS10 |  |
| Blood Vessel Endothelial Cell Migration | 3 | 10 | 6.74E-03 | 0.008 | | ROBO1, VEGFA, EFNB2 |  |
| Positive Regulation Of Myoblast Differentiation | 3 | 10 | 6.74E-03 | 0.008 | | CDON, MEF2C, PLCB1 |  |
| Regulation Of Sterol Transport | 4 | 6 | 6.74E-03 | 0.008 | | SCP2, ABCA1, PLTP, LIPG |  |
| Regulation Of Cholesterol Transport | 4 | 6 | 6.76E-03 | 0.010 | | SCP2, ABCA1, PLTP, LIPG |  |
| Regulation Of Muscle Organ Development | 8 | 3 | 6.76E-03 | 0.010 | | CDON, MEF2C, GATA6, NOG, GJA1, BMP4, EFNB2, TGFBR3 |  |
| Negative Regulation Of Protein Kinase Activity | 9 | 3 | 6.78E-03 | 0.014 | | PDCD4, GMFG, PKIA, PTPN22, IL1B, TRIB2, GPRC5A, BMP4, IRAK3 |  |
| Negative Regulation Of Map Kinase Activity | 5 | 5 | 6.88E-03 | 0.015 | | PDCD4, PTPN22, IL1B, BMP4, IRAK3 |  |
| Positive Regulation Of Binding | 8 | 3 | 6.93E-03 | 0.011 | | PLK2, ARRB1, MEF2C, TRIM6, CLIC2, BMP4, TGFB2, HES1 |  |
| Positive Regulation Of Cell-Cell Adhesion | 10 | 3 | 6.97E-03 | 0.014 | | ZMIZ1, HAS2, NCKAP1L, HLA-A, PTPN22, IL1B, CCL2, EFNB2, FOXA1, HES1 |  |
| Cellular Response To Peptide | 10 | 3 | 7.00E-03 | 0.015 | | GRB14, MMP1, PDE3B, IL1RL1, PLCB1, PTPN22, INHBB, ITGB3, CCND2, FBP1 |  |
| Activation Of Jnkk Activity | 2 | 20 | 7.00E-03 | 0.015 | | MAP3K1, NRK |  |
| Nephric Duct Morphogenesis | 2 | 20 | 7.02E-03 | 0.005 | | EFNB2, EPHA7 |  |
| Negative Regulation Of Metanephros Development | 2 | 20 | 7.02E-03 | 0.005 | | WT1, BMP4 |  |
| Desmosome Organization | 2 | 20 | 7.02E-03 | 0.005 | | DSP, SNAI2 |  |
| Cardiac Ventricle Formation | 2 | 20 | 7.02E-03 | 0.005 | | SOX4, MEF2C |  |
| Regulation Of Atrial Cardiac Muscle Cell Membrane Depolarization | 2 | 20 | 7.02E-03 | 0.005 | | SCN5A, GJA1 |  |
| Thrombin-Activated Receptor Signaling Pathway | 2 | 20 | 7.02E-03 | 0.005 | | F2RL1, F2RL2 |  |
| Regulation Of Fear Response | 2 | 20 | 7.02E-03 | 0.005 | | MEF2C, GJA1 |  |
| Regulation Of Cgmp-Mediated Signaling | 2 | 20 | 7.02E-03 | 0.005 | | PDE10A, THBS1 |  |
| Atrial Cardiac Muscle Cell Action Potential | 2 | 20 | 7.02E-03 | 0.005 | | SCN5A, GJA1 |  |
| Vascular Smooth Muscle Cell Development | 2 | 20 | 7.02E-03 | 0.005 | | VEGFA, HES1 |  |
| Regulation Of Behavioral Fear Response | 2 | 20 | 7.02E-03 | 0.005 | | MEF2C, GJA1 |  |
| Regulation Of Peptidyl-Cysteine S-Nitrosylation | 2 | 20 | 7.02E-03 | 0.005 | | DMD, ATP2B4 |  |
| Regulation Of Immature T Cell Proliferation In Thymus | 2 | 20 | 7.02E-03 | 0.005 | | IL1B, BMP4 |  |
| Negative Regulation Of T Cell Differentiation In Thymus | 2 | 20 | 7.02E-03 | 0.005 | | ZNF608, BMP4 |  |
| Interleukin-1 Secretion | 2 | 20 | 7.02E-03 | 0.005 | | ABCA1, F2RL1 |  |
| Phospholipid Homeostasis | 2 | 20 | 7.02E-03 | 0.005 | | ABCA1, LIPG |  |
| Regulation Of Lipid Localization | 7 | 4 | 7.02E-03 | 0.005 | | ABCA1, PLTP, LIPG, IL1B, ITGB3, SCP2, THBS1 |  |
| Positive Regulation Of Cell-Matrix Adhesion | 4 | 6 | 7.05E-03 | 0.013 | | DMD, VEGFA, ITGB3, CSF1 |  |
| Cholesterol Transport | 4 | 6 | 7.18E-03 | 0.010 | | CLU, SCP2, ABCA1, LIPG |  |
| Regulation Of Endocrine Process | 4 | 6 | 7.18E-03 | 0.010 | | IL1B, INHBB, KISS1, F2RL1 |  |
| Response To Nutrient Levels | 18 | 2 | 7.18E-03 | 0.010 | | ABCA1, IL1B, INHBB, CPEB4, HMGCS1, CYP24A1, MEF2C, MMP1, CTSD, LIPG, LTBP1, BCL11A, OPTN, VEGFA, GDAP1, TGFB2, TIMP3, ACSL1 |  |
| Epithelial Cell Development | 7 | 4 | 7.19E-03 | 0.017 | | DMD, HEG1, CXCR4, WT1, BMP4, PTPRS, F2RL1 |  |
| Leukocyte Chemotaxis | 7 | 4 | 7.27E-03 | 0.013 | | NCKAP1L, CXCR4, PIK3CG, IL1B, VEGFA, CCL2, TGFB2 |  |
| Positive Regulation Of Mononuclear Cell Proliferation | 7 | 4 | 7.27E-03 | 0.013 | | MEF2C, NCKAP1L, HLA-A, CSF1, PTPN22, IL1B, HES1 |  |
| Reproductive Structure Development | 12 | 2 | 7.27E-03 | 0.013 | | GATA6, GJA1, INHBB, VEGFA, WT1, BMP4, HMGCS1, TGFB2, HES1, TLR3, CCND2, TNFSF10 |  |
| Lipid Transport | 10 | 3 | 7.27E-03 | 0.016 | | ATP8B2, ABCA1, APOL6, CPT1A, PLTP, LIPG, PITPNC1, CLU, SCP2, ACSL1 |  |
| Regulation Of Macrophage Chemotaxis | 3 | 9 | 7.32E-03 | 0.015 | | MTUS1, THBS1, CSF1 |  |
| Regulation Of Macrophage Derived Foam Cell Differentiation | 3 | 9 | 7.39E-03 | 0.008 | | ITGB3, ABCA1, CSF1 |  |
| Oligodendrocyte Development | 3 | 9 | 7.39E-03 | 0.008 | | LPAR1, KCNQ3, PRDM8 |  |
| Cell Differentiation Involved In Kidney Development | 3 | 9 | 7.39E-03 | 0.008 | | MEF2C, WT1, BMP4 |  |
| Endocrine Pancreas Development | 3 | 9 | 7.39E-03 | 0.008 | | SOX4, CLU, PDE3B |  |
| Activation Of Mapk Activity | 7 | 4 | 7.39E-03 | 0.008 | | ARRB1, MAP3K1, CXCR4, IL1B, LPAR1, TGFA, THBS1 |  |
| Regulation Of Calcium Ion Transport Into Cytosol | 6 | 4 | 7.49E-03 | 0.013 | | CEMIP, DMD, ITGB3, CLIC2, BMP4, TGFB2 |  |
| Positive Regulation Of Lipid Localization | 5 | 5 | 7.55E-03 | 0.012 | | ABCA1, PLTP, LIPG, IL1B, SCP2 |  |
| Positive Regulation Of Leukocyte Cell-Cell Adhesion | 9 | 3 | 7.58E-03 | 0.011 | | ZMIZ1, HAS2, NCKAP1L, HLA-A, PTPN22, IL1B, CCL2, EFNB2, HES1 |  |
| Transmembrane Receptor Protein Serine/Threonine Kinase Signaling Pathway | 8 | 3 | 7.58E-03 | 0.015 | | PDCD4, MAP3K1, NOG, LTBP1, INHBB, BMP4, TGFB2, TGFBR3 |  |
| Positive Regulation Of Proteolysis | 12 | 2 | 7.75E-03 | 0.014 | | PLK2, ARRB1, PRICKLE1, ROBO1, IL1B, CARD8, TRIB2, CLU, IL24, F3, TNFSF10, CRADD |  |
| Regulation Of Mononuclear Cell Proliferation | 9 | 3 | 7.82E-03 | 0.016 | | TNFRSF21, MEF2C, NCKAP1L, HLA-A, CSF1, PTPN22, IL1B, BMP4, HES1 |  |
| Negative Regulation Of Immune Response | 7 | 3 | 7.94E-03 | 0.014 | | HLA-A, IL1RL1, IL13RA2, SAMSN1, TGFB2, C4BPB, IRAK3 |  |
| Positive Regulation Of Cysteine-Type Endopeptidase Activity | 7 | 3 | 7.96E-03 | 0.013 | | ARRB1, ROBO1, CARD8, IL24, F3, TNFSF10, CRADD |  |
| Cell Junction Assembly | 7 | 3 | 7.96E-03 | 0.013 | | HEG1, LAMA3, GJA1, POF1B, PARD6B, CDH11, ITGB3 |  |
| Regulation Of Supramolecular Fiber Organization | 12 | 2 | 7.96E-03 | 0.013 | | GMFG, MEF2C, MAP3K1, NCKAP1L, NAV3, FGF13, BAIAP2L1, CLU, LPAR1, GPR65, COLGALT1, F2RL1 |  |
| Vasculature Development | 4 | 6 | 7.97E-03 | 0.016 | | VEGFA, HEG1, BMP4, HES1 |  |
| Negative Regulation Of Cytokine-Mediated Signaling Pathway | 4 | 6 | 8.07E-03 | 0.009 | | ROBO1, CARD8, F2RL1, IRAK3 |  |
| Response To Nitric Oxide | 3 | 9 | 8.07E-03 | 0.009 | | MMP3, BMP4, THBS1 |  |
| Motor Neuron Axon Guidance | 3 | 9 | 8.08E-03 | 0.008 | | NOG, EPHA3, CXCR4 |  |
| Cellular Response To Lipoprotein Particle Stimulus | 3 | 9 | 8.08E-03 | 0.008 | | ABCA1, HMGCS1, F3 |  |
| Positive Regulation Of Positive Chemotaxis | 3 | 9 | 8.08E-03 | 0.008 | | VEGFA, F2RL1, F3 |  |
| Regulation Of Metabolic Process | 132 | 1 | 8.08E-03 | 0.008 | | ARRB1, ANKRD30A, ATP2B4, ARRDC4, BCL2A1, AADAC, ABCA1, TSPYL5, CLU, BMP4, C4BPB, CCND2, DMD, TRIM6, PIK3AP1, CPT1A, CSF1, CST4, CYP2J2, FGF13, FOXR2, LPAR1, EPHA7, EPHA3, EYA4, FAM172BP, F2RL1, F3, ACSL1, EFEMP1, FBP1, HAS2, NCKAP1L, GATA6, GBP1, PRICKLE1, GJA1, IGFBP4, IL1B, INHBB, ITGB3, HMGCS1, FOXA1, HES1, SH3RF2, MEF2C, MEIS3P1, MAP3K1, MMP3, NRK, SAMD9L, KISS1, LTBP1, SERPINB2, ZNF438, NEO1, PKIA, PDE3B, PIK3CG, PLAU, ADGRF1, SSX8, ROBO1, SCP2, CCL2, HMSD, SOX4, SSX1, TCF4, SNAI2, VEGFA, WT1, TGFA, TGFB2, THBS1, TGFBR3, TIMP3, TLR3, ZNF391, HIST2H4B, ZNF43, CXCR4, GPRC5A, CST7, CASK, SAMD5, SERPINB7, TNFSF10, CRADD, GMFG, PDLIM1, NOG, LIPG, RAD50, OPTN, MBNL2, CTDSPL, NUAK1, PLK2, ZNF716, MIR181B1, CARD8, IL24, IRAK3, SP140, TDRD7, ARL2BP, PLCB1, PTPN22, TIAM2, TNFRSF21, PDCD4, PCDH11X, UCA1, TRIB2, CDON, MIR604, MAFIP, KCNIP3, G0S2, BCL11A, LMO3, ZMIZ1, CEMIP, ZNF608, PMEPA1, PRDM9, PRDM8, SAMSN1, NXN, CPEB4, AFAP1L2 |  |
| Regulation Of Protein Ubiquitination | 8 | 3 | 8.12E-03 | 0.015 | | SOX4, ARRB1, ARRDC4, TSPYL5, PRICKLE1, PTPN22, TRIB2, NXN |  |
| Negative Regulation Of Cellular Component Movement | 12 | 2 | 8.17E-03 | 0.014 | | ARHGDIB, MEF2C, KISS1, NOG, PLCB1, NAV3, ROBO1, SCP2, CCL2, IL24, THBS1, TGFBR3 |  |
| Regulation Of Cell Junction Assembly | 5 | 5 | 8.26E-03 | 0.016 | | GJA1, SNAI2, VEGFA, THBS1, EPHA3 |  |
| Regulation Of Coagulation | 5 | 5 | 8.26E-03 | 0.011 | | PLAU, SERPINB2, THBS1, F2RL1, F3 |  |
| Muscle System Process | 10 | 3 | 8.26E-03 | 0.011 | | MEF2C, DMD, MYL9, GATA6, GJA1, CXCR4, IL1B, FBXO32, CHRM3, SCN5A |  |
| Regulation Of Extent Of Cell Growth | 6 | 4 | 8.32E-03 | 0.015 | | DPYSL2, FGF13, BCL11A, VEGFA, PTPRS, EPHA7 |  |
| Positive Regulation Of Hormone Secretion | 7 | 3 | 8.39E-03 | 0.012 | | SOX4, ARRB1, KISS1, GJA1, IL1B, INHBB, CASK |  |
| Heat Generation | 2 | 18 | 8.44E-03 | 0.013 | | IL1B, EPHA3 |  |
| Regulation Of Glomerular Filtration | 2 | 18 | 8.51E-03 | 0.005 | | GJA1, F2RL1 |  |
| Ether Lipid Metabolic Process | 2 | 18 | 8.51E-03 | 0.005 | | HRASLS, AGMO |  |
| Regulation Of Alkaline Phosphatase Activity | 2 | 18 | 8.51E-03 | 0.005 | | MEF2C, TGFB2 |  |
| Smooth Muscle Cell Migration | 2 | 18 | 8.51E-03 | 0.005 | | ITGB3, PLAU |  |
| Corpus Callosum Development | 2 | 18 | 8.51E-03 | 0.005 | | LPAR1, PTPRS |  |
| Positive Regulation Of Chemokine Biosynthetic Process | 2 | 18 | 8.51E-03 | 0.005 | | IL1B, TLR3 |  |
| Regulation Of Toll-Like Receptor 3 Signaling Pathway | 2 | 18 | 8.51E-03 | 0.005 | | PTPN22, F2RL1 |  |
| Response To Luteinizing Hormone | 2 | 18 | 8.51E-03 | 0.005 | | SCP2, TGFBR3 |  |
| Regulation Of Protein Heterodimerization Activity | 2 | 18 | 8.51E-03 | 0.005 | | CDON, HES1 |  |
| Limb Bud Formation | 2 | 18 | 8.51E-03 | 0.005 | | SOX4, PLXNA2 |  |
| Regulation Of Interleukin-12 Production | 4 | 6 | 8.51E-03 | 0.005 | | THBS1, TLR3, PLCB1, IRAK3 |  |
| Regulation Of Collagen Metabolic Process | 4 | 6 | 8.54E-03 | 0.009 | | BMP4, SERPINB7, LTBP1, CYP2J2 |  |
| Cardiocyte Differentiation | 4 | 6 | 8.54E-03 | 0.009 | | MEF2C, BMP4, TGFB2, GATA6 |  |
| Myeloid Cell Differentiation | 8 | 3 | 8.54E-03 | 0.009 | | MEF2C, SNX10, CSF1, PLCB1, VEGFA, BMP4, TGFBR3, F2RL1 |  |
| Regulation Of Chemokine Production | 5 | 5 | 8.60E-03 | 0.014 | | IL1RL1, SNAI2, IL1B, TLR3, F2RL1 |  |
| Leukocyte Activation Involved In Immune Response | 16 | 2 | 8.62E-03 | 0.011 | | NCKAP1L, PIK3CG, PLAU, CD33, CD68, ADGRE5, CPPED1, DOCK10, SLC44A2, GMFG, CRISPLD2, DSP, LCP1, CTSD, STOM, F2RL1 |  |
| Negative Regulation Of Glial Cell Differentiation | 3 | 9 | 8.66E-03 | 0.016 | | BMP4, NOG, HES1 |  |
| Regulation Of Positive Chemotaxis | 3 | 9 | 8.80E-03 | 0.008 | | VEGFA, F2RL1, F3 |  |
| Cellular Ion Homeostasis | 18 | 2 | 8.80E-03 | 0.008 | | ATP2B4, GJA1, CXCR4, PIK3CG, EFHC1, IL1B, ITGB3, CCL2, GPR65, BMP4, CEMIP, TBXAS1, KISS1, LPAR1, S1PR3, TGM2, F2RL1, F2RL2 |  |
| Cellular Homeostasis | 23 | 2 | 8.87E-03 | 0.016 | | ATP2B4, GJA1, CXCR4, PIK3CG, PLAU, EFHC1, IL1B, ITGB3, CCL2, GPR65, BMP4, SOX4, MEF2C, CEMIP, DMD, TBXAS1, KISS1, NXN, LPAR1, S1PR3, TGM2, F2RL1, F2RL2 |  |
| Fatty Acid Derivative Metabolic Process | 7 | 3 | 8.94E-03 | 0.017 | | PON2, PTGES, TBXAS1, CYP2J2, ELOVL7, PTGR1, ACSL1 |  |
| Neurogenesis | 5 | 5 | 8.95E-03 | 0.013 | | CXCR4, PRDM8, LPAR1, FAT4, TGFB2 |  |
| Regulation Of Camp-Mediated Signaling | 4 | 6 | 8.99E-03 | 0.011 | | ATP2B4, PDE10A, LPAR1, PDE3B |  |
| Negative Regulation Of Peptidase Activity | 11 | 2 | 9.02E-03 | 0.009 | | ARRB1, BCL2A1, CARD8, VEGFA, SERPINB2, HMSD, IL24, CST7, THBS1, TIMP3, SERPINB7 |  |
| Cell Activation Involved In Immune Response | 16 | 2 | 9.06E-03 | 0.015 | | NCKAP1L, PIK3CG, PLAU, CD33, CD68, ADGRE5, CPPED1, DOCK10, SLC44A2, GMFG, CRISPLD2, DSP, LCP1, CTSD, STOM, F2RL1 |  |
| Cellular Response To Abiotic Stimulus | 12 | 2 | 9.16E-03 | 0.016 | | MAP3K1, TSPYL5, GJA1, SNAI2, LTBP1, IL1B, IL13RA2, ITGB3, BMP4, TLR3, CCND2, CRADD |  |
| Cellular Response To Environmental Stimulus | 12 | 2 | 9.19E-03 | 0.016 | | MAP3K1, TSPYL5, GJA1, SNAI2, LTBP1, IL1B, IL13RA2, ITGB3, BMP4, TLR3, CCND2, CRADD |  |
| Positive Regulation Of Establishment Of Protein Localization | 14 | 2 | 9.19E-03 | 0.016 | | ARRB1, GJA1, IL1B, CARD8, CASK, SOX4, CEMIP, TRIM6, IL1RL1, CYP2J2, PTPN22, TGFB2, STOM, F2RL1 |  |
| Skeletal System Development | 8 | 3 | 9.22E-03 | 0.016 | | SOX4, NOG, IGFBP4, CDH11, BMP4, BMP1, TGFB2, TLL1 |  |
| Negative Regulation Of Cell Growth | 8 | 3 | 9.28E-03 | 0.014 | | GJA1, FGF13, BCL11A, WT1, PTPRS, TGFB2, EPHA7, FBP1 |  |
| Regulation Of Hemopoiesis | 14 | 2 | 9.28E-03 | 0.014 | | HIST2H4B, NCKAP1L, MYL9, IL1B, ITGB3, BMP4, HES1, ZMIZ1, MEF2C, ZNF608, CSF1, VEGFA, THBS1, TLR3 |  |
| Positive Regulation Of Blood Circulation | 5 | 5 | 9.37E-03 | 0.016 | | TBXAS1, GJA1, SCN5A, TGFB2, F2RL1 |  |
| Brain Development | 11 | 2 | 9.37E-03 | 0.011 | | DPYSL2, NOG, PLCB1, CXCR4, AK4, ADGRL3, TSPAN2, LPAR1, BMP4, HMGCS1, EPHA7 |  |
| Regulation Of Immune Response | 28 | 2 | 9.41E-03 | 0.015 | | BCL2A1, CD33, CLU, HMSD, PTPRS, C4BPB, IRAK3, TRIM6, PIK3AP1, PTPN22, SAMSN1, TGFB2, SLAMF7, TLR3, F2RL1, TNFRSF21, NCKAP1L, HLA-A, HLA-B, GBP1, IL1B, IL13RA2, HLA-DRA, MEF2C, MAP3K1, IL1RL1, PLPP4, CLEC2B |  |
| Ovarian Follicle Development | 4 | 6 | 9.45E-03 | 0.017 | | INHBB, VEGFA, BMP4, CCND2 |  |
| Positive Regulation Of Heart Growth | 4 | 6 | 9.53E-03 | 0.009 | | MEF2C, WT1, GATA6, TGFBR3 |  |
| Sprouting Angiogenesis | 3 | 8 | 9.53E-03 | 0.009 | | VEGFA, THBS1, ESM1 |  |
| Cardiac Ventricle Morphogenesis | 3 | 8 | 9.56E-03 | 0.008 | | SOX4, BMP4, TGFB2 |  |
| Response To Follicle-Stimulating Hormone | 3 | 8 | 9.56E-03 | 0.008 | | HMGCS1, TGFBR3, EPHA3 |  |
| Notch Signaling Pathway | 6 | 4 | 9.56E-03 | 0.008 | | SNAI2, S1PR3, SORBS2, FAT4, FOXA1, HES1 |  |
| Regulation Of Cell Growth | 14 | 2 | 9.61E-03 | 0.012 | | GJA1, CXCR4, IGFBP4, PTPRS, ESM1, HRASLS, DPYSL2, FGF13, BCL11A, VEGFA, WT1, TGFB2, EPHA7, FBP1 |  |
| Negative Regulation Of Endopeptidase Activity | 10 | 3 | 9.81E-03 | 0.016 | | ARRB1, BCL2A1, CARD8, VEGFA, SERPINB2, HMSD, IL24, THBS1, TIMP3, SERPINB7 |  |
| Positive Regulation Of Endocytosis | 7 | 3 | 1.00E-02 | 0.015 | | ARRB1, NCKAP1L, IL1B, VEGFA, CLU, CCL2, F2RL1 |  |
| Homeostasis Of Number Of Cells | 7 | 3 | 1.00E-02 | 0.013 | | MEF2C, ILDR2, NCKAP1L, BCL2A1, CSF1, VEGFA, DOCK10 |  |
| Collagen Metabolic Process | 4 | 6 | 1.00E-02 | 0.013 | | MMP3, MMP1, TGFBR3, CTSD |  |
| Regulation Of Endothelial Cell Apoptotic Process | 4 | 6 | 1.01E-02 | 0.009 | | PDCD4, CCL2, BMP4, THBS1 |  |
| Negative Regulation Of Response To Cytokine Stimulus | 4 | 6 | 1.01E-02 | 0.009 | | ROBO1, CARD8, F2RL1, IRAK3 |  |
| Response To Peptide Hormone | 13 | 2 | 1.01E-02 | 0.009 | | GRB14, PDE3B, GJA1, IL1B, INHBB, ITGB3, SCP2, CCND2, TNFSF10, MMP1, PLCB1, TGFBR3, FBP1 |  |
| Lymphoid Progenitor Cell Differentiation | 2 | 16 | 1.01E-02 | 0.016 | | SOX4, BMP4 |  |
| Positive Regulation Of Tolerance Induction | 2 | 16 | 1.01E-02 | 0.005 | | HLA-A, IRAK3 |  |
| Cardiac Chamber Formation | 2 | 16 | 1.01E-02 | 0.005 | | SOX4, MEF2C |  |
| **Enrichment for Cellular Components** | | | | | | |  |
| Membrane | 194 | 1 | 5.51E-10 | 0.018 | | ARHGDIB, ARRB1, ATP2B4, ARRDC4, BCL2A1, AADAC, ABCA1, NAV3, CD22, CD33, ENTPD3, CD68, ADGRE5, CDH11, SLITRK1, MARCH3, CHRM3, TLCD1, CLU, C4BPB, SYTL3, CCND2, CYP24A1, DMD, DPYSL2, OR2M3, DSP, PIK3AP1, CPT1A, OR5P2, CSF1, CTSD, CYP2J2, TMEM171, FGF13, FLG, LPAR1, S1PR3, EFNB2, STOM, EPHA7, EPHA3, LYPD6B, F2RL1, F3, F2RL2, ACSL1, GPR1, GRB14, TMEM56, HAS2, NCKAP1L, HLA-A, HLA-B, GATA6, GBP1, GBP2, GBP3, PRICKLE1, GJA1, LRFN5, GNGT2, ZDHHC15, IL13RA2, ITGB3, TOR1AIP2, CD82, HLA-DRA, SHISA3, MAP3K1, TMEM154, KCNQ3, KISS1, LCP1, MPZL3, PLPP4, METTL7B, SFTA3, SERPINB2, SLC37A2, OR5B21, NEO1, FAM83B, PON2, CD163L1, TMPRSS15, PDE3B, MAMDC2, PGM5, PIK3CG, PLAU, ADGRF1, PLXNA2, PLXNA1, ROBO1, RRBP1, SCN5A, SCP2, SCN9A, LAMA1, PTPRS, RAB27B, TBXAS1, SDC2, SLC22A3, UGCG, VEGFA, TGFA, TGFBR3, TGM2, TLR3, SERINC2, SLC7A5, ILDR2, SHISA2, HIST2H4B, SRPX, LAPTM5, CXCR4, NRP2, CLIC3, GPRC5A, SORBS2, GPR65, LGR5, CASK, TNFSF10, AGMO, PTGES, FCGR2C, IL1RL1, B4GALT6, TSPAN2, RAD50, OPTN, CLEC2B, PDE10A, MGAT4A, CNKSR2, IRAK3, SEL1L3, PLCB1, ADGRL3, TBC1D30, TIAM2, OR2M4, FLRT2, TNFRSF21, PCDH11X, OR2H1, STXBP6, CDON, SNX10, KCNIP3, ASAP1, SHC3, CNTN5, CLIC6, GDAP1, CHST15, SNX7, LPCAT2, TENM3, MCTP2, BAIAP2L1, CPPED1, DOCK10, HRASLS, SLC44A2, ATP8B2, CEMIP, HEG1, MTUS1, SORCS2, MARCH4, PMEPA1, FKBP10, SAMSN1, HHIP, CACHD1, SLAMF7, MPP4, GPR135, PORCN, ELOVL7, MYCT1, CPEB4, FAT4, VEPH1, COLGALT1, CLMP, PCDH11Y, PARD6B, AFAP1L2 |  |
| Extracellular Matrix | 25 | 5 | 7.46E-10 | 0.032 | | CDON, ADAMTS12, CRISPLD2, MMP3, MMP1, SDC2, MAMDC2, IL1RL1, LAMA3, LAMA4, CTSD, LTBP1, VEGFA, CLU, LAMA1, FLRT2, TGFB2, THBS1, TGFBR3, TGM2, CASK, TIMP3, ABI3BP, F3, EFEMP1 | |
| Plasma Membrane Part | 87 | 2 | 3.23E-09 | 0.022 | | ARRB1, ATP2B4, ABCA1, CD22, CD33, ADGRE5, CDH11, CNKSR2, CHRM3, SYTL3, DMD, DSP, ADGRL3, FGF13, PCDHB5, PTPN22, LPAR1, S1PR3, FLRT2, EFNB2, STOM, EPHA7, EPHA3, F2RL1, F3, F2RL2, GPR1, TNFRSF21, PCDH17, PCDH11X, HAS2, NCKAP1L, HLA-A, HLA-B, GJA1, GNGT2, IL13RA2, ITGB3, CD82, HLA-DRA, CDON, KCNQ3, KISS1, LCP1, KCNIP3, ASAP1, PLPP4, NEO1, CD163L1, PGM5, PLXNA2, PLXNA1, TENM3, ROBO1, SCN5A, SCN9A, PCDHB6, PTPRS, RAB27B, HEG1, PCDHB2, SLC22A3, HHIP, TGFA, THBS1, TGFBR3, TGM2, PCDHB16, SLAMF7, TLR3, MPP4, SLC7A5, PORCN, LAPTM5, CXCR4, CPEB4, GPRC5A, SORBS2, GPR65, LGR5, CASK, TNFSF10, PCDH11Y, IL1RL1, TSPAN2, PARD6B, CLEC2B | |
| Extracellular Region | 70 | 2 | 3.89E-09 | 0.024 | | PON2, OLFML1, CD163L1, MAMDC2, BAGE4, PLAU, ADGRF1, PLTP, MGAT4A, SLITRK1, CLU, CCL2, LAMA1, BMP4, BMP1, DMKN, IL24, CPPED1, C4BPB, ESM1, PRSS23, CEMIP, HEG1, CSF1, CTSD, FGF13, TUFT1, VEGFA, HHIP, FLRT2, TGFA, TGFB2, THBS1, TGFBR3, TIMP3, EPHA3, TLL1, LYPD6B, ABI3BP, F2RL2, F13A1, EFEMP1, HIST2H4B, GBP1, GBP3, IGFBP4, NRP2, IL1B, IL13RA2, INHBB, CST7, TNFSF10, ADAMTS12, GMFG, CRISPLD1, CRISPLD2, MMP3, MMP1, CPA4, APOL6, IL1RL1, KISS1, NOG, LAMA3, LAMA4, LIPG, LTBP1, CNTN5, SFTA3, SERPINB2 | |
| Collagen-Containing Extracellular Matrix | 19 | 6 | 4.29E-09 | 0.030 | | CDON, ADAMTS12, SDC2, MAMDC2, LAMA3, LAMA4, CTSD, LTBP1, VEGFA, CLU, LAMA1, TGFB2, THBS1, TGM2, CASK, TIMP3, ABI3BP, F3, EFEMP1 | |
| Intrinsic Component Of Plasma Membrane | 55 | 2 | 7.83E-09 | 0.025 | | ATP2B4, ABCA1, PLXNA2, PLXNA1, CD22, CD33, TENM3, ROBO1, ADGRE5, CHRM3, SCN9A, PCDHB6, PTPRS, PCDHB2, ADGRL3, SLC22A3, PCDHB5, HHIP, LPAR1, S1PR3, FLRT2, EFNB2, TGFA, TGFBR3, TGM2, PCDHB16, STOM, EPHA7, EPHA3, TLR3, F2RL1, F3, F2RL2, GPR1, TNFRSF21, PCDH17, PCDH11X, HAS2, NCKAP1L, LAPTM5, GJA1, ITGB3, GPRC5A, CD82, GPR65, HLA-DRA, LGR5, TNFSF10, CDON, PCDH11Y, KCNQ3, PLPP4, TSPAN2, CLEC2B, NEO1 | |
| Integral Component Of Plasma Membrane | 53 | 2 | 9.89E-09 | 0.025 | | ATP2B4, ABCA1, PLXNA2, PLXNA1, CD22, CD33, TENM3, ROBO1, ADGRE5, CHRM3, SCN9A, PCDHB6, PTPRS, PCDHB2, ADGRL3, SLC22A3, PCDHB5, HHIP, LPAR1, S1PR3, FLRT2, EFNB2, TGFA, TGFBR3, PCDHB16, STOM, EPHA7, EPHA3, TLR3, F2RL1, F2RL2, GPR1, TNFRSF21, PCDH17, PCDH11X, HAS2, NCKAP1L, LAPTM5, GJA1, ITGB3, GPRC5A, CD82, GPR65, HLA-DRA, LGR5, TNFSF10, CDON, PCDH11Y, KCNQ3, PLPP4, TSPAN2, CLEC2B, NEO1 | |
| Extracellular Region Part | 96 | 2 | 1.27E-08 | 0.021 | | ARHGDIB, ARRDC4, MIR181B1, ABCA1, CD22, MGAT4A, ADGRE5, CDH11, CNKSR2, PTGR1, CLU, BMP4, BMP1, DMKN, IL24, C4BPB, PRSS23, DPYSL2, DSP, CSF1, PLCB1, CST4, CTSD, CYP2J2, TIAM2, FLRT2, STOM, ABI3BP, F3, F13A1, EFEMP1, FBP1, NCKAP1L, HLA-A, GBP1, GBP2, GBP3, IGFBP4, IL1B, IL13RA2, INHBB, ITGB3, CD82, HLA-DRA, CDON, MMP3, MMP1, CPA4, KISS1, LAMA3, LAMA4, LCP1, LTBP1, CLIC6, SERPINB2, SLC37A2, MAMDC2, PLAU, PLTP, PLXNA1, BAIAP2L1, SCP2, CCL2, LAMA1, PTPRS, RAB27B, DOCK10, SLC44A2, MTUS1, SDC2, VEGFA, TGFA, TGFB2, THBS1, TGFBR3, TGM2, TIMP3, SERINC2, SLC7A5, HIST2H4B, CXCR4, CLIC3, GPRC5A, FAT4, CDC42BPA, CASK, SERPINB7, TNFSF10, ADAMTS12, CRISPLD1, CRISPLD2, IL1RL1, NOG, LIPG, PARD6B, CTDSPL | |
| Cell Surface | 31 | 4 | 2.46E-08 | 0.028 | | SRPX, HLA-A, HLA-B, ABCA1, CXCR4, PLAU, LRFN5, ROBO1, CDH11, ITGB3, CLU, SCN5A, HLA-DRA, CLMP, CDON, DMD, HEG1, SDC2, KCNQ3, IL1RL1, LIPG, VEGFA, HHIP, LPAR1, TGFA, TGFB2, THBS1, TGFBR3, TLR3, F3, NEO1 | |
| Response To Organic Substance | 85 | 2 | 3.41E-08 | 0.021 | | ARHGDIB, ARRB1, ARRDC4, ABCA1, CD22, CD68, MGAT4A, ADGRE5, CDH11, CNKSR2, MARCH3, PTGR1, CLU, BMP4, BMP1, PRSS23, SYTL3, DPYSL2, DSP, PLCB1, CST4, CTSD, CYP2J2, TIAM2, LPAR1, FLRT2, STOM, EPHA3, F2RL1, EFEMP1, FBP1, GRB14, NCKAP1L, GBP1, GBP2, GBP3, GJA1, IL1B, ITGB3, CD82, HLA-DRA, SAMD9L, SNX10, LAMA3, LAMA4, LCP1, SFTA3, CLIC6, SLC37A2, SNX7, NEO1, PLAU, PLXNA1, BAIAP2L1, PTPRS, RAB27B, DOCK10, SLC44A2, CEMIP, PMEPA1, VEGFA, TGFA, TGFB2, THBS1, TGFBR3, TGM2, TLR3, SERINC2, SLC7A5, HIST2H4B, GPR135, CXCR4, CLIC3, GPRC5A, FAT4, CDC42BPA, CASK, TNFSF10, CYTIP, CRISPLD1, CRISPLD2, LIPG, PARD6B, OPTN, CTDSPL | |
| Animal Organ Morphogenesis | 160 | 1 | 4.02E-06 | 0.017 | | ARRB1, ATP2B4, AADAC, ABCA1, NAV3, CD22, CD33, ENTPD3, CD68, ADGRE5, CDH11, SLITRK1, MARCH3, CHRM3, TLCD1, CLIC2, SYTL3, DMD, OR2M3, DSP, CPT1A, OR5P2, CSF1, CTSD, CYP2J2, TMEM171, FGF13, LPAR1, S1PR3, EFNB2, STOM, EPHA7, EPHA3, LYPD6B, FAM172BP, F2RL1, F3, F2RL2, ACSL1, GPR1, TMEM56, HAS2, NCKAP1L, HLA-A, HLA-B, GJA1, LRFN5, GNGT2, ZDHHC15, IL13RA2, ITGB3, TOR1AIP2, CD82, HLA-DRA, SHISA3, TMEM154, KCNQ3, KISS1, LCP1, COX7B2, MPZL3, PLPP4, METTL7B, SFTA3, SLC37A2, OR5B21, NEO1, CD163L1, HECTD4, TMPRSS15, PDE3B, PGM5, PIK3CG, ADGRF1, PLXNA2, PLXNA1, ROBO1, SCN5A, SCP2, SCN9A, PTPRS, RAB27B, TBXAS1, SDC2, SLC22A3, UGCG, TGFA, THBS1, TGFBR3, TGM2, TLR3, SERINC2, SLC7A5, ILDR2, SHISA2, LAPTM5, CXCR4, NRP2, CLIC3, GPRC5A, SORBS2, GPR65, LGR5, CASK, TNFSF10, AGMO, PTGES, FCGR2C, IL1RL1, B4GALT6, TSPAN2, CLEC2B, MGAT4A, CNKSR2, SEL1L3, ADGRL3, PCDHB5, PTPN22, OR2M4, FLRT2, TNFRSF21, PCDH17, PCDH11X, OR2H1, STXBP6, CDON, SNX10, KCNIP3, ASAP1, CNTN5, CLIC6, GDAP1, CHST15, LPCAT2, TENM3, MCTP2, PCDHB6, HRASLS, SLC44A2, ATP8B2, CEMIP, HEG1, SORCS2, MARCH4, PCDHB2, PMEPA1, HHIP, CACHD1, PCDHB16, SLAMF7, MPP4, GPR135, PORCN, ELOVL7, MYCT1, CPEB4, FAT4, CLMP, PCDH11Y, PARD6B | |
| Cellular Response To Interleukin-1 | 53 | 2 | 4.50E-06 | 0.021 | | ARHGDIB, PLAU, PLXNA1, CD22, MGAT4A, ADGRE5, CDH11, CNKSR2, PTGR1, BAIAP2L1, CLU, PTPRS, RAB27B, PRSS23, DOCK10, SLC44A2, DPYSL2, DSP, PLCB1, CST4, CTSD, CYP2J2, TIAM2, FLRT2, THBS1, TGFBR3, TGM2, STOM, SERINC2, EFEMP1, FBP1, SLC7A5, HIST2H4B, NCKAP1L, CXCR4, IL1B, CLIC3, ITGB3, GPRC5A, CD82, HLA-DRA, FAT4, CDC42BPA, TNFSF10, CRISPLD1, CRISPLD2, LAMA3, LAMA4, LCP1, PARD6B, CTDSPL, CLIC6, SLC37A2 | |
| Protein Binding | 21 | 3 | 5.31E-06 | 0.024 | | PGM5, GJA1, CXCR4, POF1B, CDH11, BAIAP2L1, SCN5A, STXBP6, LAMA1, CDC42BPA, CASK, CLMP, HEG1, DSP, PDLIM1, LAMA3, ADGRL3, FGF13, PARD6B, FLRT2, MPP4 | |
| Positive Regulation Of Kidney Development | 132 | 1 | 2.26E-05 | 0.017 | | ATP2B4, AADAC, ABCA1, CD22, CD33, ENTPD3, CD68, ADGRE5, CDH11, SLITRK1, MARCH3, CHRM3, TLCD1, OR2M3, CPT1A, OR5P2, CSF1, TMEM171, LPAR1, S1PR3, EFNB2, STOM, EPHA7, EPHA3, LYPD6B, FAM172BP, F2RL1, F3, F2RL2, ACSL1, GPR1, TMEM56, HAS2, NCKAP1L, HLA-A, HLA-B, GJA1, LRFN5, ZDHHC15, IL13RA2, ITGB3, TOR1AIP2, CD82, HLA-DRA, SHISA3, TMEM154, KCNQ3, COX7B2, MPZL3, PLPP4, SLC37A2, OR5B21, NEO1, CD163L1, HECTD4, TMPRSS15, PDE3B, ADGRF1, PLXNA2, PLXNA1, ROBO1, SCN5A, SCN9A, PTPRS, RAB27B, TBXAS1, SDC2, SLC22A3, UGCG, TGFA, TGFBR3, TGM2, TLR3, SERINC2, SLC7A5, ILDR2, SHISA2, LAPTM5, CXCR4, NRP2, CLIC3, GPRC5A, GPR65, LGR5, CASK, TNFSF10, AGMO, PTGES, FCGR2C, IL1RL1, B4GALT6, TSPAN2, CLEC2B, MGAT4A, SEL1L3, ADGRL3, PCDHB5, OR2M4, FLRT2, TNFRSF21, PCDH17, PCDH11X, OR2H1, STXBP6, CDON, CNTN5, CLIC6, GDAP1, CHST15, LPCAT2, TENM3, MCTP2, PCDHB6, HRASLS, SLC44A2, ATP8B2, HEG1, SORCS2, MARCH4, PCDHB2, PMEPA1, HHIP, CACHD1, PCDHB16, SLAMF7, GPR135, PORCN, ELOVL7, MYCT1, FAT4, CLMP, PCDH11Y | |
| Positive Regulation Of Cellular Component Movement | 20 | 3 | 3.95E-05 | 0.022 | | TNS3, PGM5, GJA1, PLAU, POF1B, ADGRE5, CDH11, ITGB3, BAIAP2L1, STXBP6, SORBS2, CASK, DSP, PDLIM1, LAMA3, LCP1, FLRT2, EFNB2, TGM2, MPP4 | |
| Endoplasmic Reticulum | 9 | 7 | 4.10E-05 | 0.019 | | DSP, PDLIM1, PGM5, LAMA3, GJA1, CDH11, BAIAP2L1, STXBP6, MPP4 | |
| Response To Acid Chemical | 178 | 1 | 4.54E-05 | 0.016 | | ARHGDIB, ARRB1, ATP2B4, ARRDC4, BCL2A1, AADAC, ABCA1, AK4, CD33, CD68, EFHC1, ADGRE5, FBXO32, MARCH3, CLU, BMP4, BMP1, SYTL3, CCND2, CYP24A1, DMD, DPYSL2, DSP, TRIM6, PIK3AP1, CPT1A, CSF1, CTSD, CYP2J2, FGF13, FLG, LPAR1, STOM, EPHA3, F2RL1, F13A1, ACSL1, FBP1, GRB14, NCKAP1L, HLA-A, HLA-B, GALC, GBP1, GBP2, GBP3, PRICKLE1, GJA1, TTLL11, IGFBP4, IL1B, ZDHHC15, INHBB, ITGB3, TOR1AIP2, HLA-DRA, HMGCS1, SHISA3, MEF2C, MAP3K1, MMP3, SAMD9L, LAMA3, LCP1, COX7B2, LTBP1, METTL7B, SFTA3, SLC37A2, ZNF438, NEO1, PON2, PDE3B, MAMDC2, PGM5, PIK3CG, PLAU, PLCB4, RRBP1, SCN5A, SCP2, PTPRS, RAB27B, RGS10, SOX4, TBXAS1, SDC2, UGCG, VEGFA, WT1, TGFA, TGFB2, THBS1, TGFBR3, TGM2, TIMP3, TLR3, SERINC2, SLC7A5, ILDR2, SHISA2, SRPX, LAPTM5, EVI5, CXCR4, GPRC5A, SORBS2, LGR5, CASK, CRADD, AGMO, GMFG, PTGES, CYTIP, PDLIM1, B4GALT6, LIPG, RAD50, OPTN, PLK2, PDE10A, MYL9, MGAT4A, CARD8, IL24, PRSS23, SP140, TDRD7, ARL2BP, PLCB1, TBC1D30, PTPN22, PITPNC1, TIAM2, FLRT2, PDCD4, STXBP6, HDDC2, SNX10, KCNIP3, G0S2, ASAP1, SHC3, CNTN5, GDAP1, KRTAP4-8, CHST15, SNX7, LPCAT2, MCTP2, BAIAP2L1, ACOXL, CPPED1, DOCK10, HRASLS, SLC44A2, ATP8B2, CEMIP, MTUS1, MARCH4, PMEPA1, FKBP10, SAMSN1, NXN, SLAMF7, MPP4, GPR135, TNS3, PORCN, CDK15, ELOVL7, CPEB4, COLGALT1, CLMP, AKR1E2, CRISPLD2, PARD6B, AFAP1L2 | |
| Positive Regulation Of Cytosolic Calcium Ion Concentration Involved In Phospholipase C-Activating G-Protein Coupled Signaling Pathway | 41 | 2 | 4.91E-05 | 0.021 | | LPCAT2, AADAC, PDE3B, MAMDC2, PLCB4, RRBP1, CLU, SCN5A, SCP2, IL24, ATP8B2, CEMIP, TBXAS1, CYP2J2, FKBP10, FLRT2, THBS1, TGFBR3, TGM2, STOM, SLAMF7, TLR3, ACSL1, ILDR2, SHISA2, SRPX, HLA-A, HLA-B, PORCN, GJA1, ELOVL7, ZDHHC15, CPEB4, TOR1AIP2, COLGALT1, SHISA3, AGMO, SNX10, LAMA3, KCNIP3, SLC37A2 | |
| Chronic Inflammatory Response | 20 | 3 | 6.29E-05 | 0.022 | | TNS3, PGM5, GJA1, PLAU, POF1B, ADGRE5, CDH11, ITGB3, BAIAP2L1, STXBP6, SORBS2, CASK, DSP, PDLIM1, LAMA3, LCP1, FLRT2, EFNB2, TGM2, MPP4 | |
| Endoplasmic Reticulum Part | 47 | 2 | 6.61E-05 | 0.020 | | PLK2, ARRB1, ATP2B4, PLCB4, TENM3, ROBO1, EFHC1, CNKSR2, CHRM3, CLU, PTPRS, DOCK10, RGS10, DMD, ARL2BP, DPYSL2, ADGRL3, TBC1D30, FGF13, TIAM2, SAMSN1, LPAR1, FLRT2, TGFB2, EPHA7, EPHA3, F2RL1, MPP4, GPR1, TNFRSF21, TTLL11, NRP2, CPEB4, SORBS2, CDC42BPA, FOXA1, CASK, MAFIP, MMP3, KCNQ3, KISS1, NOG, LCP1, KCNIP3, LTBP1, ASAP1, OPTN | |
| Response To Vitamin | 35 | 2 | 7.56E-05 | 0.021 | | PLK2, ARRB1, ATP2B4, PLCB4, TENM3, ROBO1, CNKSR2, CHRM3, CLU, PTPRS, DOCK10, RGS10, DPYSL2, ADGRL3, FGF13, LPAR1, FLRT2, TGFB2, EPHA7, EPHA3, MPP4, GPR1, TNFRSF21, NRP2, CPEB4, SORBS2, CASK, MMP3, KCNQ3, KISS1, NOG, KCNIP3, LTBP1, ASAP1, OPTN | |
| Cytoplasmic Vesicle Part | 37 | 2 | 7.62E-05 | 0.021 | | LPCAT2, AADAC, ABCA1, MGAT4A, BMP4, PRSS23, ATP8B2, TBXAS1, SDC2, CPT1A, CSF1, CYP2J2, FKBP10, FLRT2, TGFA, THBS1, TLR3, ACSL1, SERINC2, ILDR2, SHISA2, HLA-A, HLA-B, PORCN, GJA1, IGFBP4, ELOVL7, TOR1AIP2, HLA-DRA, COLGALT1, SHISA3, AGMO, PTGES, LTBP1, METTL7B, SFTA3, SLC37A2 | |
| Kinase Binding | 38 | 2 | 9.68E-05 | 0.020 | | ARRB1, PLAU, CD33, CD68, ADGRE5, MCTP2, MARCH3, CLU, PTPRS, CPPED1, RAB27B, SLC44A2, CEMIP, DSP, PMEPA1, CTSD, VEGFA, TGFA, TGFB2, THBS1, TIMP3, STOM, TLR3, F13A1, GRB14, NCKAP1L, HLA-A, HLA-B, GPR135, GJA1, ITGB3, GPRC5A, HLA-DRA, GMFG, CRISPLD2, SNX10, OPTN, SNX7 | |
| Regulation Of Cell-Cell Adhesion | 8 | 7 | 1.04E-04 | 0.017 | | LAMA3, LAMA4, VEGFA, LAMA1, TGFB2, CASK, TIMP3, EFEMP1 | |
| Negative Regulation Of Nitrogen Compound Metabolic Process | 46 | 2 | 1.07E-04 | 0.020 | | PLK2, ARRB1, ATP2B4, PDE10A, PLCB4, TENM3, ROBO1, EFHC1, MCTP2, CNKSR2, CHRM3, CLU, PTPRS, RAB27B, SYTL3, DOCK10, RGS10, DMD, DPYSL2, SDC2, ADGRL3, FGF13, TIAM2, LPAR1, FLRT2, TGFB2, EPHA7, EPHA3, MPP4, GPR1, TNFRSF21, CXCR4, NRP2, CPEB4, SORBS2, CASK, MMP3, KCNQ3, KISS1, NOG, KCNIP3, LTBP1, ASAP1, BCL11A, OPTN, NEO1 | |
| Regulation Of Leukocyte Migration | 126 | 1 | 1.08E-04 | 0.017 | | ARHGDIB, ARRB1, BCL2A1, EFHC1, CDH11, FBXO32, CLU, CLIC2, BMP4, CCND2, DMD, DPYSL2, DSP, TRIM6, PIK3AP1, CYP2J2, FGF13, LPAR1, STOM, EPHA7, EPHA3, EYA4, F3, F13A1, EFEMP1, FBP1, GRB14, HAS2, NCKAP1L, GBP1, GBP2, GBP3, PRICKLE1, GJA1, TTLL11, IL1B, HMGCS1, HES1, MEF2C, MAP3K1, MMP1, NRK, SAMD9L, KISS1, LCP1, LTBP1, SERPINB2, FAM83B, PKIA, PGM5, PIK3CG, PLCB4, ROBO1, SCN5A, SCP2, SOX4, SNAI2, TUFT1, VEGFA, WT1, TGFB2, THBS1, TGFBR3, TGM2, TIMP3, TLR3, SLC7A5, EVI5, CXCR4, CLIC3, SORBS2, CDC42BPA, CST7, CASK, SERPINB7, CRADD, PTGES, CYTIP, FCGR2C, PDLIM1, OPTN, MBNL2, EML6, NUAK1, PLK2, PDE10A, CNKSR2, CARD8, PTGR1, IFI44L, IRAK3, TDRD7, ARL2BP, PLCB1, PTPN22, PITPNC1, TIAM2, PDCD4, TRIB2, STXBP6, HDDC2, MAFIP, SNX10, SPANXA1, KCNIP3, ASAP1, BCL11A, CLIC6, LMO3, BAIAP2L1, KLHL4, CPPED1, DOCK10, HRASLS, ZMIZ1, CEMIP, MTUS1, SAMSN1, HHIP, MPP4, CDK15, CPEB4, AKR1E2, APOL6, PARD6B, AFAP1L2 | |
| Positive Regulation Of Transmembrane Receptor Protein Serine/Threonine Kinase Signaling Pathway | 35 | 2 | 1.11E-04 | 0.020 | | PGM5, PLAU, ADGRE5, CDH11, SLITRK1, CHRM3, BAIAP2L1, SCN5A, LAMA1, PTPRS, DMD, HEG1, DSP, ADGRL3, FGF13, FLRT2, EFNB2, TGM2, MPP4, TNS3, GJA1, CXCR4, POF1B, ITGB3, CPEB4, STXBP6, SORBS2, CDC42BPA, CASK, CLMP, MAP3K1, PDLIM1, LAMA3, LCP1, PARD6B | |
| Response To Abiotic Stimulus | 49 | 2 | 1.21E-04 | 0.019 | | PLK2, ARRB1, ATP2B4, PLCB4, TENM3, ROBO1, EFHC1, CNKSR2, CHRM3, CLU, SCN9A, PTPRS, DOCK10, RGS10, DMD, ARL2BP, DPYSL2, ADGRL3, TBC1D30, FGF13, TIAM2, SAMSN1, LPAR1, FLRT2, TGFB2, EPHA7, EPHA3, F2RL1, MPP4, GPR1, TNFRSF21, TTLL11, NRP2, ITGB3, CPEB4, SORBS2, CDC42BPA, FOXA1, CASK, MAFIP, MMP3, KCNQ3, KISS1, NOG, LCP1, KCNIP3, LTBP1, ASAP1, OPTN | |
| Regulation Of Peptide Transport | 198 | 1 | 1.53E-04 | 0.015 | | ARHGDIB, ARRB1, ATP2B4, AADAC, ABCA1, AK4, CD22, CD33, CD68, ADGRE5, CDH11, CLU, CLIC2, BMP4, BMP1, CCND2, CYP24A1, DMD, DPYSL2, DSP, CPT1A, CST4, CTSD, CYP2J2, FGF13, FLG, LPAR1, STOM, EPHA3, EYA4, F2RL1, ACSL1, EFEMP1, FBP1, GPR1, GRB14, NCKAP1L, HLA-A, HLA-B, GALC, GATA6, GBP1, GBP2, GBP3, GJA1, IL1B, ITGB3, CD82, HLA-DRA, FOXA1, HES1, MEF2C, MEIS3P1, MMP3, LAMA3, LAMA4, LCP1, LTBP1, NEO1, PON2, PKIA, PDE3B, PIK3CG, PLAU, PLCB4, PLXNA1, RRBP1, SCN5A, SCP2, PTPRS, RAB27B, RGS10, SOX4, SSX1, TBXAS1, TCF4, SNAI2, UGCG, VEGFA, WT1, TGFA, TGFB2, THBS1, TGFBR3, TGM2, TIMP3, TLR3, SLC7A5, HIST2H4B, SRPX, ZNF43, LAPTM5, EVI5, CXCR4, CLIC3, GPRC5A, SORBS2, CDC42BPA, LGR5, CASK, TNFSF10, CRADD, PTGES, CYTIP, B4GALT6, LIPG, RAD50, OPTN, MBNL2, CTDSPL, RAPGEF5, NUAK1, MGAT4A, CNKSR2, CARD8, PTGR1, IL24, PRSS23, IRAK3, SP140, ARL2BP, SEL1L3, PLCB1, PTPN22, TIAM2, FLRT2, PDCD4, TRIB2, HDDC2, SNX10, SPANXA1, KCNIP3, G0S2, BCL11A, CLIC6, GDAP1, CHST15, SNX7, LPCAT2, RBM47, BAIAP2L1, ACOXL, DOCK10, HRASLS, SLC44A2, ZMIZ1, ATP8B2, CEMIP, MTUS1, MARCH4, PMEPA1, PRDM9, PRDM8, FKBP10, SAMSN1, NXN, HHIP, SLAMF7, GPR135, PORCN, CDK15, ELOVL7, MYCT1, CPEB4, FAT4, MORC4, COLGALT1, AKR1E2, CRISPLD1, CRISPLD2, PARD6B, ANKRD30A, ARRDC4, TSPYL5, NAV3, FBXO32, MARCH3, SYTL3, TRIM6, PIK3AP1, TC2N, FAM172BP, PRICKLE1, ZDHHC15, TOR1AIP2, SHISA3, SAMD9L, SFTA3, SLC37A2, ZNF438, MAMDC2, SSX8, ZNF391, SERINC2, ILDR2, SHISA2, AGMO, ZNF716 | |
| Membranous Septum Morphogenesis | 48 | 2 | 2.18E-04 | 0.019 | | MIR181B1, PLAU, PLTP, CLU, SCP2, CCL2, LAMA1, BMP4, BMP1, DMKN, IL24, C4BPB, PRSS23, MTUS1, CSF1, CST4, CTSD, VEGFA, FLRT2, TGFA, TGFB2, THBS1, TGFBR3, TIMP3, STOM, ABI3BP, F3, EFEMP1, HLA-A, IGFBP4, IL1B, IL13RA2, INHBB, SERPINB7, TNFSF10, CRISPLD1, CRISPLD2, MMP3, MMP1, CPA4, IL1RL1, KISS1, NOG, LAMA4, LCP1, LIPG, LTBP1, SERPINB2 | |
| Response To Growth Factor | 3 | 30 | 2.38E-04 | 0.008 | | GBP1, GBP2, GBP3 | |
| Negative Regulation Of Peptide Secretion | 3 | 27 | 3.24E-04 | 0.008 | | NRP2, PLXNA2, PLXNA1 | |
| Bounding Membrane Of Organelle | 15 | 3 | 4.40E-04 | 0.019 | | DMD, PDLIM1, TNS3, PGM5, LAMA3, GJA1, LCP1, PLAU, ADGRE5, ITGB3, SORBS2, FLRT2, EFNB2, TGM2, CASK | |
| Positive Regulation Of Metabolic Process | 45 | 2 | 4.89E-04 | 0.019 | | LPCAT2, ARRB1, BCL2A1, NAV3, PLAU, CD33, CD68, MGAT4A, ADGRE5, MCTP2, MARCH3, RAB27B, CYP24A1, SLC44A2, CEMIP, DSP, MARCH4, CPT1A, PMEPA1, UGCG, HHIP, TGFA, STOM, TLR3, MPP4, ACSL1, GRB14, NCKAP1L, HLA-A, HLA-B, GPR135, GBP1, GBP2, GBP3, LAPTM5, GJA1, ZDHHC15, ITGB3, HLA-DRA, LGR5, CASK, B4GALT6, OPTN, GDAP1, CHST15 | |
| Positive Regulation Of Muscle Tissue Development | 2 | 66 | 4.92E-04 | 0.005 | | LAMA1, LAMA3 | |
| Cellular Response To Platelet-Derived Growth Factor Stimulus | 21 | 2 | 8.61E-04 | 0.019 | | LPCAT2, ARRB1, HLA-A, HLA-B, GBP1, GBP2, GBP3, GJA1, MGAT4A, ZDHHC15, HLA-DRA, LGR5, MARCH4, B4GALT6, PMEPA1, OPTN, UGCG, CHST15, TGFA, TLR3, MPP4 | |
| Positive Regulation Of Ovulation | 296 | 1 | 9.00E-04 | 0.014 | | ARHGDIB, ARRB1, ATP2B4, BCL2A1, AADAC, ABCA1, AK4, CD22, CD33, ENTPD3, CD68, ADGRE5, CDH11, CHRM3, CLU, CLIC2, BMP4, BMP1, C4BPB, CCND2, CYP24A1, DMD, DPYSL2, DSP, CPT1A, CSF1, CTSD, CYP2J2, FGF13, FLG, LPAR1, S1PR3, EFNB2, STOM, EPHA7, EPHA3, EYA4, F2RL1, F3, F2RL2, F13A1, ACSL1, EFEMP1, FBP1, GPR1, GRB14, HAS2, NCKAP1L, HLA-A, HLA-B, GALC, GATA6, GBP1, GBP2, GBP3, GJA1, GNGT2, IGFBP4, IL1B, IL13RA2, INHBB, ITGB3, CD82, HLA-DRA, HMGCS1, FOXA1, HES1, MEF2C, MEIS3P1, MAP3K1, MMP3, MMP1, KCNQ3, KISS1, LAMA3, LCP1, LTBP1, SERPINB2, NEO1, PON2, PKIA, TMPRSS15, PDE3B, PGM5, PIK3CG, PLAU, PLCB4, PLXNA2, PLXNA1, ROBO1, RRBP1, SCN5A, SCP2, SCN9A, CCL2, PTPRS, RAB27B, RGS10, SOX4, SSX1, TBXAS1, TCF4, SDC2, SLC22A3, SNAI2, TUFT1, UGCG, VEGFA, WT1, TGFA, TGFB2, THBS1, TGFBR3, TGM2, TIMP3, TLR3, SLC7A5, HIST2H4B, SRPX, ZNF43, LAPTM5, EVI5, CXCR4, NRP2, CLIC3, GPRC5A, SORBS2, GPR65, CDC42BPA, CST7, LGR5, CASK, SERPINB7, TNFSF10, CRADD, GMFG, PTGES, CYTIP, FCGR2C, PDLIM1, IL1RL1, NOG, B4GALT6, LIPG, TSPAN2, RAD50, OPTN, MBNL2, CTDSPL, RAPGEF5, NUAK1, CLEC2B, PLK2, PDE10A, MYL9, MGAT4A, CNKSR2, CARD8, PTGR1, IFI44L, IL24, PRSS23, IRAK3, SP140, TDRD7, ARL2BP, SEL1L3, PLCB1, ADGRL3, TBC1D30, PCDHB5, PTPN22, PITPNC1, TIAM2, OR2M4, FLRT2, TNFRSF21, PDCD4, PCDH17, PCDH11X, OR2H1, TRIB2, STXBP6, CDON, HDDC2, SNX10, SPANXA1, KCNIP3, G0S2, ASAP1, BCL11A, SHC3, CNTN5, CLIC6, GDAP1, CHST15, SNX7, LPCAT2, RBM47, TENM3, MCTP2, LMO3, BAIAP2L1, KLHL4, PCDHB6, ACOXL, CPPED1, DOCK10, HRASLS, SLC44A2, ZMIZ1, ATP8B2, CEMIP, HEG1, MTUS1, MARCH4, PCDHB2, PMEPA1, PRDM9, PRDM8, FKBP10, SAMSN1, NXN, HHIP, PCDHB16, SLAMF7, MPP4, GPR135, TNS3, PORCN, CDK15, POF1B, ELOVL7, MYCT1, CPEB4, FAT4, VEPH1, MORC4, COLGALT1, CLMP, PCDH11Y, AKR1E2, CRISPLD2, APOL6, PARD6B, AFAP1L2, ANKRD30A, ARRDC4, TSPYL5, NAV3, EFHC1, SLITRK1, FBXO32, MARCH3, TLCD1, SYTL3, OR2M3, TRIM6, PIK3AP1, OR5P2, TC2N, FOXR2, LYPD6B, FAM172BP, PRICKLE1, LRFN5, TTLL11, ZDHHC15, TOR1AIP2, SHISA3, SH3RF2, NRK, SAMD9L, COX7B2, PLPP4, METTL7B, SFTA3, SLC37A2, OR5B21, ZNF438, FAM83B, CD163L1, MAMDC2, ADGRF1, SSX8, ZNF391, SERINC2, ILDR2, SHISA2, AGMO, EML6, ZNF716, MAFIP, KRTAP4-8, SNORD123, KRTAP20-4 | |
| Regulation Of Immune System Process | 8 | 5 | 9.33E-04 | 0.016 | | DMD, ATP2B4, PDLIM1, MYL9, PGM5, FBXO32, SCN5A, SORBS2 | |
| Lipid Metabolic Process | 40 | 2 | 1.11E-03 | 0.018 | | ARHGDIB, ARRB1, ARRDC4, ABCA1, CD68, MARCH3, CLU, PTPRS, RAB27B, SYTL3, CEMIP, PMEPA1, CTSD, VEGFA, LPAR1, TGFA, TGFB2, THBS1, STOM, EPHA3, TLR3, F2RL1, GRB14, GPR135, GBP1, GBP2, GBP3, GJA1, CXCR4, IL1B, ITGB3, CYTIP, CRISPLD2, SAMD9L, SNX10, LIPG, OPTN, SFTA3, SNX7, NEO1 | |
| Negative Regulation Of Catalytic Activity | 213 | 1 | 1.19E-03 | 0.015 | | ARHGDIB, ARRB1, ATP2B4, AADAC, ABCA1, AK4, CD22, CD33, CD68, ADGRE5, CDH11, CLU, CLIC2, BMP4, BMP1, CCND2, CYP24A1, DMD, DPYSL2, DSP, CPT1A, CST4, CTSD, CYP2J2, FGF13, FLG, LPAR1, STOM, EPHA3, EYA4, F2RL1, ACSL1, EFEMP1, FBP1, GPR1, GRB14, NCKAP1L, HLA-A, HLA-B, GALC, GATA6, GBP1, GBP2, GBP3, GJA1, IL1B, ITGB3, CD82, HLA-DRA, FOXA1, HES1, MEF2C, MEIS3P1, MAP3K1, MMP3, LAMA3, LAMA4, LCP1, LTBP1, NEO1, PON2, PKIA, PDE3B, PGM5, PIK3CG, PLAU, PLCB4, PLXNA1, RRBP1, SCN5A, SCP2, PTPRS, RAB27B, RGS10, SOX4, SSX1, TBXAS1, TCF4, SNAI2, UGCG, VEGFA, WT1, TGFA, TGFB2, THBS1, TGFBR3, TGM2, TIMP3, TLR3, SLC7A5, HIST2H4B, SRPX, ZNF43, LAPTM5, EVI5, CXCR4, CLIC3, GPRC5A, SORBS2, CDC42BPA, LGR5, CASK, TNFSF10, CRADD, PTGES, CYTIP, PDLIM1, B4GALT6, LIPG, RAD50, OPTN, MBNL2, CTDSPL, RAPGEF5, NUAK1, PLK2, MGAT4A, CNKSR2, CARD8, PTGR1, IL24, PRSS23, IRAK3, SP140, TDRD7, ARL2BP, SEL1L3, PLCB1, TBC1D30, PTPN22, TIAM2, FLRT2, PDCD4, TRIB2, HDDC2, SNX10, SPANXA1, KCNIP3, G0S2, ASAP1, BCL11A, CLIC6, GDAP1, CHST15, SNX7, LPCAT2, RBM47, BAIAP2L1, KLHL4, ACOXL, DOCK10, HRASLS, SLC44A2, ZMIZ1, ATP8B2, CEMIP, MTUS1, MARCH4, PMEPA1, PRDM9, PRDM8, FKBP10, SAMSN1, NXN, HHIP, SLAMF7, MPP4, GPR135, PORCN, CDK15, ELOVL7, MYCT1, CPEB4, FAT4, MORC4, COLGALT1, AKR1E2, CRISPLD1, CRISPLD2, PARD6B, ANKRD30A, ARRDC4, TSPYL5, NAV3, EFHC1, FBXO32, MARCH3, SYTL3, TRIM6, PIK3AP1, TC2N, FAM172BP, PRICKLE1, TTLL11, ZDHHC15, TOR1AIP2, SHISA3, SAMD9L, METTL7B, SFTA3, SLC37A2, ZNF438, MAMDC2, SSX8, ZNF391, SERINC2, ILDR2, SHISA2, AGMO, EML6, ZNF716, MAFIP, SNORD123 | |
| Regulation Of Multi-Organism Process | 21 | 2 | 1.21E-03 | 0.019 | | ARRB1, NCKAP1L, HLA-A, HLA-B, GBP1, PLAU, CD33, CD68, ADGRE5, MCTP2, MARCH3, ITGB3, GPRC5A, HLA-DRA, RAB27B, SLC44A2, CEMIP, DSP, TGFA, SNX7, STOM | |
| Atrial Septum Primum Morphogenesis | 5 | 7 | 1.51E-03 | 0.012 | | FGF13, DSP, SCN5A, PGM5, GJA1 | |
| Negative Regulation Of Response To Wounding | 5 | 7 | 1.62E-03 | 0.012 | | FGF13, DMD, SCN5A, GJA1, MPP4 | |
| Positive Regulation Of Peptidyl-Lysine Acetylation | 5 | 7 | 1.73E-03 | 0.012 | | VEGFA, CLU, TGFB2, THBS1, F13A1 | |
| Cellular Component Morphogenesis | 20 | 2 | 1.90E-03 | 0.018 | | ARRB1, NCKAP1L, HLA-A, HLA-B, PLAU, CD33, CD68, ADGRE5, MCTP2, MARCH3, ITGB3, GPRC5A, HLA-DRA, RAB27B, SLC44A2, CEMIP, DSP, TGFA, SNX7, STOM | |
| Epithelial Cell Differentiation Involved In Kidney Development | 39 | 2 | 1.93E-03 | 0.018 | | ARHGDIB, ARRB1, ARRDC4, ABCA1, CD68, MARCH3, CLU, PTPRS, RAB27B, SYTL3, CEMIP, PMEPA1, CTSD, VEGFA, LPAR1, TGFA, TGFB2, THBS1, STOM, EPHA3, TLR3, F2RL1, GRB14, GPR135, GBP1, GBP2, GBP3, GJA1, CXCR4, IL1B, ITGB3, CYTIP, CRISPLD2, SAMD9L, SNX10, LIPG, OPTN, SFTA3, SNX7 | |
| Bmp Signaling Pathway Involved In Heart Development | 3 | 14 | 2.37E-03 | 0.008 | | GBP1, GBP2, GBP3 | |
| Peptide Cross-Linking | 247 | 1 | 2.47E-03 | 0.014 | | ARHGDIB, ARRB1, ATP2B4, BCL2A1, AADAC, ABCA1, AK4, CD33, CD68, ADGRE5, CDH11, CLU, CLIC2, BMP4, BMP1, CCND2, CYP24A1, DMD, DPYSL2, DSP, CPT1A, CSF1, CTSD, CYP2J2, FGF13, FLG, LPAR1, STOM, EPHA7, EPHA3, EYA4, F2RL1, F3, F13A1, ACSL1, EFEMP1, FBP1, GPR1, GRB14, HAS2, NCKAP1L, HLA-A, HLA-B, GALC, GATA6, GBP1, GBP2, GBP3, GJA1, GNGT2, IGFBP4, IL1B, INHBB, ITGB3, HLA-DRA, HMGCS1, FOXA1, HES1, MEF2C, MEIS3P1, MAP3K1, MMP3, MMP1, KISS1, LAMA3, LCP1, LTBP1, SERPINB2, NEO1, PON2, PKIA, PDE3B, PGM5, PIK3CG, PLAU, PLCB4, ROBO1, RRBP1, SCN5A, SCP2, PTPRS, RAB27B, RGS10, SOX4, SSX1, TBXAS1, TCF4, SDC2, SNAI2, TUFT1, UGCG, VEGFA, WT1, TGFA, TGFB2, THBS1, TGFBR3, TGM2, TIMP3, TLR3, SLC7A5, HIST2H4B, SRPX, ZNF43, LAPTM5, EVI5, CXCR4, CLIC3, GPRC5A, SORBS2, CDC42BPA, CST7, LGR5, CASK, SERPINB7, CRADD, GMFG, PTGES, CYTIP, FCGR2C, PDLIM1, B4GALT6, LIPG, RAD50, OPTN, MBNL2, CTDSPL, RAPGEF5, NUAK1, PLK2, PDE10A, MYL9, MGAT4A, CNKSR2, CARD8, PTGR1, IFI44L, IL24, PRSS23, IRAK3, SP140, TDRD7, ARL2BP, SEL1L3, PLCB1, TBC1D30, PTPN22, PITPNC1, TIAM2, FLRT2, PDCD4, TRIB2, STXBP6, HDDC2, SNX10, SPANXA1, KCNIP3, G0S2, ASAP1, BCL11A, SHC3, CNTN5, CLIC6, GDAP1, CHST15, SNX7, LPCAT2, RBM47, MCTP2, LMO3, BAIAP2L1, KLHL4, ACOXL, CPPED1, DOCK10, HRASLS, SLC44A2, ZMIZ1, ATP8B2, CEMIP, MTUS1, MARCH4, PMEPA1, PRDM9, PRDM8, FKBP10, SAMSN1, NXN, HHIP, SLAMF7, MPP4, GPR135, TNS3, PORCN, CDK15, POF1B, ELOVL7, MYCT1, CPEB4, MORC4, COLGALT1, CLMP, AKR1E2, CRISPLD2, APOL6, PARD6B, AFAP1L2, ANKRD30A, ARRDC4, TSPYL5, NAV3, EFHC1, FBXO32, MARCH3, SYTL3, TRIM6, PIK3AP1, TC2N, FOXR2, FAM172BP, PRICKLE1, TTLL11, ZDHHC15, TOR1AIP2, SHISA3, SH3RF2, NRK, SAMD9L, COX7B2, METTL7B, SFTA3, SLC37A2, ZNF438, FAM83B, MAMDC2, SSX8, ZNF391, SERINC2, ILDR2, SHISA2, AGMO, EML6, ZNF716, MAFIP, KRTAP4-8, SNORD123, KRTAP20-4 | |
| Positive Regulation Of Cardiac Muscle Tissue Development | 4 | 8 | 2.66E-03 | 0.010 | | GBP1, C4BPB, GBP2, GBP3 | |
| Aminoglycan Metabolic Process | 13 | 3 | 2.85E-03 | 0.017 | | PDLIM1, TNS3, PGM5, GJA1, LCP1, PLAU, ADGRE5, ITGB3, SORBS2, FLRT2, EFNB2, TGM2, CASK | |
| Behavioral Response To Pain | 31 | 2 | 2.94E-03 | 0.018 | | ARRB1, BCL2A1, NAV3, PLAU, CD33, CD68, ADGRE5, MCTP2, MARCH3, RAB27B, CYP24A1, SLC44A2, CEMIP, DSP, CPT1A, PMEPA1, TGFA, STOM, TLR3, ACSL1, GRB14, NCKAP1L, HLA-A, HLA-B, GPR135, LAPTM5, GJA1, ITGB3, HLA-DRA, OPTN, GDAP1 | |
| Cell-Substrate Adherens Junction | 17 | 2 | 2.98E-03 | 0.018 | | PDE10A, ROBO1, EFHC1, CNKSR2, SORBS2, CASK, RGS10, DPYSL2, SDC2, KCNQ3, KISS1, LTBP1, OPTN, LPAR1, TGFB2, EPHA7, NEO1 | |
| Comma-Shaped Body Morphogenesis | 13 | 3 | 3.16E-03 | 0.017 | | PDLIM1, TNS3, PGM5, GJA1, LCP1, PLAU, ADGRE5, ITGB3, SORBS2, FLRT2, EFNB2, TGM2, CASK | |
| Cellular Response To Low-Density Lipoprotein Particle Stimulus | 30 | 2 | 3.38E-03 | 0.018 | | LPCAT2, PDE3B, ABCA1, MGAT4A, CLU, BMP1, RAB27B, ATP8B2, MTUS1, MARCH4, PMEPA1, UGCG, F2RL1, GRB14, HLA-A, HLA-B, GBP1, GBP2, GBP3, EVI5, GJA1, ZDHHC15, LGR5, AKR1E2, KCNIP3, B4GALT6, LIPG, OPTN, CHST15, NEO1 | |
| Cell-Cell Contact Zone | 56 | 1 | 4.03E-03 | 0.017 | | ARRB1, BCL2A1, AADAC, NAV3, CD33, CD68, MGAT4A, ADGRE5, MARCH3, CLU, CCND2, CYP24A1, DSP, CPT1A, PLCB1, CYP2J2, FLRT2, STOM, ACSL1, GRB14, NCKAP1L, HLA-A, HLA-B, GATA6, GBP1, GBP2, GBP3, PRICKLE1, GJA1, ZDHHC15, ITGB3, TOR1AIP2, HLA-DRA, GDAP1, CHST15, SNX7, LPCAT2, PLAU, MCTP2, RAB27B, SLC44A2, CEMIP, MARCH4, PMEPA1, UGCG, HHIP, TGFA, TLR3, MPP4, GPR135, LAPTM5, GPRC5A, LGR5, CASK, B4GALT6, OPTN | |
| Cell Part Morphogenesis | 17 | 2 | 4.10E-03 | 0.017 | | ARRB1, HLA-A, HLA-B, PORCN, PGM5, GJA1, GNGT2, CDH11, ITGB3, SCN5A, SCN9A, HLA-DRA, DMD, KCNQ3, KCNIP3, TGFBR3, NEO1 | |
| Regulation Of Epithelial To Mesenchymal Transition Involved In Endocardial Cushion Formation | 2 | 25 | 4.40E-03 | 0.005 | | LAMA1, LAMA3 | |
| Negative Regulation Of Apoptotic Process Involved In Development | 5 | 6 | 4.41E-03 | 0.011 | | DSP, PGM5, GJA1, FGF13, SCN5A | |
| Positive Regulation Of Cysteine-Type Endopeptidase Activity Involved In Apoptotic Process | 24 | 2 | 4.42E-03 | 0.017 | | LPCAT2, ARRB1, HLA-A, HLA-B, GBP1, GBP2, GBP3, GJA1, MGAT4A, ZDHHC15, HLA-DRA, LGR5, RAB27B, MARCH4, SDC2, B4GALT6, PMEPA1, OPTN, UGCG, CHST15, TGFA, TGFB2, TLR3, MPP4 | |
| Regulation Of Nitrogen Compound Metabolic Process | 24 | 2 | 5.20E-03 | 0.017 | | LPCAT2, ILDR2, SHISA2, AADAC, ABCA1, PORCN, GJA1, ELOVL7, TOR1AIP2, SHISA3, AGMO, PTGES, ATP8B2, TBXAS1, CPT1A, METTL7B, CYP2J2, SFTA3, FLRT2, SLC37A2, TGFA, TLR3, ACSL1, SERINC2 | |
| Forebrain Cell Migration | 5 | 5 | 5.38E-03 | 0.011 | | HLA-A, HLA-B, GJA1, HLA-DRA, TGFA | |
| Regulation Of Ventricular Cardiac Muscle Cell Membrane Depolarization | 22 | 2 | 5.50E-03 | 0.017 | | ARRB1, PLCB4, MCTP2, CNKSR2, CHRM3, CPEB4, SORBS2, PTPRS, CASK, RAB27B, SYTL3, DOCK10, RGS10, MEF2C, DMD, DPYSL2, KCNIP3, ASAP1, BCL11A, LPAR1, EPHA7, MPP4 | |
| Regulation Of Collagen Biosynthetic Process | 18 | 2 | 5.68E-03 | 0.017 | | PDE10A, ROBO1, EFHC1, CNKSR2, SORBS2, CASK, RGS10, MMP3, DPYSL2, SDC2, KCNQ3, KISS1, LTBP1, OPTN, LPAR1, TGFB2, EPHA7, NEO1 | |
| Contractile Fiber Part | 3 | 10 | 6.05E-03 | 0.008 | | HLA-A, HLA-B, HLA-DRA | |
| Signal Transduction Involved In Cellular Response To Ammonium Ion | 9 | 3 | 6.22E-03 | 0.015 | | MEF2C, DMD, ATP2B4, PDLIM1, MYL9, PGM5, FBXO32, SCN5A, SORBS2 | |
| Pdz Domain Binding | 4 | 6 | 7.06E-03 | 0.010 | | HLA-A, HLA-B, HLA-DRA, TGFA | |
| Receptor Ligand Activity | 10 | 3 | 7.41E-03 | 0.015 | | SDC2, CSF1, LTBP1, MGAT4A, IGFBP4, FKBP10, BMP4, THBS1, COLGALT1, PRSS23 | |
| Motor Neuron Axon Guidance | 27 | 2 | 7.79E-03 | 0.017 | | AK4, MGAT4A, CLU, SCP2, BMP4, ACOXL, CPPED1, PRSS23, HRASLS, TDRD7, ARL2BP, SDC2, CSF1, CTSD, FKBP10, VEGFA, TGFB2, THBS1, TIMP3, F13A1, GALC, IGFBP4, COLGALT1, GMFG, PTGES, CRISPLD2, LTBP1 | |
| Positive Regulation Of Positive Chemotaxis | 10 | 3 | 7.90E-03 | 0.015 | | SLC44A2, DSP, NCKAP1L, PLAU, CD33, CD68, ADGRE5, ITGB3, RAB27B, STOM | |
| Positive Regulation Of Hormone Secretion | 27 | 2 | 8.10E-03 | 0.017 | | AK4, MGAT4A, CLU, SCP2, BMP4, ACOXL, CPPED1, PRSS23, HRASLS, TDRD7, ARL2BP, SDC2, CSF1, CTSD, FKBP10, VEGFA, TGFB2, THBS1, TIMP3, F13A1, GALC, IGFBP4, COLGALT1, GMFG, PTGES, CRISPLD2, LTBP1 | |
| Heat Generation | 27 | 2 | 8.10E-03 | 0.017 | | AK4, MGAT4A, CLU, SCP2, BMP4, ACOXL, CPPED1, PRSS23, HRASLS, TDRD7, ARL2BP, SDC2, CSF1, CTSD, FKBP10, VEGFA, TGFB2, THBS1, TIMP3, F13A1, GALC, IGFBP4, COLGALT1, GMFG, PTGES, CRISPLD2, LTBP1 | |
| Cellular Response To Environmental Stimulus | 3 | 9 | 8.69E-03 | 0.008 | | CLU, ABCA1, PLTP | |
| Lymphoid Progenitor Cell Differentiation | 10 | 3 | 9.72E-03 | 0.015 | | GMFG, CRISPLD2, CTSD, VEGFA, CLU, CPPED1, TGFB2, THBS1, TIMP3, F13A1 | |
| **Enrichment for Molecular Function** | | | | | | |  |
| Mesenchymal Cell Differentiation | 28 | 3 | 3.41E-06 | 0.025 | | LPCAT2, PCDH17, PCDH11X, MYL9, PLCB4, EFHC1, ADGRE5, CDH11, MCTP2, PCDHB6, FAT4, BMP1, PCDH11Y, HEG1, PCDHB2, LCP1, PLCB1, KCNIP3, ADGRL3, LTBP1, FKBP10, PCDHB5, FLG, THBS1, TGM2, PCDHB16, TLL1, EFEMP1 |  |
| Regulation Of I-Kappab Kinase/Nf-Kappab Signaling | 142 | 1 | 8.45E-06 | 0.018 | | ARHGDIB, ARRB1, ATP2B4, ARRDC4, BCL2A1, ABCA1, NAV3, EFHC1, ADGRE5, CDH11, CLU, BMP4, BMP1, SYTL3, CCND2, DMD, DPYSL2, DSP, TRIM6, PIK3AP1, CSF1, FGF13, LPAR1, S1PR3, EFNB2, STOM, EPHA7, EPHA3, EYA4, F2RL1, F3, EFEMP1, GRB14, HLA-A, HLA-B, GATA6, GBP1, GBP2, GBP3, PRICKLE1, GJA1, IGFBP4, IL1B, IL13RA2, INHBB, ITGB3, TOR1AIP2, HMGCS1, FOXA1, HES1, SH3RF2, MEF2C, MAP3K1, KCNQ3, KISS1, LAMA3, LAMA4, LCP1, LTBP1, NEO1, FAM83B, PKIA, PDE3B, PIK3CG, PLCB4, ROBO1, SCN5A, SCP2, CCL2, LAMA1, PTPRS, RAB27B, RGS10, SOX4, TCF4, SDC2, VEGFA, WT1, TGFA, TGFB2, THBS1, TGFBR3, TGM2, TIMP3, SERINC2, HIST2H4B, EVI5, CXCR4, NRP2, GPRC5A, SORBS2, CASK, SAMD5, TNFSF10, CRADD, GMFG, PDLIM1, IL1RL1, NOG, RAD50, OPTN, EML6, RAPGEF5, NUAK1, PLK2, MYL9, CNKSR2, CARD8, IL24, ESM1, IRAK3, TDRD7, PLCB1, ADGRL3, TBC1D30, PTPN22, TIAM2, FLRT2, TRIB2, STXBP6, SNX10, KCNIP3, ASAP1, BCL11A, SHC3, CLIC6, TENM3, BAIAP2L1, KLHL4, DOCK10, CEMIP, MTUS1, PMEPA1, SAMSN1, HHIP, MPP4, GPR135, PORCN, CDK15, POF1B, PARD6B, AFAP1L2 |  |
| Negative Regulation Of Biological Process | 128 | 1 | 1.61E-05 | 0.018 | | ATP2B4, ABCA1, AK4, NAV3, ENTPD3, EFHC1, ADGRE5, CDH11, MARCH3, CHRM3, BMP4, BMP1, SYTL3, CYP24A1, DMD, TRIM6, CYP2J2, FLG, LPAR1, EPHA7, EPHA3, EYA4, F3, F13A1, ACSL1, EFEMP1, FBP1, GATA6, GBP1, GBP2, GBP3, PRICKLE1, TTLL11, ZDHHC15, SH3RF2, MAP3K1, MMP3, MMP1, NRK, LCP1, LTBP1, ZNF438, PON2, PDE3B, PGM5, PIK3CG, PLCB4, PLTP, SCP2, SCN9A, PTPRS, RAB27B, TBXAS1, SNAI2, VEGFA, WT1, RFPL4A, THBS1, TGFBR3, TGM2, TIMP3, TLL1, ZNF391, ZNF43, NRP2, CDC42BPA, CASK, TNFSF10, AGMO, PTGES, PDLIM1, B4GALT6, LIPG, RAD50, OPTN, MBNL2, CTDSPL, NUAK1, PLK2, PDE10A, ZNF716, MYL9, MGAT4A, IFI44L, IRAK3, SP140, PLCB1, ADGRL3, PCDHB5, PITPNC1, ABI3BP, PCDH17, PCDH11X, TRIB2, STXBP6, CPA4, SNX10, KCNIP3, ASAP1, BCL11A, CHST15, LPCAT2, RFPL4AL1, MCTP2, LMO3, PCDHB6, ACOXL, CPPED1, ZMIZ1, ATP8B2, CEMIP, HEG1, ZNF608, MARCH4, PCDHB2, PRDM9, PRDM8, FKBP10, HHIP, PCDHB16, TNS3, CDK15, CPEB4, FAT4, MORC4, ADAMTS12, PCDH11Y, CRISPLD2 |  |
| Positive Regulation Of Cell Motility | 27 | 3 | 2.01E-05 | 0.023 | | GRB14, ARRB1, PKIA, ATP2B4, PDE3B, GATA6, GJA1, PLCB4, CNKSR2, TRIB2, ITGB3, SCN5A, SAMD5, HES1, CCND2, MAP3K1, DPYSL2, DSP, KCNQ3, TRIM6, PARD6B, BCL11A, SHC3, PTPN22, EPHA7, EPHA3, FAM83B |  |
| Cation Binding | 10 | 7 | 2.10E-05 | 0.020 | | IL1RL1, GBP1, NOG, CXCR4, LTBP1, NRP2, IL13RA2, ITGB3, THBS1, TGFBR3 |  |
| Phosphate-Containing Compound Metabolic Process | 4 | 30 | 2.11E-05 | 0.011 | | PITPNC1, SCP2, ABCA1, PLTP |  |
| Regulation Of Secretion By Cell | 94 | 1 | 2.87E-05 | 0.019 | | ATP2B4, PDE10A, ZNF716, MYL9, MGAT4A, EFHC1, ADGRE5, CDH11, MARCH3, CHRM3, BMP1, SYTL3, IRAK3, SP140, CYP24A1, DMD, TRIM6, PLCB1, ADGRL3, CYP2J2, PCDHB5, FLG, PITPNC1, EYA4, F13A1, EFEMP1, FBP1, PCDH17, PCDH11X, GATA6, PRICKLE1, ZDHHC15, SH3RF2, MAP3K1, MMP3, MMP1, CPA4, LCP1, KCNIP3, LTBP1, ASAP1, BCL11A, ZNF438, PON2, LPCAT2, PDE3B, RFPL4AL1, PGM5, PLCB4, PLTP, MCTP2, LMO3, SCN9A, PCDHB6, CPPED1, ZMIZ1, ATP8B2, HEG1, ZNF608, TBXAS1, MARCH4, PCDHB2, PRDM9, PRDM8, SNAI2, FKBP10, WT1, HHIP, RFPL4A, THBS1, TGM2, PCDHB16, TIMP3, TLL1, ZNF391, ZNF43, TNS3, CDK15, NRP2, CPEB4, FAT4, CDC42BPA, MORC4, TNFSF10, ADAMTS12, AGMO, PCDH11Y, PDLIM1, B4GALT6, RAD50, OPTN, MBNL2, CTDSPL, NUAK1 |  |
| Positive Regulation Of Locomotion | 236 | 1 | 5.49E-05 | 0.015 | | ARHGDIB, ARRB1, ATP2B4, BCL2A1, ABCA1, AK4, CD22, CD33, ENTPD3, ADGRE5, CDH11, CHRM3, CLU, BMP4, BMP1, CCND2, CYP24A1, DMD, DPYSL2, DSP, CSF1, CTSD, CYP2J2, FGF13, FLG, LPAR1, S1PR3, EFNB2, STOM, EPHA7, EPHA3, EYA4, F2RL1, F3, F13A1, ACSL1, EFEMP1, FBP1, GPR1, GRB14, NCKAP1L, HLA-A, HLA-B, GATA6, GBP1, GBP2, GBP3, GJA1, IGFBP4, IL1B, IL13RA2, INHBB, ITGB3, HLA-DRA, HMGCS1, FOXA1, HES1, MEF2C, MEIS3P1, MAP3K1, MMP3, MMP1, KCNQ3, KISS1, LAMA3, LAMA4, LCP1, LTBP1, NEO1, PON2, PKIA, PDE3B, PGM5, PIK3CG, PLCB4, PLTP, ROBO1, RRBP1, SCN5A, SCP2, SCN9A, CCL2, PTPRS, RAB27B, RGS10, SOX4, SSX1, TBXAS1, TCF4, SDC2, SNAI2, VEGFA, WT1, TGFA, TGFB2, THBS1, TGFBR3, TGM2, TIMP3, TLL1, TLR3, SLC7A5, HIST2H4B, ZNF43, EVI5, CXCR4, NRP2, GPRC5A, SORBS2, CDC42BPA, CASK, TNFSF10, CRADD, GMFG, PTGES, FCGR2C, PDLIM1, IL1RL1, NOG, B4GALT6, LIPG, RAD50, OPTN, MBNL2, CTDSPL, RAPGEF5, NUAK1, CLEC2B, PLK2, PDE10A, MYL9, MGAT4A, CNKSR2, CARD8, IFI44L, IL24, ESM1, IRAK3, SP140, TDRD7, PLCB1, ADGRL3, TBC1D30, PCDHB5, PTPN22, PITPNC1, TIAM2, FLRT2, ABI3BP, PDCD4, PCDH17, PCDH11X, TRIB2, STXBP6, CPA4, SNX10, KCNIP3, ASAP1, BCL11A, SHC3, CLIC6, CHST15, SNX7, LPCAT2, RBM47, TENM3, MCTP2, LMO3, BAIAP2L1, KLHL4, PCDHB6, ACOXL, CPPED1, DOCK10, ZMIZ1, ATP8B2, CEMIP, HEG1, MTUS1, ZNF608, MARCH4, PCDHB2, PMEPA1, PRDM9, PRDM8, FKBP10, SAMSN1, HHIP, PCDHB16, MPP4, GPR135, TNS3, PORCN, CDK15, POF1B, CPEB4, FAT4, MORC4, ADAMTS12, PCDH11Y, CRISPLD2, APOL6, PARD6B, AFAP1L2, ANKRD30A, ARRDC4, NAV3, EFHC1, MARCH3, SYTL3, TRIM6, PIK3AP1, OR5P2, FOXR2, FAM172BP, PRICKLE1, TTLL11, ZDHHC15, TOR1AIP2, SH3RF2, NRK, OR5B21, ZNF438, FAM83B, MAMDC2, SSX8, LAMA1, RFPL4A, ZNF391, SERINC2, SAMD5, AGMO, EML6, ZNF716, MIR181B1, RFPL4AL1 |  |
| Cellular Response To Growth Factor Stimulus | 91 | 1 | 5.60E-05 | 0.019 | | ATP2B4, PDE10A, ZNF716, MYL9, MGAT4A, EFHC1, ADGRE5, CDH11, MARCH3, BMP1, SYTL3, IRAK3, SP140, CYP24A1, DMD, TRIM6, PLCB1, ADGRL3, CYP2J2, PCDHB5, FLG, EYA4, F13A1, EFEMP1, FBP1, PCDH17, PCDH11X, GATA6, PRICKLE1, ZDHHC15, SH3RF2, MAP3K1, MMP3, MMP1, CPA4, LCP1, KCNIP3, LTBP1, ASAP1, BCL11A, ZNF438, PON2, LPCAT2, PDE3B, RFPL4AL1, PGM5, PLCB4, MCTP2, LMO3, SCN9A, PCDHB6, CPPED1, ZMIZ1, ATP8B2, HEG1, ZNF608, TBXAS1, MARCH4, PCDHB2, PRDM9, PRDM8, SNAI2, FKBP10, WT1, HHIP, RFPL4A, THBS1, TGM2, PCDHB16, TIMP3, TLL1, ZNF391, ZNF43, TNS3, CDK15, NRP2, CPEB4, FAT4, CDC42BPA, MORC4, TNFSF10, ADAMTS12, AGMO, PCDH11Y, PDLIM1, B4GALT6, RAD50, OPTN, MBNL2, CTDSPL, NUAK1 |  |
| Cellular Response To Mechanical Stimulus | 27 | 2 | 1.15E-04 | 0.022 | | GRB14, ARRB1, PKIA, ATP2B4, PDE3B, GATA6, GJA1, PLCB4, CNKSR2, TRIB2, ITGB3, SCN5A, SAMD5, HES1, CCND2, MAP3K1, DPYSL2, DSP, KCNQ3, TRIM6, PARD6B, BCL11A, SHC3, PTPN22, EPHA7, EPHA3, FAM83B |  |
| Adhesion Of Symbiont To Host | 11 | 5 | 1.18E-04 | 0.020 | | GRB14, ARRB1, ARRDC4, DSP, TRIM6, RAD50, OPTN, SORBS2, FLRT2, SERINC2, CRADD |  |
| Cellular Developmental Process | 2 | 100 | 1.82E-04 | 0.005 | | PORCN, CPT1A |  |
| Positive Regulation Of Cytosolic Calcium Ion Concentration | 10 | 5 | 1.90E-04 | 0.019 | | GMFG, CSF1, FGF13, INHBB, VEGFA, BMP4, BMP1, TGFA, TGFB2, EFEMP1 |  |
| Glial Cell Development | 12 | 4 | 2.55E-04 | 0.019 | | GRB14, ARRB1, ARRDC4, DSP, TRIM6, RAD50, AFAP1L2, OPTN, SORBS2, FLRT2, SERINC2, CRADD |  |
| Regulation Of Toll-Like Receptor Signaling Pathway | 9 | 5 | 3.49E-04 | 0.017 | | LTBP1, IGFBP4, NRP2, VEGFA, ITGB3, SCN5A, THBS1, TGFBR3, ESM1 |  |
| Regulation Of Establishment Of Protein Localization | 3 | 27 | 3.70E-04 | 0.008 | | NRP2, PLXNA2, PLXNA1 |  |
| Cellular Response To Fluid Shear Stress | 4 | 15 | 3.84E-04 | 0.010 | | CHRM3, PLCB1, PLCB4, F2RL2 |  |
| Animal Organ Development | 4 | 15 | 3.84E-04 | 0.010 | | ITGB3, SCN5A, THBS1, TGFBR3 |  |
| Positive Regulation Of Muscle Organ Development | 7 | 6 | 4.69E-04 | 0.015 | | PDE10A, CHRM3, PDE3B, PLCB1, PLCB4, F2RL2, FAM83B |  |
| Cellular Response To Organic Cyclic Compound | 4 | 13 | 5.91E-04 | 0.010 | | CHRM3, PLCB1, PLCB4, F2RL2 |  |
| Ventricular Cardiac Muscle Tissue Morphogenesis | 15 | 3 | 6.27E-04 | 0.019 | | PDE10A, PDE3B, PLCB1, PLCB4, PLPP4, ENTPD3, PTPN22, CTDSPL, CHRM3, PTPRS, CPPED1, EYA4, F2RL2, FBP1, FAM83B |  |
| Regulation Of Interleukin-6 Production | 11 | 4 | 7.54E-04 | 0.018 | | CEMIP, CRISPLD2, MAMDC2, LIPG, NRP2, VEGFA, BMP4, PTPRS, THBS1, TGFBR3, ABI3BP |  |
| Epithelial Cell Proliferation Involved In Renal Tubule Morphogenesis | 7 | 6 | 9.11E-04 | 0.015 | | HRASLS, PLCB1, PLCB4, LIPG, CHRM3, F2RL2, FAM83B |  |
| Negative Regulation Of Branching Morphogenesis Of A Nerve | 12 | 3 | 9.24E-04 | 0.018 | | PTGES, CRISPLD2, LIPG, NRP2, VEGFA, SCP2, BMP4, PTPRS, CHST15, THBS1, TGFBR3, ABI3BP |  |
| Positive Regulation Of Nitrogen Compound Metabolic Process | 5 | 8 | 1.02E-03 | 0.012 | | ADGRE5, LPAR1, ADGRL3, F2RL1, RGS10 |  |
| Organelle | 2 | 50 | 1.07E-03 | 0.005 | | SCP2, PLTP |  |
| Vesicle Membrane | 2 | 50 | 1.07E-03 | 0.005 | | GBP1, GBP3 |  |
| Cell Proliferation | 4 | 11 | 1.09E-03 | 0.010 | | BAIAP2L1, DSP, STXBP6, PDLIM1 |  |
| Regulation Of Wound Healing | 9 | 4 | 1.11E-03 | 0.017 | | CRISPLD2, LIPG, NRP2, VEGFA, BMP4, PTPRS, THBS1, TGFBR3, ABI3BP |  |
| Negative Regulation Of Extrinsic Apoptotic Signaling Pathway | 8 | 5 | 1.14E-03 | 0.016 | | IL1B, VEGFA, ITGB3, FLRT2, TGFA, ESM1, EFEMP1, FAM83B |  |
| Cellular Response To Gonadotropin Stimulus | 8 | 5 | 1.35E-03 | 0.016 | | HRASLS, AADAC, PLCB1, PLCB4, LIPG, CHRM3, F2RL2, FAM83B |  |
| Platelet Alpha Granule Lumen | 6 | 6 | 1.64E-03 | 0.013 | | NRP2, TGFBR3, EPHA7, EPHA3, LTBP1, EFEMP1 |  |
| Cellular Response To Cytokine Stimulus | 2 | 40 | 1.77E-03 | 0.005 | | F2RL1, F2RL2 |  |
| Cytoplasmic Vesicle | 2 | 40 | 1.77E-03 | 0.005 | | ITGB3, THBS1 |  |
| Extrinsic Apoptotic Signaling Pathway | 23 | 2 | 2.23E-03 | 0.019 | | ATP2B4, HIST2H4B, BCL2A1, GJA1, ROBO1, IL1B, CARD8, SCN5A, SORBS2, FOXA1, CASK, RAB27B, CRADD, MEF2C, SDC2, PMEPA1, ASAP1, AFAP1L2, PTPN22, WT1, LPAR1, TGFBR3, TGM2 |  |
| Fever Generation | 3 | 15 | 2.34E-03 | 0.008 | | BAIAP2L1, STXBP6, PDLIM1 |  |
| Inorganic Ion Homeostasis | 4 | 9 | 2.88E-03 | 0.010 | | BAIAP2L1, DSP, STXBP6, PDLIM1 |  |
| Regulation Of System Process | 20 | 2 | 3.13E-03 | 0.018 | | PLK2, PIK3CG, CDK15, NRP2, TRIB2, CCL2, CDC42BPA, CASK, IRAK3, CCND2, MAP3K1, NRK, LTBP1, SHC3, EFNB2, TGFBR3, EPHA7, EPHA3, NUAK1, EFEMP1 |  |
| Positive Regulation Of Glomerular Mesangial Cell Proliferation | 45 | 2 | 3.16E-03 | 0.018 | | ARRB1, ABCA1, PIK3CG, CLU, SCP2, CCL2, LAMA1, BMP4, BMP1, IL24, ESM1, SYTL3, CSF1, FGF13, VEGFA, S1PR3, FLRT2, EFNB2, TGFA, TGFB2, THBS1, TGFBR3, EPHA7, F2RL1, MPP4, EFEMP1, GRB14, HLA-A, HLA-B, GJA1, IGFBP4, IL1B, INHBB, ITGB3, CASK, TNFSF10, GMFG, KISS1, LAMA3, LAMA4, LCP1, SHC3, CLIC6, NEO1, FAM83B |  |
| Epithelial-Mesenchymal Cell Signaling | 10 | 3 | 3.17E-03 | 0.016 | | CSF1, IL1B, INHBB, VEGFA, CCL2, BMP4, BMP1, IL24, TGFB2, TNFSF10 |  |
| Mitogen-Activated Protein Kinase Kinase Binding | 52 | 1 | 3.47E-03 | 0.018 | | ARHGDIB, ARRB1, PKIA, ATP2B4, PDE3B, ABCA1, PLCB4, CNKSR2, CLU, SCN5A, SYTL3, DOCK10, CCND2, DMD, DPYSL2, DSP, TRIM6, PLCB1, TBC1D30, PTPN22, TIAM2, TGM2, TIMP3, STOM, EPHA7, EPHA3, F3, GRB14, GATA6, GBP1, EVI5, GJA1, CXCR4, TRIB2, ITGB3, TOR1AIP2, STXBP6, SAMD5, HES1, SH3RF2, CRADD, MEF2C, MAP3K1, KCNQ3, SNX10, LCP1, PARD6B, BCL11A, SHC3, OPTN, RAPGEF5, FAM83B |  |
| Negative Regulation Of Metabolic Process | 3 | 13 | 3.53E-03 | 0.008 | | THBS1, TGFBR3, LTBP1 |  |
| Nitric-Oxide Synthase Binding | 2 | 28 | 3.65E-03 | 0.005 | | EPHA7, EPHA3 |  |
| Positive Regulation Of Lipid Catabolic Process | 5 | 6 | 3.66E-03 | 0.011 | | ATP8B2, PITPNC1, SCP2, ABCA1, PLTP |  |
| Negative Regulation Of Stem Cell Differentiation | 5 | 6 | 3.66E-03 | 0.011 | | ARRB1, MAP3K1, TRIB2, EPHA7, EPHA3 |  |
| Positive Regulation Of Protein Acetylation | 3 | 12 | 3.99E-03 | 0.008 | | DMD, ATP2B4, SCN5A |  |
| Regulation Of Nik/Nf-Kappab Signaling | 24 | 2 | 4.50E-03 | 0.018 | | PLK2, PIK3CG, AK4, CDK15, PLAU, NRP2, TRIB2, CCL2, CDC42BPA, CASK, IRAK3, CCND2, MAP3K1, NRK, PIK3AP1, LTBP1, RAD50, SHC3, EFNB2, TGFBR3, EPHA7, EPHA3, NUAK1, EFEMP1 |  |
| Tube Formation | 4 | 7 | 4.59E-03 | 0.010 | | CD22, ITGB3, TGFBR3, CXCR4 |  |
| Camera-Type Eye Morphogenesis | 2 | 25 | 4.82E-03 | 0.005 | | VEGFA, ITGB3 |  |
| Macromolecule Modification | 45 | 1 | 4.91E-03 | 0.017 | | ARHGDIB, ARRB1, PKIA, ATP2B4, BCL2A1, CARD8, CCL2, BMP4, BMP1, IL24, DOCK10, RGS10, CCND2, ARL2BP, CSF1, PLCB1, CST4, TBC1D30, FGF13, TIAM2, VEGFA, FLRT2, TGFA, TGFB2, TIMP3, EPHA7, LYPD6B, EFEMP1, NCKAP1L, EVI5, IL1B, INHBB, TRIB2, TOR1AIP2, CST7, SH3RF2, SERPINB7, TNFSF10, GMFG, KCNIP3, ASAP1, SHC3, AFAP1L2, SERPINB2, RAPGEF5 |  |
| Cation Homeostasis | 3 | 11 | 5.03E-03 | 0.008 | | RAD50, CASK, AK4 |  |
| Regulation Of Proteolysis | 14 | 2 | 5.40E-03 | 0.017 | | GPR1, SLC7A5, PTGES, CEMIP, HLA-A, HLA-B, CTSD, PLTP, ASAP1, ITGB3, CLU, SCP2, HLA-DRA, TGFB2 |  |
| Coagulation | 2 | 22 | 6.14E-03 | 0.005 | | TGM2, F13A1 |  |
| Regulation Of Muscle Tissue Development | 2 | 22 | 6.14E-03 | 0.005 | | RAD50, AK4 |  |
| Cellular Protein Metabolic Process | 2 | 22 | 6.14E-03 | 0.005 | | TGFB2, TGFBR3 |  |
| Cellular Response To Interferon-Gamma | 3 | 10 | 6.21E-03 | 0.008 | | IGFBP4, ITGB3, ESM1 |  |
| Regulation Of Calcium Ion Transport Into Cytosol | 4 | 7 | 7.32E-03 | 0.010 | | INHBB, BMP4, TGFB2, TGFBR3 |  |
| Positive Regulation Of Lipid Localization | 6 | 4 | 7.33E-03 | 0.012 | | ATP2B4, SDC2, GJA1, LPAR1, TGFBR3, CASK |  |
| Positive Regulation Of Proteolysis | 16 | 2 | 7.54E-03 | 0.017 | | ARHGDIB, ARRB1, NCKAP1L, EVI5, CARD8, TOR1AIP2, CCL2, RGS10, GMFG, ARL2BP, PLCB1, TBC1D30, ASAP1, FGF13, AFAP1L2, TIAM2 |  |
| Secretory Granule Membrane | 3 | 10 | 7.54E-03 | 0.008 | | MAP3K1, LAMA1, PLTP |  |
| Cell Junction Assembly | 2 | 20 | 7.60E-03 | 0.005 | | PITPNC1, SCP2 |  |
| Regulation Of Supramolecular Fiber Organization | 10 | 3 | 7.68E-03 | 0.015 | | LPCAT2, ARRB1, PORCN, CPT1A, ELOVL7, ZDHHC15, SCP2, HMGCS1, TGM2, F13A1 |  |
| Vasculature Development | 17 | 2 | 7.75E-03 | 0.017 | | IL1B, INHBB, CCL2, BMP4, BMP1, IL24, TNFSF10, GMFG, CSF1, FGF13, VEGFA, FLRT2, TGFA, TGFB2, EPHA7, LYPD6B, EFEMP1 |  |
| Response To Nitric Oxide | 16 | 2 | 7.77E-03 | 0.017 | | IL1B, INHBB, CCL2, BMP4, BMP1, IL24, TNFSF10, GMFG, CSF1, FGF13, VEGFA, FLRT2, TGFA, TGFB2, EPHA7, EFEMP1 |  |
| Ether Lipid Metabolic Process | 12 | 3 | 8.16E-03 | 0.016 | | GPR1, SLC7A5, PTGES, CEMIP, HLA-A, HLA-B, CTSD, ASAP1, ITGB3, CLU, HLA-DRA, TGFB2 |  |
| Smooth Muscle Cell Migration | 10 | 3 | 8.21E-03 | 0.015 | | ARHGDIB, ARRB1, ARL2BP, NCKAP1L, EVI5, PLCB1, TBC1D30, ASAP1, TIAM2, RGS10 |  |
| Ovarian Follicle Development | 2 | 18 | 9.21E-03 | 0.005 | | ROBO1, EPHA7 |  |
| Positive Regulation Of Heart Growth | 2 | 18 | 9.21E-03 | 0.005 | | ARRB1, GPR135 |  |
| Regulation Of Cell Growth | 51 | 1 | 9.38E-03 | 0.017 | | LPCAT2, PLK2, ARRB1, PIK3CG, AK4, PLAU, MGAT4A, FBXO32, MARCH3, CLIC2, SCP2, CCL2, IRAK3, CCND2, HRASLS, MARCH4, TRIM6, PIK3AP1, CPT1A, PRDM9, PRDM8, UGCG, EFNB2, TGFBR3, TGM2, EPHA7, EPHA3, F13A1, EFEMP1, HAS2, PORCN, CDK15, ELOVL7, NRP2, ZDHHC15, TRIB2, CDC42BPA, HMGCS1, COLGALT1, CASK, SH3RF2, PTGES, MAP3K1, NRK, B4GALT6, LTBP1, METTL7B, RAD50, SHC3, CHST15, NUAK1 |  |
| Phosphotransferase Activity, Alcohol Group As Acceptor | 15 | 2 | 9.43E-03 | 0.016 | | TENM3, CDH11, IL1B, ITGB3, BAIAP2L1, GPRC5A, STXBP6, ESM1, DSP, PDLIM1, LCP1, ASAP1, S1PR3, THBS1, NEO1 |  |
| Cardiac Chamber Formation | 3 | 9 | 9.84E-03 | 0.008 | | EFNB2, EPHA7, PIK3CG |  |
| Phosphotransferase activity, alcohol group as acceptor | 21 | 2 | 9.95E-03 | 0.017 | | PLK2, PIK3CG, CDK15, NRP2, TRIB2, CCL2, CDC42BPA, CASK, IRAK3, CCND2, MAP3K1, NRK, PIK3AP1, LTBP1, SHC3, EFNB2, TGFBR3, EPHA7, EPHA3, NUAK1, EFEMP1 |  |
