# Supplementary material for: LAMA4-Regulating miR-4274 and Its Host Gene SORCS2 Play a Role in IGFBP6-Dependent Effects on Phenotype of Basal-Like Breast Cancer
Source: Front Mol Biosci. 2019 Nov 8;6:122. doi: 10.3389/fmolb.2019.00122 (PMC6857517; doi:10.3389/fmolb.2019.00122)
Supplement: Supplementary file 2 [file Table_2.DOCX]

**Supplementary Table 2.** Enrichment analysis of a set of genes significantly overexpressed after a transcriptional knockdown of IGFBP6 gene in MDA-MB-231 cells (N=197) (FDR p 0:01, fold change 2).

| **Ontology Component** | **Genes Overlap** | **Percent Overlap** | **P-value** | **Jaccard similarity** | **List of overlapping genes** |  |
| --- | --- | --- | --- | --- | --- | --- |
| **Enrichment for Biological Processes** | | | | | |  |
| Cell Adhesion | 24 | 2 | 2.23E-09 | 0.021 | PCDH17, PCDH11X, SRPX, PGM5, PIK3CG, CD33, ROBO1, NRP2, IL1B, STXBP6, CCL2, PCDHB6, LAMA1, PCDH11Y, PCDHB2, LAMA4, PLCB1, ADGRL3, PCDHB5, CNTN5, FLRT2, PCDHB16, EPHA3, NEO1 |  |
| Biological Adhesion | 24 | 2 | 3.01E-09 | 0.021 | PCDH17, PCDH11X, SRPX, PGM5, PIK3CG, CD33, ROBO1, NRP2, IL1B, STXBP6, CCL2, PCDHB6, LAMA1, PCDH11Y, PCDHB2, LAMA4, PLCB1, ADGRL3, PCDHB5, CNTN5, FLRT2, PCDHB16, EPHA3, NEO1 |  |
| Regulation Of Cell Migration | 21 | 2 | 7.38E-07 | 0.017 | ARHGDIB, NCKAP1L, CXCR4, NAV3, PLAU, PLXNA2, ROBO1, NRP2, IL1B, CCL2, LAMA1, IL24, DOCK10, MMP3, KISS1, LAMA4, PLCB1, PTPN22, FLRT2, EPHA3, NEO1 |  |
| Regulation Of Catalytic Activity | 36 | 1 | 1.14E-06 | 0.013 | ARHGDIB, PKIA, ARRDC4, BCL2A1, PIK3CG, PLXNA2, ROBO1, CARD8, CCL2, HMSD, IL24, DOCK10, IRAK3, RGS10, CCND2, PLCB1, TBC1D30, FGF13, PTPN22, EPHA7, EPHA3, PDCD4, PCDH11X, NCKAP1L, CXCR4, TFPI2, IL1B, TOR1AIP2, GPR65, LGR5, SAMD5, SERPINB7, CRADD, GMFG, NRK, SERPINB2 |  |
| Regulation Of Signal Transduction | 43 | 1 | 2.04E-06 | 0.011 | ARHGDIB, BCL2A1, CNKSR2, CARD8, CLIC2, IL24, ESM1, IRAK3, DMD, PIK3AP1, PLCB1, CYP2J2, FGF13, PTPN22, EPHA7, EPHA3, PDCD4, GRB14, GJA1, IL1B, FOXA1, MMP3, NRK, KISS1, G0S2, NEO1, PDE3B, PIK3CG, PLAU, ROBO1, LMO3, CCL2, RGS10, TCF4, SAMSN1, SRPX, CXCR4, GPR65, LGR5, SAMD5, CRADD, GMFG, IL1RL1 |  |
| Regulation Of Cell Motility | 21 | 1 | 2.10E-06 | 0.016 | ARHGDIB, NCKAP1L, CXCR4, NAV3, PLAU, PLXNA2, ROBO1, NRP2, IL1B, CCL2, LAMA1, IL24, DOCK10, MMP3, KISS1, LAMA4, PLCB1, PTPN22, FLRT2, EPHA3, NEO1 |  |
| Regulation Of Localization | 39 | 1 | 4.16E-06 | 0.012 | ARHGDIB, NAV3, CARD8, CLIC2, IL24, SYTL3, DMD, PLCB1, CYP2J2, PTPN22, FLRT2, STOM, EPHA3, PCDH17, NCKAP1L, GJA1, IL1B, IL13RA2, STXBP6, MMP3, KCNQ3, KISS1, LAMA4, NEO1, PKIA, PDE3B, PIK3CG, PLAU, PLCB4, PLXNA2, PLTP, ROBO1, MCTP2, CCL2, LAMA1, DOCK10, CXCR4, NRP2, IL1RL1 |  |
| Regulation Of Multicellular Organismal Process | 43 | 1 | 4.61E-06 | 0.011 | ARHGDIB, NAV3, SLITRK1, CARD8, CHRM3, CLIC2, IRAK3, CCND2, DMD, PLCB1, ADGRL3, CYP2J2, FGF13, PTPN22, S1PR3, FLRT2, EPHA7, EPHA3, PDCD4, GRB14, NCKAP1L, GJA1, IL1B, FOXA1, NRK, KISS1, LAMA4, BCL11A, SERPINB2, NEO1, PDE3B, PIK3CG, PLAU, PLXNA2, ROBO1, LAMA1, TBXAS1, TCF4, HIST2H4B, CXCR4, NRP2, SERPINB7, IL1RL1 |  |
| Nervous System Development | 14 | 2 | 5.99E-06 | 0.019 | TCF4, PCDHB2, CXCR4, NAV3, PRDM8, ROBO1, FGF13, NRP2, SLITRK1, CHRM3, PCDHB6, RAPGEF5, EPHA7, NEO1 |  |
| Regulation Of Locomotion | 21 | 1 | 6.57E-06 | 0.015 | ARHGDIB, NCKAP1L, CXCR4, NAV3, PLAU, PLXNA2, ROBO1, NRP2, IL1B, CCL2, LAMA1, IL24, DOCK10, MMP3, KISS1, LAMA4, PLCB1, PTPN22, FLRT2, EPHA3, NEO1 |  |
| Regulation Of Cellular Component Movement | 21 | 1 | 7.18E-06 | 0.015 | ARHGDIB, NCKAP1L, CXCR4, NAV3, PLAU, PLXNA2, ROBO1, NRP2, IL1B, CCL2, LAMA1, IL24, DOCK10, MMP3, KISS1, LAMA4, PLCB1, PTPN22, FLRT2, EPHA3, NEO1 |  |
| Cell-Cell Signaling | 16 | 2 | 7.20E-06 | 0.018 | PCDH17, PCDH11X, GJA1, CD33, IL1B, CHRM3, CCL2, PCDHB6, FOXA1, PCDH11Y, PCDHB2, KCNQ3, FGF13, SHC3, PCDHB5, PCDHB16 |  |
| Synapse Assembly | 6 | 7 | 7.53E-06 | 0.023 | PCDHB5, SLITRK1, PCDHB6, PCDHB2, PCDHB16, ADGRL3 |  |
| Negative Regulation Of Multicellular Organismal Process | 24 | 1 | 9.14E-06 | 0.014 | ARHGDIB, PDE3B, PIK3CG, NAV3, PLAU, ROBO1, CARD8, IRAK3, TCF4, FGF13, PTPN22, EPHA7, EPHA3, PDCD4, GRB14, HIST2H4B, NCKAP1L, GJA1, IL1B, FOXA1, IL1RL1, BCL11A, SERPINB2, NEO1 |  |
| Homophilic Cell Adhesion Via Plasma Membrane Adhesion Molecules | 8 | 4 | 9.47E-06 | 0.023 | ROBO1, PCDH17, PCDH11X, PCDHB5, PCDH11Y, PCDHB6, PCDHB2, PCDHB16 |  |
| Regulation Of Molecular Function | 42 | 1 | 1.05E-05 | 0.011 | ARHGDIB, ARRDC4, BCL2A1, CARD8, CLIC2, IL24, IRAK3, CCND2, DMD, PLCB1, TBC1D30, FGF13, PTPN22, STOM, EPHA7, EPHA3, PDCD4, PCDH11X, NCKAP1L, GJA1, IL1B, TOR1AIP2, FOXA1, NRK, SERPINB2, PKIA, PIK3CG, PLAU, PLXNA2, ROBO1, CCL2, HMSD, DOCK10, RGS10, CXCR4, TFPI2, GPR65, LGR5, SAMD5, SERPINB7, CRADD, GMFG |  |
| Positive Regulation Of Molecular Function | 28 | 1 | 1.09E-05 | 0.013 | ARHGDIB, ARRDC4, PIK3CG, ROBO1, CARD8, CLIC2, CCL2, IL24, DOCK10, IRAK3, RGS10, CCND2, DMD, PLCB1, TBC1D30, FGF13, EPHA7, EPHA3, NCKAP1L, CXCR4, IL1B, TOR1AIP2, GPR65, FOXA1, SAMD5, CRADD, GMFG, NRK |  |
| Regulation Of Cell Communication | 45 | 1 | 1.53E-05 | 0.010 | ARHGDIB, BCL2A1, CNKSR2, CARD8, CLIC2, IL24, ESM1, IRAK3, DMD, PIK3AP1, PLCB1, CYP2J2, FGF13, PTPN22, EPHA7, EPHA3, PDCD4, PCDH17, GRB14, GJA1, IL1B, FOXA1, MMP3, NRK, KISS1, G0S2, NEO1, PDE3B, PIK3CG, PLAU, ROBO1, MCTP2, LMO3, CCL2, RGS10, TCF4, SAMSN1, SRPX, CXCR4, GPR65, LGR5, SAMD5, CRADD, GMFG, IL1RL1 |  |
| Regulation Of Cell Adhesion | 16 | 2 | 1.59E-05 | 0.017 | ARHGDIB, NCKAP1L, PDE3B, PIK3CG, PLAU, PLXNA2, IL1B, CCL2, LAMA1, FOXA1, DMD, LAMA4, PTPN22, EPHA7, EPHA3, ABI3BP |  |
| Positive Regulation Of Catalytic Activity | 24 | 1 | 1.60E-05 | 0.014 | ARHGDIB, ARRDC4, PIK3CG, ROBO1, CARD8, CCL2, IL24, DOCK10, RGS10, CCND2, PLCB1, TBC1D30, FGF13, EPHA7, EPHA3, NCKAP1L, CXCR4, IL1B, TOR1AIP2, GPR65, SAMD5, CRADD, GMFG, NRK |  |
| Negative Regulation Of Cellular Process | 56 | 0 | 1.66E-05 | 0.010 | ARHGDIB, BCL2A1, NAV3, CD33, CARD8, CLIC2, IL24, IRAK3, CCND2, DMD, PIK3AP1, PLCB1, TBC1D30, CYP2J2, FGF13, PTPN22, S1PR3, EPHA7, EPHA3, FAM172BP, PDCD4, PCDH17, GRB14, PCDH11X, NCKAP1L, GJA1, IL1B, IL13RA2, STXBP6, FOXA1, MMP3, NRK, KISS1, BCL11A, SERPINB2, ZNF438, NEO1, PKIA, PDE3B, PIK3CG, PLXNA2, ROBO1, LMO3, CCL2, HMSD, RGS10, TCF4, PRDM8, SAMSN1, HIST2H4B, SRPX, TFPI2, SERPINB7, CRADD, GMFG, IL1RL1 |  |
| Regulation Of Signaling | 45 | 1 | 1.80E-05 | 0.010 | ARHGDIB, BCL2A1, CNKSR2, CARD8, CLIC2, IL24, ESM1, IRAK3, DMD, PIK3AP1, PLCB1, CYP2J2, FGF13, PTPN22, EPHA7, EPHA3, PDCD4, PCDH17, GRB14, GJA1, IL1B, FOXA1, MMP3, NRK, KISS1, G0S2, NEO1, PDE3B, PIK3CG, PLAU, ROBO1, MCTP2, LMO3, CCL2, RGS10, TCF4, SAMSN1, SRPX, CXCR4, GPR65, LGR5, SAMD5, CRADD, GMFG, IL1RL1 |  |
| Cell Communication | 19 | 1 | 1.95E-05 | 0.015 | PCDH17, PCDH11X, GJA1, CD33, IL1B, CHRM3, CCL2, PCDHB6, FOXA1, PCDH11Y, MMP3, PCDHB2, KCNQ3, FGF13, BCL11A, SHC3, PCDHB5, GDAP1, PCDHB16 |  |
| Positive Regulation Of Signal Transduction | 26 | 1 | 2.11E-05 | 0.013 | PIK3CG, ROBO1, LMO3, CCL2, IL24, ESM1, PIK3AP1, PLCB1, PTPN22, EPHA7, EPHA3, PDCD4, GRB14, SRPX, GJA1, CXCR4, IL1B, GPR65, FOXA1, LGR5, SAMD5, CRADD, NRK, KISS1, G0S2, NEO1 |  |
| Signaling | 16 | 1 | 2.32E-05 | 0.016 | PCDH17, PCDH11X, GJA1, CD33, IL1B, CHRM3, CCL2, PCDHB6, FOXA1, PCDH11Y, PCDHB2, KCNQ3, FGF13, SHC3, PCDHB5, PCDHB16 |  |
| Regulation Of Mapk Cascade | 17 | 1 | 2.35E-05 | 0.016 | PDCD4, CXCR4, PIK3CG, ROBO1, IL1B, LMO3, CCL2, SAMD5, IRAK3, DMD, NRK, KISS1, PIK3AP1, PLCB1, PTPN22, EPHA7, EPHA3 |  |
| Cell-Cell Adhesion Via Plasma-Membrane Adhesion Molecules | 9 | 3 | 2.38E-05 | 0.021 | PCDH17, PCDH11X, PCDH11Y, PCDHB2, ADGRL3, ROBO1, PCDHB5, PCDHB6, PCDHB16 |  |
| Negative Regulation Of Response To Stimulus | 26 | 1 | 2.89E-05 | 0.013 | BCL2A1, PDE3B, PLAU, ROBO1, CARD8, LMO3, CLIC2, CCL2, IRAK3, RGS10, DMD, PIK3AP1, CYP2J2, PTPN22, SAMSN1, EPHA7, EPHA3, PDCD4, GRB14, GJA1, IL1B, IL13RA2, MMP3, IL1RL1, SERPINB2, NEO1 |  |
| Regulation Of Protein Phosphorylation | 24 | 1 | 4.06E-05 | 0.013 | PKIA, PIK3CG, ROBO1, LMO3, CCL2, IL24, IRAK3, CCND2, DMD, PIK3AP1, PLCB1, FGF13, PTPN22, SAMSN1, EPHA7, EPHA3, PDCD4, NCKAP1L, CXCR4, IL1B, SAMD5, GMFG, NRK, KISS1 |  |
| Negative Regulation Of Collateral Sprouting | 3 | 23 | 5.35E-05 | 0.016 | FGF13, BCL11A, EPHA7 |  |
| Cell-Cell Adhesion | 12 | 2 | 5.88E-05 | 0.017 | PCDH17, PCDH11X, PCDH11Y, PCDHB2, PIK3CG, ADGRL3, ROBO1, PCDHB5, IL1B, STXBP6, PCDHB6, PCDHB16 |  |
| Locomotion | 20 | 1 | 6.70E-05 | 0.014 | GRB14, NCKAP1L, GJA1, CXCR4, PIK3CG, PLAU, PLXNA2, PLTP, EFHC1, ROBO1, NRP2, IL1B, CCL2, MAFIP, ADGRL3, FGF13, FLRT2, EPHA7, EPHA3, NEO1 |  |
| Regulation Of Response To Stimulus | 49 | 0 | 6.99E-05 | 0.010 | ARHGDIB, BCL2A1, CD33, CNKSR2, CARD8, CLIC2, IL24, ESM1, IRAK3, DMD, PIK3AP1, PLCB1, CYP2J2, FGF13, PTPN22, EPHA7, EPHA3, PDCD4, GRB14, NCKAP1L, GJA1, IL1B, IL13RA2, FOXA1, MMP3, NRK, KISS1, G0S2, SERPINB2, NEO1, PDE3B, PIK3CG, PLAU, ROBO1, LMO3, CCL2, HMSD, RGS10, TCF4, SAMSN1, SRPX, CXCR4, GPR65, LGR5, SAMD5, CRADD, GMFG, IL1RL1, CLEC2B |  |
| Regulation Of Hydrolase Activity | 22 | 1 | 7.01E-05 | 0.013 | ARHGDIB, BCL2A1, PLXNA2, ROBO1, CARD8, CCL2, HMSD, IL24, DOCK10, RGS10, PLCB1, TBC1D30, EPHA7, EPHA3, PCDH11X, NCKAP1L, TFPI2, TOR1AIP2, GPR65, SERPINB7, CRADD, SERPINB2 |  |
| Negative Chemotaxis | 4 | 10 | 8.67E-05 | 0.018 | FLRT2, ROBO1, NRP2, EPHA7 |  |
| Chemotaxis | 10 | 2 | 9.19E-05 | 0.018 | NCKAP1L, CXCR4, PIK3CG, PLAU, ROBO1, NRP2, IL1B, CCL2, FLRT2, EPHA7 |  |
| Taxis | 10 | 2 | 9.59E-05 | 0.018 | NCKAP1L, CXCR4, PIK3CG, PLAU, ROBO1, NRP2, IL1B, CCL2, FLRT2, EPHA7 |  |
| Regulation Of Intracellular Signal Transduction | 27 | 1 | 1.19E-04 | 0.012 | ARHGDIB, BCL2A1, PDE3B, PIK3CG, ROBO1, CARD8, LMO3, CLIC2, CCL2, IL24, IRAK3, DMD, PIK3AP1, PLCB1, PTPN22, EPHA7, EPHA3, PDCD4, GJA1, CXCR4, IL1B, GPR65, SAMD5, MMP3, NRK, IL1RL1, KISS1 |  |
| Apoptotic Process | 15 | 1 | 1.21E-04 | 0.015 | PDCD4, BCL2A1, GJA1, CXCR4, PIK3CG, IL1B, CARD8, GPR65, IL24, IRAK3, CRADD, EPB41L3, G0S2, FGF13, EPHA7 |  |
| Regulation Of Phosphorylation | 24 | 1 | 1.22E-04 | 0.012 | PKIA, PIK3CG, ROBO1, LMO3, CCL2, IL24, IRAK3, CCND2, DMD, PIK3AP1, PLCB1, FGF13, PTPN22, SAMSN1, EPHA7, EPHA3, PDCD4, NCKAP1L, CXCR4, IL1B, SAMD5, GMFG, NRK, KISS1 |  |
| Synapse Organization | 7 | 3 | 1.22E-04 | 0.019 | ROBO1, PCDHB5, SLITRK1, PCDHB6, PCDHB2, PCDHB16, ADGRL3 |  |
| Regulation Of Multicellular Organismal Development | 30 | 1 | 1.32E-04 | 0.011 | ARHGDIB, PDE3B, PLAU, PLXNA2, ROBO1, SLITRK1, LAMA1, IRAK3, CCND2, DMD, TCF4, PLCB1, ADGRL3, FGF13, S1PR3, FLRT2, EPHA7, EPHA3, PDCD4, HIST2H4B, NCKAP1L, GJA1, CXCR4, IL1B, FOXA1, SERPINB7, NRK, LAMA4, BCL11A, NEO1 |  |
| Regulation Of Transport | 27 | 1 | 1.45E-04 | 0.012 | PKIA, PDE3B, PIK3CG, PLCB4, PLTP, MCTP2, CARD8, CLIC2, CCL2, SYTL3, DMD, PLCB1, CYP2J2, PTPN22, STOM, EPHA3, PCDH17, NCKAP1L, GJA1, CXCR4, IL1B, IL13RA2, STXBP6, KCNQ3, IL1RL1, KISS1, NEO1 |  |
| Axon Guidance | 8 | 3 | 1.58E-04 | 0.018 | CXCR4, ROBO1, NRP2, SHC3, FLRT2, EPHA7, EPHA3, NEO1 |  |
| Regulation Of Cell Adhesion Mediated By Integrin | 4 | 8 | 1.64E-04 | 0.018 | PDE3B, PIK3CG, PLAU, NCKAP1L |  |
| Positive Regulation Of Multicellular Organismal Process | 26 | 1 | 1.65E-04 | 0.012 | PLAU, PLXNA2, ROBO1, SLITRK1, CARD8, CHRM3, IRAK3, DMD, TBXAS1, TCF4, PLCB1, ADGRL3, CYP2J2, PTPN22, FLRT2, EPHA3, NCKAP1L, GJA1, CXCR4, NRP2, IL1B, FOXA1, SERPINB7, IL1RL1, KISS1, BCL11A |  |
| Neuron Projection Guidance | 8 | 3 | 1.71E-04 | 0.018 | CXCR4, ROBO1, NRP2, SHC3, FLRT2, EPHA7, EPHA3, NEO1 |  |
| G-Protein Coupled Acetylcholine Receptor Signaling Pathway | 3 | 15 | 1.77E-04 | 0.015 | PLCB1, CHRM3, RGS10 |  |
| Positive Regulation Of Response To Stimulus | 32 | 1 | 1.97E-04 | 0.011 | BCL2A1, PIK3CG, ROBO1, LMO3, CCL2, HMSD, IL24, ESM1, IRAK3, PIK3AP1, PLCB1, PTPN22, EPHA7, EPHA3, PDCD4, GRB14, SRPX, NCKAP1L, GJA1, CXCR4, IL1B, GPR65, FOXA1, LGR5, SAMD5, CRADD, MMP3, NRK, IL1RL1, KISS1, G0S2, NEO1 |  |
| Negative Regulation Of Branching Morphogenesis Of A Nerve | 2 | 50 | 2.02E-04 | 0.011 | BCL11A, IL1B |  |
| Regulation Of Kinase Activity | 16 | 1 | 2.17E-04 | 0.014 | PDCD4, PKIA, NCKAP1L, CXCR4, PIK3CG, ROBO1, IL1B, SAMD5, IRAK3, CCND2, GMFG, NRK, FGF13, PTPN22, EPHA7, EPHA3 |  |
| Cell Motility | 18 | 1 | 2.18E-04 | 0.013 | GRB14, NCKAP1L, GJA1, CXCR4, PIK3CG, PLAU, PLXNA2, PLTP, EFHC1, ROBO1, NRP2, IL1B, CCL2, MAFIP, ADGRL3, FGF13, EPHA3, NEO1 |  |
| Calcium-Dependent Cell-Cell Adhesion Via Plasma Membrane Cell Adhesion Molecules | 4 | 7 | 2.43E-04 | 0.017 | PCDHB2, PCDHB5, PCDHB16, PCDHB6 |  |
| Telencephalon Cell Migration | 4 | 7 | 2.43E-04 | 0.017 | EFHC1, FGF13, NRP2, CXCR4 |  |
| Regulation Of Transferase Activity | 17 | 1 | 2.48E-04 | 0.013 | PDCD4, PKIA, ARRDC4, NCKAP1L, CXCR4, PIK3CG, ROBO1, IL1B, SAMD5, IRAK3, CCND2, GMFG, NRK, FGF13, PTPN22, EPHA7, EPHA3 |  |
| Neuron Migration | 6 | 3 | 2.57E-04 | 0.018 | FGF13, NRP2, GJA1, CXCR4, ADGRL3, NEO1 |  |
| Negative Regulation Of Intracellular Signal Transduction | 12 | 1 | 2.79E-04 | 0.015 | PDCD4, DMD, MMP3, BCL2A1, PDE3B, IL1RL1, PTPN22, IL1B, CARD8, LMO3, CLIC2, IRAK3 |  |
| Movement Of Cell Or Subcellular Component | 22 | 1 | 2.95E-04 | 0.012 | PIK3CG, PLAU, PLXNA2, PLTP, EFHC1, ROBO1, CCL2, DMD, ADGRL3, FGF13, FLRT2, EPHA7, EPHA3, GRB14, NCKAP1L, GJA1, CXCR4, NRP2, IL1B, MAFIP, SHC3, NEO1 |  |
| Regulation Of Phosphate Metabolic Process | 25 | 1 | 2.97E-04 | 0.011 | PKIA, PIK3CG, ROBO1, LMO3, CCL2, IL24, IRAK3, CCND2, DMD, PIK3AP1, PLCB1, FGF13, PTPN22, SAMSN1, EPHA7, EPHA3, PDCD4, PCDH11X, NCKAP1L, CXCR4, IL1B, SAMD5, GMFG, NRK, KISS1 |  |
| Regulation Of Phosphorus Metabolic Process | 25 | 1 | 2.99E-04 | 0.011 | PKIA, PIK3CG, ROBO1, LMO3, CCL2, IL24, IRAK3, CCND2, DMD, PIK3AP1, PLCB1, FGF13, PTPN22, SAMSN1, EPHA7, EPHA3, PDCD4, PCDH11X, NCKAP1L, CXCR4, IL1B, SAMD5, GMFG, NRK, KISS1 |  |
| Forebrain Cell Migration | 4 | 7 | 3.02E-04 | 0.017 | EFHC1, FGF13, NRP2, CXCR4 |  |
| Regulation Of Neuron Differentiation | 14 | 1 | 3.15E-04 | 0.014 | CXCR4, PLXNA2, ROBO1, SLITRK1, IL1B, FOXA1, CCND2, DMD, TCF4, FGF13, BCL11A, EPHA7, EPHA3, NEO1 |  |
| Positive Regulation Of Cell Communication | 26 | 1 | 3.47E-04 | 0.011 | PIK3CG, ROBO1, LMO3, CCL2, IL24, ESM1, PIK3AP1, PLCB1, PTPN22, EPHA7, EPHA3, PDCD4, GRB14, SRPX, GJA1, CXCR4, IL1B, GPR65, FOXA1, LGR5, SAMD5, CRADD, NRK, KISS1, G0S2, NEO1 |  |
| Regulation Of Developmental Process | 34 | 1 | 3.57E-04 | 0.010 | ARHGDIB, SLITRK1, IRAK3, CCND2, DMD, EPB41L3, PLCB1, ADGRL3, FGF13, S1PR3, FLRT2, EPHA7, EPHA3, PDCD4, GRB14, NCKAP1L, GJA1, IL1B, FOXA1, NRK, LAMA4, BCL11A, NEO1, PDE3B, PLAU, PLXNA2, ROBO1, LMO3, CCL2, LAMA1, TCF4, HIST2H4B, CXCR4, SERPINB7 |  |
| Positive Regulation Of Signaling | 26 | 1 | 3.65E-04 | 0.011 | PIK3CG, ROBO1, LMO3, CCL2, IL24, ESM1, PIK3AP1, PLCB1, PTPN22, EPHA7, EPHA3, PDCD4, GRB14, SRPX, GJA1, CXCR4, IL1B, GPR65, FOXA1, LGR5, SAMD5, CRADD, NRK, KISS1, G0S2, NEO1 |  |
| Cell Death | 16 | 1 | 3.76E-04 | 0.013 | PDCD4, BCL2A1, GJA1, CXCR4, PIK3CG, PLAU, IL1B, CARD8, GPR65, IL24, IRAK3, CRADD, EPB41L3, G0S2, FGF13, EPHA7 |  |
| Intracellular Signal Transduction | 23 | 1 | 3.81E-04 | 0.012 | ARHGDIB, BCL2A1, PIK3CG, PLCB4, MCTP2, CNKSR2, CLIC2, CCL2, DOCK10, IRAK3, HRASLS, DMD, PLCB1, FGF13, GRB14, CXCR4, IL1B, GPR65, LGR5, CRADD, NRK, SHC3, RAPGEF5 |  |
| Regulation Of Lipid Catabolic Process | 4 | 6 | 3.96E-04 | 0.017 | IL1B, AADAC, PDE3B, PIK3CG |  |
| Positive Regulation Of Mapk Cascade | 12 | 1 | 4.21E-04 | 0.015 | CXCR4, PIK3CG, ROBO1, IL1B, CCL2, SAMD5, NRK, KISS1, PLCB1, PTPN22, EPHA7, EPHA3 |  |
| Regulation Of Cell Development | 17 | 1 | 4.26E-04 | 0.013 | GRB14, CXCR4, PLXNA2, ROBO1, SLITRK1, IL1B, FOXA1, CCND2, DMD, TCF4, FGF13, BCL11A, S1PR3, FLRT2, EPHA7, EPHA3, NEO1 |  |
| Fever Generation | 2 | 33 | 5.01E-04 | 0.011 | IL1B, EPHA3 |  |
| Cell Migration | 16 | 1 | 5.03E-04 | 0.013 | GRB14, NCKAP1L, GJA1, CXCR4, PIK3CG, PLAU, PLXNA2, EFHC1, ROBO1, NRP2, IL1B, CCL2, ADGRL3, FGF13, EPHA3, NEO1 |  |
| Negative Regulation Of Lipid Catabolic Process | 3 | 11 | 5.15E-04 | 0.014 | PDE3B, IL1B, PIK3CG |  |
| Regulation Of Collateral Sprouting | 3 | 11 | 5.15E-04 | 0.014 | FGF13, BCL11A, EPHA7 |  |
| Positive Regulation Of Nervous System Development | 12 | 1 | 5.59E-04 | 0.014 | CXCR4, PLXNA2, ROBO1, SLITRK1, IL1B, FOXA1, DMD, TCF4, ADGRL3, BCL11A, FLRT2, EPHA3 |  |
| Regulation Of Map Kinase Activity | 9 | 2 | 5.80E-04 | 0.016 | PDCD4, NRK, CXCR4, PIK3CG, ROBO1, PTPN22, IL1B, SAMD5, IRAK3 |  |
| Regulation Of Response To External Stimulus | 15 | 1 | 6.08E-04 | 0.013 | PDCD4, NCKAP1L, GJA1, CXCR4, PIK3CG, PLAU, ROBO1, IL1B, CARD8, CCL2, IL1RL1, PIK3AP1, PTPN22, SERPINB2, NEO1 |  |
| Regulation Of Protein Modification Process | 25 | 1 | 6.10E-04 | 0.011 | PKIA, ARRDC4, PIK3CG, ROBO1, LMO3, CCL2, IL24, IRAK3, CCND2, DMD, PIK3AP1, PLCB1, FGF13, PTPN22, SAMSN1, EPHA7, EPHA3, PDCD4, NCKAP1L, CXCR4, IL1B, SAMD5, GMFG, NRK, KISS1 |  |
| System Development | 16 | 1 | 6.21E-04 | 0.013 | CXCR4, NAV3, PLXNA2, ROBO1, NRP2, SLITRK1, CHRM3, PCDHB6, TCF4, PCDHB2, PRDM8, FGF13, SHC3, RAPGEF5, EPHA7, NEO1 |  |
| Negative Regulation Of Biological Process | 58 | 0 | 6.25E-04 | 0.008 | ARHGDIB, BCL2A1, NAV3, CD33, CLIC2, CCND2, DMD, PIK3AP1, CYP2J2, FGF13, S1PR3, EPHA7, EPHA3, FAM172BP, GRB14, NCKAP1L, GJA1, IL1B, IL13RA2, FOXA1, MMP3, NRK, KISS1, SERPINB2, ZNF438, NEO1, PKIA, PDE3B, PIK3CG, PLAU, PLCB4, PLXNA2, ROBO1, CCL2, HMSD, RGS10, TCF4, HIST2H4B, SRPX, TFPI2, SERPINB7, CRADD, GMFG, IL1RL1, CARD8, IL24, IRAK3, PLCB1, TBC1D30, PTPN22, PDCD4, PCDH17, PCDH11X, STXBP6, BCL11A, LMO3, PRDM8, SAMSN1 |  |
| Regulation Of Jun Kinase Activity | 5 | 4 | 6.29E-04 | 0.017 | PDCD4, PTPN22, IL1B, NRK, SAMD5 |  |
| Developmental Process | 56 | 0 | 6.32E-04 | 0.008 | ARHGDIB, BCL2A1, NAV3, SLITRK1, CHRM3, DMKN, CCND2, DMD, FGF13, S1PR3, EPHA7, EPHA3, NCKAP1L, GJA1, IL1B, ZDHHC15, HMGCS1, FOXA1, SHISA3, NRK, KCNQ3, KISS1, LAMA4, ZNF438, NEO1, PDE3B, PIK3CG, PLAU, PLXNA2, ROBO1, CCL2, LAMA1, TCF4, CXCR4, NRP2, GPR65, LGR5, RAPGEF5, ESM1, EPB41L3, PLCB1, ADGRL3, PTPN22, FLRT2, PDCD4, BCL11A, SHC3, KRTAP4-8, MCTP2, PCDHB6, DOCK10, PCDHB2, PRDM8, POF1B, CLMP, CRISPLD1 |  |
| Regulation Of Nervous System Development | 16 | 1 | 6.46E-04 | 0.013 | CXCR4, PLXNA2, ROBO1, SLITRK1, IL1B, FOXA1, CCND2, DMD, TCF4, ADGRL3, FGF13, BCL11A, FLRT2, EPHA7, EPHA3, NEO1 |  |
| Negative Regulation Of Signal Transduction | 19 | 1 | 6.49E-04 | 0.012 | BCL2A1, PDE3B, ROBO1, CARD8, LMO3, CLIC2, IRAK3, RGS10, DMD, PIK3AP1, CYP2J2, PTPN22, EPHA7, EPHA3, PDCD4, GRB14, IL1B, MMP3, IL1RL1 |  |
| Regulation Of Metal Ion Transport | 10 | 2 | 6.68E-04 | 0.015 | DMD, GJA1, PLCB1, CXCR4, PIK3CG, PLCB4, PTPN22, CLIC2, CCL2, STOM |  |
| Programmed Cell Death | 15 | 1 | 6.81E-04 | 0.013 | PDCD4, BCL2A1, GJA1, CXCR4, PIK3CG, IL1B, CARD8, GPR65, IL24, IRAK3, CRADD, EPB41L3, G0S2, FGF13, EPHA7 |  |
| Negative Regulation Of Secretion By Cell | 7 | 2 | 6.86E-04 | 0.016 | PTPN22, IL1B, IL13RA2, CARD8, STXBP6, PDE3B, NEO1 |  |
| Regulation Of Protein Kinase Activity | 14 | 1 | 6.88E-04 | 0.013 | PDCD4, PKIA, NCKAP1L, CXCR4, PIK3CG, ROBO1, IL1B, SAMD5, IRAK3, CCND2, GMFG, NRK, FGF13, PTPN22 |  |
| Regulation Of Protein Serine/Threonine Kinase Activity | 11 | 1 | 7.02E-04 | 0.014 | PDCD4, PKIA, NRK, CXCR4, PIK3CG, ROBO1, PTPN22, IL1B, SAMD5, IRAK3, CCND2 |  |
| Signal Transduction Involved In Cellular Response To Ammonium Ion | 3 | 10 | 7.06E-04 | 0.014 | PLCB1, CHRM3, RGS10 |  |
| Acetylcholine Receptor Signaling Pathway | 3 | 10 | 7.06E-04 | 0.014 | PLCB1, CHRM3, RGS10 |  |
| Regulation Of Neurogenesis | 15 | 1 | 7.25E-04 | 0.013 | CXCR4, PLXNA2, ROBO1, SLITRK1, IL1B, FOXA1, CCND2, DMD, TCF4, FGF13, BCL11A, FLRT2, EPHA7, EPHA3, NEO1 |  |
| Negative Regulation Of Catalytic Activity | 15 | 1 | 7.47E-04 | 0.013 | PDCD4, PCDH11X, PKIA, NCKAP1L, BCL2A1, TFPI2, IL1B, CARD8, HMSD, IL24, SERPINB7, IRAK3, GMFG, PTPN22, SERPINB2 |  |
| Negative Regulation Of Cell Communication | 20 | 1 | 7.81E-04 | 0.012 | BCL2A1, PDE3B, ROBO1, CARD8, LMO3, CLIC2, IRAK3, RGS10, DMD, PIK3AP1, CYP2J2, PTPN22, EPHA7, EPHA3, PDCD4, PCDH17, GRB14, IL1B, MMP3, IL1RL1 |  |
| Negative Regulation Of Signaling | 20 | 1 | 8.06E-04 | 0.012 | BCL2A1, PDE3B, ROBO1, CARD8, LMO3, CLIC2, IRAK3, RGS10, DMD, PIK3AP1, CYP2J2, PTPN22, EPHA7, EPHA3, PDCD4, PCDH17, GRB14, IL1B, MMP3, IL1RL1 |  |
| Regulation Of Reproductive Process | 6 | 3 | 8.71E-04 | 0.016 | GRB14, ARHGDIB, NRK, PLCB1, PLAU, PRDM9 |  |
| Regulation Of Cellular Protein Metabolic Process | 32 | 1 | 8.75E-04 | 0.010 | ARRDC4, BCL2A1, CARD8, IL24, IRAK3, CCND2, DMD, PIK3AP1, PLCB1, FGF13, PTPN22, EPHA7, EPHA3, PDCD4, NCKAP1L, IL1B, NRK, KISS1, SERPINB2, PKIA, PIK3CG, ROBO1, LMO3, CCL2, HMSD, SAMSN1, CXCR4, TFPI2, SAMD5, SERPINB7, CRADD, GMFG |  |
| Regulation Of Gtpase Activity | 10 | 1 | 8.90E-04 | 0.015 | ARHGDIB, NCKAP1L, PLCB1, PLXNA2, TBC1D30, CCL2, GPR65, EPHA3, DOCK10, RGS10 |  |
| Regulation Of Branching Morphogenesis Of A Nerve | 2 | 25 | 9.28E-04 | 0.011 | BCL11A, IL1B |  |
| Positive Regulation Of Intracellular Signal Transduction | 17 | 1 | 1.00E-03 | 0.012 | PDCD4, GJA1, CXCR4, PIK3CG, ROBO1, IL1B, CCL2, GPR65, IL24, SAMD5, NRK, KISS1, PIK3AP1, PLCB1, PTPN22, EPHA7, EPHA3 |  |
| Regulation Of Endopeptidase Activity | 10 | 1 | 1.03E-03 | 0.014 | BCL2A1, TFPI2, ROBO1, CARD8, SERPINB2, HMSD, IL24, EPHA7, SERPINB7, CRADD |  |
| Regulation Of Stress-Activated Mapk Cascade | 7 | 2 | 1.07E-03 | 0.016 | PDCD4, NRK, KISS1, PLCB1, PTPN22, IL1B, SAMD5 |  |
| Regulation Of Stress-Activated Protein Kinase Signaling Cascade | 7 | 2 | 1.10E-03 | 0.016 | PDCD4, NRK, KISS1, PLCB1, PTPN22, IL1B, SAMD5 |  |
| Cell Surface Receptor Signaling Pathway | 29 | 1 | 1.11E-03 | 0.010 | BCL2A1, PLXNA2, ROBO1, CCL2, LAMA1, IL24, IRAK3, PLCB1, ADGRL3, PTPN22, S1PR3, FLRT2, EPHA7, EPHA3, PDCD4, GRB14, NCKAP1L, CXCR4, NRP2, IL1B, IL13RA2, FOXA1, CRADD, MMP3, IL1RL1, G0S2, SHC3, SERPINB2, FAM83B |  |
| B Cell Homeostasis | 3 | 8 | 1.12E-03 | 0.014 | DOCK10, NCKAP1L, BCL2A1 |  |
| Regulation Of Cellular Component Organization | 30 | 1 | 1.14E-03 | 0.010 | ARHGDIB, NAV3, PLXNA2, ROBO1, SLITRK1, CCL2, ESM1, IRAK3, CCND2, HRASLS, DMD, EPB41L3, PLCB1, ADGRL3, PRDM9, TBC1D30, FGF13, FLRT2, EPHA7, EPHA3, NCKAP1L, GJA1, CXCR4, IL1B, STXBP6, GPR65, GMFG, MMP3, BCL11A, NEO1 |  |
| Response To Nitrogen Compound | 18 | 1 | 1.15E-03 | 0.012 | GRB14, PDE3B, GJA1, CXCR4, PIK3CG, PLAU, IL1B, HMGCS1, IRAK3, RGS10, CCND2, MMP3, KCNQ3, IL1RL1, PLCB1, ADGRL3, BCL11A, PTPN22 |  |
| Positive Regulation Of Biological Process | 60 | 0 | 1.17E-03 | 0.008 | ARRDC4, BCL2A1, AADAC, NAV3, EFHC1, SLITRK1, CHRM3, CLIC2, CCND2, DMD, PIK3AP1, CYP2J2, FGF13, S1PR3, STOM, EPHA7, EPHA3, GRB14, NCKAP1L, GJA1, IL1B, IL13RA2, FOXA1, MMP3, NRK, KISS1, ZNF438, NEO1, PIK3CG, PLAU, PLXNA2, PLTP, ROBO1, CCL2, HMSD, TBXAS1, TCF4, SRPX, CXCR4, NRP2, GPR65, LGR5, SAMD5, SERPINB7, CRADD, IL1RL1, CARD8, IL24, ESM1, IRAK3, PLCB1, ADGRL3, PTPN22, FLRT2, ABI3BP, PDCD4, G0S2, BCL11A, LMO3, PRDM9 |  |
| Lipopolysaccharide-Mediated Signaling Pathway | 3 | 8 | 1.21E-03 | 0.014 | PTPN22, IL1B, CCL2 |  |
| Cerebral Cortex Cell Migration | 3 | 8 | 1.21E-03 | 0.014 | EFHC1, FGF13, NRP2 |  |
| Signal Transduction | 58 | 0 | 1.27E-03 | 0.008 | ARHGDIB, BCL2A1, CD33, CHRM3, CLIC2, DMD, OR2M3, PIK3AP1, FGF13, S1PR3, EPHA7, EPHA3, GRB14, NCKAP1L, GJA1, GNGT2, IL1B, IL13RA2, FOXA1, MMP3, NRK, KISS1, SERPINB2, OR5B21, NEO1, FAM83B, PDE3B, PIK3CG, PLAU, PLCB4, PLXNA2, ROBO1, CCL2, LAMA1, RGS10, CXCR4, NRP2, GPR65, LGR5, CRADD, IL1RL1, RAPGEF5, CNKSR2, IL24, IRAK3, PLCB1, ADGRL3, PTPN22, PITPNC1, FLRT2, PDCD4, OR2H1, G0S2, SHC3, MCTP2, DOCK10, HRASLS, GPR135 |  |
| Negative Regulation Of Transport | 11 | 1 | 1.31E-03 | 0.014 | PKIA, PDE3B, PLCB4, PTPN22, IL1B, IL13RA2, CARD8, CLIC2, STXBP6, EPHA3, NEO1 |  |
| Positive Regulation Of Protein Phosphorylation | 16 | 1 | 1.35E-03 | 0.012 | NCKAP1L, CXCR4, PIK3CG, ROBO1, IL1B, CCL2, IL24, SAMD5, CCND2, NRK, KISS1, PLCB1, FGF13, PTPN22, EPHA7, EPHA3 |  |
| Positive Regulation Of Hydrolase Activity | 13 | 1 | 1.36E-03 | 0.013 | ARHGDIB, NCKAP1L, ROBO1, CARD8, TOR1AIP2, CCL2, GPR65, IL24, DOCK10, RGS10, CRADD, PLCB1, TBC1D30 |  |
| Positive Regulation Of Transferase Activity | 12 | 1 | 1.40E-03 | 0.013 | ARRDC4, NCKAP1L, CXCR4, PIK3CG, ROBO1, IL1B, SAMD5, CCND2, NRK, FGF13, EPHA7, EPHA3 |  |
| Negative Regulation Of Secretion | 7 | 2 | 1.41E-03 | 0.015 | PDE3B, PTPN22, IL1B, IL13RA2, CARD8, STXBP6, NEO1 |  |
| Response To Organonitrogen Compound | 17 | 1 | 1.42E-03 | 0.012 | GRB14, PDE3B, GJA1, CXCR4, PIK3CG, PLAU, IL1B, HMGCS1, IRAK3, RGS10, CCND2, MMP3, IL1RL1, PLCB1, ADGRL3, BCL11A, PTPN22 |  |
| Positive Regulation Of Cd8-Positive, Alpha-Beta T Cell Activation | 2 | 20 | 1.48E-03 | 0.010 | PTPN22, NCKAP1L |  |
| Negative Regulation Of Response To External Stimulus | 8 | 2 | 1.56E-03 | 0.015 | PDCD4, GJA1, PLAU, ROBO1, CARD8, SERPINB2, CCL2, NEO1 |  |
| Neuron Projection Morphogenesis | 7 | 2 | 1.65E-03 | 0.015 | NRK, NCKAP1L, EPB41L3, GJA1, PRDM8, SLITRK1, DOCK10 |  |
| Negative Regulation Of Cellular Protein Metabolic Process | 17 | 1 | 1.68E-03 | 0.012 | PDCD4, PKIA, NCKAP1L, BCL2A1, TFPI2, IL1B, CARD8, LMO3, HMSD, IL24, SERPINB7, IRAK3, GMFG, DMD, PTPN22, SAMSN1, SERPINB2 |  |
| Regulation Of Jnk Cascade | 6 | 2 | 1.69E-03 | 0.015 | PDCD4, PTPN22, IL1B, NRK, SAMD5, PLCB1 |  |
| Negative Regulation Of Mapk Cascade | 6 | 2 | 1.73E-03 | 0.015 | PDCD4, PTPN22, IL1B, DMD, LMO3, IRAK3 |  |
| Regulation Of Protein Metabolic Process | 33 | 0 | 1.74E-03 | 0.009 | ARRDC4, BCL2A1, CARD8, IL24, IRAK3, CCND2, DMD, PIK3AP1, PLCB1, FGF13, PTPN22, EPHA7, EPHA3, PDCD4, NCKAP1L, GJA1, IL1B, NRK, KISS1, SERPINB2, PKIA, PIK3CG, ROBO1, LMO3, CCL2, HMSD, SAMSN1, CXCR4, TFPI2, SAMD5, SERPINB7, CRADD, GMFG |  |
| Positive Regulation Of Kinase Activity | 11 | 1 | 1.77E-03 | 0.013 | NCKAP1L, CXCR4, PIK3CG, ROBO1, IL1B, SAMD5, CCND2, NRK, FGF13, EPHA7, EPHA3 |  |
| Positive Regulation Of Cell Death | 13 | 1 | 1.80E-03 | 0.013 | PDCD4, SRPX, EFHC1, ROBO1, IL1B, CARD8, CCL2, IL24, FOXA1, CRADD, MMP3, G0S2, EPHA7 |  |
| Heat Generation | 2 | 18 | 1.80E-03 | 0.010 | IL1B, EPHA3 |  |
| Ether Lipid Metabolic Process | 2 | 18 | 1.80E-03 | 0.010 | HRASLS, AGMO |  |
| Regulation Of Multi-Organism Process | 9 | 1 | 1.84E-03 | 0.014 | GRB14, ARHGDIB, PLCB1, CXCR4, PLAU, PTPN22, IL1B, CARD8, STOM |  |
| Plasma Membrane Bounded Cell Projection Morphogenesis | 7 | 2 | 1.89E-03 | 0.015 | NRK, NCKAP1L, EPB41L3, GJA1, PRDM8, SLITRK1, DOCK10 |  |
| Neuron Recognition | 3 | 7 | 1.90E-03 | 0.014 | ROBO1, EPHA3, CXCR4 |  |
| Regulation Of Axonogenesis | 6 | 2 | 1.94E-03 | 0.015 | ROBO1, FGF13, BCL11A, SLITRK1, EPHA7, PLXNA2 |  |
| Negative Regulation Of Cytokine Production | 7 | 2 | 2.00E-03 | 0.015 | PDCD4, NCKAP1L, IL1RL1, NAV3, PTPN22, CARD8, IRAK3 |  |
| Cell Projection Morphogenesis | 7 | 2 | 2.04E-03 | 0.015 | NRK, NCKAP1L, EPB41L3, GJA1, PRDM8, SLITRK1, DOCK10 |  |
| Homeostatic Process | 21 | 1 | 2.04E-03 | 0.011 | BCL2A1, PDE3B, PIK3CG, PLAU, EFHC1, SLITRK1, CCL2, DOCK10, DMD, TBXAS1, S1PR3, EPHA3, HIST2H4B, NCKAP1L, GJA1, CXCR4, IL1B, GPR65, FOXA1, KISS1, NEO1 |  |
| Response To Lipopolysaccharide | 10 | 1 | 2.08E-03 | 0.013 | PDCD4, MMP3, GJA1, PLAU, PTPN22, IL1B, CCL2, IL24, EPHA3, IRAK3 |  |
| Negative Regulation Of Map Kinase Activity | 4 | 4 | 2.10E-03 | 0.015 | PDCD4, PTPN22, IL1B, IRAK3 |  |
| Cytokine-Mediated Signaling Pathway | 11 | 1 | 2.11E-03 | 0.013 | PDCD4, CXCR4, IL1B, IL13RA2, CCL2, IL24, IRAK3, MMP3, IL1RL1, PLCB1, SERPINB2 |  |
| Negative Regulation Of Protein Transport | 6 | 2 | 2.12E-03 | 0.015 | PTPN22, IL1B, PKIA, CARD8, PDE3B, NEO1 |  |
| Negative Regulation Of Phosphate Metabolic Process | 11 | 1 | 2.13E-03 | 0.013 | PDCD4, PCDH11X, PKIA, NCKAP1L, IL1B, LMO3, IRAK3, GMFG, DMD, PTPN22, SAMSN1 |  |
| Wound Healing | 5 | 3 | 2.14E-03 | 0.015 | IL1B, MMP3, SERPINB2, IL24, PLAU |  |
| Response To Ammonium Ion | 5 | 3 | 2.14E-03 | 0.015 | IL1B, KCNQ3, CXCR4, PLAU, ADGRL3 |  |
| Regulation Of Secretion By Cell | 13 | 1 | 2.15E-03 | 0.012 | PDE3B, GJA1, MCTP2, IL1B, IL13RA2, CARD8, STXBP6, SYTL3, IL1RL1, KISS1, CYP2J2, PTPN22, NEO1 |  |
| Negative Regulation Of Phosphorus Metabolic Process | 11 | 1 | 2.16E-03 | 0.013 | PDCD4, PCDH11X, PKIA, NCKAP1L, IL1B, LMO3, IRAK3, GMFG, DMD, PTPN22, SAMSN1 |  |
| Positive Regulation Of Phosphorylation | 16 | 1 | 2.17E-03 | 0.011 | NCKAP1L, CXCR4, PIK3CG, ROBO1, IL1B, CCL2, IL24, SAMD5, CCND2, NRK, KISS1, PLCB1, FGF13, PTPN22, EPHA7, EPHA3 |  |
| Positive Regulation Of Cytokine Secretion | 5 | 3 | 2.20E-03 | 0.015 | PTPN22, IL1B, CARD8, IL1RL1, CYP2J2 |  |
| Positive Regulation Of Neuron Differentiation | 9 | 1 | 2.21E-03 | 0.014 | DMD, TCF4, CXCR4, PLXNA2, ROBO1, BCL11A, SLITRK1, FOXA1, EPHA3 |  |
| Regulation Of Peptidase Activity | 10 | 1 | 2.27E-03 | 0.013 | BCL2A1, TFPI2, ROBO1, CARD8, SERPINB2, HMSD, IL24, EPHA7, SERPINB7, CRADD |  |
| Negative Regulation Of Cell Projection Organization | 6 | 2 | 2.31E-03 | 0.015 | FGF13, BCL11A, EPHA7, EPHA3, TBC1D30, NEO1 |  |
| Negative Regulation Of Establishment Of Protein Localization | 6 | 2 | 2.31E-03 | 0.015 | PTPN22, IL1B, PKIA, CARD8, PDE3B, NEO1 |  |
| Regulation Of Cytokine Production | 12 | 1 | 2.33E-03 | 0.013 | PDCD4, NCKAP1L, NAV3, IL1B, CARD8, SERPINB7, IRAK3, IL1RL1, PLCB1, CYP2J2, PTPN22, S1PR3 |  |
| Negative Regulation Of Protein Serine/Threonine Kinase Activity | 5 | 3 | 2.39E-03 | 0.015 | PDCD4, PTPN22, IL1B, PKIA, IRAK3 |  |
| Positive Regulation Of Apoptotic Process | 12 | 1 | 2.43E-03 | 0.012 | PDCD4, SRPX, EFHC1, ROBO1, IL1B, CARD8, CCL2, IL24, FOXA1, CRADD, G0S2, EPHA7 |  |
| Negative Regulation Of Protein Phosphorylation | 9 | 1 | 2.44E-03 | 0.014 | PDCD4, GMFG, PKIA, DMD, PTPN22, IL1B, SAMSN1, LMO3, IRAK3 |  |
| Negative Regulation Of Protein Secretion | 5 | 3 | 2.46E-03 | 0.015 | PTPN22, IL1B, CARD8, PDE3B, NEO1 |  |
| Myelin Maintenance | 2 | 15 | 2.54E-03 | 0.010 | EPB41L3, CXCR4 |  |
| Semaphorin-Plexin Signaling Pathway Involved In Neuron Projection Guidance | 2 | 15 | 2.54E-03 | 0.010 | NRP2, PLXNA2 |  |
| Negative Regulation Of Response To Wounding | 4 | 4 | 2.54E-03 | 0.014 | SERPINB2, GJA1, PLAU, NEO1 |  |
| Cellular Response To Oxygen-Containing Compound | 17 | 1 | 2.61E-03 | 0.011 | PDCD4, GRB14, PDE3B, PIK3CG, PLAU, IL1B, CCL2, IL24, HMGCS1, CCND2, MMP3, IL1RL1, PLCB1, BCL11A, PTPN22, GDAP1, EPHA3 |  |
| Positive Regulation Of Programmed Cell Death | 12 | 1 | 2.61E-03 | 0.012 | PDCD4, SRPX, EFHC1, ROBO1, IL1B, CARD8, CCL2, IL24, FOXA1, CRADD, G0S2, EPHA7 |  |
| Interleukin-12-Mediated Signaling Pathway | 3 | 6 | 2.62E-03 | 0.013 | PDCD4, PLCB1, SERPINB2 |  |
| Response To Molecule Of Bacterial Origin | 10 | 1 | 2.75E-03 | 0.013 | PDCD4, MMP3, GJA1, PLAU, PTPN22, IL1B, CCL2, IL24, EPHA3, IRAK3 |  |
| Regulation Of Synapse Assembly | 4 | 3 | 2.84E-03 | 0.014 | SLITRK1, FLRT2, EPHA7, ADGRL3 |  |
| Positive Regulation Of Neurogenesis | 10 | 1 | 2.89E-03 | 0.013 | DMD, TCF4, CXCR4, PLXNA2, ROBO1, BCL11A, SLITRK1, IL1B, FOXA1, EPHA3 |  |
| Regulation Of Ion Transport | 12 | 1 | 2.89E-03 | 0.012 | GJA1, CXCR4, PIK3CG, PLCB4, IL1B, CLIC2, CCL2, DMD, KCNQ3, PLCB1, PTPN22, STOM |  |
| Negative Regulation Of Protein Metabolic Process | 17 | 1 | 2.92E-03 | 0.011 | PDCD4, PKIA, NCKAP1L, BCL2A1, TFPI2, IL1B, CARD8, LMO3, HMSD, IL24, SERPINB7, IRAK3, GMFG, DMD, PTPN22, SAMSN1, SERPINB2 |  |
| Negative Regulation Of Cell Development | 8 | 1 | 2.93E-03 | 0.014 | GRB14, FGF13, BCL11A, IL1B, S1PR3, EPHA7, EPHA3, NEO1 |  |
| Neutrophil Chemotaxis | 4 | 3 | 2.94E-03 | 0.014 | IL1B, NCKAP1L, CCL2, PIK3CG |  |
| Regulation Of Triglyceride Catabolic Process | 2 | 14 | 2.95E-03 | 0.010 | AADAC, PIK3CG |  |
| Regulation Of Endothelial Cell Development | 2 | 14 | 2.95E-03 | 0.010 | S1PR3, IL1B |  |
| Regulation Of Establishment Of Endothelial Barrier | 2 | 14 | 2.95E-03 | 0.010 | S1PR3, IL1B |  |
| Negative Regulation Of Interleukin-6 Production | 3 | 6 | 2.96E-03 | 0.013 | PTPN22, IRAK3, NCKAP1L |  |
| Negative Regulation Of Endopeptidase Activity | 7 | 2 | 3.01E-03 | 0.014 | BCL2A1, TFPI2, CARD8, SERPINB2, HMSD, IL24, SERPINB7 |  |
| Regulation Of Calcium Ion Transport | 7 | 2 | 3.06E-03 | 0.014 | DMD, GJA1, CXCR4, PIK3CG, PTPN22, CLIC2, CCL2 |  |
| Negative Regulation Of Peptide Secretion | 5 | 2 | 3.19E-03 | 0.014 | PTPN22, IL1B, CARD8, PDE3B, NEO1 |  |
| Organophosphate Ester Transport | 4 | 3 | 3.27E-03 | 0.014 | ATP8B2, PITPNC1, GJA1, PLTP |  |
| Extrinsic Apoptotic Signaling Pathway | 4 | 3 | 3.27E-03 | 0.014 | IL1B, BCL2A1, G0S2, CRADD |  |
| Response To Fluid Shear Stress | 3 | 5 | 3.31E-03 | 0.013 | GJA1, PLAU, TFPI2 |  |
| Glycerol Ether Metabolic Process | 2 | 13 | 3.39E-03 | 0.010 | HRASLS, AGMO |  |
| Negative Regulation Of Cell Maturation | 2 | 13 | 3.39E-03 | 0.010 | BCL11A, GRB14 |  |
| Cranial Nerve Structural Organization | 2 | 13 | 3.39E-03 | 0.010 | NRP2, DMD |  |
| Leukocyte Chemotaxis | 5 | 2 | 3.44E-03 | 0.014 | IL1B, NCKAP1L, CCL2, CXCR4, PIK3CG |  |
| Anatomical Structure Development | 41 | 0 | 3.46E-03 | 0.009 | ARHGDIB, BCL2A1, NAV3, SLITRK1, CHRM3, CCND2, DMD, PLCB1, ADGRL3, FGF13, FLRT2, EPHA7, EPHA3, NCKAP1L, GJA1, HMGCS1, FOXA1, SHISA3, KCNQ3, KISS1, LAMA4, BCL11A, SHC3, ZNF438, NEO1, PDE3B, PLAU, PLXNA2, ROBO1, MCTP2, PCDHB6, LAMA1, TCF4, PCDHB2, PRDM8, CXCR4, NRP2, GPR65, LGR5, CLMP, RAPGEF5 |  |
| Negative Regulation Of Developmental Process | 16 | 1 | 3.50E-03 | 0.011 | PDCD4, GRB14, ARHGDIB, HIST2H4B, PDE3B, GJA1, ROBO1, IL1B, FOXA1, TCF4, FGF13, BCL11A, S1PR3, EPHA7, EPHA3, NEO1 |  |
| Positive Regulation Of Axonogenesis | 4 | 3 | 3.50E-03 | 0.014 | ROBO1, BCL11A, SLITRK1, PLXNA2 |  |
| Response To Mechanical Stimulus | 7 | 2 | 3.56E-03 | 0.014 | DMD, MMP3, GJA1, CXCR4, IL1B, IL13RA2, CRADD |  |
| Cell Part Morphogenesis | 7 | 2 | 3.56E-03 | 0.014 | NRK, NCKAP1L, EPB41L3, GJA1, PRDM8, SLITRK1, DOCK10 |  |
| Cellular Calcium Ion Homeostasis | 9 | 1 | 3.56E-03 | 0.013 | KISS1, GJA1, CXCR4, PIK3CG, EFHC1, IL1B, CCL2, S1PR3, GPR65 |  |
| Interleukin-1-Mediated Signaling Pathway | 3 | 5 | 3.70E-03 | 0.013 | IL1B, PLCB1, IRAK3 |  |
| Positive Regulation Of Protein Modification Process | 17 | 1 | 3.74E-03 | 0.011 | ARRDC4, PIK3CG, ROBO1, CCL2, IL24, CCND2, PLCB1, FGF13, PTPN22, EPHA7, EPHA3, NCKAP1L, CXCR4, IL1B, SAMD5, NRK, KISS1 |  |
| Regulation Of Neuron Projection Development | 10 | 1 | 3.76E-03 | 0.013 | DMD, CXCR4, PLXNA2, ROBO1, FGF13, BCL11A, SLITRK1, EPHA7, EPHA3, NEO1 |  |
| Regulation Of Peroxisome Proliferator Activated Receptor Signaling Pathway | 2 | 12 | 3.86E-03 | 0.010 | LMO3, CYP2J2 |  |
| Positive Regulation Of Sodium Ion Transmembrane Transporter Activity | 2 | 12 | 3.86E-03 | 0.010 | DMD, PLCB1 |  |
| Granulocyte Chemotaxis | 4 | 3 | 3.87E-03 | 0.014 | IL1B, NCKAP1L, CCL2, PIK3CG |  |
| Negative Regulation Of Immune System Process | 9 | 1 | 3.88E-03 | 0.013 | HIST2H4B, IL1RL1, PIK3AP1, PLCB1, PTPN22, IL13RA2, SAMSN1, CCL2, IRAK3 |  |
| Positive Regulation Of Gtpase Activity | 8 | 1 | 3.90E-03 | 0.013 | ARHGDIB, NCKAP1L, PLCB1, TBC1D30, CCL2, GPR65, DOCK10, RGS10 |  |
| Regulation Of Plasma Membrane Bounded Cell Projection Organization | 12 | 1 | 3.94E-03 | 0.012 | CXCR4, NAV3, PLXNA2, ROBO1, SLITRK1, DMD, TBC1D30, FGF13, BCL11A, EPHA7, EPHA3, NEO1 |  |
| Cellular Response To Nitrogen Compound | 11 | 1 | 4.02E-03 | 0.012 | GRB14, PDE3B, PIK3CG, PLAU, CCND2, MMP3, KCNQ3, IL1RL1, PLCB1, BCL11A, PTPN22 |  |
| Positive Regulation Of Cellular Component Organization | 17 | 1 | 4.09E-03 | 0.011 | NAV3, PLXNA2, ROBO1, SLITRK1, CCL2, DMD, PLCB1, ADGRL3, PRDM9, FLRT2, EPHA3, NCKAP1L, CXCR4, IL1B, GPR65, MMP3, BCL11A |  |
| Positive Regulation Of Phosphorus Metabolic Process | 16 | 1 | 4.12E-03 | 0.011 | NCKAP1L, CXCR4, PIK3CG, ROBO1, IL1B, CCL2, IL24, SAMD5, CCND2, NRK, KISS1, PLCB1, FGF13, PTPN22, EPHA7, EPHA3 |  |
| Positive Regulation Of Phosphate Metabolic Process | 16 | 1 | 4.12E-03 | 0.011 | NCKAP1L, CXCR4, PIK3CG, ROBO1, IL1B, CCL2, IL24, SAMD5, CCND2, NRK, KISS1, PLCB1, FGF13, PTPN22, EPHA7, EPHA3 |  |
| Transmembrane Receptor Protein Tyrosine Kinase Signaling Pathway | 9 | 1 | 4.13E-03 | 0.013 | GRB14, NCKAP1L, PLCB1, NRP2, SHC3, FLRT2, EPHA7, EPHA3, FAM83B |  |
| Neutrophil Migration | 4 | 3 | 4.13E-03 | 0.014 | IL1B, NCKAP1L, CCL2, PIK3CG |  |
| Negative Regulation Of Protein Kinase Activity | 6 | 2 | 4.24E-03 | 0.014 | PDCD4, GMFG, PKIA, PTPN22, IL1B, IRAK3 |  |
| Positive Regulation Of Peptide Secretion | 7 | 2 | 4.24E-03 | 0.013 | IL1RL1, KISS1, GJA1, CYP2J2, PTPN22, IL1B, CARD8 |  |
| Regulation Of Sodium Ion Transmembrane Transporter Activity | 3 | 5 | 4.32E-03 | 0.013 | STOM, DMD, PLCB1 |  |
| Regulation Of Cell Projection Organization | 12 | 1 | 4.32E-03 | 0.012 | CXCR4, NAV3, PLXNA2, ROBO1, SLITRK1, DMD, TBC1D30, FGF13, BCL11A, EPHA7, EPHA3, NEO1 |  |
| Regulation Of Cd8-Positive, Alpha-Beta T Cell Activation | 2 | 11 | 4.35E-03 | 0.010 | PTPN22, NCKAP1L |  |
| Regulation Of Blood Circulation | 7 | 2 | 4.45E-03 | 0.013 | DMD, TBXAS1, GJA1, PIK3CG, CYP2J2, CHRM3, CLIC2 |  |
| Calcium Ion Homeostasis | 9 | 1 | 4.49E-03 | 0.013 | KISS1, GJA1, CXCR4, PIK3CG, EFHC1, IL1B, CCL2, S1PR3, GPR65 |  |
| Negative Regulation Of Phosphorylation | 9 | 1 | 4.49E-03 | 0.013 | PDCD4, GMFG, PKIA, DMD, PTPN22, IL1B, SAMSN1, LMO3, IRAK3 |  |
| Regulation Of Cysteine-Type Endopeptidase Activity Involved In Apoptotic Process | 6 | 2 | 4.65E-03 | 0.014 | BCL2A1, ROBO1, CARD8, IL24, EPHA7, CRADD |  |
| Positive Regulation Of Cytosolic Calcium Ion Concentration | 7 | 1 | 4.66E-03 | 0.013 | KISS1, GJA1, CXCR4, PIK3CG, IL1B, S1PR3, GPR65 |  |
| Cellular Response To Endogenous Stimulus | 14 | 1 | 4.67E-03 | 0.011 | GRB14, PDE3B, GJA1, PIK3CG, PLAU, CCL2, HMGCS1, CCND2, MMP3, IL1RL1, PLCB1, BCL11A, PTPN22, EPHA3 |  |
| Chronic Inflammatory Response | 2 | 11 | 4.88E-03 | 0.010 | IL1B, GJA1 |  |
| Negative Regulation Of Jun Kinase Activity | 2 | 11 | 4.88E-03 | 0.010 | PDCD4, PTPN22 |  |
| Cellular Extravasation | 2 | 11 | 4.88E-03 | 0.010 | PIK3CG, CCL2 |  |
| Regulation Of Cell Differentiation | 23 | 1 | 4.88E-03 | 0.010 | PLXNA2, ROBO1, SLITRK1, LMO3, CCND2, DMD, TCF4, PLCB1, FGF13, S1PR3, FLRT2, EPHA7, EPHA3, PDCD4, GRB14, HIST2H4B, NCKAP1L, GJA1, CXCR4, IL1B, FOXA1, BCL11A, NEO1 |  |
| Negative Regulation Of Neuron Projection Development | 5 | 2 | 5.01E-03 | 0.014 | FGF13, BCL11A, EPHA7, EPHA3, NEO1 |  |
| Regulation Of Secretion | 13 | 1 | 5.17E-03 | 0.011 | PDE3B, GJA1, MCTP2, IL1B, IL13RA2, CARD8, STXBP6, SYTL3, IL1RL1, KISS1, CYP2J2, PTPN22, NEO1 |  |
| Negative Regulation Of Cell Migration | 7 | 1 | 5.18E-03 | 0.013 | ARHGDIB, KISS1, PLCB1, NAV3, ROBO1, CCL2, IL24 |  |
| Positive Regulation Of Stress-Activated Mapk Cascade | 5 | 2 | 5.24E-03 | 0.014 | IL1B, NRK, KISS1, SAMD5, PLCB1 |  |
| Positive Regulation Of Stress-Activated Protein Kinase Signaling Cascade | 5 | 2 | 5.36E-03 | 0.014 | IL1B, NRK, KISS1, SAMD5, PLCB1 |  |
| Epithelial Cell Maturation | 2 | 10 | 5.43E-03 | 0.010 | FOXA1, GJA1 |  |
| Dendritic Cell Chemotaxis | 2 | 10 | 5.43E-03 | 0.010 | CXCR4, PIK3CG |  |
| Positive Regulation Of Meiotic Nuclear Division | 2 | 10 | 5.43E-03 | 0.010 | PLCB1, PRDM9 |  |
| Granulocyte Migration | 4 | 3 | 5.44E-03 | 0.013 | IL1B, NCKAP1L, CCL2, PIK3CG |  |
| Regulation Of Nik/Nf-Kappab Signaling | 4 | 3 | 5.44E-03 | 0.013 | PDCD4, PTPN22, IL1B, SAMD5 |  |
| Negative Regulation Of Cytokine-Mediated Signaling Pathway | 3 | 4 | 5.49E-03 | 0.012 | ROBO1, CARD8, IRAK3 |  |
| Cellular Divalent Inorganic Cation Homeostasis | 9 | 1 | 5.54E-03 | 0.012 | KISS1, GJA1, CXCR4, PIK3CG, EFHC1, IL1B, CCL2, S1PR3, GPR65 |  |
| Regulation Of Meiotic Cell Cycle | 3 | 4 | 5.74E-03 | 0.012 | GRB14, PLCB1, PRDM9 |  |
| Regulation Of Cell Death | 22 | 1 | 6.01E-03 | 0.010 | BCL2A1, PIK3CG, EFHC1, ROBO1, CARD8, CCL2, IL24, IRAK3, CCND2, EPHA7, EPHA3, PDCD4, SRPX, NCKAP1L, CXCR4, IL1B, FOXA1, CRADD, MMP3, G0S2, SERPINB2, NEO1 |  |
| Ether Metabolic Process | 2 | 10 | 6.01E-03 | 0.010 | HRASLS, AGMO |  |
| Ganglion Development | 2 | 10 | 6.01E-03 | 0.010 | KCNQ3, NRP2 |  |
| Negative Regulation Of Peptidase Activity | 7 | 1 | 6.08E-03 | 0.013 | BCL2A1, TFPI2, CARD8, SERPINB2, HMSD, IL24, SERPINB7 |  |
| Negative Regulation Of Neuron Differentiation | 6 | 2 | 6.15E-03 | 0.013 | FGF13, BCL11A, IL1B, EPHA7, EPHA3, NEO1 |  |
| Negative Regulation Of Molecular Function | 16 | 1 | 6.21E-03 | 0.010 | PDCD4, PCDH11X, PKIA, NCKAP1L, BCL2A1, TFPI2, IL1B, CARD8, CLIC2, HMSD, IL24, SERPINB7, IRAK3, GMFG, PTPN22, SERPINB2 |  |
| Regulation Of Response To Wounding | 5 | 2 | 6.35E-03 | 0.013 | SERPINB2, GJA1, CXCR4, PLAU, NEO1 |  |
| Negative Regulation Of Kinase Activity | 6 | 2 | 6.37E-03 | 0.013 | PDCD4, GMFG, PKIA, PTPN22, IL1B, IRAK3 |  |
| Negative Regulation Of Response To Cytokine Stimulus | 3 | 4 | 6.55E-03 | 0.012 | ROBO1, CARD8, IRAK3 |  |
| Positive Regulation Of Defense Response | 8 | 1 | 6.75E-03 | 0.012 | PDCD4, IL1RL1, PIK3AP1, GJA1, PIK3CG, PTPN22, IL1B, IRAK3 |  |
| Chemical Synaptic Transmission | 7 | 1 | 6.81E-03 | 0.013 | PCDHB2, KCNQ3, SHC3, PCDHB5, CHRM3, PCDHB6, PCDHB16 |  |
| Anterograde Trans-Synaptic Signaling | 7 | 1 | 6.81E-03 | 0.013 | PCDHB2, KCNQ3, SHC3, PCDHB5, CHRM3, PCDHB6, PCDHB16 |  |
| Negative Regulation Of Cell Motility | 7 | 1 | 6.81E-03 | 0.013 | ARHGDIB, KISS1, PLCB1, NAV3, ROBO1, CCL2, IL24 |  |
| Regulation Of Cell Morphogenesis | 9 | 1 | 7.00E-03 | 0.012 | EPB41L3, CXCR4, PLXNA2, ROBO1, FGF13, BCL11A, SLITRK1, CCL2, EPHA7 |  |
| Regulation Of Cysteine-Type Endopeptidase Activity | 6 | 2 | 7.03E-03 | 0.013 | BCL2A1, ROBO1, CARD8, IL24, EPHA7, CRADD |  |
| Positive Regulation Of Neuron Projection Development | 7 | 1 | 7.10E-03 | 0.013 | DMD, CXCR4, PLXNA2, ROBO1, BCL11A, SLITRK1, EPHA3 |  |
| Lymphocyte Homeostasis | 3 | 4 | 7.12E-03 | 0.012 | NCKAP1L, BCL2A1, DOCK10 |  |
| Regulation Of Sodium Ion Transmembrane Transport | 3 | 4 | 7.12E-03 | 0.012 | DMD, STOM, PLCB1 |  |
| Cellular Response To Organonitrogen Compound | 10 | 1 | 7.20E-03 | 0.012 | GRB14, PDE3B, PIK3CG, PLAU, CCND2, MMP3, IL1RL1, PLCB1, BCL11A, PTPN22 |  |
| Positive Regulation Of Sodium Ion Transmembrane Transport | 2 | 9 | 7.26E-03 | 0.010 | DMD, PLCB1 |  |
| Positive Regulation Of Cell Development | 10 | 1 | 7.56E-03 | 0.012 | CXCR4, PLXNA2, ROBO1, SLITRK1, IL1B, FOXA1, DMD, TCF4, BCL11A, EPHA3 |  |
| Divalent Inorganic Cation Homeostasis | 9 | 1 | 7.63E-03 | 0.012 | KISS1, GJA1, CXCR4, PIK3CG, EFHC1, IL1B, CCL2, S1PR3, GPR65 |  |
| Positive Regulation Of Protein Kinase Activity | 9 | 1 | 7.63E-03 | 0.012 | NRK, NCKAP1L, CXCR4, PIK3CG, ROBO1, FGF13, IL1B, SAMD5, CCND2 |  |
| Regulation Of Peptide Secretion | 9 | 1 | 7.63E-03 | 0.012 | PDE3B, IL1RL1, KISS1, GJA1, CYP2J2, PTPN22, IL1B, CARD8, NEO1 |  |
| Negative Regulation Of Protein Modification Process | 10 | 1 | 7.78E-03 | 0.012 | PDCD4, PKIA, NCKAP1L, IL1B, LMO3, IRAK3, GMFG, DMD, PTPN22, SAMSN1 |  |
| Synaptic Signaling | 7 | 1 | 7.81E-03 | 0.012 | PCDHB2, KCNQ3, SHC3, PCDHB5, CHRM3, PCDHB6, PCDHB16 |  |
| Trans-Synaptic Signaling | 7 | 1 | 7.81E-03 | 0.012 | PCDHB2, KCNQ3, SHC3, PCDHB5, CHRM3, PCDHB6, PCDHB16 |  |
| Fibrinolysis | 2 | 8 | 7.92E-03 | 0.010 | PLAU, SERPINB2 |  |
| Dendritic Cell Migration | 2 | 8 | 7.92E-03 | 0.010 | CXCR4, PIK3CG |  |
| Negative Regulation Of Cellular Metabolic Process | 29 | 0 | 7.95E-03 | 0.009 | BCL2A1, CARD8, IL24, IRAK3, DMD, PLCB1, PTPN22, FAM172BP, PDCD4, PCDH11X, NCKAP1L, GJA1, IL1B, FOXA1, MMP3, BCL11A, SERPINB2, ZNF438, PKIA, PIK3CG, LMO3, HMSD, TCF4, PRDM8, SAMSN1, HIST2H4B, TFPI2, SERPINB7, GMFG |  |
| Regulation Of Erk1 And Erk2 Cascade | 7 | 1 | 8.02E-03 | 0.012 | DMD, PTPN22, IL1B, LMO3, CCL2, EPHA7, EPHA3 |  |
| Glial Cell Development | 3 | 4 | 8.04E-03 | 0.012 | DMD, KCNQ3, PRDM8 |  |
| Response To Lipid | 16 | 1 | 8.05E-03 | 0.010 | PDCD4, GJA1, PLAU, IL1B, CCL2, IL24, HMGCS1, FOXA1, IRAK3, CCND2, MMP3, TBXAS1, PLCB1, PTPN22, GDAP1, EPHA3 |  |
| Anatomical Structure Arrangement | 2 | 8 | 8.60E-03 | 0.010 | NRP2, DMD |  |
| Positive Regulation Of Cell Projection Organization | 8 | 1 | 8.65E-03 | 0.012 | DMD, CXCR4, NAV3, PLXNA2, ROBO1, BCL11A, SLITRK1, EPHA3 |  |
| Positive Regulation Of Map Kinase Activity | 6 | 1 | 8.77E-03 | 0.013 | NRK, CXCR4, PIK3CG, ROBO1, IL1B, SAMD5 |  |
| Regulation Of Cytosolic Calcium Ion Concentration | 7 | 1 | 8.80E-03 | 0.012 | KISS1, GJA1, CXCR4, PIK3CG, IL1B, S1PR3, GPR65 |  |
| Negative Regulation Of Hydrolase Activity | 9 | 1 | 8.82E-03 | 0.012 | PCDH11X, NCKAP1L, BCL2A1, TFPI2, CARD8, SERPINB2, HMSD, IL24, SERPINB7 |  |
| Positive Regulation Of Protein Serine/Threonine Kinase Activity | 7 | 1 | 8.92E-03 | 0.012 | NRK, CXCR4, PIK3CG, ROBO1, IL1B, SAMD5, CCND2 |  |
| Phospholipid Transport | 3 | 4 | 9.02E-03 | 0.012 | ATP8B2, PITPNC1, PLTP |  |
| Positive Regulation Of Phagocytosis | 3 | 4 | 9.02E-03 | 0.012 | IL1B, NCKAP1L, CCL2 |  |
| Regulation Of Leukocyte Migration | 5 | 2 | 9.05E-03 | 0.013 | PTPN22, IL1B, NCKAP1L, CCL2, PLCB1 |  |
| Positive Regulation Of Protein Metabolic Process | 20 | 1 | 9.06E-03 | 0.010 | ARRDC4, PIK3CG, ROBO1, CARD8, CCL2, IL24, CCND2, PLCB1, FGF13, PTPN22, EPHA7, EPHA3, NCKAP1L, GJA1, CXCR4, IL1B, SAMD5, CRADD, NRK, KISS1 |  |
| Positive Regulation Of Cellular Process | 51 | 0 | 9.18E-03 | 0.008 | ARRDC4, AADAC, NAV3, EFHC1, SLITRK1, CLIC2, CCND2, DMD, PIK3AP1, CYP2J2, FGF13, S1PR3, EPHA7, EPHA3, GRB14, NCKAP1L, GJA1, IL1B, FOXA1, MMP3, NRK, KISS1, ZNF438, NEO1, PIK3CG, PLAU, PLXNA2, ROBO1, CCL2, TCF4, SRPX, CXCR4, NRP2, GPR65, LGR5, SAMD5, SERPINB7, CRADD, IL1RL1, CARD8, IL24, ESM1, PLCB1, ADGRL3, PTPN22, FLRT2, PDCD4, G0S2, BCL11A, LMO3, PRDM9 |  |
| Response To Oxygen-Containing Compound | 22 | 0 | 9.22E-03 | 0.009 | PDE3B, PIK3CG, PLAU, CCL2, IL24, IRAK3, CCND2, TBXAS1, PLCB1, ADGRL3, PTPN22, EPHA3, PDCD4, GRB14, GJA1, IL1B, HMGCS1, FOXA1, MMP3, IL1RL1, BCL11A, GDAP1 |  |
| Positive Regulation Of Lipid Catabolic Process | 2 | 7 | 9.32E-03 | 0.010 | AADAC, IL1B |  |
| Regulation Of Vascular Endothelial Growth Factor Signaling Pathway | 2 | 7 | 9.32E-03 | 0.010 | ROBO1, TCF4 |  |
| Regulation Of Cardiac Muscle Contraction By Regulation Of The Release Of Sequestered Calcium Ion | 2 | 7 | 9.32E-03 | 0.010 | DMD, CLIC2 |  |
| Regulation Of Toll-Like Receptor Signaling Pathway | 3 | 4 | 9.36E-03 | 0.012 | PTPN22, PIK3AP1, IRAK3 |  |
| Cellular Response To Peptide | 6 | 1 | 9.46E-03 | 0.012 | GRB14, PDE3B, IL1RL1, PLCB1, PTPN22, CCND2 |  |
| Negative Regulation Of Developmental Growth | 4 | 2 | 9.51E-03 | 0.013 | FGF13, BCL11A, EPHA7, GJA1 |  |
| Inorganic Ion Homeostasis | 11 | 1 | 1.00E-02 | 0.011 | GJA1, CXCR4, PIK3CG, EFHC1, IL1B, CCL2, GPR65, TBXAS1, KISS1, S1PR3, NEO1 |  |
| Positive Regulation Of Intracellular Steroid Hormone Receptor Signaling Pathway | 2 | 7 | 1.01E-02 | 0.010 | FOXA1, LMO3 |  |
| Positive Regulation Of Toll-Like Receptor Signaling Pathway | 2 | 7 | 1.01E-02 | 0.010 | PIK3AP1, PTPN22 |  |
| Positive Regulation Of Neutrophil Chemotaxis | 2 | 7 | 1.01E-02 | 0.010 | IL1B, NCKAP1L |  |
| Positive Regulation Of Calcium Ion Import | 2 | 7 | 1.01E-02 | 0.010 | CXCR4, CCL2 |  |
| Regulation Of Cellular Response To Vascular Endothelial Growth Factor Stimulus | 2 | 7 | 1.01E-02 | 0.010 | ROBO1, TCF4 |  |
| Secretion By Tissue | 2 | 7 | 1.01E-02 | 0.010 | GJA1, CHRM3 |  |
| Epithelial Tube Branching Involved In Lung Morphogenesis | 2 | 7 | 1.01E-02 | 0.010 | LAMA1, FOXA1 |  |
| Plasma Membrane Organization | 3 | 3 | 1.01E-02 | 0.012 | DMKN, EPB41L3, CXCR4 |  |
| Regulation Of Programmed Cell Death | 20 | 1 | 1.01E-02 | 0.009 | BCL2A1, PIK3CG, EFHC1, ROBO1, CARD8, CCL2, IL24, IRAK3, CCND2, EPHA7, EPHA3, PDCD4, SRPX, NCKAP1L, CXCR4, IL1B, FOXA1, CRADD, G0S2, SERPINB2 |  |
| Positive Regulation Of Cellular Protein Metabolic Process | 19 | 1 | 1.02E-02 | 0.010 | ARRDC4, PIK3CG, ROBO1, CARD8, CCL2, IL24, CCND2, PLCB1, FGF13, PTPN22, EPHA7, EPHA3, NCKAP1L, CXCR4, IL1B, SAMD5, CRADD, NRK, KISS1 |  |
| Regulation Of Embryonic Development | 4 | 2 | 1.05E-02 | 0.012 | NRK, LAMA1, LAMA4, PLCB1 |  |
| Positive Regulation Of Protein Secretion | 6 | 1 | 1.05E-02 | 0.012 | IL1RL1, GJA1, CYP2J2, PTPN22, IL1B, CARD8 |  |
| Positive Regulation Of Synapse Assembly | 3 | 3 | 1.08E-02 | 0.012 | SLITRK1, FLRT2, ADGRL3 |  |
| Cellular Response To Follicle-Stimulating Hormone Stimulus | 2 | 7 | 1.08E-02 | 0.010 | HMGCS1, EPHA3 |  |
| Positive Regulation Of Endothelial Cell Apoptotic Process | 2 | 7 | 1.08E-02 | 0.010 | PDCD4, CCL2 |  |
| Angiogenesis | 6 | 1 | 1.11E-02 | 0.012 | PDE3B, PIK3CG, PLAU, NRP2, CCL2, ESM1 |  |
| Negative Regulation Of Cellular Component Movement | 7 | 1 | 1.12E-02 | 0.012 | ARHGDIB, KISS1, PLCB1, NAV3, ROBO1, CCL2, IL24 |  |
| Cellular Response To Mechanical Stimulus | 4 | 2 | 1.12E-02 | 0.012 | IL1B, IL13RA2, GJA1, CRADD |  |
| Regulation Of Cytokine Secretion | 5 | 2 | 1.12E-02 | 0.012 | PTPN22, IL1B, CARD8, IL1RL1, CYP2J2 |  |
| Regulation Of Cellular Component Biogenesis | 13 | 1 | 1.13E-02 | 0.010 | NCKAP1L, GJA1, NAV3, SLITRK1, STXBP6, GPR65, GMFG, MMP3, ADGRL3, TBC1D30, FLRT2, EPHA7, EPHA3 |  |
| Negative Regulation Of Transferase Activity | 6 | 1 | 1.13E-02 | 0.012 | PDCD4, GMFG, PKIA, PTPN22, IL1B, IRAK3 |  |
| Regulation Of I-Kappab Kinase/Nf-Kappab Signaling | 5 | 2 | 1.14E-02 | 0.012 | IL1B, CARD8, IL1RL1, PIK3AP1, GJA1 |  |
| Negative Regulation Of Wound Healing | 3 | 3 | 1.16E-02 | 0.012 | SERPINB2, GJA1, PLAU |  |
| Positive Regulation Of Nik/Nf-Kappab Signaling | 3 | 3 | 1.16E-02 | 0.012 | PDCD4, IL1B, SAMD5 |  |
| Cellular Response To Fluid Shear Stress | 2 | 7 | 1.16E-02 | 0.010 | PLAU, TFPI2 |  |
| Helper T Cell Extravasation | 1 | 50 | 1.17E-02 | 0.005 | CCL2 |  |
| Positive Regulation Of Platelet-Derived Growth Factor Production | 1 | 50 | 1.17E-02 | 0.005 | SERPINB7 |  |
| Atrial Ventricular Junction Remodeling | 1 | 50 | 1.17E-02 | 0.005 | GJA1 |  |
| Fasciculation Of Motor Neuron Axon | 1 | 50 | 1.17E-02 | 0.005 | EPHA3 |  |
| Ganglion Morphogenesis | 1 | 50 | 1.17E-02 | 0.005 | NRP2 |  |
| Response To Low Light Intensity Stimulus | 1 | 50 | 1.17E-02 | 0.005 | HMGCS1 |  |
| Regulation Of Cell Communication By Chemical Coupling | 1 | 50 | 1.17E-02 | 0.005 | GJA1 |  |
| Positive Regulation Of Cell Communication By Chemical Coupling | 1 | 50 | 1.17E-02 | 0.005 | GJA1 |  |
| Activation Of Meiosis Involved In Egg Activation | 1 | 50 | 1.17E-02 | 0.005 | PLCB1 |  |
| Olfactory Nerve Structural Organization | 1 | 50 | 1.17E-02 | 0.005 | DMD |  |
| Vestibulocochlear Nerve Structural Organization | 1 | 50 | 1.17E-02 | 0.005 | NRP2 |  |
| Chemorepulsion Involved In Postnatal Olfactory Bulb Interneuron Migration | 1 | 50 | 1.17E-02 | 0.005 | ROBO1 |  |
| Galactolipid Catabolic Process | 1 | 50 | 1.17E-02 | 0.005 | GALC |  |
| Cerebellar Granule Cell Precursor Tangential Migration | 1 | 50 | 1.17E-02 | 0.005 | PLXNA2 |  |
| Epithelial-Mesenchymal Signaling Involved In Prostate Gland Development | 1 | 50 | 1.17E-02 | 0.005 | FOXA1 |  |
| Regulation Of Mesenchymal Stem Cell Migration | 1 | 50 | 1.17E-02 | 0.005 | CXCR4 |  |
| Positive Regulation Of Mesenchymal Stem Cell Migration | 1 | 50 | 1.17E-02 | 0.005 | CXCR4 |  |
| Regulation Of Acid-Sensing Ion Channel Activity | 1 | 50 | 1.17E-02 | 0.005 | STOM |  |
| Regulation Of Cell Proliferation Involved In Embryonic Placenta Development | 1 | 50 | 1.17E-02 | 0.005 | NRK |  |
| Dorsal Root Ganglion Morphogenesis | 1 | 50 | 1.17E-02 | 0.005 | NRP2 |  |
| Regulation Of Spongiotrophoblast Cell Proliferation | 1 | 50 | 1.17E-02 | 0.005 | NRK |  |
| Negative Regulation Of Negative Chemotaxis | 1 | 50 | 1.17E-02 | 0.005 | ROBO1 |  |
| Metal Ion Homeostasis | 10 | 1 | 1.18E-02 | 0.011 | GJA1, CXCR4, PIK3CG, EFHC1, IL1B, CCL2, GPR65, KISS1, S1PR3, NEO1 |  |
| Positive Regulation Of Transport | 14 | 1 | 1.20E-02 | 0.010 | NCKAP1L, GJA1, CXCR4, PLTP, IL1B, CARD8, CCL2, DMD, IL1RL1, KISS1, PLCB1, CYP2J2, PTPN22, STOM |  |
| Anatomical Structure Formation Involved In Morphogenesis | 11 | 1 | 1.21E-02 | 0.011 | PDE3B, PIK3CG, PLAU, PLXNA2, ROBO1, NRP2, CCL2, FOXA1, ESM1, EPB41L3, NEO1 |  |
| Regulation Of Interleukin-6 Production | 4 | 2 | 1.23E-02 | 0.012 | PTPN22, IL1B, NCKAP1L, IRAK3 |  |
| Negative Regulation Of Axonogenesis | 3 | 3 | 1.24E-02 | 0.011 | FGF13, BCL11A, EPHA7 |  |
| Positive Regulation Of Nuclear Division | 3 | 3 | 1.24E-02 | 0.011 | IL1B, PLCB1, PRDM9 |  |
| Cellular Ion Homeostasis | 10 | 1 | 1.24E-02 | 0.011 | GJA1, CXCR4, PIK3CG, EFHC1, IL1B, CCL2, GPR65, TBXAS1, KISS1, S1PR3 |  |
| Regulation Of Dendrite Extension | 2 | 6 | 1.24E-02 | 0.010 | BCL11A, CXCR4 |  |
| Positive Regulation Of Meiotic Cell Cycle | 2 | 6 | 1.24E-02 | 0.010 | PLCB1, PRDM9 |  |
| Myeloid Leukocyte Migration | 4 | 2 | 1.25E-02 | 0.012 | IL1B, NCKAP1L, CCL2, PIK3CG |  |
| Positive Regulation Of Cell Differentiation | 14 | 1 | 1.28E-02 | 0.010 | NCKAP1L, GJA1, CXCR4, PLXNA2, ROBO1, SLITRK1, IL1B, LMO3, FOXA1, DMD, TCF4, PLCB1, BCL11A, EPHA3 |  |
| Leukocyte Homeostasis | 3 | 3 | 1.28E-02 | 0.011 | NCKAP1L, BCL2A1, DOCK10 |  |
| Regulation Of Peptide Transport | 11 | 1 | 1.29E-02 | 0.011 | PKIA, PDE3B, GJA1, IL1B, CARD8, IL1RL1, KISS1, CYP2J2, PTPN22, STOM, NEO1 |  |
| Regulation Of Cell Growth | 8 | 1 | 1.30E-02 | 0.011 | HRASLS, EPB41L3, GJA1, CXCR4, FGF13, BCL11A, ESM1, EPHA7 |  |
| T Cell Migration | 2 | 6 | 1.33E-02 | 0.009 | PIK3CG, CCL2 |  |
| Positive Regulation Of Granulocyte Chemotaxis | 2 | 6 | 1.33E-02 | 0.009 | IL1B, NCKAP1L |  |
| Regulation Of Cardiac Muscle Contraction By Calcium Ion Signaling | 2 | 6 | 1.33E-02 | 0.009 | DMD, CLIC2 |  |
| Skeletal Muscle Tissue Regeneration | 2 | 6 | 1.33E-02 | 0.009 | GJA1, PLAU |  |
| Response To Organic Cyclic Compound | 15 | 1 | 1.34E-02 | 0.010 | GJA1, CXCR4, PIK3CG, PLAU, IL1B, CCL2, HMGCS1, FOXA1, IRAK3, CCND2, MMP3, PLCB1, ADGRL3, CYP2J2, GDAP1 |  |
| Enzyme Linked Receptor Protein Signaling Pathway | 10 | 1 | 1.35E-02 | 0.011 | PDCD4, GRB14, NCKAP1L, NRP2, PLCB1, SHC3, FLRT2, EPHA7, EPHA3, FAM83B |  |
| Positive Regulation Of Developmental Process | 18 | 1 | 1.36E-02 | 0.009 | PLXNA2, ROBO1, SLITRK1, LMO3, IRAK3, DMD, TCF4, PLCB1, ADGRL3, FLRT2, EPHA3, NCKAP1L, GJA1, CXCR4, IL1B, FOXA1, SERPINB7, BCL11A |  |
| Positive Regulation Of Reproductive Process | 3 | 3 | 1.36E-02 | 0.011 | PLCB1, PLAU, PRDM9 |  |
| Regulation Of Production Of Molecular Mediator Of Immune Response | 4 | 2 | 1.37E-02 | 0.012 | PTPN22, IL1B, IL13RA2, IRAK3 |  |
| Positive Regulation Of Cysteine-Type Endopeptidase Activity Involved In Apoptotic Process | 4 | 2 | 1.37E-02 | 0.012 | ROBO1, CARD8, IL24, CRADD |  |
| Negative Regulation Of Locomotion | 7 | 1 | 1.39E-02 | 0.011 | ARHGDIB, KISS1, PLCB1, NAV3, ROBO1, CCL2, IL24 |  |
| Regulation Of Chemotaxis | 5 | 2 | 1.40E-02 | 0.012 | ROBO1, IL1B, NCKAP1L, CCL2, CXCR4 |  |
| Oligodendrocyte Development | 2 | 6 | 1.41E-02 | 0.009 | KCNQ3, PRDM8 |  |
| Aorta Development | 2 | 6 | 1.41E-02 | 0.009 | ROBO1, CXCR4 |  |
| Regulation Of Cardiac Muscle Contraction | 3 | 3 | 1.45E-02 | 0.011 | DMD, CLIC2, PIK3CG |  |
| Cellular Chemical Homeostasis | 11 | 1 | 1.46E-02 | 0.010 | GJA1, CXCR4, PIK3CG, PLAU, EFHC1, IL1B, CCL2, GPR65, TBXAS1, KISS1, S1PR3 |  |
| Positive Regulation Of Jnk Cascade | 4 | 2 | 1.46E-02 | 0.012 | IL1B, NRK, SAMD5, PLCB1 |  |
| Regulation Of Ion Transmembrane Transport | 8 | 1 | 1.46E-02 | 0.011 | DMD, KCNQ3, GJA1, PLCB1, PIK3CG, PTPN22, CLIC2, STOM |  |
| Regulation Of Protein Secretion | 8 | 1 | 1.48E-02 | 0.011 | PDE3B, IL1RL1, GJA1, CYP2J2, PTPN22, IL1B, CARD8, NEO1 |  |
| Regulation Of Wound Healing | 4 | 2 | 1.49E-02 | 0.012 | SERPINB2, GJA1, CXCR4, PLAU |  |
| Negative Regulation Of Protein Complex Disassembly | 3 | 3 | 1.49E-02 | 0.011 | FGF13, NAV3, IRAK3 |  |
| Regulation Of T-Helper 1 Type Immune Response | 2 | 6 | 1.50E-02 | 0.009 | IL1RL1, IL1B |  |
| Decidualization | 2 | 6 | 1.50E-02 | 0.009 | KISS1, GJA1 |  |
| Outflow Tract Septum Morphogenesis | 2 | 6 | 1.50E-02 | 0.009 | ROBO1, NRP2 |  |
| Motor Neuron Axon Guidance | 2 | 6 | 1.50E-02 | 0.009 | EPHA3, CXCR4 |  |
| Regulation Of Ryanodine-Sensitive Calcium-Release Channel Activity | 2 | 6 | 1.50E-02 | 0.009 | DMD, CLIC2 |  |
| Memory | 4 | 2 | 1.52E-02 | 0.012 | FGF13, IL1B, PLCB1, CCND2 |  |
| Cellular Metal Ion Homeostasis | 9 | 1 | 1.54E-02 | 0.011 | GJA1, CXCR4, PIK3CG, EFHC1, IL1B, CCL2, GPR65, KISS1, S1PR3 |  |
| Regulation Of Transmembrane Transport | 9 | 1 | 1.58E-02 | 0.011 | GJA1, PIK3CG, IL1B, CLIC2, DMD, KCNQ3, PLCB1, PTPN22, STOM |  |
| Regulation Of Developmental Growth | 7 | 1 | 1.59E-02 | 0.011 | NRK, GJA1, PLCB1, CXCR4, FGF13, BCL11A, EPHA7 |  |
| Regulation Of Neutrophil Chemotaxis | 2 | 6 | 1.59E-02 | 0.009 | IL1B, NCKAP1L |  |
| Positive Regulation Of Rho Protein Signal Transduction | 2 | 6 | 1.59E-02 | 0.009 | GPR65, ROBO1 |  |
| Regulation Of Heart Contraction | 5 | 1 | 1.61E-02 | 0.012 | DMD, GJA1, PIK3CG, CYP2J2, CLIC2 |  |
| Response To Peptide | 8 | 1 | 1.62E-02 | 0.011 | GRB14, PDE3B, IL1RL1, GJA1, PLCB1, PTPN22, IL1B, CCND2 |  |
| Cell Chemotaxis | 5 | 1 | 1.64E-02 | 0.012 | NCKAP1L, CXCR4, PIK3CG, IL1B, CCL2 |  |
| Protein Phosphorylation | 12 | 1 | 1.66E-02 | 0.010 | PIK3CG, IL1B, CCL2, LAMA1, IL24, IRAK3, GMFG, NRK, FGF13, SHC3, EPHA7, EPHA3 |  |
| Anatomical Structure Morphogenesis | 18 | 1 | 1.66E-02 | 0.009 | ROBO1, SLITRK1, CCL2, LAMA1, DOCK10, EPB41L3, PRDM8, S1PR3, FLRT2, EPHA7, NCKAP1L, GJA1, CXCR4, POF1B, NRP2, FOXA1, CRISPLD1, NRK |  |
| Response To Follicle-Stimulating Hormone | 2 | 5 | 1.68E-02 | 0.009 | HMGCS1, EPHA3 |  |
| Regulation Of Cell Maturation | 2 | 5 | 1.68E-02 | 0.009 | BCL11A, GRB14 |  |
| Cellular Response To External Stimulus | 7 | 1 | 1.70E-02 | 0.011 | MMP3, GJA1, BCL11A, IL1B, IL13RA2, GDAP1, CRADD |  |
| Maturation Of 5.8S Rrna From Tricistronic Rrna Transcript (Ssu-Rrna, 5.8S Rrna, Lsu-Rrna) | 1 | 33 | 1.74E-02 | 0.005 | FTSJ3 |  |
| Epicardial Cell To Mesenchymal Cell Transition | 1 | 33 | 1.74E-02 | 0.005 | GJA1 |  |
| Gonadotrophin-Releasing Hormone Neuronal Migration To The Hypothalamus | 1 | 33 | 1.74E-02 | 0.005 | NRP2 |  |
| Regulation Of Platelet-Derived Growth Factor Production | 1 | 33 | 1.74E-02 | 0.005 | SERPINB7 |  |
| Regulation Of Granzyme B Production | 1 | 33 | 1.74E-02 | 0.005 | PTPN22 |  |
| Positive Regulation Of Granzyme B Production | 1 | 33 | 1.74E-02 | 0.005 | PTPN22 |  |
| Negative Regulation Of Nucleotide-Binding Oligomerization Domain Containing Signaling Pathway | 1 | 33 | 1.74E-02 | 0.005 | PTPN22 |  |
| Cell Communication By Chemical Coupling | 1 | 33 | 1.74E-02 | 0.005 | GJA1 |  |
| Establishment Or Maintenance Of Neuroblast Polarity | 1 | 33 | 1.74E-02 | 0.005 | FGF13 |  |
| Substrate-Dependent Cerebral Cortex Tangential Migration | 1 | 33 | 1.74E-02 | 0.005 | NRP2 |  |
| Sensory Neuron Axon Guidance | 1 | 33 | 1.74E-02 | 0.005 | NRP2 |  |
| Fasciculation Of Sensory Neuron Axon | 1 | 33 | 1.74E-02 | 0.005 | EPHA3 |  |
| Isoprenoid Biosynthetic Process Via Mevalonate | 1 | 33 | 1.74E-02 | 0.005 | HMGCS1 |  |
| Positive Regulation Of Reciprocal Meiotic Recombination | 1 | 33 | 1.74E-02 | 0.005 | PRDM9 |  |
| Farnesyl Diphosphate Biosynthetic Process, Mevalonate Pathway | 1 | 33 | 1.74E-02 | 0.005 | HMGCS1 |  |
| Positive Regulation Of Vitamin D Biosynthetic Process | 1 | 33 | 1.74E-02 | 0.005 | IL1B |  |
| Regulation Of Meiotic Cell Cycle Process Involved In Oocyte Maturation | 1 | 33 | 1.74E-02 | 0.005 | GRB14 |  |
| Presynaptic Active Zone Organization | 1 | 33 | 1.74E-02 | 0.005 | PCDH17 |  |
| Establishment Of Glial Blood-Brain Barrier | 1 | 33 | 1.74E-02 | 0.005 | DMD |  |
| Cerebral Cortex Tangential Migration Using Cell-Axon Interactions | 1 | 33 | 1.74E-02 | 0.005 | NRP2 |  |
| Hypothalamic Tangential Migration Using Cell-Axon Interactions | 1 | 33 | 1.74E-02 | 0.005 | NRP2 |  |
| Interleukin-33-Mediated Signaling Pathway | 1 | 33 | 1.74E-02 | 0.005 | IL1RL1 |  |
| Generation Of Ovulation Cycle Rhythm | 1 | 33 | 1.74E-02 | 0.005 | KISS1 |  |
| Positive Regulation Of Vitamin Metabolic Process | 1 | 33 | 1.74E-02 | 0.005 | IL1B |  |
| Establishment Of Neuroblast Polarity | 1 | 33 | 1.74E-02 | 0.005 | FGF13 |  |
| Phosphatidylinositol Catabolic Process | 1 | 33 | 1.74E-02 | 0.005 | PLCB1 |  |
| Positive Regulation Of Calcidiol 1-Monooxygenase Activity | 1 | 33 | 1.74E-02 | 0.005 | IL1B |  |
| Natural Killer Cell Chemotaxis | 1 | 33 | 1.74E-02 | 0.005 | PIK3CG |  |
| Positive Regulation Of Protein K63-Linked Ubiquitination | 1 | 33 | 1.74E-02 | 0.005 | PTPN22 |  |
| Cellular Response To Fluoride | 1 | 33 | 1.74E-02 | 0.005 | PLCB1 |  |
| Facioacoustic Ganglion Development | 1 | 33 | 1.74E-02 | 0.005 | NRP2 |  |
| Negative Regulation Of Myofibroblast Differentiation | 1 | 33 | 1.74E-02 | 0.005 | PDCD4 |  |
| Alveolar Secondary Septum Development | 1 | 33 | 1.74E-02 | 0.005 | FOXA1 |  |
| Monocyte Aggregation | 1 | 33 | 1.74E-02 | 0.005 | IL1B |  |
| Negative Regulation Of Nucleotide-Binding Oligomerization Domain Containing 2 Signaling Pathway | 1 | 33 | 1.74E-02 | 0.005 | PTPN22 |  |
| Ion Homeostasis | 11 | 1 | 1.76E-02 | 0.010 | GJA1, CXCR4, PIK3CG, EFHC1, IL1B, CCL2, GPR65, TBXAS1, KISS1, S1PR3, NEO1 |  |
| Cellular Component Organization Or Biogenesis | 49 | 0 | 1.77E-02 | 0.008 | ARRDC4, EFHC1, SLITRK1, DMKN, SYTL3, DMD, FTSJ3, FGF13, STOM, EPHA3, FAM172BP, NCKAP1L, GJA1, TTLL11, ZDHHC15, TOR1AIP2, FOXA1, MMP3, NRK, KISS1, LAMA4, PLTP, ROBO1, CCL2, LAMA1, TCF4, HIST2H4B, SRPX, CXCR4, GPR65, GMFG, EPB41L3, ADGRL3, PCDHB5, FLRT2, ABI3BP, PCDH17, MAFIP, GDAP1, PCDHB6, DOCK10, HRASLS, ATP8B2, PCDHB2, PRDM9, PRDM8, FKBP10, PCDHB16, POF1B |  |
| Negative Regulation Of Microtubule Depolymerization | 2 | 5 | 1.78E-02 | 0.009 | FGF13, NAV3 |  |
| Actin Filament-Based Process | 7 | 1 | 1.79E-02 | 0.011 | GMFG, DMD, NRK, NCKAP1L, EPB41L3, POF1B, GPR65 |  |
| Regulation Of Apoptotic Process | 19 | 0 | 1.80E-02 | 0.009 | BCL2A1, PIK3CG, EFHC1, ROBO1, CARD8, CCL2, IL24, IRAK3, CCND2, EPHA7, EPHA3, PDCD4, SRPX, NCKAP1L, IL1B, FOXA1, CRADD, G0S2, SERPINB2 |  |
| Negative Regulation Of Neurogenesis | 6 | 1 | 1.80E-02 | 0.011 | FGF13, BCL11A, IL1B, EPHA7, EPHA3, NEO1 |  |
| Response To Estradiol | 5 | 1 | 1.85E-02 | 0.011 | MMP3, GJA1, IL1B, FOXA1, CCND2 |  |
| Positive Regulation Of Neutrophil Migration | 2 | 5 | 1.88E-02 | 0.009 | IL1B, NCKAP1L |  |
| Cellular Homeostasis | 12 | 1 | 1.88E-02 | 0.010 | GJA1, CXCR4, PIK3CG, PLAU, EFHC1, IL1B, CCL2, GPR65, DMD, TBXAS1, KISS1, S1PR3 |  |
| Response To Alkaloid | 4 | 2 | 1.89E-02 | 0.011 | IL1B, CXCR4, PLAU, ADGRL3 |  |
| G-Protein Coupled Receptor Signaling Pathway, Coupled To Cyclic Nucleotide Second Messenger | 5 | 1 | 1.91E-02 | 0.011 | CHRM3, CCL2, S1PR3, GPR65, LGR5 |  |
| Negative Regulation Of Cell Differentiation | 11 | 1 | 1.93E-02 | 0.010 | PDCD4, GRB14, HIST2H4B, IL1B, FOXA1, FGF13, BCL11A, S1PR3, EPHA7, EPHA3, NEO1 |  |
| Establishment Of Cell Polarity | 3 | 3 | 1.93E-02 | 0.011 | FGF13, LAMA1, GJA1 |  |
| Positive Regulation Of Jun Kinase Activity | 3 | 3 | 1.93E-02 | 0.011 | IL1B, NRK, SAMD5 |  |
| Negative Regulation Of Cell Proliferation | 11 | 1 | 1.94E-02 | 0.010 | PDCD4, SRPX, GJA1, CD33, ROBO1, IL1B, CCL2, IL24, NRK, IL1RL1, KISS1 |  |
| Regulation Of Response To Stress | 17 | 1 | 1.95E-02 | 0.009 | PIK3CG, PLAU, IRAK3, PIK3AP1, PLCB1, PTPN22, PDCD4, GJA1, CXCR4, IL1B, SAMD5, MMP3, NRK, IL1RL1, KISS1, SERPINB2, NEO1 |  |
| Negative Regulation Of Immune Response | 4 | 2 | 1.96E-02 | 0.011 | IL13RA2, SAMSN1, IL1RL1, IRAK3 |  |
| Positive Regulation Of Cysteine-Type Endopeptidase Activity | 4 | 2 | 1.96E-02 | 0.011 | ROBO1, CARD8, IL24, CRADD |  |
| Cardiac Muscle Cell Action Potential | 2 | 5 | 1.98E-02 | 0.009 | GJA1, DMD |  |
| Second-Messenger-Mediated Signaling | 6 | 1 | 1.98E-02 | 0.011 | DMD, CXCR4, MCTP2, CLIC2, GPR65, LGR5 |  |
| Regulation Of Epithelial To Mesenchymal Transition | 3 | 3 | 1.98E-02 | 0.011 | IL1B, FOXA1, EPHA3 |  |
| Negative Regulation Of Nitrogen Compound Metabolic Process | 26 | 0 | 2.02E-02 | 0.008 | PKIA, BCL2A1, CARD8, LMO3, HMSD, IL24, IRAK3, DMD, TCF4, PLCB1, PRDM8, PTPN22, SAMSN1, FAM172BP, PDCD4, HIST2H4B, NCKAP1L, GJA1, TFPI2, IL1B, FOXA1, SERPINB7, GMFG, BCL11A, SERPINB2, ZNF438 |  |
| Positive Regulation Of Inflammatory Response | 4 | 2 | 2.03E-02 | 0.011 | PDCD4, IL1B, IL1RL1, PIK3CG |  |
| Chemical Homeostasis | 14 | 1 | 2.06E-02 | 0.009 | PDE3B, GJA1, CXCR4, PIK3CG, PLAU, EFHC1, IL1B, CCL2, GPR65, FOXA1, TBXAS1, KISS1, S1PR3, NEO1 |  |
| Semaphorin-Plexin Signaling Pathway | 2 | 5 | 2.08E-02 | 0.009 | NRP2, PLXNA2 |  |
| Regulation Of Release Of Sequestered Calcium Ion Into Cytosol By Sarcoplasmic Reticulum | 2 | 5 | 2.08E-02 | 0.009 | DMD, CLIC2 |  |
| Negative Regulation Of Exocytosis | 2 | 5 | 2.08E-02 | 0.009 | IL13RA2, STXBP6 |  |
| Cellular Response To Gonadotropin Stimulus | 2 | 5 | 2.08E-02 | 0.009 | HMGCS1, EPHA3 |  |
| T Cell Activation | 5 | 1 | 2.14E-02 | 0.011 | BCL2A1, CXCR4, PIK3CG, BCL11A, PTPN22 |  |
| Calcium-Mediated Signaling | 4 | 2 | 2.15E-02 | 0.011 | MCTP2, DMD, CLIC2, CXCR4 |  |
| Regulation Of Anatomical Structure Morphogenesis | 14 | 1 | 2.16E-02 | 0.009 | PDE3B, GJA1, CXCR4, PLAU, PLXNA2, ROBO1, SLITRK1, IL1B, CCL2, TCF4, EPB41L3, FGF13, BCL11A, EPHA7 |  |
| Regulation Of Cell Morphogenesis Involved In Differentiation | 6 | 1 | 2.18E-02 | 0.011 | PLXNA2, ROBO1, FGF13, BCL11A, SLITRK1, EPHA7 |  |
| Negative Regulation Of Production Of Molecular Mediator Of Immune Response | 2 | 5 | 2.18E-02 | 0.009 | IL13RA2, IRAK3 |  |
| Establishment Of Epithelial Cell Polarity | 2 | 5 | 2.18E-02 | 0.009 | LAMA1, GJA1 |  |
| Regulation Of Microtubule Depolymerization | 2 | 5 | 2.18E-02 | 0.009 | FGF13, NAV3 |  |
| Regulation Of Actin Cytoskeleton Reorganization | 2 | 5 | 2.18E-02 | 0.009 | GMFG, ARHGDIB |  |
| Regulation Of Biological Process | 103 | 0 | 2.19E-02 | 0.007 | ARHGDIB, BCL2A1, AADAC, CD33, CHRM3, CLIC2, CCND2, DMD, CYP2J2, FGF13, S1PR3, STOM, EPHA7, EPHA3, GRB14, NCKAP1L, GJA1, GNGT2, IL1B, IL13RA2, HMGCS1, FOXA1, MMP3, KCNQ3, KISS1, LAMA4, SERPINB2, NEO1, PKIA, PDE3B, PIK3CG, PLAU, PLCB4, PLXNA2, PLTP, ROBO1, CCL2, RGS10, TBXAS1, TCF4, HIST2H4B, SRPX, CXCR4, TFPI2, NRP2, GPR65, LGR5, SERPINB7, CRADD, GMFG, IL1RL1, RAPGEF5, CLEC2B, CNKSR2, CARD8, IL24, ESM1, IRAK3, SP140, EPB41L3, PLCB1, ADGRL3, TBC1D30, PTPN22, PITPNC1, FLRT2, ABI3BP, PDCD4, PCDH17, PCDH11X, OR2H1, STXBP6, G0S2, BCL11A, SHC3, MCTP2, LMO3, DOCK10, HRASLS, PRDM9, PRDM8, SAMSN1, GPR135, ANKRD30A, ARRDC4, NAV3, EFHC1, SLITRK1, SYTL3, OR2M3, PIK3AP1, FOXR2, FAM172BP, NRK, OR5B21, ZNF438, FAM83B, SSX8, LAMA1, HMSD, SAMD5, ZNF716, MAFIP |  |
| Cation Homeostasis | 10 | 1 | 2.23E-02 | 0.010 | GJA1, CXCR4, PIK3CG, EFHC1, IL1B, CCL2, GPR65, KISS1, S1PR3, NEO1 |  |
| Phosphorylation | 14 | 1 | 2.25E-02 | 0.009 | PIK3CG, PLAU, IL1B, CCL2, LAMA1, IL24, IRAK3, GMFG, NRK, PIK3AP1, FGF13, SHC3, EPHA7, EPHA3 |  |
| Negative Regulation Of Lipid Metabolic Process | 3 | 2 | 2.26E-02 | 0.011 | IL1B, PDE3B, PIK3CG |  |
| Regulation Of Protein Transport | 10 | 1 | 2.27E-02 | 0.010 | PKIA, PDE3B, GJA1, IL1B, CARD8, IL1RL1, CYP2J2, PTPN22, STOM, NEO1 |  |
| Regulation Of Cell Proliferation | 20 | 0 | 2.28E-02 | 0.009 | PLAU, CD33, ROBO1, CCL2, IL24, ESM1, CCND2, PTPN22, S1PR3, PDCD4, SRPX, NCKAP1L, GJA1, NRP2, IL1B, LGR5, SERPINB7, NRK, IL1RL1, KISS1 |  |
| Signal Transduction In Absence Of Ligand | 2 | 5 | 2.29E-02 | 0.009 | IL1B, BCL2A1 |  |
| Extrinsic Apoptotic Signaling Pathway In Absence Of Ligand | 2 | 5 | 2.29E-02 | 0.009 | IL1B, BCL2A1 |  |
| Regulation Of Reciprocal Meiotic Recombination | 1 | 25 | 2.32E-02 | 0.005 | PRDM9 |  |
| Cornified Envelope Assembly | 1 | 25 | 2.32E-02 | 0.005 | DMKN |  |
| Regulation Of Smooth Muscle Cell-Matrix Adhesion | 1 | 25 | 2.32E-02 | 0.005 | PLAU |  |
| Trunk Neural Crest Cell Migration | 1 | 25 | 2.32E-02 | 0.005 | NRP2 |  |
| Toll-Like Receptor 7 Signaling Pathway | 1 | 25 | 2.32E-02 | 0.005 | PIK3AP1 |  |
| Secretory Columnal Luminar Epithelial Cell Differentiation Involved In Prostate Glandular Acinus Development | 1 | 25 | 2.32E-02 | 0.005 | FOXA1 |  |
| Epithelial Cell Maturation Involved In Prostate Gland Development | 1 | 25 | 2.32E-02 | 0.005 | FOXA1 |  |
| Regulation Of Monocyte Extravasation | 1 | 25 | 2.32E-02 | 0.005 | PLCB1 |  |
| Prostate Gland Stromal Morphogenesis | 1 | 25 | 2.32E-02 | 0.005 | FOXA1 |  |
| Positive Regulation Of Glomerular Filtration | 1 | 25 | 2.32E-02 | 0.005 | GJA1 |  |
| Meiotic Gene Conversion | 1 | 25 | 2.32E-02 | 0.005 | PRDM9 |  |
| Chromatin Silencing By Small Rna | 1 | 25 | 2.32E-02 | 0.005 | FAM172BP |  |
| Vitamin E Metabolic Process | 1 | 25 | 2.32E-02 | 0.005 | PLTP |  |
| Negative Regulation Of Cell Proliferation Involved In Contact Inhibition | 1 | 25 | 2.32E-02 | 0.005 | SRPX |  |
| Response To Ultrasound | 1 | 25 | 2.32E-02 | 0.005 | CXCR4 |  |
| Regulation Of Hepatocyte Growth Factor Receptor Signaling Pathway | 1 | 25 | 2.32E-02 | 0.005 | ESM1 |  |
| Negative Regulation Of Peroxisome Proliferator Activated Receptor Signaling Pathway | 1 | 25 | 2.32E-02 | 0.005 | CYP2J2 |  |
| Smooth Muscle Adaptation | 1 | 25 | 2.32E-02 | 0.005 | IL1B |  |
| Negative Regulation Of Dendrite Extension | 1 | 25 | 2.32E-02 | 0.005 | BCL11A |  |
| Activation Of Meiosis | 1 | 25 | 2.32E-02 | 0.005 | PLCB1 |  |
| Sequestering Of Triglyceride | 1 | 25 | 2.32E-02 | 0.005 | IL1B |  |
| Hindbrain Tangential Cell Migration | 1 | 25 | 2.32E-02 | 0.005 | PLXNA2 |  |
| Sympathetic Neuron Projection Guidance | 1 | 25 | 2.32E-02 | 0.005 | NRP2 |  |
| Sympathetic Neuron Projection Extension | 1 | 25 | 2.32E-02 | 0.005 | NRP2 |  |
| Positive Regulation Of Luteinizing Hormone Secretion | 1 | 25 | 2.32E-02 | 0.005 | KISS1 |  |
| Positive Regulation Of Estradiol Secretion | 1 | 25 | 2.32E-02 | 0.005 | IL1B |  |
| Positive Regulation Of Toll-Like Receptor 7 Signaling Pathway | 1 | 25 | 2.32E-02 | 0.005 | PTPN22 |  |
| Positive Regulation Of Immature T Cell Proliferation In Thymus | 1 | 25 | 2.32E-02 | 0.005 | IL1B |  |
| Ventral Trunk Neural Crest Cell Migration | 1 | 25 | 2.32E-02 | 0.005 | NRP2 |  |
| Cellular Response To Vasopressin | 1 | 25 | 2.32E-02 | 0.005 | PLCB1 |  |
| Positive Regulation Of Cd8-Positive, Alpha-Beta T Cell Differentiation | 1 | 25 | 2.32E-02 | 0.005 | NCKAP1L |  |
| Positive Regulation Of Ovulation | 1 | 25 | 2.32E-02 | 0.005 | PLAU |  |
| Epididymis Development | 1 | 25 | 2.32E-02 | 0.005 | GJA1 |  |
| Extracellular Vesicle Biogenesis | 1 | 25 | 2.32E-02 | 0.005 | ARRDC4 |  |
| Epithelial Cell Proliferation Involved In Renal Tubule Morphogenesis | 1 | 25 | 2.32E-02 | 0.005 | LGR5 |  |
| Negative Regulation Of Adiponectin Secretion | 1 | 25 | 2.32E-02 | 0.005 | IL1B |  |
| Cellular Response To Redox State | 1 | 25 | 2.32E-02 | 0.005 | ARHGDIB |  |
| Epithelial To Mesenchymal Transition Involved In Cardiac Fibroblast Development | 1 | 25 | 2.32E-02 | 0.005 | PDCD4 |  |
| Regulation Of Negative Chemotaxis | 1 | 25 | 2.32E-02 | 0.005 | ROBO1 |  |
| Regulation Of Phagocytosis | 3 | 2 | 2.32E-02 | 0.011 | IL1B, NCKAP1L, CCL2 |  |
| Negative Regulation Of Proteolysis | 7 | 1 | 2.34E-02 | 0.011 | BCL2A1, TFPI2, CARD8, SERPINB2, HMSD, IL24, SERPINB7 |  |
| Cellular Component Morphogenesis | 8 | 1 | 2.34E-02 | 0.010 | NRK, NCKAP1L, EPB41L3, GJA1, PRDM8, POF1B, SLITRK1, DOCK10 |  |
| Regulation Of Sodium Ion Transport | 3 | 2 | 2.38E-02 | 0.011 | DMD, STOM, PLCB1 |  |
| Positive Regulation Of Cytokine Production | 7 | 1 | 2.39E-02 | 0.011 | IL1RL1, PLCB1, CYP2J2, PTPN22, IL1B, CARD8, SERPINB7 |  |
| Temperature Homeostasis | 2 | 4 | 2.40E-02 | 0.009 | IL1B, EPHA3 |  |
| Ventricular Septum Development | 2 | 4 | 2.40E-02 | 0.009 | ROBO1, CXCR4 |  |
| Regulation Of Meiotic Nuclear Division | 2 | 4 | 2.40E-02 | 0.009 | PLCB1, PRDM9 |  |
| Positive Regulation Of Cell Adhesion | 7 | 1 | 2.41E-02 | 0.011 | DMD, NCKAP1L, PTPN22, IL1B, CCL2, FOXA1, ABI3BP |  |
| Negative Regulation Of Nervous System Development | 6 | 1 | 2.42E-02 | 0.011 | FGF13, BCL11A, IL1B, EPHA7, EPHA3, NEO1 |  |
| Positive Regulation Of Mitotic Cell Cycle | 4 | 2 | 2.47E-02 | 0.011 | IL1B, FOXA1, PLCB1, CCND2 |  |
| Regulation Of Striated Muscle Contraction | 3 | 2 | 2.49E-02 | 0.010 | DMD, CLIC2, PIK3CG |  |
| Negative Regulation Of Toll-Like Receptor Signaling Pathway | 2 | 4 | 2.51E-02 | 0.009 | PIK3AP1, IRAK3 |  |
| Negative Regulation Of Insulin Receptor Signaling Pathway | 2 | 4 | 2.51E-02 | 0.009 | GRB14, IL1B |  |
| Negative Regulation Of Jnk Cascade | 2 | 4 | 2.51E-02 | 0.009 | PDCD4, PTPN22 |  |
| Protein Kinase B Signaling | 2 | 4 | 2.51E-02 | 0.009 | IL1B, CCL2 |  |
| Establishment Or Maintenance Of Cell Polarity | 4 | 2 | 2.55E-02 | 0.011 | FGF13, NCKAP1L, LAMA1, GJA1 |  |
| Cell-Cell Junction Assembly | 3 | 2 | 2.56E-02 | 0.010 | POF1B, EPB41L3, GJA1 |  |
| Artery Development | 2 | 4 | 2.62E-02 | 0.009 | ROBO1, CXCR4 |  |
| Positive Regulation Of Sodium Ion Transport | 2 | 4 | 2.62E-02 | 0.009 | DMD, PLCB1 |  |
| Tissue Development | 10 | 1 | 2.62E-02 | 0.010 | GJA1, PLAU, PLXNA2, NRP2, LAMA1, FOXA1, DMD, KCNQ3, KISS1, EPHA3 |  |
| Positive Regulation Of Secretion By Cell | 7 | 1 | 2.65E-02 | 0.010 | IL1RL1, KISS1, GJA1, CYP2J2, PTPN22, IL1B, CARD8 |  |
| Positive Regulation Of Protein Transport | 7 | 1 | 2.65E-02 | 0.010 | IL1RL1, GJA1, CYP2J2, PTPN22, IL1B, CARD8, STOM |  |
| Regulation Of Cellular Process | 96 | 0 | 2.65E-02 | 0.007 | ARHGDIB, BCL2A1, AADAC, CD33, CHRM3, CLIC2, CCND2, DMD, CYP2J2, FGF13, S1PR3, EPHA7, EPHA3, GRB14, NCKAP1L, GJA1, GNGT2, IL1B, IL13RA2, FOXA1, MMP3, KISS1, LAMA4, SERPINB2, NEO1, PKIA, PDE3B, PIK3CG, PLAU, PLCB4, PLXNA2, ROBO1, CCL2, RGS10, TCF4, HIST2H4B, SRPX, CXCR4, TFPI2, NRP2, GPR65, LGR5, SERPINB7, CRADD, GMFG, IL1RL1, RAPGEF5, CNKSR2, CARD8, IL24, ESM1, IRAK3, SP140, EPB41L3, PLCB1, ADGRL3, TBC1D30, PTPN22, PITPNC1, FLRT2, PDCD4, PCDH17, PCDH11X, OR2H1, STXBP6, G0S2, BCL11A, SHC3, MCTP2, LMO3, DOCK10, HRASLS, PRDM9, PRDM8, SAMSN1, GPR135, ANKRD30A, ARRDC4, NAV3, EFHC1, SLITRK1, SYTL3, OR2M3, PIK3AP1, FOXR2, FAM172BP, NRK, OR5B21, ZNF438, FAM83B, SSX8, LAMA1, HMSD, SAMD5, ZNF716, MAFIP |  |
| Activation Of Cysteine-Type Endopeptidase Activity Involved In Apoptotic Process | 3 | 2 | 2.68E-02 | 0.010 | ROBO1, CARD8, CRADD |  |
| Positive Regulation Of Erk1 And Erk2 Cascade | 5 | 1 | 2.73E-02 | 0.011 | PTPN22, IL1B, CCL2, EPHA7, EPHA3 |  |
| Regulation Of Cation Transmembrane Transport | 6 | 1 | 2.73E-02 | 0.011 | DMD, PLCB1, PIK3CG, PTPN22, CLIC2, STOM |  |
| Positive Regulation Of Epithelial Cell Apoptotic Process | 2 | 4 | 2.73E-02 | 0.009 | PDCD4, CCL2 |  |
| Regulation Of System Process | 9 | 1 | 2.76E-02 | 0.010 | GJA1, PIK3CG, IL1B, CHRM3, CLIC2, DMD, TBXAS1, KISS1, CYP2J2 |  |
| Regulation Of Calcium Ion Transmembrane Transport | 4 | 2 | 2.77E-02 | 0.011 | PTPN22, DMD, CLIC2, PIK3CG |  |
| Morphogenesis Of A Branching Structure | 4 | 2 | 2.82E-02 | 0.011 | LAMA1, FOXA1, EPHA7, CXCR4 |  |
| Phosphate-Containing Compound Metabolic Process | 21 | 0 | 2.82E-02 | 0.008 | PDE3B, PIK3CG, PLAU, PLCB4, ENTPD3, CCL2, LAMA1, IL24, IRAK3, PIK3AP1, PLCB1, FGF13, PTPN22, EPHA7, EPHA3, IL1B, HMGCS1, HDDC2, GMFG, NRK, SHC3 |  |
| Cellular Cation Homeostasis | 9 | 1 | 2.82E-02 | 0.010 | GJA1, CXCR4, PIK3CG, EFHC1, IL1B, CCL2, GPR65, KISS1, S1PR3 |  |
| Cellular Response To Organic Substance | 20 | 0 | 2.85E-02 | 0.009 | PDE3B, PIK3CG, PLAU, CCL2, IL24, CCND2, PLCB1, PTPN22, EPHA3, PDCD4, GRB14, GJA1, CXCR4, NRP2, IL1B, HMGCS1, MMP3, IL1RL1, BCL11A, GDAP1 |  |
| Negative Regulation Of Leukocyte Migration | 2 | 4 | 2.85E-02 | 0.009 | PLCB1, CCL2 |  |
| Cortical Actin Cytoskeleton Organization | 2 | 4 | 2.85E-02 | 0.009 | EPB41L3, NCKAP1L |  |
| Negative Regulation Of I-Kappab Kinase/Nf-Kappab Signaling | 2 | 4 | 2.85E-02 | 0.009 | IL1RL1, CARD8 |  |
| Regulation Of Muscle Contraction | 4 | 2 | 2.86E-02 | 0.011 | DMD, CHRM3, CLIC2, PIK3CG |  |
| Positive Regulation Of Cell Morphogenesis Involved In Differentiation | 4 | 2 | 2.86E-02 | 0.011 | ROBO1, BCL11A, SLITRK1, PLXNA2 |  |
| T Cell Extravasation | 1 | 20 | 2.89E-02 | 0.005 | CCL2 |  |
| Branching Morphogenesis Of A Nerve | 1 | 20 | 2.89E-02 | 0.005 | EPHA7 |  |
| Chronic Inflammatory Response To Antigenic Stimulus | 1 | 20 | 2.89E-02 | 0.005 | IL1B |  |
| Negative Regulation Of Chemokine-Mediated Signaling Pathway | 1 | 20 | 2.89E-02 | 0.005 | ROBO1 |  |
| Protein Localization To Paranode Region Of Axon | 1 | 20 | 2.89E-02 | 0.005 | EPB41L3 |  |
| Negative Regulation Of Macrophage Cytokine Production | 1 | 20 | 2.89E-02 | 0.005 | IRAK3 |  |
| Gene Conversion | 1 | 20 | 2.89E-02 | 0.005 | PRDM9 |  |
| Cell Adhesion Involved In Heart Morphogenesis | 1 | 20 | 2.89E-02 | 0.005 | FLRT2 |  |
| Regulation Of Notch Signaling Pathway Involved In Heart Induction | 1 | 20 | 2.89E-02 | 0.005 | ROBO1 |  |
| Farnesyl Diphosphate Biosynthetic Process | 1 | 20 | 2.89E-02 | 0.005 | HMGCS1 |  |
| Negative Regulation Of Triglyceride Catabolic Process | 1 | 20 | 2.89E-02 | 0.005 | PIK3CG |  |
| Axon Midline Choice Point Recognition | 1 | 20 | 2.89E-02 | 0.005 | ROBO1 |  |
| Serine Phosphorylation Of Stat Protein | 1 | 20 | 2.89E-02 | 0.005 | IL24 |  |
| Olfactory Bulb Interneuron Development | 1 | 20 | 2.89E-02 | 0.005 | ROBO1 |  |
| Protein Localization To Juxtaparanode Region Of Axon | 1 | 20 | 2.89E-02 | 0.005 | EPB41L3 |  |
| Regulation Of Estradiol Secretion | 1 | 20 | 2.89E-02 | 0.005 | IL1B |  |
| Positive Regulation Of Sodium:Proton Antiporter Activity | 1 | 20 | 2.89E-02 | 0.005 | PLCB1 |  |
| Cellular Response To Muramyl Dipeptide | 1 | 20 | 2.89E-02 | 0.005 | PTPN22 |  |
| Positive Regulation Of Immature T Cell Proliferation | 1 | 20 | 2.89E-02 | 0.005 | IL1B |  |
| Positive Regulation Of Notch Signaling Pathway Involved In Heart Induction | 1 | 20 | 2.89E-02 | 0.005 | ROBO1 |  |
| Negative Regulation Of Arp2/3 Complex-Mediated Actin Nucleation | 1 | 20 | 2.89E-02 | 0.005 | GMFG |  |
| Negative Regulation Of Natural Killer Cell Chemotaxis | 1 | 20 | 2.89E-02 | 0.005 | CCL2 |  |
| Response To Vasopressin | 1 | 20 | 2.89E-02 | 0.005 | PLCB1 |  |
| Regulation Of Myofibroblast Differentiation | 1 | 20 | 2.89E-02 | 0.005 | PDCD4 |  |
| Regulation Of Ovulation | 1 | 20 | 2.89E-02 | 0.005 | PLAU |  |
| Positive Regulation Of Cd8-Positive, Alpha-Beta T Cell Proliferation | 1 | 20 | 2.89E-02 | 0.005 | PTPN22 |  |
| Trigeminal Ganglion Development | 1 | 20 | 2.89E-02 | 0.005 | NRP2 |  |
| Regulation Of Synapse Organization | 4 | 2 | 2.91E-02 | 0.011 | SLITRK1, FLRT2, EPHA7, ADGRL3 |  |
| Cytokine Production | 3 | 2 | 2.94E-02 | 0.010 | IL1B, S1PR3, PIK3CG |  |
| Response To Endogenous Stimulus | 16 | 0 | 2.96E-02 | 0.009 | PDE3B, PIK3CG, PLAU, CCL2, CCND2, PLCB1, PTPN22, EPHA3, GRB14, GJA1, IL1B, HMGCS1, FOXA1, MMP3, IL1RL1, BCL11A |  |
| Positive Regulation Of Acute Inflammatory Response | 2 | 4 | 2.97E-02 | 0.009 | IL1B, PIK3CG |  |
| Regulation Of Neutrophil Migration | 2 | 4 | 2.97E-02 | 0.009 | IL1B, NCKAP1L |  |
| Regulation Of P38Mapk Cascade | 2 | 4 | 2.97E-02 | 0.009 | PTPN22, IL1B |  |
| Negative Regulation Of Cell Morphogenesis Involved In Differentiation | 3 | 2 | 3.00E-02 | 0.010 | FGF13, BCL11A, EPHA7 |  |
| Regulation Of Vasoconstriction | 3 | 2 | 3.00E-02 | 0.010 | CHRM3, TBXAS1, GJA1 |  |
| Phototransduction | 2 | 4 | 3.09E-02 | 0.009 | PLCB4, GNGT2 |  |
| Negative Regulation Of Cellular Response To Insulin Stimulus | 2 | 4 | 3.09E-02 | 0.009 | GRB14, IL1B |  |
| Regulation Of Establishment Of Protein Localization | 10 | 1 | 3.09E-02 | 0.010 | PKIA, PDE3B, GJA1, IL1B, CARD8, IL1RL1, CYP2J2, PTPN22, STOM, NEO1 |  |
| Leukocyte Activation | 11 | 1 | 3.10E-02 | 0.009 | NCKAP1L, BCL2A1, CXCR4, PIK3CG, PLAU, CD33, DOCK10, GMFG, BCL11A, PTPN22, STOM |  |
| Regulation Of Cellular Localization | 11 | 1 | 3.16E-02 | 0.009 | PCDH17, PKIA, PDE3B, MCTP2, IL1B, IL13RA2, CLIC2, DMD, PTPN22, STOM, EPHA3 |  |
| Phosphorus Metabolic Process | 21 | 0 | 3.17E-02 | 0.008 | PDE3B, PIK3CG, PLAU, PLCB4, ENTPD3, CCL2, LAMA1, IL24, IRAK3, PIK3AP1, PLCB1, FGF13, PTPN22, EPHA7, EPHA3, IL1B, HMGCS1, HDDC2, GMFG, NRK, SHC3 |  |
| Negative Regulation Of Cysteine-Type Endopeptidase Activity | 3 | 2 | 3.20E-02 | 0.010 | CARD8, BCL2A1, IL24 |  |
| Cellular Response To Monosaccharide Stimulus | 3 | 2 | 3.20E-02 | 0.010 | IL1B, PLCB1, PLAU |  |
| Regulation Of Proteolysis | 11 | 1 | 3.22E-02 | 0.009 | BCL2A1, TFPI2, ROBO1, IL1B, CARD8, HMSD, IL24, SERPINB7, CRADD, SERPINB2, EPHA7 |  |
| Leukocyte Migration | 6 | 1 | 3.26E-02 | 0.010 | GRB14, NCKAP1L, CXCR4, PIK3CG, IL1B, CCL2 |  |
| Cellular Component Organization | 47 | 0 | 3.27E-02 | 0.007 | EFHC1, SLITRK1, DMKN, SYTL3, DMD, FGF13, STOM, EPHA3, FAM172BP, NCKAP1L, GJA1, TTLL11, ZDHHC15, TOR1AIP2, FOXA1, MMP3, NRK, KISS1, LAMA4, PLTP, ROBO1, CCL2, LAMA1, TCF4, HIST2H4B, SRPX, CXCR4, GPR65, GMFG, EPB41L3, ADGRL3, PCDHB5, FLRT2, ABI3BP, PCDH17, MAFIP, GDAP1, PCDHB6, DOCK10, HRASLS, ATP8B2, PCDHB2, PRDM9, PRDM8, FKBP10, PCDHB16, POF1B |  |
| Positive Regulation Of Endopeptidase Activity | 4 | 1 | 3.30E-02 | 0.010 | ROBO1, CARD8, IL24, CRADD |  |
| Cell Activation | 12 | 1 | 3.31E-02 | 0.009 | NCKAP1L, BCL2A1, CXCR4, PIK3CG, PLAU, CD33, DOCK10, GMFG, TCF4, BCL11A, PTPN22, STOM |  |
| Regulation Of Triglyceride Metabolic Process | 2 | 4 | 3.33E-02 | 0.009 | AADAC, PIK3CG |  |
| Positive Regulation Of Vasoconstriction | 2 | 4 | 3.33E-02 | 0.009 | GJA1, TBXAS1 |  |
| Regulation Of Neuron Migration | 2 | 4 | 3.33E-02 | 0.009 | FLRT2, NEO1 |  |
| Negative Regulation Of Microtubule Polymerization Or Depolymerization | 2 | 4 | 3.33E-02 | 0.009 | FGF13, NAV3 |  |
| Positive Regulation Of G1/S Transition Of Mitotic Cell Cycle | 2 | 4 | 3.33E-02 | 0.009 | PLCB1, CCND2 |  |
| Cellular Response To Hormone Stimulus | 7 | 1 | 3.34E-02 | 0.010 | GRB14, PDE3B, GJA1, PLCB1, HMGCS1, EPHA3, CCND2 |  |
| Anatomical Structure Maturation | 3 | 2 | 3.34E-02 | 0.010 | FOXA1, GJA1, PLCB1 |  |
| Regulation Of G1/S Transition Of Mitotic Cell Cycle | 4 | 1 | 3.35E-02 | 0.010 | CCL2, PLCB1, CCND2, CRADD |  |
| Biological Regulation | 106 | 0 | 3.38E-02 | 0.006 | ARHGDIB, BCL2A1, AADAC, CD33, CHRM3, CLIC2, CCND2, DMD, CYP2J2, FGF13, S1PR3, STOM, EPHA7, EPHA3, GRB14, NCKAP1L, GJA1, GNGT2, IL1B, IL13RA2, HMGCS1, FOXA1, MMP3, KCNQ3, KISS1, LAMA4, SERPINB2, NEO1, PKIA, PDE3B, PIK3CG, PLAU, PLCB4, PLXNA2, PLTP, ROBO1, CCL2, RGS10, TBXAS1, TCF4, SLC22A3, HIST2H4B, SRPX, CXCR4, TFPI2, NRP2, GPR65, LGR5, SERPINB7, CRADD, GMFG, IL1RL1, RAPGEF5, CLEC2B, CNKSR2, CARD8, IL24, ESM1, IRAK3, SP140, EPB41L3, PLCB1, ADGRL3, TBC1D30, PTPN22, PITPNC1, FLRT2, ABI3BP, PDCD4, PCDH17, PCDH11X, OR2H1, STXBP6, G0S2, BCL11A, SHC3, MCTP2, LMO3, DOCK10, HRASLS, ATP8B2, PRDM9, PRDM8, SAMSN1, GPR135, ANKRD30A, ARRDC4, NAV3, EFHC1, SLITRK1, SYTL3, OR2M3, PIK3AP1, FOXR2, FAM172BP, TOR1AIP2, NRK, OR5B21, ZNF438, FAM83B, SSX8, LAMA1, HMSD, SAMD5, ZNF716, MAFIP |  |
| Regulation Of Transmembrane Transporter Activity | 5 | 1 | 3.41E-02 | 0.010 | DMD, GJA1, PLCB1, CLIC2, STOM |  |
| Response To Anesthetic | 3 | 2 | 3.41E-02 | 0.010 | IL1B, CXCR4, ADGRL3 |  |
| Adenylate Cyclase-Modulating G-Protein Coupled Receptor Signaling Pathway | 4 | 1 | 3.45E-02 | 0.010 | CHRM3, S1PR3, GPR65, LGR5 |  |
| Positive Regulation Of Cell-Cell Adhesion | 5 | 1 | 3.45E-02 | 0.010 | NCKAP1L, PTPN22, IL1B, CCL2, FOXA1 |  |
| Cellular Response To Staurosporine | 1 | 16 | 3.46E-02 | 0.005 | PLAU |  |
| Response To Staurosporine | 1 | 16 | 3.46E-02 | 0.005 | PLAU |  |
| Phagolysosome Assembly | 1 | 16 | 3.46E-02 | 0.005 | SRPX |  |
| Regulation Of T-Helper 1 Cell Cytokine Production | 1 | 16 | 3.46E-02 | 0.005 | IL1B |  |
| Positive Regulation Of T-Helper 1 Cell Cytokine Production | 1 | 16 | 3.46E-02 | 0.005 | IL1B |  |
| Oocyte Differentiation | 1 | 16 | 3.46E-02 | 0.005 | LGR5 |  |
| Negative Regulation Of Lymphocyte Chemotaxis | 1 | 16 | 3.46E-02 | 0.005 | CCL2 |  |
| Dump Biosynthetic Process | 1 | 16 | 3.46E-02 | 0.005 | HDDC2 |  |
| Pyrimidine Nucleotide Salvage | 1 | 16 | 3.46E-02 | 0.005 | HDDC2 |  |
| Farnesyl Diphosphate Metabolic Process | 1 | 16 | 3.46E-02 | 0.005 | HMGCS1 |  |
| Positive Regulation Of Vascular Endothelial Growth Factor Signaling Pathway | 1 | 16 | 3.46E-02 | 0.005 | ROBO1 |  |
| Regulation Of Skeletal Muscle Contraction By Regulation Of Release Of Sequestered Calcium Ion | 1 | 16 | 3.46E-02 | 0.005 | DMD |  |
| Negative Regulation Of Neuron Maturation | 1 | 16 | 3.46E-02 | 0.005 | BCL11A |  |
| Hypothalamus Cell Migration | 1 | 16 | 3.46E-02 | 0.005 | NRP2 |  |
| Hematopoietic Stem Cell Migration | 1 | 16 | 3.46E-02 | 0.005 | CXCR4 |  |
| Positive Regulation Of Heart Induction | 1 | 16 | 3.46E-02 | 0.005 | ROBO1 |  |
| Positive Regulation Of Vascular Wound Healing | 1 | 16 | 3.46E-02 | 0.005 | CXCR4 |  |
| Negative Regulation Of Trophoblast Cell Migration | 1 | 16 | 3.46E-02 | 0.005 | ARHGDIB |  |
| Positive Regulation Of Interferon-Beta Secretion | 1 | 16 | 3.46E-02 | 0.005 | PTPN22 |  |
| Positive Regulation Of Transforming Growth Factor Beta1 Production | 1 | 16 | 3.46E-02 | 0.005 | SERPINB7 |  |
| Negative Regulation Of Myosin-Light-Chain-Phosphatase Activity | 1 | 16 | 3.46E-02 | 0.005 | NCKAP1L |  |
| Regulation Of Toll-Like Receptor 7 Signaling Pathway | 1 | 16 | 3.46E-02 | 0.005 | PTPN22 |  |
| Negative Regulation Of Oocyte Maturation | 1 | 16 | 3.46E-02 | 0.005 | GRB14 |  |
| Regulation Of Cd8-Positive, Alpha-Beta T Cell Differentiation | 1 | 16 | 3.46E-02 | 0.005 | NCKAP1L |  |
| Regulation Of Adiponectin Secretion | 1 | 16 | 3.46E-02 | 0.005 | IL1B |  |
| Positive Regulation Of Cell Adhesion Molecule Production | 1 | 16 | 3.46E-02 | 0.005 | IL1B |  |
| Cranial Ganglion Development | 1 | 16 | 3.46E-02 | 0.005 | NRP2 |  |
| Positive Regulation Of Glial Cell Differentiation | 2 | 3 | 3.46E-02 | 0.009 | IL1B, CXCR4 |  |
| Positive Regulation Of Establishment Of Protein Localization | 7 | 1 | 3.55E-02 | 0.010 | IL1RL1, GJA1, CYP2J2, PTPN22, IL1B, CARD8, STOM |  |
| Regulation Of Growth | 10 | 1 | 3.55E-02 | 0.009 | GJA1, CXCR4, ESM1, HRASLS, NRK, EPB41L3, PLCB1, FGF13, BCL11A, EPHA7 |  |
| Negative Regulation Of Adaptive Immune Response | 2 | 3 | 3.59E-02 | 0.009 | IL1RL1, SAMSN1 |  |
| Cellular Response To Ammonium Ion | 2 | 3 | 3.59E-02 | 0.009 | KCNQ3, PLAU |  |
| Regulation Of Defense Response To Virus By Host | 2 | 3 | 3.59E-02 | 0.009 | PTPN22, IL1B |  |
| Regulation Of Cell-Matrix Adhesion | 3 | 2 | 3.63E-02 | 0.010 | DMD, EPHA3, PLAU |  |
| Exocytosis | 9 | 1 | 3.64E-02 | 0.010 | NCKAP1L, PIK3CG, PLAU, CD33, STXBP6, CCL2, SYTL3, GMFG, STOM |  |
| Cellular Response To Lipid | 9 | 1 | 3.64E-02 | 0.010 | PDCD4, PLAU, IL1B, CCL2, IL24, HMGCS1, PLCB1, GDAP1, EPHA3 |  |
| Extracellular Structure Organization | 6 | 1 | 3.64E-02 | 0.010 | MMP3, LAMA4, PLTP, LAMA1, FLRT2, ABI3BP |  |
| Epithelial Tube Morphogenesis | 3 | 2 | 3.71E-02 | 0.010 | EPHA7, GJA1, CXCR4 |  |
| Regulation Of Granulocyte Chemotaxis | 2 | 3 | 3.72E-02 | 0.009 | IL1B, NCKAP1L |  |
| Response To Vitamin D | 2 | 3 | 3.72E-02 | 0.009 | IL1B, GDAP1 |  |
| Regulation Of Cellular Response To Stress | 9 | 1 | 3.74E-02 | 0.010 | PDCD4, IL1B, SAMD5, MMP3, NRK, KISS1, PLCB1, PTPN22, NEO1 |  |
| Regulation Of Leukocyte Chemotaxis | 3 | 2 | 3.78E-02 | 0.010 | IL1B, NCKAP1L, CCL2 |  |
| Muscle System Process | 5 | 1 | 3.79E-02 | 0.010 | DMD, GJA1, CXCR4, IL1B, CHRM3 |  |
| Positive Regulation Of Cytosolic Calcium Ion Concentration Involved In Phospholipase C-Activating G-Protein Coupled Signaling Pathway | 2 | 3 | 3.85E-02 | 0.009 | GPR65, KISS1 |  |
| Response To Ph | 2 | 3 | 3.85E-02 | 0.009 | GPR65, GJA1 |  |
| Regulation Of Endothelial Cell Differentiation | 2 | 3 | 3.85E-02 | 0.009 | S1PR3, IL1B |  |
| T Cell Proliferation | 2 | 3 | 3.85E-02 | 0.009 | CXCR4, PIK3CG |  |
| Positive Regulation Of T Cell Proliferation | 3 | 2 | 3.86E-02 | 0.010 | PTPN22, IL1B, NCKAP1L |  |
| Regulation Of Transporter Activity | 5 | 1 | 3.96E-02 | 0.010 | DMD, GJA1, PLCB1, CLIC2, STOM |  |
| Positive Regulation Of Glomerular Mesangial Cell Proliferation | 1 | 14 | 4.02E-02 | 0.005 | SERPINB7 |  |
| Establishment Of Blood-Nerve Barrier | 1 | 14 | 4.02E-02 | 0.005 | DMD |  |
| Interleukin-6 Production | 1 | 14 | 4.02E-02 | 0.005 | IL1B |  |
| Toll-Like Receptor 2 Signaling Pathway | 1 | 14 | 4.02E-02 | 0.005 | PIK3AP1 |  |
| Negative Regulation Of T-Helper 1 Type Immune Response | 1 | 14 | 4.02E-02 | 0.005 | IL1RL1 |  |
| Negative Regulation Of Mononuclear Cell Migration | 1 | 14 | 4.02E-02 | 0.005 | PLCB1 |  |
| Endocardial Cushion Formation | 1 | 14 | 4.02E-02 | 0.005 | ROBO1 |  |
| Glycosylceramide Catabolic Process | 1 | 14 | 4.02E-02 | 0.005 | GALC |  |
| Paranodal Junction Assembly | 1 | 14 | 4.02E-02 | 0.005 | EPB41L3 |  |
| Axon Choice Point Recognition | 1 | 14 | 4.02E-02 | 0.005 | ROBO1 |  |
| Epithelial-Mesenchymal Cell Signaling | 1 | 14 | 4.02E-02 | 0.005 | FOXA1 |  |
| Saliva Secretion | 1 | 14 | 4.02E-02 | 0.005 | CHRM3 |  |
| Regulation Of Sodium:Proton Antiporter Activity | 1 | 14 | 4.02E-02 | 0.005 | PLCB1 |  |
| Negative Regulation Of Oogenesis | 1 | 14 | 4.02E-02 | 0.005 | GRB14 |  |
| Response To Ionomycin | 1 | 14 | 4.02E-02 | 0.005 | PLCB1 |  |
| Regulation Of Skeletal Muscle Contraction By Calcium Ion Signaling | 1 | 14 | 4.02E-02 | 0.005 | DMD |  |
| Fusion Of Virus Membrane With Host Plasma Membrane | 1 | 14 | 4.02E-02 | 0.005 | CXCR4 |  |
| Cerebral Cortex Tangential Migration | 1 | 14 | 4.02E-02 | 0.005 | NRP2 |  |
| Corticospinal Tract Morphogenesis | 1 | 14 | 4.02E-02 | 0.005 | PRDM8 |  |
| Positive Regulation Of Behavioral Fear Response | 1 | 14 | 4.02E-02 | 0.005 | GJA1 |  |
| Negative Regulation Of Peptidyl-Cysteine S-Nitrosylation | 1 | 14 | 4.02E-02 | 0.005 | DMD |  |
| Cellular Response To Cell-Matrix Adhesion | 1 | 14 | 4.02E-02 | 0.005 | MMP3 |  |
| Regulation Of Calcidiol 1-Monooxygenase Activity | 1 | 14 | 4.02E-02 | 0.005 | IL1B |  |
| Regulation Of Interferon-Beta Secretion | 1 | 14 | 4.02E-02 | 0.005 | PTPN22 |  |
| Negative Regulation Of Mammary Gland Epithelial Cell Proliferation | 1 | 14 | 4.02E-02 | 0.005 | ROBO1 |  |
| Positive Regulation Of Glucocorticoid Receptor Signaling Pathway | 1 | 14 | 4.02E-02 | 0.005 | LMO3 |  |
| Negative Regulation Of Actin Nucleation | 1 | 14 | 4.02E-02 | 0.005 | GMFG |  |
| Membrane Fusion Involved In Viral Entry Into Host Cell | 1 | 14 | 4.02E-02 | 0.005 | CXCR4 |  |
| Positive Regulation Of Fear Response | 1 | 14 | 4.02E-02 | 0.005 | GJA1 |  |
| Negative Regulation Of Synapse Assembly | 1 | 14 | 4.02E-02 | 0.005 | EPHA7 |  |
| Cellular Response To Ionomycin | 1 | 14 | 4.02E-02 | 0.005 | PLCB1 |  |
| Negative Regulation Of Oocyte Development | 1 | 14 | 4.02E-02 | 0.005 | GRB14 |  |
| Positive Regulation Of Gamma-Delta T Cell Differentiation | 1 | 14 | 4.02E-02 | 0.005 | NCKAP1L |  |
| Positive Regulation Of Meiosis I | 1 | 14 | 4.02E-02 | 0.005 | PRDM9 |  |
| Regulation Of Cd8-Positive, Alpha-Beta T Cell Proliferation | 1 | 14 | 4.02E-02 | 0.005 | PTPN22 |  |
| Multi-Organism Membrane Fusion | 1 | 14 | 4.02E-02 | 0.005 | CXCR4 |  |
| Retinal Blood Vessel Morphogenesis | 1 | 14 | 4.02E-02 | 0.005 | LAMA1 |  |
| Regulation Of Nucleotide-Binding Oligomerization Domain Containing 2 Signaling Pathway | 1 | 14 | 4.02E-02 | 0.005 | PTPN22 |  |
| Mammary Duct Terminal End Bud Growth | 1 | 14 | 4.02E-02 | 0.005 | ROBO1 |  |
| Regulation Of Interferon-Gamma Production | 3 | 2 | 4.09E-02 | 0.010 | PTPN22, IL1B, IL1RL1 |  |
| Response To Acid Chemical | 8 | 1 | 4.09E-02 | 0.010 | GJA1, IL1B, MMP3, TBXAS1, PLCB1, BCL11A, GDAP1, EPHA3 |  |
| Negative Regulation Of Metabolic Process | 32 | 0 | 4.11E-02 | 0.008 | BCL2A1, CARD8, IL24, IRAK3, DMD, PLCB1, CYP2J2, PTPN22, FAM172BP, PDCD4, PCDH11X, NCKAP1L, GJA1, IL1B, FOXA1, MMP3, BCL11A, SERPINB2, ZNF438, PKIA, PDE3B, PIK3CG, ROBO1, LMO3, HMSD, TCF4, PRDM8, SAMSN1, HIST2H4B, TFPI2, SERPINB7, GMFG |  |
| Lymphocyte Chemotaxis | 2 | 3 | 4.12E-02 | 0.008 | PIK3CG, CCL2 |  |
| Regulation Of Calcium Ion Import | 2 | 3 | 4.12E-02 | 0.008 | CXCR4, CCL2 |  |
| Negative Regulation Of Stress-Activated Mapk Cascade | 2 | 3 | 4.12E-02 | 0.008 | PDCD4, PTPN22 |  |
| Negative Regulation Of Stress-Activated Protein Kinase Signaling Cascade | 2 | 3 | 4.12E-02 | 0.008 | PDCD4, PTPN22 |  |
| Cellular Response To Carbohydrate Stimulus | 3 | 2 | 4.16E-02 | 0.010 | IL1B, PLCB1, PLAU |  |
| Response To Fatty Acid | 3 | 2 | 4.24E-02 | 0.010 | IL1B, TBXAS1, PLCB1 |  |
| Epidermal Growth Factor Receptor Signaling Pathway | 2 | 3 | 4.25E-02 | 0.008 | SHC3, FAM83B |  |
| Regulation Of Collagen Biosynthetic Process | 2 | 3 | 4.25E-02 | 0.008 | SERPINB7, CYP2J2 |  |
| Regulation Of Extrinsic Apoptotic Signaling Pathway In Absence Of Ligand | 2 | 3 | 4.25E-02 | 0.008 | IL1B, SRPX |  |
| Positive Regulation Of Peptidase Activity | 4 | 1 | 4.27E-02 | 0.010 | ROBO1, CARD8, IL24, CRADD |  |
| Developmental Maturation | 4 | 1 | 4.27E-02 | 0.010 | ZDHHC15, FOXA1, GJA1, PLCB1 |  |
| Negative Regulation Of Catabolic Process | 5 | 1 | 4.28E-02 | 0.010 | PDE3B, PIK3CG, PTPN22, IL1B, IRAK3 |  |
| Positive Regulation Of Cell Cycle Process | 5 | 1 | 4.28E-02 | 0.010 | PLCB1, PRDM9, IL1B, CCND2, CRADD |  |
| Regulation Of Vesicle-Mediated Transport | 7 | 1 | 4.30E-02 | 0.010 | NCKAP1L, IL1B, IL13RA2, STXBP6, CCL2, EPHA3, SYTL3 |  |
| Regulation Of Protein Complex Disassembly | 3 | 2 | 4.32E-02 | 0.010 | FGF13, NAV3, IRAK3 |  |
| Negative Regulation Of Cell Growth | 4 | 1 | 4.33E-02 | 0.010 | FGF13, BCL11A, EPHA7, GJA1 |  |
| Cytoskeleton Organization | 11 | 1 | 4.44E-02 | 0.009 | NCKAP1L, EFHC1, TTLL11, POF1B, CCL2, GPR65, DMD, NRK, KISS1, EPB41L3, FGF13 |  |
| Regulation Of Extent Of Cell Growth | 3 | 2 | 4.49E-02 | 0.010 | FGF13, BCL11A, EPHA7 |  |
| Inflammatory Response | 7 | 1 | 4.51E-02 | 0.010 | GJA1, CXCR4, PIK3CG, IL1B, CCL2, S1PR3, EPHA3 |  |
| Regulation Of Cellular Response To Growth Factor Stimulus | 5 | 1 | 4.52E-02 | 0.010 | DMD, TCF4, ROBO1, IL1B, NEO1 |  |
| Regulation Of Lipid Metabolic Process | 6 | 1 | 4.52E-02 | 0.010 | AADAC, PDE3B, PIK3CG, G0S2, IL1B, HMGCS1 |  |
| Response To Morphine | 2 | 3 | 4.53E-02 | 0.008 | IL1B, CXCR4 |  |
| Cardiac Muscle Contraction | 2 | 3 | 4.53E-02 | 0.008 | DMD, CXCR4 |  |
| Negative Regulation Of Blood Coagulation | 2 | 3 | 4.53E-02 | 0.008 | SERPINB2, PLAU |  |
| Negative Regulation Of Hemostasis | 2 | 3 | 4.53E-02 | 0.008 | SERPINB2, PLAU |  |
| Neutrophil Extravasation | 1 | 12 | 4.58E-02 | 0.005 | PIK3CG |  |
| Neural Crest Cell Migration Involved In Autonomic Nervous System Development | 1 | 12 | 4.58E-02 | 0.005 | NRP2 |  |
| Interleukin-1 Production | 1 | 12 | 4.58E-02 | 0.005 | IL1B |  |
| Respiratory Burst Involved In Defense Response | 1 | 12 | 4.58E-02 | 0.005 | PIK3CG |  |
| Negative Regulation Of Mast Cell Degranulation | 1 | 12 | 4.58E-02 | 0.005 | IL13RA2 |  |
| Negative Regulation Of Cellular Extravasation | 1 | 12 | 4.58E-02 | 0.005 | PLCB1 |  |
| Regulation Of Heart Induction | 1 | 12 | 4.58E-02 | 0.005 | ROBO1 |  |
| Hyaluronan Biosynthetic Process | 1 | 12 | 4.58E-02 | 0.005 | IL1B |  |
| Galactosylceramide Metabolic Process | 1 | 12 | 4.58E-02 | 0.005 | GALC |  |
| Fat-Soluble Vitamin Biosynthetic Process | 1 | 12 | 4.58E-02 | 0.005 | PLTP |  |
| Gap Junction Assembly | 1 | 12 | 4.58E-02 | 0.005 | GJA1 |  |
| Adenylate Cyclase-Inhibiting G-Protein Coupled Acetylcholine Receptor Signaling Pathway | 1 | 12 | 4.58E-02 | 0.005 | CHRM3 |  |
| Pyrimidine Deoxyribonucleoside Monophosphate Biosynthetic Process | 1 | 12 | 4.58E-02 | 0.005 | HDDC2 |  |
| Dump Metabolic Process | 1 | 12 | 4.58E-02 | 0.005 | HDDC2 |  |
| Positive Regulation Of Cell-Cell Adhesion Mediated By Cadherin | 1 | 12 | 4.58E-02 | 0.005 | FOXA1 |  |
| Positive Regulation Of Gonadotropin Secretion | 1 | 12 | 4.58E-02 | 0.005 | KISS1 |  |
| Negative Regulation Of Interleukin-8 Secretion | 1 | 12 | 4.58E-02 | 0.005 | PTPN22 |  |
| Negative Regulation Of P38Mapk Cascade | 1 | 12 | 4.58E-02 | 0.005 | PTPN22 |  |
| Interleukin-1 Beta Production | 1 | 12 | 4.58E-02 | 0.005 | IL1B |  |
| Positive Regulation Of Toll-Like Receptor 3 Signaling Pathway | 1 | 12 | 4.58E-02 | 0.005 | PTPN22 |  |
| Positive Regulation Of Toll-Like Receptor 9 Signaling Pathway | 1 | 12 | 4.58E-02 | 0.005 | PTPN22 |  |
| Response To Odorant | 1 | 12 | 4.58E-02 | 0.005 | FGF13 |  |
| Neurotransmitter Receptor Metabolic Process | 1 | 12 | 4.58E-02 | 0.005 | DMD |  |
| Multi-Organism Membrane Organization | 1 | 12 | 4.58E-02 | 0.005 | CXCR4 |  |
| Actin Polymerization-Dependent Cell Motility | 1 | 12 | 4.58E-02 | 0.005 | NCKAP1L |  |
| Positive Regulation Of Apoptotic Cell Clearance | 1 | 12 | 4.58E-02 | 0.005 | CCL2 |  |
| Establishment Of Blood-Brain Barrier | 1 | 12 | 4.58E-02 | 0.005 | DMD |  |
| Regulation Of Cell-Substrate Adhesion | 4 | 1 | 4.63E-02 | 0.010 | DMD, EPHA3, ABI3BP, PLAU |  |
| Regulation Of Cell Cycle G1/S Phase Transition | 4 | 1 | 4.63E-02 | 0.010 | CCL2, PLCB1, CCND2, CRADD |  |
| Glycosphingolipid Metabolic Process | 2 | 3 | 4.67E-02 | 0.008 | GALC, B4GALT6 |  |
| Regulation Of Endocrine Process | 2 | 3 | 4.67E-02 | 0.008 | IL1B, KISS1 |  |
| Response To Purine-Containing Compound | 4 | 1 | 4.75E-02 | 0.010 | IL1B, HMGCS1, PIK3CG, CCND2 |  |
| Lymphocyte Activation | 6 | 1 | 4.78E-02 | 0.010 | BCL2A1, CXCR4, PIK3CG, BCL11A, PTPN22, DOCK10 |  |
| Inositol Phosphate Metabolic Process | 2 | 3 | 4.82E-02 | 0.008 | PLCB1, PLCB4 |  |
| Response To Isoquinoline Alkaloid | 2 | 3 | 4.82E-02 | 0.008 | IL1B, CXCR4 |  |
| Positive Regulation Of Cell Cycle G1/S Phase Transition | 2 | 3 | 4.82E-02 | 0.008 | PLCB1, CCND2 |  |
| Cellular Response To Insulin Stimulus | 3 | 2 | 4.82E-02 | 0.009 | GRB14, PDE3B, CCND2 |  |
| Positive Regulation Of Secretion | 7 | 1 | 4.84E-02 | 0.009 | IL1RL1, KISS1, GJA1, CYP2J2, PTPN22, IL1B, CARD8 |  |
| Mapk Cascade | 5 | 1 | 4.87E-02 | 0.010 | NRK, FGF13, SHC3, IL1B, CCL2 |  |
| Learning Or Memory | 5 | 1 | 4.92E-02 | 0.010 | PLCB1, FGF13, SHC3, IL1B, CCND2 |  |
| Acute-Phase Response | 2 | 3 | 4.96E-02 | 0.008 | IL1B, EPHA3 |  |
| Cortical Cytoskeleton Organization | 2 | 3 | 4.96E-02 | 0.008 | NCKAP1L, EPB41L3 |  |
| Negative Regulation Of Tumor Necrosis Factor Production | 2 | 3 | 4.96E-02 | 0.008 | PTPN22, IRAK3 |  |
| Positive Regulation Of Ion Transport | 5 | 1 | 5.02E-02 | 0.010 | DMD, PLCB1, CXCR4, IL1B, CCL2 |  |
| Peptidyl-Amino Acid Modification | 10 | 1 | 5.05E-02 | 0.009 | TTLL11, ZDHHC15, IL24, PRDM9, PRDM8, FKBP10, BCL11A, SHC3, EPHA7, EPHA3 |  |
| T Cell Differentiation | 3 | 2 | 5.08E-02 | 0.009 | BCL11A, PTPN22, BCL2A1 |  |
| Regulation Of Epithelial Cell Proliferation | 6 | 1 | 5.08E-02 | 0.010 | GJA1, PLAU, ROBO1, NRP2, CCL2, CCND2 |  |
| Regulation Of Interleukin-12 Production | 2 | 3 | 5.11E-02 | 0.008 | PLCB1, IRAK3 |  |
| Cardiac Septum Development | 2 | 3 | 5.11E-02 | 0.008 | ROBO1, CXCR4 |  |
| Regulation Of Collagen Metabolic Process | 2 | 3 | 5.11E-02 | 0.008 | SERPINB7, CYP2J2 |  |
| Negative Regulation Of Coagulation | 2 | 3 | 5.11E-02 | 0.008 | SERPINB2, PLAU |  |
| Regulation Of Chemokine-Mediated Signaling Pathway | 1 | 11 | 5.14E-02 | 0.005 | ROBO1 |  |
| Ectopic Germ Cell Programmed Cell Death | 1 | 11 | 5.14E-02 | 0.005 | IL1B |  |
| Endothelium Development | 1 | 11 | 5.14E-02 | 0.005 | GJA1 |  |
| Regulation Of Ventricular Cardiac Muscle Cell Membrane Depolarization | 1 | 11 | 5.14E-02 | 0.005 | GJA1 |  |
| Galactolipid Metabolic Process | 1 | 11 | 5.14E-02 | 0.005 | GALC |  |
| Roundabout Signaling Pathway | 1 | 11 | 5.14E-02 | 0.005 | ROBO1 |  |
| Astrocyte Cell Migration | 1 | 11 | 5.14E-02 | 0.005 | CCL2 |  |
| Positive Regulation Of Cell Proliferation Involved In Kidney Development | 1 | 11 | 5.14E-02 | 0.005 | SERPINB7 |  |
| Pyrimidine Deoxyribonucleoside Monophosphate Metabolic Process | 1 | 11 | 5.14E-02 | 0.005 | HDDC2 |  |
| Positive Regulation Of Triglyceride Catabolic Process | 1 | 11 | 5.14E-02 | 0.005 | AADAC |  |
| Negative Regulation Of Glutamate Secretion | 1 | 11 | 5.14E-02 | 0.005 | IL1B |  |
| Protein Localization To Nuclear Envelope | 1 | 11 | 5.14E-02 | 0.005 | TOR1AIP2 |  |
| Regulation Of Cardiac Muscle Cell Action Potential Involved In Regulation Of Contraction | 1 | 11 | 5.14E-02 | 0.005 | FGF13 |  |
| Cellular Chloride Ion Homeostasis | 1 | 11 | 5.14E-02 | 0.005 | TBXAS1 |  |
| Regulation Of Vitamin D Biosynthetic Process | 1 | 11 | 5.14E-02 | 0.005 | IL1B |  |
| Locomotion Involved In Locomotory Behavior | 1 | 11 | 5.14E-02 | 0.005 | ADGRL3 |  |
| Regulation Of Luteinizing Hormone Secretion | 1 | 11 | 5.14E-02 | 0.005 | KISS1 |  |
| Cellular Response To L-Glutamate | 1 | 11 | 5.14E-02 | 0.005 | BCL11A |  |
| Inositol Trisphosphate Metabolic Process | 1 | 11 | 5.14E-02 | 0.005 | PLCB1 |  |
| Regulation Of Myosin-Light-Chain-Phosphatase Activity | 1 | 11 | 5.14E-02 | 0.005 | NCKAP1L |  |
| Exocyst Localization | 1 | 11 | 5.14E-02 | 0.005 | STXBP6 |  |
| Neuron Projection Extension Involved In Neuron Projection Guidance | 1 | 11 | 5.14E-02 | 0.005 | NRP2 |  |
| Axon Extension Involved In Axon Guidance | 1 | 11 | 5.14E-02 | 0.005 | NRP2 |  |
| Negative Regulation Of Cardiac Muscle Contraction | 1 | 11 | 5.14E-02 | 0.005 | PIK3CG |  |
| Positive Regulation By Host Of Viral Genome Replication | 1 | 11 | 5.14E-02 | 0.005 | STOM |  |
| Positive Regulation Of Gamma-Delta T Cell Activation | 1 | 11 | 5.14E-02 | 0.005 | NCKAP1L |  |
| Tube Morphogenesis | 5 | 1 | 5.33E-02 | 0.010 | GJA1, CXCR4, LAMA1, FOXA1, EPHA7 |  |
| Cellular Response To Lipopolysaccharide | 5 | 1 | 5.33E-02 | 0.010 | PDCD4, PLAU, IL1B, CCL2, IL24 |  |
| Response To Monosaccharide | 4 | 1 | 5.39E-02 | 0.010 | IL1B, GJA1, PLCB1, PLAU |  |
| Arachidonic Acid Metabolic Process | 2 | 3 | 5.40E-02 | 0.008 | TBXAS1, CYP2J2 |  |
| Regulation Of Interleukin-2 Production | 2 | 3 | 5.40E-02 | 0.008 | IL1B, NAV3 |  |
| Negative Regulation Of Cellular Component Organization | 9 | 1 | 5.43E-02 | 0.009 | NAV3, IRAK3, GMFG, TBC1D30, FGF13, BCL11A, EPHA7, EPHA3, NEO1 |  |
| Regulation Of Rho Protein Signal Transduction | 3 | 2 | 5.43E-02 | 0.009 | ROBO1, ARHGDIB, GPR65 |  |
| Regulation Of Cell-Cell Adhesion | 6 | 1 | 5.44E-02 | 0.009 | NCKAP1L, PTPN22, IL1B, CCL2, FOXA1, EPHA7 |  |
| Regulation Of Endothelial Cell Apoptotic Process | 2 | 3 | 5.55E-02 | 0.008 | PDCD4, CCL2 |  |
| Positive Regulation Of Extrinsic Apoptotic Signaling Pathway | 2 | 3 | 5.55E-02 | 0.008 | SRPX, G0S2 |  |
| Regulation Of Nuclear Division | 4 | 1 | 5.66E-02 | 0.009 | IL1B, PLCB1, PRDM9, CCND2 |  |
| Small Gtpase Mediated Signal Transduction | 5 | 1 | 5.66E-02 | 0.009 | HRASLS, ARHGDIB, SHC3, RAPGEF5, DOCK10 |  |
| Activation Of Jnkk Activity | 1 | 10 | 5.69E-02 | 0.005 | NRK |  |
| Nephric Duct Morphogenesis | 1 | 10 | 5.69E-02 | 0.005 | EPHA7 |  |
| Negative Regulation Of Camp-Dependent Protein Kinase Activity | 1 | 10 | 5.69E-02 | 0.005 | PKIA |  |
| Microtubule Severing | 1 | 10 | 5.69E-02 | 0.005 | TTLL11 |  |
| Marginal Zone B Cell Differentiation | 1 | 10 | 5.69E-02 | 0.005 | DOCK10 |  |
| Positive Regulation Of Fever Generation | 1 | 10 | 5.69E-02 | 0.005 | IL1B |  |
| Negative Regulation Of Mast Cell Activation Involved In Immune Response | 1 | 10 | 5.69E-02 | 0.005 | IL13RA2 |  |
| Regulation Of Vascular Smooth Muscle Contraction | 1 | 10 | 5.69E-02 | 0.005 | CHRM3 |  |
| Regulation Of Atrial Cardiac Muscle Cell Membrane Depolarization | 1 | 10 | 5.69E-02 | 0.005 | GJA1 |  |
| Sphingosine-1-Phosphate Receptor Signaling Pathway | 1 | 10 | 5.69E-02 | 0.005 | S1PR3 |  |
| Galactose Catabolic Process | 1 | 10 | 5.69E-02 | 0.005 | PGM5 |  |
| Sphingolipid Mediated Signaling Pathway | 1 | 10 | 5.69E-02 | 0.005 | S1PR3 |  |
| Regulation Of Fear Response | 1 | 10 | 5.69E-02 | 0.005 | GJA1 |  |
| Nucleoside Diphosphate Catabolic Process | 1 | 10 | 5.69E-02 | 0.005 | ENTPD3 |  |
| Positive Regulation Of Peroxisome Proliferator Activated Receptor Signaling Pathway | 1 | 10 | 5.69E-02 | 0.005 | LMO3 |  |
| Atrial Cardiac Muscle Cell Action Potential | 1 | 10 | 5.69E-02 | 0.005 | GJA1 |  |
| Synaptic Vesicle Maturation | 1 | 10 | 5.69E-02 | 0.005 | ZDHHC15 |  |
| Corpus Callosum Morphogenesis | 1 | 10 | 5.69E-02 | 0.005 | PRDM8 |  |
| Regulation Of Snare Complex Assembly | 1 | 10 | 5.69E-02 | 0.005 | STXBP6 |  |
| Regulation Of Behavioral Fear Response | 1 | 10 | 5.69E-02 | 0.005 | GJA1 |  |
| Secretory Granule Localization | 1 | 10 | 5.69E-02 | 0.005 | PIK3CG |  |
| Regulation Of Peptidyl-Cysteine S-Nitrosylation | 1 | 10 | 5.69E-02 | 0.005 | DMD |  |
| Negative Regulation Of Interleukin-6 Secretion | 1 | 10 | 5.69E-02 | 0.005 | PTPN22 |  |
| Positive Regulation Of Interferon-Alpha Secretion | 1 | 10 | 5.69E-02 | 0.005 | PTPN22 |  |
| Positive Regulation Of Interleukin-6 Biosynthetic Process | 1 | 10 | 5.69E-02 | 0.005 | IL1B |  |
| Regulation Of Natural Killer Cell Proliferation | 1 | 10 | 5.69E-02 | 0.005 | PTPN22 |  |
| Regulation Of Transforming Growth Factor Beta1 Production | 1 | 10 | 5.69E-02 | 0.005 | SERPINB7 |  |
| Regulation Of Interferon-Alpha Secretion | 1 | 10 | 5.69E-02 | 0.005 | PTPN22 |  |
| Regulation Of Immature T Cell Proliferation In Thymus | 1 | 10 | 5.69E-02 | 0.005 | IL1B |  |
| Negative Regulation Of Glial Cell Apoptotic Process | 1 | 10 | 5.69E-02 | 0.005 | CCL2 |  |
| Regulation Of Oocyte Maturation | 1 | 10 | 5.69E-02 | 0.005 | GRB14 |  |
| Regulation Of Nucleotide-Binding Oligomerization Domain Containing Signaling Pathway | 1 | 10 | 5.69E-02 | 0.005 | PTPN22 |  |
| **Enrichment for Cellular Components** | | | | | |  |
| Integral Component Of Plasma Membrane | 25 | 1 | 6.65E-05 | 0.013 | PLXNA2, CD33, ROBO1, CHRM3, PCDHB6, PCDHB2, ADGRL3, SLC22A3, PCDHB5, S1PR3, FLRT2, PCDHB16, STOM, EPHA7, EPHA3, PCDH17, PCDH11X, NCKAP1L, GJA1, GPR65, LGR5, PCDH11Y, KCNQ3, CLEC2B, NEO1 |  |
| Membrane | 89 | 0 | 7.26E-05 | 0.008 | ARHGDIB, ARRDC4, BCL2A1, AADAC, NAV3, CD33, ENTPD3, SLITRK1, CHRM3, TLCD1, SYTL3, CCND2, DMD, OR2M3, PIK3AP1, CYP2J2, TMEM171, FGF13, S1PR3, STOM, EPHA7, EPHA3, GRB14, NCKAP1L, GJA1, LRFN5, GNGT2, ZDHHC15, IL13RA2, TOR1AIP2, SHISA3, TMEM154, KCNQ3, KISS1, METTL7B, SFTA3, SERPINB2, OR5B21, NEO1, FAM83B, CD163L1, PDE3B, PGM5, PIK3CG, PLAU, PLXNA2, ROBO1, LAMA1, TBXAS1, SLC22A3, HIST2H4B, SRPX, CXCR4, NRP2, GPR65, LGR5, AGMO, IL1RL1, B4GALT6, CLEC2B, CNKSR2, IRAK3, EPB41L3, SEL1L3, PLCB1, ADGRL3, TBC1D30, OR2M4, FLRT2, PCDH11X, OR2H1, STXBP6, SHC3, CNTN5, GDAP1, MCTP2, DOCK10, HRASLS, ATP8B2, MARCH4, FKBP10, SAMSN1, CACHD1, MPP4, GPR135, MYCT1, VEPH1, CLMP, PCDH11Y | |
| Cell-Cell Junction | 12 | 2 | 8.62E-05 | 0.017 | EPB41L3, PGM5, GJA1, CXCR4, ADGRL3, POF1B, FGF13, STXBP6, LAMA1, FLRT2, CLMP, MPP4 | |
| Intrinsic Component Of Plasma Membrane | 25 | 1 | 1.47E-04 | 0.012 | PLXNA2, CD33, ROBO1, CHRM3, PCDHB6, PCDHB2, ADGRL3, SLC22A3, PCDHB5, S1PR3, FLRT2, PCDHB16, STOM, EPHA7, EPHA3, PCDH17, PCDH11X, NCKAP1L, GJA1, GPR65, LGR5, PCDH11Y, KCNQ3, CLEC2B, NEO1 | |
| Plasma Membrane Part | 39 | 1 | 1.81E-04 | 0.010 | CD33, CNKSR2, CHRM3, SYTL3, DMD, EPB41L3, ADGRL3, FGF13, PCDHB5, PTPN22, S1PR3, FLRT2, STOM, EPHA7, EPHA3, PCDH17, PCDH11X, NCKAP1L, GJA1, GNGT2, IL13RA2, KCNQ3, KISS1, NEO1, CD163L1, PGM5, PLXNA2, ROBO1, PCDHB6, PCDHB2, SLC22A3, PCDHB16, MPP4, CXCR4, GPR65, LGR5, PCDH11Y, IL1RL1, CLEC2B | |
| Membrane Part | 77 | 0 | 4.21E-04 | 0.008 | AADAC, NAV3, CD33, ENTPD3, SLITRK1, CHRM3, TLCD1, CLIC2, SYTL3, DMD, OR2M3, CYP2J2, TMEM171, FGF13, S1PR3, STOM, EPHA7, EPHA3, FAM172BP, NCKAP1L, GJA1, LRFN5, GNGT2, ZDHHC15, IL13RA2, TOR1AIP2, SHISA3, TMEM154, KCNQ3, KISS1, METTL7B, SFTA3, OR5B21, NEO1, CD163L1, PDE3B, PGM5, PIK3CG, PLXNA2, ROBO1, TBXAS1, SLC22A3, CXCR4, NRP2, GPR65, LGR5, AGMO, IL1RL1, B4GALT6, CLEC2B, CNKSR2, EPB41L3, SEL1L3, ADGRL3, PCDHB5, PTPN22, OR2M4, FLRT2, PCDH17, PCDH11X, OR2H1, STXBP6, CNTN5, GDAP1, MCTP2, PCDHB6, HRASLS, ATP8B2, MARCH4, PCDHB2, CACHD1, PCDHB16, MPP4, GPR135, MYCT1, CLMP, PCDH11Y | |
| Lateral Plasma Membrane | 4 | 6 | 6.88E-04 | 0.016 | FGF13, DMD, GJA1, MPP4 | |
| Neuron Part | 24 | 1 | 1.01E-03 | 0.011 | PLCB4, EFHC1, ROBO1, MCTP2, CNKSR2, CHRM3, SYTL3, DOCK10, RGS10, DMD, EPB41L3, ADGRL3, FGF13, FLRT2, EPHA7, EPHA3, MPP4, CXCR4, NRP2, MMP3, KCNQ3, KISS1, BCL11A, NEO1 | |
| Extracellular Region | 29 | 1 | 1.13E-03 | 0.010 | OLFML1, CD163L1, BAGE4, PLAU, PLTP, SLITRK1, CCL2, LAMA1, DMKN, IL24, ESM1, FGF13, FLRT2, EPHA3, ABI3BP, HIST2H4B, TFPI2, NRP2, IL1B, IL13RA2, GMFG, CRISPLD1, MMP3, IL1RL1, KISS1, LAMA4, CNTN5, SFTA3, SERPINB2 | |
| Semaphorin Receptor Complex | 2 | 18 | 1.92E-03 | 0.010 | NRP2, PLXNA2 | |
| Plasma Membrane Bounded Cell Projection | 23 | 1 | 2.53E-03 | 0.010 | PLCB4, EFHC1, ROBO1, CNKSR2, CHRM3, DOCK10, RGS10, DMD, ADGRL3, TBC1D30, FGF13, SAMSN1, FLRT2, EPHA7, EPHA3, MPP4, TTLL11, NRP2, FOXA1, MAFIP, MMP3, KCNQ3, KISS1 | |
| Integral Component Of Membrane | 60 | 0 | 4.25E-03 | 0.008 | AADAC, CD33, ENTPD3, SLITRK1, CHRM3, TLCD1, OR2M3, TMEM171, S1PR3, STOM, EPHA7, EPHA3, FAM172BP, NCKAP1L, GJA1, LRFN5, ZDHHC15, IL13RA2, TOR1AIP2, SHISA3, TMEM154, KCNQ3, OR5B21, NEO1, CD163L1, PDE3B, PLXNA2, ROBO1, TBXAS1, SLC22A3, CXCR4, NRP2, GPR65, LGR5, AGMO, IL1RL1, B4GALT6, CLEC2B, SEL1L3, ADGRL3, PCDHB5, OR2M4, FLRT2, PCDH17, PCDH11X, OR2H1, STXBP6, GDAP1, MCTP2, PCDHB6, HRASLS, ATP8B2, MARCH4, PCDHB2, CACHD1, PCDHB16, GPR135, MYCT1, CLMP, PCDH11Y | |
| Dystrophin-Associated Glycoprotein Complex | 2 | 11 | 4.64E-03 | 0.010 | PGM5, DMD | |
| Glycoprotein Complex | 2 | 11 | 4.64E-03 | 0.010 | PGM5, DMD | |
| Intrinsic Component Of Membrane | 61 | 0 | 4.69E-03 | 0.008 | AADAC, CD33, ENTPD3, SLITRK1, CHRM3, TLCD1, OR2M3, TMEM171, S1PR3, STOM, EPHA7, EPHA3, FAM172BP, NCKAP1L, GJA1, LRFN5, ZDHHC15, IL13RA2, TOR1AIP2, SHISA3, TMEM154, KCNQ3, OR5B21, NEO1, CD163L1, PDE3B, PLXNA2, ROBO1, TBXAS1, SLC22A3, CXCR4, NRP2, GPR65, LGR5, AGMO, IL1RL1, B4GALT6, CLEC2B, SEL1L3, ADGRL3, PCDHB5, OR2M4, FLRT2, PCDH17, PCDH11X, OR2H1, STXBP6, CNTN5, GDAP1, MCTP2, PCDHB6, HRASLS, ATP8B2, MARCH4, PCDHB2, CACHD1, PCDHB16, GPR135, MYCT1, CLMP, PCDH11Y | |
| Plasma Membrane | 54 | 0 | 4.88E-03 | 0.008 | ARRDC4, CD33, ENTPD3, SLITRK1, CHRM3, TLCD1, DMD, OR2M3, PIK3AP1, FGF13, S1PR3, STOM, EPHA7, EPHA3, GRB14, NCKAP1L, GJA1, GNGT2, KCNQ3, KISS1, SERPINB2, OR5B21, NEO1, CD163L1, PGM5, PIK3CG, PLAU, PLXNA2, ROBO1, SLC22A3, CXCR4, NRP2, GPR65, LGR5, IL1RL1, CLEC2B, CNKSR2, IRAK3, EPB41L3, ADGRL3, TBC1D30, OR2M4, FLRT2, PCDH11X, OR2H1, STXBP6, SHC3, CNTN5, ATP8B2, SAMSN1, GPR135, VEPH1, CLMP, PCDH11Y | |
| Cell Projection | 23 | 1 | 6.85E-03 | 0.010 | PLCB4, EFHC1, ROBO1, CNKSR2, CHRM3, DOCK10, RGS10, DMD, ADGRL3, TBC1D30, FGF13, SAMSN1, FLRT2, EPHA7, EPHA3, MPP4, TTLL11, NRP2, FOXA1, MAFIP, MMP3, KCNQ3, KISS1 | |
| Intercalated Disc | 3 | 4 | 7.17E-03 | 0.012 | FGF13, PGM5, GJA1 | |
| Cell-Cell Adherens Junction | 4 | 3 | 7.44E-03 | 0.013 | STXBP6, PGM5, GJA1, MPP4 | |
| Neuron Projection | 16 | 1 | 7.92E-03 | 0.011 | PLCB4, ROBO1, NRP2, CNKSR2, CHRM3, DOCK10, RGS10, MMP3, KCNQ3, KISS1, ADGRL3, FGF13, FLRT2, EPHA7, EPHA3, MPP4 | |
| Axolemma | 2 | 8 | 8.43E-03 | 0.010 | ROBO1, EPB41L3 | |
| Costamere | 2 | 8 | 9.16E-03 | 0.010 | PGM5, DMD | |
| Cell Junction | 16 | 1 | 9.46E-03 | 0.010 | PGM5, GJA1, CXCR4, PLAU, POF1B, SLITRK1, CHRM3, STXBP6, LAMA1, CLMP, DMD, EPB41L3, ADGRL3, FGF13, FLRT2, MPP4 | |
| Neuron Projection Terminus | 4 | 2 | 9.65E-03 | 0.013 | DMD, CHRM3, MPP4, RGS10 | |
| Tertiary Granule Membrane | 3 | 4 | 1.02E-02 | 0.012 | CD33, STOM, PLAU | |
| Synapse Part | 12 | 1 | 1.19E-02 | 0.011 | PLCB4, MCTP2, CNKSR2, CHRM3, SYTL3, DOCK10, RGS10, DMD, EPB41L3, BCL11A, EPHA7, MPP4 | |
| Cell-Cell Contact Zone | 3 | 3 | 1.39E-02 | 0.011 | FGF13, PGM5, GJA1 | |
| Axon Part | 8 | 1 | 1.60E-02 | 0.011 | KCNQ3, EPB41L3, CXCR4, ROBO1, FGF13, CHRM3, MPP4, RGS10 | |
| Extracellular Matrix | 7 | 1 | 1.79E-02 | 0.011 | MMP3, IL1RL1, LAMA4, TFPI2, LAMA1, FLRT2, ABI3BP | |
| Cyclin D2-Cdk4 Complex | 1 | 33 | 1.80E-02 | 0.005 | CCND2 | |
| Spot Adherens Junction | 1 | 33 | 1.80E-02 | 0.005 | PGM5 | |
| Phosphatidylinositol 3-Kinase Complex, Class Ib | 1 | 33 | 1.80E-02 | 0.005 | PIK3CG | |
| Laminin-1 Complex | 1 | 33 | 1.80E-02 | 0.005 | LAMA1 | |
| Laminin-3 Complex | 1 | 33 | 1.80E-02 | 0.005 | LAMA1 | |
| Rod Spherule | 1 | 33 | 1.80E-02 | 0.005 | MPP4 | |
| Beta-Catenin-Tcf7L2 Complex | 1 | 33 | 1.80E-02 | 0.005 | TCF4 | |
| Specific Granule Membrane | 3 | 3 | 1.83E-02 | 0.011 | CD33, STOM, PLAU | |
| Cell Surface | 10 | 1 | 1.87E-02 | 0.011 | SRPX, CXCR4, PLAU, LRFN5, ROBO1, CLMP, DMD, KCNQ3, IL1RL1, NEO1 | |
| Syntrophin Complex | 1 | 25 | 2.39E-02 | 0.005 | DMD | |
| Catenin-Tcf7L2 Complex | 1 | 25 | 2.39E-02 | 0.005 | TCF4 | |
| Axon Terminus | 3 | 2 | 2.71E-02 | 0.010 | CHRM3, MPP4, RGS10 | |
| Phosphatidylinositol 3-Kinase Complex, Class I | 1 | 20 | 2.98E-02 | 0.005 | PIK3CG | |
| Cone Cell Pedicle | 1 | 20 | 2.98E-02 | 0.005 | MPP4 | |
| Cell Body | 9 | 1 | 3.01E-02 | 0.010 | EFHC1, ROBO1, CNKSR2, RGS10, MMP3, KCNQ3, KISS1, EPHA7, NEO1 | |
| Neuronal Cell Body | 8 | 1 | 3.46E-02 | 0.010 | KCNQ3, KISS1, EFHC1, ROBO1, CNKSR2, EPHA7, RGS10, NEO1 | |
| Cbm Complex | 1 | 16 | 3.57E-02 | 0.005 | CRADD | |
| Endoplasmic Reticulum | 16 | 0 | 4.33E-02 | 0.009 | AADAC, PDE3B, PLCB4, IL24, ATP8B2, TBXAS1, CYP2J2, FKBP10, FLRT2, STOM, SRPX, GJA1, ZDHHC15, TOR1AIP2, SHISA3, AGMO | |
| Laminin Complex | 1 | 12 | 4.73E-02 | 0.005 | LAMA1 | |
| Adherens Junction | 7 | 1 | 5.18E-02 | 0.010 | PGM5, GJA1, PLAU, POF1B, STXBP6, FLRT2, MPP4 | |
| Organelle Outer Membrane | 4 | 1 | 5.24E-02 | 0.010 | GDAP1, BCL2A1, GJA1, NAV3 | |
| Outer Membrane | 4 | 1 | 5.38E-02 | 0.010 | GDAP1, BCL2A1, GJA1, NAV3 | |
| Endoplasmic Reticulum Membrane | 11 | 1 | 5.46E-02 | 0.009 | AADAC, GJA1, TOR1AIP2, SHISA3, AGMO, ATP8B2, TBXAS1, METTL7B, CYP2J2, SFTA3, FLRT2 | |
| **Enrichment for Molecular Function** | | | | | |  |
| Lipase Activity | 6 | 3 | 3.80E-04 | 0.018 | HRASLS, CHRM3, AADAC, PLCB1, PLCB4, FAM83B |  |
| Phosphoric Diester Hydrolase Activity | 5 | 4 | 3.81E-04 | 0.018 | CHRM3, PDE3B, PLCB1, PLCB4, FAM83B |  |
| Kinase Binding | 15 | 1 | 3.83E-04 | 0.014 | GRB14, PKIA, PDE3B, GJA1, PLCB4, CNKSR2, SAMD5, CCND2, KCNQ3, BCL11A, SHC3, PTPN22, EPHA7, EPHA3, FAM83B |  |
| Phosphatidylinositol Phospholipase C Activity | 3 | 11 | 5.00E-04 | 0.015 | PLCB1, CHRM3, PLCB4 |  |
| Phospholipase Activity | 5 | 4 | 6.34E-04 | 0.017 | HRASLS, CHRM3, PLCB1, PLCB4, FAM83B |  |
| Phospholipase C Activity | 3 | 10 | 6.94E-04 | 0.014 | PLCB1, CHRM3, PLCB4 |  |
| Gpi-Linked Ephrin Receptor Activity | 2 | 28 | 7.40E-04 | 0.011 | EPHA7, EPHA3 |  |
| Calcium Ion Binding | 13 | 1 | 9.60E-04 | 0.014 | PCDH17, PCDH11X, PLCB4, EFHC1, MCTP2, PCDHB6, PCDH11Y, PCDHB2, PLCB1, ADGRL3, FKBP10, PCDHB5, PCDHB16 |  |
| Axon Guidance Receptor Activity | 2 | 18 | 1.91E-03 | 0.010 | ROBO1, EPHA7 |  |
| Semaphorin Receptor Activity | 2 | 18 | 1.91E-03 | 0.010 | NRP2, PLXNA2 |  |
| Phosphatidylcholine Transporter Activity | 2 | 15 | 2.69E-03 | 0.010 | PITPNC1, PLTP |  |
| Phosphatidylinositol 3-Kinase Regulatory Subunit Binding | 2 | 15 | 2.69E-03 | 0.010 | PIK3AP1, FAM83B |  |
| Protein Tyrosine Kinase Binding | 4 | 4 | 2.82E-03 | 0.014 | GRB14, SHC3, PTPN22, GJA1 |  |
| Protein Binding | 63 | 0 | 2.94E-03 | 0.008 | ARHGDIB, ARRDC4, BCL2A1, NAV3, EFHC1, SYTL3, CCND2, DMD, PIK3AP1, FGF13, S1PR3, STOM, EPHA7, EPHA3, GRB14, GJA1, IL1B, IL13RA2, TOR1AIP2, HMGCS1, FOXA1, KCNQ3, KISS1, LAMA4, NEO1, FAM83B, PKIA, PDE3B, PIK3CG, PLCB4, ROBO1, CCL2, LAMA1, RGS10, TCF4, HIST2H4B, CXCR4, NRP2, SAMD5, CRADD, GMFG, IL1RL1, EML6, RAPGEF5, CNKSR2, CARD8, IL24, ESM1, IRAK3, EPB41L3, PLCB1, ADGRL3, TBC1D30, PTPN22, FLRT2, STXBP6, BCL11A, SHC3, DOCK10, SAMSN1, MPP4, GPR135, POF1B |  |
| Enzyme Activator Activity | 10 | 1 | 3.34E-03 | 0.013 | GMFG, ARHGDIB, NCKAP1L, PLCB1, TBC1D30, FGF13, CARD8, TOR1AIP2, CCL2, RGS10 |  |
| Phosphatidylglycerol Binding | 2 | 13 | 3.59E-03 | 0.010 | PITPNC1, PLTP |  |
| Enzyme Regulator Activity | 16 | 1 | 3.99E-03 | 0.011 | ARHGDIB, PKIA, NCKAP1L, BCL2A1, TFPI2, CARD8, TOR1AIP2, CCL2, SERPINB7, RGS10, CCND2, GMFG, PLCB1, TBC1D30, FGF13, SERPINB2 |  |
| Enzyme Binding | 27 | 1 | 4.00E-03 | 0.010 | ARHGDIB, PKIA, PDE3B, PLCB4, CNKSR2, SYTL3, DOCK10, CCND2, DMD, PLCB1, TBC1D30, PTPN22, STOM, EPHA7, EPHA3, GRB14, GJA1, CXCR4, TOR1AIP2, STXBP6, SAMD5, CRADD, KCNQ3, BCL11A, SHC3, RAPGEF5, FAM83B |  |
| Phosphoprotein Binding | 4 | 3 | 4.14E-03 | 0.014 | GRB14, SHC3, SAMSN1, PDE3B |  |
| Transmembrane-Ephrin Receptor Activity | 2 | 11 | 4.61E-03 | 0.010 | EPHA7, EPHA3 |  |
| Ephrin Receptor Activity | 2 | 10 | 5.75E-03 | 0.010 | EPHA7, EPHA3 |  |
| Phosphatidic Acid Binding | 2 | 10 | 6.36E-03 | 0.010 | PITPNC1, PLTP |  |
| Binding | 105 | 0 | 7.41E-03 | 0.007 | ARHGDIB, BCL2A1, CD33, ENTPD3, CHRM3, CCND2, DMD, CYP2J2, FGF13, S1PR3, STOM, EPHA7, EPHA3, GRB14, NCKAP1L, GJA1, IL1B, IL13RA2, HMGCS1, FOXA1, MMP3, KCNQ3, KISS1, LAMA4, NEO1, PKIA, PDE3B, PGM5, PIK3CG, PLCB4, PLTP, ROBO1, CCL2, RGS10, TBXAS1, TCF4, HIST2H4B, CXCR4, NRP2, CRADD, GMFG, IL1RL1, B4GALT6, RAPGEF5, CLEC2B, CNKSR2, CARD8, IFI44L, IL24, ESM1, IRAK3, SP140, EPB41L3, PLCB1, ADGRL3, TBC1D30, PCDHB5, PTPN22, PITPNC1, FLRT2, ABI3BP, PDCD4, PCDH17, PCDH11X, STXBP6, BCL11A, SHC3, MCTP2, LMO3, PCDHB6, DOCK10, ATP8B2, MARCH4, PCDHB2, PRDM9, PRDM8, FKBP10, SAMSN1, PCDHB16, MPP4, GPR135, POF1B, PCDH11Y, ANKRD30A, ARRDC4, NAV3, EFHC1, SYTL3, FTSJ3, PIK3AP1, FOXR2, FAM172BP, TTLL11, ZDHHC15, TOR1AIP2, NRK, OR5B21, ZNF438, FAM83B, SSX8, LAMA1, SAMD5, AGMO, EML6, ZNF716 |  |
| Molecular Function Regulator | 23 | 1 | 8.02E-03 | 0.010 | ARHGDIB, PKIA, BCL2A1, CARD8, CCL2, IL24, DOCK10, RGS10, CCND2, PLCB1, TBC1D30, FGF13, FLRT2, EPHA7, NCKAP1L, TFPI2, IL1B, TOR1AIP2, SERPINB7, GMFG, SHC3, SERPINB2, RAPGEF5 |  |
| Transmembrane Receptor Protein Tyrosine Kinase Activity | 3 | 4 | 9.39E-03 | 0.012 | NRP2, EPHA7, EPHA3 |  |
| Protein Kinase Regulator Activity | 5 | 2 | 1.02E-02 | 0.013 | FGF13, GMFG, PKIA, NCKAP1L, CCND2 |  |
| Cytokine Binding | 4 | 2 | 1.02E-02 | 0.013 | NRP2, IL13RA2, IL1RL1, CXCR4 |  |
| Phospholipid Transporter Activity | 3 | 3 | 1.09E-02 | 0.012 | ATP8B2, PITPNC1, PLTP |  |
| Interleukin-33 Receptor Activity | 1 | 50 | 1.20E-02 | 0.005 | IL1RL1 |  |
| Hydroxymethylglutaryl-Coa Synthase Activity | 1 | 50 | 1.20E-02 | 0.005 | HMGCS1 |  |
| Cgmp-Inhibited Cyclic-Nucleotide Phosphodiesterase Activity | 1 | 50 | 1.20E-02 | 0.005 | PDE3B |  |
| Hepatocyte Growth Factor Receptor Binding | 1 | 50 | 1.20E-02 | 0.005 | ESM1 |  |
| Arachidonic Acid 11,12-Epoxygenase Activity | 1 | 50 | 1.20E-02 | 0.005 | CYP2J2 |  |
| Rrna (Uridine-2'-O-)-Methyltransferase Activity | 1 | 50 | 1.20E-02 | 0.005 | FTSJ3 |  |
| Udp-Galactose:Glucosylceramide Beta-1,4-Galactosyltransferase Activity | 1 | 50 | 1.20E-02 | 0.005 | B4GALT6 |  |
| Phosphatidylinositol 3-Kinase Catalytic Subunit Binding | 1 | 50 | 1.20E-02 | 0.005 | FAM83B |  |
| Chemorepellent Activity | 2 | 6 | 1.31E-02 | 0.010 | FLRT2, EPHA7 |  |
| Phosphoric Ester Hydrolase Activity | 7 | 1 | 1.37E-02 | 0.012 | PDE3B, PLCB1, PLCB4, ENTPD3, PTPN22, CHRM3, FAM83B |  |
| Growth Factor Receptor Binding | 4 | 2 | 1.38E-02 | 0.012 | IL1B, FLRT2, ESM1, FAM83B |  |
| Sphingolipid Binding | 2 | 6 | 1.40E-02 | 0.009 | LAMA1, PLTP |  |
| Quaternary Ammonium Group Binding | 2 | 6 | 1.68E-02 | 0.009 | PITPNC1, PLTP |  |
| Ephrin Receptor Binding | 2 | 6 | 1.68E-02 | 0.009 | EPHA7, PIK3CG |  |
| Phosphatidylcholine Binding | 2 | 6 | 1.68E-02 | 0.009 | PITPNC1, PLTP |  |
| Kinase Regulator Activity | 5 | 2 | 1.75E-02 | 0.012 | FGF13, GMFG, PKIA, NCKAP1L, CCND2 |  |
| Rho Gdp-Dissociation Inhibitor Activity | 1 | 33 | 1.80E-02 | 0.005 | ARHGDIB |  |
| Rrna (Uridine) Methyltransferase Activity | 1 | 33 | 1.80E-02 | 0.005 | FTSJ3 |  |
| Gtp Phosphohydrolase Activity | 1 | 33 | 1.80E-02 | 0.005 | ENTPD3 |  |
| 8-Oxo-Dgtp Phosphohydrolase Activity | 1 | 33 | 1.80E-02 | 0.005 | ENTPD3 |  |
| Dgtp Phosphohydrolase Activity | 1 | 33 | 1.80E-02 | 0.005 | ENTPD3 |  |
| Datp Phosphohydrolase Activity | 1 | 33 | 1.80E-02 | 0.005 | ENTPD3 |  |
| Dctp Phosphohydrolase Activity | 1 | 33 | 1.80E-02 | 0.005 | ENTPD3 |  |
| Dutp Phosphohydrolase Activity | 1 | 33 | 1.80E-02 | 0.005 | ENTPD3 |  |
| Dttp Phosphohydrolase Activity | 1 | 33 | 1.80E-02 | 0.005 | ENTPD3 |  |
| Nacht Domain Binding | 1 | 33 | 1.80E-02 | 0.005 | CARD8 |  |
| Transmembrane Receptor Protein Kinase Activity | 3 | 3 | 1.87E-02 | 0.011 | NRP2, EPHA7, EPHA3 |  |
| Ammonium Ion Binding | 3 | 3 | 2.03E-02 | 0.011 | PITPNC1, CHRM3, PLTP |  |
| Cytokine Receptor Activity | 3 | 3 | 2.14E-02 | 0.011 | IL13RA2, IL1RL1, CXCR4 |  |
| Isomerase Activity | 4 | 2 | 2.27E-02 | 0.011 | FKBP10, TBXAS1, HMGCS1, PGM5 |  |
| Tfiib-Class Transcription Factor Binding | 1 | 25 | 2.39E-02 | 0.005 | TCF4 |  |
| Linoleic Acid Epoxygenase Activity | 1 | 25 | 2.39E-02 | 0.005 | CYP2J2 |  |
| Phosphatidylethanolamine Transporter Activity | 1 | 25 | 2.39E-02 | 0.005 | PLTP |  |
| Protein Binding, Bridging Involved In Substrate Recognition For Ubiquitination | 1 | 25 | 2.39E-02 | 0.005 | ARRDC4 |  |
| Phosphatidylinositol 3-Kinase Binding | 2 | 4 | 2.65E-02 | 0.009 | PIK3AP1, FAM83B |  |
| Phosphotyrosine Residue Binding | 2 | 4 | 2.76E-02 | 0.009 | SHC3, SAMSN1 |  |
| Nucleoside-Triphosphatase Regulator Activity | 6 | 1 | 2.88E-02 | 0.011 | ARHGDIB, NCKAP1L, PLCB1, TBC1D30, TOR1AIP2, RGS10 |  |
| Protein Tyrosine Kinase Activity | 4 | 2 | 2.89E-02 | 0.011 | NRP2, SHC3, EPHA7, EPHA3 |  |
| Rrna (Guanine) Methyltransferase Activity | 1 | 20 | 2.97E-02 | 0.005 | FTSJ3 |  |
| Gap Junction Channel Activity Involved In Cardiac Conduction Electrical Coupling | 1 | 20 | 2.97E-02 | 0.005 | GJA1 |  |
| Transferase Activity, Transferring Acyl Groups, Acyl Groups Converted Into Alkyl On Transfer | 1 | 20 | 2.97E-02 | 0.005 | HMGCS1 |  |
| Extracellular Matrix Structural Constituent | 3 | 2 | 3.09E-02 | 0.010 | LAMA1, LAMA4, TFPI2 |  |
| Beta-Tubulin Binding | 2 | 4 | 3.26E-02 | 0.009 | FGF13, GJA1 |  |
| Cysteine-Type Endopeptidase Regulator Activity Involved In Apoptotic Process | 2 | 4 | 3.52E-02 | 0.009 | CARD8, BCL2A1 |  |
| Phosphoglucomutase Activity | 1 | 16 | 3.56E-02 | 0.005 | PGM5 |  |
| Gap Junction Channel Activity Involved In Cell Communication By Electrical Coupling | 1 | 16 | 3.56E-02 | 0.005 | GJA1 |  |
| Phosphatidic Acid Transporter Activity | 1 | 16 | 3.56E-02 | 0.005 | PLTP |  |
| Protein Binding, Bridging | 4 | 1 | 3.61E-02 | 0.010 | GRB14, ARRDC4, FLRT2, CRADD |  |
| Gtpase Activator Activity | 5 | 1 | 3.61E-02 | 0.010 | ARHGDIB, NCKAP1L, PLCB1, TBC1D30, RGS10 |  |
| Protein Kinase Activity | 9 | 1 | 3.78E-02 | 0.010 | PIK3CG, NRP2, CCL2, IRAK3, CCND2, NRK, SHC3, EPHA7, EPHA3 |  |
| Kinase Activity | 11 | 1 | 3.80E-02 | 0.009 | PIK3CG, PLAU, NRP2, CCL2, IRAK3, CCND2, NRK, PIK3AP1, SHC3, EPHA7, EPHA3 |  |
| Signaling Receptor Binding | 20 | 0 | 4.05E-02 | 0.008 | PIK3CG, CCL2, LAMA1, IL24, ESM1, SYTL3, FGF13, S1PR3, FLRT2, EPHA7, MPP4, GRB14, GJA1, IL1B, GMFG, KISS1, LAMA4, SHC3, NEO1, FAM83B |  |
| Oxidoreductase Activity, Acting On Paired Donors, With Incorporation Or Reduction Of Molecular Oxygen, Reduced Pteridine As One Donor, And Incorporation Of One Atom Of Oxygen | 1 | 14 | 4.14E-02 | 0.005 | AGMO |  |
| Rap Guanyl-Nucleotide Exchange Factor Activity | 1 | 14 | 4.14E-02 | 0.005 | RAPGEF5 |  |
| Phospholipase D Activity | 1 | 14 | 4.14E-02 | 0.005 | FAM83B |  |
| Connexin Binding | 1 | 14 | 4.14E-02 | 0.005 | GJA1 |  |
| 1-Phosphatidylinositol-4-Phosphate 3-Kinase Activity | 1 | 14 | 4.14E-02 | 0.005 | PIK3CG |  |
| Myosin Light Chain Binding | 1 | 14 | 4.14E-02 | 0.005 | CXCR4 |  |
| Toxin Transmembrane Transporter Activity | 1 | 14 | 4.14E-02 | 0.005 | SLC22A3 |  |
| Phosphotransferase Activity, Alcohol Group As Acceptor | 10 | 1 | 4.33E-02 | 0.009 | PIK3CG, NRP2, CCL2, IRAK3, CCND2, NRK, PIK3AP1, SHC3, EPHA7, EPHA3 |  |
| Histone-Lysine N-Methyltransferase Activity | 2 | 3 | 4.34E-02 | 0.008 | PRDM9, PRDM8 |  |
| G-Protein Alpha-Subunit Binding | 2 | 3 | 4.63E-02 | 0.008 | ADGRL3, RGS10 |  |
| Protein Phosphorylated Amino Acid Binding | 2 | 3 | 4.63E-02 | 0.008 | SHC3, SAMSN1 |  |
| Beta-N-Acetylglucosaminylglycopeptide Beta-1,4-Galactosyltransferase Activity | 1 | 12 | 4.72E-02 | 0.005 | B4GALT6 |  |
| Nadph-Hemoprotein Reductase Activity | 1 | 12 | 4.72E-02 | 0.005 | CYP2J2 |  |
| Interleukin-1 Receptor Activity | 1 | 12 | 4.72E-02 | 0.005 | IL1RL1 |  |
| C-X-C Chemokine Receptor Activity | 1 | 12 | 4.72E-02 | 0.005 | CXCR4 |  |
| G-Protein Coupled Acetylcholine Receptor Activity | 1 | 12 | 4.72E-02 | 0.005 | CHRM3 |  |
| Histone Methyltransferase Activity (H3-K9 Specific) | 1 | 12 | 4.72E-02 | 0.005 | PRDM8 |  |
| Protein-Glutamic Acid Ligase Activity | 1 | 12 | 4.72E-02 | 0.005 | TTLL11 |  |
| Sphingosine-1-Phosphate Receptor Activity | 1 | 12 | 4.72E-02 | 0.005 | S1PR3 |  |
| Tubulin-Glutamic Acid Ligase Activity | 1 | 12 | 4.72E-02 | 0.005 | TTLL11 |  |
| Cation Binding | 37 | 0 | 4.98E-02 | 0.008 | ZNF716, EFHC1, CHRM3, SYTL3, IRAK3, SP140, DMD, PLCB1, ADGRL3, CYP2J2, PCDHB5, PITPNC1, PCDH17, PCDH11X, ZDHHC15, MMP3, BCL11A, ZNF438, PDE3B, PGM5, PLCB4, PLTP, MCTP2, LMO3, PCDHB6, ATP8B2, TBXAS1, MARCH4, PCDHB2, PRDM9, PRDM8, FKBP10, PCDHB16, NRP2, AGMO, PCDH11Y, B4GALT6 |  |
| Peptidase Regulator Activity | 5 | 1 | 5.00E-02 | 0.010 | BCL2A1, TFPI2, CARD8, SERPINB2, SERPINB7 |  |
| Gtpase Regulator Activity | 5 | 1 | 5.16E-02 | 0.010 | ARHGDIB, NCKAP1L, PLCB1, TBC1D30, RGS10 |  |
| Calcium-Dependent Phospholipid Binding | 2 | 3 | 5.23E-02 | 0.008 | MCTP2, SYTL3 |  |
| Camp-Dependent Protein Kinase Inhibitor Activity | 1 | 11 | 5.29E-02 | 0.005 | PKIA |  |
| Vascular Endothelial Growth Factor-Activated Receptor Activity | 1 | 11 | 5.29E-02 | 0.005 | NRP2 |  |
