# Supplementary material for: LAMA4-Regulating miR-4274 and Its Host Gene SORCS2 Play a Role in IGFBP6-Dependent Effects on Phenotype of Basal-Like Breast Cancer
Source: Front Mol Biosci. 2019 Nov 8;6:122. doi: 10.3389/fmolb.2019.00122 (PMC6857517; doi:10.3389/fmolb.2019.00122)
Supplement: Supplementary file 3 [file Table_3.DOCX]

**Supplementary Table 3.** Enrichment analysis of a set of genes significantly downregulated after a transcriptional knockdown of IGFBP6 gene in MDA-MB-231 cells (N=183) (FDR p 0:01, fold change 2).

| **Ontology Component** | | **Gene Overlap** | | **Percent Overlap** | **P-value** | **Jaccard similarity** | **List of overlapping genes** | |  |  |  |  |  |  |  |  |  |  |  |  |  |  |  |  |  |  |  |  |  |  |  |
| --- | --- | --- | --- | --- | --- | --- | --- | --- | --- | --- | --- | --- | --- | --- | --- | --- | --- | --- | --- | --- | --- | --- | --- | --- | --- | --- | --- | --- | --- | --- | --- |
| **Enrichment for Biological Processes** | | | | | | | | |  |  |  |  |  |  |  |  |  |  |  |  |  |  |  |  |  |  |  |  |  |  |  |
| Regulation Of Signal Transduction | | 57 | | 1 | 6.72E-10 | 0.015 | PLK2, ARRB1, ATP2B4, PDE10A, MIR181B1, ABCA1, TSPYL5, CLU, BMP4, BMP1, ARL2BP, TRIM6, CSF1, TIAM2, LPAR1, LYPD6B, EYA4, F2RL1, EFEMP1, FBP1, GATA6, GBP1, PRICKLE1, IGFBP4, INHBB, TRIB2, ITGB3, CDON, MEF2C, MAP3K1, LTBP1, BAIAP2L1, PTPRS, SOX4, SLC44A2, ZMIZ1, PMEPA1, SNAI2, VEGFA, NXN, HHIP, TGFA, TGFB2, THBS1, TGFBR3, TGM2, TIMP3, TLR3, SHISA2, GPRC5A, CDC42BPA, TNFSF10, ADAMTS12, NOG, AFAP1L2, OPTN, NUAK1 | |  |  |  |  |  |  |  |  |  |  |  |  |  |  |  |  |  |  |  |  |  |  |  |
| Wound Healing | | 12 | | 7 | 9.10E-10 | 0.038 | OPTN, MAP3K1, VEGFA, ITGB3, DSP, SDC2, BMP4, TGFA, TGFB2, NOG, F13A1, LTBP1 | |  |  |  |  |  |  |  |  |  |  |  |  |  |  |  |  |  |  |  |  |  |  |  |
| Tube Morphogenesis | | 16 | | 4 | 3.11E-09 | 0.031 | ZMIZ1, MEF2C, HEG1, GATA6, NOG, CSF1, CTSD, VEGFA, WT1, HHIP, FAT4, BMP4, EFNB2, TGFB2, THBS1, TGFBR3 | |  |  |  |  |  |  |  |  |  |  |  |  |  |  |  |  |  |  |  |  |  |  |  |
| Regulation Of Response To Stimulus | | 68 | | 1 | 3.90E-09 | 0.013 | PLK2, ARRB1, ATP2B4, PDE10A, MIR181B1, ABCA1, TSPYL5, FBXO32, CLU, BMP4, BMP1, C4BPB, ARL2BP, TRIM6, CSF1, TIAM2, LPAR1, EFNB2, LYPD6B, EYA4, F2RL1, EFEMP1, FBP1, TNFRSF21, HLA-A, HLA-B, GATA6, GBP1, PRICKLE1, IGFBP4, INHBB, TRIB2, ITGB3, HLA-DRA, CDON, MEF2C, MAP3K1, LTBP1, PLPP4, BAIAP2L1, PTPRS, SOX4, SLC44A2, ZMIZ1, MTUS1, PMEPA1, SNAI2, VEGFA, NXN, HHIP, TGFA, TGFB2, THBS1, TGFBR3, TGM2, TIMP3, SLAMF7, TLR3, SHISA2, GPRC5A, CDC42BPA, CASK, TNFSF10, ADAMTS12, NOG, AFAP1L2, OPTN, NUAK1 | |  |  |  |  |  |  |  |  |  |  |  |  |  |  |  |  |  |  |  |  |  |  |  |
| Anatomical Structure Development | | 65 | | 1 | 4.79E-09 | 0.014 | ATP2B4, AK4, CDH11, CLU, BMP4, BMP1, TDRD7, DPYSL2, DSP, CSF1, FLG, LPAR1, EFNB2, EYA4, F2RL1, EFEMP1, HAS2, HLA-A, GATA6, PRICKLE1, IGFBP4, INHBB, ITGB3, CDON, MEF2C, MAP3K1, SAMD9L, SNX10, LAMA3, LCP1, LTBP1, PLXNA1, TENM3, SCN5A, PTPRS, SOX4, ZMIZ1, HEG1, SDC2, SNAI2, TUFT1, UGCG, VEGFA, WT1, NXN, TGFA, TGFB2, THBS1, TGFBR3, TIMP3, TLL1, TLR3, SLC7A5, ILDR2, SHISA2, TNS3, EVI5, FAT4, CASK, TNFSF10, CRISPLD2, NOG, TSPAN2, RAD50, OPTN | |  |  |  |  |  |  |  |  |  |  |  |  |  |  |  |  |  |  |  |  |  |  |  |
| Anatomical Structure Morphogenesis | | 36 | | 2 | 6.00E-09 | 0.019 | PLXNA1, TENM3, CDH11, CLU, SCN5A, BMP4, SOX4, TDRD7, ZMIZ1, HEG1, DSP, SDC2, CSF1, SNAI2, CTSD, TUFT1, VEGFA, WT1, HHIP, EFNB2, TGFB2, THBS1, TGFBR3, TGM2, EYA4, EFEMP1, HAS2, GATA6, ITGB3, FAT4, CDON, MEF2C, MAP3K1, CRISPLD2, NOG, PARD6B | |  |  |  |  |  |  |  |  |  |  |  |  |  |  |  |  |  |  |  |  |  |  |  |
| Tissue Morphogenesis | | 18 | | 3 | 6.96E-09 | 0.028 | MEF2C, MAP3K1, HEG1, DSP, NOG, CSF1, SNAI2, CTSD, PLXNA1, VEGFA, WT1, HHIP, FAT4, BMP4, EFNB2, TGFB2, TGFBR3, TGM2 | |  |  |  |  |  |  |  |  |  |  |  |  |  |  |  |  |  |  |  |  |  |  |  |
| Regulation Of Cell Communication | | 60 | | 1 | 7.37E-09 | 0.014 | PLK2, ARRB1, ATP2B4, PDE10A, MIR181B1, ABCA1, TSPYL5, CLU, BMP4, BMP1, ARL2BP, TRIM6, CPT1A, CSF1, TIAM2, LPAR1, LYPD6B, EYA4, F2RL1, EFEMP1, FBP1, GATA6, GBP1, PRICKLE1, IGFBP4, INHBB, TRIB2, ITGB3, CDON, MEF2C, MAP3K1, LTBP1, BAIAP2L1, SCN9A, PTPRS, SOX4, SLC44A2, ZMIZ1, PMEPA1, SNAI2, VEGFA, NXN, HHIP, TGFA, TGFB2, THBS1, TGFBR3, TGM2, TIMP3, TLR3, SHISA2, GPRC5A, CDC42BPA, CASK, TNFSF10, ADAMTS12, NOG, AFAP1L2, OPTN, NUAK1 | |  |  |  |  |  |  |  |  |  |  |  |  |  |  |  |  |  |  |  |  |  |  |  |
| Regulation Of Signaling | | 60 | | 1 | 9.36E-09 | 0.014 | PLK2, ARRB1, ATP2B4, PDE10A, MIR181B1, ABCA1, TSPYL5, CLU, BMP4, BMP1, ARL2BP, TRIM6, CPT1A, CSF1, TIAM2, LPAR1, LYPD6B, EYA4, F2RL1, EFEMP1, FBP1, GATA6, GBP1, PRICKLE1, IGFBP4, INHBB, TRIB2, ITGB3, CDON, MEF2C, MAP3K1, LTBP1, BAIAP2L1, SCN9A, PTPRS, SOX4, SLC44A2, ZMIZ1, PMEPA1, SNAI2, VEGFA, NXN, HHIP, TGFA, TGFB2, THBS1, TGFBR3, TGM2, TIMP3, TLR3, SHISA2, GPRC5A, CDC42BPA, CASK, TNFSF10, ADAMTS12, NOG, AFAP1L2, OPTN, NUAK1 | |  |  |  |  |  |  |  |  |  |  |  |  |  |  |  |  |  |  |  |  |  |  |  |
| Regulation Of Multicellular Organismal Process | | 55 | | 1 | 1.73E-08 | 0.014 | PLK2, ARRB1, ATP2B4, MIR181B1, ABCA1, MYL9, ADGRE5, FBXO32, CLU, BMP4, BMP1, DPYSL2, DSP, TRIM6, CSF1, LPAR1, EFNB2, F2RL1, EFEMP1, TNFRSF21, HAS2, HLA-A, GATA6, GBP1, PRICKLE1, INHBB, TRIB2, ITGB3, CDON, MEF2C, LAMA3, ASAP1, PLXNA1, TENM3, SCN5A, PTPRS, ZMIZ1, HEG1, ZNF608, SDC2, SNAI2, VEGFA, WT1, HHIP, TGFB2, THBS1, TGFBR3, TLR3, FAT4, CASK, ADAMTS12, NOG, LIPG, AFAP1L2, OPTN | |  |  |  |  |  |  |  |  |  |  |  |  |  |  |  |  |  |  |  |  |  |  |  |
| Regulation Of Protein Modification Process | | 39 | | 1 | 1.83E-08 | 0.017 | ARRB1, ATP2B4, MIR181B1, TSPYL5, CLU, BMP4, SOX4, CEMIP, ARL2BP, TRIM6, CSF1, PMEPA1, SNAI2, VEGFA, NXN, LPAR1, TGFA, TGFB2, THBS1, TGFBR3, TIMP3, TLR3, F2RL1, ACSL1, GBP1, PRICKLE1, IGFBP4, INHBB, TRIB2, ITGB3, GPRC5A, SH3RF2, CDON, MAP3K1, NOG, RAD50, AFAP1L2, CTDSPL, NUAK1 | |  |  |  |  |  |  |  |  |  |  |  |  |  |  |  |  |  |  |  |  |  |  |  |
| Biological Adhesion | | 25 | | 2 | 2.50E-08 | 0.022 | MYL9, GBP1, GBP2, GBP3, CD22, TENM3, ADGRE5, CDH11, INHBB, ITGB3, BAIAP2L1, FAT4, PTPRS, CASK, CDON, ADAMTS12, DSP, PDLIM1, LAMA3, MPZL3, EFNB2, TGFB2, THBS1, NUAK1, SLAMF7 | |  |  |  |  |  |  |  |  |  |  |  |  |  |  |  |  |  |  |  |  |  |  |  |
| Negative Regulation Of Cell Communication | | 32 | | 2 | 3.43E-08 | 0.019 | PLK2, ARRB1, ATP2B4, PDE10A, CLU, BMP4, PTPRS, PMEPA1, SNAI2, VEGFA, NXN, HHIP, LPAR1, TGFB2, THBS1, TGFBR3, TIMP3, EYA4, TLR3, F2RL1, FBP1, SHISA2, GBP1, PRICKLE1, IGFBP4, INHBB, GPRC5A, TNFSF10, ADAMTS12, NOG, LTBP1, OPTN | |  |  |  |  |  |  |  |  |  |  |  |  |  |  |  |  |  |  |  |  |  |  |  |
| Negative Regulation Of Signaling | | 32 | | 2 | 3.64E-08 | 0.019 | PLK2, ARRB1, ATP2B4, PDE10A, CLU, BMP4, PTPRS, PMEPA1, SNAI2, VEGFA, NXN, HHIP, LPAR1, TGFB2, THBS1, TGFBR3, TIMP3, EYA4, TLR3, F2RL1, FBP1, SHISA2, GBP1, PRICKLE1, IGFBP4, INHBB, GPRC5A, TNFSF10, ADAMTS12, NOG, LTBP1, OPTN | |  |  |  |  |  |  |  |  |  |  |  |  |  |  |  |  |  |  |  |  |  |  |  |
| Response To Wounding | | 15 | | 4 | 4.22E-08 | 0.029 | MAP3K1, DSP, SDC2, NOG, LCP1, LTBP1, OPTN, INHBB, VEGFA, ITGB3, CLU, BMP4, TGFA, TGFB2, F13A1 | |  |  |  |  |  |  |  |  |  |  |  |  |  |  |  |  |  |  |  |  |  |  |  |
| Regulation Of Cell Differentiation | | 39 | | 1 | 5.11E-08 | 0.017 | PLK2, MIR181B1, ABCA1, MYL9, PLXNA1, TENM3, CLU, BMP4, PTPRS, ZMIZ1, DPYSL2, ZNF608, SDC2, TRIM6, CSF1, SNAI2, VEGFA, LPAR1, EFNB2, TGFB2, THBS1, TGFBR3, TLR3, EFEMP1, TNFRSF21, HAS2, HLA-A, GATA6, GBP1, PRICKLE1, TRIB2, ITGB3, FAT4, CASK, CDON, ADAMTS12, MEF2C, NOG, ASAP1 | |  |  |  |  |  |  |  |  |  |  |  |  |  |  |  |  |  |  |  |  |  |  |  |
| Negative Regulation Of Signal Transduction | | 30 | | 2 | 5.33E-08 | 0.019 | ARRB1, ATP2B4, PDE10A, CLU, BMP4, PTPRS, PMEPA1, SNAI2, VEGFA, NXN, HHIP, LPAR1, TGFB2, THBS1, TGFBR3, TIMP3, EYA4, TLR3, F2RL1, FBP1, SHISA2, GBP1, PRICKLE1, IGFBP4, GPRC5A, TNFSF10, ADAMTS12, NOG, LTBP1, OPTN | |  |  |  |  |  |  |  |  |  |  |  |  |  |  |  |  |  |  |  |  |  |  |  |
| Negative Regulation Of Cartilage Development | | 6 | | 20 | 5.46E-08 | 0.030 | BMP4, ADAMTS12, TGFB2, NOG, SNAI2, EFEMP1 | |  |  |  |  |  |  |  |  |  |  |  |  |  |  |  |  |  |  |  |  |  |  |  |
| Cell Surface Receptor Signaling Pathway | | 44 | | 1 | 8.34E-08 | 0.015 | ABCA1, ADGRF1, PLXNA1, CD22, ADGRE5, BMP4, SOX4, SDC2, CSF1, SNAI2, VEGFA, NXN, HHIP, EFNB2, TGFA, TGFB2, TGFBR3, TLR3, F13A1, ACSL1, EFEMP1, TNFRSF21, HLA-A, HLA-B, GBP1, PORCN, GBP2, PRICKLE1, INHBB, ITGB3, CPEB4, CD82, SORBS2, HLA-DRA, FAT4, TNFSF10, CDON, MEF2C, MAP3K1, NOG, LCP1, LTBP1, PLPP4, TSPAN2 | |  |  |  |  |  |  |  |  |  |  |  |  |  |  |  |  |  |  |  |  |  |  |  |
| Blood Vessel Morphogenesis | | 9 | | 7 | 1.37E-07 | 0.032 | ZMIZ1, VEGFA, HEG1, BMP4, EFNB2, TGFB2, THBS1, TGFBR3, NOG | |  |  |  |  |  |  |  |  |  |  |  |  |  |  |  |  |  |  |  |  |  |  |  |
| Positive Regulation Of Protein Modification Process | | 29 | | 2 | 1.60E-07 | 0.019 | ARRB1, ATP2B4, MIR181B1, TSPYL5, CLU, BMP4, SOX4, CEMIP, ARL2BP, TRIM6, CSF1, SNAI2, VEGFA, LPAR1, TGFA, TGFB2, THBS1, TLR3, F2RL1, ACSL1, PRICKLE1, IGFBP4, INHBB, TRIB2, ITGB3, CDON, MAP3K1, RAD50, AFAP1L2 | |  |  |  |  |  |  |  |  |  |  |  |  |  |  |  |  |  |  |  |  |  |  |  |
| Regulation Of Cell Adhesion | | 21 | | 2 | 1.60E-07 | 0.022 | TNFRSF21, HAS2, HLA-A, GBP1, PLXNA1, TENM3, ITGB3, BMP4, CASK, ZMIZ1, CYTIP, ZNF608, LAMA3, CSF1, SNAI2, VEGFA, EFNB2, TGFB2, THBS1, TGM2, NUAK1 | |  |  |  |  |  |  |  |  |  |  |  |  |  |  |  |  |  |  |  |  |  |  |  |
| Regulation Of Phosphate Metabolic Process | | 36 | | 1 | 1.62E-07 | 0.017 | ARRB1, ATP2B4, MIR181B1, CLU, BMP4, CEMIP, ARL2BP, TRIM6, CSF1, PMEPA1, VEGFA, LPAR1, TGFA, TGFB2, THBS1, TGFBR3, TIMP3, TLR3, F2RL1, ACSL1, FBP1, GBP1, IGFBP4, INHBB, TRIB2, ITGB3, GPRC5A, SH3RF2, CDON, MEF2C, MAP3K1, NOG, RAD50, AFAP1L2, CTDSPL, NUAK1 | |  |  |  |  |  |  |  |  |  |  |  |  |  |  |  |  |  |  |  |  |  |  |  |
| Regulation Of Phosphorus Metabolic Process | | 36 | | 1 | 1.64E-07 | 0.017 | ARRB1, ATP2B4, MIR181B1, CLU, BMP4, CEMIP, ARL2BP, TRIM6, CSF1, PMEPA1, VEGFA, LPAR1, TGFA, TGFB2, THBS1, TGFBR3, TIMP3, TLR3, F2RL1, ACSL1, FBP1, GBP1, IGFBP4, INHBB, TRIB2, ITGB3, GPRC5A, SH3RF2, CDON, MEF2C, MAP3K1, NOG, RAD50, AFAP1L2, CTDSPL, NUAK1 | |  |  |  |  |  |  |  |  |  |  |  |  |  |  |  |  |  |  |  |  |  |  |  |
| Regulation Of Protein Phosphorylation | | 32 | | 1 | 1.81E-07 | 0.018 | ARRB1, ATP2B4, MIR181B1, CLU, BMP4, CEMIP, ARL2BP, TRIM6, CSF1, PMEPA1, VEGFA, LPAR1, TGFA, TGFB2, THBS1, TGFBR3, TIMP3, TLR3, F2RL1, ACSL1, GBP1, IGFBP4, INHBB, TRIB2, ITGB3, GPRC5A, CDON, MAP3K1, NOG, RAD50, AFAP1L2, CTDSPL | |  |  |  |  |  |  |  |  |  |  |  |  |  |  |  |  |  |  |  |  |  |  |  |
| Animal Organ Morphogenesis | | 19 | | 2 | 1.95E-07 | 0.023 | HAS2, GATA6, TENM3, SCN5A, FAT4, BMP4, SOX4, ZMIZ1, MEF2C, NOG, CSF1, SNAI2, TUFT1, VEGFA, HHIP, EFNB2, TGFB2, TGFBR3, EFEMP1 | |  |  |  |  |  |  |  |  |  |  |  |  |  |  |  |  |  |  |  |  |  |  |  |
| Negative Regulation Of Response To Stimulus | | 34 | | 1 | 2.05E-07 | 0.017 | PLK2, ARRB1, ATP2B4, PDE10A, CLU, BMP4, PTPRS, C4BPB, PMEPA1, SNAI2, VEGFA, NXN, HHIP, LPAR1, TGFB2, THBS1, TGFBR3, TIMP3, EYA4, TLR3, F2RL1, FBP1, SHISA2, HLA-A, GBP1, PRICKLE1, IGFBP4, GPRC5A, CASK, TNFSF10, ADAMTS12, NOG, LTBP1, OPTN | |  |  |  |  |  |  |  |  |  |  |  |  |  |  |  |  |  |  |  |  |  |  |  |
| Regulation Of Intracellular Signal Transduction | | 37 | | 1 | 2.24E-07 | 0.016 | PLK2, ARRB1, ATP2B4, PDE10A, MIR181B1, ABCA1, TSPYL5, CLU, BMP4, SLC44A2, ARL2BP, TRIM6, CSF1, SNAI2, TIAM2, VEGFA, LPAR1, TGFA, TGFB2, THBS1, TGFBR3, TGM2, TIMP3, TLR3, F2RL1, FBP1, GBP1, IGFBP4, INHBB, TRIB2, ITGB3, CDC42BPA, TNFSF10, CDON, MAP3K1, OPTN, NUAK1 | |  |  |  |  |  |  |  |  |  |  |  |  |  |  |  |  |  |  |  |  |  |  |  |
| Negative Regulation Of Multicellular Organismal Process | | 30 | | 1 | 2.66E-07 | 0.018 | PLK2, ARRB1, ATP2B4, FBXO32, BMP4, PTPRS, ZNF608, SNAI2, VEGFA, WT1, HHIP, LPAR1, EFNB2, TGFB2, THBS1, TGFBR3, TLR3, F2RL1, EFEMP1, TNFRSF21, HLA-A, GATA6, GBP1, PRICKLE1, INHBB, TRIB2, ADAMTS12, MEF2C, NOG, ASAP1 | |  |  |  |  |  |  |  |  |  |  |  |  |  |  |  |  |  |  |  |  |  |  |  |
| Developmental Process | | 76 | | 1 | 2.69E-07 | 0.012 | ATP2B4, AK4, CDH11, CLU, BMP4, BMP1, CYP24A1, DPYSL2, DSP, CPT1A, CSF1, CTSD, FLG, LPAR1, EFNB2, EYA4, F2RL1, EFEMP1, HAS2, HLA-A, GATA6, PRICKLE1, IGFBP4, INHBB, ITGB3, MEF2C, MAP3K1, SAMD9L, LAMA3, LCP1, LTBP1, PLXNA1, RRBP1, SCN5A, SCP2, PTPRS, SOX4, SDC2, SNAI2, TUFT1, UGCG, VEGFA, WT1, TGFA, TGFB2, THBS1, TGFBR3, TGM2, TIMP3, TLL1, TLR3, SLC7A5, ILDR2, SHISA2, EVI5, SORBS2, CASK, TNFSF10, NOG, TSPAN2, RAD50, OPTN, MBNL2, TDRD7, CDON, SNX10, RBM47, TENM3, ZMIZ1, HEG1, NXN, HHIP, TNS3, FAT4, CRISPLD2, PARD6B | |  |  |  |  |  |  |  |  |  |  |  |  |  |  |  |  |  |  |  |  |  |  |  |
| Regulation Of Phosphorylation | | 33 | | 1 | 2.80E-07 | 0.017 | ARRB1, ATP2B4, MIR181B1, CLU, BMP4, CEMIP, ARL2BP, TRIM6, CSF1, PMEPA1, VEGFA, LPAR1, TGFA, TGFB2, THBS1, TGFBR3, TIMP3, TLR3, F2RL1, ACSL1, FBP1, GBP1, IGFBP4, INHBB, TRIB2, ITGB3, GPRC5A, CDON, MAP3K1, NOG, RAD50, AFAP1L2, CTDSPL | |  |  |  |  |  |  |  |  |  |  |  |  |  |  |  |  |  |  |  |  |  |  |  |
| Outflow Tract Morphogenesis | | 7 | | 11 | 2.83E-07 | 0.030 | MEF2C, VEGFA, BMP4, TGFB2, THBS1, TGFBR3, NOG | |  |  |  |  |  |  |  |  |  |  |  |  |  |  |  |  |  |  |  |  |  |  |  |
| Positive Regulation Of Cell Proliferation | | 26 | | 2 | 3.72E-07 | 0.019 | ARRB1, HAS2, HLA-A, MIR181B1, TNS3, GATA6, TSPYL5, ITGB3, CLU, SCN5A, BMP4, SOX4, CDON, ZMIZ1, MEF2C, NOG, CSF1, OPTN, VEGFA, EFNB2, TGFA, TGFB2, THBS1, TGFBR3, TGM2, EFEMP1 | |  |  |  |  |  |  |  |  |  |  |  |  |  |  |  |  |  |  |  |  |  |  |  |
| Regulation Of Cellular Protein Metabolic Process | | 46 | | 1 | 3.85E-07 | 0.014 | PLK2, ARRB1, ATP2B4, MIR181B1, TSPYL5, CLU, BMP4, C4BPB, ARL2BP, TRIM6, CSF1, CST4, LPAR1, F2RL1, ACSL1, GBP1, PRICKLE1, IGFBP4, INHBB, TRIB2, ITGB3, SH3RF2, CDON, MAP3K1, SOX4, CEMIP, PMEPA1, SNAI2, VEGFA, WT1, NXN, TGFA, TGFB2, THBS1, TGFBR3, TIMP3, TLR3, CPEB4, GPRC5A, CST7, TNFSF10, NOG, RAD50, AFAP1L2, CTDSPL, NUAK1 | |  |  |  |  |  |  |  |  |  |  |  |  |  |  |  |  |  |  |  |  |  |  |  |
| Regulation Of Anatomical Structure Morphogenesis | | 27 | | 2 | 4.28E-07 | 0.018 | PLK2, HAS2, MIR181B1, GATA6, GBP1, PRICKLE1, PLXNA1, ITGB3, BMP4, PTPRS, CASK, ADAMTS12, MEF2C, DPYSL2, SDC2, NOG, CSF1, SNAI2, VEGFA, WT1, HHIP, LPAR1, EFNB2, TGFB2, THBS1, TGFBR3, TLR3 | |  |  |  |  |  |  |  |  |  |  |  |  |  |  |  |  |  |  |  |  |  |  |  |
| Negative Regulation Of Cell Differentiation | | 22 | | 2 | 4.82E-07 | 0.020 | PLK2, HLA-A, ABCA1, GBP1, PRICKLE1, TRIB2, ITGB3, BMP4, PTPRS, ADAMTS12, ZNF608, TRIM6, NOG, SNAI2, ASAP1, VEGFA, LPAR1, EFNB2, TGFB2, TGFBR3, TLR3, EFEMP1 | |  |  |  |  |  |  |  |  |  |  |  |  |  |  |  |  |  |  |  |  |  |  |  |
| Regulation Of Cellular Response To Growth Factor Stimulus | | 13 | | 3 | 6.21E-07 | 0.026 | ADAMTS12, SHISA2, GATA6, NOG, PMEPA1, LTBP1, VEGFA, ITGB3, HHIP, BMP4, THBS1, TGFBR3, CASK | |  |  |  |  |  |  |  |  |  |  |  |  |  |  |  |  |  |  |  |  |  |  |  |
| Regulation Of Protein Metabolic Process | | 48 | | 1 | 6.50E-07 | 0.014 | PLK2, ARRB1, ATP2B4, MIR181B1, TSPYL5, CLU, BMP4, C4BPB, ARL2BP, TRIM6, CSF1, CST4, LPAR1, F2RL1, ACSL1, GBP1, PRICKLE1, IGFBP4, INHBB, TRIB2, ITGB3, SH3RF2, CDON, MAP3K1, SAMD9L, SOX4, CEMIP, PMEPA1, SNAI2, VEGFA, WT1, NXN, TGFA, TGFB2, THBS1, TGFBR3, TIMP3, TLR3, CPEB4, GPRC5A, CST7, TNFSF10, NOG, LIPG, RAD50, AFAP1L2, CTDSPL, NUAK1 | |  |  |  |  |  |  |  |  |  |  |  |  |  |  |  |  |  |  |  |  |  |  |  |
| Regulation Of Cartilage Development | | 7 | | 9 | 7.15E-07 | 0.029 | ADAMTS12, BMP4, BMP1, TGFB2, NOG, SNAI2, EFEMP1 | |  |  |  |  |  |  |  |  |  |  |  |  |  |  |  |  |  |  |  |  |  |  |  |
| Positive Regulation Of Apoptotic Process | | 20 | | 2 | 7.37E-07 | 0.021 | ARRB1, GATA6, FBXO32, INHBB, CLU, SCP2, BMP4, TNFSF10, SOX4, MEF2C, MAP3K1, OPTN, TIAM2, WT1, LPAR1, TGFB2, THBS1, TGM2, TIMP3, TLR3 | |  |  |  |  |  |  |  |  |  |  |  |  |  |  |  |  |  |  |  |  |  |  |  |
| Positive Regulation Of Cell Death | | 21 | | 2 | 7.73E-07 | 0.020 | ARRB1, GATA6, FBXO32, INHBB, CLU, SCP2, BMP4, TNFSF10, SOX4, MEF2C, MAP3K1, OPTN, TIAM2, WT1, LPAR1, EFNB2, TGFB2, THBS1, TGM2, TIMP3, TLR3 | |  |  |  |  |  |  |  |  |  |  |  |  |  |  |  |  |  |  |  |  |  |  |  |
| Positive Regulation Of Multicellular Organismal Process | | 35 | | 1 | 7.98E-07 | 0.016 | PLK2, MIR181B1, PLXNA1, TENM3, ADGRE5, CLU, SCN5A, BMP4, BMP1, ZMIZ1, HEG1, TRIM6, CSF1, SNAI2, VEGFA, WT1, LPAR1, EFNB2, TGFB2, THBS1, TGFBR3, TLR3, F2RL1, HAS2, HLA-A, GATA6, INHBB, ITGB3, CASK, CDON, MEF2C, NOG, LIPG, AFAP1L2, OPTN | |  |  |  |  |  |  |  |  |  |  |  |  |  |  |  |  |  |  |  |  |  |  |  |
| Positive Regulation Of Programmed Cell Death | | 20 | | 2 | 8.44E-07 | 0.021 | ARRB1, GATA6, FBXO32, INHBB, CLU, SCP2, BMP4, TNFSF10, SOX4, MEF2C, MAP3K1, OPTN, TIAM2, WT1, LPAR1, TGFB2, THBS1, TGM2, TIMP3, TLR3 | |  |  |  |  |  |  |  |  |  |  |  |  |  |  |  |  |  |  |  |  |  |  |  |
| Regulation Of Localization | | 46 | | 1 | 8.80E-07 | 0.014 | PLK2, ARRB1, ATP2B4, MIR181B1, ABCA1, CLU, BMP4, C4BPB, ARL2BP, DPYSL2, DSP, TRIM6, CPT1A, CSF1, LPAR1, F2RL1, EFEMP1, TNFRSF21, HAS2, GBP1, INHBB, ITGB3, MEF2C, MAP3K1, LAMA3, LCP1, KCNIP3, CLIC6, PLXNA1, SCN5A, SCP2, SCN9A, RAB27B, SOX4, CEMIP, MTUS1, SNAI2, VEGFA, TGFB2, THBS1, TGFBR3, CLIC3, CASK, NOG, LIPG, PARD6B | |  |  |  |  |  |  |  |  |  |  |  |  |  |  |  |  |  |  |  |  |  |  |  |
| Negative Regulation Of Developmental Process | | 26 | | 2 | 9.90E-07 | 0.018 | TNFRSF21, PLK2, HLA-A, ABCA1, GBP1, PRICKLE1, TRIB2, ITGB3, BMP4, PTPRS, ADAMTS12, ZNF608, TRIM6, NOG, SNAI2, ASAP1, VEGFA, WT1, HHIP, LPAR1, EFNB2, TGFB2, THBS1, TGFBR3, TLR3, EFEMP1 | |  |  |  |  |  |  |  |  |  |  |  |  |  |  |  |  |  |  |  |  |  |  |  |
| Regulation Of Cellular Component Movement | | 25 | | 2 | 1.06E-06 | 0.018 | PLK2, HAS2, MIR181B1, PLXNA1, ITGB3, SCN5A, SCP2, BMP4, MEF2C, CEMIP, MAP3K1, MTUS1, DSP, NOG, LAMA3, CSF1, SNAI2, PARD6B, VEGFA, LPAR1, TGFB2, THBS1, TGFBR3, F2RL1, EFEMP1 | |  |  |  |  |  |  |  |  |  |  |  |  |  |  |  |  |  |  |  |  |  |  |  |
| Regulation Of Cell Migration | | 23 | | 2 | 1.07E-06 | 0.019 | PLK2, HAS2, MIR181B1, PLXNA1, ITGB3, SCP2, BMP4, MEF2C, CEMIP, MAP3K1, MTUS1, NOG, LAMA3, CSF1, SNAI2, PARD6B, VEGFA, LPAR1, TGFB2, THBS1, TGFBR3, F2RL1, EFEMP1 | |  |  |  |  |  |  |  |  |  |  |  |  |  |  |  |  |  |  |  |  |  |  |  |
| Cell Differentiation | | 41 | | 1 | 1.14E-06 | 0.014 | RBM47, TENM3, RRBP1, BMP4, BMP1, SOX4, CYP24A1, TDRD7, ZMIZ1, HEG1, DPYSL2, DSP, SDC2, CPT1A, CSF1, SNAI2, FLG, VEGFA, WT1, NXN, LPAR1, EFNB2, TGFB2, TGFBR3, TLL1, EYA4, F2RL1, SLC7A5, ILDR2, HAS2, GATA6, INHBB, ITGB3, FAT4, CDON, MEF2C, SAMD9L, SNX10, NOG, LAMA3, TSPAN2 | |  |  |  |  |  |  |  |  |  |  |  |  |  |  |  |  |  |  |  |  |  |  |  |
| Positive Regulation Of Transferase Activity | | 19 | | 2 | 1.14E-06 | 0.021 | ARRB1, ATP2B4, TRIB2, ITGB3, CLU, BMP4, CEMIP, MAP3K1, CSF1, RAD50, AFAP1L2, VEGFA, LPAR1, TGFA, TGFB2, THBS1, TLR3, ACSL1, SERINC2 | |  |  |  |  |  |  |  |  |  |  |  |  |  |  |  |  |  |  |  |  |  |  |  |
| Trabecula Morphogenesis | | 5 | | 17 | 1.62E-06 | 0.025 | TGFB2, TGFBR3, NOG, VEGFA, HEG1 | |  |  |  |  |  |  |  |  |  |  |  |  |  |  |  |  |  |  |  |  |  |  |  |
| Morphogenesis Of An Epithelium | | 13 | | 3 | 1.74E-06 | 0.025 | MEF2C, MAP3K1, NOG, CSF1, CTSD, PLXNA1, VEGFA, WT1, HHIP, FAT4, BMP4, EFNB2, TGM2 | |  |  |  |  |  |  |  |  |  |  |  |  |  |  |  |  |  |  |  |  |  |  |  |
| Positive Regulation Of Protein Phosphorylation | | 24 | | 2 | 2.09E-06 | 0.018 | ARRB1, ATP2B4, MIR181B1, IGFBP4, INHBB, ITGB3, CLU, BMP4, CDON, CEMIP, MAP3K1, ARL2BP, TRIM6, CSF1, RAD50, AFAP1L2, VEGFA, LPAR1, TGFA, TGFB2, THBS1, TLR3, F2RL1, ACSL1 | |  |  |  |  |  |  |  |  |  |  |  |  |  |  |  |  |  |  |  |  |  |  |  |
| Regulation Of Developmental Process | | 45 | | 1 | 2.58E-06 | 0.013 | PLK2, MIR181B1, ABCA1, MYL9, ADGRE5, CLU, BMP4, BMP1, DPYSL2, TRIM6, CSF1, LPAR1, EFNB2, EFEMP1, TNFRSF21, HAS2, HLA-A, GATA6, GBP1, PRICKLE1, TRIB2, ITGB3, CDON, MEF2C, LAMA3, ASAP1, PLXNA1, TENM3, PTPRS, ZMIZ1, ZNF608, SDC2, SNAI2, VEGFA, WT1, HHIP, TGFB2, THBS1, TGFBR3, TLR3, FAT4, CASK, ADAMTS12, NOG, NUAK1 | |  |  |  |  |  |  |  |  |  |  |  |  |  |  |  |  |  |  |  |  |  |  |  |
| Platelet Degranulation | | 8 | | 6 | 2.63E-06 | 0.027 | VEGFA, ITGB3, CLU, TGFB2, THBS1, RAB27B, TIMP3, F13A1 | |  |  |  |  |  |  |  |  |  |  |  |  |  |  |  |  |  |  |  |  |  |  |  |
| Cardiac Epithelial To Mesenchymal Transition | | 5 | | 15 | 3.16E-06 | 0.024 | HAS2, BMP4, TGFB2, TGFBR3, SNAI2 | |  |  |  |  |  |  |  |  |  |  |  |  |  |  |  |  |  |  |  |  |  |  |  |
| Regulation Of Cell Motility | | 23 | | 2 | 3.22E-06 | 0.018 | PLK2, HAS2, MIR181B1, PLXNA1, ITGB3, SCP2, BMP4, MEF2C, CEMIP, MAP3K1, MTUS1, NOG, LAMA3, CSF1, SNAI2, PARD6B, VEGFA, LPAR1, TGFB2, THBS1, TGFBR3, F2RL1, EFEMP1 | |  |  |  |  |  |  |  |  |  |  |  |  |  |  |  |  |  |  |  |  |  |  |  |
| Regulation Of Locomotion | | 24 | | 1 | 3.27E-06 | 0.018 | PLK2, HAS2, MIR181B1, PLXNA1, ITGB3, SCP2, BMP4, MEF2C, CEMIP, MAP3K1, MTUS1, NOG, LAMA3, CSF1, SNAI2, PARD6B, VEGFA, LPAR1, EFNB2, TGFB2, THBS1, TGFBR3, F2RL1, EFEMP1 | |  |  |  |  |  |  |  |  |  |  |  |  |  |  |  |  |  |  |  |  |  |  |  |
| Positive Regulation Of Cellular Protein Metabolic Process | | 31 | | 1 | 3.37E-06 | 0.016 | PLK2, ARRB1, ATP2B4, MIR181B1, TSPYL5, CLU, BMP4, SOX4, CEMIP, ARL2BP, TRIM6, CSF1, SNAI2, VEGFA, LPAR1, TGFA, TGFB2, THBS1, TLR3, F2RL1, ACSL1, PRICKLE1, IGFBP4, INHBB, TRIB2, ITGB3, TNFSF10, CDON, MAP3K1, RAD50, AFAP1L2 | |  |  |  |  |  |  |  |  |  |  |  |  |  |  |  |  |  |  |  |  |  |  |  |
| Positive Regulation Of Protein Kinase Activity | | 16 | | 2 | 3.75E-06 | 0.021 | ARRB1, CEMIP, MAP3K1, ATP2B4, CSF1, AFAP1L2, VEGFA, ITGB3, CLU, LPAR1, BMP4, TGFA, TGFB2, THBS1, TLR3, ACSL1 | |  |  |  |  |  |  |  |  |  |  |  |  |  |  |  |  |  |  |  |  |  |  |  |
| Regulation Of Epithelial Cell Proliferation | | 14 | | 3 | 3.93E-06 | 0.023 | MEF2C, HAS2, NOG, SNAI2, VEGFA, ITGB3, SCN5A, BMP4, EFNB2, TGFA, TGFB2, THBS1, TGFBR3, CASK | |  |  |  |  |  |  |  |  |  |  |  |  |  |  |  |  |  |  |  |  |  |  |  |
| Positive Regulation Of Protein Metabolic Process | | 32 | | 1 | 3.94E-06 | 0.015 | PLK2, ARRB1, ATP2B4, MIR181B1, TSPYL5, CLU, BMP4, C4BPB, SOX4, CEMIP, ARL2BP, TRIM6, CSF1, SNAI2, VEGFA, LPAR1, TGFA, TGFB2, THBS1, TLR3, F2RL1, ACSL1, PRICKLE1, IGFBP4, INHBB, TRIB2, ITGB3, TNFSF10, CDON, MAP3K1, RAD50, AFAP1L2 | |  |  |  |  |  |  |  |  |  |  |  |  |  |  |  |  |  |  |  |  |  |  |  |
| Positive Regulation Of Kinase Activity | | 17 | | 2 | 4.08E-06 | 0.021 | ARRB1, ATP2B4, ITGB3, CLU, BMP4, CEMIP, MAP3K1, CSF1, RAD50, AFAP1L2, VEGFA, LPAR1, TGFA, TGFB2, THBS1, TLR3, ACSL1 | |  |  |  |  |  |  |  |  |  |  |  |  |  |  |  |  |  |  |  |  |  |  |  |
| Regulation Of Multicellular Organismal Development | | 38 | | 1 | 4.28E-06 | 0.014 | PLK2, MIR181B1, MYL9, PLXNA1, TENM3, ADGRE5, BMP4, BMP1, PTPRS, ZMIZ1, DPYSL2, ZNF608, SDC2, CSF1, SNAI2, VEGFA, WT1, HHIP, LPAR1, EFNB2, TGFB2, THBS1, TGFBR3, TLR3, EFEMP1, TNFRSF21, HLA-A, GATA6, PRICKLE1, ITGB3, FAT4, CASK, CDON, ADAMTS12, MEF2C, NOG, LAMA3, ASAP1 | |  |  |  |  |  |  |  |  |  |  |  |  |  |  |  |  |  |  |  |  |  |  |  |
| Positive Regulation Of Phosphorus Metabolic Process | | 25 | | 1 | 4.44E-06 | 0.017 | ARRB1, ATP2B4, MIR181B1, IGFBP4, INHBB, ITGB3, CLU, BMP4, CDON, MEF2C, CEMIP, MAP3K1, ARL2BP, TRIM6, CSF1, RAD50, AFAP1L2, VEGFA, LPAR1, TGFA, TGFB2, THBS1, TLR3, F2RL1, ACSL1 | |  |  |  |  |  |  |  |  |  |  |  |  |  |  |  |  |  |  |  |  |  |  |  |
| Positive Regulation Of Phosphate Metabolic Process | | 25 | | 1 | 4.44E-06 | 0.017 | ARRB1, ATP2B4, MIR181B1, IGFBP4, INHBB, ITGB3, CLU, BMP4, CDON, MEF2C, CEMIP, MAP3K1, ARL2BP, TRIM6, CSF1, RAD50, AFAP1L2, VEGFA, LPAR1, TGFA, TGFB2, THBS1, TLR3, F2RL1, ACSL1 | |  |  |  |  |  |  |  |  |  |  |  |  |  |  |  |  |  |  |  |  |  |  |  |
| Cell Adhesion | | 21 | | 2 | 4.57E-06 | 0.019 | MYL9, CD22, TENM3, ADGRE5, CDH11, ITGB3, BAIAP2L1, FAT4, PTPRS, CASK, CDON, ADAMTS12, DSP, PDLIM1, LAMA3, MPZL3, EFNB2, TGFB2, THBS1, NUAK1, SLAMF7 | |  |  |  |  |  |  |  |  |  |  |  |  |  |  |  |  |  |  |  |  |  |  |  |
| Cellular Response To Growth Factor Stimulus | | 13 | | 3 | 4.62E-06 | 0.023 | ADAMTS12, MEF2C, HAS2, GATA6, NOG, SNAI2, LTBP1, OPTN, VEGFA, ITGB3, CLU, BMP4, THBS1 | |  |  |  |  |  |  |  |  |  |  |  |  |  |  |  |  |  |  |  |  |  |  |  |
| Positive Regulation Of Phosphorylation | | 24 | | 1 | 4.65E-06 | 0.017 | ARRB1, ATP2B4, MIR181B1, IGFBP4, INHBB, ITGB3, CLU, BMP4, CDON, CEMIP, MAP3K1, ARL2BP, TRIM6, CSF1, RAD50, AFAP1L2, VEGFA, LPAR1, TGFA, TGFB2, THBS1, TLR3, F2RL1, ACSL1 | |  |  |  |  |  |  |  |  |  |  |  |  |  |  |  |  |  |  |  |  |  |  |  |
| Regulation Of Glomerulus Development | | 4 | | 23 | 5.07E-06 | 0.021 | BMP4, NOG, ITGB3, WT1 | |  |  |  |  |  |  |  |  |  |  |  |  |  |  |  |  |  |  |  |  |  |  |  |
| Regulation Of Kidney Development | | 6 | | 9 | 5.66E-06 | 0.026 | VEGFA, ITGB3, WT1, FAT4, BMP4, NOG | |  |  |  |  |  |  |  |  |  |  |  |  |  |  |  |  |  |  |  |  |  |  |  |
| Mesenchymal Cell Differentiation | | 7 | | 7 | 5.77E-06 | 0.026 | MEF2C, HAS2, BMP4, TGFB2, TGFBR3, NOG, SNAI2 | |  |  |  |  |  |  |  |  |  |  |  |  |  |  |  |  |  |  |  |  |  |  |  |
| Cellular Response To Organic Substance | | 34 | | 1 | 6.22E-06 | 0.015 | ATP2B4, ABCA1, FBXO32, CLU, SCP2, BMP4, SOX4, TRIM6, CPT1A, SNAI2, VEGFA, WT1, LPAR1, THBS1, TIMP3, TLR3, FBP1, TNFRSF21, HAS2, HLA-A, GATA6, GBP1, GBP2, GBP3, LAPTM5, INHBB, ITGB3, CPEB4, ADAMTS12, MEF2C, SNX10, NOG, LTBP1, OPTN | |  |  |  |  |  |  |  |  |  |  |  |  |  |  |  |  |  |  |  |  |  |  |  |
| Response To Gonadotropin | | 6 | | 8 | 7.41E-06 | 0.025 | RAD50, INHBB, WT1, SCP2, GATA6, TGFBR3 | |  |  |  |  |  |  |  |  |  |  |  |  |  |  |  |  |  |  |  |  |  |  |  |
| Response To Chemical | | 52 | | 1 | 7.66E-06 | 0.012 | ARRB1, ATP2B4, ABCA1, AK4, FBXO32, PTGR1, CLU, BMP4, CYP24A1, DPYSL2, TRIM6, CPT1A, LPAR1, ACSL1, FBP1, TNFRSF21, HAS2, HLA-A, GATA6, GBP1, GBP2, GBP3, INHBB, ITGB3, MEF2C, SNX10, LTBP1, PON2, SCN5A, SCP2, SCN9A, SOX4, SDC2, SNAI2, VEGFA, WT1, TGFA, TGFB2, THBS1, TGFBR3, TIMP3, TLR3, ILDR2, LAPTM5, CPEB4, TNFSF10, ADAMTS12, PTGES, NOG, LIPG, RAD50, OPTN | |  |  |  |  |  |  |  |  |  |  |  |  |  |  |  |  |  |  |  |  |  |  |  |
| Extracellular Structure Organization | | 13 | | 3 | 8.04E-06 | 0.022 | CRISPLD2, HAS2, ABCA1, LAMA3, LCP1, LIPG, MPZL3, ITGB3, WT1, BMP1, TGFB2, THBS1, TLL1 | |  |  |  |  |  |  |  |  |  |  |  |  |  |  |  |  |  |  |  |  |  |  |  |
| Regulation Of Cell Proliferation | | 33 | | 1 | 9.98E-06 | 0.015 | ARRB1, MIR181B1, TSPYL5, CLU, SCN5A, SCP2, BMP4, SOX4, ZMIZ1, CSF1, SNAI2, VEGFA, WT1, EFNB2, TGFA, TGFB2, THBS1, TGFBR3, TGM2, EFEMP1, TNFRSF21, HAS2, HLA-A, TNS3, GATA6, ITGB3, CASK, CDON, PTGES, MEF2C, NOG, OPTN, NUAK1 | |  |  |  |  |  |  |  |  |  |  |  |  |  |  |  |  |  |  |  |  |  |  |  |
| Positive Regulation Of Signal Transduction | | 30 | | 1 | 1.04E-05 | 0.015 | PLK2, ARRB1, MIR181B1, TSPYL5, CLU, BMP4, SOX4, SLC44A2, ZMIZ1, ARL2BP, TRIM6, CSF1, VEGFA, LPAR1, TGFA, TGFB2, THBS1, TGFBR3, TGM2, TIMP3, TLR3, F2RL1, GATA6, IGFBP4, INHBB, ITGB3, TNFSF10, CDON, MAP3K1, AFAP1L2 | |  |  |  |  |  |  |  |  |  |  |  |  |  |  |  |  |  |  |  |  |  |  |  |
| Regulation Of Cytokine Production | | 18 | | 2 | 1.09E-05 | 0.019 | TNFRSF21, ARRB1, HLA-A, GATA6, GBP1, INHBB, TRIB2, CLU, PTPRS, HEG1, TRIM6, SNAI2, AFAP1L2, OPTN, TGFB2, THBS1, TLR3, F2RL1 | |  |  |  |  |  |  |  |  |  |  |  |  |  |  |  |  |  |  |  |  |  |  |  |
| Positive Regulation Of Response To Stimulus | | 40 | | 1 | 1.09E-05 | 0.013 | PLK2, ARRB1, MIR181B1, TSPYL5, CLU, BMP4, C4BPB, SOX4, SLC44A2, ZMIZ1, ARL2BP, TRIM6, CSF1, SNAI2, VEGFA, LPAR1, TGFA, TGFB2, THBS1, TGFBR3, TGM2, TIMP3, EYA4, TLR3, F2RL1, TNFRSF21, HLA-A, HLA-B, GATA6, IGFBP4, INHBB, ITGB3, HLA-DRA, TNFSF10, CDON, MEF2C, MAP3K1, PLPP4, AFAP1L2, OPTN | |  |  |  |  |  |  |  |  |  |  |  |  |  |  |  |  |  |  |  |  |  |  |  |
| Negative Regulation Of Protein Metabolic Process | | 25 | | 1 | 1.13E-05 | 0.016 | ARRB1, ATP2B4, MIR181B1, GBP1, INHBB, TRIB2, ITGB3, CPEB4, GPRC5A, CLU, BMP4, CST7, C4BPB, SH3RF2, SOX4, NOG, PMEPA1, CST4, CTDSPL, VEGFA, WT1, NXN, THBS1, TIMP3, F2RL1 | |  |  |  |  |  |  |  |  |  |  |  |  |  |  |  |  |  |  |  |  |  |  |  |
| Regulation Of Cardioblast Differentiation | | 3 | | 42 | 1.14E-05 | 0.017 | TGFB2, GATA6, PRICKLE1 | |  |  |  |  |  |  |  |  |  |  |  |  |  |  |  |  |  |  |  |  |  |  |  |
| Cellular Response To Interferon-Beta | | 5 | | 11 | 1.21E-05 | 0.023 | TRIM6, GBP1, GBP2, GBP3, TLR3 | |  |  |  |  |  |  |  |  |  |  |  |  |  |  |  |  |  |  |  |  |  |  |  |
| Adhesion Of Symbiont To Host | | 4 | | 19 | 1.25E-05 | 0.021 | GBP1, GBP2, GBP3, INHBB | |  |  |  |  |  |  |  |  |  |  |  |  |  |  |  |  |  |  |  |  |  |  |  |
| Coronary Vasculature Development | | 5 | | 11 | 1.36E-05 | 0.023 | WT1, BMP4, TGFBR3, PRICKLE1, LTBP1 | |  |  |  |  |  |  |  |  |  |  |  |  |  |  |  |  |  |  |  |  |  |  |  |
| Regulation Of Cellular Component Organization | | 40 | | 1 | 1.41E-05 | 0.013 | PLK2, ARRB1, ABCA1, PLXNA1, TENM3, ADGRE5, BAIAP2L1, CLU, BMP4, PTPRS, C4BPB, DPYSL2, SDC2, PMEPA1, SNAI2, VEGFA, WT1, LPAR1, EFNB2, TGFA, TGFB2, THBS1, F2RL1, EFEMP1, FBP1, HAS2, HLA-A, GBP1, EVI5, IGFBP4, ITGB3, COLGALT1, CASK, TNFSF10, MEF2C, MAP3K1, LCP1, ASAP1, RAD50, OPTN | |  |  |  |  |  |  |  |  |  |  |  |  |  |  |  |  |  |  |  |  |  |  |  |
| Response To Growth Factor | | 13 | | 2 | 1.67E-05 | 0.021 | ADAMTS12, MEF2C, HAS2, GATA6, NOG, SNAI2, LTBP1, OPTN, VEGFA, ITGB3, CLU, BMP4, THBS1 | |  |  |  |  |  |  |  |  |  |  |  |  |  |  |  |  |  |  |  |  |  |  |  |
| Negative Regulation Of Chondrocyte Differentiation | | 4 | | 17 | 1.83E-05 | 0.020 | BMP4, ADAMTS12, SNAI2, EFEMP1 | |  |  |  |  |  |  |  |  |  |  |  |  |  |  |  |  |  |  |  |  |  |  |  |
| Positive Regulation Of Kidney Development | | 5 | | 10 | 1.89E-05 | 0.023 | VEGFA, ITGB3, WT1, BMP4, NOG | |  |  |  |  |  |  |  |  |  |  |  |  |  |  |  |  |  |  |  |  |  |  |  |
| Regulation Of Cell Development | | 22 | | 1 | 1.89E-05 | 0.017 | TNFRSF21, PLK2, HAS2, HLA-A, GBP1, PLXNA1, TENM3, ITGB3, BMP4, PTPRS, CASK, CDON, MEF2C, DPYSL2, SDC2, NOG, CSF1, ASAP1, VEGFA, LPAR1, EFNB2, EFEMP1 | |  |  |  |  |  |  |  |  |  |  |  |  |  |  |  |  |  |  |  |  |  |  |  |
| Regulation Of Mapk Cascade | | 19 | | 2 | 2.07E-05 | 0.018 | ARRB1, MIR181B1, GBP1, IGFBP4, INHBB, TRIB2, ITGB3, BMP4, CDON, MAP3K1, VEGFA, LPAR1, TGFA, TGFB2, THBS1, TGFBR3, TIMP3, TLR3, F2RL1 | |  |  |  |  |  |  |  |  |  |  |  |  |  |  |  |  |  |  |  |  |  |  |  |
| Epithelial To Mesenchymal Transition | | 6 | | 7 | 2.07E-05 | 0.024 | HAS2, BMP4, TGFB2, TGFBR3, NOG, SNAI2 | |  |  |  |  |  |  |  |  |  |  |  |  |  |  |  |  |  |  |  |  |  |  |  |
| Regulation Of Cardiocyte Differentiation | | 6 | | 7 | 2.07E-05 | 0.024 | MEF2C, BMP4, EFNB2, TGFB2, GATA6, PRICKLE1 | |  |  |  |  |  |  |  |  |  |  |  |  |  |  |  |  |  |  |  |  |  |  |  |
| Response To Organic Substance | | 45 | | 1 | 2.12E-05 | 0.012 | ATP2B4, ABCA1, FBXO32, CLU, BMP4, CYP24A1, DPYSL2, TRIM6, CPT1A, LPAR1, ACSL1, FBP1, TNFRSF21, HAS2, HLA-A, GATA6, GBP1, GBP2, GBP3, INHBB, ITGB3, MEF2C, SNX10, LTBP1, SCN5A, SCP2, SOX4, SDC2, SNAI2, VEGFA, WT1, TGFB2, THBS1, TGFBR3, TIMP3, TLR3, ILDR2, LAPTM5, CPEB4, TNFSF10, ADAMTS12, PTGES, NOG, RAD50, OPTN | |  |  |  |  |  |  |  |  |  |  |  |  |  |  |  |  |  |  |  |  |  |  |  |
| Organonitrogen Compound Metabolic Process | | 69 | | 1 | 2.26E-05 | 0.011 | ARRB1, ABCA1, AK4, FBXO32, MARCH3, CLU, BMP4, BMP1, C4BPB, DSP, TRIM6, CPT1A, CSF1, CTSD, FLG, EFNB2, EYA4, F13A1, ACSL1, EFEMP1, HAS2, GATA6, IGFBP4, SH3RF2, MEF2C, MAP3K1, LTBP1, SLC37A2, HECTD4, TMPRSS15, MAMDC2, RRBP1, SCP2, PTPRS, SDC2, UGCG, VEGFA, TGFB2, THBS1, TGFBR3, TGM2, TLL1, TLR3, SERINC2, SLC7A5, CDC42BPA, CASK, PTGES, CTDSPL, NUAK1, PLK2, PDE10A, MGAT4A, PRSS23, TRIB2, CPA4, CHST15, LPCAT2, TENM3, KLHL4, CPPED1, SLC44A2, CEMIP, PORCN, CDK15, ELOVL7, CPEB4, ADAMTS12, APOL6 | |  |  |  |  |  |  |  |  |  |  |  |  |  |  |  |  |  |  |  |  |  |  |  |
| Animal Organ Development | | 31 | | 1 | 2.35E-05 | 0.014 | AK4, BMP4, BMP1, PTPRS, SOX4, HEG1, DPYSL2, DSP, CSF1, VEGFA, WT1, LPAR1, TGFB2, THBS1, TGFBR3, TLR3, EFEMP1, ILDR2, HAS2, GATA6, INHBB, FAT4, TNFSF10, MEF2C, MAP3K1, CRISPLD2, SAMD9L, NOG, LCP1, TSPAN2, RAD50 | |  |  |  |  |  |  |  |  |  |  |  |  |  |  |  |  |  |  |  |  |  |  |  |
| Osteoblast Differentiation | | 7 | | 5 | 2.43E-05 | 0.024 | CYP24A1, MEF2C, RRBP1, BMP4, TGFBR3, NOG, SNAI2 | |  |  |  |  |  |  |  |  |  |  |  |  |  |  |  |  |  |  |  |  |  |  |  |
| Positive Regulation Of Cardiocyte Differentiation | | 5 | | 10 | 2.56E-05 | 0.023 | MEF2C, BMP4, EFNB2, TGFB2, GATA6 | |  |  |  |  |  |  |  |  |  |  |  |  |  |  |  |  |  |  |  |  |  |  |  |
| Heart Trabecula Morphogenesis | | 4 | | 15 | 2.58E-05 | 0.020 | TGFB2, TGFBR3, NOG, HEG1 | |  |  |  |  |  |  |  |  |  |  |  |  |  |  |  |  |  |  |  |  |  |  |  |
| Regulation Of Cell Death | | 32 | | 1 | 2.58E-05 | 0.014 | PLK2, ARRB1, FBXO32, CLU, SCP2, BMP4, SOX4, SNAI2, TIAM2, VEGFA, WT1, HHIP, LPAR1, EFNB2, TGFA, TGFB2, THBS1, TGFBR3, TGM2, TIMP3, EYA4, TLR3, GATA6, INHBB, ITGB3, CPEB4, TNFSF10, MEF2C, MAP3K1, NOG, KCNIP3, OPTN | |  |  |  |  |  |  |  |  |  |  |  |  |  |  |  |  |  |  |  |  |  |  |  |
| Ventricular Compact Myocardium Morphogenesis | | 3 | | 33 | 2.72E-05 | 0.016 | TGFBR3, NOG, DSP | |  |  |  |  |  |  |  |  |  |  |  |  |  |  |  |  |  |  |  |  |  |  |  |
| Blood Vessel Remodeling | | 5 | | 9 | 2.82E-05 | 0.022 | MEF2C, VEGFA, TGFB2, TGFBR3, TGM2 | |  |  |  |  |  |  |  |  |  |  |  |  |  |  |  |  |  |  |  |  |  |  |  |
| Regulation Of Apoptotic Process | | 30 | | 1 | 2.87E-05 | 0.014 | PLK2, ARRB1, FBXO32, CLU, SCP2, BMP4, SOX4, SNAI2, TIAM2, VEGFA, WT1, HHIP, LPAR1, TGFA, TGFB2, THBS1, TGFBR3, TGM2, TIMP3, EYA4, TLR3, GATA6, INHBB, CPEB4, TNFSF10, MEF2C, MAP3K1, NOG, KCNIP3, OPTN | |  |  |  |  |  |  |  |  |  |  |  |  |  |  |  |  |  |  |  |  |  |  |  |
| Positive Regulation Of Mesonephros Development | | 4 | | 15 | 3.03E-05 | 0.020 | BMP4, NOG, VEGFA, WT1 | |  |  |  |  |  |  |  |  |  |  |  |  |  |  |  |  |  |  |  |  |  |  |  |
| Positive Regulation Of Epithelial Cell Migration | | 8 | | 4 | 3.42E-05 | 0.023 | PLK2, VEGFA, HAS2, ITGB3, BMP4, TGFB2, THBS1, SNAI2 | |  |  |  |  |  |  |  |  |  |  |  |  |  |  |  |  |  |  |  |  |  |  |  |
| Regulation Of Neuron Projection Development | | 15 | | 2 | 3.43E-05 | 0.019 | PLK2, HLA-A, PLXNA1, TENM3, BMP4, PTPRS, CASK, MEF2C, DPYSL2, SDC2, ASAP1, VEGFA, LPAR1, EFNB2, EFEMP1 | |  |  |  |  |  |  |  |  |  |  |  |  |  |  |  |  |  |  |  |  |  |  |  |
| Regulation Of Programmed Cell Death | | 30 | | 1 | 3.52E-05 | 0.014 | PLK2, ARRB1, FBXO32, CLU, SCP2, BMP4, SOX4, SNAI2, TIAM2, VEGFA, WT1, HHIP, LPAR1, TGFA, TGFB2, THBS1, TGFBR3, TGM2, TIMP3, EYA4, TLR3, GATA6, INHBB, CPEB4, TNFSF10, MEF2C, MAP3K1, NOG, KCNIP3, OPTN | |  |  |  |  |  |  |  |  |  |  |  |  |  |  |  |  |  |  |  |  |  |  |  |
| Blood Vessel Development | | 8 | | 4 | 3.56E-05 | 0.023 | MEF2C, VEGFA, WT1, BMP4, TGFB2, TGFBR3, PRICKLE1, LTBP1 | |  |  |  |  |  |  |  |  |  |  |  |  |  |  |  |  |  |  |  |  |  |  |  |
| Cardiac Septum Morphogenesis | | 6 | | 6 | 3.57E-05 | 0.023 | SOX4, BMP4, TGFB2, GATA6, TGFBR3, NOG | |  |  |  |  |  |  |  |  |  |  |  |  |  |  |  |  |  |  |  |  |  |  |  |
| Cell Junction Organization | | 9 | | 3 | 3.59E-05 | 0.023 | HEG1, DSP, LAMA3, SNAI2, PARD6B, CDH11, ITGB3, TGFB2, F2RL1 | |  |  |  |  |  |  |  |  |  |  |  |  |  |  |  |  |  |  |  |  |  |  |  |
| Membranous Septum Morphogenesis | | 3 | | 30 | 3.86E-05 | 0.016 | BMP4, TGFB2, NOG | |  |  |  |  |  |  |  |  |  |  |  |  |  |  |  |  |  |  |  |  |  |  |  |
| Pharyngeal Arch Artery Morphogenesis | | 3 | | 30 | 3.86E-05 | 0.016 | BMP4, TGFB2, NOG | |  |  |  |  |  |  |  |  |  |  |  |  |  |  |  |  |  |  |  |  |  |  |  |
| Ventricular Cardiac Muscle Tissue Morphogenesis | | 5 | | 9 | 4.09E-05 | 0.022 | HEG1, DSP, TGFB2, TGFBR3, NOG | |  |  |  |  |  |  |  |  |  |  |  |  |  |  |  |  |  |  |  |  |  |  |  |
| Negative Regulation Of Cellular Response To Growth Factor Stimulus | | 8 | | 4 | 4.18E-05 | 0.023 | ADAMTS12, SHISA2, THBS1, TGFBR3, NOG, CASK, PMEPA1, LTBP1 | |  |  |  |  |  |  |  |  |  |  |  |  |  |  |  |  |  |  |  |  |  |  |  |
| Biological Regulation | | 137 | | 0 | 4.49E-05 | 0.008 | ARRB1, ATP2B4, ABCA1, CD22, ADGRE5, CLU, BMP4, BMP1, C4BPB, CYP24A1, DPYSL2, DSP, CPT1A, CSF1, CST4, FLG, LPAR1, EFNB2, EYA4, F2RL1, F13A1, ACSL1, EFEMP1, FBP1, GPR1, HAS2, HLA-A, HLA-B, GATA6, GBP1, GBP2, IGFBP4, INHBB, ITGB3, CD82, HLA-DRA, MEF2C, MEIS3P1, MAP3K1, LAMA3, LCP1, LTBP1, PLXNA1, RRBP1, SCN5A, SCP2, SCN9A, PTPRS, RAB27B, SOX4, SDC2, SNAI2, TUFT1, VEGFA, WT1, TGFA, TGFB2, THBS1, TGFBR3, TGM2, TIMP3, TLR3, ZNF43, EVI5, CLIC3, GPRC5A, SORBS2, CDC42BPA, CST7, CASK, TNFSF10, PTGES, CYTIP, FCGR2C, PDLIM1, NOG, LIPG, TSPAN2, RAD50, OPTN, MBNL2, CTDSPL, NUAK1, PLK2, PDE10A, MYL9, TDRD7, ARL2BP, TIAM2, TNFRSF21, TRIB2, CDON, SNX10, KCNIP3, ASAP1, CLIC6, TENM3, BAIAP2L1, ACOXL, SLC44A2, ZMIZ1, CEMIP, HEG1, MTUS1, ZNF608, SORCS2, PMEPA1, NXN, HHIP, SLAMF7, TNS3, PORCN, CDK15, CPEB4, FAT4, COLGALT1, ADAMTS12, PARD6B, AFAP1L2, TSPYL5, FBXO32, TRIM6, OR5P2, LYPD6B, PRICKLE1, SH3RF2, SAMD9L, PLPP4, HECTD4, ADGRF1, ZNF391, SERINC2, ILDR2, SHISA2, MIR181B1, UCA1, MIR604 | |  |  |  |  |  |  |  |  |  |  |  |  |  |  |  |  |  |  |  |  |  |  |  |
| Signal Transduction | | 73 | | 1 | 4.53E-05 | 0.010 | ARRB1, ABCA1, CD22, ADGRE5, CLU, BMP4, CYP24A1, DPYSL2, OR5P2, CSF1, LPAR1, EFNB2, F2RL1, F13A1, ACSL1, EFEMP1, GPR1, HLA-A, HLA-B, GBP1, GBP2, PRICKLE1, IGFBP4, INHBB, ITGB3, CD82, HLA-DRA, MEF2C, MAP3K1, LCP1, LTBP1, PLPP4, ADGRF1, PLXNA1, RRBP1, RAB27B, SOX4, SDC2, SNAI2, TUFT1, VEGFA, TGFA, TGFB2, TGFBR3, TGM2, TLR3, CLIC3, GPRC5A, SORBS2, CDC42BPA, TNFSF10, PTGES, FCGR2C, NOG, TSPAN2, OPTN, NUAK1, PLK2, PDE10A, ARL2BP, TIAM2, TNFRSF21, CDON, KCNIP3, TENM3, SORCS2, PMEPA1, NXN, HHIP, TNS3, PORCN, CPEB4, FAT4 | |  |  |  |  |  |  |  |  |  |  |  |  |  |  |  |  |  |  |  |  |  |  |  |
| Regulation Of Mesonephros Development | | 4 | | 13 | 4.74E-05 | 0.020 | BMP4, NOG, VEGFA, WT1 | |  |  |  |  |  |  |  |  |  |  |  |  |  |  |  |  |  |  |  |  |  |  |  |
| Negative Regulation Of Cardiac Epithelial To Mesenchymal Transition | | 2 | | 100 | 4.84E-05 | 0.011 | TGFB2, NOG | |  |  |  |  |  |  |  |  |  |  |  |  |  |  |  |  |  |  |  |  |  |  |  |
| Negative Regulation Of Epithelial To Mesenchymal Transition Involved In Endocardial Cushion Formation | | 2 | | 100 | 4.84E-05 | 0.011 | TGFB2, NOG | |  |  |  |  |  |  |  |  |  |  |  |  |  |  |  |  |  |  |  |  |  |  |  |
| Regulation Of Protein Kinase Activity | | 18 | | 2 | 4.85E-05 | 0.017 | ARRB1, ATP2B4, TRIB2, ITGB3, GPRC5A, CLU, BMP4, CEMIP, MAP3K1, CSF1, AFAP1L2, VEGFA, LPAR1, TGFA, TGFB2, THBS1, TLR3, ACSL1 | |  |  |  |  |  |  |  |  |  |  |  |  |  |  |  |  |  |  |  |  |  |  |  |
| Response To Interferon-Beta | | 5 | | 8 | 4.87E-05 | 0.022 | TRIM6, GBP1, GBP2, GBP3, TLR3 | |  |  |  |  |  |  |  |  |  |  |  |  |  |  |  |  |  |  |  |  |  |  |  |
| Morphogenesis Of A Branching Epithelium | | 8 | | 4 | 5.07E-05 | 0.023 | VEGFA, WT1, HHIP, FAT4, BMP4, TGM2, CSF1, CTSD | |  |  |  |  |  |  |  |  |  |  |  |  |  |  |  |  |  |  |  |  |  |  |  |
| Vasculogenesis | | 6 | | 6 | 5.18E-05 | 0.023 | ZMIZ1, VEGFA, HEG1, HAS2, WT1, TGFBR3 | |  |  |  |  |  |  |  |  |  |  |  |  |  |  |  |  |  |  |  |  |  |  |  |
| Muscle Tissue Morphogenesis | | 6 | | 6 | 5.18E-05 | 0.023 | HEG1, DSP, BMP4, TGFB2, TGFBR3, NOG | |  |  |  |  |  |  |  |  |  |  |  |  |  |  |  |  |  |  |  |  |  |  |  |
| Negative Regulation Of Cytokine Production | | 10 | | 3 | 5.31E-05 | 0.021 | TNFRSF21, ARRB1, GATA6, GBP1, INHBB, TRIB2, PTPRS, TGFB2, THBS1, F2RL1 | |  |  |  |  |  |  |  |  |  |  |  |  |  |  |  |  |  |  |  |  |  |  |  |
| Regulation Of Dendritic Spine Development | | 6 | | 6 | 5.49E-05 | 0.023 | PLK2, MEF2C, LPAR1, PTPRS, CASK, ASAP1 | |  |  |  |  |  |  |  |  |  |  |  |  |  |  |  |  |  |  |  |  |  |  |  |
| Extracellular Matrix Organization | | 11 | | 3 | 5.55E-05 | 0.021 | CRISPLD2, HAS2, LAMA3, LCP1, MPZL3, ITGB3, WT1, BMP1, TGFB2, THBS1, TLL1 | |  |  |  |  |  |  |  |  |  |  |  |  |  |  |  |  |  |  |  |  |  |  |  |
| Positive Regulation Of Cellular Component Organization | | 24 | | 1 | 5.67E-05 | 0.015 | ARRB1, PLXNA1, TENM3, ADGRE5, BAIAP2L1, CLU, BMP4, SNAI2, VEGFA, LPAR1, TGFA, TGFB2, F2RL1, EFEMP1, HAS2, ITGB3, COLGALT1, CASK, TNFSF10, MAP3K1, LCP1, ASAP1, RAD50, OPTN | |  |  |  |  |  |  |  |  |  |  |  |  |  |  |  |  |  |  |  |  |  |  |  |
| Regulation Of Extrinsic Apoptotic Signaling Pathway | | 8 | | 4 | 5.88E-05 | 0.022 | VEGFA, BMP4, TGFB2, THBS1, TIMP3, EYA4, SNAI2, TNFSF10 | |  |  |  |  |  |  |  |  |  |  |  |  |  |  |  |  |  |  |  |  |  |  |  |
| Positive Regulation Of Intracellular Signal Transduction | | 22 | | 1 | 5.95E-05 | 0.016 | PLK2, ARRB1, MIR181B1, TSPYL5, IGFBP4, ITGB3, CLU, BMP4, TNFSF10, CDON, SLC44A2, MAP3K1, ARL2BP, CSF1, VEGFA, LPAR1, TGFA, TGFB2, THBS1, TGM2, TLR3, F2RL1 | |  |  |  |  |  |  |  |  |  |  |  |  |  |  |  |  |  |  |  |  |  |  |  |
| Regulation Of Kinase Activity | | 19 | | 1 | 6.28E-05 | 0.017 | ARRB1, ATP2B4, TRIB2, ITGB3, GPRC5A, CLU, BMP4, CEMIP, MAP3K1, CSF1, RAD50, AFAP1L2, VEGFA, LPAR1, TGFA, TGFB2, THBS1, TLR3, ACSL1 | |  |  |  |  |  |  |  |  |  |  |  |  |  |  |  |  |  |  |  |  |  |  |  |
| Regulation Of Epithelial Cell Migration | | 10 | | 3 | 6.60E-05 | 0.021 | PLK2, MEF2C, HAS2, SNAI2, VEGFA, ITGB3, BMP4, TGFB2, THBS1, TGFBR3 | |  |  |  |  |  |  |  |  |  |  |  |  |  |  |  |  |  |  |  |  |  |  |  |
| Regulation Of Neurogenesis | | 19 | | 1 | 6.89E-05 | 0.016 | TNFRSF21, PLK2, HLA-A, PLXNA1, TENM3, BMP4, PTPRS, CASK, CDON, MEF2C, DPYSL2, SDC2, NOG, CSF1, ASAP1, VEGFA, LPAR1, EFNB2, EFEMP1 | |  |  |  |  |  |  |  |  |  |  |  |  |  |  |  |  |  |  |  |  |  |  |  |
| Cellular Developmental Process | | 46 | | 1 | 6.95E-05 | 0.012 | CDH11, CLU, BMP4, BMP1, CYP24A1, TDRD7, DPYSL2, DSP, CPT1A, CSF1, FLG, LPAR1, EFNB2, EYA4, F2RL1, HAS2, GATA6, INHBB, ITGB3, CDON, MEF2C, MAP3K1, SAMD9L, SNX10, LAMA3, RBM47, TENM3, RRBP1, PTPRS, SOX4, ZMIZ1, HEG1, SDC2, SNAI2, VEGFA, WT1, NXN, TGFB2, TGFBR3, TLL1, SLC7A5, ILDR2, FAT4, NOG, TSPAN2, PARD6B | |  |  |  |  |  |  |  |  |  |  |  |  |  |  |  |  |  |  |  |  |  |  |  |
| Regulation Of Glomerular Mesangial Cell Proliferation | | 3 | | 25 | 7.01E-05 | 0.016 | BMP4, ITGB3, WT1 | |  |  |  |  |  |  |  |  |  |  |  |  |  |  |  |  |  |  |  |  |  |  |  |
| Cellular Response To Chemical Stimulus | | 35 | | 1 | 7.18E-05 | 0.013 | ATP2B4, ABCA1, FBXO32, CLU, SCN5A, SCP2, BMP4, SOX4, TRIM6, CPT1A, SNAI2, VEGFA, WT1, LPAR1, THBS1, TIMP3, TLR3, FBP1, TNFRSF21, HAS2, HLA-A, GATA6, GBP1, GBP2, GBP3, LAPTM5, INHBB, ITGB3, CPEB4, ADAMTS12, MEF2C, SNX10, NOG, LTBP1, OPTN | |  |  |  |  |  |  |  |  |  |  |  |  |  |  |  |  |  |  |  |  |  |  |  |
| Morphogenesis Of A Branching Structure | | 8 | | 4 | 7.32E-05 | 0.022 | VEGFA, WT1, HHIP, FAT4, BMP4, TGM2, CSF1, CTSD | |  |  |  |  |  |  |  |  |  |  |  |  |  |  |  |  |  |  |  |  |  |  |  |
| Cellular Response To Tumor Necrosis Factor | | 8 | | 4 | 7.58E-05 | 0.022 | TNFRSF21, ADAMTS12, OPTN, HAS2, GBP1, THBS1, GBP2, GBP3 | |  |  |  |  |  |  |  |  |  |  |  |  |  |  |  |  |  |  |  |  |  |  |  |
| Regulation Of Nervous System Development | | 20 | | 1 | 7.70E-05 | 0.016 | TNFRSF21, PLK2, HLA-A, PLXNA1, TENM3, ADGRE5, BMP4, PTPRS, CASK, CDON, MEF2C, DPYSL2, SDC2, NOG, CSF1, ASAP1, VEGFA, LPAR1, EFNB2, EFEMP1 | |  |  |  |  |  |  |  |  |  |  |  |  |  |  |  |  |  |  |  |  |  |  |  |
| Positive Regulation Of Developmental Process | | 27 | | 1 | 8.11E-05 | 0.014 | PLK2, MIR181B1, PLXNA1, TENM3, ADGRE5, CLU, BMP4, BMP1, ZMIZ1, CSF1, SNAI2, VEGFA, WT1, LPAR1, EFNB2, TGFB2, THBS1, TGFBR3, TLR3, HAS2, HLA-A, GATA6, ITGB3, CASK, CDON, MEF2C, NOG | |  |  |  |  |  |  |  |  |  |  |  |  |  |  |  |  |  |  |  |  |  |  |  |
| Positive Regulation Of Animal Organ Morphogenesis | | 6 | | 5 | 8.18E-05 | 0.022 | VEGFA, WT1, BMP4, TGFB2, NOG, CSF1 | |  |  |  |  |  |  |  |  |  |  |  |  |  |  |  |  |  |  |  |  |  |  |  |
| Negative Regulation Of Epithelial Cell Proliferation | | 8 | | 3 | 8.43E-05 | 0.022 | MEF2C, BMP4, EFNB2, TGFB2, THBS1, TGFBR3, CASK, SNAI2 | |  |  |  |  |  |  |  |  |  |  |  |  |  |  |  |  |  |  |  |  |  |  |  |
| Cell-Cell Junction Organization | | 7 | | 4 | 8.69E-05 | 0.022 | PARD6B, CDH11, HEG1, DSP, TGFB2, F2RL1, SNAI2 | |  |  |  |  |  |  |  |  |  |  |  |  |  |  |  |  |  |  |  |  |  |  |  |
| Tissue Development | | 17 | | 2 | 8.80E-05 | 0.017 | GATA6, BMP4, MEF2C, HEG1, DSP, SNX10, NOG, LAMA3, CSF1, SNAI2, TUFT1, UGCG, VEGFA, WT1, TGFB2, THBS1, TIMP3 | |  |  |  |  |  |  |  |  |  |  |  |  |  |  |  |  |  |  |  |  |  |  |  |
| Regulation Of Plasma Membrane Bounded Cell Projection Organization | | 17 | | 2 | 8.80E-05 | 0.017 | PLK2, HLA-A, EVI5, PLXNA1, TENM3, BMP4, PTPRS, CASK, MEF2C, DPYSL2, SDC2, ASAP1, VEGFA, LPAR1, EFNB2, F2RL1, EFEMP1 | |  |  |  |  |  |  |  |  |  |  |  |  |  |  |  |  |  |  |  |  |  |  |  |
| Cellular Response To Platelet-Derived Growth Factor Stimulus | | 4 | | 11 | 9.01E-05 | 0.019 | HAS2, ITGB3, SNAI2, LTBP1 | |  |  |  |  |  |  |  |  |  |  |  |  |  |  |  |  |  |  |  |  |  |  |  |
| Regulation Of Transferase Activity | | 20 | | 1 | 9.05E-05 | 0.016 | ARRB1, ATP2B4, TRIB2, ITGB3, GPRC5A, CLU, BMP4, CEMIP, MAP3K1, CSF1, RAD50, AFAP1L2, VEGFA, LPAR1, TGFA, TGFB2, THBS1, TLR3, ACSL1, SERINC2 | |  |  |  |  |  |  |  |  |  |  |  |  |  |  |  |  |  |  |  |  |  |  |  |
| Eye Development | | 7 | | 4 | 9.06E-05 | 0.022 | MAP3K1, INHBB, VEGFA, WT1, BMP4, TGFB2, EFEMP1 | |  |  |  |  |  |  |  |  |  |  |  |  |  |  |  |  |  |  |  |  |  |  |  |
| Venous Blood Vessel Morphogenesis | | 3 | | 23 | 9.07E-05 | 0.016 | EFNB2, VEGFA, HEG1 | |  |  |  |  |  |  |  |  |  |  |  |  |  |  |  |  |  |  |  |  |  |  |  |
| Secretion By Cell | | 19 | | 1 | 9.07E-05 | 0.016 | ILDR2, ABCA1, ADGRE5, ITGB3, CLU, CPPED1, CASK, RAB27B, SLC44A2, CRISPLD2, DSP, CTSD, OPTN, VEGFA, TGFB2, THBS1, TIMP3, F2RL1, F13A1 | |  |  |  |  |  |  |  |  |  |  |  |  |  |  |  |  |  |  |  |  |  |  |  |
| Artery Morphogenesis | | 5 | | 7 | 9.18E-05 | 0.021 | ZMIZ1, VEGFA, BMP4, TGFB2, NOG | |  |  |  |  |  |  |  |  |  |  |  |  |  |  |  |  |  |  |  |  |  |  |  |
| Regulation Of Pathway-Restricted Smad Protein Phosphorylation | | 5 | | 7 | 9.88E-05 | 0.021 | INHBB, BMP4, TGFB2, NOG, PMEPA1 | |  |  |  |  |  |  |  |  |  |  |  |  |  |  |  |  |  |  |  |  |  |  |  |
| Positive Regulation Of Biological Process | | 74 | | 1 | 1.01E-04 | 0.010 | ARRB1, ATP2B4, ABCA1, TSPYL5, ADGRE5, FBXO32, CLU, BMP4, BMP1, C4BPB, DPYSL2, TRIM6, CPT1A, CSF1, LPAR1, EFNB2, EYA4, F2RL1, ACSL1, EFEMP1, HAS2, HLA-A, HLA-B, GATA6, PRICKLE1, IGFBP4, INHBB, ITGB3, HLA-DRA, MEF2C, MAP3K1, LCP1, LTBP1, PLPP4, ADGRF1, PLXNA1, SCN5A, SCP2, RAB27B, SOX4, SNAI2, VEGFA, WT1, TGFA, TGFB2, THBS1, TGFBR3, TGM2, TIMP3, TLR3, CASK, TNFSF10, PDLIM1, NOG, LIPG, RAD50, OPTN, PLK2, MIR181B1, ARL2BP, TIAM2, TNFRSF21, TRIB2, CDON, ASAP1, TENM3, BAIAP2L1, SLC44A2, ZMIZ1, CEMIP, HEG1, TNS3, COLGALT1, AFAP1L2 | |  |  |  |  |  |  |  |  |  |  |  |  |  |  |  |  |  |  |  |  |  |  |  |
| Response To Platelet-Derived Growth Factor | | 4 | | 11 | 1.01E-04 | 0.019 | HAS2, ITGB3, SNAI2, LTBP1 | |  |  |  |  |  |  |  |  |  |  |  |  |  |  |  |  |  |  |  |  |  |  |  |
| Negative Regulation Of Epithelial To Mesenchymal Transition | | 4 | | 11 | 1.01E-04 | 0.019 | VEGFA, TGFB2, TGFBR3, NOG | |  |  |  |  |  |  |  |  |  |  |  |  |  |  |  |  |  |  |  |  |  |  |  |
| Peptide Cross-Linking | | 6 | | 5 | 1.01E-04 | 0.022 | FLG, DSP, MAMDC2, THBS1, TGM2, F13A1 | |  |  |  |  |  |  |  |  |  |  |  |  |  |  |  |  |  |  |  |  |  |  |  |
| Regulation Of Cell Projection Organization | | 17 | | 1 | 1.02E-04 | 0.017 | PLK2, HLA-A, EVI5, PLXNA1, TENM3, BMP4, PTPRS, CASK, MEF2C, DPYSL2, SDC2, ASAP1, VEGFA, LPAR1, EFNB2, F2RL1, EFEMP1 | |  |  |  |  |  |  |  |  |  |  |  |  |  |  |  |  |  |  |  |  |  |  |  |
| Positive Regulation Of Cell Communication | | 31 | | 1 | 1.02E-04 | 0.013 | PLK2, ARRB1, MIR181B1, TSPYL5, CLU, BMP4, SOX4, SLC44A2, ZMIZ1, ARL2BP, TRIM6, CSF1, VEGFA, LPAR1, TGFA, TGFB2, THBS1, TGFBR3, TGM2, TIMP3, TLR3, F2RL1, GATA6, IGFBP4, INHBB, ITGB3, CASK, TNFSF10, CDON, MAP3K1, AFAP1L2 | |  |  |  |  |  |  |  |  |  |  |  |  |  |  |  |  |  |  |  |  |  |  |  |
| Regulation Of Cellular Process | | 124 | | 0 | 1.09E-04 | 0.009 | ARRB1, ATP2B4, ABCA1, CD22, ADGRE5, CLU, BMP4, BMP1, C4BPB, CYP24A1, DPYSL2, DSP, CPT1A, CSF1, CST4, LPAR1, EFNB2, EYA4, F2RL1, F13A1, ACSL1, EFEMP1, FBP1, GPR1, HAS2, HLA-A, HLA-B, GATA6, GBP1, GBP2, IGFBP4, INHBB, ITGB3, CD82, HLA-DRA, MEF2C, MEIS3P1, MAP3K1, LAMA3, LCP1, LTBP1, PLXNA1, RRBP1, SCN5A, SCP2, SCN9A, PTPRS, RAB27B, SOX4, SDC2, SNAI2, TUFT1, VEGFA, WT1, TGFA, TGFB2, THBS1, TGFBR3, TGM2, TIMP3, TLR3, ZNF43, EVI5, CLIC3, GPRC5A, SORBS2, CDC42BPA, CST7, CASK, TNFSF10, PTGES, FCGR2C, PDLIM1, NOG, LIPG, TSPAN2, RAD50, OPTN, MBNL2, CTDSPL, NUAK1, PLK2, PDE10A, MYL9, ARL2BP, TIAM2, TNFRSF21, TRIB2, CDON, KCNIP3, ASAP1, TENM3, BAIAP2L1, SLC44A2, ZMIZ1, CEMIP, MTUS1, ZNF608, SORCS2, PMEPA1, NXN, HHIP, SLAMF7, TNS3, PORCN, CDK15, CPEB4, FAT4, COLGALT1, ADAMTS12, PARD6B, AFAP1L2, TSPYL5, FBXO32, TRIM6, OR5P2, LYPD6B, PRICKLE1, SH3RF2, PLPP4, ADGRF1, ZNF391, SHISA2, MIR181B1 | |  |  |  |  |  |  |  |  |  |  |  |  |  |  |  |  |  |  |  |  |  |  |  |
| Regulation Of Transmembrane Receptor Protein Serine/Threonine Kinase Signaling Pathway | | 9 | | 3 | 1.10E-04 | 0.021 | GATA6, NOG, PMEPA1, LTBP1, INHBB, BMP4, TGFB2, THBS1, TGFBR3 | |  |  |  |  |  |  |  |  |  |  |  |  |  |  |  |  |  |  |  |  |  |  |  |
| Positive Regulation Of Striated Muscle Tissue Development | | 6 | | 5 | 1.12E-04 | 0.022 | CDON, MEF2C, BMP4, EFNB2, GATA6, TGFBR3 | |  |  |  |  |  |  |  |  |  |  |  |  |  |  |  |  |  |  |  |  |  |  |  |
| Positive Regulation Of Muscle Organ Development | | 6 | | 5 | 1.12E-04 | 0.022 | CDON, MEF2C, BMP4, EFNB2, GATA6, TGFBR3 | |  |  |  |  |  |  |  |  |  |  |  |  |  |  |  |  |  |  |  |  |  |  |  |
| Positive Regulation Of Catalytic Activity | | 25 | | 1 | 1.12E-04 | 0.014 | ARRB1, ATP2B4, CLU, BMP4, CEMIP, CSF1, TIAM2, VEGFA, LPAR1, TGFA, TGFB2, THBS1, TLR3, F2RL1, ACSL1, SERINC2, EVI5, TRIB2, ITGB3, TNFSF10, MEF2C, MAP3K1, ASAP1, RAD50, AFAP1L2 | |  |  |  |  |  |  |  |  |  |  |  |  |  |  |  |  |  |  |  |  |  |  |  |
| Regulation Of Keratinocyte Proliferation | | 4 | | 11 | 1.13E-04 | 0.019 | HAS2, EFNB2, CASK, SNAI2 | |  |  |  |  |  |  |  |  |  |  |  |  |  |  |  |  |  |  |  |  |  |  |  |
| Negative Regulation Of Intracellular Signal Transduction | | 14 | | 2 | 1.14E-04 | 0.018 | ARRB1, ATP2B4, PDE10A, GBP1, CLU, BMP4, SNAI2, OPTN, LPAR1, TGFB2, THBS1, TIMP3, F2RL1, FBP1 | |  |  |  |  |  |  |  |  |  |  |  |  |  |  |  |  |  |  |  |  |  |  |  |
| Regulation Of Nitric Oxide Mediated Signal Transduction | | 3 | | 21 | 1.15E-04 | 0.016 | THBS1, ATP2B4, VEGFA | |  |  |  |  |  |  |  |  |  |  |  |  |  |  |  |  |  |  |  |  |  |  |  |
| Positive Regulation Of Muscle Tissue Development | | 6 | | 5 | 1.18E-04 | 0.021 | CDON, MEF2C, BMP4, EFNB2, GATA6, TGFBR3 | |  |  |  |  |  |  |  |  |  |  |  |  |  |  |  |  |  |  |  |  |  |  |  |
| Positive Regulation Of Molecular Function | | 29 | | 1 | 1.20E-04 | 0.014 | PLK2, ARRB1, ATP2B4, ADGRF1, CLU, BMP4, CEMIP, TRIM6, CSF1, TIAM2, VEGFA, LPAR1, TGFA, TGFB2, THBS1, TGFBR3, TLR3, F2RL1, ACSL1, SERINC2, EVI5, TRIB2, ITGB3, TNFSF10, MEF2C, MAP3K1, ASAP1, RAD50, AFAP1L2 | |  |  |  |  |  |  |  |  |  |  |  |  |  |  |  |  |  |  |  |  |  |  |  |
| Negative Regulation Of Cellular Protein Metabolic Process | | 22 | | 1 | 1.20E-04 | 0.015 | ARRB1, ATP2B4, MIR181B1, GBP1, TRIB2, CPEB4, GPRC5A, BMP4, CST7, C4BPB, SH3RF2, SOX4, NOG, PMEPA1, CST4, CTDSPL, VEGFA, WT1, NXN, THBS1, TIMP3, F2RL1 | |  |  |  |  |  |  |  |  |  |  |  |  |  |  |  |  |  |  |  |  |  |  |  |
| Positive Regulation Of Small Gtpase Mediated Signal Transduction | | 5 | | 7 | 1.31E-04 | 0.021 | CDON, ARRB1, LPAR1, CSF1, F2RL1 | |  |  |  |  |  |  |  |  |  |  |  |  |  |  |  |  |  |  |  |  |  |  |  |
| Phosphate-Containing Compound Metabolic Process | | 32 | | 1 | 1.31E-04 | 0.013 | LPCAT2, PLK2, PDE10A, AK4, SCP2, BMP4, PTPRS, CPPED1, SLC44A2, EFNB2, TGFB2, TGFBR3, EYA4, TLR3, ACSL1, SERINC2, EFEMP1, FBP1, GATA6, CDK15, ELOVL7, TRIB2, CDC42BPA, CASK, MEF2C, MAP3K1, LIPG, PLPP4, RAD50, CTDSPL, SLC37A2, NUAK1 | |  |  |  |  |  |  |  |  |  |  |  |  |  |  |  |  |  |  |  |  |  |  |  |
| Tube Formation | | 7 | | 4 | 1.35E-04 | 0.021 | SOX4, VEGFA, BMP4, TGFB2, NOG, TGM2, PRICKLE1 | |  |  |  |  |  |  |  |  |  |  |  |  |  |  |  |  |  |  |  |  |  |  |  |
| Cardiac Right Ventricle Morphogenesis | | 3 | | 20 | 1.43E-04 | 0.016 | SOX4, BMP4, TGFB2 | |  |  |  |  |  |  |  |  |  |  |  |  |  |  |  |  |  |  |  |  |  |  |  |
| Negative Regulation Of Keratinocyte Proliferation | | 3 | | 20 | 1.43E-04 | 0.016 | EFNB2, CASK, SNAI2 | |  |  |  |  |  |  |  |  |  |  |  |  |  |  |  |  |  |  |  |  |  |  |  |
| Regulation Of Biological Process | | 131 | | 0 | 1.44E-04 | 0.008 | ARRB1, ATP2B4, ABCA1, CD22, ADGRE5, CLU, BMP4, BMP1, C4BPB, CYP24A1, DPYSL2, DSP, CPT1A, CSF1, CST4, LPAR1, EFNB2, EYA4, F2RL1, F13A1, ACSL1, EFEMP1, FBP1, GPR1, HAS2, HLA-A, HLA-B, GATA6, GBP1, GBP2, IGFBP4, INHBB, ITGB3, CD82, HLA-DRA, MEF2C, MEIS3P1, MAP3K1, LAMA3, LCP1, LTBP1, PLXNA1, RRBP1, SCN5A, SCP2, SCN9A, PTPRS, RAB27B, SOX4, SDC2, SNAI2, TUFT1, VEGFA, WT1, TGFA, TGFB2, THBS1, TGFBR3, TGM2, TIMP3, TLR3, ZNF43, EVI5, CLIC3, GPRC5A, SORBS2, CDC42BPA, CST7, CASK, TNFSF10, PTGES, CYTIP, FCGR2C, PDLIM1, NOG, LIPG, TSPAN2, RAD50, OPTN, MBNL2, CTDSPL, NUAK1, PLK2, PDE10A, MYL9, TDRD7, ARL2BP, TIAM2, TNFRSF21, TRIB2, CDON, KCNIP3, ASAP1, CLIC6, TENM3, BAIAP2L1, SLC44A2, ZMIZ1, CEMIP, HEG1, MTUS1, ZNF608, SORCS2, PMEPA1, NXN, HHIP, SLAMF7, TNS3, PORCN, CDK15, CPEB4, FAT4, COLGALT1, ADAMTS12, PARD6B, AFAP1L2, TSPYL5, FBXO32, TRIM6, OR5P2, LYPD6B, PRICKLE1, SH3RF2, SAMD9L, PLPP4, ADGRF1, ZNF391, SHISA2, MIR181B1, UCA1, MIR604 | |  |  |  |  |  |  |  |  |  |  |  |  |  |  |  |  |  |  |  |  |  |  |  |
| Interferon-Gamma-Mediated Signaling Pathway | | 5 | | 6 | 1.50E-04 | 0.020 | HLA-A, HLA-B, HLA-DRA, GBP1, GBP2 | |  |  |  |  |  |  |  |  |  |  |  |  |  |  |  |  |  |  |  |  |  |  |  |
| Regulation Of Small Gtpase Mediated Signal Transduction | | 10 | | 2 | 1.52E-04 | 0.020 | CDON, ARRB1, ABCA1, CSF1, TIAM2, LPAR1, CDC42BPA, TGFB2, F2RL1, FBP1 | |  |  |  |  |  |  |  |  |  |  |  |  |  |  |  |  |  |  |  |  |  |  |  |
| Branching Morphogenesis Of An Epithelial Tube | | 7 | | 4 | 1.52E-04 | 0.021 | VEGFA, WT1, HHIP, FAT4, BMP4, CSF1, CTSD | |  |  |  |  |  |  |  |  |  |  |  |  |  |  |  |  |  |  |  |  |  |  |  |
| Cardiac Chamber Morphogenesis | | 4 | | 10 | 1.56E-04 | 0.019 | SOX4, HEG1, BMP4, TGFB2 | |  |  |  |  |  |  |  |  |  |  |  |  |  |  |  |  |  |  |  |  |  |  |  |
| Heart Morphogenesis | | 5 | | 6 | 1.60E-04 | 0.020 | ZMIZ1, VEGFA, FAT4, TGFB2, TGFBR3 | |  |  |  |  |  |  |  |  |  |  |  |  |  |  |  |  |  |  |  |  |  |  |  |
| Phosphorus Metabolic Process | | 32 | | 1 | 1.65E-04 | 0.013 | LPCAT2, PLK2, PDE10A, AK4, SCP2, BMP4, PTPRS, CPPED1, SLC44A2, EFNB2, TGFB2, TGFBR3, EYA4, TLR3, ACSL1, SERINC2, EFEMP1, FBP1, GATA6, CDK15, ELOVL7, TRIB2, CDC42BPA, CASK, MEF2C, MAP3K1, LIPG, PLPP4, RAD50, CTDSPL, SLC37A2, NUAK1 | |  |  |  |  |  |  |  |  |  |  |  |  |  |  |  |  |  |  |  |  |  |  |  |
| Positive Regulation Of Transmembrane Receptor Protein Serine/Threonine Kinase Signaling Pathway | | 6 | | 5 | 1.66E-04 | 0.021 | INHBB, BMP4, TGFB2, GATA6, THBS1, TGFBR3 | |  |  |  |  |  |  |  |  |  |  |  |  |  |  |  |  |  |  |  |  |  |  |  |
| Negative Regulation Of Cellular Process | | 61 | | 1 | 1.74E-04 | 0.010 | PLK2, ARRB1, ATP2B4, PDE10A, MIR181B1, ABCA1, CLU, BMP4, C4BPB, TRIM6, CST4, LPAR1, EFNB2, EYA4, F2RL1, EFEMP1, FBP1, TNFRSF21, HLA-A, GATA6, GBP1, PRICKLE1, IGFBP4, INHBB, TRIB2, ITGB3, SH3RF2, MEF2C, MAP3K1, KCNIP3, LTBP1, ASAP1, PLXNA1, SCP2, PTPRS, SOX4, ZNF608, PMEPA1, SNAI2, VEGFA, WT1, NXN, HHIP, TGFA, TGFB2, THBS1, TGFBR3, TIMP3, TLR3, SHISA2, CPEB4, GPRC5A, CST7, CASK, TNFSF10, ADAMTS12, PTGES, NOG, RAD50, OPTN, CTDSPL | |  |  |  |  |  |  |  |  |  |  |  |  |  |  |  |  |  |  |  |  |  |  |  |
| Regulation Of Cell Proliferation Involved In Kidney Development | | 3 | | 18 | 1.75E-04 | 0.016 | BMP4, ITGB3, WT1 | |  |  |  |  |  |  |  |  |  |  |  |  |  |  |  |  |  |  |  |  |  |  |  |
| Negative Regulation Of Dendritic Spine Development | | 3 | | 18 | 1.75E-04 | 0.016 | PTPRS, PLK2, ASAP1 | |  |  |  |  |  |  |  |  |  |  |  |  |  |  |  |  |  |  |  |  |  |  |  |
| Ossification | | 7 | | 4 | 1.77E-04 | 0.021 | CDH11, MEF2C, FAT4, BMP4, BMP1, NOG, CSF1 | |  |  |  |  |  |  |  |  |  |  |  |  |  |  |  |  |  |  |  |  |  |  |  |
| Response To Endogenous Stimulus | | 25 | | 1 | 1.77E-04 | 0.014 | ATP2B4, ABCA1, FBXO32, SCP2, BMP4, SNAI2, VEGFA, WT1, TGFB2, THBS1, TGFBR3, TIMP3, FBP1, HAS2, GATA6, INHBB, ITGB3, CPEB4, TNFSF10, ADAMTS12, MEF2C, NOG, LTBP1, RAD50, OPTN | |  |  |  |  |  |  |  |  |  |  |  |  |  |  |  |  |  |  |  |  |  |  |  |
| Regulation Of Vasculature Development | | 12 | | 2 | 1.81E-04 | 0.018 | PLK2, MIR181B1, GATA6, VEGFA, ITGB3, WT1, HHIP, BMP4, EFNB2, TGFB2, THBS1, TLR3 | |  |  |  |  |  |  |  |  |  |  |  |  |  |  |  |  |  |  |  |  |  |  |  |
| Positive Regulation Of Cardiac Muscle Tissue Development | | 5 | | 6 | 1.81E-04 | 0.020 | MEF2C, BMP4, EFNB2, GATA6, TGFBR3 | |  |  |  |  |  |  |  |  |  |  |  |  |  |  |  |  |  |  |  |  |  |  |  |
| Positive Regulation Of Mapk Cascade | | 14 | | 2 | 1.84E-04 | 0.017 | ARRB1, MIR181B1, IGFBP4, ITGB3, BMP4, CDON, MAP3K1, VEGFA, LPAR1, TGFA, TGFB2, THBS1, TLR3, F2RL1 | |  |  |  |  |  |  |  |  |  |  |  |  |  |  |  |  |  |  |  |  |  |  |  |
| Skeletal System Development | | 8 | | 3 | 1.90E-04 | 0.020 | SOX4, IGFBP4, CDH11, BMP4, BMP1, TGFB2, NOG, TLL1 | |  |  |  |  |  |  |  |  |  |  |  |  |  |  |  |  |  |  |  |  |  |  |  |
| Odontogenesis | | 6 | | 5 | 1.91E-04 | 0.021 | TUFT1, SCN5A, BMP4, TGFB2, GATA6, CSF1 | |  |  |  |  |  |  |  |  |  |  |  |  |  |  |  |  |  |  |  |  |  |  |  |
| Secretion | | 20 | | 1 | 1.92E-04 | 0.015 | ILDR2, ABCA1, ADGRE5, ITGB3, CLU, CPPED1, CASK, RAB27B, SLC44A2, CRISPLD2, DSP, SNX10, CTSD, OPTN, VEGFA, TGFB2, THBS1, TIMP3, F2RL1, F13A1 | |  |  |  |  |  |  |  |  |  |  |  |  |  |  |  |  |  |  |  |  |  |  |  |
| Regulation Of Neuron Differentiation | | 16 | | 1 | 2.02E-04 | 0.016 | PLK2, HLA-A, PLXNA1, TENM3, BMP4, PTPRS, CASK, CDON, MEF2C, DPYSL2, SDC2, ASAP1, VEGFA, LPAR1, EFNB2, EFEMP1 | |  |  |  |  |  |  |  |  |  |  |  |  |  |  |  |  |  |  |  |  |  |  |  |
| Response To Organic Cyclic Compound | | 22 | | 1 | 2.05E-04 | 0.015 | ATP2B4, ABCA1, GATA6, FBXO32, INHBB, SCN5A, SCP2, BMP4, CYP24A1, PTGES, MEF2C, DPYSL2, SDC2, CPT1A, LTBP1, VEGFA, WT1, TGFB2, THBS1, TIMP3, TLR3, ACSL1 | |  |  |  |  |  |  |  |  |  |  |  |  |  |  |  |  |  |  |  |  |  |  |  |
| Activation Of Protein Kinase Activity | | 10 | | 2 | 2.05E-04 | 0.019 | ARRB1, MAP3K1, VEGFA, ITGB3, LPAR1, BMP4, TGFA, TGFB2, THBS1, TLR3 | |  |  |  |  |  |  |  |  |  |  |  |  |  |  |  |  |  |  |  |  |  |  |  |
| Response To Tumor Necrosis Factor | | 8 | | 3 | 2.08E-04 | 0.020 | TNFRSF21, ADAMTS12, HAS2, GBP1, GBP2, GBP3, OPTN, THBS1 | |  |  |  |  |  |  |  |  |  |  |  |  |  |  |  |  |  |  |  |  |  |  |  |
| Regulation Of Extracellular Matrix Organization | | 4 | | 9 | 2.08E-04 | 0.019 | HAS2, ITGB3, TGFB2, COLGALT1 | |  |  |  |  |  |  |  |  |  |  |  |  |  |  |  |  |  |  |  |  |  |  |  |
| Myeloid Leukocyte Differentiation | | 6 | | 4 | 2.09E-04 | 0.021 | MEF2C, VEGFA, BMP4, SNX10, CSF1, F2RL1 | |  |  |  |  |  |  |  |  |  |  |  |  |  |  |  |  |  |  |  |  |  |  |  |
| Cellular Response To Cholesterol | | 3 | | 17 | 2.11E-04 | 0.016 | ABCA1, INHBB, SCP2 | |  |  |  |  |  |  |  |  |  |  |  |  |  |  |  |  |  |  |  |  |  |  |  |
| Cardiac Muscle Tissue Morphogenesis | | 5 | | 6 | 2.18E-04 | 0.020 | HEG1, DSP, TGFB2, TGFBR3, NOG | |  |  |  |  |  |  |  |  |  |  |  |  |  |  |  |  |  |  |  |  |  |  |  |
| Regulation Of I-Kappab Kinase/Nf-Kappab Signaling | | 8 | | 3 | 2.20E-04 | 0.020 | SLC44A2, PLK2, OPTN, LPAR1, TGM2, TLR3, F2RL1, TNFSF10 | |  |  |  |  |  |  |  |  |  |  |  |  |  |  |  |  |  |  |  |  |  |  |  |
| Cell Proliferation | | 16 | | 1 | 2.32E-04 | 0.016 | EVI5, IGFBP4, CLU, FAT4, BMP4, SOX4, MEF2C, SAMD9L, CSF1, LIPG, VEGFA, HHIP, TGFA, TGFB2, TGFBR3, F2RL1 | |  |  |  |  |  |  |  |  |  |  |  |  |  |  |  |  |  |  |  |  |  |  |  |
| Negative Regulation Of Extrinsic Apoptotic Signaling Pathway | | 6 | | 4 | 2.50E-04 | 0.020 | VEGFA, BMP4, THBS1, EYA4, SNAI2, TNFSF10 | |  |  |  |  |  |  |  |  |  |  |  |  |  |  |  |  |  |  |  |  |  |  |  |
| Cellular Response To Bmp Stimulus | | 4 | | 9 | 2.50E-04 | 0.018 | ADAMTS12, BMP4, GATA6, NOG | |  |  |  |  |  |  |  |  |  |  |  |  |  |  |  |  |  |  |  |  |  |  |  |
| Response To Bmp | | 4 | | 9 | 2.50E-04 | 0.018 | ADAMTS12, BMP4, GATA6, NOG | |  |  |  |  |  |  |  |  |  |  |  |  |  |  |  |  |  |  |  |  |  |  |  |
| Regulation Of Fibroblast Growth Factor Receptor Signaling Pathway | | 4 | | 9 | 2.50E-04 | 0.018 | SHISA2, HHIP, THBS1, NOG | |  |  |  |  |  |  |  |  |  |  |  |  |  |  |  |  |  |  |  |  |  |  |  |
| Reverse Cholesterol Transport | | 3 | | 16 | 2.52E-04 | 0.016 | ABCA1, CLU, LIPG | |  |  |  |  |  |  |  |  |  |  |  |  |  |  |  |  |  |  |  |  |  |  |  |
| Response To Drug | | 21 | | 1 | 2.54E-04 | 0.015 | ARRB1, ABCA1, GATA6, AK4, FBXO32, PTGR1, ITGB3, BMP4, MEF2C, DPYSL2, SDC2, CPT1A, OPTN, VEGFA, TGFA, TGFB2, THBS1, TIMP3, TLR3, ACSL1, FBP1 | |  |  |  |  |  |  |  |  |  |  |  |  |  |  |  |  |  |  |  |  |  |  |  |
| Regulation Of Cardiac Muscle Tissue Development | | 6 | | 4 | 2.72E-04 | 0.020 | MEF2C, BMP4, EFNB2, GATA6, TGFBR3, NOG | |  |  |  |  |  |  |  |  |  |  |  |  |  |  |  |  |  |  |  |  |  |  |  |
| Positive Regulation Of I-Kappab Kinase/Nf-Kappab Signaling | | 7 | | 3 | 2.79E-04 | 0.020 | SLC44A2, PLK2, LPAR1, TGM2, TLR3, F2RL1, TNFSF10 | |  |  |  |  |  |  |  |  |  |  |  |  |  |  |  |  |  |  |  |  |  |  |  |
| Enzyme Linked Receptor Protein Signaling Pathway | | 15 | | 1 | 2.80E-04 | 0.016 | INHBB, ITGB3, FAT4, BMP4, MAP3K1, SDC2, NOG, CSF1, LTBP1, VEGFA, EFNB2, TGFA, TGFB2, TGFBR3, EFEMP1 | |  |  |  |  |  |  |  |  |  |  |  |  |  |  |  |  |  |  |  |  |  |  |  |
| Response To Nutrient | | 10 | | 2 | 2.85E-04 | 0.019 | CYP24A1, MEF2C, ABCA1, LIPG, LTBP1, OPTN, VEGFA, TGFB2, TIMP3, ACSL1 | |  |  |  |  |  |  |  |  |  |  |  |  |  |  |  |  |  |  |  |  |  |  |  |
| Negative Regulation Of Glomerular Mesangial Cell Proliferation | | 2 | | 50 | 2.88E-04 | 0.011 | BMP4, WT1 | |  |  |  |  |  |  |  |  |  |  |  |  |  |  |  |  |  |  |  |  |  |  |  |
| Ascending Aorta Morphogenesis | | 2 | | 50 | 2.88E-04 | 0.011 | SOX4, TGFB2 | |  |  |  |  |  |  |  |  |  |  |  |  |  |  |  |  |  |  |  |  |  |  |  |
| Negative Regulation Of Cgmp-Mediated Signaling | | 2 | | 50 | 2.88E-04 | 0.011 | THBS1, PDE10A | |  |  |  |  |  |  |  |  |  |  |  |  |  |  |  |  |  |  |  |  |  |  |  |
| Negative Regulation Of Nitric Oxide Mediated Signal Transduction | | 2 | | 50 | 2.88E-04 | 0.011 | THBS1, ATP2B4 | |  |  |  |  |  |  |  |  |  |  |  |  |  |  |  |  |  |  |  |  |  |  |  |
| Negative Regulation Of Glomerulus Development | | 2 | | 50 | 2.88E-04 | 0.011 | BMP4, WT1 | |  |  |  |  |  |  |  |  |  |  |  |  |  |  |  |  |  |  |  |  |  |  |  |
| Regulation Of Cytokine Production Involved In Immune Response | | 5 | | 6 | 2.92E-04 | 0.020 | HLA-A, TRIM6, TGFB2, TLR3, F2RL1 | |  |  |  |  |  |  |  |  |  |  |  |  |  |  |  |  |  |  |  |  |  |  |  |
| Regulation Of Stem Cell Differentiation | | 5 | | 6 | 2.92E-04 | 0.020 | BMP4, TRIM6, TGFB2, GATA6, PRICKLE1 | |  |  |  |  |  |  |  |  |  |  |  |  |  |  |  |  |  |  |  |  |  |  |  |
| Carbohydrate Derivative Metabolic Process | | 18 | | 1 | 2.95E-04 | 0.015 | PDE10A, HAS2, MAMDC2, PORCN, AK4, MGAT4A, ELOVL7, SCP2, CASK, ADAMTS12, CEMIP, SDC2, UGCG, VEGFA, SLC37A2, CHST15, ACSL1, FBP1 | |  |  |  |  |  |  |  |  |  |  |  |  |  |  |  |  |  |  |  |  |  |  |  |
| Cellular Response To Interleukin-1 | | 6 | | 4 | 2.96E-04 | 0.020 | ADAMTS12, INHBB, HAS2, GBP1, GBP2, GBP3 | |  |  |  |  |  |  |  |  |  |  |  |  |  |  |  |  |  |  |  |  |  |  |  |
| Negative Regulation Of Cell Death | | 21 | | 1 | 2.97E-04 | 0.014 | PLK2, ARRB1, GATA6, ITGB3, CPEB4, CLU, BMP4, TNFSF10, SOX4, MEF2C, NOG, SNAI2, OPTN, VEGFA, WT1, HHIP, TGFA, TGFB2, THBS1, TGFBR3, EYA4 | |  |  |  |  |  |  |  |  |  |  |  |  |  |  |  |  |  |  |  |  |  |  |  |
| Heart Valve Morphogenesis | | 4 | | 8 | 2.97E-04 | 0.018 | SOX4, MEF2C, BMP4, TGFB2 | |  |  |  |  |  |  |  |  |  |  |  |  |  |  |  |  |  |  |  |  |  |  |  |
| Response To Oxygen-Containing Compound | | 30 | | 1 | 2.97E-04 | 0.013 | ATP2B4, ABCA1, FBXO32, SCP2, BMP4, SOX4, CYP24A1, DPYSL2, TRIM6, CPT1A, VEGFA, WT1, LPAR1, TGFB2, THBS1, TGFBR3, TIMP3, ACSL1, FBP1, ILDR2, GATA6, GBP2, INHBB, ITGB3, CPEB4, TNFSF10, PTGES, MEF2C, LTBP1, OPTN | |  |  |  |  |  |  |  |  |  |  |  |  |  |  |  |  |  |  |  |  |  |  |  |
| Macrophage Differentiation | | 3 | | 15 | 2.98E-04 | 0.016 | BMP4, CSF1, VEGFA | |  |  |  |  |  |  |  |  |  |  |  |  |  |  |  |  |  |  |  |  |  |  |  |
| Neural Retina Development | | 3 | | 15 | 2.98E-04 | 0.016 | OPTN, TGFB2, ATP2B4 | |  |  |  |  |  |  |  |  |  |  |  |  |  |  |  |  |  |  |  |  |  |  |  |
| Regulation Of Apoptotic Process Involved In Development | | 3 | | 15 | 2.98E-04 | 0.016 | TGFB2, TGFBR3, WT1 | |  |  |  |  |  |  |  |  |  |  |  |  |  |  |  |  |  |  |  |  |  |  |  |
| Regulation Of Chemotaxis | | 8 | | 3 | 3.10E-04 | 0.019 | MTUS1, CSF1, SNAI2, VEGFA, LPAR1, EFNB2, THBS1, F2RL1 | |  |  |  |  |  |  |  |  |  |  |  |  |  |  |  |  |  |  |  |  |  |  |  |
| Regulation Of Ras Protein Signal Transduction | | 8 | | 3 | 3.19E-04 | 0.019 | ARRB1, ABCA1, CSF1, TIAM2, LPAR1, TGFB2, F2RL1, FBP1 | |  |  |  |  |  |  |  |  |  |  |  |  |  |  |  |  |  |  |  |  |  |  |  |
| Response To Stress | | 46 | | 1 | 3.22E-04 | 0.011 | PLK2, ATP2B4, ABCA1, ADGRE5, CLU, BMP4, C4BPB, DSP, CSF1, EYA4, F2RL1, F13A1, HAS2, HLA-A, GATA6, GBP1, GBP2, GBP3, IGFBP4, INHBB, ITGB3, MEF2C, MAP3K1, LCP1, KCNIP3, LTBP1, PON2, SCN9A, SOX4, SDC2, VEGFA, TGFA, TGFB2, THBS1, TGFBR3, SLAMF7, TLR3, CPEB4, PTGES, PDLIM1, NOG, TSPAN2, RAD50, AFAP1L2, OPTN, NUAK1 | |  |  |  |  |  |  |  |  |  |  |  |  |  |  |  |  |  |  |  |  |  |  |  |
| Ventricular Septum Morphogenesis | | 4 | | 8 | 3.23E-04 | 0.018 | SOX4, TGFB2, TGFBR3, NOG | |  |  |  |  |  |  |  |  |  |  |  |  |  |  |  |  |  |  |  |  |  |  |  |
| Multicellular Organism Development | | 24 | | 1 | 3.26E-04 | 0.014 | PLXNA1, CDH11, BMP4, BMP1, TDRD7, ZMIZ1, HEG1, DPYSL2, SNAI2, FLG, VEGFA, NXN, EFNB2, TGFB2, TGFBR3, TLL1, EYA4, SLC7A5, SHISA2, GATA6, EVI5, INHBB, MEF2C, NOG | |  |  |  |  |  |  |  |  |  |  |  |  |  |  |  |  |  |  |  |  |  |  |  |
| Positive Regulation Of Cell Migration | | 13 | | 2 | 3.29E-04 | 0.017 | PLK2, CEMIP, HAS2, MIR181B1, CSF1, SNAI2, VEGFA, ITGB3, LPAR1, BMP4, TGFB2, THBS1, F2RL1 | |  |  |  |  |  |  |  |  |  |  |  |  |  |  |  |  |  |  |  |  |  |  |  |
| Positive Regulation Of Endothelial Cell Migration | | 6 | | 4 | 3.35E-04 | 0.020 | PLK2, VEGFA, ITGB3, BMP4, THBS1, SNAI2 | |  |  |  |  |  |  |  |  |  |  |  |  |  |  |  |  |  |  |  |  |  |  |  |
| Negative Regulation Of Nervous System Development | | 10 | | 2 | 3.45E-04 | 0.018 | TNFRSF21, PLK2, HLA-A, NOG, ASAP1, LPAR1, BMP4, EFNB2, PTPRS, EFEMP1 | |  |  |  |  |  |  |  |  |  |  |  |  |  |  |  |  |  |  |  |  |  |  |  |
| Anatomical Structure Formation Involved In Morphogenesis | | 16 | | 1 | 3.48E-04 | 0.016 | GATA6, PRICKLE1, ITGB3, BMP4, SOX4, CDON, MEF2C, NOG, VEGFA, WT1, EFNB2, TGFA, TGFB2, THBS1, TGFBR3, TGM2 | |  |  |  |  |  |  |  |  |  |  |  |  |  |  |  |  |  |  |  |  |  |  |  |
| Epithelial Cell Differentiation Involved In Kidney Development | | 3 | | 15 | 3.49E-04 | 0.015 | BMP4, MEF2C, WT1 | |  |  |  |  |  |  |  |  |  |  |  |  |  |  |  |  |  |  |  |  |  |  |  |
| Regulated Exocytosis | | 14 | | 2 | 3.58E-04 | 0.016 | ADGRE5, ITGB3, CLU, CPPED1, RAB27B, SLC44A2, CRISPLD2, DSP, CTSD, VEGFA, TGFB2, THBS1, TIMP3, F13A1 | |  |  |  |  |  |  |  |  |  |  |  |  |  |  |  |  |  |  |  |  |  |  |  |
| Negative Regulation Of Neuron Projection Development | | 7 | | 3 | 3.63E-04 | 0.020 | PLK2, HLA-A, LPAR1, EFNB2, PTPRS, ASAP1, EFEMP1 | |  |  |  |  |  |  |  |  |  |  |  |  |  |  |  |  |  |  |  |  |  |  |  |
| Negative Regulation Of Protein Modification Process | | 14 | | 2 | 3.85E-04 | 0.016 | ARRB1, ATP2B4, GBP1, TRIB2, GPRC5A, BMP4, SH3RF2, SOX4, NOG, PMEPA1, CTDSPL, NXN, TIMP3, F2RL1 | |  |  |  |  |  |  |  |  |  |  |  |  |  |  |  |  |  |  |  |  |  |  |  |
| Regulation Of Animal Organ Morphogenesis | | 8 | | 3 | 3.96E-04 | 0.019 | NOG, PRICKLE1, CSF1, SNAI2, VEGFA, WT1, BMP4, TGFB2 | |  |  |  |  |  |  |  |  |  |  |  |  |  |  |  |  |  |  |  |  |  |  |  |
| Sensory Organ Development | | 7 | | 3 | 4.25E-04 | 0.019 | MAP3K1, INHBB, VEGFA, WT1, BMP4, TGFB2, EFEMP1 | |  |  |  |  |  |  |  |  |  |  |  |  |  |  |  |  |  |  |  |  |  |  |  |
| Response To Fluid Shear Stress | | 4 | | 7 | 4.43E-04 | 0.018 | MEF2C, HAS2, ABCA1, TGFB2 | |  |  |  |  |  |  |  |  |  |  |  |  |  |  |  |  |  |  |  |  |  |  |  |
| Regulation Of Ion Transmembrane Transport | | 12 | | 2 | 4.49E-04 | 0.017 | MEF2C, CEMIP, ATP2B4, KCNIP3, CLIC6, CLIC3, ITGB3, SCN5A, SCN9A, BMP4, TGFB2, THBS1 | |  |  |  |  |  |  |  |  |  |  |  |  |  |  |  |  |  |  |  |  |  |  |  |
| Cell Development | | 17 | | 1 | 4.62E-04 | 0.015 | GATA6, TENM3, INHBB, BMP4, PTPRS, SOX4, TDRD7, MEF2C, HEG1, SNAI2, TSPAN2, VEGFA, WT1, LPAR1, TGFB2, TGFBR3, F2RL1 | |  |  |  |  |  |  |  |  |  |  |  |  |  |  |  |  |  |  |  |  |  |  |  |
| Mononuclear Cell Differentiation | | 3 | | 13 | 4.66E-04 | 0.015 | BMP4, MEF2C, VEGFA | |  |  |  |  |  |  |  |  |  |  |  |  |  |  |  |  |  |  |  |  |  |  |  |
| Monocyte Differentiation | | 3 | | 13 | 4.66E-04 | 0.015 | BMP4, MEF2C, VEGFA | |  |  |  |  |  |  |  |  |  |  |  |  |  |  |  |  |  |  |  |  |  |  |  |
| Regulation Of Signaling Receptor Activity | | 13 | | 2 | 4.76E-04 | 0.016 | INHBB, GPRC5A, BMP4, BMP1, TNFSF10, MEF2C, NOG, CSF1, VEGFA, TGFA, TGFB2, LYPD6B, EFEMP1 | |  |  |  |  |  |  |  |  |  |  |  |  |  |  |  |  |  |  |  |  |  |  |  |
| Atrial Septum Primum Morphogenesis | | 2 | | 40 | 4.78E-04 | 0.011 | SOX4, TGFB2 | |  |  |  |  |  |  |  |  |  |  |  |  |  |  |  |  |  |  |  |  |  |  |  |
| Apolipoprotein A-I-Mediated Signaling Pathway | | 2 | | 40 | 4.78E-04 | 0.011 | ABCA1, ITGB3 | |  |  |  |  |  |  |  |  |  |  |  |  |  |  |  |  |  |  |  |  |  |  |  |
| Positive Regulation Of Cardioblast Differentiation | | 2 | | 40 | 4.78E-04 | 0.011 | TGFB2, GATA6 | |  |  |  |  |  |  |  |  |  |  |  |  |  |  |  |  |  |  |  |  |  |  |  |
| Cellular Protein Modification Process | | 40 | | 1 | 4.81E-04 | 0.011 | PLK2, ARRB1, ABCA1, MGAT4A, FBXO32, MARCH3, BMP4, PRSS23, DSP, TRIM6, CSF1, FLG, EFNB2, EYA4, F13A1, EFEMP1, IGFBP4, TRIB2, SH3RF2, MEF2C, MAP3K1, CPA4, LTBP1, HECTD4, MAMDC2, KLHL4, PTPRS, CPPED1, SDC2, TGFB2, THBS1, TGFBR3, TGM2, TLR3, PORCN, CDK15, CDC42BPA, CASK, CTDSPL, NUAK1 | |  |  |  |  |  |  |  |  |  |  |  |  |  |  |  |  |  |  |  |  |  |  |  |
| Protein Modification Process | | 40 | | 1 | 4.81E-04 | 0.011 | PLK2, ARRB1, ABCA1, MGAT4A, FBXO32, MARCH3, BMP4, PRSS23, DSP, TRIM6, CSF1, FLG, EFNB2, EYA4, F13A1, EFEMP1, IGFBP4, TRIB2, SH3RF2, MEF2C, MAP3K1, CPA4, LTBP1, HECTD4, MAMDC2, KLHL4, PTPRS, CPPED1, SDC2, TGFB2, THBS1, TGFBR3, TGM2, TLR3, PORCN, CDK15, CDC42BPA, CASK, CTDSPL, NUAK1 | |  |  |  |  |  |  |  |  |  |  |  |  |  |  |  |  |  |  |  |  |  |  |  |
| Positive Regulation Of Protein Serine/Threonine Kinase Activity | | 10 | | 2 | 4.96E-04 | 0.018 | ARRB1, CEMIP, MAP3K1, ATP2B4, VEGFA, LPAR1, BMP4, TGFA, THBS1, ACSL1 | |  |  |  |  |  |  |  |  |  |  |  |  |  |  |  |  |  |  |  |  |  |  |  |
| Positive Regulation Of Cell Motility | | 13 | | 2 | 4.98E-04 | 0.016 | PLK2, HAS2, MIR181B1, ITGB3, BMP4, CEMIP, CSF1, SNAI2, VEGFA, LPAR1, TGFB2, THBS1, F2RL1 | |  |  |  |  |  |  |  |  |  |  |  |  |  |  |  |  |  |  |  |  |  |  |  |
| Regulation Of Response To External Stimulus | | 17 | | 1 | 5.01E-04 | 0.015 | CLU, PTPRS, C4BPB, CASK, ADAMTS12, MTUS1, TRIM6, CSF1, SNAI2, OPTN, VEGFA, LPAR1, EFNB2, THBS1, TGM2, TLR3, F2RL1 | |  |  |  |  |  |  |  |  |  |  |  |  |  |  |  |  |  |  |  |  |  |  |  |
| Negative Regulation Of Apoptotic Process | | 19 | | 1 | 5.06E-04 | 0.014 | PLK2, ARRB1, GATA6, CPEB4, CLU, BMP4, TNFSF10, MEF2C, NOG, SNAI2, OPTN, VEGFA, WT1, HHIP, TGFA, TGFB2, THBS1, TGFBR3, EYA4 | |  |  |  |  |  |  |  |  |  |  |  |  |  |  |  |  |  |  |  |  |  |  |  |
| Regulation Of Chondrocyte Differentiation | | 4 | | 7 | 5.13E-04 | 0.018 | ADAMTS12, BMP4, SNAI2, EFEMP1 | |  |  |  |  |  |  |  |  |  |  |  |  |  |  |  |  |  |  |  |  |  |  |  |
| Negative Regulation Of Cell Adhesion | | 9 | | 2 | 5.15E-04 | 0.018 | TNFRSF21, ZNF608, GBP1, SNAI2, PLXNA1, VEGFA, BMP4, THBS1, CASK | |  |  |  |  |  |  |  |  |  |  |  |  |  |  |  |  |  |  |  |  |  |  |  |
| Response To Cytokine | | 17 | | 1 | 5.19E-04 | 0.015 | TNFRSF21, HAS2, HLA-A, GBP1, GBP2, GBP3, LAPTM5, INHBB, ADAMTS12, PTGES, TRIM6, SNX10, OPTN, TGFB2, THBS1, TIMP3, TLR3 | |  |  |  |  |  |  |  |  |  |  |  |  |  |  |  |  |  |  |  |  |  |  |  |
| Behavioral Response To Pain | | 3 | | 13 | 5.34E-04 | 0.015 | THBS1, KCNIP3, SCN9A | |  |  |  |  |  |  |  |  |  |  |  |  |  |  |  |  |  |  |  |  |  |  |  |
| Positive Regulation Of Branching Involved In Ureteric Bud Morphogenesis | | 3 | | 13 | 5.34E-04 | 0.015 | BMP4, NOG, VEGFA | |  |  |  |  |  |  |  |  |  |  |  |  |  |  |  |  |  |  |  |  |  |  |  |
| Cellular Response To Sterol | | 3 | | 13 | 5.34E-04 | 0.015 | ABCA1, INHBB, SCP2 | |  |  |  |  |  |  |  |  |  |  |  |  |  |  |  |  |  |  |  |  |  |  |  |
| Positive Regulation Of Vasculature Development | | 8 | | 3 | 5.53E-04 | 0.018 | PLK2, MIR181B1, GATA6, VEGFA, ITGB3, EFNB2, THBS1, TLR3 | |  |  |  |  |  |  |  |  |  |  |  |  |  |  |  |  |  |  |  |  |  |  |  |
| Regulation Of Dendrite Development | | 7 | | 3 | 5.58E-04 | 0.019 | PLK2, MEF2C, LPAR1, SDC2, PTPRS, CASK, ASAP1 | |  |  |  |  |  |  |  |  |  |  |  |  |  |  |  |  |  |  |  |  |  |  |  |
| Response To Abiotic Stimulus | | 23 | | 1 | 5.62E-04 | 0.013 | ARRB1, ATP2B4, TSPYL5, FBXO32, CLU, BMP4, SOX4, SDC2, SNAI2, VEGFA, LPAR1, TGFB2, THBS1, TGFBR3, TIMP3, TLR3, GATA6, ITGB3, CPEB4, MAP3K1, PDLIM1, NOG, LTBP1 | |  |  |  |  |  |  |  |  |  |  |  |  |  |  |  |  |  |  |  |  |  |  |  |
| Regulation Of Apoptotic Signaling Pathway | | 11 | | 2 | 5.76E-04 | 0.017 | NOG, SNAI2, INHBB, VEGFA, CLU, BMP4, TGFB2, THBS1, TIMP3, EYA4, TNFSF10 | |  |  |  |  |  |  |  |  |  |  |  |  |  |  |  |  |  |  |  |  |  |  |  |
| System Development | | 18 | | 1 | 6.04E-04 | 0.014 | SLC7A5, IGFBP4, CDH11, BMP4, BMP1, SOX4, MEF2C, HEG1, DPYSL2, SDC2, NOG, VEGFA, WT1, NXN, EFNB2, TGFB2, TIMP3, TLL1 | |  |  |  |  |  |  |  |  |  |  |  |  |  |  |  |  |  |  |  |  |  |  |  |
| Positive Regulation Of Sterol Transport | | 3 | | 12 | 6.07E-04 | 0.015 | ABCA1, LIPG, SCP2 | |  |  |  |  |  |  |  |  |  |  |  |  |  |  |  |  |  |  |  |  |  |  |  |
| Positive Regulation Of Cholesterol Transport | | 3 | | 12 | 6.07E-04 | 0.015 | ABCA1, LIPG, SCP2 | |  |  |  |  |  |  |  |  |  |  |  |  |  |  |  |  |  |  |  |  |  |  |  |
| Positive Regulation Of Stem Cell Differentiation | | 3 | | 12 | 6.07E-04 | 0.015 | BMP4, TGFB2, GATA6 | |  |  |  |  |  |  |  |  |  |  |  |  |  |  |  |  |  |  |  |  |  |  |  |
| Animal Organ Formation | | 3 | | 12 | 6.07E-04 | 0.015 | BMP4, GATA6, NOG | |  |  |  |  |  |  |  |  |  |  |  |  |  |  |  |  |  |  |  |  |  |  |  |
| Negative Regulation Of Programmed Cell Death | | 19 | | 1 | 6.15E-04 | 0.014 | PLK2, ARRB1, GATA6, CPEB4, CLU, BMP4, TNFSF10, MEF2C, NOG, SNAI2, OPTN, VEGFA, WT1, HHIP, TGFA, TGFB2, THBS1, TGFBR3, EYA4 | |  |  |  |  |  |  |  |  |  |  |  |  |  |  |  |  |  |  |  |  |  |  |  |
| Regulation Of Epithelial To Mesenchymal Transition | | 5 | | 5 | 6.28E-04 | 0.019 | VEGFA, BMP4, TGFB2, TGFBR3, NOG | |  |  |  |  |  |  |  |  |  |  |  |  |  |  |  |  |  |  |  |  |  |  |  |
| Immune System Process | | 36 | | 1 | 6.29E-04 | 0.012 | ADGRE5, CLU, C4BPB, DSP, CSF1, CTSD, EFNB2, F2RL1, TNFRSF21, HLA-A, HLA-B, GBP1, GBP2, GBP3, ITGB3, HLA-DRA, MEF2C, MAP3K1, LCP1, PLPP4, CPPED1, SOX4, SLC44A2, SDC2, VEGFA, TGFB2, THBS1, TGFBR3, SLAMF7, TLR3, SLC7A5, CST7, TNFSF10, CRISPLD2, FCGR2C, OPTN | |  |  |  |  |  |  |  |  |  |  |  |  |  |  |  |  |  |  |  |  |  |  |  |
| Spinal Cord Development | | 4 | | 7 | 6.33E-04 | 0.017 | SOX4, DPYSL2, PTPRS, NOG | |  |  |  |  |  |  |  |  |  |  |  |  |  |  |  |  |  |  |  |  |  |  |  |
| Response To Nitrogen Compound | | 21 | | 1 | 6.37E-04 | 0.014 | ATP2B4, ABCA1, GATA6, INHBB, ITGB3, CPEB4, SCP2, BMP4, TNFSF10, MEF2C, DPYSL2, SDC2, OPTN, VEGFA, WT1, TGFB2, THBS1, TGFBR3, TIMP3, TLR3, FBP1 | |  |  |  |  |  |  |  |  |  |  |  |  |  |  |  |  |  |  |  |  |  |  |  |
| Regulation Of Morphogenesis Of An Epithelium | | 6 | | 4 | 6.37E-04 | 0.019 | ADAMTS12, VEGFA, BMP4, NOG, PRICKLE1, SNAI2 | |  |  |  |  |  |  |  |  |  |  |  |  |  |  |  |  |  |  |  |  |  |  |  |
| Negative Regulation Of Cell Development | | 10 | | 2 | 6.48E-04 | 0.017 | PLK2, HLA-A, GBP1, NOG, ASAP1, LPAR1, BMP4, EFNB2, PTPRS, EFEMP1 | |  |  |  |  |  |  |  |  |  |  |  |  |  |  |  |  |  |  |  |  |  |  |  |
| Positive Regulation Of Metabolic Process | | 45 | | 1 | 6.52E-04 | 0.011 | PLK2, ARRB1, ATP2B4, MIR181B1, TSPYL5, CLU, BMP4, C4BPB, ARL2BP, TRIM6, CPT1A, CSF1, LPAR1, EYA4, F2RL1, ACSL1, HAS2, GATA6, PRICKLE1, IGFBP4, INHBB, TRIB2, ITGB3, CDON, MEF2C, MAP3K1, LTBP1, SCP2, SOX4, ZMIZ1, CEMIP, SNAI2, VEGFA, WT1, TGFA, TGFB2, THBS1, TLR3, CASK, TNFSF10, PDLIM1, NOG, RAD50, AFAP1L2, OPTN | |  |  |  |  |  |  |  |  |  |  |  |  |  |  |  |  |  |  |  |  |  |  |  |
| Negative Regulation Of Apoptotic Signaling Pathway | | 8 | | 2 | 6.56E-04 | 0.018 | NOG, SNAI2, VEGFA, CLU, BMP4, THBS1, EYA4, TNFSF10 | |  |  |  |  |  |  |  |  |  |  |  |  |  |  |  |  |  |  |  |  |  |  |  |
| Tube Development | | 8 | | 2 | 6.56E-04 | 0.018 | GATA6, NOG, INHBB, ITGB3, WT1, FAT4, BMP4, TGFB2 | |  |  |  |  |  |  |  |  |  |  |  |  |  |  |  |  |  |  |  |  |  |  |  |
| Regulation Of Striated Muscle Tissue Development | | 7 | | 3 | 6.64E-04 | 0.019 | CDON, MEF2C, BMP4, EFNB2, GATA6, TGFBR3, NOG | |  |  |  |  |  |  |  |  |  |  |  |  |  |  |  |  |  |  |  |  |  |  |  |
| Positive Regulation Of Cellular Component Movement | | 13 | | 1 | 6.66E-04 | 0.016 | PLK2, HAS2, MIR181B1, ITGB3, BMP4, CEMIP, CSF1, SNAI2, VEGFA, LPAR1, TGFB2, THBS1, F2RL1 | |  |  |  |  |  |  |  |  |  |  |  |  |  |  |  |  |  |  |  |  |  |  |  |
| Negative Regulation Of Camp-Mediated Signaling | | 3 | | 11 | 6.86E-04 | 0.015 | LPAR1, ATP2B4, PDE10A | |  |  |  |  |  |  |  |  |  |  |  |  |  |  |  |  |  |  |  |  |  |  |  |
| Muscular Septum Morphogenesis | | 2 | | 33 | 7.13E-04 | 0.011 | BMP4, TGFBR3 | |  |  |  |  |  |  |  |  |  |  |  |  |  |  |  |  |  |  |  |  |  |  |  |
| Negative Regulation Of Cell Proliferation Involved In Kidney Development | | 2 | | 33 | 7.13E-04 | 0.011 | BMP4, WT1 | |  |  |  |  |  |  |  |  |  |  |  |  |  |  |  |  |  |  |  |  |  |  |  |
| Bmp Signaling Pathway Involved In Heart Development | | 2 | | 33 | 7.13E-04 | 0.011 | BMP4, NOG | |  |  |  |  |  |  |  |  |  |  |  |  |  |  |  |  |  |  |  |  |  |  |  |
| Regulation Of Muscle Tissue Development | | 7 | | 3 | 7.22E-04 | 0.018 | CDON, MEF2C, BMP4, EFNB2, GATA6, TGFBR3, NOG | |  |  |  |  |  |  |  |  |  |  |  |  |  |  |  |  |  |  |  |  |  |  |  |
| Positive Regulation Of Chemotaxis | | 6 | | 3 | 7.32E-04 | 0.019 | VEGFA, LPAR1, THBS1, CSF1, F2RL1, SNAI2 | |  |  |  |  |  |  |  |  |  |  |  |  |  |  |  |  |  |  |  |  |  |  |  |
| Regulation Of Muscle Organ Development | | 7 | | 3 | 7.63E-04 | 0.018 | CDON, MEF2C, BMP4, EFNB2, GATA6, TGFBR3, NOG | |  |  |  |  |  |  |  |  |  |  |  |  |  |  |  |  |  |  |  |  |  |  |  |
| Regulation Of Metanephros Development | | 3 | | 11 | 7.71E-04 | 0.015 | FAT4, BMP4, WT1 | |  |  |  |  |  |  |  |  |  |  |  |  |  |  |  |  |  |  |  |  |  |  |  |
| Positive Regulation Of Vascular Endothelial Growth Factor Receptor Signaling Pathway | | 3 | | 11 | 7.71E-04 | 0.015 | BMP4, VEGFA, ITGB3 | |  |  |  |  |  |  |  |  |  |  |  |  |  |  |  |  |  |  |  |  |  |  |  |
| Regulation Of Branching Involved In Ureteric Bud Morphogenesis | | 3 | | 11 | 7.71E-04 | 0.015 | BMP4, NOG, VEGFA | |  |  |  |  |  |  |  |  |  |  |  |  |  |  |  |  |  |  |  |  |  |  |  |
| Epithelial Tube Branching Involved In Lung Morphogenesis | | 3 | | 11 | 7.71E-04 | 0.015 | BMP4, CTSD, HHIP | |  |  |  |  |  |  |  |  |  |  |  |  |  |  |  |  |  |  |  |  |  |  |  |
| Cholesterol Transport | | 4 | | 6 | 7.72E-04 | 0.017 | CLU, SCP2, ABCA1, LIPG | |  |  |  |  |  |  |  |  |  |  |  |  |  |  |  |  |  |  |  |  |  |  |  |
| Positive Regulation Of Nitrogen Compound Metabolic Process | | 41 | | 1 | 7.77E-04 | 0.011 | PLK2, ARRB1, ATP2B4, MIR181B1, TSPYL5, CLU, BMP4, C4BPB, ARL2BP, TRIM6, CSF1, LPAR1, EYA4, F2RL1, ACSL1, HAS2, GATA6, PRICKLE1, IGFBP4, INHBB, TRIB2, ITGB3, CDON, MEF2C, MAP3K1, SOX4, ZMIZ1, CEMIP, SNAI2, VEGFA, WT1, TGFA, TGFB2, THBS1, TLR3, CASK, TNFSF10, PDLIM1, NOG, RAD50, AFAP1L2 | |  |  |  |  |  |  |  |  |  |  |  |  |  |  |  |  |  |  |  |  |  |  |  |
| Response To Acid Chemical | | 13 | | 1 | 7.88E-04 | 0.016 | ATP2B4, ABCA1, CPEB4, BMP4, PTGES, MEF2C, CPT1A, OPTN, VEGFA, TGFB2, TGFBR3, TIMP3, ACSL1 | |  |  |  |  |  |  |  |  |  |  |  |  |  |  |  |  |  |  |  |  |  |  |  |
| Regulation Of Molecular Function | | 42 | | 1 | 8.05E-04 | 0.011 | PLK2, ARRB1, ATP2B4, CLU, BMP4, BMP1, ARL2BP, TRIM6, CSF1, TIAM2, LPAR1, LYPD6B, F2RL1, ACSL1, EFEMP1, INHBB, TRIB2, ITGB3, SH3RF2, CDON, MEF2C, MAP3K1, ASAP1, ADGRF1, PLXNA1, CEMIP, VEGFA, TGFA, TGFB2, THBS1, TGFBR3, TIMP3, TLR3, SERINC2, EVI5, GPRC5A, CST7, TNFSF10, NOG, RAD50, AFAP1L2, NUAK1 | |  |  |  |  |  |  |  |  |  |  |  |  |  |  |  |  |  |  |  |  |  |  |  |
| Positive Regulation Of Locomotion | | 13 | | 1 | 8.32E-04 | 0.016 | PLK2, HAS2, MIR181B1, ITGB3, BMP4, CEMIP, CSF1, SNAI2, VEGFA, LPAR1, TGFB2, THBS1, F2RL1 | |  |  |  |  |  |  |  |  |  |  |  |  |  |  |  |  |  |  |  |  |  |  |  |
| Positive Regulation Of Cellular Metabolic Process | | 42 | | 1 | 8.37E-04 | 0.011 | PLK2, ARRB1, ATP2B4, MIR181B1, TSPYL5, CLU, BMP4, ARL2BP, TRIM6, CPT1A, CSF1, LPAR1, EYA4, F2RL1, ACSL1, HAS2, GATA6, PRICKLE1, IGFBP4, INHBB, TRIB2, ITGB3, CDON, MEF2C, MAP3K1, SOX4, ZMIZ1, CEMIP, SNAI2, VEGFA, WT1, TGFA, TGFB2, THBS1, TLR3, CASK, TNFSF10, PDLIM1, NOG, RAD50, AFAP1L2, OPTN | |  |  |  |  |  |  |  |  |  |  |  |  |  |  |  |  |  |  |  |  |  |  |  |
| Camera-Type Eye Morphogenesis | | 3 | | 11 | 8.63E-04 | 0.015 | BMP4, TENM3, VEGFA | |  |  |  |  |  |  |  |  |  |  |  |  |  |  |  |  |  |  |  |  |  |  |  |
| Transmembrane Receptor Protein Serine/Threonine Kinase Signaling Pathway | | 7 | | 3 | 8.74E-04 | 0.018 | MAP3K1, INHBB, BMP4, TGFB2, TGFBR3, NOG, LTBP1 | |  |  |  |  |  |  |  |  |  |  |  |  |  |  |  |  |  |  |  |  |  |  |  |
| Negative Regulation Of Neurogenesis | | 9 | | 2 | 8.86E-04 | 0.017 | PLK2, HLA-A, NOG, ASAP1, LPAR1, BMP4, EFNB2, PTPRS, EFEMP1 | |  |  |  |  |  |  |  |  |  |  |  |  |  |  |  |  |  |  |  |  |  |  |  |
| Macromolecule Modification | | 41 | | 1 | 9.13E-04 | 0.011 | PLK2, ARRB1, ABCA1, MGAT4A, FBXO32, MARCH3, BMP4, PRSS23, DSP, TRIM6, CSF1, FLG, EFNB2, EYA4, F13A1, EFEMP1, IGFBP4, TRIB2, SH3RF2, MEF2C, MAP3K1, CPA4, LTBP1, HECTD4, RBM47, MAMDC2, KLHL4, PTPRS, CPPED1, SDC2, TGFB2, THBS1, TGFBR3, TGM2, TLR3, PORCN, CDK15, CDC42BPA, CASK, CTDSPL, NUAK1 | |  |  |  |  |  |  |  |  |  |  |  |  |  |  |  |  |  |  |  |  |  |  |  |
| Cardiocyte Differentiation | | 4 | | 6 | 9.31E-04 | 0.017 | MEF2C, BMP4, TGFB2, GATA6 | |  |  |  |  |  |  |  |  |  |  |  |  |  |  |  |  |  |  |  |  |  |  |  |
| Epithelial Cell Proliferation | | 5 | | 4 | 9.34E-04 | 0.018 | IGFBP4, MEF2C, VEGFA, BMP4, TGFA | |  |  |  |  |  |  |  |  |  |  |  |  |  |  |  |  |  |  |  |  |  |  |  |
| Positive Regulation Of Cell Differentiation | | 19 | | 1 | 9.59E-04 | 0.014 | HAS2, MIR181B1, GATA6, PLXNA1, TENM3, ITGB3, CLU, BMP4, CASK, CDON, ZMIZ1, MEF2C, NOG, CSF1, SNAI2, VEGFA, LPAR1, EFNB2, TGFB2 | |  |  |  |  |  |  |  |  |  |  |  |  |  |  |  |  |  |  |  |  |  |  |  |
| Negative Regulation Of Phosphate Metabolic Process | | 13 | | 1 | 9.66E-04 | 0.015 | ARRB1, GBP1, TRIB2, GPRC5A, BMP4, SH3RF2, NOG, PMEPA1, CTDSPL, TGFB2, TIMP3, F2RL1, FBP1 | |  |  |  |  |  |  |  |  |  |  |  |  |  |  |  |  |  |  |  |  |  |  |  |
| Myeloid Cell Differentiation | | 7 | | 3 | 9.72E-04 | 0.018 | MEF2C, VEGFA, BMP4, SNX10, TGFBR3, CSF1, F2RL1 | |  |  |  |  |  |  |  |  |  |  |  |  |  |  |  |  |  |  |  |  |  |  |  |
| Camera-Type Eye Development | | 5 | | 4 | 9.74E-04 | 0.018 | MAP3K1, VEGFA, WT1, BMP4, EFEMP1 | |  |  |  |  |  |  |  |  |  |  |  |  |  |  |  |  |  |  |  |  |  |  |  |
| Negative Regulation Of Phosphorus Metabolic Process | | 13 | | 1 | 9.79E-04 | 0.015 | ARRB1, GBP1, TRIB2, GPRC5A, BMP4, SH3RF2, NOG, PMEPA1, CTDSPL, TGFB2, TIMP3, F2RL1, FBP1 | |  |  |  |  |  |  |  |  |  |  |  |  |  |  |  |  |  |  |  |  |  |  |  |
| Positive Regulation Of Ras Protein Signal Transduction | | 4 | | 6 | 9.89E-04 | 0.017 | ARRB1, LPAR1, CSF1, F2RL1 | |  |  |  |  |  |  |  |  |  |  |  |  |  |  |  |  |  |  |  |  |  |  |  |
| Exocytosis | | 14 | | 1 | 1.01E-03 | 0.015 | ADGRE5, ITGB3, CLU, CPPED1, RAB27B, SLC44A2, CRISPLD2, DSP, CTSD, VEGFA, TGFB2, THBS1, TIMP3, F13A1 | |  |  |  |  |  |  |  |  |  |  |  |  |  |  |  |  |  |  |  |  |  |  |  |
| Positive Regulation Of Response To External Stimulus | | 9 | | 2 | 1.02E-03 | 0.017 | CSF1, SNAI2, OPTN, VEGFA, LPAR1, THBS1, TGM2, TLR3, F2RL1 | |  |  |  |  |  |  |  |  |  |  |  |  |  |  |  |  |  |  |  |  |  |  |  |
| Male Gonad Development | | 6 | | 3 | 1.02E-03 | 0.018 | INHBB, WT1, TGFB2, GATA6, TLR3, TNFSF10 | |  |  |  |  |  |  |  |  |  |  |  |  |  |  |  |  |  |  |  |  |  |  |  |
| Positive Regulation Of Organelle Organization | | 13 | | 1 | 1.05E-03 | 0.015 | ARRB1, BAIAP2L1, TNFSF10, MAP3K1, LCP1, SNAI2, ASAP1, RAD50, OPTN, VEGFA, LPAR1, TGFA, F2RL1 | |  |  |  |  |  |  |  |  |  |  |  |  |  |  |  |  |  |  |  |  |  |  |  |
| Positive Regulation Of Heart Growth | | 4 | | 6 | 1.05E-03 | 0.017 | MEF2C, WT1, GATA6, TGFBR3 | |  |  |  |  |  |  |  |  |  |  |  |  |  |  |  |  |  |  |  |  |  |  |  |
| Regulation Of Heart Growth | | 5 | | 4 | 1.06E-03 | 0.018 | MEF2C, WT1, GATA6, TGFBR3, NOG | |  |  |  |  |  |  |  |  |  |  |  |  |  |  |  |  |  |  |  |  |  |  |  |
| Tissue Remodeling | | 5 | | 4 | 1.10E-03 | 0.018 | MEF2C, VEGFA, TGFB2, TGFBR3, TGM2 | |  |  |  |  |  |  |  |  |  |  |  |  |  |  |  |  |  |  |  |  |  |  |  |
| Positive Regulation Of Extrinsic Apoptotic Signaling Pathway | | 4 | | 6 | 1.11E-03 | 0.017 | TGFB2, THBS1, TIMP3, TNFSF10 | |  |  |  |  |  |  |  |  |  |  |  |  |  |  |  |  |  |  |  |  |  |  |  |
| Positive Regulation Of Macromolecule Metabolic Process | | 42 | | 1 | 1.13E-03 | 0.011 | PLK2, ARRB1, ATP2B4, MIR181B1, TSPYL5, CLU, BMP4, C4BPB, ARL2BP, TRIM6, CSF1, LPAR1, EYA4, F2RL1, ACSL1, HAS2, GATA6, PRICKLE1, IGFBP4, INHBB, TRIB2, ITGB3, CDON, MEF2C, MAP3K1, SOX4, ZMIZ1, CEMIP, SNAI2, VEGFA, WT1, TGFA, TGFB2, THBS1, TLR3, CASK, TNFSF10, PDLIM1, NOG, RAD50, AFAP1L2, OPTN | |  |  |  |  |  |  |  |  |  |  |  |  |  |  |  |  |  |  |  |  |  |  |  |
| Cell-Cell Adhesion | | 11 | | 2 | 1.14E-03 | 0.016 | CDON, DSP, PDLIM1, MYL9, LAMA3, TENM3, CDH11, ITGB3, BAIAP2L1, FAT4, TGFB2 | |  |  |  |  |  |  |  |  |  |  |  |  |  |  |  |  |  |  |  |  |  |  |  |
| Glycosaminoglycan Metabolic Process | | 6 | | 3 | 1.15E-03 | 0.018 | CEMIP, VEGFA, HAS2, SDC2, MAMDC2, CHST15 | |  |  |  |  |  |  |  |  |  |  |  |  |  |  |  |  |  |  |  |  |  |  |  |
| Negative Regulation Of Cell Projection Organization | | 7 | | 3 | 1.16E-03 | 0.018 | PLK2, HLA-A, ASAP1, LPAR1, EFNB2, PTPRS, EFEMP1 | |  |  |  |  |  |  |  |  |  |  |  |  |  |  |  |  |  |  |  |  |  |  |  |
| Sterol Transport | | 4 | | 6 | 1.18E-03 | 0.017 | CLU, SCP2, ABCA1, LIPG | |  |  |  |  |  |  |  |  |  |  |  |  |  |  |  |  |  |  |  |  |  |  |  |
| Positive Regulation Of Neural Precursor Cell Proliferation | | 4 | | 6 | 1.18E-03 | 0.017 | CDON, OPTN, VEGFA, NOG | |  |  |  |  |  |  |  |  |  |  |  |  |  |  |  |  |  |  |  |  |  |  |  |
| Regulation Of Morphogenesis Of A Branching Structure | | 4 | | 6 | 1.18E-03 | 0.017 | VEGFA, BMP4, NOG, SNAI2 | |  |  |  |  |  |  |  |  |  |  |  |  |  |  |  |  |  |  |  |  |  |  |  |
| Regulation Of Myoblast Differentiation | | 4 | | 6 | 1.18E-03 | 0.017 | CDON, MEF2C, BMP4, PRICKLE1 | |  |  |  |  |  |  |  |  |  |  |  |  |  |  |  |  |  |  |  |  |  |  |  |
| Regulation Of Endothelial Cell Chemotaxis | | 3 | | 10 | 1.18E-03 | 0.015 | VEGFA, THBS1, SNAI2 | |  |  |  |  |  |  |  |  |  |  |  |  |  |  |  |  |  |  |  |  |  |  |  |
| Regulation Of Developmental Growth | | 10 | | 2 | 1.18E-03 | 0.016 | MEF2C, DPYSL2, GATA6, NOG, CSF1, VEGFA, WT1, BMP4, PTPRS, TGFBR3 | |  |  |  |  |  |  |  |  |  |  |  |  |  |  |  |  |  |  |  |  |  |  |  |
| Regulation Of Cell-Substrate Adhesion | | 7 | | 3 | 1.19E-03 | 0.018 | HAS2, GBP1, CSF1, VEGFA, ITGB3, THBS1, CASK | |  |  |  |  |  |  |  |  |  |  |  |  |  |  |  |  |  |  |  |  |  |  |  |
| Regulation Of Cell Morphogenesis Involved In Differentiation | | 9 | | 2 | 1.21E-03 | 0.017 | DPYSL2, HAS2, SDC2, GBP1, PLXNA1, VEGFA, ITGB3, PTPRS, CASK | |  |  |  |  |  |  |  |  |  |  |  |  |  |  |  |  |  |  |  |  |  |  |  |
| Response To Interleukin-1 | | 6 | | 3 | 1.22E-03 | 0.018 | ADAMTS12, INHBB, HAS2, GBP1, GBP2, GBP3 | |  |  |  |  |  |  |  |  |  |  |  |  |  |  |  |  |  |  |  |  |  |  |  |
| Negative Regulation Of Immune System Process | | 11 | | 2 | 1.23E-03 | 0.016 | TNFRSF21, ZNF608, HLA-A, GBP1, BMP4, PTPRS, TGFB2, THBS1, C4BPB, TLR3, F2RL1 | |  |  |  |  |  |  |  |  |  |  |  |  |  |  |  |  |  |  |  |  |  |  |  |
| Regulation Of Immune System Process | | 26 | | 1 | 1.24E-03 | 0.012 | MYL9, CLU, BMP4, PTPRS, C4BPB, ZMIZ1, MTUS1, ZNF608, TRIM6, CSF1, VEGFA, EFNB2, TGFB2, THBS1, SLAMF7, TLR3, F2RL1, TNFRSF21, HLA-A, HLA-B, GBP1, ITGB3, HLA-DRA, MEF2C, MAP3K1, PLPP4 | |  |  |  |  |  |  |  |  |  |  |  |  |  |  |  |  |  |  |  |  |  |  |  |
| Regulation Of Macrophage Chemotaxis | | 3 | | 9 | 1.30E-03 | 0.015 | MTUS1, THBS1, CSF1 | |  |  |  |  |  |  |  |  |  |  |  |  |  |  |  |  |  |  |  |  |  |  |  |
| Regulation Of Macrophage Derived Foam Cell Differentiation | | 3 | | 9 | 1.30E-03 | 0.015 | ITGB3, ABCA1, CSF1 | |  |  |  |  |  |  |  |  |  |  |  |  |  |  |  |  |  |  |  |  |  |  |  |
| Response To Cholesterol | | 3 | | 9 | 1.30E-03 | 0.015 | INHBB, SCP2, ABCA1 | |  |  |  |  |  |  |  |  |  |  |  |  |  |  |  |  |  |  |  |  |  |  |  |
| Cell Differentiation Involved In Kidney Development | | 3 | | 9 | 1.30E-03 | 0.015 | MEF2C, WT1, BMP4 | |  |  |  |  |  |  |  |  |  |  |  |  |  |  |  |  |  |  |  |  |  |  |  |
| Eye Morphogenesis | | 4 | | 5 | 1.32E-03 | 0.017 | TENM3, VEGFA, BMP4, EFEMP1 | |  |  |  |  |  |  |  |  |  |  |  |  |  |  |  |  |  |  |  |  |  |  |  |
| Blood Vessel Endothelial Cell Proliferation Involved In Sprouting Angiogenesis | | 2 | | 25 | 1.32E-03 | 0.011 | BMP4, VEGFA | |  |  |  |  |  |  |  |  |  |  |  |  |  |  |  |  |  |  |  |  |  |  |  |
| Hyaluronan Biosynthetic Process | | 2 | | 25 | 1.32E-03 | 0.011 | CEMIP, HAS2 | |  |  |  |  |  |  |  |  |  |  |  |  |  |  |  |  |  |  |  |  |  |  |  |
| Regulation Of Transforming Growth Factor Beta2 Production | | 2 | | 25 | 1.32E-03 | 0.011 | TGFB2, GATA6 | |  |  |  |  |  |  |  |  |  |  |  |  |  |  |  |  |  |  |  |  |  |  |  |
| Regulation Of Epithelial To Mesenchymal Transition Involved In Endocardial Cushion Formation | | 2 | | 25 | 1.32E-03 | 0.011 | TGFB2, NOG | |  |  |  |  |  |  |  |  |  |  |  |  |  |  |  |  |  |  |  |  |  |  |  |
| Negative Regulation Of Apoptotic Process Involved In Development | | 2 | | 25 | 1.32E-03 | 0.011 | TGFBR3, WT1 | |  |  |  |  |  |  |  |  |  |  |  |  |  |  |  |  |  |  |  |  |  |  |  |
| Interleukin-1 Beta Secretion | | 2 | | 25 | 1.32E-03 | 0.011 | ABCA1, F2RL1 | |  |  |  |  |  |  |  |  |  |  |  |  |  |  |  |  |  |  |  |  |  |  |  |
| Mucopolysaccharide Metabolic Process | | 5 | | 4 | 1.34E-03 | 0.017 | CEMIP, VEGFA, HAS2, MAMDC2, CHST15 | |  |  |  |  |  |  |  |  |  |  |  |  |  |  |  |  |  |  |  |  |  |  |  |
| Positive Regulation Of Angiogenesis | | 7 | | 2 | 1.35E-03 | 0.017 | PLK2, MIR181B1, GATA6, VEGFA, ITGB3, THBS1, TLR3 | |  |  |  |  |  |  |  |  |  |  |  |  |  |  |  |  |  |  |  |  |  |  |  |
| Regulation Of Smooth Muscle Cell Migration | | 5 | | 4 | 1.39E-03 | 0.017 | MEF2C, HAS2, ITGB3, LPAR1, PLXNA1 | |  |  |  |  |  |  |  |  |  |  |  |  |  |  |  |  |  |  |  |  |  |  |  |
| Epithelial Cell Differentiation | | 10 | | 2 | 1.40E-03 | 0.016 | SOX4, TDRD7, MEF2C, DSP, GATA6, CPT1A, FLG, VEGFA, WT1, BMP4 | |  |  |  |  |  |  |  |  |  |  |  |  |  |  |  |  |  |  |  |  |  |  |  |
| Response To Vitamin | | 6 | | 3 | 1.42E-03 | 0.017 | CYP24A1, MEF2C, VEGFA, TGFB2, TIMP3, LTBP1 | |  |  |  |  |  |  |  |  |  |  |  |  |  |  |  |  |  |  |  |  |  |  |  |
| Outflow Tract Septum Morphogenesis | | 3 | | 9 | 1.43E-03 | 0.015 | BMP4, TGFB2, GATA6 | |  |  |  |  |  |  |  |  |  |  |  |  |  |  |  |  |  |  |  |  |  |  |  |
| Negative Regulation Of Phosphorylation | | 11 | | 2 | 1.47E-03 | 0.016 | ARRB1, GBP1, NOG, PMEPA1, CTDSPL, TRIB2, GPRC5A, BMP4, TIMP3, F2RL1, FBP1 | |  |  |  |  |  |  |  |  |  |  |  |  |  |  |  |  |  |  |  |  |  |  |  |
| Response To Decreased Oxygen Levels | | 10 | | 2 | 1.54E-03 | 0.016 | SOX4, PDLIM1, SDC2, GATA6, NOG, VEGFA, CPEB4, TGFB2, THBS1, TGFBR3 | |  |  |  |  |  |  |  |  |  |  |  |  |  |  |  |  |  |  |  |  |  |  |  |
| Regulation Of Cardiac Muscle Cell Proliferation | | 4 | | 5 | 1.55E-03 | 0.016 | MEF2C, GATA6, TGFBR3, NOG | |  |  |  |  |  |  |  |  |  |  |  |  |  |  |  |  |  |  |  |  |  |  |  |
| Regulation Of Response To Stress | | 23 | | 1 | 1.55E-03 | 0.012 | PLK2, ATP2B4, MIR181B1, CLU, PTPRS, C4BPB, TRIM6, SNAI2, VEGFA, TGFB2, THBS1, TGFBR3, TGM2, EYA4, TLR3, F2RL1, HLA-A, CASK, ADAMTS12, MEF2C, MAP3K1, OPTN, NUAK1 | |  |  |  |  |  |  |  |  |  |  |  |  |  |  |  |  |  |  |  |  |  |  |  |
| Cellular Response To Cytokine Stimulus | | 14 | | 1 | 1.55E-03 | 0.014 | TNFRSF21, HAS2, HLA-A, GBP1, GBP2, GBP3, LAPTM5, INHBB, ADAMTS12, TRIM6, SNX10, OPTN, THBS1, TLR3 | |  |  |  |  |  |  |  |  |  |  |  |  |  |  |  |  |  |  |  |  |  |  |  |
| Regulation Of Endothelial Cell Migration | | 7 | | 2 | 1.56E-03 | 0.017 | PLK2, MEF2C, SNAI2, VEGFA, ITGB3, BMP4, THBS1 | |  |  |  |  |  |  |  |  |  |  |  |  |  |  |  |  |  |  |  |  |  |  |  |
| Regulation Of Transforming Growth Factor Beta Production | | 3 | | 9 | 1.56E-03 | 0.014 | TGFB2, GATA6, THBS1 | |  |  |  |  |  |  |  |  |  |  |  |  |  |  |  |  |  |  |  |  |  |  |  |
| Positive Regulation Of Rho Protein Signal Transduction | | 3 | | 9 | 1.56E-03 | 0.014 | ARRB1, LPAR1, F2RL1 | |  |  |  |  |  |  |  |  |  |  |  |  |  |  |  |  |  |  |  |  |  |  |  |
| Regulation Of Cell-Cell Adhesion | | 10 | | 2 | 1.56E-03 | 0.016 | TNFRSF21, ZMIZ1, HAS2, ZNF608, HLA-A, SNAI2, TENM3, VEGFA, BMP4, EFNB2 | |  |  |  |  |  |  |  |  |  |  |  |  |  |  |  |  |  |  |  |  |  |  |  |
| Regulation Of Protein Secretion | | 11 | | 2 | 1.58E-03 | 0.015 | SOX4, TNFRSF21, ARRB1, ARL2BP, TRIM6, GBP1, CPT1A, INHBB, TGFB2, CASK, F2RL1 | |  |  |  |  |  |  |  |  |  |  |  |  |  |  |  |  |  |  |  |  |  |  |  |
| Response To Oxygen Levels | | 11 | | 2 | 1.58E-03 | 0.015 | SOX4, PDLIM1, SDC2, GATA6, NOG, VEGFA, CPEB4, LPAR1, TGFB2, THBS1, TGFBR3 | |  |  |  |  |  |  |  |  |  |  |  |  |  |  |  |  |  |  |  |  |  |  |  |
| Regulation Of Catalytic Activity | | 32 | | 1 | 1.59E-03 | 0.011 | ARRB1, ATP2B4, PLXNA1, CLU, BMP4, CEMIP, ARL2BP, CSF1, TIAM2, VEGFA, LPAR1, TGFA, TGFB2, THBS1, TIMP3, TLR3, F2RL1, ACSL1, SERINC2, EVI5, TRIB2, ITGB3, GPRC5A, CST7, SH3RF2, TNFSF10, MEF2C, MAP3K1, ASAP1, RAD50, AFAP1L2, NUAK1 | |  |  |  |  |  |  |  |  |  |  |  |  |  |  |  |  |  |  |  |  |  |  |  |
| Regulation Of Cellular Component Biogenesis | | 17 | | 1 | 1.59E-03 | 0.014 | PLK2, HAS2, ABCA1, EVI5, ADGRE5, BAIAP2L1, CLU, MEF2C, MAP3K1, LCP1, PMEPA1, SNAI2, ASAP1, VEGFA, LPAR1, THBS1, F2RL1 | |  |  |  |  |  |  |  |  |  |  |  |  |  |  |  |  |  |  |  |  |  |  |  |
| Cellular Response To Endogenous Stimulus | | 17 | | 1 | 1.62E-03 | 0.014 | ATP2B4, HAS2, GATA6, FBXO32, INHBB, ITGB3, CPEB4, BMP4, ADAMTS12, MEF2C, NOG, SNAI2, LTBP1, OPTN, VEGFA, WT1, FBP1 | |  |  |  |  |  |  |  |  |  |  |  |  |  |  |  |  |  |  |  |  |  |  |  |
| Regulation Of Interleukin-8 Production | | 4 | | 5 | 1.63E-03 | 0.016 | AFAP1L2, ARRB1, TLR3, F2RL1 | |  |  |  |  |  |  |  |  |  |  |  |  |  |  |  |  |  |  |  |  |  |  |  |
| Positive Regulation Of Epithelial Cell Proliferation | | 7 | | 2 | 1.63E-03 | 0.017 | HAS2, NOG, VEGFA, ITGB3, SCN5A, BMP4, TGFA | |  |  |  |  |  |  |  |  |  |  |  |  |  |  |  |  |  |  |  |  |  |  |  |
| Positive Regulation Of Cellular Process | | 63 | | 0 | 1.65E-03 | 0.010 | ARRB1, ATP2B4, TSPYL5, ADGRE5, FBXO32, CLU, BMP4, DPYSL2, TRIM6, CPT1A, CSF1, LPAR1, EFNB2, EYA4, F2RL1, ACSL1, EFEMP1, HAS2, HLA-A, GATA6, PRICKLE1, IGFBP4, INHBB, ITGB3, MEF2C, MAP3K1, LCP1, PLXNA1, SCN5A, SCP2, RAB27B, SOX4, SNAI2, VEGFA, WT1, TGFA, TGFB2, THBS1, TGFBR3, TGM2, TIMP3, TLR3, CASK, TNFSF10, PDLIM1, NOG, RAD50, OPTN, PLK2, MIR181B1, ARL2BP, TIAM2, TRIB2, CDON, ASAP1, TENM3, BAIAP2L1, SLC44A2, ZMIZ1, CEMIP, TNS3, COLGALT1, AFAP1L2 | |  |  |  |  |  |  |  |  |  |  |  |  |  |  |  |  |  |  |  |  |  |  |  |
| Regulation Of Ion Transport | | 14 | | 1 | 1.68E-03 | 0.014 | ATP2B4, CLIC3, ITGB3, SCN5A, SCN9A, BMP4, CASK, MEF2C, CEMIP, DPYSL2, KCNIP3, CLIC6, TGFB2, THBS1 | |  |  |  |  |  |  |  |  |  |  |  |  |  |  |  |  |  |  |  |  |  |  |  |
| Antigen Processing And Presentation Of Exogenous Peptide Antigen Via Mhc Class I, Tap-Independent | | 2 | | 22 | 1.69E-03 | 0.011 | HLA-A, HLA-B | |  |  |  |  |  |  |  |  |  |  |  |  |  |  |  |  |  |  |  |  |  |  |  |
| Positive Regulation Of Cytokine Secretion Involved In Immune Response | | 2 | | 22 | 1.69E-03 | 0.011 | TRIM6, F2RL1 | |  |  |  |  |  |  |  |  |  |  |  |  |  |  |  |  |  |  |  |  |  |  |  |
| Heart Field Specification | | 2 | | 22 | 1.69E-03 | 0.011 | BMP4, MEF2C | |  |  |  |  |  |  |  |  |  |  |  |  |  |  |  |  |  |  |  |  |  |  |  |
| Secondary Heart Field Specification | | 2 | | 22 | 1.69E-03 | 0.011 | BMP4, MEF2C | |  |  |  |  |  |  |  |  |  |  |  |  |  |  |  |  |  |  |  |  |  |  |  |
| Regulation Of Cardiac Epithelial To Mesenchymal Transition | | 2 | | 22 | 1.69E-03 | 0.011 | TGFB2, NOG | |  |  |  |  |  |  |  |  |  |  |  |  |  |  |  |  |  |  |  |  |  |  |  |
| Visceral Serous Pericardium Development | | 2 | | 22 | 1.69E-03 | 0.011 | TGFBR3, WT1 | |  |  |  |  |  |  |  |  |  |  |  |  |  |  |  |  |  |  |  |  |  |  |  |
| Cardiac Ventricle Morphogenesis | | 3 | | 8 | 1.70E-03 | 0.014 | SOX4, BMP4, TGFB2 | |  |  |  |  |  |  |  |  |  |  |  |  |  |  |  |  |  |  |  |  |  |  |  |
| Negative Regulation Of Cellular Metabolic Process | | 36 | | 1 | 1.71E-03 | 0.011 | ARRB1, ATP2B4, MIR181B1, CLU, BMP4, C4BPB, TRIM6, CST4, F2RL1, FBP1, GATA6, GBP1, PRICKLE1, INHBB, TRIB2, ITGB3, SH3RF2, MEF2C, KCNIP3, SOX4, ZNF608, PMEPA1, SNAI2, VEGFA, WT1, NXN, TGFB2, THBS1, TIMP3, CPEB4, GPRC5A, CST7, NOG, RAD50, OPTN, CTDSPL | |  |  |  |  |  |  |  |  |  |  |  |  |  |  |  |  |  |  |  |  |  |  |  |
| Negative Regulation Of Wnt Signaling Pathway | | 6 | | 3 | 1.79E-03 | 0.017 | IGFBP4, SHISA2, NXN, NOG, PRICKLE1, SNAI2 | |  |  |  |  |  |  |  |  |  |  |  |  |  |  |  |  |  |  |  |  |  |  |  |
| Regulation Of Neuron Death | | 9 | | 2 | 1.79E-03 | 0.016 | ARRB1, MEF2C, KCNIP3, OPTN, VEGFA, CPEB4, CLU, EFNB2, TGFB2 | |  |  |  |  |  |  |  |  |  |  |  |  |  |  |  |  |  |  |  |  |  |  |  |
| Vesicle-Mediated Transport | | 24 | | 1 | 1.79E-03 | 0.012 | ARRB1, TMPRSS15, ABCA1, ADGRE5, MARCH3, CLU, CPPED1, RAB27B, SLC44A2, DPYSL2, DSP, CTSD, VEGFA, TGFA, TGFB2, THBS1, TGM2, TIMP3, F13A1, EVI5, ITGB3, CRISPLD2, SNX10, OPTN | |  |  |  |  |  |  |  |  |  |  |  |  |  |  |  |  |  |  |  |  |  |  |  |
| Regulation Of Cell-Matrix Adhesion | | 5 | | 4 | 1.80E-03 | 0.017 | VEGFA, ITGB3, THBS1, CASK, CSF1 | |  |  |  |  |  |  |  |  |  |  |  |  |  |  |  |  |  |  |  |  |  |  |  |
| Cell Motility | | 18 | | 1 | 1.81E-03 | 0.013 | SLC7A5, ATP2B4, TNS3, ITGB3, CDC42BPA, ADAMTS12, MEF2C, SDC2, LCP1, SNAI2, VEGFA, WT1, LPAR1, EFNB2, TGFB2, THBS1, TGFBR3, F2RL1 | |  |  |  |  |  |  |  |  |  |  |  |  |  |  |  |  |  |  |  |  |  |  |  |
| Locomotion | | 19 | | 1 | 1.82E-03 | 0.013 | SLC7A5, ATP2B4, TNS3, ITGB3, BMP4, CDC42BPA, ADAMTS12, MEF2C, SDC2, LCP1, SNAI2, VEGFA, WT1, LPAR1, EFNB2, TGFB2, THBS1, TGFBR3, F2RL1 | |  |  |  |  |  |  |  |  |  |  |  |  |  |  |  |  |  |  |  |  |  |  |  |
| Defense Response To Protozoan | | 3 | | 8 | 1.85E-03 | 0.014 | GBP1, GBP2, GBP3 | |  |  |  |  |  |  |  |  |  |  |  |  |  |  |  |  |  |  |  |  |  |  |  |
| Response To Nutrient Levels | | 13 | | 1 | 1.89E-03 | 0.015 | ABCA1, INHBB, CPEB4, CYP24A1, MEF2C, CTSD, LIPG, LTBP1, OPTN, VEGFA, TGFB2, TIMP3, ACSL1 | |  |  |  |  |  |  |  |  |  |  |  |  |  |  |  |  |  |  |  |  |  |  |  |
| Regulation Of Protein Transport | | 14 | | 1 | 1.92E-03 | 0.014 | TNFRSF21, ARRB1, GBP1, INHBB, BMP4, CASK, SOX4, CEMIP, ARL2BP, TRIM6, CPT1A, LCP1, TGFB2, F2RL1 | |  |  |  |  |  |  |  |  |  |  |  |  |  |  |  |  |  |  |  |  |  |  |  |
| Regulation Of Leukocyte Chemotaxis | | 5 | | 3 | 1.93E-03 | 0.017 | VEGFA, MTUS1, THBS1, CSF1, F2RL1 | |  |  |  |  |  |  |  |  |  |  |  |  |  |  |  |  |  |  |  |  |  |  |  |
| Cellular Protein Metabolic Process | | 43 | | 1 | 1.94E-03 | 0.010 | PLK2, ARRB1, ABCA1, MGAT4A, FBXO32, MARCH3, BMP4, PRSS23, DSP, TRIM6, CSF1, FLG, EFNB2, EYA4, F13A1, EFEMP1, GATA6, IGFBP4, TRIB2, SH3RF2, MEF2C, MAP3K1, CPA4, LTBP1, HECTD4, MAMDC2, RRBP1, KLHL4, PTPRS, CPPED1, SDC2, TGFB2, THBS1, TGFBR3, TGM2, TLR3, PORCN, CDK15, CPEB4, CDC42BPA, CASK, CTDSPL, NUAK1 | |  |  |  |  |  |  |  |  |  |  |  |  |  |  |  |  |  |  |  |  |  |  |  |
| Aminoglycan Metabolic Process | | 6 | | 3 | 1.99E-03 | 0.017 | CEMIP, VEGFA, HAS2, SDC2, MAMDC2, CHST15 | |  |  |  |  |  |  |  |  |  |  |  |  |  |  |  |  |  |  |  |  |  |  |  |
| Protein Metabolic Process | | 52 | | 1 | 2.01E-03 | 0.010 | PLK2, ARRB1, ABCA1, MGAT4A, FBXO32, MARCH3, CLU, BMP4, BMP1, C4BPB, PRSS23, DSP, TRIM6, CSF1, CTSD, FLG, EFNB2, EYA4, F13A1, EFEMP1, GATA6, IGFBP4, TRIB2, SH3RF2, MEF2C, MAP3K1, CPA4, LTBP1, HECTD4, TMPRSS15, MAMDC2, TENM3, RRBP1, KLHL4, PTPRS, CPPED1, SDC2, TGFB2, THBS1, TGFBR3, TGM2, TLL1, TLR3, PORCN, CDK15, CPEB4, CDC42BPA, CASK, ADAMTS12, APOL6, CTDSPL, NUAK1 | |  |  |  |  |  |  |  |  |  |  |  |  |  |  |  |  |  |  |  |  |  |  |  |
| Regulation Of Macrophage Migration | | 3 | | 8 | 2.01E-03 | 0.014 | MTUS1, THBS1, CSF1 | |  |  |  |  |  |  |  |  |  |  |  |  |  |  |  |  |  |  |  |  |  |  |  |
| Negative Regulation Of Nitrogen Compound Metabolic Process | | 34 | | 1 | 2.04E-03 | 0.011 | ARRB1, ATP2B4, MIR181B1, CLU, BMP4, C4BPB, TRIM6, CST4, F2RL1, FBP1, GATA6, GBP1, PRICKLE1, INHBB, TRIB2, ITGB3, SH3RF2, MEF2C, KCNIP3, SOX4, ZNF608, PMEPA1, SNAI2, VEGFA, WT1, NXN, THBS1, TIMP3, CPEB4, GPRC5A, CST7, NOG, RAD50, CTDSPL | |  |  |  |  |  |  |  |  |  |  |  |  |  |  |  |  |  |  |  |  |  |  |  |
| Regulation Of Ossification | | 7 | | 2 | 2.05E-03 | 0.017 | MEF2C, NOG, CSF1, SNAI2, VEGFA, BMP4, TGFB2 | |  |  |  |  |  |  |  |  |  |  |  |  |  |  |  |  |  |  |  |  |  |  |  |
| Negative Regulation Of Cell Proliferation | | 15 | | 1 | 2.06E-03 | 0.014 | TNFRSF21, SCP2, BMP4, CASK, SOX4, PTGES, MEF2C, NOG, SNAI2, OPTN, WT1, EFNB2, TGFB2, THBS1, TGFBR3 | |  |  |  |  |  |  |  |  |  |  |  |  |  |  |  |  |  |  |  |  |  |  |  |
| Negative Regulation Of Cellular Component Organization | | 14 | | 1 | 2.06E-03 | 0.014 | PLK2, HLA-A, GBP1, CLU, BMP4, PTPRS, MAP3K1, PMEPA1, ASAP1, RAD50, LPAR1, EFNB2, THBS1, EFEMP1 | |  |  |  |  |  |  |  |  |  |  |  |  |  |  |  |  |  |  |  |  |  |  |  |
| Cellular Response To Interferon-Gamma | | 5 | | 3 | 2.07E-03 | 0.017 | HLA-A, GBP1, GBP2, GBP3, TLR3 | |  |  |  |  |  |  |  |  |  |  |  |  |  |  |  |  |  |  |  |  |  |  |  |
| Positive Regulation Of Organ Growth | | 4 | | 5 | 2.08E-03 | 0.016 | MEF2C, WT1, GATA6, TGFBR3 | |  |  |  |  |  |  |  |  |  |  |  |  |  |  |  |  |  |  |  |  |  |  |  |
| Negative Regulation Of Metanephros Development | | 2 | | 20 | 2.10E-03 | 0.011 | BMP4, WT1 | |  |  |  |  |  |  |  |  |  |  |  |  |  |  |  |  |  |  |  |  |  |  |  |
| Desmosome Organization | | 2 | | 20 | 2.10E-03 | 0.011 | SNAI2, DSP | |  |  |  |  |  |  |  |  |  |  |  |  |  |  |  |  |  |  |  |  |  |  |  |
| Cardiac Ventricle Formation | | 2 | | 20 | 2.10E-03 | 0.011 | SOX4, MEF2C | |  |  |  |  |  |  |  |  |  |  |  |  |  |  |  |  |  |  |  |  |  |  |  |
| Regulation Of Cgmp-Mediated Signaling | | 2 | | 20 | 2.10E-03 | 0.011 | THBS1, PDE10A | |  |  |  |  |  |  |  |  |  |  |  |  |  |  |  |  |  |  |  |  |  |  |  |
| Regulation Of Transforming Growth Factor Beta1 Production | | 2 | | 20 | 2.10E-03 | 0.011 | GATA6, THBS1 | |  |  |  |  |  |  |  |  |  |  |  |  |  |  |  |  |  |  |  |  |  |  |  |
| Negative Regulation Of T Cell Differentiation In Thymus | | 2 | | 20 | 2.10E-03 | 0.011 | BMP4, ZNF608 | |  |  |  |  |  |  |  |  |  |  |  |  |  |  |  |  |  |  |  |  |  |  |  |
| Interleukin-1 Secretion | | 2 | | 20 | 2.10E-03 | 0.011 | ABCA1, F2RL1 | |  |  |  |  |  |  |  |  |  |  |  |  |  |  |  |  |  |  |  |  |  |  |  |
| Phospholipid Homeostasis | | 2 | | 20 | 2.10E-03 | 0.011 | ABCA1, LIPG | |  |  |  |  |  |  |  |  |  |  |  |  |  |  |  |  |  |  |  |  |  |  |  |
| Response To Hormone | | 17 | | 1 | 2.14E-03 | 0.013 | GATA6, FBXO32, INHBB, ITGB3, SCP2, BMP4, TNFSF10, MEF2C, LTBP1, RAD50, VEGFA, WT1, TGFB2, THBS1, TGFBR3, TIMP3, FBP1 | |  |  |  |  |  |  |  |  |  |  |  |  |  |  |  |  |  |  |  |  |  |  |  |
| Response To Sterol | | 3 | | 8 | 2.18E-03 | 0.014 | INHBB, SCP2, ABCA1 | |  |  |  |  |  |  |  |  |  |  |  |  |  |  |  |  |  |  |  |  |  |  |  |
| Smooth Muscle Cell Differentiation | | 3 | | 8 | 2.18E-03 | 0.014 | MEF2C, BMP4, GATA6 | |  |  |  |  |  |  |  |  |  |  |  |  |  |  |  |  |  |  |  |  |  |  |  |
| Regulation Of Primary Metabolic Process | | 69 | | 0 | 2.18E-03 | 0.009 | ARRB1, ATP2B4, ABCA1, TSPYL5, CLU, BMP4, C4BPB, TRIM6, CPT1A, CSF1, CST4, LPAR1, EYA4, F2RL1, ACSL1, EFEMP1, FBP1, HAS2, GATA6, GBP1, PRICKLE1, IGFBP4, INHBB, ITGB3, SH3RF2, MEF2C, MEIS3P1, MAP3K1, SAMD9L, ADGRF1, SCP2, SOX4, SNAI2, VEGFA, WT1, TGFA, TGFB2, THBS1, TGFBR3, TIMP3, TLR3, ZNF391, ZNF43, GPRC5A, CST7, CASK, TNFSF10, PDLIM1, NOG, LIPG, RAD50, MBNL2, CTDSPL, NUAK1, PLK2, MIR181B1, ARL2BP, TIAM2, TNFRSF21, TRIB2, CDON, KCNIP3, ZMIZ1, CEMIP, ZNF608, PMEPA1, NXN, CPEB4, AFAP1L2 | |  |  |  |  |  |  |  |  |  |  |  |  |  |  |  |  |  |  |  |  |  |  |  |
| Sensory Organ Morphogenesis | | 4 | | 5 | 2.18E-03 | 0.016 | TENM3, VEGFA, BMP4, EFEMP1 | |  |  |  |  |  |  |  |  |  |  |  |  |  |  |  |  |  |  |  |  |  |  |  |
| Positive Regulation Of Smooth Muscle Cell Proliferation | | 5 | | 3 | 2.21E-03 | 0.017 | VEGFA, ITGB3, BMP4, THBS1, TGM2 | |  |  |  |  |  |  |  |  |  |  |  |  |  |  |  |  |  |  |  |  |  |  |  |
| Positive Regulation Of Stress-Activated Mapk Cascade | | 6 | | 3 | 2.22E-03 | 0.017 | MAP3K1, VEGFA, MIR181B1, TGFB2, TLR3, F2RL1 | |  |  |  |  |  |  |  |  |  |  |  |  |  |  |  |  |  |  |  |  |  |  |  |
| Positive Regulation Of Cell Adhesion | | 10 | | 2 | 2.23E-03 | 0.015 | ZMIZ1, HAS2, HLA-A, CSF1, VEGFA, ITGB3, EFNB2, TGFB2, THBS1, TGM2 | |  |  |  |  |  |  |  |  |  |  |  |  |  |  |  |  |  |  |  |  |  |  |  |
| Regulation Of Biological Quality | | 46 | | 1 | 2.23E-03 | 0.010 | PLK2, ARRB1, ATP2B4, MIR181B1, ABCA1, CLU, BMP4, C4BPB, ARL2BP, DSP, TRIM6, CPT1A, CSF1, CST4, FLG, LPAR1, F2RL1, F13A1, HAS2, GATA6, INHBB, ITGB3, MEF2C, MAP3K1, SNX10, LTBP1, HECTD4, PLXNA1, BAIAP2L1, SCN5A, SCP2, SCN9A, ACOXL, SOX4, CEMIP, HEG1, VEGFA, NXN, TGFB2, THBS1, TGFBR3, TGM2, ILDR2, CASK, LIPG, RAD50 | |  |  |  |  |  |  |  |  |  |  |  |  |  |  |  |  |  |  |  |  |  |  |  |
| Positive Regulation Of Stress-Activated Protein Kinase Signaling Cascade | | 6 | | 3 | 2.28E-03 | 0.017 | MAP3K1, VEGFA, MIR181B1, TGFB2, TLR3, F2RL1 | |  |  |  |  |  |  |  |  |  |  |  |  |  |  |  |  |  |  |  |  |  |  |  |
| Striated Muscle Cell Differentiation | | 4 | | 5 | 2.29E-03 | 0.016 | CDON, MEF2C, BMP4, GATA6 | |  |  |  |  |  |  |  |  |  |  |  |  |  |  |  |  |  |  |  |  |  |  |  |
| Regulation Of Protein Binding | | 7 | | 2 | 2.34E-03 | 0.016 | CDON, PLK2, ARRB1, MEF2C, NOG, BMP4, TGFBR3 | |  |  |  |  |  |  |  |  |  |  |  |  |  |  |  |  |  |  |  |  |  |  |  |
| Negative Regulation Of Dendrite Development | | 3 | | 7 | 2.35E-03 | 0.014 | PLK2, PTPRS, ASAP1 | |  |  |  |  |  |  |  |  |  |  |  |  |  |  |  |  |  |  |  |  |  |  |  |
| Positive Regulation Of Bmp Signaling Pathway | | 3 | | 7 | 2.35E-03 | 0.014 | BMP4, GATA6, TGFBR3 | |  |  |  |  |  |  |  |  |  |  |  |  |  |  |  |  |  |  |  |  |  |  |  |
| Pituitary Gland Development | | 3 | | 7 | 2.35E-03 | 0.014 | INHBB, BMP4, NOG | |  |  |  |  |  |  |  |  |  |  |  |  |  |  |  |  |  |  |  |  |  |  |  |
| Positive Regulation Of Morphogenesis Of An Epithelium | | 3 | | 7 | 2.35E-03 | 0.014 | VEGFA, BMP4, NOG | |  |  |  |  |  |  |  |  |  |  |  |  |  |  |  |  |  |  |  |  |  |  |  |
| Regulation Of Transmembrane Transport | | 12 | | 1 | 2.40E-03 | 0.015 | ATP2B4, CLIC3, ITGB3, SCN5A, SCN9A, BMP4, MEF2C, CEMIP, KCNIP3, CLIC6, TGFB2, THBS1 | |  |  |  |  |  |  |  |  |  |  |  |  |  |  |  |  |  |  |  |  |  |  |  |
| Negative Regulation Of Protein Phosphorylation | | 10 | | 2 | 2.43E-03 | 0.015 | ARRB1, GBP1, NOG, PMEPA1, CTDSPL, TRIB2, GPRC5A, BMP4, TIMP3, F2RL1 | |  |  |  |  |  |  |  |  |  |  |  |  |  |  |  |  |  |  |  |  |  |  |  |
| Gonad Development | | 6 | | 3 | 2.46E-03 | 0.016 | INHBB, WT1, TGFB2, GATA6, TLR3, TNFSF10 | |  |  |  |  |  |  |  |  |  |  |  |  |  |  |  |  |  |  |  |  |  |  |  |
| Hyaluronan Metabolic Process | | 3 | | 7 | 2.54E-03 | 0.014 | CEMIP, VEGFA, HAS2 | |  |  |  |  |  |  |  |  |  |  |  |  |  |  |  |  |  |  |  |  |  |  |  |
| Positive Regulation Of Cardiac Muscle Cell Differentiation | | 3 | | 7 | 2.54E-03 | 0.014 | MEF2C, BMP4, EFNB2 | |  |  |  |  |  |  |  |  |  |  |  |  |  |  |  |  |  |  |  |  |  |  |  |
| Regulation Of Alkaline Phosphatase Activity | | 2 | | 18 | 2.56E-03 | 0.011 | TGFB2, MEF2C | |  |  |  |  |  |  |  |  |  |  |  |  |  |  |  |  |  |  |  |  |  |  |  |
| Corpus Callosum Development | | 2 | | 18 | 2.56E-03 | 0.011 | LPAR1, PTPRS | |  |  |  |  |  |  |  |  |  |  |  |  |  |  |  |  |  |  |  |  |  |  |  |
| Response To Luteinizing Hormone | | 2 | | 18 | 2.56E-03 | 0.011 | TGFBR3, SCP2 | |  |  |  |  |  |  |  |  |  |  |  |  |  |  |  |  |  |  |  |  |  |  |  |
| Kidney Development | | 6 | | 3 | 2.59E-03 | 0.016 | VEGFA, HAS2, WT1, FAT4, BMP4, TGFB2 | |  |  |  |  |  |  |  |  |  |  |  |  |  |  |  |  |  |  |  |  |  |  |  |
| Cellular Response To Stimulus | | 41 | | 1 | 2.65E-03 | 0.010 | PLK2, ATP2B4, ABCA1, TSPYL5, FBXO32, CLU, BMP4, TRIM6, CPT1A, LPAR1, EYA4, FBP1, TNFRSF21, HAS2, HLA-A, GATA6, GBP1, GBP2, GBP3, INHBB, ITGB3, MEF2C, MAP3K1, SNX10, LTBP1, SCN5A, SCP2, SOX4, SNAI2, VEGFA, WT1, THBS1, TIMP3, TLR3, LAPTM5, CPEB4, ADAMTS12, NOG, RAD50, OPTN, NUAK1 | |  |  |  |  |  |  |  |  |  |  |  |  |  |  |  |  |  |  |  |  |  |  |  |
| Response To Extracellular Stimulus | | 13 | | 1 | 2.71E-03 | 0.014 | ABCA1, INHBB, CPEB4, CYP24A1, MEF2C, CTSD, LIPG, LTBP1, OPTN, VEGFA, TGFB2, TIMP3, ACSL1 | |  |  |  |  |  |  |  |  |  |  |  |  |  |  |  |  |  |  |  |  |  |  |  |
| Response To Protozoan | | 3 | | 7 | 2.73E-03 | 0.014 | GBP1, GBP2, GBP3 | |  |  |  |  |  |  |  |  |  |  |  |  |  |  |  |  |  |  |  |  |  |  |  |
| Regulation Of Lipid Transport | | 5 | | 3 | 2.78E-03 | 0.016 | ITGB3, SCP2, ABCA1, THBS1, LIPG | |  |  |  |  |  |  |  |  |  |  |  |  |  |  |  |  |  |  |  |  |  |  |  |
| Regulation Of Peptide Secretion | | 11 | | 1 | 2.81E-03 | 0.015 | SOX4, TNFRSF21, ARRB1, ARL2BP, TRIM6, GBP1, CPT1A, INHBB, TGFB2, CASK, F2RL1 | |  |  |  |  |  |  |  |  |  |  |  |  |  |  |  |  |  |  |  |  |  |  |  |
| Heart Development | | 8 | | 2 | 2.90E-03 | 0.016 | SOX4, MEF2C, HEG1, GATA6, RAD50, WT1, BMP4, TGFB2 | |  |  |  |  |  |  |  |  |  |  |  |  |  |  |  |  |  |  |  |  |  |  |  |
| Regulation Of Protein Serine/Threonine Kinase Activity | | 11 | | 1 | 2.92E-03 | 0.015 | ARRB1, CEMIP, MAP3K1, ATP2B4, VEGFA, TRIB2, LPAR1, BMP4, TGFA, THBS1, ACSL1 | |  |  |  |  |  |  |  |  |  |  |  |  |  |  |  |  |  |  |  |  |  |  |  |
| Regulation Of Pri-Mirna Transcription By Rna Polymerase Ii | | 3 | | 7 | 2.93E-03 | 0.014 | BMP4, TGFB2, GATA6 | |  |  |  |  |  |  |  |  |  |  |  |  |  |  |  |  |  |  |  |  |  |  |  |
| Positive Regulation Of Peptidyl-Lysine Acetylation | | 3 | | 7 | 2.93E-03 | 0.014 | SOX4, ARRB1, SNAI2 | |  |  |  |  |  |  |  |  |  |  |  |  |  |  |  |  |  |  |  |  |  |  |  |
| Regulation Of Stress-Activated Mapk Cascade | | 7 | | 2 | 2.95E-03 | 0.016 | MAP3K1, MIR181B1, VEGFA, TGFB2, TGFBR3, TLR3, F2RL1 | |  |  |  |  |  |  |  |  |  |  |  |  |  |  |  |  |  |  |  |  |  |  |  |
| Positive Regulation Of Cell-Substrate Adhesion | | 5 | | 3 | 2.96E-03 | 0.016 | VEGFA, HAS2, ITGB3, THBS1, CSF1 | |  |  |  |  |  |  |  |  |  |  |  |  |  |  |  |  |  |  |  |  |  |  |  |
| Regulation Of Stress-Activated Protein Kinase Signaling Cascade | | 7 | | 2 | 3.01E-03 | 0.016 | MAP3K1, MIR181B1, VEGFA, TGFB2, TGFBR3, TLR3, F2RL1 | |  |  |  |  |  |  |  |  |  |  |  |  |  |  |  |  |  |  |  |  |  |  |  |
| Regulation Of Cellular Response To Stress | | 13 | | 1 | 3.03E-03 | 0.014 | PLK2, MIR181B1, CLU, PTPRS, MAP3K1, SNAI2, VEGFA, TGFB2, TGFBR3, NUAK1, EYA4, TLR3, F2RL1 | |  |  |  |  |  |  |  |  |  |  |  |  |  |  |  |  |  |  |  |  |  |  |  |
| Response To Hypoxia | | 9 | | 2 | 3.03E-03 | 0.015 | SOX4, PDLIM1, SDC2, GATA6, NOG, VEGFA, TGFB2, THBS1, TGFBR3 | |  |  |  |  |  |  |  |  |  |  |  |  |  |  |  |  |  |  |  |  |  |  |  |
| Transport | | 47 | | 1 | 3.03E-03 | 0.010 | ARRB1, ATP2B4, ABCA1, ADGRE5, MARCH3, CLU, DPYSL2, DSP, CPT1A, CTSD, F2RL1, F13A1, ACSL1, HAS2, PRICKLE1, ITGB3, SNX10, COX7B2, KCNIP3, CLIC6, SLC37A2, SNX7, TMPRSS15, RRBP1, SCN5A, SCP2, SCN9A, CPPED1, RAB27B, SLC44A2, VEGFA, TGFA, TGFB2, THBS1, TGM2, TIMP3, SERINC2, SLC7A5, ILDR2, EVI5, CLIC3, CDC42BPA, CASK, CRISPLD2, APOL6, LIPG, OPTN | |  |  |  |  |  |  |  |  |  |  |  |  |  |  |  |  |  |  |  |  |  |  |  |
| Lymphoid Progenitor Cell Differentiation | | 2 | | 16 | 3.05E-03 | 0.011 | SOX4, BMP4 | |  |  |  |  |  |  |  |  |  |  |  |  |  |  |  |  |  |  |  |  |  |  |  |
| Cardiac Chamber Formation | | 2 | | 16 | 3.05E-03 | 0.011 | SOX4, MEF2C | |  |  |  |  |  |  |  |  |  |  |  |  |  |  |  |  |  |  |  |  |  |  |  |
| Positive Regulation Of Glomerulus Development | | 2 | | 16 | 3.05E-03 | 0.011 | NOG, ITGB3 | |  |  |  |  |  |  |  |  |  |  |  |  |  |  |  |  |  |  |  |  |  |  |  |
| Cranial Suture Morphogenesis | | 2 | | 16 | 3.05E-03 | 0.011 | BMP4, TGFB2 | |  |  |  |  |  |  |  |  |  |  |  |  |  |  |  |  |  |  |  |  |  |  |  |
| Regulation Of Peptide Transport | | 14 | | 1 | 3.09E-03 | 0.014 | TNFRSF21, ARRB1, GBP1, INHBB, BMP4, CASK, SOX4, CEMIP, ARL2BP, TRIM6, CPT1A, LCP1, TGFB2, F2RL1 | |  |  |  |  |  |  |  |  |  |  |  |  |  |  |  |  |  |  |  |  |  |  |  |
| Regulation Of Establishment Of Protein Localization | | 14 | | 1 | 3.09E-03 | 0.014 | TNFRSF21, ARRB1, GBP1, INHBB, BMP4, CASK, SOX4, CEMIP, ARL2BP, TRIM6, CPT1A, LCP1, TGFB2, F2RL1 | |  |  |  |  |  |  |  |  |  |  |  |  |  |  |  |  |  |  |  |  |  |  |  |
| Regulation Of Organelle Organization | | 20 | | 1 | 3.10E-03 | 0.012 | PLK2, ARRB1, BAIAP2L1, CLU, BMP4, SNAI2, VEGFA, LPAR1, TGFA, TGFB2, F2RL1, EVI5, ITGB3, TNFSF10, MEF2C, MAP3K1, LCP1, ASAP1, RAD50, OPTN | |  |  |  |  |  |  |  |  |  |  |  |  |  |  |  |  |  |  |  |  |  |  |  |
| Endothelial Cell Development | | 3 | | 7 | 3.14E-03 | 0.014 | HEG1, PTPRS, F2RL1 | |  |  |  |  |  |  |  |  |  |  |  |  |  |  |  |  |  |  |  |  |  |  |  |
| Negative Regulation Of Toll-Like Receptor Signaling Pathway | | 3 | | 7 | 3.14E-03 | 0.014 | PTPRS, TLR3, F2RL1 | |  |  |  |  |  |  |  |  |  |  |  |  |  |  |  |  |  |  |  |  |  |  |  |
| Positive Regulation Of Cardiac Muscle Cell Proliferation | | 3 | | 7 | 3.14E-03 | 0.014 | MEF2C, GATA6, TGFBR3 | |  |  |  |  |  |  |  |  |  |  |  |  |  |  |  |  |  |  |  |  |  |  |  |
| Regulation Of Body Fluid Levels | | 10 | | 1 | 3.24E-03 | 0.015 | HEG1, HAS2, GATA6, FLG, VEGFA, ITGB3, THBS1, C4BPB, F2RL1, F13A1 | |  |  |  |  |  |  |  |  |  |  |  |  |  |  |  |  |  |  |  |  |  |  |  |
| Cellular Response To Oxygen-Containing Compound | | 19 | | 1 | 3.24E-03 | 0.012 | ATP2B4, ABCA1, GBP2, FBXO32, INHBB, ITGB3, CPEB4, SCP2, BMP4, SOX4, MEF2C, CPT1A, LTBP1, OPTN, VEGFA, WT1, LPAR1, THBS1, FBP1 | |  |  |  |  |  |  |  |  |  |  |  |  |  |  |  |  |  |  |  |  |  |  |  |
| Regulation Of Neuron Apoptotic Process | | 7 | | 2 | 3.27E-03 | 0.016 | ARRB1, MEF2C, KCNIP3, OPTN, VEGFA, CPEB4, TGFB2 | |  |  |  |  |  |  |  |  |  |  |  |  |  |  |  |  |  |  |  |  |  |  |  |
| Cell Migration | | 16 | | 1 | 3.27E-03 | 0.013 | SLC7A5, TNS3, ITGB3, CDC42BPA, ADAMTS12, MEF2C, SDC2, LCP1, SNAI2, VEGFA, LPAR1, EFNB2, TGFB2, THBS1, TGFBR3, F2RL1 | |  |  |  |  |  |  |  |  |  |  |  |  |  |  |  |  |  |  |  |  |  |  |  |
| Face Morphogenesis | | 3 | | 6 | 3.36E-03 | 0.014 | CRISPLD2, TGFB2, NOG | |  |  |  |  |  |  |  |  |  |  |  |  |  |  |  |  |  |  |  |  |  |  |  |
| Positive Regulation Of Cell Development | | 12 | | 1 | 3.48E-03 | 0.014 | HAS2, PLXNA1, TENM3, ITGB3, BMP4, CASK, CDON, MEF2C, NOG, CSF1, VEGFA, LPAR1 | |  |  |  |  |  |  |  |  |  |  |  |  |  |  |  |  |  |  |  |  |  |  |  |
| Muscle Adaptation | | 3 | | 6 | 3.58E-03 | 0.014 | MEF2C, FBXO32, GATA6 | |  |  |  |  |  |  |  |  |  |  |  |  |  |  |  |  |  |  |  |  |  |  |  |
| Regulation Of Vascular Endothelial Growth Factor Receptor Signaling Pathway | | 3 | | 6 | 3.58E-03 | 0.014 | VEGFA, ITGB3, BMP4 | |  |  |  |  |  |  |  |  |  |  |  |  |  |  |  |  |  |  |  |  |  |  |  |
| Pancreas Development | | 3 | | 6 | 3.58E-03 | 0.014 | ILDR2, TGFB2, GATA6 | |  |  |  |  |  |  |  |  |  |  |  |  |  |  |  |  |  |  |  |  |  |  |  |
| Cardiac Muscle Cell Differentiation | | 3 | | 6 | 3.58E-03 | 0.014 | MEF2C, BMP4, GATA6 | |  |  |  |  |  |  |  |  |  |  |  |  |  |  |  |  |  |  |  |  |  |  |  |
| Negative Regulation Of Animal Organ Morphogenesis | | 3 | | 6 | 3.58E-03 | 0.014 | BMP4, TGFB2, NOG | |  |  |  |  |  |  |  |  |  |  |  |  |  |  |  |  |  |  |  |  |  |  |  |
| Regulation Of Heart Morphogenesis | | 3 | | 6 | 3.58E-03 | 0.014 | BMP4, TGFB2, NOG | |  |  |  |  |  |  |  |  |  |  |  |  |  |  |  |  |  |  |  |  |  |  |  |
| Lymph Vessel Development | | 2 | | 15 | 3.59E-03 | 0.011 | EFNB2, HEG1 | |  |  |  |  |  |  |  |  |  |  |  |  |  |  |  |  |  |  |  |  |  |  |  |
| Alpha-Linolenic Acid Metabolic Process | | 2 | | 15 | 3.59E-03 | 0.011 | ACSL1, SCP2 | |  |  |  |  |  |  |  |  |  |  |  |  |  |  |  |  |  |  |  |  |  |  |  |
| Epithelial To Mesenchymal Transition Involved In Endocardial Cushion Formation | | 2 | | 15 | 3.59E-03 | 0.011 | BMP4, SNAI2 | |  |  |  |  |  |  |  |  |  |  |  |  |  |  |  |  |  |  |  |  |  |  |  |
| Pathway-Restricted Smad Protein Phosphorylation | | 2 | | 15 | 3.59E-03 | 0.011 | TGFB2, TGFBR3 | |  |  |  |  |  |  |  |  |  |  |  |  |  |  |  |  |  |  |  |  |  |  |  |
| High-Density Lipoprotein Particle Assembly | | 2 | | 15 | 3.59E-03 | 0.011 | ABCA1, BMP1 | |  |  |  |  |  |  |  |  |  |  |  |  |  |  |  |  |  |  |  |  |  |  |  |
| Secondary Palate Development | | 2 | | 15 | 3.59E-03 | 0.011 | TGFB2, TGFBR3 | |  |  |  |  |  |  |  |  |  |  |  |  |  |  |  |  |  |  |  |  |  |  |  |
| Negative Regulation Of Neuron Differentiation | | 7 | | 2 | 3.60E-03 | 0.016 | PLK2, HLA-A, ASAP1, LPAR1, EFNB2, PTPRS, EFEMP1 | |  |  |  |  |  |  |  |  |  |  |  |  |  |  |  |  |  |  |  |  |  |  |  |
| Regulation Of Gliogenesis | | 5 | | 3 | 3.65E-03 | 0.016 | TNFRSF21, BMP4, NOG, CSF1, EFEMP1 | |  |  |  |  |  |  |  |  |  |  |  |  |  |  |  |  |  |  |  |  |  |  |  |
| Regulation Of Rho Protein Signal Transduction | | 5 | | 3 | 3.65E-03 | 0.016 | TIAM2, ARRB1, LPAR1, ABCA1, F2RL1 | |  |  |  |  |  |  |  |  |  |  |  |  |  |  |  |  |  |  |  |  |  |  |  |
| Cellular Response To Hormone Stimulus | | 10 | | 1 | 3.66E-03 | 0.015 | MEF2C, GATA6, LTBP1, FBXO32, INHBB, VEGFA, ITGB3, WT1, BMP4, FBP1 | |  |  |  |  |  |  |  |  |  |  |  |  |  |  |  |  |  |  |  |  |  |  |  |
| Cell Morphogenesis | | 7 | | 2 | 3.67E-03 | 0.016 | MEF2C, MAP3K1, HEG1, CDH11, ITGB3, CLU, TGFB2 | |  |  |  |  |  |  |  |  |  |  |  |  |  |  |  |  |  |  |  |  |  |  |  |
| Positive Regulation Of Immune System Process | | 19 | | 1 | 3.73E-03 | 0.012 | TNFRSF21, HLA-A, HLA-B, ITGB3, CLU, HLA-DRA, C4BPB, ZMIZ1, MEF2C, MAP3K1, TRIM6, CSF1, PLPP4, VEGFA, EFNB2, TGFB2, THBS1, TLR3, F2RL1 | |  |  |  |  |  |  |  |  |  |  |  |  |  |  |  |  |  |  |  |  |  |  |  |
| Regulation Of Organ Growth | | 5 | | 3 | 3.76E-03 | 0.016 | MEF2C, WT1, GATA6, TGFBR3, NOG | |  |  |  |  |  |  |  |  |  |  |  |  |  |  |  |  |  |  |  |  |  |  |  |
| Regulation Of Growth | | 14 | | 1 | 3.82E-03 | 0.013 | GATA6, TSPYL5, IGFBP4, BMP4, PTPRS, MEF2C, DPYSL2, NOG, CSF1, VEGFA, WT1, TGFB2, TGFBR3, FBP1 | |  |  |  |  |  |  |  |  |  |  |  |  |  |  |  |  |  |  |  |  |  |  |  |
| Positive Regulation Of Proteasomal Ubiquitin-Dependent Protein Catabolic Process | | 4 | | 4 | 3.82E-03 | 0.015 | PLK2, TRIB2, CLU, PRICKLE1 | |  |  |  |  |  |  |  |  |  |  |  |  |  |  |  |  |  |  |  |  |  |  |  |
| Regulation Of Protein Tyrosine Kinase Activity | | 4 | | 4 | 3.82E-03 | 0.015 | AFAP1L2, ITGB3, GPRC5A, TGFA | |  |  |  |  |  |  |  |  |  |  |  |  |  |  |  |  |  |  |  |  |  |  |  |
| Homeostasis Of Number Of Cells Within A Tissue | | 3 | | 6 | 3.82E-03 | 0.014 | ILDR2, VEGFA, CSF1 | |  |  |  |  |  |  |  |  |  |  |  |  |  |  |  |  |  |  |  |  |  |  |  |
| Regulation Of Secretion By Cell | | 14 | | 1 | 3.85E-03 | 0.013 | TNFRSF21, ARRB1, GBP1, INHBB, CASK, RAB27B, SOX4, MEF2C, ARL2BP, DPYSL2, TRIM6, CPT1A, TGFB2, F2RL1 | |  |  |  |  |  |  |  |  |  |  |  |  |  |  |  |  |  |  |  |  |  |  |  |
| Cellular Response To Mechanical Stimulus | | 5 | | 3 | 3.87E-03 | 0.016 | MAP3K1, ITGB3, BMP4, TLR3, LTBP1 | |  |  |  |  |  |  |  |  |  |  |  |  |  |  |  |  |  |  |  |  |  |  |  |
| Fatty Acid Metabolic Process | | 8 | | 2 | 3.88E-03 | 0.015 | PON2, PTGES, CPT1A, ELOVL7, PTGR1, SCP2, ACOXL, ACSL1 | |  |  |  |  |  |  |  |  |  |  |  |  |  |  |  |  |  |  |  |  |  |  |  |
| Positive Regulation Of Apoptotic Signaling Pathway | | 6 | | 2 | 3.90E-03 | 0.016 | INHBB, CLU, TGFB2, THBS1, TIMP3, TNFSF10 | |  |  |  |  |  |  |  |  |  |  |  |  |  |  |  |  |  |  |  |  |  |  |  |
| Roof Of Mouth Development | | 4 | | 4 | 3.97E-03 | 0.015 | MEF2C, TGFB2, TGFBR3, SNAI2 | |  |  |  |  |  |  |  |  |  |  |  |  |  |  |  |  |  |  |  |  |  |  |  |
| Response To Organonitrogen Compound | | 18 | | 1 | 3.98E-03 | 0.012 | ATP2B4, ABCA1, GATA6, INHBB, ITGB3, CPEB4, SCP2, TNFSF10, MEF2C, DPYSL2, SDC2, OPTN, VEGFA, WT1, TGFB2, TGFBR3, TIMP3, FBP1 | |  |  |  |  |  |  |  |  |  |  |  |  |  |  |  |  |  |  |  |  |  |  |  |
| Positive Regulation Of Binding | | 6 | | 2 | 3.99E-03 | 0.016 | PLK2, ARRB1, MEF2C, BMP4, TRIM6, TGFB2 | |  |  |  |  |  |  |  |  |  |  |  |  |  |  |  |  |  |  |  |  |  |  |  |
| Lipid Metabolic Process | | 19 | | 1 | 4.00E-03 | 0.012 | PON2, LPCAT2, ABCA1, PTGR1, CLU, SCP2, ACOXL, CYP24A1, SLC44A2, SDC2, CPT1A, UGCG, ACSL1, SERINC2, GATA6, ELOVL7, PTGES, LIPG, PLPP4 | |  |  |  |  |  |  |  |  |  |  |  |  |  |  |  |  |  |  |  |  |  |  |  |
| Regulation Of Binding | | 9 | | 2 | 4.04E-03 | 0.015 | CDON, PLK2, ARRB1, MEF2C, TRIM6, NOG, BMP4, TGFB2, TGFBR3 | |  |  |  |  |  |  |  |  |  |  |  |  |  |  |  |  |  |  |  |  |  |  |  |
| Regulation Of Hemopoiesis | | 10 | | 1 | 4.07E-03 | 0.014 | ZMIZ1, MEF2C, ZNF608, MYL9, CSF1, VEGFA, ITGB3, BMP4, THBS1, TLR3 | |  |  |  |  |  |  |  |  |  |  |  |  |  |  |  |  |  |  |  |  |  |  |  |
| Regulation Of Smooth Muscle Cell Proliferation | | 6 | | 2 | 4.08E-03 | 0.016 | MEF2C, VEGFA, ITGB3, BMP4, THBS1, TGM2 | |  |  |  |  |  |  |  |  |  |  |  |  |  |  |  |  |  |  |  |  |  |  |  |
| Lung Development | | 5 | | 3 | 4.09E-03 | 0.016 | VEGFA, CRISPLD2, HEG1, BMP4, TGFB2 | |  |  |  |  |  |  |  |  |  |  |  |  |  |  |  |  |  |  |  |  |  |  |  |
| Regulation Of Angiogenesis | | 9 | | 2 | 4.15E-03 | 0.015 | PLK2, MIR181B1, GATA6, VEGFA, ITGB3, HHIP, TGFB2, THBS1, TLR3 | |  |  |  |  |  |  |  |  |  |  |  |  |  |  |  |  |  |  |  |  |  |  |  |
| Glomerular Visceral Epithelial Cell Development | | 2 | | 14 | 4.17E-03 | 0.011 | BMP4, WT1 | |  |  |  |  |  |  |  |  |  |  |  |  |  |  |  |  |  |  |  |  |  |  |  |
| Mesonephros Development | | 2 | | 14 | 4.17E-03 | 0.011 | BMP4, WT1 | |  |  |  |  |  |  |  |  |  |  |  |  |  |  |  |  |  |  |  |  |  |  |  |
| Negative Regulation Of Pathway-Restricted Smad Protein Phosphorylation | | 2 | | 14 | 4.17E-03 | 0.011 | NOG, PMEPA1 | |  |  |  |  |  |  |  |  |  |  |  |  |  |  |  |  |  |  |  |  |  |  |  |
| Nucleoside Monophosphate Phosphorylation | | 2 | | 14 | 4.17E-03 | 0.011 | RAD50, AK4 | |  |  |  |  |  |  |  |  |  |  |  |  |  |  |  |  |  |  |  |  |  |  |  |
| Negative Regulation Of Macrophage Derived Foam Cell Differentiation | | 2 | | 14 | 4.17E-03 | 0.011 | ABCA1, ITGB3 | |  |  |  |  |  |  |  |  |  |  |  |  |  |  |  |  |  |  |  |  |  |  |  |
| Regulation Of Lipoprotein Metabolic Process | | 2 | | 14 | 4.17E-03 | 0.011 | ITGB3, LIPG | |  |  |  |  |  |  |  |  |  |  |  |  |  |  |  |  |  |  |  |  |  |  |  |
| Embryonic Skeletal Joint Morphogenesis | | 2 | | 14 | 4.17E-03 | 0.011 | BMP4, NOG | |  |  |  |  |  |  |  |  |  |  |  |  |  |  |  |  |  |  |  |  |  |  |  |
| Coronary Vasculature Morphogenesis | | 2 | | 14 | 4.17E-03 | 0.011 | TGFBR3, VEGFA | |  |  |  |  |  |  |  |  |  |  |  |  |  |  |  |  |  |  |  |  |  |  |  |
| Localization | | 52 | | 0 | 4.17E-03 | 0.010 | ARRB1, ATP2B4, ABCA1, ADGRE5, MARCH3, CLU, BMP4, ARL2BP, DPYSL2, DSP, CPT1A, CTSD, F2RL1, F13A1, ACSL1, HAS2, GBP1, GBP2, PRICKLE1, ITGB3, SNX10, COX7B2, KCNIP3, LTBP1, CLIC6, SLC37A2, SNX7, TMPRSS15, RRBP1, SCN5A, SCP2, SCN9A, CPPED1, RAB27B, SLC44A2, VEGFA, TGFA, TGFB2, THBS1, TGM2, TIMP3, SERINC2, SLC7A5, ILDR2, EVI5, CLIC3, CDC42BPA, CASK, CRISPLD2, APOL6, LIPG, OPTN | |  |  |  |  |  |  |  |  |  |  |  |  |  |  |  |  |  |  |  |  |  |  |  |
| Regulation Of Leukocyte Migration | | 6 | | 2 | 4.26E-03 | 0.015 | VEGFA, ITGB3, MTUS1, THBS1, CSF1, F2RL1 | |  |  |  |  |  |  |  |  |  |  |  |  |  |  |  |  |  |  |  |  |  |  |  |
| Regulation Of Actin Filament-Based Process | | 9 | | 2 | 4.28E-03 | 0.015 | MEF2C, MAP3K1, DSP, ITGB3, BAIAP2L1, SCN5A, LPAR1, TGFB2, F2RL1 | |  |  |  |  |  |  |  |  |  |  |  |  |  |  |  |  |  |  |  |  |  |  |  |
| Post-Translational Protein Modification | | 8 | | 2 | 4.29E-03 | 0.015 | SDC2, CSF1, LTBP1, MGAT4A, IGFBP4, FBXO32, BMP4, PRSS23 | |  |  |  |  |  |  |  |  |  |  |  |  |  |  |  |  |  |  |  |  |  |  |  |
| Positive Regulation Of Cytokine Production Involved In Immune Response | | 3 | | 6 | 4.32E-03 | 0.014 | HLA-A, TRIM6, F2RL1 | |  |  |  |  |  |  |  |  |  |  |  |  |  |  |  |  |  |  |  |  |  |  |  |
| Regulation Of Cell Adhesion Mediated By Integrin | | 3 | | 6 | 4.32E-03 | 0.014 | ITGB3, TGFB2, SNAI2 | |  |  |  |  |  |  |  |  |  |  |  |  |  |  |  |  |  |  |  |  |  |  |  |
| Positive Regulation Of Leukocyte Migration | | 5 | | 3 | 4.33E-03 | 0.015 | VEGFA, ITGB3, THBS1, CSF1, F2RL1 | |  |  |  |  |  |  |  |  |  |  |  |  |  |  |  |  |  |  |  |  |  |  |  |
| Cellular Lipid Metabolic Process | | 16 | | 1 | 4.43E-03 | 0.013 | PON2, LPCAT2, GATA6, ELOVL7, PTGR1, SCP2, ACOXL, SLC44A2, PTGES, SDC2, CPT1A, LIPG, PLPP4, UGCG, ACSL1, SERINC2 | |  |  |  |  |  |  |  |  |  |  |  |  |  |  |  |  |  |  |  |  |  |  |  |
| Positive Regulation Of Supramolecular Fiber Organization | | 6 | | 2 | 4.46E-03 | 0.015 | MAP3K1, BAIAP2L1, CLU, LPAR1, COLGALT1, F2RL1 | |  |  |  |  |  |  |  |  |  |  |  |  |  |  |  |  |  |  |  |  |  |  |  |
| Multicellular Organismal Process | | 56 | | 0 | 4.52E-03 | 0.009 | PLK2, ARRB1, ABCA1, MYL9, CDH11, FBXO32, BMP4, BMP1, C4BPB, TDRD7, DPYSL2, DSP, OR5P2, CSF1, CTSD, FLG, EFNB2, EYA4, F2RL1, F13A1, EFEMP1, HAS2, HLA-A, GATA6, INHBB, ITGB3, HLA-DRA, CDON, MEF2C, SNX10, KCNIP3, MPZL3, ADGRF1, PLXNA1, SCN5A, SCN9A, SOX4, ZMIZ1, CEMIP, HEG1, SNAI2, VEGFA, WT1, NXN, HHIP, TGFB2, TGFBR3, TGM2, TIMP3, TLL1, SLC7A5, SHISA2, EVI5, FAT4, NOG, LIPG | |  |  |  |  |  |  |  |  |  |  |  |  |  |  |  |  |  |  |  |  |  |  |  |
| Response To Stimulus | | 78 | | 0 | 4.54E-03 | 0.009 | ARRB1, ATP2B4, ABCA1, TSPYL5, AK4, ADGRE5, FBXO32, CLU, BMP4, C4BPB, CYP24A1, DPYSL2, DSP, TRIM6, CPT1A, OR5P2, CSF1, CST4, CTSD, LPAR1, EYA4, F2RL1, F13A1, ACSL1, FBP1, HAS2, HLA-A, HLA-B, GATA6, GBP1, GBP2, GBP3, IGFBP4, INHBB, ITGB3, HLA-DRA, MEF2C, MAP3K1, LCP1, LTBP1, PON2, SCN5A, SCP2, SCN9A, SOX4, SDC2, SNAI2, VEGFA, WT1, TGFA, TGFB2, THBS1, TGFBR3, TIMP3, TLR3, ILDR2, LAPTM5, CST7, TNFSF10, PTGES, FCGR2C, PDLIM1, NOG, LIPG, TSPAN2, RAD50, OPTN, NUAK1, PLK2, PTGR1, TNFRSF21, SNX10, KCNIP3, BAIAP2L1, SLAMF7, CPEB4, ADAMTS12, AFAP1L2 | |  |  |  |  |  |  |  |  |  |  |  |  |  |  |  |  |  |  |  |  |  |  |  |
| Regulation Of Insulin Secretion | | 6 | | 2 | 4.56E-03 | 0.015 | SOX4, ARRB1, INHBB, ARL2BP, CPT1A, CASK | |  |  |  |  |  |  |  |  |  |  |  |  |  |  |  |  |  |  |  |  |  |  |  |
| Regulation Of Protein Ubiquitination | | 6 | | 2 | 4.56E-03 | 0.015 | SOX4, ARRB1, TRIB2, NXN, TSPYL5, PRICKLE1 | |  |  |  |  |  |  |  |  |  |  |  |  |  |  |  |  |  |  |  |  |  |  |  |
| Negative Regulation Of Vasculature Development | | 6 | | 2 | 4.56E-03 | 0.015 | PLK2, WT1, HHIP, BMP4, TGFB2, THBS1 | |  |  |  |  |  |  |  |  |  |  |  |  |  |  |  |  |  |  |  |  |  |  |  |
| Positive Regulation Of Muscle Cell Apoptotic Process | | 3 | | 6 | 4.59E-03 | 0.014 | ARRB1, FBXO32, GATA6 | |  |  |  |  |  |  |  |  |  |  |  |  |  |  |  |  |  |  |  |  |  |  |  |
| Positive Regulation Of Leukocyte Chemotaxis | | 4 | | 4 | 4.62E-03 | 0.015 | VEGFA, THBS1, CSF1, F2RL1 | |  |  |  |  |  |  |  |  |  |  |  |  |  |  |  |  |  |  |  |  |  |  |  |
| Cerebral Cortex Development | | 4 | | 4 | 4.62E-03 | 0.015 | CDON, FAT4, PTPRS, CASK | |  |  |  |  |  |  |  |  |  |  |  |  |  |  |  |  |  |  |  |  |  |  |  |
| Positive Regulation Of Podosome Assembly | | 2 | | 13 | 4.79E-03 | 0.011 | LCP1, ASAP1 | |  |  |  |  |  |  |  |  |  |  |  |  |  |  |  |  |  |  |  |  |  |  |  |
| Glomerular Epithelial Cell Development | | 2 | | 13 | 4.79E-03 | 0.011 | BMP4, WT1 | |  |  |  |  |  |  |  |  |  |  |  |  |  |  |  |  |  |  |  |  |  |  |  |
| Specification Of Animal Organ Identity | | 2 | | 13 | 4.79E-03 | 0.011 | BMP4, MEF2C | |  |  |  |  |  |  |  |  |  |  |  |  |  |  |  |  |  |  |  |  |  |  |  |
| Pulmonary Valve Morphogenesis | | 2 | | 13 | 4.79E-03 | 0.011 | BMP4, TGFB2 | |  |  |  |  |  |  |  |  |  |  |  |  |  |  |  |  |  |  |  |  |  |  |  |
| Endocardial Cushion Development | | 2 | | 13 | 4.79E-03 | 0.011 | BMP4, THBS1 | |  |  |  |  |  |  |  |  |  |  |  |  |  |  |  |  |  |  |  |  |  |  |  |
| Fatty Acid Beta-Oxidation Using Acyl-Coa Oxidase | | 2 | | 13 | 4.79E-03 | 0.011 | ACOXL, SCP2 | |  |  |  |  |  |  |  |  |  |  |  |  |  |  |  |  |  |  |  |  |  |  |  |
| Glial Cell Proliferation | | 2 | | 13 | 4.79E-03 | 0.011 | SOX4, CLU | |  |  |  |  |  |  |  |  |  |  |  |  |  |  |  |  |  |  |  |  |  |  |  |
| Response To Muscle Inactivity Involved In Regulation Of Muscle Adaptation | | 2 | | 13 | 4.79E-03 | 0.011 | FBXO32, SCN5A | |  |  |  |  |  |  |  |  |  |  |  |  |  |  |  |  |  |  |  |  |  |  |  |
| Response To Denervation Involved In Regulation Of Muscle Adaptation | | 2 | | 13 | 4.79E-03 | 0.011 | FBXO32, SCN5A | |  |  |  |  |  |  |  |  |  |  |  |  |  |  |  |  |  |  |  |  |  |  |  |
| Regulation Of Plasma Membrane Organization | | 2 | | 13 | 4.79E-03 | 0.011 | TGFB2, ASAP1 | |  |  |  |  |  |  |  |  |  |  |  |  |  |  |  |  |  |  |  |  |  |  |  |
| Regulation Of Osteoblast Differentiation | | 5 | | 3 | 4.83E-03 | 0.015 | MEF2C, VEGFA, BMP4, NOG, SNAI2 | |  |  |  |  |  |  |  |  |  |  |  |  |  |  |  |  |  |  |  |  |  |  |  |
| Regulation Of Production Of Molecular Mediator Of Immune Response | | 5 | | 3 | 4.96E-03 | 0.015 | HLA-A, TRIM6, TGFB2, TLR3, F2RL1 | |  |  |  |  |  |  |  |  |  |  |  |  |  |  |  |  |  |  |  |  |  |  |  |
| Cellular Response To Organic Cyclic Compound | | 11 | | 1 | 5.06E-03 | 0.014 | ATP2B4, ABCA1, FBXO32, INHBB, SCP2, BMP4, MEF2C, LTBP1, VEGFA, WT1, TLR3 | |  |  |  |  |  |  |  |  |  |  |  |  |  |  |  |  |  |  |  |  |  |  |  |
| Positive Regulation Of Transport | | 17 | | 1 | 5.13E-03 | 0.012 | ARRB1, ABCA1, INHBB, CLU, SCN5A, SCP2, BMP4, CASK, RAB27B, SOX4, CEMIP, DPYSL2, TRIM6, LIPG, VEGFA, TGFB2, F2RL1 | |  |  |  |  |  |  |  |  |  |  |  |  |  |  |  |  |  |  |  |  |  |  |  |
| Ureteric Bud Development | | 3 | | 5 | 5.15E-03 | 0.013 | WT1, BMP4, NOG | |  |  |  |  |  |  |  |  |  |  |  |  |  |  |  |  |  |  |  |  |  |  |  |
| Positive Regulation Of Map Kinase Activity | | 7 | | 2 | 5.42E-03 | 0.015 | ARRB1, MAP3K1, VEGFA, LPAR1, BMP4, TGFA, THBS1 | |  |  |  |  |  |  |  |  |  |  |  |  |  |  |  |  |  |  |  |  |  |  |  |
| Establishment Of Localization | | 47 | | 0 | 5.43E-03 | 0.010 | ARRB1, ATP2B4, ABCA1, ADGRE5, MARCH3, CLU, DPYSL2, DSP, CPT1A, CTSD, F2RL1, F13A1, ACSL1, HAS2, PRICKLE1, ITGB3, SNX10, COX7B2, KCNIP3, CLIC6, SLC37A2, SNX7, TMPRSS15, RRBP1, SCN5A, SCP2, SCN9A, CPPED1, RAB27B, SLC44A2, VEGFA, TGFA, TGFB2, THBS1, TGM2, TIMP3, SERINC2, SLC7A5, ILDR2, EVI5, CLIC3, CDC42BPA, CASK, CRISPLD2, APOL6, LIPG, OPTN | |  |  |  |  |  |  |  |  |  |  |  |  |  |  |  |  |  |  |  |  |  |  |  |
| Positive Regulation Of Pathway-Restricted Smad Protein Phosphorylation | | 3 | | 5 | 5.44E-03 | 0.013 | INHBB, BMP4, TGFB2 | |  |  |  |  |  |  |  |  |  |  |  |  |  |  |  |  |  |  |  |  |  |  |  |
| Positive Regulation Of Protein Acetylation | | 3 | | 5 | 5.44E-03 | 0.013 | SOX4, ARRB1, SNAI2 | |  |  |  |  |  |  |  |  |  |  |  |  |  |  |  |  |  |  |  |  |  |  |  |
| Negative Regulation Of Pri-Mirna Transcription By Rna Polymerase Ii | | 2 | | 12 | 5.45E-03 | 0.010 | BMP4, GATA6 | |  |  |  |  |  |  |  |  |  |  |  |  |  |  |  |  |  |  |  |  |  |  |  |
| Regulation Of Transcription From Rna Polymerase Ii Promoter Involved In Heart Development | | 2 | | 12 | 5.45E-03 | 0.010 | BMP4, NOG | |  |  |  |  |  |  |  |  |  |  |  |  |  |  |  |  |  |  |  |  |  |  |  |
| Regulation Of Glial Cell Migration | | 2 | | 12 | 5.45E-03 | 0.010 | CSF1, EFEMP1 | |  |  |  |  |  |  |  |  |  |  |  |  |  |  |  |  |  |  |  |  |  |  |  |
| Regulation Of Apoptotic Process Involved In Morphogenesis | | 2 | | 12 | 5.45E-03 | 0.010 | TGFB2, TGFBR3 | |  |  |  |  |  |  |  |  |  |  |  |  |  |  |  |  |  |  |  |  |  |  |  |
| Cellular Response To Parathyroid Hormone Stimulus | | 2 | | 12 | 5.45E-03 | 0.010 | MEF2C, LTBP1 | |  |  |  |  |  |  |  |  |  |  |  |  |  |  |  |  |  |  |  |  |  |  |  |
| Neural Tube Closure | | 4 | | 3 | 5.53E-03 | 0.015 | BMP4, TGFB2, NOG, PRICKLE1 | |  |  |  |  |  |  |  |  |  |  |  |  |  |  |  |  |  |  |  |  |  |  |  |
| Glycosaminoglycan Biosynthetic Process | | 4 | | 3 | 5.53E-03 | 0.015 | CEMIP, HAS2, SDC2, CHST15 | |  |  |  |  |  |  |  |  |  |  |  |  |  |  |  |  |  |  |  |  |  |  |  |
| Regulation Of Cardiac Muscle Tissue Growth | | 4 | | 3 | 5.53E-03 | 0.015 | MEF2C, GATA6, TGFBR3, NOG | |  |  |  |  |  |  |  |  |  |  |  |  |  |  |  |  |  |  |  |  |  |  |  |
| Cell Activation | | 16 | | 1 | 5.71E-03 | 0.012 | ARRB1, ADGRE5, ITGB3, CLU, CPPED1, SOX4, SLC44A2, MEF2C, CRISPLD2, DSP, CSF1, LCP1, CTSD, SLAMF7, TLR3, F2RL1 | |  |  |  |  |  |  |  |  |  |  |  |  |  |  |  |  |  |  |  |  |  |  |  |
| Plasma Lipoprotein Particle Organization | | 3 | | 5 | 5.74E-03 | 0.013 | ABCA1, BMP1, LIPG | |  |  |  |  |  |  |  |  |  |  |  |  |  |  |  |  |  |  |  |  |  |  |  |
| Branching Involved In Ureteric Bud Morphogenesis | | 3 | | 5 | 5.74E-03 | 0.013 | WT1, FAT4, BMP4 | |  |  |  |  |  |  |  |  |  |  |  |  |  |  |  |  |  |  |  |  |  |  |  |
| Response To Vitamin D | | 3 | | 5 | 5.74E-03 | 0.013 | CYP24A1, TGFB2, LTBP1 | |  |  |  |  |  |  |  |  |  |  |  |  |  |  |  |  |  |  |  |  |  |  |  |
| Response To Interferon-Gamma | | 5 | | 3 | 5.80E-03 | 0.015 | HLA-A, GBP1, GBP2, GBP3, TLR3 | |  |  |  |  |  |  |  |  |  |  |  |  |  |  |  |  |  |  |  |  |  |  |  |
| Inflammatory Response | | 10 | | 1 | 5.81E-03 | 0.014 | PTGES, CSF1, IGFBP4, TSPAN2, ADGRE5, AFAP1L2, SCN9A, THBS1, TLR3, F2RL1 | |  |  |  |  |  |  |  |  |  |  |  |  |  |  |  |  |  |  |  |  |  |  |  |
| Negative Regulation Of Biological Process | | 63 | | 0 | 5.82E-03 | 0.009 | ARRB1, ATP2B4, ABCA1, FBXO32, CLU, BMP4, C4BPB, TRIM6, CST4, LPAR1, EFNB2, EYA4, F2RL1, EFEMP1, FBP1, HLA-A, GATA6, GBP1, PRICKLE1, IGFBP4, INHBB, ITGB3, SH3RF2, MEF2C, MAP3K1, LTBP1, PLXNA1, SCP2, PTPRS, SOX4, SNAI2, VEGFA, WT1, TGFA, TGFB2, THBS1, TGFBR3, TIMP3, TLR3, SHISA2, GPRC5A, CST7, CASK, TNFSF10, PTGES, NOG, RAD50, OPTN, CTDSPL, PLK2, PDE10A, MIR181B1, TNFRSF21, TRIB2, MIR604, KCNIP3, ASAP1, ZNF608, PMEPA1, NXN, HHIP, CPEB4, ADAMTS12 | |  |  |  |  |  |  |  |  |  |  |  |  |  |  |  |  |  |  |  |  |  |  |  |
| Tube Closure | | 4 | | 3 | 5.92E-03 | 0.014 | BMP4, TGFB2, NOG, PRICKLE1 | |  |  |  |  |  |  |  |  |  |  |  |  |  |  |  |  |  |  |  |  |  |  |  |
| Positive Regulation Of Interleukin-8 Production | | 3 | | 5 | 6.06E-03 | 0.013 | AFAP1L2, TLR3, F2RL1 | |  |  |  |  |  |  |  |  |  |  |  |  |  |  |  |  |  |  |  |  |  |  |  |
| Positive Regulation Of Cytosolic Calcium Ion Concentration Involved In Phospholipase C-Activating G-Protein Coupled Signaling Pathway | | 3 | | 5 | 6.06E-03 | 0.013 | LPAR1, TGM2, F2RL1 | |  |  |  |  |  |  |  |  |  |  |  |  |  |  |  |  |  |  |  |  |  |  |  |
| Regulation Of Endothelial Cell Differentiation | | 3 | | 5 | 6.06E-03 | 0.013 | VEGFA, MIR181B1, BMP4 | |  |  |  |  |  |  |  |  |  |  |  |  |  |  |  |  |  |  |  |  |  |  |  |
| Lipid Transport | | 7 | | 2 | 6.13E-03 | 0.015 | ABCA1, APOL6, CPT1A, LIPG, CLU, SCP2, ACSL1 | |  |  |  |  |  |  |  |  |  |  |  |  |  |  |  |  |  |  |  |  |  |  |  |
| Regulation Of Bmp Signaling Pathway | | 4 | | 3 | 6.13E-03 | 0.014 | BMP4, GATA6, TGFBR3, NOG | |  |  |  |  |  |  |  |  |  |  |  |  |  |  |  |  |  |  |  |  |  |  |  |
| Regulation Of Erk1 And Erk2 Cascade | | 8 | | 2 | 6.14E-03 | 0.014 | ARRB1, GBP1, VEGFA, ITGB3, BMP4, TGFBR3, TIMP3, F2RL1 | |  |  |  |  |  |  |  |  |  |  |  |  |  |  |  |  |  |  |  |  |  |  |  |
| Cell Migration Involved In Sprouting Angiogenesis | | 2 | | 11 | 6.15E-03 | 0.010 | EFNB2, VEGFA | |  |  |  |  |  |  |  |  |  |  |  |  |  |  |  |  |  |  |  |  |  |  |  |
| Ventricular Trabecula Myocardium Morphogenesis | | 2 | | 11 | 6.15E-03 | 0.010 | TGFB2, HEG1 | |  |  |  |  |  |  |  |  |  |  |  |  |  |  |  |  |  |  |  |  |  |  |  |
| Response To Muscle Inactivity | | 2 | | 11 | 6.15E-03 | 0.010 | FBXO32, SCN5A | |  |  |  |  |  |  |  |  |  |  |  |  |  |  |  |  |  |  |  |  |  |  |  |
| Mesodermal Cell Differentiation | | 2 | | 11 | 6.15E-03 | 0.010 | BMP4, ITGB3 | |  |  |  |  |  |  |  |  |  |  |  |  |  |  |  |  |  |  |  |  |  |  |  |
| Mesenchymal To Epithelial Transition | | 2 | | 11 | 6.15E-03 | 0.010 | BMP4, WT1 | |  |  |  |  |  |  |  |  |  |  |  |  |  |  |  |  |  |  |  |  |  |  |  |
| Regulation Of Cation Transmembrane Transport | | 8 | | 2 | 6.23E-03 | 0.014 | MEF2C, CEMIP, ATP2B4, KCNIP3, ITGB3, SCN5A, BMP4, TGFB2 | |  |  |  |  |  |  |  |  |  |  |  |  |  |  |  |  |  |  |  |  |  |  |  |
| Lung Alveolus Development | | 3 | | 5 | 6.38E-03 | 0.013 | VEGFA, BMP4, TNS3 | |  |  |  |  |  |  |  |  |  |  |  |  |  |  |  |  |  |  |  |  |  |  |  |
| Positive Regulation Of Protein Catabolic Process | | 6 | | 2 | 6.50E-03 | 0.015 | PLK2, MIR181B1, PRICKLE1, TRIB2, CLU, C4BPB | |  |  |  |  |  |  |  |  |  |  |  |  |  |  |  |  |  |  |  |  |  |  |  |
| Positive Regulation Of Ubiquitin-Dependent Protein Catabolic Process | | 4 | | 3 | 6.55E-03 | 0.014 | PLK2, TRIB2, CLU, PRICKLE1 | |  |  |  |  |  |  |  |  |  |  |  |  |  |  |  |  |  |  |  |  |  |  |  |
| Movement Of Cell Or Subcellular Component | | 21 | | 1 | 6.71E-03 | 0.011 | ATP2B4, PLXNA1, DPYSL2, SDC2, SNAI2, VEGFA, WT1, LPAR1, EFNB2, TGFB2, THBS1, TGFBR3, F2RL1, SLC7A5, TNS3, ITGB3, CDC42BPA, ADAMTS12, MEF2C, NOG, LCP1 | |  |  |  |  |  |  |  |  |  |  |  |  |  |  |  |  |  |  |  |  |  |  |  |
| Regulation Of Cytokine Secretion Involved In Immune Response | | 2 | | 11 | 6.88E-03 | 0.010 | TRIM6, F2RL1 | |  |  |  |  |  |  |  |  |  |  |  |  |  |  |  |  |  |  |  |  |  |  |  |
| Chronic Inflammatory Response | | 2 | | 11 | 6.88E-03 | 0.010 | PTGES, THBS1 | |  |  |  |  |  |  |  |  |  |  |  |  |  |  |  |  |  |  |  |  |  |  |  |
| Positive Regulation Of Macrophage Chemotaxis | | 2 | | 11 | 6.88E-03 | 0.010 | THBS1, CSF1 | |  |  |  |  |  |  |  |  |  |  |  |  |  |  |  |  |  |  |  |  |  |  |  |
| Cardiac Muscle Cell Proliferation | | 2 | | 11 | 6.88E-03 | 0.010 | TGFB2, TGFBR3 | |  |  |  |  |  |  |  |  |  |  |  |  |  |  |  |  |  |  |  |  |  |  |  |
| Atrial Septum Morphogenesis | | 2 | | 11 | 6.88E-03 | 0.010 | SOX4, TGFB2 | |  |  |  |  |  |  |  |  |  |  |  |  |  |  |  |  |  |  |  |  |  |  |  |
| Aminoglycan Biosynthetic Process | | 4 | | 3 | 6.99E-03 | 0.014 | CEMIP, HAS2, SDC2, CHST15 | |  |  |  |  |  |  |  |  |  |  |  |  |  |  |  |  |  |  |  |  |  |  |  |
| Positive Regulation Of Developmental Growth | | 6 | | 2 | 7.04E-03 | 0.015 | MEF2C, GATA6, CSF1, VEGFA, WT1, TGFBR3 | |  |  |  |  |  |  |  |  |  |  |  |  |  |  |  |  |  |  |  |  |  |  |  |
| Protein-Lipid Complex Subunit Organization | | 3 | | 5 | 7.06E-03 | 0.013 | ABCA1, BMP1, LIPG | |  |  |  |  |  |  |  |  |  |  |  |  |  |  |  |  |  |  |  |  |  |  |  |
| Cell Fate Determination | | 3 | | 5 | 7.06E-03 | 0.013 | MEF2C, BMP4, GATA6 | |  |  |  |  |  |  |  |  |  |  |  |  |  |  |  |  |  |  |  |  |  |  |  |
| Regulation Of Substrate Adhesion-Dependent Cell Spreading | | 3 | | 5 | 7.06E-03 | 0.013 | HAS2, ITGB3, GBP1 | |  |  |  |  |  |  |  |  |  |  |  |  |  |  |  |  |  |  |  |  |  |  |  |
| Regulation Of Extrinsic Apoptotic Signaling Pathway In Absence Of Ligand | | 3 | | 5 | 7.06E-03 | 0.013 | TGFB2, EYA4, SNAI2 | |  |  |  |  |  |  |  |  |  |  |  |  |  |  |  |  |  |  |  |  |  |  |  |
| Regulation Of Lipid Localization | | 5 | | 2 | 7.08E-03 | 0.015 | ITGB3, SCP2, ABCA1, THBS1, LIPG | |  |  |  |  |  |  |  |  |  |  |  |  |  |  |  |  |  |  |  |  |  |  |  |
| Positive Regulation Of Cytokine Production | | 9 | | 1 | 7.09E-03 | 0.014 | HEG1, HLA-A, TRIM6, AFAP1L2, OPTN, CLU, THBS1, TLR3, F2RL1 | |  |  |  |  |  |  |  |  |  |  |  |  |  |  |  |  |  |  |  |  |  |  |  |
| Regulation Of Map Kinase Activity | | 8 | | 2 | 7.11E-03 | 0.014 | ARRB1, MAP3K1, VEGFA, TRIB2, LPAR1, BMP4, TGFA, THBS1 | |  |  |  |  |  |  |  |  |  |  |  |  |  |  |  |  |  |  |  |  |  |  |  |
| Angiogenesis | | 7 | | 2 | 7.13E-03 | 0.014 | VEGFA, ITGB3, BMP4, EFNB2, TGFA, TGFB2, THBS1 | |  |  |  |  |  |  |  |  |  |  |  |  |  |  |  |  |  |  |  |  |  |  |  |
| Cartilage Development | | 4 | | 3 | 7.22E-03 | 0.014 | BMP4, BMP1, THBS1, NOG | |  |  |  |  |  |  |  |  |  |  |  |  |  |  |  |  |  |  |  |  |  |  |  |
| Epithelial Cell Development | | 5 | | 2 | 7.25E-03 | 0.015 | HEG1, WT1, BMP4, PTPRS, F2RL1 | |  |  |  |  |  |  |  |  |  |  |  |  |  |  |  |  |  |  |  |  |  |  |  |
| Positive Regulation Of Nervous System Development | | 11 | | 1 | 7.31E-03 | 0.013 | PLXNA1, TENM3, ADGRE5, BMP4, CASK, CDON, MEF2C, NOG, CSF1, VEGFA, LPAR1 | |  |  |  |  |  |  |  |  |  |  |  |  |  |  |  |  |  |  |  |  |  |  |  |
| Activation Of Mapk Activity | | 5 | | 2 | 7.42E-03 | 0.014 | ARRB1, MAP3K1, LPAR1, TGFA, THBS1 | |  |  |  |  |  |  |  |  |  |  |  |  |  |  |  |  |  |  |  |  |  |  |  |
| Positive Regulation Of Protein Binding | | 4 | | 3 | 7.45E-03 | 0.014 | PLK2, ARRB1, MEF2C, BMP4 | |  |  |  |  |  |  |  |  |  |  |  |  |  |  |  |  |  |  |  |  |  |  |  |
| Regulation Of Cell Morphogenesis | | 10 | | 1 | 7.55E-03 | 0.013 | DPYSL2, HAS2, SDC2, GBP1, PLXNA1, VEGFA, ITGB3, LPAR1, PTPRS, CASK | |  |  |  |  |  |  |  |  |  |  |  |  |  |  |  |  |  |  |  |  |  |  |  |
| Response To External Stimulus | | 28 | | 1 | 7.63E-03 | 0.011 | ARRB1, ABCA1, BAIAP2L1, CLU, BMP4, CYP24A1, TRIM6, CTSD, VEGFA, TGFB2, THBS1, TIMP3, TLR3, F2RL1, ACSL1, HLA-A, GBP1, GBP2, GBP3, INHBB, ITGB3, CPEB4, PTGES, MEF2C, MAP3K1, LIPG, LTBP1, OPTN | |  |  |  |  |  |  |  |  |  |  |  |  |  |  |  |  |  |  |  |  |  |  |  |
| Regulation Of Protein Catabolic Process | | 8 | | 1 | 7.63E-03 | 0.014 | PLK2, SAMD9L, MIR181B1, PRICKLE1, TRIB2, CLU, C4BPB, TIMP3 | |  |  |  |  |  |  |  |  |  |  |  |  |  |  |  |  |  |  |  |  |  |  |  |
| Regulation Of Podosome Assembly | | 2 | | 10 | 7.66E-03 | 0.010 | LCP1, ASAP1 | |  |  |  |  |  |  |  |  |  |  |  |  |  |  |  |  |  |  |  |  |  |  |  |
| Mesenchymal Cell Proliferation | | 2 | | 10 | 7.66E-03 | 0.010 | FAT4, BMP4 | |  |  |  |  |  |  |  |  |  |  |  |  |  |  |  |  |  |  |  |  |  |  |  |
| Positive Regulation Of Endothelial Cell Chemotaxis | | 2 | | 10 | 7.66E-03 | 0.010 | VEGFA, SNAI2 | |  |  |  |  |  |  |  |  |  |  |  |  |  |  |  |  |  |  |  |  |  |  |  |
| Negative Regulation Of Kidney Development | | 2 | | 10 | 7.66E-03 | 0.010 | BMP4, WT1 | |  |  |  |  |  |  |  |  |  |  |  |  |  |  |  |  |  |  |  |  |  |  |  |
| Positive Regulation Of Creb Transcription Factor Activity | | 2 | | 10 | 7.66E-03 | 0.010 | VEGFA, ADGRF1 | |  |  |  |  |  |  |  |  |  |  |  |  |  |  |  |  |  |  |  |  |  |  |  |
| Long-Chain Fatty-Acyl-Coa Biosynthetic Process | | 2 | | 10 | 7.66E-03 | 0.010 | ELOVL7, ACSL1 | |  |  |  |  |  |  |  |  |  |  |  |  |  |  |  |  |  |  |  |  |  |  |  |
| Mammary Gland Alveolus Development | | 2 | | 10 | 7.66E-03 | 0.010 | TGFA, VEGFA | |  |  |  |  |  |  |  |  |  |  |  |  |  |  |  |  |  |  |  |  |  |  |  |
| Regulation Of Neural Precursor Cell Proliferation | | 4 | | 3 | 7.69E-03 | 0.014 | CDON, OPTN, VEGFA, NOG | |  |  |  |  |  |  |  |  |  |  |  |  |  |  |  |  |  |  |  |  |  |  |  |
| Mesonephric Tubule Development | | 3 | | 5 | 7.78E-03 | 0.013 | WT1, BMP4, NOG | |  |  |  |  |  |  |  |  |  |  |  |  |  |  |  |  |  |  |  |  |  |  |  |
| Cytokine Secretion | | 3 | | 5 | 7.78E-03 | 0.013 | OPTN, ABCA1, F2RL1 | |  |  |  |  |  |  |  |  |  |  |  |  |  |  |  |  |  |  |  |  |  |  |  |
| Regulation Of Sterol Transport | | 3 | | 5 | 7.78E-03 | 0.013 | SCP2, ABCA1, LIPG | |  |  |  |  |  |  |  |  |  |  |  |  |  |  |  |  |  |  |  |  |  |  |  |
| Regulation Of Cholesterol Transport | | 3 | | 5 | 7.78E-03 | 0.013 | SCP2, ABCA1, LIPG | |  |  |  |  |  |  |  |  |  |  |  |  |  |  |  |  |  |  |  |  |  |  |  |
| Cell Junction Assembly | | 5 | | 2 | 7.78E-03 | 0.014 | PARD6B, CDH11, HEG1, ITGB3, LAMA3 | |  |  |  |  |  |  |  |  |  |  |  |  |  |  |  |  |  |  |  |  |  |  |  |
| Cellular Response To Drug | | 9 | | 1 | 7.85E-03 | 0.014 | MEF2C, OPTN, FBXO32, VEGFA, ITGB3, BMP4, THBS1, TLR3, FBP1 | |  |  |  |  |  |  |  |  |  |  |  |  |  |  |  |  |  |  |  |  |  |  |  |
| Cytokine-Mediated Signaling Pathway | | 11 | | 1 | 8.04E-03 | 0.013 | TNFRSF21, HLA-A, HLA-B, GBP1, GBP2, HLA-DRA, CSF1, LCP1, VEGFA, F13A1, ACSL1 | |  |  |  |  |  |  |  |  |  |  |  |  |  |  |  |  |  |  |  |  |  |  |  |
| Reproductive Structure Development | | 8 | | 1 | 8.08E-03 | 0.014 | GATA6, INHBB, VEGFA, WT1, BMP4, TGFB2, TLR3, TNFSF10 | |  |  |  |  |  |  |  |  |  |  |  |  |  |  |  |  |  |  |  |  |  |  |  |
| Homeostatic Process | | 22 | | 1 | 8.10E-03 | 0.011 | HECTD4, ATP2B4, ABCA1, SCN5A, BMP4, ACOXL, SOX4, CEMIP, CSF1, CST4, FLG, VEGFA, NXN, LPAR1, TGM2, F2RL1, ILDR2, ITGB3, MEF2C, SNX10, LIPG, RAD50 | |  |  |  |  |  |  |  |  |  |  |  |  |  |  |  |  |  |  |  |  |  |  |  |
| Positive Regulation Of Secretion By Cell | | 9 | | 1 | 8.15E-03 | 0.014 | SOX4, ARRB1, DPYSL2, TRIM6, INHBB, TGFB2, CASK, RAB27B, F2RL1 | |  |  |  |  |  |  |  |  |  |  |  |  |  |  |  |  |  |  |  |  |  |  |  |
| Mesonephric Epithelium Development | | 3 | | 5 | 8.15E-03 | 0.013 | WT1, BMP4, NOG | |  |  |  |  |  |  |  |  |  |  |  |  |  |  |  |  |  |  |  |  |  |  |  |
| Positive Regulation Of Cell-Matrix Adhesion | | 3 | | 5 | 8.15E-03 | 0.013 | VEGFA, ITGB3, CSF1 | |  |  |  |  |  |  |  |  |  |  |  |  |  |  |  |  |  |  |  |  |  |  |  |
| Positive Regulation Of Ossification | | 4 | | 3 | 8.18E-03 | 0.014 | MEF2C, VEGFA, BMP4, TGFB2 | |  |  |  |  |  |  |  |  |  |  |  |  |  |  |  |  |  |  |  |  |  |  |  |
| Response To Retinoic Acid | | 5 | | 2 | 8.34E-03 | 0.014 | PTGES, MEF2C, ABCA1, BMP4, TGFB2 | |  |  |  |  |  |  |  |  |  |  |  |  |  |  |  |  |  |  |  |  |  |  |  |
| Positive Regulation Of Proteasomal Protein Catabolic Process | | 4 | | 3 | 8.43E-03 | 0.014 | PLK2, TRIB2, CLU, PRICKLE1 | |  |  |  |  |  |  |  |  |  |  |  |  |  |  |  |  |  |  |  |  |  |  |  |
| Seminiferous Tubule Development | | 2 | | 10 | 8.47E-03 | 0.010 | INHBB, WT1 | |  |  |  |  |  |  |  |  |  |  |  |  |  |  |  |  |  |  |  |  |  |  |  |
| Negative Regulation Of Interleukin-10 Production | | 2 | | 10 | 8.47E-03 | 0.010 | TNFRSF21, TRIB2 | |  |  |  |  |  |  |  |  |  |  |  |  |  |  |  |  |  |  |  |  |  |  |  |
| Positive Regulation Of Macrophage Migration | | 2 | | 10 | 8.47E-03 | 0.010 | THBS1, CSF1 | |  |  |  |  |  |  |  |  |  |  |  |  |  |  |  |  |  |  |  |  |  |  |  |
| Cardiac Muscle Hypertrophy In Response To Stress | | 2 | | 10 | 8.47E-03 | 0.010 | GATA6, MEF2C | |  |  |  |  |  |  |  |  |  |  |  |  |  |  |  |  |  |  |  |  |  |  |  |
| Muscle Hypertrophy In Response To Stress | | 2 | | 10 | 8.47E-03 | 0.010 | GATA6, MEF2C | |  |  |  |  |  |  |  |  |  |  |  |  |  |  |  |  |  |  |  |  |  |  |  |
| Apoptotic Cell Clearance | | 2 | | 10 | 8.47E-03 | 0.010 | TGM2, ITGB3 | |  |  |  |  |  |  |  |  |  |  |  |  |  |  |  |  |  |  |  |  |  |  |  |
| Negative Regulation Of Lipid Storage | | 2 | | 10 | 8.47E-03 | 0.010 | ABCA1, ITGB3 | |  |  |  |  |  |  |  |  |  |  |  |  |  |  |  |  |  |  |  |  |  |  |  |
| Striated Muscle Cell Proliferation | | 2 | | 10 | 8.47E-03 | 0.010 | TGFB2, TGFBR3 | |  |  |  |  |  |  |  |  |  |  |  |  |  |  |  |  |  |  |  |  |  |  |  |
| Cardiac Muscle Adaptation | | 2 | | 10 | 8.47E-03 | 0.010 | GATA6, MEF2C | |  |  |  |  |  |  |  |  |  |  |  |  |  |  |  |  |  |  |  |  |  |  |  |
| Response To Laminar Fluid Shear Stress | | 2 | | 10 | 8.47E-03 | 0.010 | ABCA1, TGFB2 | |  |  |  |  |  |  |  |  |  |  |  |  |  |  |  |  |  |  |  |  |  |  |  |
| Regulation Of Odontogenesis Of Dentin-Containing Tooth | | 2 | | 10 | 8.47E-03 | 0.010 | BMP4, CSF1 | |  |  |  |  |  |  |  |  |  |  |  |  |  |  |  |  |  |  |  |  |  |  |  |
| Regulation Of Protein Modification By Small Protein Conjugation Or Removal | | 6 | | 2 | 8.51E-03 | 0.014 | SOX4, ARRB1, TSPYL5, PRICKLE1, TRIB2, NXN | |  |  |  |  |  |  |  |  |  |  |  |  |  |  |  |  |  |  |  |  |  |  |  |
| Fatty Acid Derivative Metabolic Process | | 5 | | 2 | 8.53E-03 | 0.014 | PON2, ELOVL7, PTGES, PTGR1, ACSL1 | |  |  |  |  |  |  |  |  |  |  |  |  |  |  |  |  |  |  |  |  |  |  |  |
| Negative Regulation Of Lipid Localization | | 3 | | 5 | 8.54E-03 | 0.013 | ITGB3, ABCA1, THBS1 | |  |  |  |  |  |  |  |  |  |  |  |  |  |  |  |  |  |  |  |  |  |  |  |
| Positive Regulation Of Cardiac Muscle Tissue Growth | | 3 | | 5 | 8.54E-03 | 0.013 | MEF2C, GATA6, TGFBR3 | |  |  |  |  |  |  |  |  |  |  |  |  |  |  |  |  |  |  |  |  |  |  |  |
| Vasculature Development | | 3 | | 4 | 8.94E-03 | 0.013 | VEGFA, HEG1, BMP4 | |  |  |  |  |  |  |  |  |  |  |  |  |  |  |  |  |  |  |  |  |  |  |  |
| Myeloid Leukocyte Activation | | 10 | | 1 | 9.15E-03 | 0.013 | SLC44A2, CRISPLD2, DSP, CSF1, CTSD, ADGRE5, CLU, CPPED1, TLR3, F2RL1 | |  |  |  |  |  |  |  |  |  |  |  |  |  |  |  |  |  |  |  |  |  |  |  |
| Response To Mechanical Stimulus | | 7 | | 2 | 9.20E-03 | 0.014 | MAP3K1, LTBP1, ITGB3, BMP4, THBS1, TIMP3, TLR3 | |  |  |  |  |  |  |  |  |  |  |  |  |  |  |  |  |  |  |  |  |  |  |  |
| Unsaturated Fatty Acid Metabolic Process | | 4 | | 3 | 9.21E-03 | 0.014 | PTGES, PTGR1, SCP2, ACSL1 | |  |  |  |  |  |  |  |  |  |  |  |  |  |  |  |  |  |  |  |  |  |  |  |
| Transforming Growth Factor Beta Receptor Signaling Pathway | | 4 | | 3 | 9.21E-03 | 0.014 | MAP3K1, TGFB2, TGFBR3, LTBP1 | |  |  |  |  |  |  |  |  |  |  |  |  |  |  |  |  |  |  |  |  |  |  |  |
| Regulation Of Secretion | | 14 | | 1 | 9.30E-03 | 0.012 | TNFRSF21, ARRB1, GBP1, INHBB, CASK, RAB27B, SOX4, MEF2C, ARL2BP, DPYSL2, TRIM6, CPT1A, TGFB2, F2RL1 | |  |  |  |  |  |  |  |  |  |  |  |  |  |  |  |  |  |  |  |  |  |  |  |
| Endocardial Cushion Morphogenesis | | 2 | | 9 | 9.32E-03 | 0.010 | TGFB2, NOG | |  |  |  |  |  |  |  |  |  |  |  |  |  |  |  |  |  |  |  |  |  |  |  |
| Response To Folic Acid | | 2 | | 9 | 9.32E-03 | 0.010 | TIMP3, VEGFA | |  |  |  |  |  |  |  |  |  |  |  |  |  |  |  |  |  |  |  |  |  |  |  |
| Positive Regulation Of Fibroblast Migration | | 2 | | 9 | 9.32E-03 | 0.010 | THBS1, ITGB3 | |  |  |  |  |  |  |  |  |  |  |  |  |  |  |  |  |  |  |  |  |  |  |  |
| Positive Regulation Of Cell Adhesion Mediated By Integrin | | 2 | | 9 | 9.32E-03 | 0.010 | TGFB2, ITGB3 | |  |  |  |  |  |  |  |  |  |  |  |  |  |  |  |  |  |  |  |  |  |  |  |
| Positive Regulation Of Extrinsic Apoptotic Signaling Pathway Via Death Domain Receptors | | 2 | | 9 | 9.32E-03 | 0.010 | THBS1, TIMP3 | |  |  |  |  |  |  |  |  |  |  |  |  |  |  |  |  |  |  |  |  |  |  |  |
| Negative Regulation Of Neuron Apoptotic Process | | 5 | | 2 | 9.33E-03 | 0.014 | OPTN, ARRB1, MEF2C, VEGFA, CPEB4 | |  |  |  |  |  |  |  |  |  |  |  |  |  |  |  |  |  |  |  |  |  |  |  |
| Cardiac Septum Development | | 3 | | 4 | 9.34E-03 | 0.013 | HEG1, BMP4, LTBP1 | |  |  |  |  |  |  |  |  |  |  |  |  |  |  |  |  |  |  |  |  |  |  |  |
| Response To Lipid | | 18 | | 1 | 9.43E-03 | 0.012 | ABCA1, FBXO32, SCP2, BMP4, CYP24A1, TRIM6, CPT1A, VEGFA, LPAR1, TGFB2, THBS1, TGFBR3, ACSL1, GBP2, INHBB, PTGES, MEF2C, LTBP1 | |  |  |  |  |  |  |  |  |  |  |  |  |  |  |  |  |  |  |  |  |  |  |  |
| Fatty Acid Beta-Oxidation | | 3 | | 4 | 9.76E-03 | 0.013 | SCP2, ACOXL, CPT1A | |  |  |  |  |  |  |  |  |  |  |  |  |  |  |  |  |  |  |  |  |  |  |  |
| Extracellular Matrix Disassembly | | 3 | | 4 | 9.76E-03 | 0.013 | BMP1, TLL1, LCP1 | |  |  |  |  |  |  |  |  |  |  |  |  |  |  |  |  |  |  |  |  |  |  |  |
| Regulation Of Camp-Mediated Signaling | | 3 | | 4 | 9.76E-03 | 0.013 | ATP2B4, PDE10A, LPAR1 | |  |  |  |  |  |  |  |  |  |  |  |  |  |  |  |  |  |  |  |  |  |  |  |
| Regulation Of Cardiac Muscle Cell Differentiation | | 3 | | 4 | 9.76E-03 | 0.013 | MEF2C, BMP4, EFNB2 | |  |  |  |  |  |  |  |  |  |  |  |  |  |  |  |  |  |  |  |  |  |  |  |
| Positive Regulation Of Receptor-Mediated Endocytosis | | 3 | | 4 | 9.76E-03 | 0.013 | ARRB1, VEGFA, CLU | |  |  |  |  |  |  |  |  |  |  |  |  |  |  |  |  |  |  |  |  |  |  |  |
| Regulation Of Cytokine Biosynthetic Process | | 4 | | 3 | 9.76E-03 | 0.014 | INHBB, TRIB2, THBS1, TLR3 | |  |  |  |  |  |  |  |  |  |  |  |  |  |  |  |  |  |  |  |  |  |  |  |
| Leukocyte Activation | | 14 | | 1 | 9.78E-03 | 0.012 | ADGRE5, CLU, CPPED1, SOX4, SLC44A2, MEF2C, CRISPLD2, DSP, CSF1, LCP1, CTSD, SLAMF7, TLR3, F2RL1 | |  |  |  |  |  |  |  |  |  |  |  |  |  |  |  |  |  |  |  |  |  |  |  |
| Positive Regulation Of Neurogenesis | | 10 | | 1 | 9.88E-03 | 0.013 | CDON, MEF2C, NOG, CSF1, PLXNA1, TENM3, VEGFA, LPAR1, BMP4, CASK | |  |  |  |  |  |  |  |  |  |  |  |  |  |  |  |  |  |  |  |  |  |  |  |
| Chemical Homeostasis | | 17 | | 1 | 9.90E-03 | 0.012 | HECTD4, ATP2B4, ABCA1, ITGB3, SCN5A, BMP4, ACOXL, SOX4, MEF2C, CEMIP, SNX10, LIPG, FLG, VEGFA, LPAR1, TGM2, F2RL1 | |  |  |  |  |  |  |  |  |  |  |  |  |  |  |  |  |  |  |  |  |  |  |  |
| Regulation Of Wnt Signaling Pathway | | 7 | | 2 | 9.93E-03 | 0.014 | SOX4, SHISA2, NOG, PRICKLE1, SNAI2, IGFBP4, NXN | |  |  |  |  |  |  |  |  |  |  |  |  |  |  |  |  |  |  |  |  |  |  |  |
| Leukocyte Proliferation | | 4 | | 3 | 1.00E-02 | 0.014 | MEF2C, CLU, CSF1, F2RL1 | |  |  |  |  |  |  |  |  |  |  |  |  |  |  |  |  |  |  |  |  |  |  |  |
| Tissue Homeostasis | | 5 | | 2 | 1.02E-02 | 0.014 | ILDR2, VEGFA, SNX10, CSF1, CST4 | |  |  |  |  |  |  |  |  |  |  |  |  |  |  |  |  |  |  |  |  |  |  |  |
| Ovarian Follicle Development | | 3 | | 4 | 1.02E-02 | 0.013 | INHBB, VEGFA, BMP4 | |  |  |  |  |  |  |  |  |  |  |  |  |  |  |  |  |  |  |  |  |  |  |  |
| Intracellular Lipid Transport | | 2 | | 9 | 1.02E-02 | 0.010 | ABCA1, CPT1A | |  |  |  |  |  |  |  |  |  |  |  |  |  |  |  |  |  |  |  |  |  |  |  |
| Craniofacial Suture Morphogenesis | | 2 | | 9 | 1.02E-02 | 0.010 | BMP4, TGFB2 | |  |  |  |  |  |  |  |  |  |  |  |  |  |  |  |  |  |  |  |  |  |  |  |
| Response To Parathyroid Hormone | | 2 | | 9 | 1.02E-02 | 0.010 | MEF2C, LTBP1 | |  |  |  |  |  |  |  |  |  |  |  |  |  |  |  |  |  |  |  |  |  |  |  |
| Response To Stimulus Involved In Regulation Of Muscle Adaptation | | 2 | | 9 | 1.02E-02 | 0.010 | FBXO32, SCN5A | |  |  |  |  |  |  |  |  |  |  |  |  |  |  |  |  |  |  |  |  |  |  |  |
| Organophosphate Metabolic Process | | 15 | | 1 | 1.03E-02 | 0.012 | LPCAT2, PDE10A, GATA6, AK4, ELOVL7, SCP2, CASK, SLC44A2, LIPG, PLPP4, RAD50, SLC37A2, ACSL1, SERINC2, FBP1 | |  |  |  |  |  |  |  |  |  |  |  |  |  |  |  |  |  |  |  |  |  |  |  |
| Negative Regulation Of Cell-Substrate Adhesion | | 3 | | 4 | 1.06E-02 | 0.013 | GBP1, THBS1, CASK | |  |  |  |  |  |  |  |  |  |  |  |  |  |  |  |  |  |  |  |  |  |  |  |
| Neuron Projection Guidance | | 6 | | 2 | 1.09E-02 | 0.014 | DPYSL2, NOG, PLXNA1, VEGFA, EFNB2, TGFB2 | |  |  |  |  |  |  |  |  |  |  |  |  |  |  |  |  |  |  |  |  |  |  |  |
| Positive Regulation Of Neuron Death | | 4 | | 3 | 1.09E-02 | 0.014 | OPTN, CLU, EFNB2, TGFB2 | |  |  |  |  |  |  |  |  |  |  |  |  |  |  |  |  |  |  |  |  |  |  |  |
| Atrioventricular Valve Morphogenesis | | 2 | | 8 | 1.11E-02 | 0.010 | SOX4, TGFB2 | |  |  |  |  |  |  |  |  |  |  |  |  |  |  |  |  |  |  |  |  |  |  |  |
| Response To Inactivity | | 2 | | 8 | 1.11E-02 | 0.010 | FBXO32, SCN5A | |  |  |  |  |  |  |  |  |  |  |  |  |  |  |  |  |  |  |  |  |  |  |  |
| Positive Regulation Of Extracellular Matrix Organization | | 2 | | 8 | 1.11E-02 | 0.010 | TGFB2, COLGALT1 | |  |  |  |  |  |  |  |  |  |  |  |  |  |  |  |  |  |  |  |  |  |  |  |
| Muscle Cell Differentiation | | 4 | | 3 | 1.12E-02 | 0.013 | CDON, MEF2C, BMP4, GATA6 | |  |  |  |  |  |  |  |  |  |  |  |  |  |  |  |  |  |  |  |  |  |  |  |
| Regulation Of Peptide Hormone Secretion | | 6 | | 2 | 1.13E-02 | 0.014 | SOX4, ARRB1, ARL2BP, CPT1A, INHBB, CASK | |  |  |  |  |  |  |  |  |  |  |  |  |  |  |  |  |  |  |  |  |  |  |  |
| Regulation Of Epithelial Cell Differentiation | | 5 | | 2 | 1.13E-02 | 0.014 | VEGFA, MIR181B1, FAT4, BMP4, TGFB2 | |  |  |  |  |  |  |  |  |  |  |  |  |  |  |  |  |  |  |  |  |  |  |  |
| Negative Regulation Of Macromolecule Metabolic Process | | 38 | | 1 | 1.14E-02 | 0.010 | ARRB1, ATP2B4, MIR181B1, CLU, BMP4, C4BPB, TRIM6, CST4, F2RL1, FBP1, GATA6, GBP1, PRICKLE1, INHBB, TRIB2, ITGB3, SH3RF2, MIR604, MEF2C, KCNIP3, SOX4, ZNF608, PMEPA1, SNAI2, VEGFA, WT1, NXN, TGFB2, THBS1, TGFBR3, TIMP3, CPEB4, GPRC5A, CST7, NOG, RAD50, OPTN, CTDSPL | |  |  |  |  |  |  |  |  |  |  |  |  |  |  |  |  |  |  |  |  |  |  |  |
| Homotypic Cell-Cell Adhesion | | 3 | | 4 | 1.15E-02 | 0.012 | ITGB3, DSP, MYL9 | |  |  |  |  |  |  |  |  |  |  |  |  |  |  |  |  |  |  |  |  |  |  |  |
| Anion Transmembrane Transport | | 6 | | 2 | 1.17E-02 | 0.014 | SLC7A5, ABCA1, CPT1A, CLIC6, CLIC3, SLC37A2 | |  |  |  |  |  |  |  |  |  |  |  |  |  |  |  |  |  |  |  |  |  |  |  |
| Response To Calcium Ion | | 5 | | 2 | 1.18E-02 | 0.014 | PTGES, MEF2C, INHBB, SCN5A, THBS1 | |  |  |  |  |  |  |  |  |  |  |  |  |  |  |  |  |  |  |  |  |  |  |  |
| Cell Fate Commitment | | 5 | | 2 | 1.18E-02 | 0.014 | MEF2C, WT1, BMP4, TGFB2, GATA6 | |  |  |  |  |  |  |  |  |  |  |  |  |  |  |  |  |  |  |  |  |  |  |  |
| Regulation Of Transforming Growth Factor Beta Receptor Signaling Pathway | | 4 | | 3 | 1.19E-02 | 0.013 | THBS1, TGFBR3, PMEPA1, LTBP1 | |  |  |  |  |  |  |  |  |  |  |  |  |  |  |  |  |  |  |  |  |  |  |  |
| Embryo Development Ending In Birth Or Egg Hatching | | 7 | | 1 | 1.20E-02 | 0.013 | ZMIZ1, HEG1, GATA6, NOG, VEGFA, TGFB2, TGFBR3 | |  |  |  |  |  |  |  |  |  |  |  |  |  |  |  |  |  |  |  |  |  |  |  |
| Regulation Of Leukocyte Cell-Cell Adhesion | | 7 | | 1 | 1.20E-02 | 0.013 | TNFRSF21, ZMIZ1, HAS2, ZNF608, HLA-A, BMP4, EFNB2 | |  |  |  |  |  |  |  |  |  |  |  |  |  |  |  |  |  |  |  |  |  |  |  |
| Microglial Cell Activation | | 2 | | 8 | 1.21E-02 | 0.010 | TLR3, CLU | |  |  |  |  |  |  |  |  |  |  |  |  |  |  |  |  |  |  |  |  |  |  |  |
| Leukocyte Activation Involved In Inflammatory Response | | 2 | | 8 | 1.21E-02 | 0.010 | TLR3, CLU | |  |  |  |  |  |  |  |  |  |  |  |  |  |  |  |  |  |  |  |  |  |  |  |
| Cell Surface Receptor Signaling Pathway Involved In Heart Development | | 2 | | 8 | 1.21E-02 | 0.010 | BMP4, NOG | |  |  |  |  |  |  |  |  |  |  |  |  |  |  |  |  |  |  |  |  |  |  |  |
| Long-Chain Fatty Acid Metabolic Process | | 4 | | 3 | 1.22E-02 | 0.013 | PTGES, SCP2, CPT1A, ACSL1 | |  |  |  |  |  |  |  |  |  |  |  |  |  |  |  |  |  |  |  |  |  |  |  |
| Positive Regulation Of Protein Ubiquitination | | 4 | | 3 | 1.22E-02 | 0.013 | ARRB1, TRIB2, TSPYL5, PRICKLE1 | |  |  |  |  |  |  |  |  |  |  |  |  |  |  |  |  |  |  |  |  |  |  |  |
| Developmental Growth | | 8 | | 1 | 1.22E-02 | 0.013 | ZMIZ1, HEG1, HLA-A, CSF1, PLXNA1, SORBS2, BMP4, TIMP3 | |  |  |  |  |  |  |  |  |  |  |  |  |  |  |  |  |  |  |  |  |  |  |  |
| Regulation Of Nitrogen Compound Metabolic Process | | 64 | | 0 | 1.22E-02 | 0.009 | ARRB1, ATP2B4, TSPYL5, CLU, BMP4, C4BPB, TRIM6, CSF1, CST4, LPAR1, EYA4, F2RL1, ACSL1, EFEMP1, FBP1, HAS2, GATA6, GBP1, PRICKLE1, IGFBP4, INHBB, ITGB3, SH3RF2, MEF2C, MEIS3P1, MAP3K1, SAMD9L, ADGRF1, SOX4, SNAI2, VEGFA, WT1, TGFA, TGFB2, THBS1, TGFBR3, TIMP3, TLR3, ZNF391, ZNF43, GPRC5A, CST7, CASK, TNFSF10, PDLIM1, NOG, LIPG, RAD50, MBNL2, CTDSPL, NUAK1, PLK2, MIR181B1, ARL2BP, TRIB2, CDON, KCNIP3, ZMIZ1, CEMIP, ZNF608, PMEPA1, NXN, CPEB4, AFAP1L2 | |  |  |  |  |  |  |  |  |  |  |  |  |  |  |  |  |  |  |  |  |  |  |  |
| Protein Phosphorylation | | 14 | | 1 | 1.23E-02 | 0.012 | PLK2, CDK15, TRIB2, BMP4, CDC42BPA, CASK, MEF2C, MAP3K1, EFNB2, TGFB2, TGFBR3, NUAK1, TLR3, EFEMP1 | |  |  |  |  |  |  |  |  |  |  |  |  |  |  |  |  |  |  |  |  |  |  |  |
| Positive Regulation Of Growth | | 7 | | 1 | 1.24E-02 | 0.013 | MEF2C, GATA6, CSF1, VEGFA, WT1, TGFB2, TGFBR3 | |  |  |  |  |  |  |  |  |  |  |  |  |  |  |  |  |  |  |  |  |  |  |  |
| Erythrocyte Differentiation | | 3 | | 4 | 1.25E-02 | 0.012 | VEGFA, BMP4, TGFBR3 | |  |  |  |  |  |  |  |  |  |  |  |  |  |  |  |  |  |  |  |  |  |  |  |
| Regulation Of Cellular Response To Transforming Growth Factor Beta Stimulus | | 4 | | 3 | 1.25E-02 | 0.013 | THBS1, TGFBR3, PMEPA1, LTBP1 | |  |  |  |  |  |  |  |  |  |  |  |  |  |  |  |  |  |  |  |  |  |  |  |
| Positive Regulation Of Reactive Oxygen Species Metabolic Process | | 4 | | 3 | 1.25E-02 | 0.013 | CLU, MIR181B1, THBS1, F2RL1 | |  |  |  |  |  |  |  |  |  |  |  |  |  |  |  |  |  |  |  |  |  |  |  |
| Positive Regulation Of Cell Morphogenesis Involved In Differentiation | | 5 | | 2 | 1.25E-02 | 0.014 | VEGFA, HAS2, ITGB3, CASK, PLXNA1 | |  |  |  |  |  |  |  |  |  |  |  |  |  |  |  |  |  |  |  |  |  |  |  |
| Embryo Development | | 7 | | 1 | 1.25E-02 | 0.013 | ZMIZ1, HEG1, GATA6, NOG, VEGFA, TGFB2, TGFBR3 | |  |  |  |  |  |  |  |  |  |  |  |  |  |  |  |  |  |  |  |  |  |  |  |
| Transmembrane Receptor Protein Tyrosine Kinase Signaling Pathway | | 9 | | 1 | 1.26E-02 | 0.013 | SDC2, NOG, CSF1, VEGFA, ITGB3, FAT4, EFNB2, TGFA, EFEMP1 | |  |  |  |  |  |  |  |  |  |  |  |  |  |  |  |  |  |  |  |  |  |  |  |
| Positive Regulation Of Peptidyl-Serine Phosphorylation | | 4 | | 3 | 1.28E-02 | 0.013 | ARRB1, ATP2B4, VEGFA, TRIM6 | |  |  |  |  |  |  |  |  |  |  |  |  |  |  |  |  |  |  |  |  |  |  |  |
| Glial Cell Development | | 3 | | 4 | 1.30E-02 | 0.012 | SOX4, TSPAN2, LPAR1 | |  |  |  |  |  |  |  |  |  |  |  |  |  |  |  |  |  |  |  |  |  |  |  |
| Type I Interferon Signaling Pathway | | 3 | | 4 | 1.30E-02 | 0.012 | HLA-A, HLA-B, GBP2 | |  |  |  |  |  |  |  |  |  |  |  |  |  |  |  |  |  |  |  |  |  |  |  |
| Cartilage Condensation | | 2 | | 7 | 1.31E-02 | 0.010 | BMP1, TGFB2 | |  |  |  |  |  |  |  |  |  |  |  |  |  |  |  |  |  |  |  |  |  |  |  |
| Positive Regulation Of Endothelial Cell Differentiation | | 2 | | 7 | 1.31E-02 | 0.010 | MIR181B1, BMP4 | |  |  |  |  |  |  |  |  |  |  |  |  |  |  |  |  |  |  |  |  |  |  |  |
| Negative Regulation Of Stem Cell Differentiation | | 2 | | 7 | 1.31E-02 | 0.010 | TRIM6, PRICKLE1 | |  |  |  |  |  |  |  |  |  |  |  |  |  |  |  |  |  |  |  |  |  |  |  |
| Regulation Of Leukocyte Proliferation | | 6 | | 2 | 1.34E-02 | 0.013 | TNFRSF21, MEF2C, HLA-A, MIR181B1, CSF1, BMP4 | |  |  |  |  |  |  |  |  |  |  |  |  |  |  |  |  |  |  |  |  |  |  |  |
| Mesoderm Development | | 3 | | 4 | 1.35E-02 | 0.012 | VEGFA, BMP4, NOG | |  |  |  |  |  |  |  |  |  |  |  |  |  |  |  |  |  |  |  |  |  |  |  |
| Regulation Of Extrinsic Apoptotic Signaling Pathway Via Death Domain Receptors | | 3 | | 4 | 1.35E-02 | 0.012 | THBS1, TIMP3, TNFSF10 | |  |  |  |  |  |  |  |  |  |  |  |  |  |  |  |  |  |  |  |  |  |  |  |
| Regulation Of Proteasomal Ubiquitin-Dependent Protein Catabolic Process | | 4 | | 3 | 1.35E-02 | 0.013 | PLK2, TRIB2, CLU, PRICKLE1 | |  |  |  |  |  |  |  |  |  |  |  |  |  |  |  |  |  |  |  |  |  |  |  |
| Positive Regulation Of Proteolysis Involved In Cellular Protein Catabolic Process | | 4 | | 3 | 1.35E-02 | 0.013 | PLK2, TRIB2, CLU, PRICKLE1 | |  |  |  |  |  |  |  |  |  |  |  |  |  |  |  |  |  |  |  |  |  |  |  |
| Positive Regulation Of Catabolic Process | | 8 | | 1 | 1.36E-02 | 0.013 | PLK2, MIR181B1, CPT1A, PRICKLE1, OPTN, TRIB2, CLU, C4BPB | |  |  |  |  |  |  |  |  |  |  |  |  |  |  |  |  |  |  |  |  |  |  |  |
| Calcium Ion Homeostasis | | 9 | | 1 | 1.37E-02 | 0.013 | CEMIP, ATP2B4, SNX10, ITGB3, SCN5A, LPAR1, BMP4, TGM2, F2RL1 | |  |  |  |  |  |  |  |  |  |  |  |  |  |  |  |  |  |  |  |  |  |  |  |
| Regulation Of Calcium Ion Transport Into Cytosol | | 4 | | 3 | 1.39E-02 | 0.013 | CEMIP, ITGB3, BMP4, TGFB2 | |  |  |  |  |  |  |  |  |  |  |  |  |  |  |  |  |  |  |  |  |  |  |  |
| Anterior/Posterior Pattern Specification Involved In Kidney Development | | 1 | | 50 | 1.39E-02 | 0.006 | BMP4 | |  |  |  |  |  |  |  |  |  |  |  |  |  |  |  |  |  |  |  |  |  |  |  |
| Regulation Of Metanephric Glomerular Mesangial Cell Proliferation | | 1 | | 50 | 1.39E-02 | 0.006 | WT1 | |  |  |  |  |  |  |  |  |  |  |  |  |  |  |  |  |  |  |  |  |  |  |  |
| Nephron Tubule Epithelial Cell Differentiation | | 1 | | 50 | 1.39E-02 | 0.006 | MEF2C | |  |  |  |  |  |  |  |  |  |  |  |  |  |  |  |  |  |  |  |  |  |  |  |
| Posterior Mesonephric Tubule Development | | 1 | | 50 | 1.39E-02 | 0.006 | WT1 | |  |  |  |  |  |  |  |  |  |  |  |  |  |  |  |  |  |  |  |  |  |  |  |
| Regulation Of Arginine Catabolic Process | | 1 | | 50 | 1.39E-02 | 0.006 | ATP2B4 | |  |  |  |  |  |  |  |  |  |  |  |  |  |  |  |  |  |  |  |  |  |  |  |
| Positive Regulation Of Metanephric Ureteric Bud Development | | 1 | | 50 | 1.39E-02 | 0.006 | WT1 | |  |  |  |  |  |  |  |  |  |  |  |  |  |  |  |  |  |  |  |  |  |  |  |
| Regulation Of Metanephric Ureteric Bud Development | | 1 | | 50 | 1.39E-02 | 0.006 | WT1 | |  |  |  |  |  |  |  |  |  |  |  |  |  |  |  |  |  |  |  |  |  |  |  |
| Endodermal Cell Fate Determination | | 1 | | 50 | 1.39E-02 | 0.006 | GATA6 | |  |  |  |  |  |  |  |  |  |  |  |  |  |  |  |  |  |  |  |  |  |  |  |
| Antigen Processing And Presentation Of Endogenous Peptide Antigen Via Mhc Class I Via Er Pathway, Tap-Dependent | | 1 | | 50 | 1.39E-02 | 0.006 | HLA-A | |  |  |  |  |  |  |  |  |  |  |  |  |  |  |  |  |  |  |  |  |  |  |  |
| Antigen Processing And Presentation Of Endogenous Peptide Antigen Via Mhc Class I Via Er Pathway | | 1 | | 50 | 1.39E-02 | 0.006 | HLA-A | |  |  |  |  |  |  |  |  |  |  |  |  |  |  |  |  |  |  |  |  |  |  |  |
| Positive Regulation Of Eosinophil Degranulation | | 1 | | 50 | 1.39E-02 | 0.006 | F2RL1 | |  |  |  |  |  |  |  |  |  |  |  |  |  |  |  |  |  |  |  |  |  |  |  |
| Negative Regulation Of Dendritic Cell Antigen Processing And Presentation | | 1 | | 50 | 1.39E-02 | 0.006 | THBS1 | |  |  |  |  |  |  |  |  |  |  |  |  |  |  |  |  |  |  |  |  |  |  |  |
| Positive Regulation Of Activation-Induced Cell Death Of T Cells | | 1 | | 50 | 1.39E-02 | 0.006 | TGFB2 | |  |  |  |  |  |  |  |  |  |  |  |  |  |  |  |  |  |  |  |  |  |  |  |
| Positive Regulation Of Eosinophil Activation | | 1 | | 50 | 1.39E-02 | 0.006 | F2RL1 | |  |  |  |  |  |  |  |  |  |  |  |  |  |  |  |  |  |  |  |  |  |  |  |
| Coronary Vein Morphogenesis | | 1 | | 50 | 1.39E-02 | 0.006 | VEGFA | |  |  |  |  |  |  |  |  |  |  |  |  |  |  |  |  |  |  |  |  |  |  |  |
| Regulation Of Transcription From Rna Polymerase Ii Promoter Involved In Kidney Development | | 1 | | 50 | 1.39E-02 | 0.006 | BMP4 | |  |  |  |  |  |  |  |  |  |  |  |  |  |  |  |  |  |  |  |  |  |  |  |
| Regulation Of Cellular Amino Acid Biosynthetic Process | | 1 | | 50 | 1.39E-02 | 0.006 | ATP2B4 | |  |  |  |  |  |  |  |  |  |  |  |  |  |  |  |  |  |  |  |  |  |  |  |
| Lymph Vessel Morphogenesis | | 1 | | 50 | 1.39E-02 | 0.006 | VEGFA | |  |  |  |  |  |  |  |  |  |  |  |  |  |  |  |  |  |  |  |  |  |  |  |
| Reproductive Senescence | | 1 | | 50 | 1.39E-02 | 0.006 | INHBB | |  |  |  |  |  |  |  |  |  |  |  |  |  |  |  |  |  |  |  |  |  |  |  |
| Negative Regulation Of Vitamin D Receptor Signaling Pathway | | 1 | | 50 | 1.39E-02 | 0.006 | SNAI2 | |  |  |  |  |  |  |  |  |  |  |  |  |  |  |  |  |  |  |  |  |  |  |  |
| Positive Regulation Of High-Density Lipoprotein Particle Clearance | | 1 | | 50 | 1.39E-02 | 0.006 | LIPG | |  |  |  |  |  |  |  |  |  |  |  |  |  |  |  |  |  |  |  |  |  |  |  |
| Microglial Cell Proliferation | | 1 | | 50 | 1.39E-02 | 0.006 | CLU | |  |  |  |  |  |  |  |  |  |  |  |  |  |  |  |  |  |  |  |  |  |  |  |
| Bundle Of His Cell Action Potential | | 1 | | 50 | 1.39E-02 | 0.006 | SCN5A | |  |  |  |  |  |  |  |  |  |  |  |  |  |  |  |  |  |  |  |  |  |  |  |
| Isopeptide Cross-Linking | | 1 | | 50 | 1.39E-02 | 0.006 | TGM2 | |  |  |  |  |  |  |  |  |  |  |  |  |  |  |  |  |  |  |  |  |  |  |  |
| Isopeptide Cross-Linking Via N6-(L-Isoglutamyl)-L-Lysine | | 1 | | 50 | 1.39E-02 | 0.006 | TGM2 | |  |  |  |  |  |  |  |  |  |  |  |  |  |  |  |  |  |  |  |  |  |  |  |
| Regulation Of Neurofibrillary Tangle Assembly | | 1 | | 50 | 1.39E-02 | 0.006 | CLU | |  |  |  |  |  |  |  |  |  |  |  |  |  |  |  |  |  |  |  |  |  |  |  |
| Negative Regulation Of Chemokine Secretion | | 1 | | 50 | 1.39E-02 | 0.006 | F2RL1 | |  |  |  |  |  |  |  |  |  |  |  |  |  |  |  |  |  |  |  |  |  |  |  |
| Oligodendrocyte Apoptotic Process | | 1 | | 50 | 1.39E-02 | 0.006 | TNFRSF21 | |  |  |  |  |  |  |  |  |  |  |  |  |  |  |  |  |  |  |  |  |  |  |  |
| Uterine Wall Breakdown | | 1 | | 50 | 1.39E-02 | 0.006 | TGFB2 | |  |  |  |  |  |  |  |  |  |  |  |  |  |  |  |  |  |  |  |  |  |  |  |
| Regulation Of Cellular Amine Catabolic Process | | 1 | | 50 | 1.39E-02 | 0.006 | ATP2B4 | |  |  |  |  |  |  |  |  |  |  |  |  |  |  |  |  |  |  |  |  |  |  |  |
| Negative Regulation Of Cellular Amine Catabolic Process | | 1 | | 50 | 1.39E-02 | 0.006 | ATP2B4 | |  |  |  |  |  |  |  |  |  |  |  |  |  |  |  |  |  |  |  |  |  |  |  |
| Positive Regulation Of Elastin Catabolic Process | | 1 | | 50 | 1.39E-02 | 0.006 | MIR181B1 | |  |  |  |  |  |  |  |  |  |  |  |  |  |  |  |  |  |  |  |  |  |  |  |
| Negative Regulation Of Interleukin-10 Biosynthetic Process | | 1 | | 50 | 1.39E-02 | 0.006 | TRIB2 | |  |  |  |  |  |  |  |  |  |  |  |  |  |  |  |  |  |  |  |  |  |  |  |
| Negative Regulation Of Interleukin-5 Secretion | | 1 | | 50 | 1.39E-02 | 0.006 | TNFRSF21 | |  |  |  |  |  |  |  |  |  |  |  |  |  |  |  |  |  |  |  |  |  |  |  |
| Negative Regulation Of Transforming Growth Factor Beta2 Production | | 1 | | 50 | 1.39E-02 | 0.006 | GATA6 | |  |  |  |  |  |  |  |  |  |  |  |  |  |  |  |  |  |  |  |  |  |  |  |
| Negative Regulation Of Arginine Catabolic Process | | 1 | | 50 | 1.39E-02 | 0.006 | ATP2B4 | |  |  |  |  |  |  |  |  |  |  |  |  |  |  |  |  |  |  |  |  |  |  |  |
| Negative Regulation Of Cytokine Activity | | 1 | | 50 | 1.39E-02 | 0.006 | NOG | |  |  |  |  |  |  |  |  |  |  |  |  |  |  |  |  |  |  |  |  |  |  |  |
| Regulation Of Neutrophil Mediated Killing Of Bacterium | | 1 | | 50 | 1.39E-02 | 0.006 | F2RL1 | |  |  |  |  |  |  |  |  |  |  |  |  |  |  |  |  |  |  |  |  |  |  |  |
| Adrenal Cortex Formation | | 1 | | 50 | 1.39E-02 | 0.006 | WT1 | |  |  |  |  |  |  |  |  |  |  |  |  |  |  |  |  |  |  |  |  |  |  |  |
| Positive Regulation Of Mesenchymal Stem Cell Proliferation | | 1 | | 50 | 1.39E-02 | 0.006 | BMP4 | |  |  |  |  |  |  |  |  |  |  |  |  |  |  |  |  |  |  |  |  |  |  |  |
| Vegf-Activated Neuropilin Signaling Pathway | | 1 | | 50 | 1.39E-02 | 0.006 | VEGFA | |  |  |  |  |  |  |  |  |  |  |  |  |  |  |  |  |  |  |  |  |  |  |  |
| Positive Regulation Of Microglial Cell Migration | | 1 | | 50 | 1.39E-02 | 0.006 | CSF1 | |  |  |  |  |  |  |  |  |  |  |  |  |  |  |  |  |  |  |  |  |  |  |  |
| Positive Regulation Of Collagen Fibril Organization | | 1 | | 50 | 1.39E-02 | 0.006 | COLGALT1 | |  |  |  |  |  |  |  |  |  |  |  |  |  |  |  |  |  |  |  |  |  |  |  |
| Negative Regulation Of Cardioblast Differentiation | | 1 | | 50 | 1.39E-02 | 0.006 | PRICKLE1 | |  |  |  |  |  |  |  |  |  |  |  |  |  |  |  |  |  |  |  |  |  |  |  |
| Regulation Of Macrophage Proliferation | | 1 | | 50 | 1.39E-02 | 0.006 | MIR181B1 | |  |  |  |  |  |  |  |  |  |  |  |  |  |  |  |  |  |  |  |  |  |  |  |
| Positive Regulation Of Macrophage Proliferation | | 1 | | 50 | 1.39E-02 | 0.006 | MIR181B1 | |  |  |  |  |  |  |  |  |  |  |  |  |  |  |  |  |  |  |  |  |  |  |  |
| Regulation Of Neutrophil Mediated Killing Of Gram-Negative Bacterium | | 1 | | 50 | 1.39E-02 | 0.006 | F2RL1 | |  |  |  |  |  |  |  |  |  |  |  |  |  |  |  |  |  |  |  |  |  |  |  |
| Pattern Specification Involved In Mesonephros Development | | 1 | | 50 | 1.39E-02 | 0.006 | BMP4 | |  |  |  |  |  |  |  |  |  |  |  |  |  |  |  |  |  |  |  |  |  |  |  |
| Macrophage Proliferation | | 1 | | 50 | 1.39E-02 | 0.006 | CLU | |  |  |  |  |  |  |  |  |  |  |  |  |  |  |  |  |  |  |  |  |  |  |  |
| Growth | | 8 | | 1 | 1.40E-02 | 0.013 | ZMIZ1, HEG1, HLA-A, CSF1, PLXNA1, SORBS2, BMP4, TIMP3 | |  |  |  |  |  |  |  |  |  |  |  |  |  |  |  |  |  |  |  |  |  |  |  |
| Positive Regulation Of Lipid Transport | | 3 | | 4 | 1.40E-02 | 0.012 | SCP2, ABCA1, LIPG | |  |  |  |  |  |  |  |  |  |  |  |  |  |  |  |  |  |  |  |  |  |  |  |
| Small Molecule Metabolic Process | | 23 | | 1 | 1.40E-02 | 0.011 | PON2, HECTD4, PDE10A, ABCA1, MAMDC2, AK4, PTGR1, SCP2, ACOXL, CYP24A1, CEMIP, CPT1A, VEGFA, ACSL1, FBP1, SLC7A5, HAS2, ELOVL7, CASK, PTGES, RAD50, SLC37A2, CHST15 | |  |  |  |  |  |  |  |  |  |  |  |  |  |  |  |  |  |  |  |  |  |  |  |
| Antigen Processing And Presentation Of Exogenous Peptide Antigen Via Mhc Class I, Tap-Dependent | | 2 | | 7 | 1.41E-02 | 0.010 | HLA-A, HLA-B | |  |  |  |  |  |  |  |  |  |  |  |  |  |  |  |  |  |  |  |  |  |  |  |
| Cell Aggregation | | 2 | | 7 | 1.41E-02 | 0.010 | BMP1, TGFB2 | |  |  |  |  |  |  |  |  |  |  |  |  |  |  |  |  |  |  |  |  |  |  |  |
| Positive Regulation Of Toll-Like Receptor Signaling Pathway | | 2 | | 7 | 1.41E-02 | 0.010 | TLR3, F2RL1 | |  |  |  |  |  |  |  |  |  |  |  |  |  |  |  |  |  |  |  |  |  |  |  |
| Long-Chain Fatty-Acyl-Coa Metabolic Process | | 2 | | 7 | 1.41E-02 | 0.010 | ELOVL7, ACSL1 | |  |  |  |  |  |  |  |  |  |  |  |  |  |  |  |  |  |  |  |  |  |  |  |
| Trabecula Formation | | 2 | | 7 | 1.41E-02 | 0.010 | TGFBR3, VEGFA | |  |  |  |  |  |  |  |  |  |  |  |  |  |  |  |  |  |  |  |  |  |  |  |
| Cellular Response To External Stimulus | | 8 | | 1 | 1.41E-02 | 0.013 | MAP3K1, LTBP1, OPTN, INHBB, ITGB3, CPEB4, BMP4, TLR3 | |  |  |  |  |  |  |  |  |  |  |  |  |  |  |  |  |  |  |  |  |  |  |  |
| Gland Development | | 8 | | 1 | 1.41E-02 | 0.013 | GATA6, NOG, AK4, INHBB, WT1, BMP4, TGFB2, TGFBR3 | |  |  |  |  |  |  |  |  |  |  |  |  |  |  |  |  |  |  |  |  |  |  |  |
| Regulation Of Endothelial Cell Proliferation | | 5 | | 2 | 1.44E-02 | 0.013 | MEF2C, VEGFA, ITGB3, BMP4, THBS1 | |  |  |  |  |  |  |  |  |  |  |  |  |  |  |  |  |  |  |  |  |  |  |  |
| Cellular Response To Dexamethasone Stimulus | | 3 | | 4 | 1.45E-02 | 0.012 | FBXO32, VEGFA, BMP4 | |  |  |  |  |  |  |  |  |  |  |  |  |  |  |  |  |  |  |  |  |  |  |  |
| Antigen Processing And Presentation Of Exogenous Peptide Antigen | | 4 | | 2 | 1.46E-02 | 0.013 | HLA-A, HLA-B, HLA-DRA, CTSD | |  |  |  |  |  |  |  |  |  |  |  |  |  |  |  |  |  |  |  |  |  |  |  |
| Positive Regulation Of Muscle Cell Differentiation | | 4 | | 2 | 1.46E-02 | 0.013 | CDON, MEF2C, BMP4, EFNB2 | |  |  |  |  |  |  |  |  |  |  |  |  |  |  |  |  |  |  |  |  |  |  |  |
| Regulation Of Metabolic Process | | 74 | | 0 | 1.47E-02 | 0.009 | ARRB1, ATP2B4, ABCA1, TSPYL5, CLU, BMP4, C4BPB, TRIM6, CPT1A, CSF1, CST4, LPAR1, EYA4, F2RL1, ACSL1, EFEMP1, FBP1, HAS2, GATA6, GBP1, PRICKLE1, IGFBP4, INHBB, ITGB3, SH3RF2, MEF2C, MEIS3P1, MAP3K1, SAMD9L, LTBP1, ADGRF1, SCP2, SOX4, SNAI2, VEGFA, WT1, TGFA, TGFB2, THBS1, TGFBR3, TIMP3, TLR3, ZNF391, ZNF43, GPRC5A, CST7, CASK, TNFSF10, PDLIM1, NOG, LIPG, RAD50, OPTN, MBNL2, CTDSPL, NUAK1, PLK2, MIR181B1, TDRD7, ARL2BP, TIAM2, TNFRSF21, UCA1, TRIB2, CDON, MIR604, KCNIP3, ZMIZ1, CEMIP, ZNF608, PMEPA1, NXN, CPEB4, AFAP1L2 | |  |  |  |  |  |  |  |  |  |  |  |  |  |  |  |  |  |  |  |  |  |  |  |
| Regulation Of Toll-Like Receptor Signaling Pathway | | 3 | | 4 | 1.51E-02 | 0.012 | PTPRS, TLR3, F2RL1 | |  |  |  |  |  |  |  |  |  |  |  |  |  |  |  |  |  |  |  |  |  |  |  |
| Regulation Of Protein Localization | | 15 | | 1 | 1.51E-02 | 0.011 | TNFRSF21, ARRB1, GBP1, INHBB, BMP4, CASK, SOX4, CEMIP, ARL2BP, TRIM6, CPT1A, LCP1, VEGFA, TGFB2, F2RL1 | |  |  |  |  |  |  |  |  |  |  |  |  |  |  |  |  |  |  |  |  |  |  |  |
| Muscle Cell Proliferation | | 2 | | 7 | 1.52E-02 | 0.010 | TGFB2, TGFBR3 | |  |  |  |  |  |  |  |  |  |  |  |  |  |  |  |  |  |  |  |  |  |  |  |
| Lung Morphogenesis | | 2 | | 7 | 1.52E-02 | 0.010 | BMP4, NOG | |  |  |  |  |  |  |  |  |  |  |  |  |  |  |  |  |  |  |  |  |  |  |  |
| Negative Regulation Of Fibroblast Growth Factor Receptor Signaling Pathway | | 2 | | 7 | 1.52E-02 | 0.010 | THBS1, SHISA2 | |  |  |  |  |  |  |  |  |  |  |  |  |  |  |  |  |  |  |  |  |  |  |  |
| Positive Regulation Of Striated Muscle Cell Apoptotic Process | | 2 | | 7 | 1.52E-02 | 0.010 | GATA6, FBXO32 | |  |  |  |  |  |  |  |  |  |  |  |  |  |  |  |  |  |  |  |  |  |  |  |
| Positive Regulation Of Cardiac Muscle Cell Apoptotic Process | | 2 | | 7 | 1.52E-02 | 0.010 | GATA6, FBXO32 | |  |  |  |  |  |  |  |  |  |  |  |  |  |  |  |  |  |  |  |  |  |  |  |
| Positive Regulation Of P38Mapk Cascade | | 2 | | 7 | 1.52E-02 | 0.010 | MIR181B1, VEGFA | |  |  |  |  |  |  |  |  |  |  |  |  |  |  |  |  |  |  |  |  |  |  |  |
| Positive Regulation Of Endothelial Cell Apoptotic Process | | 2 | | 7 | 1.52E-02 | 0.010 | BMP4, THBS1 | |  |  |  |  |  |  |  |  |  |  |  |  |  |  |  |  |  |  |  |  |  |  |  |
| Middle Ear Morphogenesis | | 2 | | 7 | 1.52E-02 | 0.010 | NOG, EYA4 | |  |  |  |  |  |  |  |  |  |  |  |  |  |  |  |  |  |  |  |  |  |  |  |
| Blood Coagulation | | 5 | | 2 | 1.52E-02 | 0.013 | ITGB3, GATA6, C4BPB, F2RL1, F13A1 | |  |  |  |  |  |  |  |  |  |  |  |  |  |  |  |  |  |  |  |  |  |  |  |
| Mesenchyme Development | | 3 | | 3 | 1.56E-02 | 0.012 | WT1, BMP4, THBS1 | |  |  |  |  |  |  |  |  |  |  |  |  |  |  |  |  |  |  |  |  |  |  |  |
| Positive Regulation Of Smooth Muscle Cell Migration | | 3 | | 3 | 1.56E-02 | 0.012 | HAS2, ITGB3, LPAR1 | |  |  |  |  |  |  |  |  |  |  |  |  |  |  |  |  |  |  |  |  |  |  |  |
| Actin Filament Bundle Assembly | | 3 | | 3 | 1.56E-02 | 0.012 | ARRB1, BAIAP2L1, LCP1 | |  |  |  |  |  |  |  |  |  |  |  |  |  |  |  |  |  |  |  |  |  |  |  |
| Positive Regulation Of Protein Complex Assembly | | 6 | | 2 | 1.59E-02 | 0.013 | MAP3K1, LCP1, ASAP1, VEGFA, BAIAP2L1, CLU | |  |  |  |  |  |  |  |  |  |  |  |  |  |  |  |  |  |  |  |  |  |  |  |
| Negative Regulation Of Cell-Cell Adhesion | | 5 | | 2 | 1.61E-02 | 0.013 | TNFRSF21, VEGFA, ZNF608, BMP4, SNAI2 | |  |  |  |  |  |  |  |  |  |  |  |  |  |  |  |  |  |  |  |  |  |  |  |
| Plasma Membrane Organization | | 3 | | 3 | 1.62E-02 | 0.012 | BAIAP2L1, CLU, FAT4 | |  |  |  |  |  |  |  |  |  |  |  |  |  |  |  |  |  |  |  |  |  |  |  |
| Response To Progesterone | | 3 | | 3 | 1.62E-02 | 0.012 | VEGFA, TGFB2, THBS1 | |  |  |  |  |  |  |  |  |  |  |  |  |  |  |  |  |  |  |  |  |  |  |  |
| Mesenchyme Morphogenesis | | 2 | | 7 | 1.62E-02 | 0.010 | TGFB2, NOG | |  |  |  |  |  |  |  |  |  |  |  |  |  |  |  |  |  |  |  |  |  |  |  |
| Prostate Gland Epithelium Morphogenesis | | 2 | | 7 | 1.62E-02 | 0.010 | BMP4, NOG | |  |  |  |  |  |  |  |  |  |  |  |  |  |  |  |  |  |  |  |  |  |  |  |
| Cellular Response To Thyroid Hormone Stimulus | | 2 | | 7 | 1.62E-02 | 0.010 | BMP4, INHBB | |  |  |  |  |  |  |  |  |  |  |  |  |  |  |  |  |  |  |  |  |  |  |  |
| Cellular Response To Fluid Shear Stress | | 2 | | 7 | 1.62E-02 | 0.010 | MEF2C, HAS2 | |  |  |  |  |  |  |  |  |  |  |  |  |  |  |  |  |  |  |  |  |  |  |  |
| Leukocyte Differentiation | | 7 | | 1 | 1.63E-02 | 0.013 | SOX4, MEF2C, SNX10, CSF1, VEGFA, BMP4, F2RL1 | |  |  |  |  |  |  |  |  |  |  |  |  |  |  |  |  |  |  |  |  |  |  |  |
| Coagulation | | 5 | | 2 | 1.64E-02 | 0.013 | ITGB3, GATA6, C4BPB, F2RL1, F13A1 | |  |  |  |  |  |  |  |  |  |  |  |  |  |  |  |  |  |  |  |  |  |  |  |
| Negative Regulation Of Transmembrane Receptor Protein Serine/Threonine Kinase Signaling Pathway | | 4 | | 2 | 1.65E-02 | 0.013 | TGFBR3, NOG, PMEPA1, LTBP1 | |  |  |  |  |  |  |  |  |  |  |  |  |  |  |  |  |  |  |  |  |  |  |  |
| Hemostasis | | 5 | | 2 | 1.67E-02 | 0.013 | ITGB3, GATA6, C4BPB, F2RL1, F13A1 | |  |  |  |  |  |  |  |  |  |  |  |  |  |  |  |  |  |  |  |  |  |  |  |
| Positive Regulation Of Osteoblast Differentiation | | 3 | | 3 | 1.68E-02 | 0.012 | MEF2C, VEGFA, BMP4 | |  |  |  |  |  |  |  |  |  |  |  |  |  |  |  |  |  |  |  |  |  |  |  |
| Negative Regulation Of Canonical Wnt Signaling Pathway | | 4 | | 2 | 1.69E-02 | 0.013 | IGFBP4, NOG, PRICKLE1, SNAI2 | |  |  |  |  |  |  |  |  |  |  |  |  |  |  |  |  |  |  |  |  |  |  |  |
| Cellular Component Assembly | | 30 | | 1 | 1.73E-02 | 0.010 | ARRB1, ABCA1, TSPYL5, ADGRF1, CDH11, BAIAP2L1, CLU, BMP1, HEG1, TRIM6, CPT1A, CTSD, LPAR1, TGFA, TGFBR3, TGM2, FBP1, HAS2, HLA-A, GBP1, ITGB3, HLA-DRA, MAP3K1, SNX10, LAMA3, LCP1, ASAP1, PARD6B, RAD50, OPTN | |  |  |  |  |  |  |  |  |  |  |  |  |  |  |  |  |  |  |  |  |  |  |  |
| Kidney Epithelium Development | | 3 | | 3 | 1.73E-02 | 0.012 | WT1, BMP4, NOG | |  |  |  |  |  |  |  |  |  |  |  |  |  |  |  |  |  |  |  |  |  |  |  |
| Negative Regulation Of Cytokine Secretion | | 3 | | 3 | 1.73E-02 | 0.012 | TNFRSF21, GBP1, F2RL1 | |  |  |  |  |  |  |  |  |  |  |  |  |  |  |  |  |  |  |  |  |  |  |  |
| Regulation Of Peptidyl-Lysine Acetylation | | 3 | | 3 | 1.73E-02 | 0.012 | SOX4, ARRB1, SNAI2 | |  |  |  |  |  |  |  |  |  |  |  |  |  |  |  |  |  |  |  |  |  |  |  |
| Epithelium Development | | 5 | | 2 | 1.73E-02 | 0.013 | HEG1, WT1, BMP4, NOG, SNAI2 | |  |  |  |  |  |  |  |  |  |  |  |  |  |  |  |  |  |  |  |  |  |  |  |
| Positive Regulation Of Cellular Catabolic Process | | 7 | | 1 | 1.74E-02 | 0.013 | PLK2, MIR181B1, CPT1A, PRICKLE1, OPTN, TRIB2, CLU | |  |  |  |  |  |  |  |  |  |  |  |  |  |  |  |  |  |  |  |  |  |  |  |
| Endothelial Cell Proliferation | | 2 | | 6 | 1.74E-02 | 0.010 | VEGFA, BMP4 | |  |  |  |  |  |  |  |  |  |  |  |  |  |  |  |  |  |  |  |  |  |  |  |
| Regulation Of Epidermal Growth Factor-Activated Receptor Activity | | 2 | | 6 | 1.74E-02 | 0.010 | GPRC5A, TGFA | |  |  |  |  |  |  |  |  |  |  |  |  |  |  |  |  |  |  |  |  |  |  |  |
| Positive Regulation Of Pri-Mirna Transcription By Rna Polymerase Ii | | 2 | | 6 | 1.74E-02 | 0.010 | BMP4, TGFB2 | |  |  |  |  |  |  |  |  |  |  |  |  |  |  |  |  |  |  |  |  |  |  |  |
| Regulation Of Peptidyl-Tyrosine Phosphorylation | | 6 | | 2 | 1.77E-02 | 0.013 | ARL2BP, AFAP1L2, VEGFA, ITGB3, GPRC5A, TGFA | |  |  |  |  |  |  |  |  |  |  |  |  |  |  |  |  |  |  |  |  |  |  |  |
| Regulation Of Cellular Metabolic Process | | 65 | | 0 | 1.79E-02 | 0.009 | ARRB1, ATP2B4, TSPYL5, CLU, BMP4, C4BPB, TRIM6, CPT1A, CSF1, CST4, LPAR1, EYA4, F2RL1, ACSL1, EFEMP1, FBP1, HAS2, GATA6, GBP1, PRICKLE1, IGFBP4, INHBB, ITGB3, SH3RF2, MEF2C, MEIS3P1, MAP3K1, ADGRF1, SOX4, SNAI2, VEGFA, WT1, TGFA, TGFB2, THBS1, TGFBR3, TIMP3, TLR3, ZNF391, ZNF43, GPRC5A, CST7, CASK, TNFSF10, PDLIM1, NOG, LIPG, RAD50, OPTN, MBNL2, CTDSPL, NUAK1, PLK2, MIR181B1, ARL2BP, TRIB2, CDON, KCNIP3, ZMIZ1, CEMIP, ZNF608, PMEPA1, NXN, CPEB4, AFAP1L2 | |  |  |  |  |  |  |  |  |  |  |  |  |  |  |  |  |  |  |  |  |  |  |  |
| Smad Protein Signal Transduction | | 3 | | 3 | 1.79E-02 | 0.012 | INHBB, BMP4, TGFB2 | |  |  |  |  |  |  |  |  |  |  |  |  |  |  |  |  |  |  |  |  |  |  |  |
| Actin Filament Bundle Organization | | 3 | | 3 | 1.79E-02 | 0.012 | ARRB1, BAIAP2L1, LCP1 | |  |  |  |  |  |  |  |  |  |  |  |  |  |  |  |  |  |  |  |  |  |  |  |
| Negative Regulation Of Protein Ubiquitination | | 3 | | 3 | 1.79E-02 | 0.012 | SOX4, ARRB1, NXN | |  |  |  |  |  |  |  |  |  |  |  |  |  |  |  |  |  |  |  |  |  |  |  |
| Negative Regulation Of Mapk Cascade | | 5 | | 2 | 1.80E-02 | 0.013 | ARRB1, BMP4, GBP1, TIMP3, F2RL1 | |  |  |  |  |  |  |  |  |  |  |  |  |  |  |  |  |  |  |  |  |  |  |  |
| Positive Regulation Of Secretion | | 9 | | 1 | 1.80E-02 | 0.012 | SOX4, ARRB1, DPYSL2, TRIM6, INHBB, TGFB2, CASK, RAB27B, F2RL1 | |  |  |  |  |  |  |  |  |  |  |  |  |  |  |  |  |  |  |  |  |  |  |  |
| Immune Response | | 20 | | 1 | 1.83E-02 | 0.011 | ADGRE5, CLU, C4BPB, CSF1, VEGFA, THBS1, TGFBR3, SLAMF7, TLR3, F2RL1, TNFRSF21, HLA-A, HLA-B, GBP2, HLA-DRA, CST7, TNFSF10, MEF2C, FCGR2C, OPTN | |  |  |  |  |  |  |  |  |  |  |  |  |  |  |  |  |  |  |  |  |  |  |  |
| Hematopoietic Progenitor Cell Differentiation | | 4 | | 2 | 1.85E-02 | 0.013 | SOX4, SAMD9L, RBM47, BMP4 | |  |  |  |  |  |  |  |  |  |  |  |  |  |  |  |  |  |  |  |  |  |  |  |
| Plasma Lipoprotein Particle Assembly | | 2 | | 6 | 1.85E-02 | 0.010 | ABCA1, BMP1 | |  |  |  |  |  |  |  |  |  |  |  |  |  |  |  |  |  |  |  |  |  |  |  |
| Positive Regulation Of Osteoclast Differentiation | | 2 | | 6 | 1.85E-02 | 0.010 | ITGB3, CSF1 | |  |  |  |  |  |  |  |  |  |  |  |  |  |  |  |  |  |  |  |  |  |  |  |
| **Enrichment for Cellular Components** | | | | | | | | |  |  |  |  |  |  |  |  |  |  |  |  |  |  |  |  |  |  |  |  |  |  |  |
| Vesicle | 62 | | 1 | | 1.06E-12 | 0.016 | | ARRB1, ABCA1, CD22, MGAT4A, ADGRE5, CDH11, MARCH3, PTGR1, CLU, BMP4, BMP1, PRSS23, DPYSL2, DSP, CST4, CTSD, TIAM2, LPAR1, F2RL1, EFEMP1, FBP1, GBP1, GBP2, GBP3, ITGB3, CD82, HLA-DRA, SAMD9L, SNX10, LAMA3, LCP1, CLIC6, SLC37A2, SNX7, PLXNA1, BAIAP2L1, PTPRS, RAB27B, SLC44A2, CEMIP, PMEPA1, VEGFA, TGFA, TGFB2, THBS1, TGFBR3, TGM2, TLR3, SERINC2, SLC7A5, CLIC3, GPRC5A, FAT4, CDC42BPA, CASK, TNFSF10, CYTIP, CRISPLD2, LIPG, PARD6B, OPTN, CTDSPL |  |  |  |  |  |  |  |  |  |  |  |  |  |  |  |  |  |  |  |  |  |  |  |
| Extracellular Region Part | 67 | | 1 | | 2.41E-12 | 0.015 | | MIR181B1, ABCA1, CD22, MGAT4A, ADGRE5, CDH11, PTGR1, CLU, BMP4, BMP1, C4BPB, PRSS23, DPYSL2, DSP, CSF1, CST4, CTSD, TIAM2, F13A1, EFEMP1, FBP1, HLA-A, GBP1, GBP2, GBP3, IGFBP4, INHBB, ITGB3, CD82, HLA-DRA, CDON, CPA4, LAMA3, LCP1, LTBP1, CLIC6, SLC37A2, MAMDC2, PLXNA1, BAIAP2L1, SCP2, PTPRS, RAB27B, SLC44A2, MTUS1, SDC2, VEGFA, TGFA, TGFB2, THBS1, TGFBR3, TGM2, TIMP3, SERINC2, SLC7A5, CLIC3, GPRC5A, FAT4, CDC42BPA, CASK, TNFSF10, ADAMTS12, CRISPLD2, NOG, LIPG, PARD6B, CTDSPL | |  |  |  |  |  |  |  |  |  |  |  |  |  |  |  |  |  |  |  |  |  |  |
| Collagen-Containing Extracellular Matrix | 15 | | 5 | | 7.16E-10 | 0.034 | | CDON, ADAMTS12, SDC2, MAMDC2, LAMA3, CTSD, LTBP1, VEGFA, CLU, TGFB2, THBS1, TGM2, CASK, TIMP3, EFEMP1 | |  |  |  |  |  |  |  |  |  |  |  |  |  |  |  |  |  |  |  |  |  |  |
| Extracellular Matrix | 17 | | 3 | | 8.28E-09 | 0.028 | | CDON, ADAMTS12, CRISPLD2, SDC2, MAMDC2, LAMA3, CTSD, LTBP1, VEGFA, CLU, TGFB2, THBS1, TGFBR3, TGM2, CASK, TIMP3, EFEMP1 | |  |  |  |  |  |  |  |  |  |  |  |  |  |  |  |  |  |  |  |  |  |  |
| Extracellular Exosome | 39 | | 1 | | 1.01E-08 | 0.017 | | PLXNA1, CD22, MGAT4A, ADGRE5, CDH11, PTGR1, BAIAP2L1, CLU, PTPRS, RAB27B, PRSS23, SLC44A2, DPYSL2, DSP, CST4, CTSD, TIAM2, THBS1, TGFBR3, TGM2, SERINC2, EFEMP1, FBP1, SLC7A5, CLIC3, ITGB3, GPRC5A, CD82, HLA-DRA, FAT4, CDC42BPA, TNFSF10, CRISPLD2, LAMA3, LCP1, PARD6B, CTDSPL, CLIC6, SLC37A2 | |  |  |  |  |  |  |  |  |  |  |  |  |  |  |  |  |  |  |  |  |  |  |
| Extracellular Vesicle | 39 | | 1 | | 1.38E-08 | 0.017 | | PLXNA1, CD22, MGAT4A, ADGRE5, CDH11, PTGR1, BAIAP2L1, CLU, PTPRS, RAB27B, PRSS23, SLC44A2, DPYSL2, DSP, CST4, CTSD, TIAM2, THBS1, TGFBR3, TGM2, SERINC2, EFEMP1, FBP1, SLC7A5, CLIC3, ITGB3, GPRC5A, CD82, HLA-DRA, FAT4, CDC42BPA, TNFSF10, CRISPLD2, LAMA3, LCP1, PARD6B, CTDSPL, CLIC6, SLC37A2 | |  |  |  |  |  |  |  |  |  |  |  |  |  |  |  |  |  |  |  |  |  |  |
| Extracellular Organelle | 39 | | 1 | | 1.42E-08 | 0.017 | | PLXNA1, CD22, MGAT4A, ADGRE5, CDH11, PTGR1, BAIAP2L1, CLU, PTPRS, RAB27B, PRSS23, SLC44A2, DPYSL2, DSP, CST4, CTSD, TIAM2, THBS1, TGFBR3, TGM2, SERINC2, EFEMP1, FBP1, SLC7A5, CLIC3, ITGB3, GPRC5A, CD82, HLA-DRA, FAT4, CDC42BPA, TNFSF10, CRISPLD2, LAMA3, LCP1, PARD6B, CTDSPL, CLIC6, SLC37A2 | |  |  |  |  |  |  |  |  |  |  |  |  |  |  |  |  |  |  |  |  |  |  |
| Membrane-Bounded Organelle | 119 | | 0 | | 1.91E-07 | 0.009 | | ARRB1, ATP2B4, ABCA1, AK4, CD22, ADGRE5, CDH11, CLU, BMP4, BMP1, CYP24A1, DPYSL2, DSP, CPT1A, CST4, CTSD, FLG, LPAR1, EYA4, F2RL1, ACSL1, EFEMP1, FBP1, GPR1, HLA-A, HLA-B, GATA6, GBP1, GBP2, GBP3, ITGB3, CD82, HLA-DRA, MEF2C, MEIS3P1, LAMA3, LCP1, LTBP1, PON2, PLXNA1, RRBP1, SCN5A, SCP2, PTPRS, RAB27B, SOX4, SNAI2, UGCG, VEGFA, WT1, TGFA, TGFB2, THBS1, TGFBR3, TGM2, TIMP3, TLR3, SLC7A5, ZNF43, LAPTM5, EVI5, CLIC3, GPRC5A, SORBS2, CDC42BPA, CASK, TNFSF10, PTGES, CYTIP, LIPG, RAD50, OPTN, MBNL2, CTDSPL, NUAK1, MGAT4A, PTGR1, PRSS23, ARL2BP, TIAM2, TRIB2, SNX10, KCNIP3, CLIC6, CHST15, SNX7, LPCAT2, RBM47, BAIAP2L1, ACOXL, SLC44A2, ZMIZ1, CEMIP, MTUS1, PMEPA1, NXN, HHIP, SLAMF7, PORCN, CDK15, ELOVL7, CPEB4, FAT4, MORC4, COLGALT1, CRISPLD2, PARD6B, TSPYL5, FBXO32, MARCH3, TRIM6, PRICKLE1, SAMD9L, SLC37A2, MAMDC2, ZNF391, SERINC2, ILDR2, SHISA2 | |  |  |  |  |  |  |  |  |  |  |  |  |  |  |  |  |  |  |  |  |  |  |
| Cell Surface | 20 | | 2 | | 2.63E-07 | 0.022 | | HLA-A, HLA-B, ABCA1, CDH11, ITGB3, CLU, SCN5A, HLA-DRA, CDON, HEG1, SDC2, LIPG, VEGFA, HHIP, LPAR1, TGFA, TGFB2, THBS1, TGFBR3, TLR3 | |  |  |  |  |  |  |  |  |  |  |  |  |  |  |  |  |  |  |  |  |  |  |
| Cytoplasmic Part | 107 | | 0 | | 2.98E-07 | 0.010 | | ARRB1, ATP2B4, ABCA1, AK4, ADGRE5, FBXO32, MARCH3, CLU, BMP4, BMP1, CYP24A1, DPYSL2, DSP, TRIM6, CPT1A, CSF1, CTSD, FLG, LPAR1, F2RL1, F13A1, ACSL1, FBP1, HLA-A, HLA-B, GBP1, GBP2, GBP3, PRICKLE1, IGFBP4, INHBB, ITGB3, HLA-DRA, MEF2C, MAP3K1, SAMD9L, LAMA3, LCP1, COX7B2, LTBP1, SLC37A2, PON2, MAMDC2, RRBP1, SCN5A, SCP2, PTPRS, RAB27B, SOX4, SDC2, UGCG, VEGFA, WT1, TGFA, TGFB2, THBS1, TGFBR3, TGM2, TIMP3, TLR3, SERINC2, SLC7A5, ILDR2, SHISA2, LAPTM5, EVI5, GPRC5A, SORBS2, CASK, PTGES, CYTIP, PDLIM1, LIPG, RAD50, OPTN, PLK2, PDE10A, MYL9, MGAT4A, PRSS23, TDRD7, ARL2BP, TIAM2, SNX10, KCNIP3, ASAP1, CHST15, SNX7, LPCAT2, BAIAP2L1, ACOXL, CPPED1, SLC44A2, CEMIP, MTUS1, PMEPA1, NXN, SLAMF7, TNS3, PORCN, CDK15, ELOVL7, CPEB4, COLGALT1, CRISPLD2, PARD6B, AFAP1L2 | |  |  |  |  |  |  |  |  |  |  |  |  |  |  |  |  |  |  |  |  |  |  |
| Extracellular Region | 40 | | 1 | | 7.15E-07 | 0.014 | | PON2, MAMDC2, ADGRF1, MGAT4A, CLU, BMP4, BMP1, CPPED1, C4BPB, PRSS23, CEMIP, HEG1, CSF1, CTSD, TUFT1, VEGFA, HHIP, TGFA, TGFB2, THBS1, TGFBR3, TIMP3, TLL1, LYPD6B, F13A1, EFEMP1, GBP1, GBP3, IGFBP4, INHBB, CST7, TNFSF10, ADAMTS12, CRISPLD2, CPA4, APOL6, NOG, LAMA3, LIPG, LTBP1 | |  |  |  |  |  |  |  |  |  |  |  |  |  |  |  |  |  |  |  |  |  |  |
| Cytoplasmic Vesicle Part | 28 | | 1 | | 1.44E-06 | 0.017 | | ARRB1, ADGRE5, MARCH3, CLU, PTPRS, CPPED1, RAB27B, SLC44A2, CEMIP, DSP, PMEPA1, CTSD, VEGFA, TGFA, TGFB2, THBS1, TIMP3, TLR3, F13A1, HLA-A, HLA-B, ITGB3, GPRC5A, HLA-DRA, CRISPLD2, SNX10, OPTN, SNX7 | |  |  |  |  |  |  |  |  |  |  |  |  |  |  |  |  |  |  |  |  |  |  |
| Membrane | 103 | | 0 | | 1.88E-06 | 0.009 | | ARRB1, ATP2B4, ABCA1, CD22, ADGRE5, CDH11, MARCH3, CLU, C4BPB, CYP24A1, DPYSL2, DSP, CPT1A, OR5P2, CSF1, CTSD, FLG, LPAR1, EFNB2, LYPD6B, F2RL1, ACSL1, GPR1, TMEM56, HAS2, HLA-A, HLA-B, GATA6, GBP1, GBP2, GBP3, PRICKLE1, ITGB3, CD82, HLA-DRA, MAP3K1, LCP1, MPZL3, PLPP4, SLC37A2, PON2, TMPRSS15, MAMDC2, ADGRF1, PLXNA1, RRBP1, SCN5A, SCP2, SCN9A, PTPRS, RAB27B, SDC2, UGCG, VEGFA, TGFA, TGFBR3, TGM2, TLR3, SERINC2, SLC7A5, ILDR2, SHISA2, LAPTM5, CLIC3, GPRC5A, SORBS2, CASK, TNFSF10, PTGES, FCGR2C, TSPAN2, RAD50, OPTN, PDE10A, MGAT4A, TIAM2, TNFRSF21, CDON, SNX10, KCNIP3, ASAP1, CLIC6, CHST15, SNX7, LPCAT2, TENM3, BAIAP2L1, CPPED1, SLC44A2, CEMIP, HEG1, MTUS1, SORCS2, PMEPA1, HHIP, SLAMF7, PORCN, ELOVL7, CPEB4, FAT4, COLGALT1, PARD6B, AFAP1L2 | |  |  |  |  |  |  |  |  |  |  |  |  |  |  |  |  |  |  |  |  |  |  |
| Organelle | 126 | | 0 | | 2.03E-06 | 0.009 | | ARRB1, ATP2B4, ABCA1, AK4, CD22, ADGRE5, CDH11, CLU, BMP4, BMP1, CYP24A1, DPYSL2, DSP, CPT1A, CST4, CTSD, FLG, LPAR1, EYA4, F2RL1, ACSL1, EFEMP1, FBP1, GPR1, HLA-A, HLA-B, GATA6, GBP1, GBP2, GBP3, ITGB3, CD82, HLA-DRA, MEF2C, MEIS3P1, MAP3K1, LAMA3, LCP1, LTBP1, PON2, PLXNA1, RRBP1, SCN5A, SCP2, PTPRS, RAB27B, SOX4, SNAI2, UGCG, VEGFA, WT1, TGFA, TGFB2, THBS1, TGFBR3, TGM2, TIMP3, TLR3, SLC7A5, ZNF43, LAPTM5, EVI5, CLIC3, GPRC5A, SORBS2, CDC42BPA, CASK, TNFSF10, PTGES, CYTIP, PDLIM1, LIPG, RAD50, OPTN, MBNL2, CTDSPL, NUAK1, PLK2, MGAT4A, PTGR1, PRSS23, TDRD7, ARL2BP, TIAM2, TRIB2, SNX10, KCNIP3, ASAP1, CLIC6, CHST15, SNX7, LPCAT2, RBM47, BAIAP2L1, KLHL4, ACOXL, SLC44A2, ZMIZ1, CEMIP, MTUS1, PMEPA1, NXN, HHIP, SLAMF7, PORCN, CDK15, ELOVL7, CPEB4, FAT4, MORC4, COLGALT1, CRISPLD2, PARD6B, TSPYL5, FBXO32, MARCH3, TRIM6, PRICKLE1, SAMD9L, SLC37A2, MAMDC2, ZNF391, SERINC2, ILDR2, SHISA2, SNORD123 | |  |  |  |  |  |  |  |  |  |  |  |  |  |  |  |  |  |  |  |  |  |  |
| Plasma Membrane Part | 47 | | 1 | | 4.52E-06 | 0.012 | | ARRB1, ATP2B4, ABCA1, CD22, ADGRE5, CDH11, DSP, LPAR1, EFNB2, F2RL1, GPR1, TNFRSF21, HAS2, HLA-A, HLA-B, ITGB3, CD82, HLA-DRA, CDON, LCP1, KCNIP3, ASAP1, PLPP4, PLXNA1, TENM3, SCN5A, SCN9A, PTPRS, RAB27B, HEG1, HHIP, TGFA, THBS1, TGFBR3, TGM2, SLAMF7, TLR3, SLC7A5, PORCN, LAPTM5, CPEB4, GPRC5A, SORBS2, CASK, TNFSF10, TSPAN2, PARD6B | |  |  |  |  |  |  |  |  |  |  |  |  |  |  |  |  |  |  |  |  |  |  |
| Intracellular Organelle Lumen | 23 | | 1 | | 1.49E-05 | 0.016 | | AK4, MGAT4A, IGFBP4, CLU, SCP2, BMP4, ACOXL, CPPED1, COLGALT1, PRSS23, TDRD7, PTGES, ARL2BP, CRISPLD2, SDC2, CSF1, CTSD, LTBP1, VEGFA, TGFB2, THBS1, TIMP3, F13A1 | |  |  |  |  |  |  |  |  |  |  |  |  |  |  |  |  |  |  |  |  |  |  |
| Membrane-Enclosed Lumen | 23 | | 1 | | 1.57E-05 | 0.016 | | AK4, MGAT4A, IGFBP4, CLU, SCP2, BMP4, ACOXL, CPPED1, COLGALT1, PRSS23, TDRD7, PTGES, ARL2BP, CRISPLD2, SDC2, CSF1, CTSD, LTBP1, VEGFA, TGFB2, THBS1, TIMP3, F13A1 | |  |  |  |  |  |  |  |  |  |  |  |  |  |  |  |  |  |  |  |  |  |  |
| Organelle Lumen | 23 | | 1 | | 1.57E-05 | 0.016 | | AK4, MGAT4A, IGFBP4, CLU, SCP2, BMP4, ACOXL, CPPED1, COLGALT1, PRSS23, TDRD7, PTGES, ARL2BP, CRISPLD2, SDC2, CSF1, CTSD, LTBP1, VEGFA, TGFB2, THBS1, TIMP3, F13A1 | |  |  |  |  |  |  |  |  |  |  |  |  |  |  |  |  |  |  |  |  |  |  |
| Endoplasmic Reticulum Part | 25 | | 1 | | 1.69E-05 | 0.015 | | LPCAT2, ABCA1, MGAT4A, BMP4, PRSS23, SDC2, CPT1A, CSF1, TGFA, THBS1, TLR3, ACSL1, SERINC2, ILDR2, SHISA2, HLA-A, HLA-B, PORCN, IGFBP4, ELOVL7, HLA-DRA, COLGALT1, PTGES, LTBP1, SLC37A2 | |  |  |  |  |  |  |  |  |  |  |  |  |  |  |  |  |  |  |  |  |  |  |
| Symbiont-Containing Vacuole Membrane | 3 | | 30 | | 3.47E-05 | 0.016 | | GBP1, GBP2, GBP3 | |  |  |  |  |  |  |  |  |  |  |  |  |  |  |  |  |  |  |  |  |  |  |
| Intrinsic Component Of Plasma Membrane | 28 | | 1 | | 5.53E-05 | 0.014 | | ATP2B4, ABCA1, PLXNA1, CD22, TENM3, ADGRE5, SCN9A, PTPRS, HHIP, LPAR1, EFNB2, TGFA, TGFBR3, TGM2, TLR3, F2RL1, GPR1, TNFRSF21, HAS2, LAPTM5, ITGB3, GPRC5A, CD82, HLA-DRA, TNFSF10, CDON, PLPP4, TSPAN2 | |  |  |  |  |  |  |  |  |  |  |  |  |  |  |  |  |  |  |  |  |  |  |
| Integral Component Of Plasma Membrane | 27 | | 1 | | 6.05E-05 | 0.014 | | ATP2B4, ABCA1, PLXNA1, CD22, TENM3, ADGRE5, SCN9A, PTPRS, HHIP, LPAR1, EFNB2, TGFA, TGFBR3, TLR3, F2RL1, GPR1, TNFRSF21, HAS2, LAPTM5, ITGB3, GPRC5A, CD82, HLA-DRA, TNFSF10, CDON, PLPP4, TSPAN2 | |  |  |  |  |  |  |  |  |  |  |  |  |  |  |  |  |  |  |  |  |  |  |
| Cytoplasm | 73 | | 0 | | 7.37E-05 | 0.010 | | ARRB1, CDH11, FBXO32, CLU, BMP4, DPYSL2, DSP, TRIM6, LPAR1, EYA4, F13A1, EFEMP1, FBP1, HAS2, GBP1, GBP2, GBP3, PRICKLE1, MEF2C, MAP3K1, SAMD9L, LCP1, LTBP1, SCN5A, SCP2, SOX4, SNAI2, TUFT1, VEGFA, WT1, TGFB2, THBS1, TGFBR3, TGM2, TIMP3, TLR3, SLC7A5, EVI5, CLIC3, SORBS2, CDC42BPA, CST7, CASK, PTGES, CYTIP, FCGR2C, PDLIM1, OPTN, MBNL2, NUAK1, PLK2, PDE10A, PTGR1, TDRD7, ARL2BP, TIAM2, TRIB2, SNX10, KCNIP3, ASAP1, CLIC6, BAIAP2L1, KLHL4, CPPED1, ZMIZ1, CEMIP, MTUS1, HHIP, CDK15, CPEB4, APOL6, PARD6B, AFAP1L2 | |  |  |  |  |  |  |  |  |  |  |  |  |  |  |  |  |  |  |  |  |  |  |
| Platelet Alpha Granule Lumen | 5 | | 7 | | 8.95E-05 | 0.021 | | VEGFA, CLU, TGFB2, THBS1, F13A1 | |  |  |  |  |  |  |  |  |  |  |  |  |  |  |  |  |  |  |  |  |  |  |
| Adherens Junction | 13 | | 2 | | 1.03E-04 | 0.018 | | DSP, PDLIM1, TNS3, LAMA3, LCP1, ADGRE5, CDH11, ITGB3, BAIAP2L1, SORBS2, EFNB2, TGM2, CASK | |  |  |  |  |  |  |  |  |  |  |  |  |  |  |  |  |  |  |  |  |  |  |
| Plasma Membrane | 66 | | 1 | | 1.08E-04 | 0.010 | | ARRB1, ATP2B4, ABCA1, CD22, ADGRE5, CDH11, C4BPB, CYP24A1, DPYSL2, DSP, OR5P2, CSF1, FLG, LPAR1, EFNB2, LYPD6B, F2RL1, ACSL1, GPR1, HLA-A, HLA-B, GBP1, GBP3, ITGB3, CD82, HLA-DRA, LCP1, PLPP4, PON2, ADGRF1, PLXNA1, SCN5A, SCN9A, PTPRS, RAB27B, SDC2, VEGFA, TGFA, TGFBR3, TGM2, SERINC2, SLC7A5, GPRC5A, SORBS2, CASK, FCGR2C, TSPAN2, TNFRSF21, CDON, KCNIP3, ASAP1, CLIC6, TENM3, BAIAP2L1, CPPED1, SLC44A2, CEMIP, HEG1, MTUS1, PMEPA1, HHIP, SLAMF7, CPEB4, FAT4, PARD6B, AFAP1L2 | |  |  |  |  |  |  |  |  |  |  |  |  |  |  |  |  |  |  |  |  |  |  |
| Basement Membrane | 6 | | 5 | | 1.12E-04 | 0.021 | | VEGFA, TGFB2, CASK, TIMP3, LAMA3, EFEMP1 | |  |  |  |  |  |  |  |  |  |  |  |  |  |  |  |  |  |  |  |  |  |  |
| Cytoplasmic Vesicle | 27 | | 1 | | 1.26E-04 | 0.013 | | ARRB1, ABCA1, MARCH3, CLU, PTPRS, RAB27B, CEMIP, PMEPA1, CTSD, VEGFA, LPAR1, TGFA, TGFB2, THBS1, TLR3, F2RL1, GBP1, GBP2, GBP3, ITGB3, CYTIP, CRISPLD2, SAMD9L, SNX10, LIPG, OPTN, SNX7 | |  |  |  |  |  |  |  |  |  |  |  |  |  |  |  |  |  |  |  |  |  |  |
| Endoplasmic Reticulum | 25 | | 1 | | 1.27E-04 | 0.014 | | LPCAT2, MAMDC2, RRBP1, CLU, SCN5A, SCP2, CEMIP, THBS1, TGFBR3, TGM2, SLAMF7, TLR3, ACSL1, ILDR2, SHISA2, HLA-A, HLA-B, PORCN, ELOVL7, CPEB4, COLGALT1, SNX10, LAMA3, KCNIP3, SLC37A2 | |  |  |  |  |  |  |  |  |  |  |  |  |  |  |  |  |  |  |  |  |  |  |
| Intracellular Vesicle | 27 | | 1 | | 1.32E-04 | 0.013 | | ARRB1, ABCA1, MARCH3, CLU, PTPRS, RAB27B, CEMIP, PMEPA1, CTSD, VEGFA, LPAR1, TGFA, TGFB2, THBS1, TLR3, F2RL1, GBP1, GBP2, GBP3, ITGB3, CYTIP, CRISPLD2, SAMD9L, SNX10, LIPG, OPTN, SNX7 | |  |  |  |  |  |  |  |  |  |  |  |  |  |  |  |  |  |  |  |  |  |  |
| Anchoring Junction | 13 | | 2 | | 1.44E-04 | 0.018 | | DSP, PDLIM1, TNS3, LAMA3, LCP1, ADGRE5, CDH11, ITGB3, BAIAP2L1, SORBS2, EFNB2, TGM2, CASK | |  |  |  |  |  |  |  |  |  |  |  |  |  |  |  |  |  |  |  |  |  |  |
| Golgi Membrane | 15 | | 1 | | 1.87E-04 | 0.016 | | LPCAT2, ARRB1, HLA-A, HLA-B, GBP1, GBP2, GBP3, MGAT4A, HLA-DRA, PMEPA1, OPTN, UGCG, CHST15, TGFA, TLR3 | |  |  |  |  |  |  |  |  |  |  |  |  |  |  |  |  |  |  |  |  |  |  |
| Golgi Apparatus Part | 18 | | 1 | | 2.31E-04 | 0.015 | | LPCAT2, ARRB1, HLA-A, HLA-B, GBP1, GBP2, GBP3, MGAT4A, HLA-DRA, RAB27B, SDC2, PMEPA1, OPTN, UGCG, CHST15, TGFA, TGFB2, TLR3 | |  |  |  |  |  |  |  |  |  |  |  |  |  |  |  |  |  |  |  |  |  |  |
| Other Organism Part | 4 | | 8 | | 2.37E-04 | 0.018 | | GBP1, C4BPB, GBP2, GBP3 | |  |  |  |  |  |  |  |  |  |  |  |  |  |  |  |  |  |  |  |  |  |  |
| Vesicle Membrane | 15 | | 1 | | 2.50E-04 | 0.016 | | ARRB1, HLA-A, HLA-B, GBP1, ADGRE5, MARCH3, ITGB3, GPRC5A, HLA-DRA, RAB27B, SLC44A2, CEMIP, DSP, TGFA, SNX7 | |  |  |  |  |  |  |  |  |  |  |  |  |  |  |  |  |  |  |  |  |  |  |
| Intracellular Part | 137 | | 0 | | 2.63E-04 | 0.008 | | ARRB1, ATP2B4, ABCA1, AK4, ADGRE5, CDH11, CLU, BMP4, BMP1, CYP24A1, DPYSL2, DSP, CPT1A, CSF1, CTSD, FLG, LPAR1, EYA4, F2RL1, F13A1, ACSL1, EFEMP1, FBP1, GPR1, HAS2, HLA-A, HLA-B, GATA6, GBP1, GBP2, GBP3, IGFBP4, INHBB, ITGB3, HLA-DRA, MEF2C, MEIS3P1, MAP3K1, LAMA3, LCP1, LTBP1, PON2, RRBP1, SCN5A, SCP2, PTPRS, RAB27B, SOX4, SDC2, SNAI2, TUFT1, UGCG, VEGFA, WT1, TGFA, TGFB2, THBS1, TGFBR3, TGM2, TIMP3, TLR3, SLC7A5, ZNF43, LAPTM5, EVI5, CLIC3, GPRC5A, SORBS2, CDC42BPA, CST7, CASK, PTGES, CYTIP, FCGR2C, PDLIM1, LIPG, RAD50, OPTN, MBNL2, CTDSPL, NUAK1, PLK2, PDE10A, MYL9, MGAT4A, PTGR1, PRSS23, TDRD7, ARL2BP, TIAM2, TRIB2, SNX10, KCNIP3, ASAP1, CLIC6, CHST15, SNX7, LPCAT2, RBM47, BAIAP2L1, KLHL4, ACOXL, CPPED1, SLC44A2, ZMIZ1, CEMIP, MTUS1, PMEPA1, NXN, HHIP, SLAMF7, TNS3, PORCN, CDK15, ELOVL7, CPEB4, MORC4, COLGALT1, CRISPLD2, APOL6, PARD6B, AFAP1L2, TSPYL5, FBXO32, MARCH3, TRIM6, PRICKLE1, SH3RF2, SAMD9L, COX7B2, SLC37A2, MAMDC2, ZNF391, SERINC2, ILDR2, SHISA2, SNORD123 | |  |  |  |  |  |  |  |  |  |  |  |  |  |  |  |  |  |  |  |  |  |  |
| Endoplasmic Reticulum Lumen | 9 | | 2 | | 2.69E-04 | 0.019 | | SDC2, CSF1, LTBP1, MGAT4A, IGFBP4, BMP4, THBS1, COLGALT1, PRSS23 | |  |  |  |  |  |  |  |  |  |  |  |  |  |  |  |  |  |  |  |  |  |  |
| Bounding Membrane Of Organelle | 28 | | 1 | | 3.50E-04 | 0.012 | | LPCAT2, ARRB1, MGAT4A, ADGRE5, MARCH3, RAB27B, CYP24A1, SLC44A2, CEMIP, DSP, CPT1A, PMEPA1, UGCG, HHIP, TGFA, TLR3, ACSL1, HLA-A, HLA-B, GBP1, GBP2, GBP3, LAPTM5, ITGB3, HLA-DRA, CASK, OPTN, CHST15 | |  |  |  |  |  |  |  |  |  |  |  |  |  |  |  |  |  |  |  |  |  |  |
| Secretory Granule Lumen | 9 | | 2 | | 3.63E-04 | 0.018 | | CRISPLD2, CTSD, VEGFA, CLU, CPPED1, TGFB2, THBS1, TIMP3, F13A1 | |  |  |  |  |  |  |  |  |  |  |  |  |  |  |  |  |  |  |  |  |  |  |
| Host Cell Part | 3 | | 14 | | 3.64E-04 | 0.015 | | GBP1, GBP2, GBP3 | |  |  |  |  |  |  |  |  |  |  |  |  |  |  |  |  |  |  |  |  |  |  |
| Perinuclear Region Of Cytoplasm | 15 | | 1 | | 4.41E-04 | 0.015 | | ABCA1, GBP2, GBP3, INHBB, CPEB4, CLU, SCN5A, SORBS2, PTGES, CSF1, LCP1, LTBP1, RAD50, OPTN, TGFA | |  |  |  |  |  |  |  |  |  |  |  |  |  |  |  |  |  |  |  |  |  |  |
| Cytoplasmic Vesicle Lumen | 9 | | 2 | | 5.25E-04 | 0.018 | | CRISPLD2, CTSD, VEGFA, CLU, CPPED1, TGFB2, THBS1, TIMP3, F13A1 | |  |  |  |  |  |  |  |  |  |  |  |  |  |  |  |  |  |  |  |  |  |  |
| Integral Component Of Endoplasmic Reticulum Membrane | 6 | | 3 | | 5.45E-04 | 0.019 | | ELOVL7, HLA-A, HLA-B, HLA-DRA, SLC37A2, PORCN | |  |  |  |  |  |  |  |  |  |  |  |  |  |  |  |  |  |  |  |  |  |  |
| Cytoplasmic Vesicle Membrane | 14 | | 1 | | 5.77E-04 | 0.015 | | ARRB1, HLA-A, HLA-B, ADGRE5, MARCH3, ITGB3, GPRC5A, HLA-DRA, RAB27B, SLC44A2, CEMIP, DSP, TGFA, SNX7 | |  |  |  |  |  |  |  |  |  |  |  |  |  |  |  |  |  |  |  |  |  |  |
| Vesicle Lumen | 9 | | 2 | | 5.82E-04 | 0.018 | | CRISPLD2, CTSD, VEGFA, CLU, CPPED1, TGFB2, THBS1, TIMP3, F13A1 | |  |  |  |  |  |  |  |  |  |  |  |  |  |  |  |  |  |  |  |  |  |  |
| Cell-Substrate Junction | 10 | | 2 | | 6.22E-04 | 0.017 | | PDLIM1, TNS3, LAMA3, LCP1, ADGRE5, ITGB3, SORBS2, EFNB2, TGM2, CASK | |  |  |  |  |  |  |  |  |  |  |  |  |  |  |  |  |  |  |  |  |  |  |
| Z Disc | 6 | | 3 | | 6.48E-04 | 0.018 | | FBXO32, ATP2B4, SCN5A, PDLIM1, SORBS2, MYL9 | |  |  |  |  |  |  |  |  |  |  |  |  |  |  |  |  |  |  |  |  |  |  |
| Intrinsic Component Of Endoplasmic Reticulum Membrane | 6 | | 3 | | 6.70E-04 | 0.018 | | ELOVL7, HLA-A, HLA-B, HLA-DRA, SLC37A2, PORCN | |  |  |  |  |  |  |  |  |  |  |  |  |  |  |  |  |  |  |  |  |  |  |
| Er To Golgi Transport Vesicle Membrane | 4 | | 6 | | 6.74E-04 | 0.017 | | HLA-A, HLA-B, HLA-DRA, TGFA | |  |  |  |  |  |  |  |  |  |  |  |  |  |  |  |  |  |  |  |  |  |  |
| Extracellular Space | 28 | | 1 | | 7.59E-04 | 0.012 | | MIR181B1, CLU, SCP2, BMP4, BMP1, C4BPB, PRSS23, MTUS1, CSF1, CST4, CTSD, VEGFA, TGFA, TGFB2, THBS1, TGFBR3, TIMP3, EFEMP1, HLA-A, IGFBP4, INHBB, TNFSF10, CRISPLD2, CPA4, NOG, LCP1, LIPG, LTBP1 | |  |  |  |  |  |  |  |  |  |  |  |  |  |  |  |  |  |  |  |  |  |  |
| Golgi Apparatus | 20 | | 1 | | 9.32E-04 | 0.013 | | LPCAT2, HLA-A, HLA-B, ABCA1, GBP1, GBP2, GBP3, EVI5, MGAT4A, CLU, BMP1, RAB27B, MTUS1, KCNIP3, PMEPA1, LIPG, OPTN, UGCG, CHST15, F2RL1 | |  |  |  |  |  |  |  |  |  |  |  |  |  |  |  |  |  |  |  |  |  |  |
| Integral Component Of Lumenal Side Of Endoplasmic Reticulum Membrane | 3 | | 10 | | 9.61E-04 | 0.015 | | HLA-DRA, HLA-A, HLA-B | |  |  |  |  |  |  |  |  |  |  |  |  |  |  |  |  |  |  |  |  |  |  |
| Cell Junction | 20 | | 1 | | 1.03E-03 | 0.013 | | TNS3, ADGRE5, CDH11, ITGB3, BAIAP2L1, CPEB4, SCN5A, SORBS2, CDC42BPA, PTPRS, CASK, MAP3K1, HEG1, DSP, PDLIM1, LAMA3, LCP1, PARD6B, EFNB2, TGM2 | |  |  |  |  |  |  |  |  |  |  |  |  |  |  |  |  |  |  |  |  |  |  |
| Intracellular Organelle | 110 | | 0 | | 1.23E-03 | 0.008 | | ARRB1, ATP2B4, ABCA1, AK4, CLU, BMP1, CYP24A1, DPYSL2, DSP, CPT1A, CTSD, FLG, LPAR1, EYA4, F2RL1, ACSL1, FBP1, GPR1, HLA-A, HLA-B, GATA6, GBP1, GBP2, GBP3, ITGB3, HLA-DRA, MEF2C, MEIS3P1, MAP3K1, LAMA3, LCP1, LTBP1, PON2, RRBP1, SCN5A, SCP2, PTPRS, RAB27B, SOX4, SNAI2, UGCG, VEGFA, WT1, TGFA, TGFB2, THBS1, TGFBR3, TGM2, TIMP3, TLR3, SLC7A5, ZNF43, LAPTM5, EVI5, CLIC3, GPRC5A, SORBS2, CASK, PTGES, CYTIP, PDLIM1, LIPG, RAD50, OPTN, MBNL2, CTDSPL, NUAK1, PLK2, MGAT4A, PRSS23, TDRD7, ARL2BP, TRIB2, SNX10, KCNIP3, ASAP1, CHST15, SNX7, LPCAT2, RBM47, BAIAP2L1, KLHL4, ACOXL, ZMIZ1, CEMIP, MTUS1, PMEPA1, NXN, HHIP, SLAMF7, PORCN, CDK15, ELOVL7, CPEB4, MORC4, COLGALT1, CRISPLD2, PARD6B, TSPYL5, FBXO32, MARCH3, TRIM6, PRICKLE1, SAMD9L, SLC37A2, MAMDC2, ZNF391, ILDR2, SHISA2, SNORD123 | |  |  |  |  |  |  |  |  |  |  |  |  |  |  |  |  |  |  |  |  |  |  |
| Cell-Cell Adherens Junction | 5 | | 3 | | 1.70E-03 | 0.017 | | CDH11, BAIAP2L1, DSP, PDLIM1, LAMA3 | |  |  |  |  |  |  |  |  |  |  |  |  |  |  |  |  |  |  |  |  |  |  |
| Contractile Fiber Part | 7 | | 2 | | 1.79E-03 | 0.016 | | MEF2C, ATP2B4, PDLIM1, MYL9, FBXO32, SCN5A, SORBS2 | |  |  |  |  |  |  |  |  |  |  |  |  |  |  |  |  |  |  |  |  |  |  |
| Organelle Part | 95 | | 0 | | 1.81E-03 | 0.008 | | ARRB1, ATP2B4, ABCA1, AK4, ADGRE5, FBXO32, MARCH3, CLU, BMP4, CYP24A1, DPYSL2, DSP, CPT1A, CSF1, CTSD, FLG, F13A1, ACSL1, GPR1, HLA-A, HLA-B, GATA6, GBP1, GBP2, GBP3, PRICKLE1, IGFBP4, ITGB3, HLA-DRA, SH3RF2, MEF2C, MAP3K1, LCP1, COX7B2, LTBP1, SLC37A2, SCN5A, SCP2, PTPRS, RAB27B, SOX4, SDC2, SNAI2, UGCG, VEGFA, WT1, TGFA, TGFB2, THBS1, TIMP3, TLR3, SERINC2, ILDR2, SHISA2, LAPTM5, EVI5, CLIC3, GPRC5A, SORBS2, CDC42BPA, CASK, PTGES, CYTIP, PDLIM1, RAD50, OPTN, MBNL2, NUAK1, PLK2, MYL9, MGAT4A, PRSS23, TDRD7, ARL2BP, SNX10, ASAP1, CHST15, SNX7, LPCAT2, BAIAP2L1, KLHL4, SNORD123, ACOXL, CPPED1, SLC44A2, ZMIZ1, CEMIP, MTUS1, PMEPA1, HHIP, PORCN, ELOVL7, MORC4, COLGALT1, CRISPLD2 | |  |  |  |  |  |  |  |  |  |  |  |  |  |  |  |  |  |  |  |  |  |  |
| Focal Adhesion | 9 | | 2 | | 1.96E-03 | 0.016 | | PDLIM1, TNS3, LCP1, ADGRE5, ITGB3, SORBS2, EFNB2, TGM2, CASK | |  |  |  |  |  |  |  |  |  |  |  |  |  |  |  |  |  |  |  |  |  |  |
| Mhc Protein Complex | 3 | | 8 | | 1.96E-03 | 0.014 | | HLA-A, HLA-B, HLA-DRA | |  |  |  |  |  |  |  |  |  |  |  |  |  |  |  |  |  |  |  |  |  |  |
| Neuron Projection | 19 | | 1 | | 1.97E-03 | 0.013 | | GPR1, TNFRSF21, PLK2, ARRB1, ATP2B4, TENM3, CPEB4, CLU, SORBS2, PTPRS, CASK, DPYSL2, NOG, KCNIP3, LTBP1, ASAP1, OPTN, LPAR1, TGFB2 | |  |  |  |  |  |  |  |  |  |  |  |  |  |  |  |  |  |  |  |  |  |  |
| Cell-Substrate Adherens Junction | 9 | | 2 | | 2.13E-03 | 0.015 | | PDLIM1, TNS3, LCP1, ADGRE5, ITGB3, SORBS2, EFNB2, TGM2, CASK | |  |  |  |  |  |  |  |  |  |  |  |  |  |  |  |  |  |  |  |  |  |  |
| Intracellular Membrane-Bounded Organelle | 94 | | 0 | | 2.47E-03 | 0.008 | | ARRB1, ATP2B4, ABCA1, TSPYL5, AK4, FBXO32, MARCH3, CLU, BMP1, CYP24A1, DPYSL2, DSP, TRIM6, CPT1A, CTSD, FLG, EYA4, F2RL1, ACSL1, FBP1, GPR1, HLA-A, HLA-B, GATA6, GBP1, GBP2, GBP3, PRICKLE1, ITGB3, HLA-DRA, MEF2C, MEIS3P1, LAMA3, LTBP1, SLC37A2, PON2, MAMDC2, RRBP1, SCN5A, SCP2, RAB27B, SOX4, SNAI2, UGCG, VEGFA, WT1, TGFA, THBS1, TGFBR3, TGM2, TIMP3, TLR3, ZNF391, SLC7A5, ILDR2, SHISA2, ZNF43, LAPTM5, EVI5, CLIC3, GPRC5A, SORBS2, CASK, PTGES, LIPG, RAD50, OPTN, MBNL2, CTDSPL, NUAK1, MGAT4A, PRSS23, ARL2BP, TRIB2, SNX10, KCNIP3, CHST15, LPCAT2, RBM47, ACOXL, ZMIZ1, CEMIP, MTUS1, PMEPA1, NXN, HHIP, SLAMF7, PORCN, CDK15, ELOVL7, CPEB4, MORC4, COLGALT1, PARD6B | |  |  |  |  |  |  |  |  |  |  |  |  |  |  |  |  |  |  |  |  |  |  |
| Membrane Part | 81 | | 0 | | 2.68E-03 | 0.009 | | ARRB1, ATP2B4, ABCA1, CD22, ADGRE5, CDH11, MARCH3, DSP, CPT1A, OR5P2, CSF1, CTSD, LPAR1, EFNB2, LYPD6B, F2RL1, ACSL1, GPR1, TMEM56, HAS2, HLA-A, HLA-B, ITGB3, CD82, HLA-DRA, LCP1, COX7B2, MPZL3, PLPP4, SLC37A2, HECTD4, TMPRSS15, ADGRF1, PLXNA1, SCN5A, SCP2, SCN9A, PTPRS, RAB27B, SDC2, UGCG, TGFA, THBS1, TGFBR3, TGM2, TLR3, SERINC2, SLC7A5, ILDR2, SHISA2, LAPTM5, CLIC3, GPRC5A, SORBS2, CASK, TNFSF10, PTGES, FCGR2C, TSPAN2, MGAT4A, TNFRSF21, CDON, SNX10, KCNIP3, ASAP1, CLIC6, CHST15, LPCAT2, TENM3, SLC44A2, CEMIP, HEG1, SORCS2, PMEPA1, HHIP, SLAMF7, PORCN, ELOVL7, CPEB4, FAT4, PARD6B | |  |  |  |  |  |  |  |  |  |  |  |  |  |  |  |  |  |  |  |  |  |  |
| Coated Vesicle Membrane | 5 | | 3 | | 2.77E-03 | 0.016 | | CEMIP, HLA-A, HLA-B, HLA-DRA, TGFA | |  |  |  |  |  |  |  |  |  |  |  |  |  |  |  |  |  |  |  |  |  |  |
| Golgi-Associated Vesicle Membrane | 4 | | 4 | | 2.85E-03 | 0.015 | | HLA-A, HLA-B, HLA-DRA, TGFA | |  |  |  |  |  |  |  |  |  |  |  |  |  |  |  |  |  |  |  |  |  |  |
| Intrinsic Component Of Membrane | 68 | | 0 | | 2.91E-03 | 0.009 | | ATP2B4, ABCA1, CD22, ADGRE5, CDH11, MARCH3, CPT1A, OR5P2, CSF1, LPAR1, EFNB2, LYPD6B, F2RL1, ACSL1, GPR1, TMEM56, HAS2, HLA-A, HLA-B, ITGB3, CD82, HLA-DRA, COX7B2, MPZL3, PLPP4, SLC37A2, HECTD4, TMPRSS15, ADGRF1, PLXNA1, SCN5A, SCN9A, PTPRS, RAB27B, SDC2, UGCG, TGFA, TGFBR3, TGM2, TLR3, SERINC2, SLC7A5, ILDR2, SHISA2, LAPTM5, CLIC3, GPRC5A, CASK, TNFSF10, PTGES, FCGR2C, TSPAN2, MGAT4A, TNFRSF21, CDON, CLIC6, CHST15, LPCAT2, TENM3, SLC44A2, HEG1, SORCS2, PMEPA1, HHIP, SLAMF7, PORCN, ELOVL7, FAT4 | |  |  |  |  |  |  |  |  |  |  |  |  |  |  |  |  |  |  |  |  |  |  |
| Cell-Cell Junction | 10 | | 1 | | 3.02E-03 | 0.014 | | HEG1, DSP, PDLIM1, LAMA3, PARD6B, CDH11, BAIAP2L1, SCN5A, CDC42BPA, CASK | |  |  |  |  |  |  |  |  |  |  |  |  |  |  |  |  |  |  |  |  |  |  |
| Whole Membrane | 19 | | 1 | | 3.26E-03 | 0.012 | | ARRB1, ADGRE5, MARCH3, RAB27B, CYP24A1, SLC44A2, CEMIP, DSP, CPT1A, PMEPA1, TGFA, TLR3, ACSL1, HLA-A, HLA-B, LAPTM5, ITGB3, HLA-DRA, OPTN | |  |  |  |  |  |  |  |  |  |  |  |  |  |  |  |  |  |  |  |  |  |  |
| Mhc Class I Protein Complex | 2 | | 15 | | 3.34E-03 | 0.011 | | HLA-A, HLA-B | |  |  |  |  |  |  |  |  |  |  |  |  |  |  |  |  |  |  |  |  |  |  |
| Cell Projection | 26 | | 1 | | 3.47E-03 | 0.011 | | PLK2, ARRB1, ATP2B4, TENM3, CLU, SCN9A, PTPRS, ARL2BP, DPYSL2, TIAM2, LPAR1, TGFB2, F2RL1, GPR1, TNFRSF21, ITGB3, CPEB4, SORBS2, CDC42BPA, CASK, NOG, LCP1, KCNIP3, LTBP1, ASAP1, OPTN | |  |  |  |  |  |  |  |  |  |  |  |  |  |  |  |  |  |  |  |  |  |  |
| Organelle Membrane | 33 | | 1 | | 3.85E-03 | 0.010 | | ARRB1, MGAT4A, ADGRE5, MARCH3, CLU, CYP24A1, DSP, CPT1A, ACSL1, HLA-A, HLA-B, GATA6, GBP1, GBP2, GBP3, PRICKLE1, ITGB3, HLA-DRA, CHST15, SNX7, LPCAT2, RAB27B, SLC44A2, CEMIP, PMEPA1, UGCG, HHIP, TGFA, TLR3, LAPTM5, GPRC5A, CASK, OPTN | |  |  |  |  |  |  |  |  |  |  |  |  |  |  |  |  |  |  |  |  |  |  |
| Voltage-Gated Sodium Channel Complex | 2 | | 14 | | 3.88E-03 | 0.011 | | SCN5A, SCN9A | |  |  |  |  |  |  |  |  |  |  |  |  |  |  |  |  |  |  |  |  |  |  |
| Integral Component Of Membrane | 66 | | 0 | | 4.03E-03 | 0.009 | | ATP2B4, ABCA1, CD22, ADGRE5, CDH11, MARCH3, CPT1A, OR5P2, CSF1, LPAR1, EFNB2, LYPD6B, F2RL1, ACSL1, GPR1, TMEM56, HAS2, HLA-A, HLA-B, ITGB3, CD82, HLA-DRA, COX7B2, MPZL3, PLPP4, SLC37A2, HECTD4, TMPRSS15, ADGRF1, PLXNA1, SCN5A, SCN9A, PTPRS, SDC2, UGCG, TGFA, TGFBR3, TLR3, SERINC2, SLC7A5, ILDR2, SHISA2, LAPTM5, CLIC3, GPRC5A, CASK, TNFSF10, PTGES, FCGR2C, TSPAN2, MGAT4A, TNFRSF21, CDON, CLIC6, CHST15, LPCAT2, TENM3, SLC44A2, HEG1, SORCS2, PMEPA1, HHIP, SLAMF7, PORCN, ELOVL7, FAT4 | |  |  |  |  |  |  |  |  |  |  |  |  |  |  |  |  |  |  |  |  |  |  |
| Plasma Membrane Protein Complex | 11 | | 1 | | 4.10E-03 | 0.014 | | ARRB1, HLA-A, HLA-B, PORCN, CDH11, ITGB3, SCN5A, SCN9A, HLA-DRA, KCNIP3, TGFBR3 | |  | | |  | | | |  | | | |  | | | |  | | | |  | | |
| Intracellular Organelle Part | 91 | | 0 | | 4.67E-03 | 0.008 | | ARRB1, ABCA1, AK4, ADGRE5, FBXO32, MARCH3, CLU, BMP4, CYP24A1, DPYSL2, DSP, CPT1A, CSF1, CTSD, FLG, F13A1, ACSL1, GPR1, HLA-A, HLA-B, GATA6, GBP1, GBP2, GBP3, PRICKLE1, IGFBP4, ITGB3, HLA-DRA, SH3RF2, MEF2C, MAP3K1, LCP1, COX7B2, LTBP1, SLC37A2, SCP2, PTPRS, RAB27B, SOX4, SDC2, SNAI2, UGCG, VEGFA, WT1, TGFA, TGFB2, THBS1, TIMP3, TLR3, SERINC2, ILDR2, SHISA2, LAPTM5, EVI5, CLIC3, GPRC5A, SORBS2, CDC42BPA, CASK, PTGES, CYTIP, RAD50, OPTN, MBNL2, NUAK1, PLK2, MYL9, MGAT4A, PRSS23, TDRD7, ARL2BP, SNX10, ASAP1, CHST15, SNX7, LPCAT2, BAIAP2L1, KLHL4, SNORD123, ACOXL, CPPED1, SLC44A2, ZMIZ1, CEMIP, MTUS1, PMEPA1, PORCN, ELOVL7, MORC4, COLGALT1, CRISPLD2 | |  | | |  | | | |  | | | |  | | | |  | | | |  | | |
| Plasma Membrane Bounded Cell Projection | 24 | | 1 | | 4.90E-03 | 0.011 | | PLK2, ARRB1, ATP2B4, TENM3, CLU, PTPRS, ARL2BP, DPYSL2, TIAM2, LPAR1, TGFB2, F2RL1, GPR1, TNFRSF21, CPEB4, SORBS2, CDC42BPA, CASK, NOG, LCP1, KCNIP3, LTBP1, ASAP1, OPTN | |  | | |  | | | |  | | | |  | | | |  | | | |  | | |
| Cell Part | 156 | | 0 | | 4.90E-03 | 0.007 | | ARRB1, ATP2B4, ABCA1, AK4, CD22, ADGRE5, CDH11, CLU, BMP4, BMP1, C4BPB, CYP24A1, DPYSL2, DSP, CPT1A, CSF1, CTSD, FLG, LPAR1, EFNB2, EYA4, F2RL1, F13A1, ACSL1, EFEMP1, FBP1, GPR1, HAS2, HLA-A, HLA-B, GATA6, GBP1, GBP2, GBP3, IGFBP4, INHBB, ITGB3, CD82, HLA-DRA, MEF2C, MEIS3P1, MAP3K1, LAMA3, LCP1, LTBP1, PON2, TMPRSS15, PLXNA1, RRBP1, SCN5A, SCP2, SCN9A, PTPRS, RAB27B, SOX4, SDC2, SNAI2, TUFT1, UGCG, VEGFA, WT1, TGFA, TGFB2, THBS1, TGFBR3, TGM2, TIMP3, TLR3, SLC7A5, ZNF43, LAPTM5, EVI5, CLIC3, GPRC5A, SORBS2, CDC42BPA, CST7, CASK, TNFSF10, PTGES, CYTIP, FCGR2C, PDLIM1, NOG, LIPG, TSPAN2, RAD50, OPTN, MBNL2, CTDSPL, NUAK1, PLK2, PDE10A, MYL9, MGAT4A, PTGR1, PRSS23, TDRD7, ARL2BP, TIAM2, TNFRSF21, TRIB2, CDON, SNX10, KCNIP3, ASAP1, CLIC6, CHST15, SNX7, LPCAT2, RBM47, TENM3, BAIAP2L1, KLHL4, ACOXL, CPPED1, SLC44A2, ZMIZ1, CEMIP, HEG1, MTUS1, PMEPA1, NXN, HHIP, SLAMF7, TNS3, PORCN, CDK15, ELOVL7, CPEB4, FAT4, MORC4, COLGALT1, CRISPLD2, APOL6, PARD6B, AFAP1L2, TSPYL5, FBXO32, MARCH3, TRIM6, OR5P2, LYPD6B, PRICKLE1, SH3RF2, SAMD9L, COX7B2, PLPP4, SLC37A2, MAMDC2, ADGRF1, ZNF391, SERINC2, ILDR2, SHISA2, SNORD123 | |  | | |  | | | |  | | | |  | | | |  | | | |  | | |
| Pseudopodium | 2 | | 11 | | 5.73E-03 | 0.010 | | ARRB1, F2RL1 | |  | | |  | | | |  | | | |  | | | |  | | | |  | | |
| Neuron Part | 23 | | 1 | | 8.69E-03 | 0.011 | | PLK2, ARRB1, ATP2B4, PDE10A, TENM3, CLU, PTPRS, RAB27B, DPYSL2, SDC2, TIAM2, LPAR1, TGFB2, GPR1, TNFRSF21, CPEB4, SORBS2, CASK, NOG, KCNIP3, LTBP1, ASAP1, OPTN | |  | | |  | | | |  | | | |  | | | |  | | | |  | | |
| Actin Cytoskeleton | 6 | | 2 | | 9.40E-03 | 0.014 | | GBP1, GBP2, LCP1, BAIAP2L1, SORBS2, CASK | |  | | |  | | | |  | | | |  | | | |  | | | |  | | |
| Cytoskeleton | 19 | | 1 | | 9.58E-03 | 0.011 | | PLK2, BAIAP2L1, CLU, KLHL4, ARL2BP, DPYSL2, MTUS1, DSP, GBP1, GBP2, EVI5, TRIB2, SORBS2, CASK, MAP3K1, PDLIM1, SNX10, LCP1, NUAK1 | |  | | |  | | | |  | | | |  | | | |  | | | |  | | |
| Endosomal Part | 9 | | 1 | | 1.05E-02 | 0.013 | | HLA-A, HLA-B, SNX10, PMEPA1, OPTN, MARCH3, HLA-DRA, RAB27B, TLR3 | |  | | |  | | | |  | | | |  | | | |  | | | |  | | |
| Sodium Channel Complex | 2 | | 8 | | 1.13E-02 | 0.010 | | SCN5A, SCN9A | |  | | |  | | | |  | | | |  | | | |  | | | |  | | |
| Plasma Membrane Region | 16 | | 1 | | 1.21E-02 | 0.011 | | SLC7A5, ARRB1, ATP2B4, ITGB3, CPEB4, SCN5A, SORBS2, PTPRS, CASK, RAB27B, DSP, LCP1, ASAP1, PARD6B, HHIP, TGFA | |  | | |  | | | |  | | | |  | | | |  | | | |  | | |
| Integrin Alphav-Beta3 Complex | 1 | | 50 | | 1.34E-02 | 0.006 | | ITGB3 | |  | | |  | | | |  | | | |  | | | |  | | | |  | | |
| Apical Cytoplasm | 1 | | 50 | | 1.34E-02 | 0.006 | | SNX10 | |  | | |  | | | |  | | | |  | | | |  | | | |  | | |
| Alpha9-Beta1 Integrin-Adam8 Complex | 1 | | 50 | | 1.34E-02 | 0.006 | | ITGB3 | |  | | |  | | | |  | | | |  | | | |  | | | |  | | |
| Podocyte Foot | 1 | | 50 | | 1.34E-02 | 0.006 | | CASK | |  | | |  | | | |  | | | |  | | | |  | | | |  | | |
| Csf1-Csf1R Complex | 1 | | 50 | | 1.34E-02 | 0.006 | | CSF1 | |  | | |  | | | |  | | | |  | | | |  | | | |  | | |
| Inhibin B Complex | 1 | | 50 | | 1.34E-02 | 0.006 | | INHBB | |  | | |  | | | |  | | | |  | | | |  | | | |  | | |
| Axon | 8 | | 1 | | 1.29E-02 | 0.013 | | TNFRSF21, DPYSL2, NOG, KCNIP3, TENM3, OPTN, PTPRS, TGFB2 | |  | | |  | | | |  | | | |  | | | |  | | | |  | | |
| Endosome Membrane | 8 | | 1 | | 1.40E-02 | 0.013 | | HLA-A, HLA-B, PMEPA1, OPTN, MARCH3, HLA-DRA, RAB27B, TLR3 | |  | | |  | | | |  | | | |  | | | |  | | | |  | | |
| Apical Dendrite | 2 | | 7 | | 1.41E-02 | 0.010 | | CASK, CLU | |  | | |  | | | |  | | | |  | | | |  | | | |  | | |
| Intrinsic Component Of Synaptic Vesicle Membrane | 2 | | 6 | | 1.84E-02 | 0.010 | | PTPRS, RAB27B | |  | | |  | | | |  | | | |  | | | |  | | | |  | | |
| **Enrichment for Molecular Function** | | | | | | | | |  | |  | | |  |  | | |  |  | | |  |  | | |  |  | | |  |  |
| Ion Binding | 77 | | 1 | | 7.65E-06 | 0.011 | | ATP2B4, ABCA1, AK4, ADGRE5, CDH11, MARCH3, BMP4, BMP1, CYP24A1, TRIM6, FLG, LPAR1, EYA4, F13A1, ACSL1, EFEMP1, FBP1, GATA6, GBP1, GBP2, GBP3, PRICKLE1, SH3RF2, MAP3K1, LCP1, LTBP1, PON2, SCP2, SCN9A, PTPRS, RAB27B, SNAI2, VEGFA, WT1, THBS1, TGFBR3, TGM2, TIMP3, TLL1, ZNF391, ZNF43, CDC42BPA, CASK, TNFSF10, PTGES, PDLIM1, LIPG, RAD50, OPTN, MBNL2, CTDSPL, NUAK1, PLK2, PDE10A, MYL9, MGAT4A, TRIB2, CPA4, SNX10, KCNIP3, ASAP1, CHST15, LPCAT2, ACOXL, CPPED1, ZMIZ1, CEMIP, HEG1, ZNF608, HHIP, TNS3, CDK15, CPEB4, FAT4, MORC4, ADAMTS12, CRISPLD2 |  | | |  | | | |  | | | |  | | | |  | | | |  | | | |
| Fibroblast Growth Factor Binding | 4 | | 15 | | 3.57E-05 | 0.020 | | THBS1, TGFBR3, ITGB3, SCN5A |  | | |  | | | |  | | | |  | | | |  | | | |  | | | |
| Peptide Binding | 12 | | 3 | | 3.61E-05 | 0.021 | | GPR1, SLC7A5, PTGES, CEMIP, HLA-A, HLA-B, CTSD, ASAP1, ITGB3, CLU, HLA-DRA, TGFB2 |  | | |  | | | |  | | | |  | | | |  | | | |  | | | |
| Amide Binding | 13 | | 2 | | 4.61E-05 | 0.020 | | GPR1, SLC7A5, PTGES, CEMIP, HLA-A, HLA-B, CTSD, ASAP1, ITGB3, CLU, SCP2, HLA-DRA, TGFB2 |  | | |  | | | |  | | | |  | | | |  | | | |  | | | |
| Palmitoleoyltransferase Activity | 2 | | 100 | | 5.27E-05 | 0.011 | | PORCN, CPT1A |  | | |  | | | |  | | | |  | | | |  | | | |  | | | |
| Growth Factor Activity | 8 | | 4 | | 5.76E-05 | 0.023 | | INHBB, VEGFA, BMP4, BMP1, TGFA, TGFB2, CSF1, EFEMP1 |  | | |  | | | |  | | | |  | | | |  | | | |  | | | |
| Sulfur Compound Binding | 10 | | 3 | | 8.02E-05 | 0.021 | | PTGES, CRISPLD2, LIPG, VEGFA, SCP2, BMP4, PTPRS, CHST15, THBS1, TGFBR3 |  | | |  | | | |  | | | |  | | | |  | | | |  | | | |
| Glycosaminoglycan Binding | 9 | | 3 | | 1.11E-04 | 0.021 | | CEMIP, CRISPLD2, MAMDC2, LIPG, VEGFA, BMP4, PTPRS, THBS1, TGFBR3 |  | | |  | | | |  | | | |  | | | |  | | | |  | | | |
| Metal Ion Binding | 54 | | 1 | | 1.20E-04 | 0.011 | | ATP2B4, PDE10A, MYL9, MGAT4A, ADGRE5, CDH11, MARCH3, BMP1, CYP24A1, TRIM6, FLG, EYA4, F13A1, EFEMP1, FBP1, GATA6, PRICKLE1, SH3RF2, MAP3K1, CPA4, LCP1, KCNIP3, LTBP1, ASAP1, PON2, LPCAT2, SCN9A, CPPED1, ZMIZ1, HEG1, ZNF608, SNAI2, WT1, HHIP, THBS1, TGM2, TIMP3, TLL1, ZNF391, ZNF43, TNS3, CDK15, CPEB4, FAT4, CDC42BPA, MORC4, TNFSF10, ADAMTS12, PDLIM1, RAD50, OPTN, MBNL2, CTDSPL, NUAK1 |  | | |  | | | |  | | | |  | | | |  | | | |  | | | |
| Intracellular Vesicle | 27 | | 1 | | 1.32E-04 | 0.013 | | ARRB1, ABCA1, MARCH3, CLU, PTPRS, RAB27B, CEMIP, PMEPA1, CTSD, VEGFA, LPAR1, TGFA, TGFB2, THBS1, TLR3, F2RL1, GBP1, GBP2, GBP3, ITGB3, CYTIP, CRISPLD2, SAMD9L, SNX10, LIPG, OPTN, SNX7 |  | | |  | | | |  | | | |  | | | |  | | | |  | | | |
| Anchoring Junction | 13 | | 2 | | 1.44E-04 | 0.018 | | DSP, PDLIM1, TNS3, LAMA3, LCP1, ADGRE5, CDH11, ITGB3, BAIAP2L1, SORBS2, EFNB2, TGM2, CASK |  | | |  | | | |  | | | |  | | | |  | | | |  | | | |
| Growth Factor Binding | 7 | | 4 | | 1.81E-04 | 0.021 | | IGFBP4, VEGFA, ITGB3, SCN5A, THBS1, TGFBR3, LTBP1 |  | | |  | | | |  | | | |  | | | |  | | | |  | | | |
| Cation Binding | 54 | | 1 | | 2.36E-04 | 0.011 | | ATP2B4, PDE10A, MYL9, MGAT4A, ADGRE5, CDH11, MARCH3, BMP1, CYP24A1, TRIM6, FLG, EYA4, F13A1, EFEMP1, FBP1, GATA6, PRICKLE1, SH3RF2, MAP3K1, CPA4, LCP1, KCNIP3, LTBP1, ASAP1, PON2, LPCAT2, SCN9A, CPPED1, ZMIZ1, HEG1, ZNF608, SNAI2, WT1, HHIP, THBS1, TGM2, TIMP3, TLL1, ZNF391, ZNF43, TNS3, CDK15, CPEB4, FAT4, CDC42BPA, MORC4, TNFSF10, ADAMTS12, PDLIM1, RAD50, OPTN, MBNL2, CTDSPL, NUAK1 |  | | |  | | | |  | | | |  | | | |  | | | |  | | | |
| Gmp Binding | 2 | | 50 | | 3.13E-04 | 0.011 | | GBP1, GBP3 |  | | |  | | | |  | | | |  | | | |  | | | |  | | | |
| Protein Binding | 78 | | 0 | | 4.16E-04 | 0.010 | | ARRB1, ATP2B4, ABCA1, ADGRE5, CDH11, CLU, BMP4, BMP1, DPYSL2, DSP, TRIM6, CSF1, LPAR1, EFNB2, EYA4, F2RL1, EFEMP1, HLA-A, HLA-B, GATA6, GBP1, GBP2, GBP3, PRICKLE1, IGFBP4, INHBB, ITGB3, SH3RF2, MEF2C, MAP3K1, LAMA3, LCP1, LTBP1, SCN5A, SCP2, PTPRS, RAB27B, SOX4, SDC2, VEGFA, WT1, TGFA, TGFB2, THBS1, TGFBR3, TGM2, TIMP3, SERINC2, EVI5, GPRC5A, SORBS2, CASK, TNFSF10, PDLIM1, NOG, RAD50, OPTN, NUAK1, PLK2, MYL9, TDRD7, TIAM2, TRIB2, SNX10, KCNIP3, ASAP1, CLIC6, TENM3, BAIAP2L1, KLHL4, CEMIP, MTUS1, PMEPA1, HHIP, PORCN, CDK15, PARD6B, AFAP1L2 |  | | |  | | | |  | | | |  | | | |  | | | |  | | | |
| Heparin Binding | 7 | | 3 | | 4.78E-04 | 0.019 | | VEGFA, CRISPLD2, BMP4, PTPRS, THBS1, TGFBR3, LIPG |  | | |  | | | |  | | | |  | | | |  | | | |  | | | |
| Fibrinogen Binding | 2 | | 40 | | 5.19E-04 | 0.011 | | THBS1, ITGB3 |  | | |  | | | |  | | | |  | | | |  | | | |  | | | |
| Cytokine Binding | 6 | | 4 | | 5.68E-04 | 0.019 | | ITGB3, GBP1, THBS1, TGFBR3, NOG, LTBP1 |  | | |  | | | |  | | | |  | | | |  | | | |  | | | |
| Transforming Growth Factor Beta Binding | 3 | | 13 | | 6.02E-04 | 0.015 | | THBS1, TGFBR3, LTBP1 |  |  |  |  |  |  |  |  |  |  |  |  |  |  |  |  |  |  |  |  |  |  |  |
| Calcium Ion Binding | 15 | | 1 | | 6.66E-04 | 0.016 | | LPCAT2, MYL9, ADGRE5, CDH11, FAT4, BMP1, HEG1, LCP1, KCNIP3, LTBP1, FLG, THBS1, TGM2, TLL1, EFEMP1 |  |  |  |  |  |  |  |  |  |  |  |  |  |  |  |  |  |  |  |  |  |  |  |
| Molecular Adaptor Activity | 8 | | 3 | | 7.02E-04 | 0.019 | | ARRB1, DSP, TRIM6, RAD50, AFAP1L2, OPTN, SORBS2, SERINC2 |  |  |  |  |  |  |  |  |  |  |  |  |  |  |  |  |  |  |  |  |  |  |  |
| Transforming Growth Factor Beta Receptor Binding | 4 | | 7 | | 7.91E-04 | 0.017 | | INHBB, BMP4, TGFB2, TGFBR3 |  |  |  |  |  |  |  |  |  |  |  |  |  |  |  |  |  |  |  |  |  |  |  |
| Protein Binding, Bridging | 7 | | 3 | | 7.96E-04 | 0.019 | | RAD50, OPTN, ARRB1, DSP, SORBS2, TRIM6, SERINC2 |  |  |  |  |  |  |  |  |  |  |  |  |  |  |  |  |  |  |  |  |  |  |  |
| Nucleoside Monophosphate Kinase Activity | 3 | | 11 | | 8.70E-04 | 0.015 | | RAD50, CASK, AK4 |  |  |  |  |  |  |  |  |  |  |  |  |  |  |  |  |  |  |  |  |  |  |  |
| Peptide Antigen Binding | 4 | | 6 | | 1.37E-03 | 0.017 | | SLC7A5, HLA-A, HLA-B, HLA-DRA |  |  |  |  |  |  |  |  |  |  |  |  |  |  |  |  |  |  |  |  |  |  |  |
| Vascular Endothelial Growth Factor Receptor 2 Binding | 2 | | 25 | | 1.43E-03 | 0.011 | | VEGFA, ITGB3 |  |  |  |  |  |  |  |  |  |  |  |  |  |  |  |  |  |  |  |  |  |  |  |
| Transferase Activity, Transferring Acyl Groups | 8 | | 2 | | 1.60E-03 | 0.017 | | LPCAT2, ARRB1, PORCN, CPT1A, ELOVL7, SCP2, TGM2, F13A1 |  |  |  |  |  |  |  |  |  |  |  |  |  |  |  |  |  |  |  |  |  |  |  |
| Protein-Glutamine Gamma-Glutamyltransferase Activity | 2 | | 22 | | 1.83E-03 | 0.011 | | TGM2, F13A1 |  |  |  |  |  |  |  |  |  |  |  |  |  |  |  |  |  |  |  |  |  |  |  |
| Adenylate Kinase Activity | 2 | | 22 | | 1.83E-03 | 0.011 | | RAD50, AK4 |  |  |  |  |  |  |  |  |  |  |  |  |  |  |  |  |  |  |  |  |  |  |  |
| Type Ii Transforming Growth Factor Beta Receptor Binding | 2 | | 22 | | 1.83E-03 | 0.011 | | TGFB2, TGFBR3 |  |  |  |  |  |  |  |  |  |  |  |  |  |  |  |  |  |  |  |  |  |  |  |
| Carbohydrate Derivative Binding | 31 | | 1 | | 1.90E-03 | 0.012 | | PLK2, ATP2B4, PDE10A, ABCA1, MAMDC2, AK4, SCP2, BMP4, PTPRS, RAB27B, CEMIP, VEGFA, LPAR1, THBS1, TGFBR3, TGM2, ACSL1, FBP1, GBP1, GBP2, GBP3, CDK15, TRIB2, CDC42BPA, CASK, MAP3K1, CRISPLD2, LIPG, RAD50, CHST15, NUAK1 |  |  |  |  |  |  |  |  |  |  |  |  |  |  |  |  |  |  |  |  |  |  |  |
| Cell-Cell Adhesion Mediator Activity | 3 | | 8 | | 1.92E-03 | 0.014 | | PDLIM1, BAIAP2L1, DSP |  |  |  |  |  |  |  |  |  |  |  |  |  |  |  |  |  |  |  |  |  |  |  |
| Pdz Domain Binding | 5 | | 3 | | 2.31E-03 | 0.017 | | ATP2B4, LPAR1, SDC2, TGFBR3, CASK |  |  |  |  |  |  |  |  |  |  |  |  |  |  |  |  |  |  |  |  |  |  |  |
| Fibronectin Binding | 3 | | 8 | | 2.45E-03 | 0.014 | | THBS1, VEGFA, ITGB3 |  |  |  |  |  |  |  |  |  |  |  |  |  |  |  |  |  |  |  |  |  |  |  |
| Cytokine Activity | 7 | | 2 | | 3.20E-03 | 0.016 | | CSF1, INHBB, VEGFA, BMP4, BMP1, TGFB2, TNFSF10 |  |  |  |  |  |  |  |  |  |  |  |  |  |  |  |  |  |  |  |  |  |  |  |
| Insulin-Like Growth Factor I Binding | 2 | | 16 | | 3.31E-03 | 0.011 | | IGFBP4, ITGB3 |  |  |  |  |  |  |  |  |  |  |  |  |  |  |  |  |  |  |  |  |  |  |  |
| Binding | 127 | | 0 | | 3.42E-03 | 0.008 | | ARRB1, ATP2B4, ABCA1, AK4, CD22, ADGRE5, CDH11, CLU, BMP4, BMP1, CYP24A1, DPYSL2, DSP, CSF1, CTSD, FLG, LPAR1, EFNB2, EYA4, F2RL1, F13A1, ACSL1, EFEMP1, FBP1, GPR1, HLA-A, HLA-B, GATA6, GBP1, GBP2, GBP3, IGFBP4, INHBB, ITGB3, HLA-DRA, MEF2C, MEIS3P1, MAP3K1, LAMA3, LCP1, LTBP1, PON2, RRBP1, SCN5A, SCP2, SCN9A, PTPRS, RAB27B, SOX4, SDC2, SNAI2, VEGFA, WT1, TGFA, TGFB2, THBS1, TGFBR3, TGM2, TIMP3, TLL1, TLR3, SLC7A5, ZNF43, EVI5, GPRC5A, SORBS2, CDC42BPA, CASK, TNFSF10, PTGES, FCGR2C, PDLIM1, NOG, LIPG, RAD50, OPTN, MBNL2, CTDSPL, NUAK1, PLK2, PDE10A, MYL9, MGAT4A, TDRD7, TIAM2, TRIB2, CPA4, SNX10, KCNIP3, ASAP1, CLIC6, CHST15, SNX7, LPCAT2, RBM47, TENM3, BAIAP2L1, KLHL4, ACOXL, CPPED1, ZMIZ1, CEMIP, HEG1, MTUS1, ZNF608, PMEPA1, HHIP, TNS3, PORCN, CDK15, CPEB4, FAT4, MORC4, ADAMTS12, CRISPLD2, APOL6, PARD6B, AFAP1L2, MARCH3, TRIM6, OR5P2, PRICKLE1, SH3RF2, MAMDC2, ZNF391, SERINC2, MIR181B1 |  |  |  |  |  |  |  |  |  |  |  |  |  |  |  |  |  |  |  |  |  |  |  |
| Phosphatidylcholine Transporter Activity | 2 | | 15 | | 3.90E-03 | 0.011 | | ABCA1, SCP2 |  |  |  |  |  |  |  |  |  |  |  |  |  |  |  |  |  |  |  |  |  |  |  |
| Cell Adhesion Mediator Activity | 3 | | 6 | | 4.03E-03 | 0.014 | | PDLIM1, BAIAP2L1, DSP |  |  |  |  |  |  |  |  |  |  |  |  |  |  |  |  |  |  |  |  |  |  |  |
| Phosphotransferase Activity, Phosphate Group As Acceptor | 3 | | 6 | | 4.03E-03 | 0.014 | | RAD50, CASK, AK4 |  |  |  |  |  |  |  |  |  |  |  |  |  |  |  |  |  |  |  |  |  |  |  |
| Vascular Endothelial Growth Factor Receptor Binding | 2 | | 14 | | 4.53E-03 | 0.011 | | VEGFA, ITGB3 |  |  |  |  |  |  |  |  |  |  |  |  |  |  |  |  |  |  |  |  |  |  |  |
| Protein Domain Specific Binding | 14 | | 1 | | 5.46E-03 | 0.013 | | ATP2B4, SCN5A, SORBS2, CASK, RAB27B, MEF2C, SDC2, PMEPA1, ASAP1, AFAP1L2, WT1, LPAR1, TGFBR3, TGM2 |  |  |  |  |  |  |  |  |  |  |  |  |  |  |  |  |  |  |  |  |  |  |  |
| Coreceptor Activity | 3 | | 5 | | 5.78E-03 | 0.013 | | CD22, ITGB3, TGFBR3 |  |  |  |  |  |  |  |  |  |  |  |  |  |  |  |  |  |  |  |  |  |  |  |
| Nucleobase-Containing Compound Kinase Activity | 3 | | 5 | | 6.11E-03 | 0.013 | | RAD50, CASK, AK4 |  |  |  |  |  |  |  |  |  |  |  |  |  |  |  |  |  |  |  |  |  |  |  |
| Platelet-Derived Growth Factor Receptor Binding | 2 | | 11 | | 6.67E-03 | 0.010 | | VEGFA, ITGB3 |  |  |  |  |  |  |  |  |  |  |  |  |  |  |  |  |  |  |  |  |  |  |  |
| Transferase Activity, Transferring Amino-Acyl Groups | 2 | | 11 | | 6.67E-03 | 0.010 | | TGM2, F13A1 |  |  |  |  |  |  |  |  |  |  |  |  |  |  |  |  |  |  |  |  |  |  |  |
| Transferase Activity | 31 | | 1 | | 7.31E-03 | 0.011 | | LPCAT2, PLK2, ARRB1, AK4, MGAT4A, FBXO32, MARCH3, SCP2, TRIM6, CPT1A, UGCG, EFNB2, TGFBR3, TGM2, F13A1, EFEMP1, HAS2, PORCN, CDK15, ELOVL7, TRIB2, CDC42BPA, COLGALT1, CASK, SH3RF2, PTGES, MAP3K1, LTBP1, RAD50, CHST15, NUAK1 |  |  |  |  |  |  |  |  |  |  |  |  |  |  |  |  |  |  |  |  |  |  |  |
| Zinc Ion Binding | 14 | | 1 | | 7.37E-03 | 0.013 | | GATA6, PRICKLE1, MARCH3, BMP1, MORC4, TNFSF10, ZMIZ1, MAP3K1, CPA4, TRIM6, WT1, HHIP, TIMP3, TLL1 |  |  |  |  |  |  |  |  |  |  |  |  |  |  |  |  |  |  |  |  |  |  |  |
| Transforming Growth Factor Beta-Activated Receptor Activity | 2 | | 11 | | 7.47E-03 | 0.010 | | TGFBR3, LTBP1 |  |  |  |  |  |  |  |  |  |  |  |  |  |  |  |  |  |  |  |  |  |  |  |
| Cell Adhesion Molecule Binding | 10 | | 1 | | 8.02E-03 | 0.014 | | DSP, PDLIM1, LCP1, ASAP1, TENM3, CDH11, ITGB3, BAIAP2L1, GPRC5A, THBS1 |  |  |  |  |  |  |  |  |  |  |  |  |  |  |  |  |  |  |  |  |  |  |  |
| G-Protein Alpha-Subunit Binding | 3 | | 5 | | 8.31E-03 | 0.013 | | ADGRE5, LPAR1, F2RL1 |  |  |  |  |  |  |  |  |  |  |  |  |  |  |  |  |  |  |  |  |  |  |  |
| Cytokine Receptor Binding | 8 | | 2 | | 8.48E-03 | 0.014 | | CSF1, INHBB, VEGFA, ITGB3, BMP4, TGFB2, TGFBR3, TNFSF10 |  |  |  |  |  |  |  |  |  |  |  |  |  |  |  |  |  |  |  |  |  |  |  |
| Catalytic Activity | 65 | | 0 | | 9.13E-03 | 0.009 | | ARRB1, ATP2B4, ABCA1, AK4, FBXO32, MARCH3, CLU, BMP1, CYP24A1, DPYSL2, TRIM6, CPT1A, CTSD, EFNB2, EYA4, F13A1, ACSL1, EFEMP1, FBP1, HAS2, GBP1, GBP2, GBP3, ITGB3, SH3RF2, MAP3K1, COX7B2, LTBP1, PLPP4, PON2, TMPRSS15, SCP2, PTPRS, RAB27B, UGCG, TGFBR3, TGM2, TLL1, CDC42BPA, CASK, PTGES, LIPG, RAD50, CTDSPL, NUAK1, PLK2, PDE10A, MGAT4A, PTGR1, PRSS23, TRIB2, CPA4, CHST15, LPCAT2, TENM3, ACOXL, CPPED1, CEMIP, NXN, HHIP, PORCN, CDK15, ELOVL7, COLGALT1, ADAMTS12 |  |  |  |  |  |  |  |  |  |  |  |  |  |  |  |  |  |  |  |  |  |  |  |
| Voltage-Gated Sodium Channel Activity | 2 | | 10 | | 9.19E-03 | 0.010 | | SCN5A, SCN9A |  |  |  |  |  |  |  |  |  |  |  |  |  |  |  |  |  |  |  |  |  |  |  |
| Cadherin Binding Involved In Cell-Cell Adhesion | 2 | | 10 | | 9.19E-03 | 0.010 | | PDLIM1, BAIAP2L1 |  |  |  |  |  |  |  |  |  |  |  |  |  |  |  |  |  |  |  |  |  |  |  |
| Anion Binding | 34 | | 1 | | 1.01E-02 | 0.010 | | PLK2, ATP2B4, PDE10A, ABCA1, AK4, BMP4, LPAR1, ACSL1, FBP1, GBP1, GBP2, GBP3, TRIB2, MAP3K1, SNX10, ASAP1, CHST15, SCP2, ACOXL, PTPRS, RAB27B, CEMIP, VEGFA, THBS1, TGFBR3, TGM2, CDK15, CDC42BPA, CASK, PTGES, CRISPLD2, LIPG, RAD50, NUAK1 |  |  |  |  |  |  |  |  |  |  |  |  |  |  |  |  |  |  |  |  |  |  |  |
| Cholesterol Transporter Activity | 2 | | 9 | | 1.01E-02 | 0.010 | | ABCA1, SCP2 |  |  |  |  |  |  |  |  |  |  |  |  |  |  |  |  |  |  |  |  |  |  |  |
| Transferase Activity, Transferring Acyl Groups Other Than Amino-Acyl Groups | 6 | | 2 | | 1.19E-02 | 0.014 | | LPCAT2, ARRB1, PORCN, CPT1A, ELOVL7, SCP2 |  |  |  |  |  |  |  |  |  |  |  |  |  |  |  |  |  |  |  |  |  |  |  |
| Extracellular Matrix Binding | 3 | | 4 | | 1.24E-02 | 0.013 | | VEGFA, ITGB3, THBS1 |  |  |  |  |  |  |  |  |  |  |  |  |  |  |  |  |  |  |  |  |  |  |  |
| Transmembrane Receptor Protein Serine/Threonine Kinase Activity | 2 | | 8 | | 1.31E-02 | 0.010 | | TGFBR3, LTBP1 |  |  |  |  |  |  |  |  |  |  |  |  |  |  |  |  |  |  |  |  |  |  |  |
| Nitric-Oxide Synthase Binding | 2 | | 8 | | 1.31E-02 | 0.010 | | ATP2B4, SCN5A |  |  |  |  |  |  |  |  |  |  |  |  |  |  |  |  |  |  |  |  |  |  |  |
| Phosphatidylserine-Translocating Atpase Activity | 1 | | 50 | | 1.45E-02 | 0.006 | | ABCA1 |  |  |  |  |  |  |  |  |  |  |  |  |  |  |  |  |  |  |  |  |  |  |  |
| Dihydropyrimidinase Activity | 1 | | 50 | | 1.45E-02 | 0.006 | | DPYSL2 |  |  |  |  |  |  |  |  |  |  |  |  |  |  |  |  |  |  |  |  |  |  |  |
| Apolipoprotein A-I Receptor Activity | 1 | | 50 | | 1.45E-02 | 0.006 | | ABCA1 |  |  |  |  |  |  |  |  |  |  |  |  |  |  |  |  |  |  |  |  |  |  |  |
| Microfibril Binding | 1 | | 50 | | 1.45E-02 | 0.006 | | LTBP1 |  |  |  |  |  |  |  |  |  |  |  |  |  |  |  |  |  |  |  |  |  |  |  |
| Vascular Endothelial Growth Factor Receptor 1 Binding | 1 | | 50 | | 1.45E-02 | 0.006 | | VEGFA |  |  |  |  |  |  |  |  |  |  |  |  |  |  |  |  |  |  |  |  |  |  |  |
| Ceramide Glucosyltransferase Activity | 1 | | 50 | | 1.45E-02 | 0.006 | | UGCG |  |  |  |  |  |  |  |  |  |  |  |  |  |  |  |  |  |  |  |  |  |  |  |
| N-Acetylgalactosamine 4-Sulfate 6-O-Sulfotransferase Activity | 1 | | 50 | | 1.45E-02 | 0.006 | | CHST15 |  |  |  |  |  |  |  |  |  |  |  |  |  |  |  |  |  |  |  |  |  |  |  |
| Voltage-Gated Sodium Channel Activity Involved In Purkinje Myocyte Action Potential | 1 | | 50 | | 1.45E-02 | 0.006 | | SCN5A |  |  |  |  |  |  |  |  |  |  |  |  |  |  |  |  |  |  |  |  |  |  |  |
| Nucleoside Triphosphate Adenylate Kinase Activity | 1 | | 50 | | 1.45E-02 | 0.006 | | AK4 |  |  |  |  |  |  |  |  |  |  |  |  |  |  |  |  |  |  |  |  |  |  |  |
| Alpha-1A Adrenergic Receptor Binding | 1 | | 50 | | 1.45E-02 | 0.006 | | ARRB1 |  |  |  |  |  |  |  |  |  |  |  |  |  |  |  |  |  |  |  |  |  |  |  |
| D4 Dopamine Receptor Binding | 1 | | 50 | | 1.45E-02 | 0.006 | | CLIC6 |  |  |  |  |  |  |  |  |  |  |  |  |  |  |  |  |  |  |  |  |  |  |  |
| V2 Vasopressin Receptor Binding | 1 | | 50 | | 1.45E-02 | 0.006 | | ARRB1 |  |  |  |  |  |  |  |  |  |  |  |  |  |  |  |  |  |  |  |  |  |  |  |
| Fructose 1,6-Bisphosphate 1-Phosphatase Activity | 1 | | 50 | | 1.45E-02 | 0.006 | | FBP1 |  |  |  |  |  |  |  |  |  |  |  |  |  |  |  |  |  |  |  |  |  |  |  |
| Protein Kinase C Binding | 3 | | 4 | | 1.45E-02 | 0.012 | | PARD6B, ITGB3, DSP |  |  |  |  |  |  |  |  |  |  |  |  |  |  |  |  |  |  |  |  |  |  |  |
| Signaling Receptor Binding | 25 | | 1 | | 1.67E-02 | 0.011 | | ARRB1, ABCA1, CLU, SCP2, BMP4, BMP1, CSF1, VEGFA, EFNB2, TGFA, TGFB2, THBS1, TGFBR3, F2RL1, EFEMP1, HLA-A, HLA-B, IGFBP4, INHBB, ITGB3, CASK, TNFSF10, LAMA3, LCP1, CLIC6 |  |  |  |  |  |  |  |  |  |  |  |  |  |  |  |  |  |  |  |  |  |  |  |
| Voltage-Gated Ion Channel Activity | 5 | | 2 | | 1.69E-02 | 0.013 | | CLIC6, CLIC3, SCN5A, SCN9A, KCNIP3 |  |  |  |  |  |  |  |  |  |  |  |  |  |  |  |  |  |  |  |  |  |  |  |
| Voltage-Gated Channel Activity | 5 | | 2 | | 1.69E-02 | 0.013 | | CLIC6, CLIC3, SCN5A, SCN9A, KCNIP3 |  |  |  |  |  |  |  |  |  |  |  |  |  |  |  |  |  |  |  |  |  |  |  |
| Insulin-Like Growth Factor Binding | 2 | | 7 | | 1.76E-02 | 0.010 | | IGFBP4, ITGB3 |  |  |  |  |  |  |  |  |  |  |  |  |  |  |  |  |  |  |  |  |  |  |  |
| Mitogen-Activated Protein Kinase Kinase Binding | 3 | | 3 | | 1.81E-02 | 0.012 | | ARRB1, MAP3K1, TRIB2 |  |  |  |  |  |  |  |  |  |  |  |  |  |  |  |  |  |  |  |  |  |  |  |
| Ion Binding | 77 | | 1 | | 7.65E-06 | 0.011 | | ATP2B4, ABCA1, AK4, ADGRE5, CDH11, MARCH3, BMP4, BMP1, CYP24A1, TRIM6, FLG, LPAR1, EYA4, F13A1, ACSL1, EFEMP1, FBP1, GATA6, GBP1, GBP2, GBP3, PRICKLE1, SH3RF2, MAP3K1, LCP1, LTBP1, PON2, SCP2, SCN9A, PTPRS, RAB27B, SNAI2, VEGFA, WT1, THBS1, TGFBR3, TGM2, TIMP3, TLL1, ZNF391, ZNF43, CDC42BPA, CASK, TNFSF10, PTGES, PDLIM1, LIPG, RAD50, OPTN, MBNL2, CTDSPL, NUAK1, PLK2, PDE10A, MYL9, MGAT4A, TRIB2, CPA4, SNX10, KCNIP3, ASAP1, CHST15, LPCAT2, ACOXL, CPPED1, ZMIZ1, CEMIP, HEG1, ZNF608, HHIP, TNS3, CDK15, CPEB4, FAT4, MORC4, ADAMTS12, CRISPLD2 |  |  |  |  |  |  |  |  |  |  |  |  |  |  |  |  |  |  |  |  |  |  |  |
| Fibroblast Growth Factor Binding | 4 | | 15 | | 3.57E-05 | 0.020 | | THBS1, TGFBR3, ITGB3, SCN5A |  |  |  |  |  |  |  |  |  |  |  |  |  |  |  |  |  |  |  |  |  |  |  |
| Peptide Binding | 12 | | 3 | | 3.61E-05 | 0.021 | | GPR1, SLC7A5, PTGES, CEMIP, HLA-A, HLA-B, CTSD, ASAP1, ITGB3, CLU, HLA-DRA, TGFB2 |  |  |  |  |  |  |  |  |  |  |  |  |  |  |  |  |  |  |  |  |  |  |  |
| Amide Binding | 13 | | 2 | | 4.61E-05 | 0.020 | | GPR1, SLC7A5, PTGES, CEMIP, HLA-A, HLA-B, CTSD, ASAP1, ITGB3, CLU, SCP2, HLA-DRA, TGFB2 |  |  |  |  |  |  |  |  |  |  |  |  |  |  |  |  |  |  |  |  |  |  |  |
| Palmitoleoyltransferase Activity | 2 | | 100 | | 5.27E-05 | 0.011 | | PORCN, CPT1A |  |  |  |  |  |  |  |  |  |  |  |  |  |  |  |  |  |  |  |  |  |  |  |
| Growth Factor Activity | 8 | | 4 | | 5.76E-05 | 0.023 | | INHBB, VEGFA, BMP4, BMP1, TGFA, TGFB2, CSF1, EFEMP1 |  |  |  |  |  |  |  |  |  |  |  |  |  |  |  |  |  |  |  |  |  |  |  |
| Sulfur Compound Binding | 10 | | 3 | | 8.02E-05 | 0.021 | | PTGES, CRISPLD2, LIPG, VEGFA, SCP2, BMP4, PTPRS, CHST15, THBS1, TGFBR3 |  |  |  |  |  |  |  |  |  |  |  |  |  |  |  |  |  |  |  |  |  |  |  |
| Glycosaminoglycan Binding | 9 | | 3 | | 1.11E-04 | 0.021 | | CEMIP, CRISPLD2, MAMDC2, LIPG, VEGFA, BMP4, PTPRS, THBS1, TGFBR3 |  |  |  |  |  |  |  |  |  |  |  |  |  |  |  |  |  |  |  |  |  |  |  |
| Metal Ion Binding | 54 | | 1 | | 1.20E-04 | 0.011 | | ATP2B4, PDE10A, MYL9, MGAT4A, ADGRE5, CDH11, MARCH3, BMP1, CYP24A1, TRIM6, FLG, EYA4, F13A1, EFEMP1, FBP1, GATA6, PRICKLE1, SH3RF2, MAP3K1, CPA4, LCP1, KCNIP3, LTBP1, ASAP1, PON2, LPCAT2, SCN9A, CPPED1, ZMIZ1, HEG1, ZNF608, SNAI2, WT1, HHIP, THBS1, TGM2, TIMP3, TLL1, ZNF391, ZNF43, TNS3, CDK15, CPEB4, FAT4, CDC42BPA, MORC4, TNFSF10, ADAMTS12, PDLIM1, RAD50, OPTN, MBNL2, CTDSPL, NUAK1 |  |  |  |  |  |  |  |  |  |  |  |  |  |  |  |  |  |  |  |  |  |  |  |
| Intracellular Vesicle | 27 | | 1 | | 1.32E-04 | 0.013 | | ARRB1, ABCA1, MARCH3, CLU, PTPRS, RAB27B, CEMIP, PMEPA1, CTSD, VEGFA, LPAR1, TGFA, TGFB2, THBS1, TLR3, F2RL1, GBP1, GBP2, GBP3, ITGB3, CYTIP, CRISPLD2, SAMD9L, SNX10, LIPG, OPTN, SNX7 |  |  |  |  |  |  |  |  |  |  |  |  |  |  |  |  |  |  |  |  |  |  |  |
| Anchoring Junction | 13 | | 2 | | 1.44E-04 | 0.018 | | DSP, PDLIM1, TNS3, LAMA3, LCP1, ADGRE5, CDH11, ITGB3, BAIAP2L1, SORBS2, EFNB2, TGM2, CASK |  |  |  |  |  |  |  |  |  |  |  |  |  |  |  |  |  |  |  |  |  |  |  |
| Growth Factor Binding | 7 | | 4 | | 1.81E-04 | 0.021 | | IGFBP4, VEGFA, ITGB3, SCN5A, THBS1, TGFBR3, LTBP1 |  |  |  |  |  |  |  |  |  |  |  |  |  |  |  |  |  |  |  |  |  |  |  |
| Cation Binding | 54 | | 1 | | 2.36E-04 | 0.011 | | ATP2B4, PDE10A, MYL9, MGAT4A, ADGRE5, CDH11, MARCH3, BMP1, CYP24A1, TRIM6, FLG, EYA4, F13A1, EFEMP1, FBP1, GATA6, PRICKLE1, SH3RF2, MAP3K1, CPA4, LCP1, KCNIP3, LTBP1, ASAP1, PON2, LPCAT2, SCN9A, CPPED1, ZMIZ1, HEG1, ZNF608, SNAI2, WT1, HHIP, THBS1, TGM2, TIMP3, TLL1, ZNF391, ZNF43, TNS3, CDK15, CPEB4, FAT4, CDC42BPA, MORC4, TNFSF10, ADAMTS12, PDLIM1, RAD50, OPTN, MBNL2, CTDSPL, NUAK1 |  |  |  |  |  |  |  |  |  |  |  |  |  |  |  |  |  |  |  |  |  |  |  |
| Gmp Binding | 2 | | 50 | | 3.13E-04 | 0.011 | | GBP1, GBP3 |  |  |  |  |  |  |  |  |  |  |  |  |  |  |  |  |  |  |  |  |  |  |  |
| Protein Binding | 78 | | 0 | | 4.16E-04 | 0.010 | | ARRB1, ATP2B4, ABCA1, ADGRE5, CDH11, CLU, BMP4, BMP1, DPYSL2, DSP, TRIM6, CSF1, LPAR1, EFNB2, EYA4, F2RL1, EFEMP1, HLA-A, HLA-B, GATA6, GBP1, GBP2, GBP3, PRICKLE1, IGFBP4, INHBB, ITGB3, SH3RF2, MEF2C, MAP3K1, LAMA3, LCP1, LTBP1, SCN5A, SCP2, PTPRS, RAB27B, SOX4, SDC2, VEGFA, WT1, TGFA, TGFB2, THBS1, TGFBR3, TGM2, TIMP3, SERINC2, EVI5, GPRC5A, SORBS2, CASK, TNFSF10, PDLIM1, NOG, RAD50, OPTN, NUAK1, PLK2, MYL9, TDRD7, TIAM2, TRIB2, SNX10, KCNIP3, ASAP1, CLIC6, TENM3, BAIAP2L1, KLHL4, CEMIP, MTUS1, PMEPA1, HHIP, PORCN, CDK15, PARD6B, AFAP1L2 |  |  |  |  |  |  |  |  |  |  |  |  |  |  |  |  |  |  |  |  |  |  |  |
| Heparin Binding | 7 | | 3 | | 4.78E-04 | 0.019 | | VEGFA, CRISPLD2, BMP4, PTPRS, THBS1, TGFBR3, LIPG |  |  |  |  |  |  |  |  |  |  |  |  |  |  |  |  |  |  |  |  |  |  |  |
| Fibrinogen Binding | 2 | | 40 | | 5.19E-04 | 0.011 | | THBS1, ITGB3 |  |  |  |  |  |  |  |  |  |  |  |  |  |  |  |  |  |  |  |  |  |  |  |
| Cytokine Binding | 6 | | 4 | | 5.68E-04 | 0.019 | | ITGB3, GBP1, THBS1, TGFBR3, NOG, LTBP1 |  |  |  |  |  |  |  |  |  |  |  |  |  |  |  |  |  |  |  |  |  |  |  |
| Transforming Growth Factor Beta Binding | 3 | | 13 | | 6.02E-04 | 0.015 | | THBS1, TGFBR3, LTBP1 |  |  |  |  |  |  |  |  |  |  |  |  |  |  |  |  |  |  |  |  |  |  |  |
| Calcium Ion Binding | 15 | | 1 | | 6.66E-04 | 0.016 | | LPCAT2, MYL9, ADGRE5, CDH11, FAT4, BMP1, HEG1, LCP1, KCNIP3, LTBP1, FLG, THBS1, TGM2, TLL1, EFEMP1 |  |  |  |  |  |  |  |  |  |  |  |  |  |  |  |  |  |  |  |  |  |  |  |
| Molecular Adaptor Activity | 8 | | 3 | | 7.02E-04 | 0.019 | | ARRB1, DSP, TRIM6, RAD50, AFAP1L2, OPTN, SORBS2, SERINC2 |  |  |  |  |  |  |  |  |  |  |  |  |  |  |  |  |  |  |  |  |  |  |  |
| Transforming Growth Factor Beta Receptor Binding | 4 | | 7 | | 7.91E-04 | 0.017 | | INHBB, BMP4, TGFB2, TGFBR3 |  |  |  |  |  |  |  |  |  |  |  |  |  |  |  |  |  |  |  |  |  |  |  |
| Protein Binding, Bridging | 7 | | 3 | | 7.96E-04 | 0.019 | | RAD50, OPTN, ARRB1, DSP, SORBS2, TRIM6, SERINC2 |  |  |  |  |  |  |  |  |  |  |  |  |  |  |  |  |  |  |  |  |  |  |  |
| Nucleoside Monophosphate Kinase Activity | 3 | | 11 | | 8.70E-04 | 0.015 | | RAD50, CASK, AK4 |  |  |  |  |  |  |  |  |  |  |  |  |  |  |  |  |  |  |  |  |  |  |  |
| Peptide Antigen Binding | 4 | | 6 | | 1.37E-03 | 0.017 | | SLC7A5, HLA-A, HLA-B, HLA-DRA |  |  |  |  |  |  |  |  |  |  |  |  |  |  |  |  |  |  |  |  |  |  |  |
| Vascular Endothelial Growth Factor Receptor 2 Binding | 2 | | 25 | | 1.43E-03 | 0.011 | | VEGFA, ITGB3 |  |  |  |  |  |  |  |  |  |  |  |  |  |  |  |  |  |  |  |  |  |  |  |
| Transferase Activity, Transferring Acyl Groups | 8 | | 2 | | 1.60E-03 | 0.017 | | LPCAT2, ARRB1, PORCN, CPT1A, ELOVL7, SCP2, TGM2, F13A1 |  |  |  |  |  |  |  |  |  |  |  |  |  |  |  |  |  |  |  |  |  |  |  |
| Protein-Glutamine Gamma-Glutamyltransferase Activity | 2 | | 22 | | 1.83E-03 | 0.011 | | TGM2, F13A1 |  |  |  |  |  |  |  |  |  |  |  |  |  |  |  |  |  |  |  |  |  |  |  |
| Adenylate Kinase Activity | 2 | | 22 | | 1.83E-03 | 0.011 | | RAD50, AK4 |  |  |  |  |  |  |  |  |  |  |  |  |  |  |  |  |  |  |  |  |  |  |  |
| Type Ii Transforming Growth Factor Beta Receptor Binding | 2 | | 22 | | 1.83E-03 | 0.011 | | TGFB2, TGFBR3 |  |  |  |  |  |  |  |  |  |  |  |  |  |  |  |  |  |  |  |  |  |  |  |
| Carbohydrate Derivative Binding | 31 | | 1 | | 1.90E-03 | 0.012 | | PLK2, ATP2B4, PDE10A, ABCA1, MAMDC2, AK4, SCP2, BMP4, PTPRS, RAB27B, CEMIP, VEGFA, LPAR1, THBS1, TGFBR3, TGM2, ACSL1, FBP1, GBP1, GBP2, GBP3, CDK15, TRIB2, CDC42BPA, CASK, MAP3K1, CRISPLD2, LIPG, RAD50, CHST15, NUAK1 |  |  |  |  |  |  |  |  |  |  |  |  |  |  |  |  |  |  |  |  |  |  |  |
| Cell-Cell Adhesion Mediator Activity | 3 | | 8 | | 1.92E-03 | 0.014 | | PDLIM1, BAIAP2L1, DSP |  |  |  |  |  |  |  |  |  |  |  |  |  |  |  |  |  |  |  |  |  |  |  |
| Pdz Domain Binding | 5 | | 3 | | 2.31E-03 | 0.017 | | ATP2B4, LPAR1, SDC2, TGFBR3, CASK |  |  |  |  |  |  |  |  |  |  |  |  |  |  |  |  |  |  |  |  |  |  |  |
| Fibronectin Binding | 3 | | 8 | | 2.45E-03 | 0.014 | | THBS1, VEGFA, ITGB3 |  |  |  |  |  |  |  |  |  |  |  |  |  |  |  |  |  |  |  |  |  |  |  |
| Cytokine Activity | 7 | | 2 | | 3.20E-03 | 0.016 | | CSF1, INHBB, VEGFA, BMP4, BMP1, TGFB2, TNFSF10 |  |  |  |  |  |  |  |  |  |  |  |  |  |  |  |  |  |  |  |  |  |  |  |
| Insulin-Like Growth Factor I Binding | 2 | | 16 | | 3.31E-03 | 0.011 | | IGFBP4, ITGB3 |  |  |  |  |  |  |  |  |  |  |  |  |  |  |  |  |  |  |  |  |  |  |  |
| Binding | 127 | | 0 | | 3.42E-03 | 0.008 | | ARRB1, ATP2B4, ABCA1, AK4, CD22, ADGRE5, CDH11, CLU, BMP4, BMP1, CYP24A1, DPYSL2, DSP, CSF1, CTSD, FLG, LPAR1, EFNB2, EYA4, F2RL1, F13A1, ACSL1, EFEMP1, FBP1, GPR1, HLA-A, HLA-B, GATA6, GBP1, GBP2, GBP3, IGFBP4, INHBB, ITGB3, HLA-DRA, MEF2C, MEIS3P1, MAP3K1, LAMA3, LCP1, LTBP1, PON2, RRBP1, SCN5A, SCP2, SCN9A, PTPRS, RAB27B, SOX4, SDC2, SNAI2, VEGFA, WT1, TGFA, TGFB2, THBS1, TGFBR3, TGM2, TIMP3, TLL1, TLR3, SLC7A5, ZNF43, EVI5, GPRC5A, SORBS2, CDC42BPA, CASK, TNFSF10, PTGES, FCGR2C, PDLIM1, NOG, LIPG, RAD50, OPTN, MBNL2, CTDSPL, NUAK1, PLK2, PDE10A, MYL9, MGAT4A, TDRD7, TIAM2, TRIB2, CPA4, SNX10, KCNIP3, ASAP1, CLIC6, CHST15, SNX7, LPCAT2, RBM47, TENM3, BAIAP2L1, KLHL4, ACOXL, CPPED1, ZMIZ1, CEMIP, HEG1, MTUS1, ZNF608, PMEPA1, HHIP, TNS3, PORCN, CDK15, CPEB4, FAT4, MORC4, ADAMTS12, CRISPLD2, APOL6, PARD6B, AFAP1L2, MARCH3, TRIM6, OR5P2, PRICKLE1, SH3RF2, MAMDC2, ZNF391, SERINC2, MIR181B1 |  |  |  |  |  |  |  |  |  |  |  |  |  |  |  |  |  |  |  |  |  |  |  |
| Phosphatidylcholine Transporter Activity | 2 | | 15 | | 3.90E-03 | 0.011 | | ABCA1, SCP2 |  |  |  |  |  |  |  |  |  |  |  |  |  |  |  |  |  |  |  |  |  |  |  |
| Cell Adhesion Mediator Activity | 3 | | 6 | | 4.03E-03 | 0.014 | | PDLIM1, BAIAP2L1, DSP |  |  |  |  |  |  |  |  |  |  |  |  |  |  |  |  |  |  |  |  |  |  |  |
| Phosphotransferase Activity, Phosphate Group As Acceptor | 3 | | 6 | | 4.03E-03 | 0.014 | | RAD50, CASK, AK4 |  |  |  |  |  |  |  |  |  |  |  |  |  |  |  |  |  |  |  |  |  |  |  |
| Vascular Endothelial Growth Factor Receptor Binding | 2 | | 14 | | 4.53E-03 | 0.011 | | VEGFA, ITGB3 |  |  |  |  |  |  |  |  |  |  |  |  |  |  |  |  |  |  |  |  |  |  |  |
| Protein Domain Specific Binding | 14 | | 1 | | 5.46E-03 | 0.013 | | ATP2B4, SCN5A, SORBS2, CASK, RAB27B, MEF2C, SDC2, PMEPA1, ASAP1, AFAP1L2, WT1, LPAR1, TGFBR3, TGM2 |  |  |  |  |  |  |  |  |  |  |  |  |  |  |  |  |  |  |  |  |  |  |  |
| Coreceptor Activity | 3 | | 5 | | 5.78E-03 | 0.013 | | CD22, ITGB3, TGFBR3 |  |  |  |  |  |  |  |  |  |  |  |  |  |  |  |  |  |  |  |  |  |  |  |
| Nucleobase-Containing Compound Kinase Activity | 3 | | 5 | | 6.11E-03 | 0.013 | | RAD50, CASK, AK4 |  |  |  |  |  |  |  |  |  |  |  |  |  |  |  |  |  |  |  |  |  |  |  |
| Platelet-Derived Growth Factor Receptor Binding | 2 | | 11 | | 6.67E-03 | 0.010 | | VEGFA, ITGB3 |  |  |  |  |  |  |  |  |  |  |  |  |  |  |  |  |  |  |  |  |  |  |  |
| Transferase Activity, Transferring Amino-Acyl Groups | 2 | | 11 | | 6.67E-03 | 0.010 | | TGM2, F13A1 |  |  |  |  |  |  |  |  |  |  |  |  |  |  |  |  |  |  |  |  |  |  |  |
| Transferase Activity | 31 | | 1 | | 7.31E-03 | 0.011 | | LPCAT2, PLK2, ARRB1, AK4, MGAT4A, FBXO32, MARCH3, SCP2, TRIM6, CPT1A, UGCG, EFNB2, TGFBR3, TGM2, F13A1, EFEMP1, HAS2, PORCN, CDK15, ELOVL7, TRIB2, CDC42BPA, COLGALT1, CASK, SH3RF2, PTGES, MAP3K1, LTBP1, RAD50, CHST15, NUAK1 |  |  |  |  |  |  |  |  |  |  |  |  |  |  |  |  |  |  |  |  |  |  |  |
| Zinc Ion Binding | 14 | | 1 | | 7.37E-03 | 0.013 | | GATA6, PRICKLE1, MARCH3, BMP1, MORC4, TNFSF10, ZMIZ1, MAP3K1, CPA4, TRIM6, WT1, HHIP, TIMP3, TLL1 |  |  |  |  |  |  |  |  |  |  |  |  |  |  |  |  |  |  |  |  |  |  |  |
| Transforming Growth Factor Beta-Activated Receptor Activity | 2 | | 11 | | 7.47E-03 | 0.010 | | TGFBR3, LTBP1 |  |  |  |  |  |  |  |  |  |  |  |  |  |  |  |  |  |  |  |  |  |  |  |
| Cell Adhesion Molecule Binding | 10 | | 1 | | 8.02E-03 | 0.014 | | DSP, PDLIM1, LCP1, ASAP1, TENM3, CDH11, ITGB3, BAIAP2L1, GPRC5A, THBS1 |  |  |  |  |  |  |  |  |  |  |  |  |  |  |  |  |  |  |  |  |  |  |  |
| G-Protein Alpha-Subunit Binding | 3 | | 5 | | 8.31E-03 | 0.013 | | ADGRE5, LPAR1, F2RL1 |  |  |  |  |  |  |  |  |  |  |  |  |  |  |  |  |  |  |  |  |  |  |  |
| Cytokine Receptor Binding | 8 | | 2 | | 8.48E-03 | 0.014 | | CSF1, INHBB, VEGFA, ITGB3, BMP4, TGFB2, TGFBR3, TNFSF10 |  |  |  |  |  |  |  |  |  |  |  |  |  |  |  |  |  |  |  |  |  |  |  |
| Catalytic Activity | 65 | | 0 | | 9.13E-03 | 0.009 | | ARRB1, ATP2B4, ABCA1, AK4, FBXO32, MARCH3, CLU, BMP1, CYP24A1, DPYSL2, TRIM6, CPT1A, CTSD, EFNB2, EYA4, F13A1, ACSL1, EFEMP1, FBP1, HAS2, GBP1, GBP2, GBP3, ITGB3, SH3RF2, MAP3K1, COX7B2, LTBP1, PLPP4, PON2, TMPRSS15, SCP2, PTPRS, RAB27B, UGCG, TGFBR3, TGM2, TLL1, CDC42BPA, CASK, PTGES, LIPG, RAD50, CTDSPL, NUAK1, PLK2, PDE10A, MGAT4A, PTGR1, PRSS23, TRIB2, CPA4, CHST15, LPCAT2, TENM3, ACOXL, CPPED1, CEMIP, NXN, HHIP, PORCN, CDK15, ELOVL7, COLGALT1, ADAMTS12 |  |  |  |  |  |  |  |  |  |  |  |  |  |  |  |  |  |  |  |  |  |  |  |
| Voltage-Gated Sodium Channel Activity | 2 | | 10 | | 9.19E-03 | 0.010 | | SCN5A, SCN9A |  |  |  |  |  |  |  |  |  |  |  |  |  |  |  |  |  |  |  |  |  |  |  |
| Cadherin Binding Involved In Cell-Cell Adhesion | 2 | | 10 | | 9.19E-03 | 0.010 | | PDLIM1, BAIAP2L1 |  |  |  |  |  |  |  |  |  |  |  |  |  |  |  |  |  |  |  |  |  |  |  |
| Anion Binding | 34 | | 1 | | 1.01E-02 | 0.010 | | PLK2, ATP2B4, PDE10A, ABCA1, AK4, BMP4, LPAR1, ACSL1, FBP1, GBP1, GBP2, GBP3, TRIB2, MAP3K1, SNX10, ASAP1, CHST15, SCP2, ACOXL, PTPRS, RAB27B, CEMIP, VEGFA, THBS1, TGFBR3, TGM2, CDK15, CDC42BPA, CASK, PTGES, CRISPLD2, LIPG, RAD50, NUAK1 |  |  |  |  |  |  |  |  |  |  |  |  |  |  |  |  |  |  |  |  |  |  |  |
| Cholesterol Transporter Activity | 2 | | 9 | | 1.01E-02 | 0.010 | | ABCA1, SCP2 |  |  |  |  |  |  |  |  |  |  |  |  |  |  |  |  |  |  |  |  |  |  |  |
| Transferase Activity, Transferring Acyl Groups Other Than Amino-Acyl Groups | 6 | | 2 | | 1.19E-02 | 0.014 | | LPCAT2, ARRB1, PORCN, CPT1A, ELOVL7, SCP2 |  |  |  |  |  |  |  |  |  |  |  |  |  |  |  |  |  |  |  |  |  |  |  |
| Extracellular Matrix Binding | 3 | | 4 | | 1.24E-02 | 0.013 | | VEGFA, ITGB3, THBS1 |  |  |  |  |  |  |  |  |  |  |  |  |  |  |  |  |  |  |  |  |  |  |  |
| Transmembrane Receptor Protein Serine/Threonine Kinase Activity | 2 | | 8 | | 1.31E-02 | 0.010 | | TGFBR3, LTBP1 |  |  |  |  |  |  |  |  |  |  |  |  |  |  |  |  |  |  |  |  |  |  |  |
| Nitric-Oxide Synthase Binding | 2 | | 8 | | 1.31E-02 | 0.010 | | ATP2B4, SCN5A |  |  |  |  |  |  |  |  |  |  |  |  |  |  |  |  |  |  |  |  |  |  |  |
| Phosphatidylserine-Translocating Atpase Activity | 1 | | 50 | | 1.45E-02 | 0.006 | | ABCA1 |  |  |  |  |  |  |  |  |  |  |  |  |  |  |  |  |  |  |  |  |  |  |  |
| Dihydropyrimidinase Activity | 1 | | 50 | | 1.45E-02 | 0.006 | | DPYSL2 |  |  |  |  |  |  |  |  |  |  |  |  |  |  |  |  |  |  |  |  |  |  |  |
| Apolipoprotein A-I Receptor Activity | 1 | | 50 | | 1.45E-02 | 0.006 | | ABCA1 |  |  |  |  |  |  |  |  |  |  |  |  |  |  |  |  |  |  |  |  |  |  |  |
| Microfibril Binding | 1 | | 50 | | 1.45E-02 | 0.006 | | LTBP1 |  |  |  |  |  |  |  |  |  |  |  |  |  |  |  |  |  |  |  |  |  |  |  |
| Vascular Endothelial Growth Factor Receptor 1 Binding | 1 | | 50 | | 1.45E-02 | 0.006 | | VEGFA |  |  |  |  |  |  |  |  |  |  |  |  |  |  |  |  |  |  |  |  |  |  |  |
| Ceramide Glucosyltransferase Activity | 1 | | 50 | | 1.45E-02 | 0.006 | | UGCG |  |  |  |  |  |  |  |  |  |  |  |  |  |  |  |  |  |  |  |  |  |  |  |
| N-Acetylgalactosamine 4-Sulfate 6-O-Sulfotransferase Activity | 1 | | 50 | | 1.45E-02 | 0.006 | | CHST15 |  |  |  |  |  |  |  |  |  |  |  |  |  |  |  |  |  |  |  |  |  |  |  |
| Voltage-Gated Sodium Channel Activity Involved In Purkinje Myocyte Action Potential | 1 | | 50 | | 1.45E-02 | 0.006 | | SCN5A |  |  |  |  |  |  |  |  |  |  |  |  |  |  |  |  |  |  |  |  |  |  |  |
| Nucleoside Triphosphate Adenylate Kinase Activity | 1 | | 50 | | 1.45E-02 | 0.006 | | AK4 |  |  |  |  |  |  |  |  |  |  |  |  |  |  |  |  |  |  |  |  |  |  |  |
| Alpha-1A Adrenergic Receptor Binding | 1 | | 50 | | 1.45E-02 | 0.006 | | ARRB1 |  |  |  |  |  |  |  |  |  |  |  |  |  |  |  |  |  |  |  |  |  |  |  |
| D4 Dopamine Receptor Binding | 1 | | 50 | | 1.45E-02 | 0.006 | | CLIC6 |  |  |  |  |  |  |  |  |  |  |  |  |  |  |  |  |  |  |  |  |  |  |  |
| V2 Vasopressin Receptor Binding | 1 | | 50 | | 1.45E-02 | 0.006 | | ARRB1 |  |  |  |  |  |  |  |  |  |  |  |  |  |  |  |  |  |  |  |  |  |  |  |
| Fructose 1,6-Bisphosphate 1-Phosphatase Activity | 1 | | 50 | | 1.45E-02 | 0.006 | | FBP1 |  |  |  |  |  |  |  |  |  |  |  |  |  |  |  |  |  |  |  |  |  |  |  |
| Protein Kinase C Binding | 3 | | 4 | | 1.45E-02 | 0.012 | | PARD6B, ITGB3, DSP |  |  |  |  |  |  |  |  |  |  |  |  |  |  |  |  |  |  |  |  |  |  |  |
| Signaling Receptor Binding | 25 | | 1 | | 1.67E-02 | 0.011 | | ARRB1, ABCA1, CLU, SCP2, BMP4, BMP1, CSF1, VEGFA, EFNB2, TGFA, TGFB2, THBS1, TGFBR3, F2RL1, EFEMP1, HLA-A, HLA-B, IGFBP4, INHBB, ITGB3, CASK, TNFSF10, LAMA3, LCP1, CLIC6 |  |  |  |  |  |  |  |  |  |  |  |  |  |  |  |  |  |  |  |  |  |  |  |
| Voltage-Gated Ion Channel Activity | 5 | | 2 | | 1.69E-02 | 0.013 | | CLIC6, CLIC3, SCN5A, SCN9A, KCNIP3 |  |  |  |  |  |  |  |  |  |  |  |  |  |  |  |  |  |  |  |  |  |  |  |
| Voltage-Gated Channel Activity | 5 | | 2 | | 1.69E-02 | 0.013 | | CLIC6, CLIC3, SCN5A, SCN9A, KCNIP3 |  |  |  |  |  |  |  |  |  |  |  |  |  |  |  |  |  |  |  |  |  |  |  |
| Insulin-Like Growth Factor Binding | 2 | | 7 | | 1.76E-02 | 0.010 | | IGFBP4, ITGB3 |  |  |  |  |  |  |  |  |  |  |  |  |  |  |  |  |  |  |  |  |  |  |  |
| Mitogen-Activated Protein Kinase Kinase Binding | 3 | | 3 | | 1.81E-02 | 0.012 | | ARRB1, MAP3K1, TRIB2 |  |  |  |  |  |  |  |  |  |  |  |  |  |  |  |  |  |  |  |  |  |  |  |
